# Supplementary material for: Enantioselective access to tricyclic tetrahydropyran derivatives by a remote hydrogen bonding mediated intramolecular IEDHDA reaction
Source: Nat Commun. 2021 Dec 10;12:7188. doi: 10.1038/s41467-021-27521-z (PMC8664811; doi:10.1038/s41467-021-27521-z)
Supplement: Supplementary file 1 — Supplementary Information [file 41467_2021_27521_MOESM1_ESM.pdf]

Supplementary Information for

Enantioselective Access to Tricyclic Tetrahydropyran Derivatives by a Remote Hydrogen Bonding Mediated Intramolecular IEDHDA Reaction

Min Jin, [1,6] Congyun Tang, [2,6] Yingying Li, [3] Shuai Yang, [1] Ying-Tao Yang, [1] Lin Peng, [2] Xiao-Nian Li, [1] Wenjing Zhang, *, [4] Zhili Zuo,* [1] Fabien Gagosz [5] & Liang-Liang Wang*, [1]

[1] State Key Laboratory of Phytochemistry and Plant Resources in West China, Kunming Institute of Botany, Chinese Academy of Sciences, Kunming, 650201, PR China. [2] School of Food and Chemical Engineering, Shaoyang University, Shaoyang, 422000, PR China. [3] School of Chemical Engineering, Sichuan University of Science & Engineering, Zigong, 643000, PR China. [4] College of Chemistry and Molecular Engineering, Zhengzhou University, Zhengzhou, Henan Province 450001, PR China. [5] Department of Chemistry and Biomolecular Sciences, University of Ottawa, K1N 6N5, Ottawa, Canada. [6] These authors contributed equally: Min Jin, Congyun Tang.

* Corresponding Author. Email: zhangwj@zzu.edu.cn; zuozhili@mail.kib.ac.cn; wangliangliang@maik.kib.ac.cn.

Table of Contents

Supplementary Notes……...S2

Supplementary Methods..S2

Supplementary Discussion...S65

Supplementary References..S277

1. Supplementary Notes

NMR spectra were recorded with tetramethylsilane as the internal standard. ^{1}H NMR spectra were recorded at 400 MHz、500 MHz、600 MHz or 800 MHz, respectively, ^{13}C NMR spectra were recorded at 100 MHz、125 MHz、150 MHz or 200 MHz (Bruker Avance), and ^{19}F NMR spectra were recorded at 471 MHz、376 MHz (Bruker Avance) respectively, by using $CDCl_3$ or acetone as internal references: the solvent ($CDCl_3$: δ_H = 7.26 ppm; δ_C = 77.16 ppm; acetone: δ_H = 2.05 ppm; δ_C = 29.84 ppm). Chemical shift values of NMR data are reported as values in ppm relative to (residual undeuterated) solvent signal as internal standard. Multiplicities for ^{1}H NMR signals are described using the following abbreviations: s = singlet, d = doublet, t = triplet, q = quartet, m = multiplet, br = broad. High resolution mass spectra were obtained with the Q-TOF-Premier mass spectrometer. Enantiomeric excess was determined by HPLC analysis on chiralpak AD-H, or OD-H columns. Optical rotations are reported as follows: $[\alpha]_D^{25}$ (589 nm, c = 1 corresponding to 10 mg ml^{-1}, DCM or Acetone).

All reactions were performed under an argon atmosphere unless otherwise stated. Glassware was dried by heating under vacuum followed by flashing with argon gas prior to use. Solvents was dried and purified by standard procedures as specified in Purification of Laboratory Chemicals, 4th Ed (Armarego, W. L. F.; Perrin, D. D. Butterworth Heinemann: 1997) or purchased from commercial providers. 4Å molecular sieves were activated by high temperature of 210 degrees Celsius for 24 hours to use.

2. Supplementary Methods

2.1 Synthesis and characterization of substrates **5a-t**

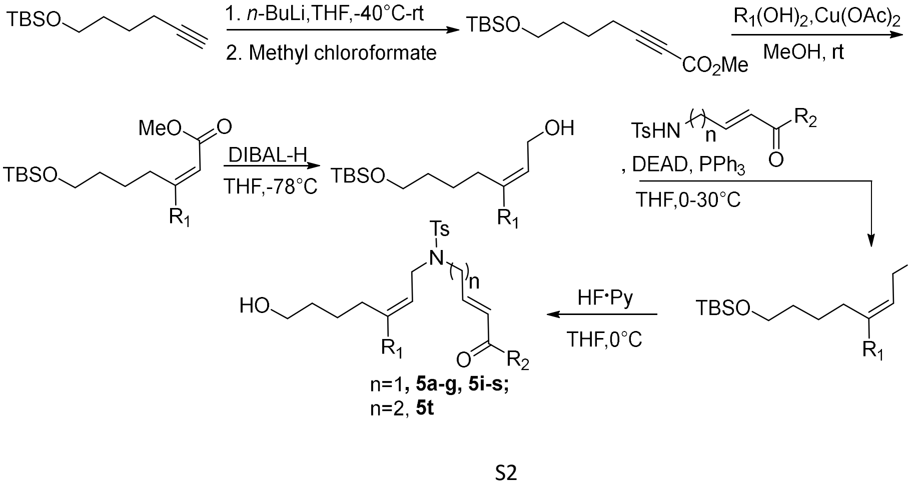

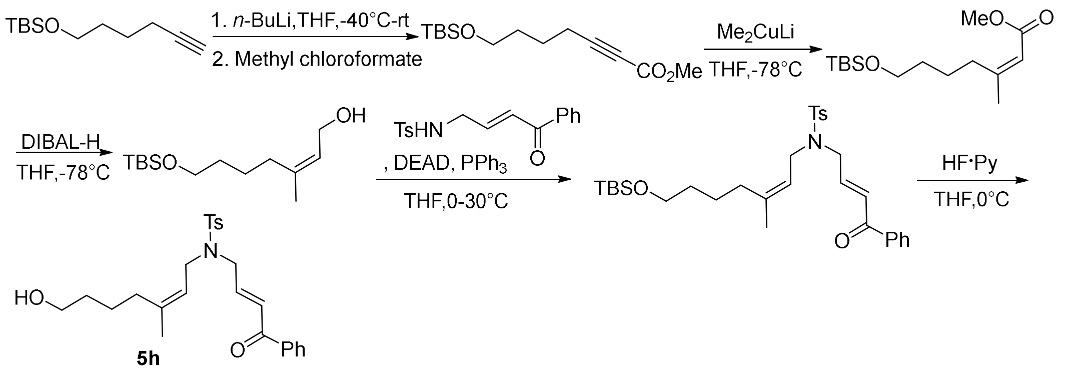

5h

***N*-((*E*))-7-hydroxy-3-phenylhept-2-en-1-yl)-4-methyl-*N*-((*E*))-4-oxo-4-phenylbut-2-en-1-yl)benzenesulfonamide (5a)**

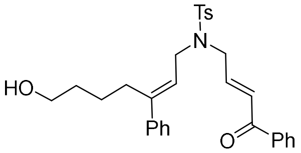

Colorless oil. **^{1}H NMR** (400 MHz, Chloroform-*d*) δ 7.77 (d, J = 7.7 Hz, 2H), 7.67 (d, J = 8.0 Hz, 2H), 7.48 (t, J = 7.5 Hz, 1H), 7.35 (t, J = 7.6 Hz, 2H), 7.22 (d, J = 8.0 Hz, 2H), 7.16 (d, J = 6.8 Hz, 3H), 7.11 – 7.05 (m, 2H), 6.94 (d, J = 15.5 Hz, 1H), 6.77 (dt, J = 15.4, 5.1 Hz, 1H), 5.35 (t, J = 7.0 Hz, 1H), 4.03 (d, J = 5.4 Hz, 2H), 3.98 (d, J = 7.0 Hz, 2H), 3.43 (t, J = 6.3 Hz, 2H), 2.35 (d, J = 7.9 Hz, 2H), 2.31 (s, 3H), 1.38 (p, J = 6.7 Hz, 2H), 1.31 – 1.17 (m, 2H). **^{13}C NMR** (101 MHz, Chloroform-*d*) δ 190.29, 145.43, 143.85, 142.81, 137.33, 137.11, 133.28, 130.05, 129.90, 128.79 (2C), 128.48, 127.63, 127.39, 126.43, 125.51, 122.08, 62.50, 48.33, 45.76, 32.47, 29.61, 24.99, 21.66. **HRMS** (ESI): m/z Calcd. For $C_{30}H_{33}NO_4SNa$ ([M+Na]$^+$): 526.2023, found: 526.2021.

***N*-((*E*))-3-(4-chlorophenyl)-7-hydroxyhept-2-en-1-yl)-4-methyl-*N*-((*E*))-4-oxo-4-phenylbut-2-en-1-yl)benzenesulfonamide (5b)**

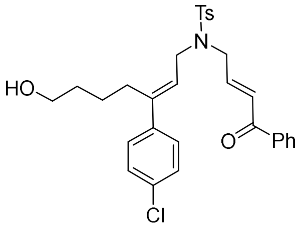

Colorless oil. **^{1}H NMR** (400 MHz, Chloroform-*d*) δ 7.90 (d, J=7.4Hz, 2H), 7.80 (d, J = 8.0 Hz, 2H), 7.63 (t, J = 7.4 Hz, 1H), 7.50 (t, J = 7.6 Hz, 2H), 7.37 (d, J = 8.0 Hz, 2H), 7.27 (d, J = 8.2 Hz, 2H), 7.15 (d, J = 8.3 Hz, 2H), 7.07 (d, J = 15.5 Hz, 1H), 6.90 (dt, J = 15.5, 5.2 Hz, 1H), 5.50 (t, J = 6.9 Hz, 1H), 4.17 (d, J =4.8Hz, 2H), 4.10 (d, J = 6.9 Hz, 2H), 3.58 (t, J = 6.3 Hz, 2H), 2.47 (m, 5H), 1.50 (m, 2H), 1.38 (m, 3H). **^{13}C NMR** (101 MHz, Chloroform-*d*) δ 190.18, 144.12, 143.89, 142.71, 140.06, 137.23, 136.94, 133.36, 133.30, 130.03, 128.76, 128.72,

128.58, 127.70, 127.59, 127.34, 122.80, 62.34, 48.56, 45.84, 32.32, 29.49, 24.88, 21.62. **HRMS** (ESI): m/z Calcd. For $C_{30}H_{32}ClNO_4SH$ ([M+H]$^+$): 538.1813, found: 538.1807.

***N*-((*E*))-3-(3-chlorophenyl)-7-hydroxyhept-2-en-1-yl)-4-methyl-*N*-((*E*))-4-oxo-4-phenylbut-2-en-1-yl)benzenesulfonamide (5c)**

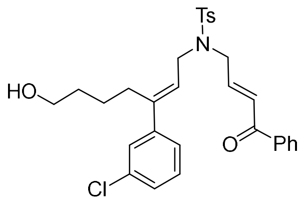

Colorless oil. **^{1}H NMR** (400 MHz, Chloroform-*d*) δ 8.00 – 7.93 (m, 2H), 7.85 (d, *J* = 8.0 Hz, 2H), 7.68 (t, *J* = 7.4 Hz, 1H), 7.55 (t, *J* = 7.6 Hz, 2H), 7.42 (d, *J* = 8.0 Hz, 2H), 7.39 – 7.24 (m, 2H), 7.22 – 7.09 (m, 3H), 6.95 (dt, *J* = 15.4, 5.1 Hz, 1H), 5.51 (t, *J* = 6.8 Hz, 1H), 4.25 – 4.18 (m, 2H), 4.16 (d, *J* = 6.9 Hz, 2H), 3.63 (t, *J* = 6.3 Hz, 2H), 2.51 (d, *J* = 4.5 Hz, 5H), 1.62 – 1.50 (m, 2H), 1.44 (td, *J* = 8.5, 4.1 Hz, 2H). **^{13}C NMR** (101 MHz, Chloroform-*d*) *δ* 190.20, 144.04, 143.99, 143.57, 142.63, 137.26, 137.05, 134.36, 133.32, 130.08, 129.71, 128.79, 128.76, 127.65, 127.62, 127.35, 126.55, 124.55, 123.26, 62.36, 48.51, 45.69, 32.34, 29.52, 24.91, 21.66. **HRMS** (ESI): m/z Calcd. For $C_{30}H_{32}ClNO_4SNa$ ([M+Na]$^+$): 560.1633, found: 560.1635.

***N*-((*E*))-3-(4-bromophenyl)-7-hydroxyhept-2-en-1-yl)-4-methyl-*N*-((*E*))-4-oxo-4-phenylbut-2-en-1-yl)benzenesulfonamide (5d)**

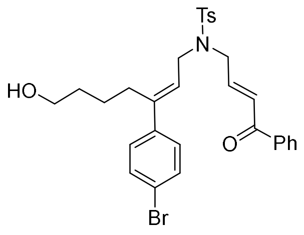

Colorless oil. **^{1}H NMR** (400 MHz, Chloroform-*d*) *δ* 7.84 (d, *J*=7.5Hz, 2H), 7.73 (d, *J* = 8.1 Hz, 2H), 7.57 (t, *J* = 7.4 Hz, 1H), 7.44 (t, *J* = 7.6 Hz, 2H), 7.37 (d, *J* = 1.9 Hz, 2H), 7.30 (d, *J* = 8.0 Hz, 2H), 7.02 (m, 3H), 6.83 (dt, *J* = 15.4, 5.2 Hz, 1H), 5.43 (t, *J* = 7.0 Hz, 1H), 4.10 (d, *J* = 5.3 Hz, 2H), 4.03 (d, *J* = 6.9 Hz, 2H), 3.51 (t, *J* = 6.3 Hz, 2H), 2.41 (m, 5H), 1.43 (m, 2H), 1.31 (m, 2H). **^{13}C NMR** (101 MHz, Chloroform-*d*) *δ* 190.17, 144.16, 143.89, 142.69, 140.54, 137.22, 136.93, 133.31, 131.53, 130.03, 128.77, 128.72, 128.04, 127.59, 127.33, 122.86, 121.51, 62.33, 48.56, 45.84, 32.31, 29.44, 24.87, 21.63. **HRMS** (ESI): m/z Calcd. For $C_{30}H_{32}BrNO_4SNa$ ([M+Na]$^+$): 604.1133, found: 604.1130.

N-((*E*))-7-hydroxy-3-(3-methoxyphenyl)hept-2-en-1-yl)-4-methyl-*N*-((*E*))-4-oxo-4-phenylbut-2-en-1-yl)benzenesulfonamide (5e)

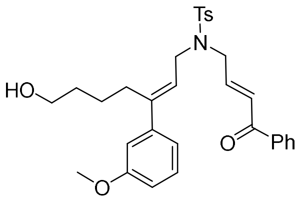

Colorless oil. **^{1}H NMR** (400 MHz, Chloroform-*d*) δ 7.89 – 7.82 (m, 2H), 7.75 (d, J = 8.0 Hz, 2H), 7.57 (t, J = 7.6 Hz, 1H), 7.44 (t, J = 7.6 Hz, 2H), 7.31 (d, J = 8.0 Hz, 2H), 7.17 (t, J = 7.9 Hz, 1H), 7.03 (dd, J = 15.5, 1.8 Hz, 1H), 6.85 (dt, J = 15.6, 5.2 Hz, 1H), 6.82 – 6.73 (m, 2H), 6.70 (t, J = 2.1 Hz, 1H), 5.43 (t, J = 6.9 Hz, 1H), 4.14 – 4.09 (m, 2H), 4.06 (d, J = 7.0 Hz, 2H), 3.76 (s, 3H), 3.52 (t, J = 6.3 Hz, 2H), 2.41 (d, J = 5.4 Hz, 5H), 1.52 – 1.40 (m, 2H), 1.34 (ddt, J = 15.1, 9.8, 5.8 Hz, 2H). **^{13}C NMR** (101 MHz, Chloroform-*d*) δ 190.22, 159.65, 145.26, 143.85, 143.21, 142.75, 137.29, 137.07, 133.25, 130.03, 129.40, 128.75 (2C), 127.59, 127.34, 122.10, 118.89, 112.60, 112.53, 62.44, 55.30, 48.32, 45.72, 32.44, 29.66, 24.98, 21.61. **HRMS** (ESI): m/z Calcd. For $C_{31}H_{35}O_5SNa$ ([M+Na]$^+$): 556.2128, found: 556.2125.

N-((*E*))-7-hydroxy-3-(2-methoxyphenyl)hept-2-en-1-yl)-4-methyl-*N*-((*E*))-4-oxo-4-phenylbut-2-en-1-yl)benzenesulfonamide (5f)

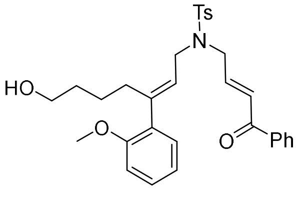

Light yellow oil. **^{1}H NMR** (400 MHz, Chloroform-*d*) δ 7.91 – 7.84 (m, 2H), 7.75 (d, J = 8.0 Hz, 2H), 7.57 (t, J = 7.4 Hz, 1H), 7.45 (t, J = 7.6 Hz, 2H), 7.29 (d, J = 8.0 Hz, 2H), 7.21 (td, J = 7.8, 1.9 Hz, 1H), 7.10 – 7.00 (m, 1H), 6.95 – 6.86 (m, 2H), 6.83 (dd, J = 14.6, 6.5 Hz, 2H), 5.23 (t, J = 7.0 Hz, 1H), 4.17 – 4.10 (m, 2H), 4.05 (d, J = 7.1 Hz, 2H), 3.72 (s, 3H), 3.49 (t, J = 6.4 Hz, 2H), 2.41 (d, J = 10.3 Hz, 5H), 1.41 (q, J = 7.0 Hz, 2H), 1.24 (q, J = 9.1, 8.0 Hz, 2H). **^{13}C NMR** (101 MHz, Chloroform-*d*) δ 190.41, 156.50, 145.39, 143.70, 142.97, 137.39, 137.11, 133.21 , 131.98, 130.12, 129.97, 128.79, 128.75, 128.64, 127.73, 127.37, 123.20, 120.53, 110.66, 62.56, 55.40, 47.94, 45.21, 32.57, 30.28, 24.61, 21.63. **HRMS** (ESI): m/z Calcd. For $C_{31}H_{35}NO_5SNa$ ([M+Na]$^+$): 556.2128, found: 556.2125.

***N*-((*E*))-7-hydroxy-3-(*m*-tolyl)hept-2-en-1-yl)-4-methyl-*N*-((*E*))-4-oxo-4-phenyl-but-2-en-1-yl)benzenesulfonamide (5g)**

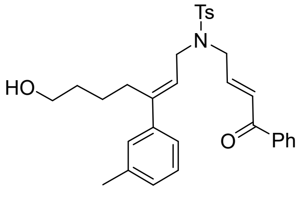

Colorless oil. ^{1}**H NMR** (400 MHz, Chloroform-*d*) *δ* 7.87 (d, *J* = 7.1 Hz, 2H), 7.76 (d, *J* = 8.0 Hz, 2H), 7.57 (t, *J* =7.4Hz, 1H), 7.45 (t, *J* = 7.6 Hz, 2H), 7.31 (d, *J* = 8.0 Hz, 2H), 7.15 (t, *J* = 7.9 Hz, 1H), 7.06 (d, *J* = 1.7 Hz, 1H), 7.03 (d, *J* = 8.8 Hz, 1H), 6.97 (d, *J* = 5.7 Hz, 2H), 6.86 (dt, *J* = 15.4, 5.1 Hz, 1H), 5.41 (t, *J* = 7.0 Hz, 1H), 4.12 (d, *J* = 5.2Hz, 2H), 4.07 (d, *J* = 7.1 Hz, 2H), 3.53 (t, *J* = 6.4 Hz, 2H), 2.42 (m, 5H), 2.29 (s, 3H), 1.49 – 1.43 (m, 2H), 1.38 – 1.30 (m, 2H). ^{13}**C NMR** (101 MHz, Chloroform-*d*) *δ* 190.24, 145.54, 143.78, 142.84, 141.69, 138.00, 137.31, 137.12, 133.25, 130.02, 128.77 (2C), 128.36, 128.31, 127.56, 127.37, 127.17, 123.47, 121.80, 62.49, 48.31, 45.79, 32.47, 29.62, 25.00, 21.65, 21.55. **HRMS** (ESI): m/z Calcd. For $C_{31}H_{35}NO_4SNa$ ($[M+Na]^+$): 540.2179, found: 540.2177.

***N*-((Z))-7-hydroxy-3-methylhept-2-en-1-yl)-4-methyl-*N*-((E))-4-oxo-4-phenylbut-2-en-1-yl)benzenesulfonamide (5h)**

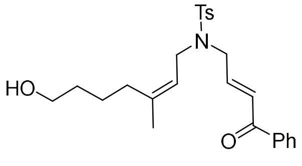

Colorless oil. ^{1}**H NMR** (400 MHz, Chloroform-*d*) *δ* 7.92 – 7.84 (m, 2H), 7.72 (d, *J* = 7.9 Hz, 2H), 7.58 (t, *J* = 7.4 Hz, 1H), 7.47 (t, *J* = 7.6 Hz, 2H), 7.30 (d, *J* = 8.1 Hz, 2H), 7.06 – 6.94 (m, 1H), 6.83 (dt, *J* = 15.3, 5.2 Hz, 1H), 5.06 (t, *J* = 7.3 Hz, 1H), 4.00 (dd, *J* = 5.4, 1.6 Hz, 2H), 3.84 (d, *J* = 7.0 Hz, 2H), 3.59 (t, *J* = 6.3 Hz, 2H), 2.41 (s, 3H), 1.96 (t, *J* = 7.5 Hz, 2H), 1.56 (s, 3H), 1.51 – 1.40 (m, 2H), 1.42 – 1.30 (m, 2H). ^{13}**C NMR** (101 MHz, Chloroform-*d*) *δ* 190.15, 143.66, 143.32, 141.45, 137.40, 136.95, 133.25, 129.93, 128.79, 128.76, 127.39, 127.20, 118.60, 62.83, 48.42, 45.94, 39.31, 32.41, 23.80, 21.66, 16.32. **HRMS** (ESI): m/z Calcd. For $C_{25}H_{31}NO_4SNa$ ($[M+Na]^+$): 464.1866, found: 464.1863.

***N*-((*E*)-7-hydroxy-3-phenylhept-2-en-1-yl)-4-methyl-*N*-((*E*)-4-oxo-(*p*-toly)but-2-en-1-yl)benzenesulfonamide (5i)**

Colorless oil. ^{1}**H NMR** (400 MHz, Chloroform-*d*) *δ* 7.78 (dd, *J* = 9.9, 8.0 Hz, 4H), 7.32 (d, *J* = 8.0 Hz, 2H), 7.32 – 7.21 (m, 5H), 7.19 (dd, *J* = 7.5, 2.2 Hz, 2H), 7.04 (d, *J* =

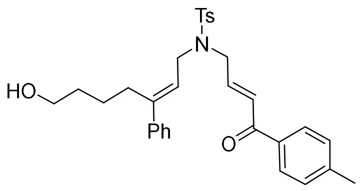

15.5 Hz, 1H), 6.85 (dt, J = 15.3, 5.2 Hz, 1H), 5.45 (t, J = 6.9 Hz, 1H), 4.17 – 4.12 (m, 2H), 4.09 (d, J = 7.0 Hz, 2H), 3.53 (t, J = 6.4 Hz, 2H), 2.47 (d, J = 7.8 Hz, 2H), 2.43 (d, J = 3.9 Hz, 6H), 1.47 (q, J = 6.8, 6.4 Hz, 2H), 1.39 – 1.32 (m，2H). **^{13}C NMR** (101 MHz, Chloroform-*d*) δ 189.79, 145.42, 144.23, 143.81, 142.20, 141.71, 137.17, 134.79, 130.04, 129.50, 128.95, 128.47, 127.69, 127.61, 127.39, 126.43, 122.10, 62.49, 48.28, 45.67, 32.47, 29.61, 24.99, 21.84, 21.65. **HRMS** (ESI): m/z Calcd. For $C_{31}H_{35}NO_4SNa$ ([M+Na]$^+$): 540.2179, found [M+Na]$^+$: 540.2179.

***N*-((*E*)-7-hydroxy-3-phenylhept-2-en-1-yl)-4-methyl-*N*-((*E*)-4-oxo-(*o*-toly)but-2-en-1-yl)benzenesulfonamide (5j)**

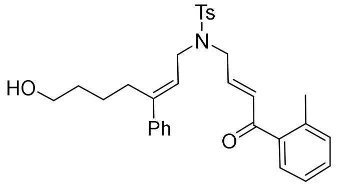

Colorless oil. **^{1}H NMR** (400 MHz, Chloroform-*d*) δ 7.76 (d, J = 8.0 Hz, 2H), 7.39 (td, J = 7.5, 1.5 Hz, 1H), 7.34 (d, J = 7.8 Hz, 3H), 7.32 – 7.27 (m, 4H), 7.23 – 7.29 (m, 3H), 6.68 (d, J = 15.9 Hz, 1H), 6.60 (dt, J = 15.8, 5.0 Hz, 1H), 5.45 (t, J = 7.0 Hz, 1H), 4.09 (d, J = 5.0 Hz, 2H), 4.07 (d, J = 7.0 Hz, 2H), 3.59 (t, J = 6.3 Hz, 2H), 2.50 (d, J = 7.8 Hz, 2H), 2.46 (s, 3H), 2.40 (s, 3H), 1.52 (dt, J = 12.6, 6.4 Hz, 2H), 1.44 – 1.36 (m, 2H). **^{13}C NMR** (101 MHz, Chloroform-*d*) δ 195.69, 145.37, 144.04, 143.83, 141.65, 138.01, 137.41, 136.95, 131.93, 131.56, 130.99, 130.02, 128.47 (2C), 127.65, 127.33, 126.41, 125.58, 122.05, 62.49, 48.46, 45.98, 32.48, 29.63, 24.99, 21.66, 20.42. **HRMS** (ESI): m/z Calcd. For $C_{31}H_{35}NO_4S$ Na ([M+Na]$^+$): 540.2179, found [M+Na]$^+$: 540.2178.

***N*-((*E*)-4-(4-ethylphenyl)-4-oxobut-2-en-1-yl)-*N*-((*E*)-7-hydroxy-3-phenylhept-2-en-1-yl)-4-methylbenzenesulfonamide (5k)**

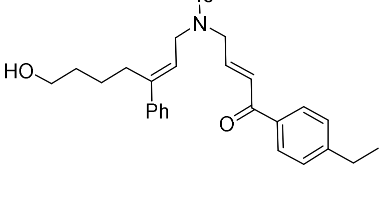

Colorless oil. **^{1}H NMR** (400 MHz, Chloroform-*d*) δ 7.83 (d, J = 8.0 Hz, 2H), 7.78 (d, J = 8.0 Hz, 2H), 7.34 (d, J = 8.0 Hz, 2H), 7.30 (d, J = 7.8 Hz, 3H), 7.27 (s, 2H), 7.20 (dd, J = 7.5, 2.2 Hz, 2H), 7.10 – 7.00 (m, 1H), 6.86 (dt, J = 15.4, 5.2 Hz, 1H), 5.46 (t, J = 7.0 Hz, 1H), 4.15 (dd, J = 5.2, 1.7 Hz, 2H), 4.10 (d, J = 7.0 Hz, 2H), 3.55 (t, J = 6.3 Hz, 2H), 2.74 (q, J = 7.6 Hz, 2H), 2.47 (t, J =

7.7 Hz, 2H), 2.44 (s, 3H), 1.55 – 1.44 (m, 2H), 1.45 – 1.30 (m, 2H), 1.29 (t, *J* = 7.6 Hz, 3H). **^{13}C NMR** (101 MHz, Chloroform-*d*) *δ* 189.90, 150.48, 145.48, 143.89, 142.28, 141.79, 137.26, 135.07, 130.12, 129.14, 128.55, 128.40, 127.79, 127.69, 127.47, 126.52, 122.20, 62.58, 48.38, 45.76, 32.56, 29.70, 29.21, 25.08, 21.75, 15.43. **HRMS** (ESI): m/z Calcd. For $C_{32}H_{37}NO_4SNa$ ([M+Na]$^+$): 554.2336, found [M+Na]$^+$: 554.2334.

***N*-((*E*)-7-hydroxy-3-phenylhept-2-en-1-yl)-*N*-((*E*)-4-(4-methoxyphenyl)-4-oxobut-2-en-1-yl) 4-methylbenzenesulfonamide (5l)**

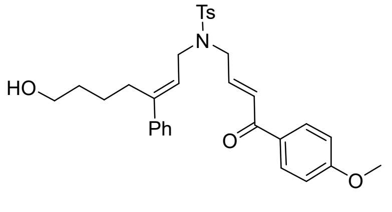

Colorless oil. **^{1}H NMR** (400 MHz, Chloroform-*d*) *δ* 7.90 – 7.82 (m, 2H), 7.74 (d, *J* = 8.0 Hz, 2H), 7.29 (d, *J* = 8.0 Hz, 2H), 7.24 (d, *J* = 6.3 Hz, 3H), 7.15 (dd, *J* = 7.5, 2.1 Hz, 2H), 7.02 (dt, *J* = 15.3, 1.8 Hz, 1H), 6.94 – 6.87 (m, 2H), 6.80 (dt, *J* = 15.4, 5.2 Hz, 1H), 5.42 (t, *J* = 7.0 Hz, 1H), 4.13 – 4.07 (m, 2H), 4.05 (d, *J* = 7.0 Hz, 2H), 3.86 (s, 3H), 3.51 (t, *J* = 6.3 Hz, 2H), 2.42 (d, *J* = 15.7 Hz, 2H), 2.40 (s, 3H), 1.51 – 1.39 (m, 2H), 1.39 – 1.24 (m, 2H). **^{13}C NMR** (101 MHz, Chloroform-*d*) *δ* 188.53, 163.81, 145.36, 143.79, 141.70, 141.63, 137.18, 131.17, 130.23, 130.02, 128.46, 127.59, 127.50, 127.38, 126.43, 122.13, 114.01, 62.47, 55.65, 48.29, 45.66, 32.47, 29.60, 24.99, 21.65. **HRMS** (ESI): m/z Calcd. For $C_{31}H_{35}NO_5SNa$ ([M+Na]$^+$): 556.2134, found[M+Na]$^+$: 556.2131.

***N*-((*E*)-7-hydroxy-3-phenylhept-2-en-1-yl)-*N*-((*E*)-4-(3-methoxyphenyl)-4-oxobut-2-en-1-yl) 4-methylbenzenesulfonamide (5m)**

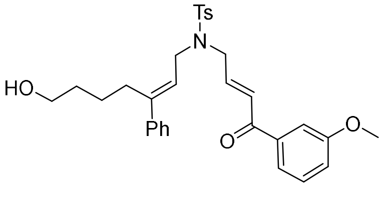

Colorless oil. **^{1}H NMR** ^{1}H NMR (400 MHz, Chloroform-*d*) *δ* 7.89 – 7.82 (m, 2H), 7.75 (d, *J* = 8.0 Hz, 2H), 7.57 (t, *J* = 7.5 Hz, 1H), 7.44 (t, *J* = 7.7 Hz, 2H), 7.31 (d, *J* = 8.0 Hz, 2H), 7.17 (t, *J* = 7.9 Hz, 1H), 7.03 (dd, *J* = 15.5, 1.8 Hz, 1H), 6.85 (dt, *J* = 15.5, 5.2 Hz, 1H), 6.77 (td, *J* = 6.9, 5.6, 2.0 Hz, 2H), 6.70 (t, *J* = 2.0 Hz, 1H), 5.43 (t, *J* = 6.9 Hz, 1H), 4.16 – 4.09 (m, 2H), 4.06 (d, *J* = 7.0 Hz, 2H), 3.76 (s, 3H), 3.52 (t, *J* = 6.3 Hz, 2H), 2.46 – 2.38 (m, 5H), 1.54 – 1.40 (m, 2H), 1.35 (td, *J* = 8.4, 4.0 Hz, 2H). **^{13}C NMR** (101 MHz, Chloroform-*d*) *δ* 190.23, 159.66, 145.27, 143.86, 143.22, 142.76, 137.30, 137.07, 133.25, 130.04,

129.41, 128.76 (2C), 127.60, 127.35, 122.11, 118.90, 112.61, 112.53, 62.45, 55.31, 48.32, 45.73, 32.45, 29.67, 24.98, 21.62. **HRMS** (ESI): m/z Calcd. For $C_{31}H_{35}NO_5SNa$ ([M+Na]$^+$): 556.2134, found [M+Na]$^+$: 556.2133.

***N*-((*E*)-7-hydroxy-3-phenylhept-2-en-1-yl)-4-methyl-*N*-((*E*)-4-oxo-4-(thiophen-2-yl) but-2-en-1-yl)benzenesulfonamide (5n)**

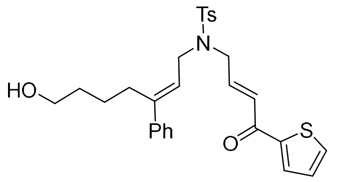

Light yellow oil. **^{1}H NMR** (400 MHz, Chloroform-*d*) δ 7.80 (d, *J* = 8.1 Hz, 2H), 7.73 (t, *J* = 4.5 Hz, 2H), 7.36 (d, *J* = 7.9 Hz, 2H), 7.30 (d, *J* = 7.0 Hz, 3H), 7.27 – 7.19 (m, 2H), 7.17 (t, *J* = 4.4 Hz, 1H), 7.06 – 6.89 (m, 2H), 5.48 (t, *J* = 7.0 Hz, 1H), 4.17 (d, *J* = 4.0 Hz, 2H), 4.12 (d, *J* = 7.0 Hz, 2H), 3.57 (t, *J* = 6.3 Hz, 2H), 2.49 (d, *J* = 15.6 Hz, 5H), 1.56 – 1.45 (m, 2H), 1.55 – 1.34(m, 2H). **^{13}C NMR** (101 MHz, Chloroform-*d*) δ 181.78, 145.38, 144.58, 143.82, 142.01, 141.63, 136.99, 134.67, 132.76, 130.01, 128.42, 128.40, 127.54, 127.32, 127.16, 126.37, 121.99, 62.36, 48.14, 45.71, 32.38, 29.54, 24.93, 21.57. **HRMS** (ESI): m/z Calcd. For $C_{28}H_{31}NO_4S_2Na$ ([M+Na]$^+$): 532.1587, found [M+Na]$^+$: 532.1583.

***N*-((*E*)-7-hydroxy-3-phenylhept-2-en-1-yl)-4-methyl-*N*-((*E*)-4-(5-methylthiophen-2-yl)-4-oxobut-2-en-1-yl) benzenesulfonamide (5o)**

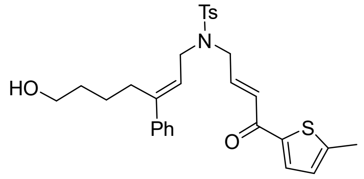

Light yellow oil. **^{1}H NMR** (400 MHz, Chloroform-*d*) δ 7.79 (d, *J* = 8.0 Hz, 2H), 7.55 (d, *J* = 3.8 Hz, 1H), 7.35 (d, *J* = 8.0 Hz, 2H), 7.33 – 7.25 (m, 3H), 7.22 (dd, *J* = 7.5, 2.1 Hz, 2H), 6.98 – 6.84 (m, 2H), 6.85 (d, *J* = 3.9 Hz, 1H), 5.47 (t, *J* = 7.0 Hz, 1H), 4.15 (d, *J* = 3.8 Hz, 2H), 4.10 (d, *J* = 7.0 Hz, 2H), 3.57 (t, *J* = 6.3 Hz, 2H), 2.59 (s, 3H), 2.46 (s, 5H), 1.51 (dd, *J* = 8.6, 6.2 Hz, 2H), 1.39 (td, *J* = 8.5, 4.2 Hz, 2H). **^{13}C NMR** (101 MHz, Chloroform-*d*) δ 181.33, 151.03, 145.39, 143.78, 142.49, 141.67, 141.14, 137.11, 133.43, 130.00, 128.41, 127.55, 127.34, 127.14, 127.09, 126.39, 122.03, 62.41, 48.05, 45.56, 32.42, 29.56, 24.97, 21.61, 16.25. **HRMS** (ESI): m/z Calcd. For $C_{29}H_{33}NO_4S_2Na$ ([M+Na]$^+$): 546.1743, found [M+Na]$^+$: 546.1743.

***N*-((*E*)-4-(5-bromothiophen-2-yl)-4-oxobut-2-en-1-yl)-*N*-((*E*)-7-hydroxy-3-phenylhept-2-en-1-yl)-4-methylbenzenesulfonamide (5p)**

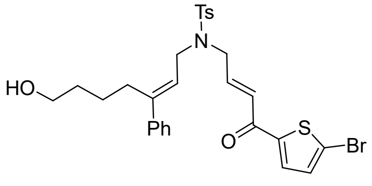

Light yellow oil. **^{1}H NMR** (400 MHz, Chloroform-*d*) *δ* 7.83 (d, *J* = 8.0 Hz, 2H), 7.49 (d, *J* = 4.1 Hz, 1H), 7.47 – 7.35 (m, 2H), 7.38 – 7.28 (m, 3H), 7.24 (dd, *J* = 4.8, 2.4 Hz, 2H), 7.17 (d, *J* = 4.1 Hz, 1H), 7.01 – 6.89 (m, 2H), 5.51 (d, *J* = 6.9 Hz, 1H), 4.29 – 4.05 (m, 4H), 3.61 (d, *J* = 6.5 Hz, 2H), 2.51 (s, 5H), 1.60 – 1.48 (m, 2H), 1.49 – 1.36 (m, 2H). **^{13}C NMR** (101 MHz, Chloroform-*d*) *δ* 180.68, 146.16, 145.52, 143.91, 142.72, 141.63, 137.00, 132.84, 131.57, 130.06, 128.48, 127.67, 127.39, 126.40, 126.15, 123.70, 121.99, 62.48, 48.22, 45.86, 32.44, 29.60, 25.00, 21.66. **HRMS** (ESI+): m/z Calcd. For $C_{28}H_{30}BrNO_4S_2Na$ ([M+Na]$^+$): 610.0695, found [M+Na]$^+$: 610.0692.

***N*-((*E*)-4-(5-chlorothiophen-2-yl)-4-oxobut-2-en-1-yl)-*N*-((*E*)-7-hydroxy-3-phenylhept-2-en-1-yl)-4-methylbenzenesulfonamide (5q)**

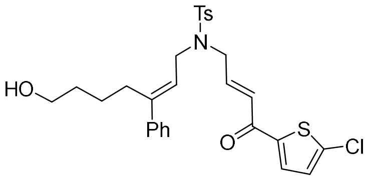

Light yellow oil. **^{1}H NMR** (400 MHz, Chloroform-*d*) *δ* 7.76 (d, *J* = 8.0 Hz, 2H), 7.47 (d, *J* = 4.1 Hz, 1H), 7.33 (d, *J* = 7.9 Hz, 2H), 7.28 (d, *J* = 15.2 Hz, 3H), 7.21 – 7.14 (m, 2H), 6.95 (d, *J* = 4.1 Hz, 1H), 6.94 – 6.78 (m, 2H), 5.43 (t, *J* = 7.0 Hz, 1H), 4.11 (d, *J* = 4.1 Hz, 2H), 4.06 (d, *J* = 7.1 Hz, 2H), 3.56 (d, *J* = 6.3 Hz, 2H), 2.43 (s, 5H), 1.49 (dd, *J* = 14.3, 7.0 Hz, 2H), 1.35 (qd, *J* = 9.5, 8.9, 5.9 Hz, 2H). **^{13}C NMR** (101 MHz, Chloroform-*d*) *δ* 180.86, 145.51, 143.92, 143.33, 142.62, 141.62, 140.56, 136.98, 132.29, 130.06, 128.48, 127.92, 127.66, 127.39, 126.40, 125.98, 121.99, 62.46, 48.22, 45.86, 32.43, 29.58, 24.98, 21.65. **HRMS** (ESI): m/z Calcd. For $C_{28}H_{30}ClNO_4S_2Na$ ([M+Na]$^+$): 566.1197, found [M+Na]$^+$: 566.1195.

***N*-((*E*)-7-hydroxy-3-phenylhept-2-en-1-yl)-4-methyl-N-((*E*)-4-oxopent-2-en-1-yl)-benzenesulfonamide (5r)**

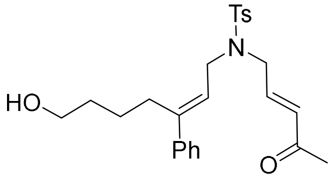

Colorless or light yellow oil, 72% yield. **^{1}H NMR** (400 MHz, Acetone-*d*$_6$) δ 7.83 – 7.76 (m, 2H), 7.43 (d, *J* = 7.9 Hz, 2H), 7.32 – 7.23 (m, 5H), 6.71 (dt, *J* = 16.0, 5.7 Hz,

1H), 6.20 (dt, J = 16.1, 1.8 Hz, 1H), 5.45 (t, J = 6.9 Hz, 1H), 4.08 (dd, J = 6.5, 3.5 Hz, 4H), 3.47 (q, J = 6.1 Hz, 2H), 2.52 (t, J = 7.7 Hz, 2H), 2.43 (s, 3H), 2.16 (s, 3H), 1.50 – 1.44 (m, 2H), 1.40 – 1.34 (m, 2H). **^{13}C NMR** (101 MHz, Acetone-d_6) δ 197.64, 145.47, 144.47, 142.92, 142.81, 138.37, 133.14, 130.73, 129.12, 128.22, 128.10, 127.16, 123.32, 62.16, 49.29, 46.80, 33.40, 30.09, 27.06, 25.93, 21.41. **HRMS** (ESI): m/z Calcd. For $C_{25}H_{31}NO_4SNa$ ([M+Na]$^+$): 464.1866, found: 464.1869.

***N*-((*E*)-7-hydroxy-3-phenylhept-2-en-1-yl)-4-methyl-N-((*E*)-4-oxobut-2-en-1-yl)-benzenesulfonamide (5s)**

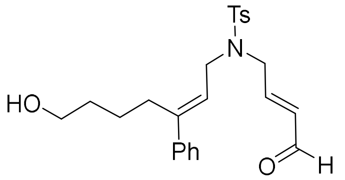

Colorless or light yellow oil, 80% yield. **^{1}H NMR** (400 MHz, Acetone-d_6) δ 9.57 (d, J = 7.8 Hz, 1H), 7.81 (d, J = 8.0 Hz, 2H), 7.43 (d, J = 8.0 Hz, 2H), 7.30 – 7.23 (m, 5H), 6.96 – 6.89 (m, 1H), 6.26 (m, 1H), 5.43 (d, J = 7.0 Hz, 1H), 4.19 (dd, J = 5.3, 1.7 Hz, 2H), 4.11 (d, J = 7.0 Hz, 2H), 3.47 (dd, J = 6.5, 3.4 Hz, 2H), 2.51 (d, J = 7.5 Hz, 2H), 2.43 (s, 3H), 1.49 – 1.44 (m, 2H), 1.39 – 1.34 (m, 2H). **^{13}C NMR** (101 MHz, Acetone-d_6) δ 193.63, 153.38, 145.68, 144.55, 142.89, 138.18, 134.27, 130.75, 129.12, 128.23, 128.11, 127.15, 123.11, 62.14, 49.32, 47.01, 33.37, 30.09, 25.92, 21.42. **HRMS** (ESI): m/z Calcd. For $C_{24}H_{29}NO_4SNa$ ([M+Na]$^+$): 450.1710, found: 450.1710.

***N*-((*E*))-7-hydroxy-3-phenylhept-2-en-1-yl)-4-methyl-*N*-((*E*))-5-oxo-5-phenylpent-3-en-1-yl)benzenesulfonamide (5t)**

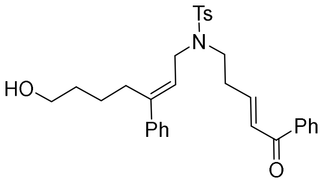

Light yellow oil. **^{1}H NMR** (400 MHz, Chloroform-*d*) *δ* 7.96 – 7.89 (m, 2H), 7.75 (d, J = 8.0 Hz, 2H), 7.59 (t, J = 7.3 Hz, 1H), 7.48 (t, J = 7.6 Hz, 2H), 7.35 – 7.26 (m, 5H), 7.22 (d, J = 2.0 Hz, 2H), 6.99 – 6.92 (m, 2H), 5.44 (t, J = 6.8 Hz, 1H), 4.07 (d, J = 6.8 Hz, 2H), 3.60 (t, J = 6.3 Hz, 2H), 3.39 (t, J = 7.2 Hz, 2H), 2.66 (h, J = 6.0 Hz, 2H), 2.53 (t, J = 7.7 Hz, 2H), 2.43 (s, 3H), 1.59 – 1.50 (m, 2H), 1.41 (tt, J = 9.9, 6.0 Hz, 2H). **^{13}C NMR** (101 MHz, Chloroform-*d*) *δ* 190.56, 145.05, 144.67, 143.59, 141.70, 137.57, 137.03, 133.02, 129.91, 128.74, 128.70, 128.46, 127.94, 127.55, 127.32, 126.36, 122.62, 62.48, 46.43, 46.39, 32.86, 32.46, 29.64, 24.98, 21.60. **HRMS** (ESI): m/z Calcd. For $C_{31}H_{35}NO_4SNa$ ([M+Na]$^+$): 540.2179, found: 540.2177.

2.2 General procedure for IEDHDA-keto acetalization reactions with **5a-t**

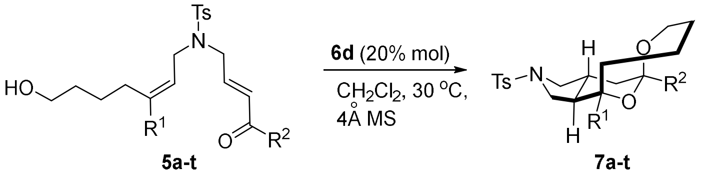

Substrate **5a-t** (30 mg, 1.0 eq.) was dissolved in DCM (1.0 ml), then 4Å molecular sieves (60 mg) followed by catalyst **6d** (0.2 eq.) were added. The reaction mixture was stirred at 30 ºC and monitored by TLC (generally for 48h). When the reaction was completed, the mixture was purified by silica gel column to afford products **7a-r**.

(3a*R*, 11a*R*)-5, 11-diphenyl-2-tosyldodecahydro-5, 11-epoxyoxecino [4, 5-*c*] pyrrole (7a)

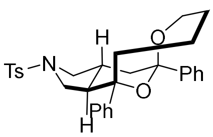

Light yellow oil, $[\alpha]_D^{25}$ = + 57.57 (*c* 0.25, DCM), 26.1 mg, 87% yield, 95% ee, dr>20:1, determined by HPLC (Chiralcel column AD-H, hexane/i-PrOH = 80/20, flow rate 1.0 mL/min, UV detection at 254 nm), t_{major} = 39.8min ,t_{minor} = 25.7min. **^{1}H NMR** (400 MHz, Chloroform-*d*) δ 7.66 (d, J = 8.2 Hz, 2H), 7.54 (dt, J = 17.8, 8.9 Hz, 4H), 7.48 – 7.34 (m, 4H), 7.35 – 7.25 (m, 4H), 3.63 (td, J = 9.4, 5.1 Hz, 2H), 3.48 (dd, J = 9.2, 7.3 Hz, 1H), 3.41 (td, J = 12.3, 2.2 Hz, 1H), 3.23 (dd, J = 11.6, 9.2 Hz, 1H), 2.69 (dd, J = 10.9, 9.2 Hz, 1H), 2.61 – 2.43 (m, 1H), 2.42 (s, 3H), 2.26 (dd, J = 9.2, 3.6 Hz, 3H), 1.76 (td, J = 11.7, 7.3 Hz, 1H), 1.63 – 1.50 (m, 2H), 1.49 – 1.39 (m, 1H), 1.32 (t, J = 12.7 Hz, 1H), 1.28 – 1.16 (m, 1H). **^{13}C NMR** (101 MHz, Chloroform-*d*) δ 146.09, 144.87, 143.53, 134.73, 129.89, 128.47 (2C), 127.88, 127.31, 127.16, 125.88, 125.51, 101.25, 79.89, 67.00, 52.73, 52.51, 49.37, 42.00, 34.46, 31.98, 31.51, 21.66, 20.99. **HRMS** (ESI): m/z calcd for $C_{30}H_{33}NO_4SNa$ ($[M+Na]^+$): 526.2023, found: 526.2020.

(3a*R*,11a*R*)-11-(4-chlorophenyl)-5-phenyl-2-tosyldodecahydro-5,11-epoxyoxecino [4, 5-*c*] pyrrole (7b)

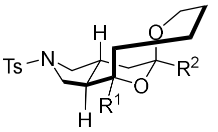

R1=4-Cl-Ph, R2=Ph

Light yellow oil, $[\alpha]_D^{25}$ = +29.26(*c* 1.37, DCM), 25.8 mg, 86% yield, 94% ee, dr=20>1, determined by HPLC (Chiralcel column AD-H, hexane/i-PrOH = 80/20, flow rate 1.0 mL/min, UV detection at 254 nm), t_{major} = 11.7min, t_{minor} = 20.1min. **^{1}H NMR** (400 MHz, Chloroform-*d*) δ 7.66 (d,

J = 8.0 Hz, 2H), 7.53 (d, J = 7.6 Hz, 2H), 7.46 (d, J = 8.8 Hz, 2H), 7.42 – 7.30 (m, 4H), 7.33 – 7.23 (m, 3H), 3.69 – 3.58 (m, 2H), 3.49 – 3.33 (m, 2H), 3.22 (dd, J = 11.5, 9.2 Hz, 1H), 2.68 (dd, J = 10.8, 9.2 Hz, 1H), 2.54 (dddd, J = 19.3, 15.3, 9.1, 5.5 Hz, 1H), 2.43 (s, 3H), 2.34 – 2.12 (m, 3H), 1.70 (td, J = 11.8, 7.4 Hz, 1H), 1.65 – 1.50 (m, 2H), 1.45 (ddt, J = 13.3, 5.5, 2.6 Hz, 1H), 1.31 (t, J = 12.7 Hz, 1H), 1.17 (d, J = 8.3 Hz, 1H). **^{13}C NMR** (151 MHz, Chloroform-*d*) δ 144.71, 144.63, 143.62, 134.65, 133.01, 129.94, 128.61, 128.52, 127.99, 127.31, 127.03, 125.80, 101.34, 79.66, 67.05, 52.65, 52.41, 49.20, 41.94, 34.46, 32.04, 31.41, 21.68, 20.89. **HRMS** (ESI): m/z calcd for $C_{30}H_{32}ClNO_4SNa$ ([M+Na]$^+$): 560.1633, found: 560.1625.

(3a*R*,11a*R*)-11-(3-chlorophenyl)-5-phenyl-2-tosyldodecahydro-5,11-epoxyoxecino [4, 5-*c*] pyrrole (7c)

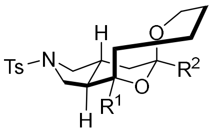

R^1=3-Cl-Ph, R^2=Ph

Light yellow oil, $[\alpha]_D^{25}$ = +36.40(*c* 0.25, DCM), 19.8mg, 66% yield, 91% ee, dr>20:1, determined by HPLC (Chiralcel column AD-H, hexane/i-PrOH = 80/20, flow rate 1.0 mL/min, UV detection at 254 nm), t_{major} = 6.6min, t_{minor} = 9.1min. **^{1}H NMR** (400 MHz, Chloroform-*d*) δ 7.66 (d, J = 8.0 Hz, 2H), 7.58 (s, 1H), 7.54 (d, J = 7.6 Hz, 2H), 7.39 (t, J = 7.6 Hz, 2H), 7.31 (dt, J = 11.4, 4.2 Hz, 6H), 3.69 – 3.58 (m, 2H), 3.50 – 3.34 (m, 2H), 3.21 (dd, J = 11.5, 9.2 Hz, 1H), 2.74 – 2.64 (m, 1H), 2.65 – 2.43 (m, 1H), 2.43 (s, 3H), 2.34 – 2.13 (m, 3H), 1.70 (td, J = 11.8, 7.3 Hz, 1H), 1.57 (m, 2H), 1.50 – 1.40 (m, 1H), 1.32 (t, J = 12.8 Hz, 1H), 1.18 (d, J = 23.3 Hz, 1H). **^{13}C NMR** (151 MHz, Chloroform-*d*) δ 148.28, 144.55, 143.65, 134.59, 129.93, 129.76, 128.54, 127.99, 127.39, 127.31 (2C), 126.11, 125.79, 123.63, 101.37, 79.68, 67.06, 52.66, 52.31, 49.17, 41.93, 34.45, 31.93, 31.38, 21.68, 20.90. **HRMS** (ESI): m/z calcd for $C_{30}H_{32}ClNO_4SNa$ ([M+Na]$^+$): 560.1633, found: 560.1635.

(3a*R*,11a*R*)-11-(4-bromophenyl)-5-phenyl-2-tosyldodecahydro-5,11-epoxyoxecino [4, 5-*c*] pyrrole (7d)

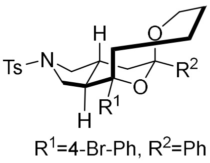

R^1=4-Br-Ph, R^2=Ph

Light yellow oil, $[\alpha]_D^{25}$ = +24.25 (*c* 1.00, DCM), 28.2 mg, 94% yield, 98% ee, dr>20:1, determined by HPLC (Chiralcel column AD-H, hexane/i-PrOH = 80/20, flow rate 1.0 mL/min, UV detection at 254

nm), *t* $_{major}$ = 14.2 min, *t* $_{minor}$ = 19.3 min. **^{1}H NMR** (600 MHz, Chloroform-*d*) *δ* 7.56 – 7.51 (m, 2H), 7.45 – 7.37 (m, 3H), 7.31 – 7.22 (m, 4H), 7.22 – 7.14 (m, 3H), 7.13 (s, 1H), 3.55 – 3.47 (m, 2H), 3.31 (dd, *J* = 9.2, 7.4 Hz, 1H), 3.27 (td, *J* = 12.3, 2.2 Hz, 1H), 3.10 (dd, *J* = 11.5, 9.2 Hz, 1H), 2.55 (dd, *J* = 10.9, 9.3 Hz, 1H), 2.46 – 2.37 (m, 1H), 2.30 (s, 3H), 2.19 – 2.09 (m, 2H), 2.04 (dd, *J* = 14.5, 10.3 Hz, 1H), 1.57 (td, *J* = 11.8, 7.3 Hz, 1H), 1.51 – 1.40 (m, 2H), 1.35 – 1.29 (m, 1H), 1.18 (dd, *J* = 13.4, 12.3 Hz, 1H), 1.10 – 1.00 (m, 1H). **^{13}C NMR** (151 MHz, Chloroform-*d*) *δ* 145.27, 144.62, 143.63, 134.64, 131.57, 129.94, 128.52, 127.99, 127.40, 127.31, 125.80, 121.16, 101.33, 79.70, 67.05, 52.63, 52.33, 49.18, 41.93, 34.45, 32.03, 31.41, 21.69, 20.88. **HRMS** (ESI): m/z calcd for $C_{30}H_{32}BrNO_4SNa$ ([M+Na]$^+$): 604.1128, found: 604.1128.

(3a*R*,11a*R*)-11-(3-methoxyphenyl)-5-phenyl-2-tosyldodecahydro-5,11-epoxy-oxecino [4, 5-*c*] pyrrole (7e)

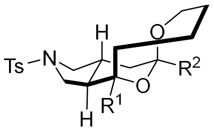

R^1=3-MeO-Ph, R^2=Ph

Light yellow oil, [α]$_D^{25}$ = +24.08 (*c* 0.78, DCM), 28.5 mg, 95% yield, 91% ee, dr>20:1, determined by HPLC (Chiralcel column AD-H, hexane/i-PrOH = 80/20, flow rate 1.0 mL/min, UV detection at 254 nm), *t* $_{major}$ = 7.3min, *t* $_{minor}$ = 10.7min. **^{1}H NMR** (400 MHz, Chloroform-*d*) *δ* 7.66 (d, *J* = 8.0 Hz, 2H), 7.57 (d, *J* = 7.6 Hz, 2H), 7.37 (t, *J* = 7.5 Hz, 2H), 7.36 – 7.23 (m, 4H), 7.18 (s, 1H), 7.03 (dd, *J* = 7.8, 1.8 Hz, 1H), 6.84 (dd, *J* = 8.1, 2.5 Hz, 1H), 3.86 (s, 3H), 3.63 (td, *J* = 11.0, 10.3, 5.3 Hz, 2H), 3.48 (dd, *J* = 9.2, 7.3 Hz, 1H), 3.40 (td, *J* = 12.2, 2.3 Hz, 1H), 3.20 (dd, *J* = 11.5, 9.3 Hz, 1H), 2.69 (dd, *J* = 10.8, 9.2 Hz, 1H), 2.59 – 2.46 (m, 1H), 2.42 (s, 3H), 2.29 – 2.20 (m, 3H), 1.76 (td, *J* = 11.7, 7.3 Hz, 1H), 1.56 (m, 2H), 1.50 – 1.39 (m, 1H), 1.32 (t, *J* = 12.8 Hz, 1H), 1.21 (d, *J* = 9.6 Hz, 1H). **^{13}C NMR** (151 MHz, Chloroform-*d*) *δ* 159.76, 147.91, 144.86, 143.54, 134.72, 129.91, 129.45, 128.47, 127.88, 127.32, 125.87, 117.82, 112.43, 111.69, 101.25, 79.83, 67.02, 55.37, 52.75, 52.41, 49.37, 41.98, 34.45, 31.96, 31.51, 21.67, 21.02. **HRMS** (ESI): m/z calcd for $C_{31}H_{35}NO_5SH$ ([M+H]$^+$): 534.2309, found: 534.2307.

(3a*R*,11a*R*)-11-(2-methoxyphenyl)-5-phenyl-2-tosyldodecahydro-5,11-epoxyoxe-cino [4, 5-*c*] pyrrole (7f)

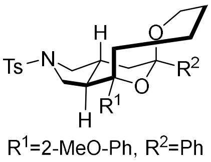

R^1=2-MeO-Ph, R^2=Ph

Light yellow oil, $[\alpha]_D^{25}$ = -5.26 (*c* 0.53, DCM), 15 mg, 50% yield, 96% ee, dr>20:1, determined by HPLC (Chiralcel column AD-H, hexane/i-PrOH = 60/40, flow rate 1.0 mL/min, UV detection at 254 nm), t_{major} = 21.5min, t_{minor} = 12.1min. **^{1}H NMR** (400 MHz, Chloroform-*d*) δ 7.90 (d, *J* = 7.9 Hz, 1H), 7.65 (d, *J* = 7.9 Hz, 2H), 7.56 (d, *J* = 7.5 Hz, 2H), 7.36 (t, *J* = 6.4 Hz, 3H), 7.31 – 7.21 (m, 3H), 7.00 (t, *J* = 7.6 Hz, 1H), 6.90 (d, *J* = 8.2 Hz, 1H), 3.81 (s, 3H), 3.69 – 3.54 (m, 2H), 3.56 – 3.34 (m, 2H), 3.22 (t, *J* = 10.8 Hz, 1H), 2.78 – 2.62 (m, 2H), 2.58 – 2.44 (m, 1H), 2.40 (s, 3H), 2.22 – 2.04 (m, 2H), 1.95 (td, *J* = 11.6, 7.1 Hz, 1H), 1.53 (dd, *J* = 35.6, 11.6 Hz, 2H), 1.44 – 1.23 (m, 2H), 1.21 – 1.06 (m, 1H). **^{13}C NMR** (151 MHz, Chloroform-*d*) δ 156.05, 145.27, 143.19, 134.78, 132.89, 129.67, 128.55, 128.47, 128.27, 127.59, 127.17, 125.85, 120.48, 111.34, 101.14, 80.42, 67.17, 54.57, 52.24, 50.66, 50.09, 42.06, 34.12, 31.55, 29.21, 21.50, 21.27. **HRMS** (ESI): m/z calcd for $C_{31}H_{35}NO_5SNa$ ($[M+Na]^+$): 556.2128, found: 556.2122.

(3a*R*,11a*R*)-5-phenyl-11-(*m*-tolyl)-2-tosyldodecahydro-5,11-epoxyoxecino [4, 5-*c*] pyrrole (7g)

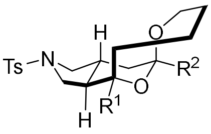

R^1=3-Me-Ph, R^2=Ph

Light yellow oil, $[\alpha]_D^{25}$ = -6.37(*c* 1.22, DCM), 24.3 mg, 81% yield, 90% ee, dr>20:1, determined by HPLC (Chiralcel column AD-H, hexane/i-PrOH = 80/20, flow rate 1.0 mL/min, UV detection at 254 nm), t_{major} = 6.2min, t_{minor} = 8.9min. **^{1}H NMR** (400 MHz, Chloroform-*d*) δ 7.65 (d, *J* = 7.9 Hz, 2H), 7.55 (d, *J* = 7.5 Hz, 2H), 7.36 (t, *J* = 7.5 Hz, 2H), 7.33 – 7.21 (m, 6H), 7.09 (d, *J* = 7.1 Hz, 1H), 3.61 (q, *J* = 7.6, 6.5 Hz, 2H), 3.41 (dt, *J* = 23.8, 10.2 Hz, 2H), 3.19 (t, *J* = 10.4 Hz, 1H), 2.67 (t, *J* = 10.0 Hz, 1H), 2.59 – 2.47 (m, 1H), 2.40 (d, *J* = 3.1 Hz, 6H), 2.23 (dd, *J* = 10.1, 4.6 Hz, 3H), 1.75 (td, *J* = 11.8, 7.3 Hz, 1H), 1.59 (d, *J* = 8.9 Hz, 2H), 1.42 (d, *J* = 13.4 Hz, 1H), 1.36 – 1.16 (m, 2H). **^{13}C NMR** (101 MHz, Chloroform-*d*) δ 146.03, 144.91, 143.51, 137.94, 134.71, 129.88, 128.44, 128.36, 127.92, 127.85, 127.31, 126.20, 125.88, 122.66, 101.20, 79.87, 66.97, 52.74, 52.52, 49.38, 41.97, 34.43, 31.82, 31.53, 22.06, 21.66, 21.06. **HRMS** (ESI): m/z calcd for $C_{31}H_{35}NO_4SNa$ ($[M+Na]^+$): 540.2179, found: 540.2175.

(3a*R*, 11a*R*)-11-methyl-5-phenyl-2-tosyldodecahydro-5, 11-epoxyoxecino [4, 5-*c*] pyrrole (7h)

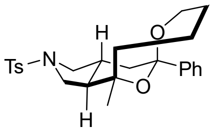

Light yellow oil, $[\alpha]_D^{25}$ = +23.30(*c* 1.02, DCM), 14.1mg, 47% yield, 93% ee, dr>20:1, determined by HPLC (Chiralcel column AD-H, hexane/i-PrOH = 70/30, flow rate 1.0 mL/min, UV detection at 254 nm), t_{major} = 23.0min, t_{minor} = 13.0min. **^{1}H NMR** (600 MHz, Chloroform-*d*) δ 7.73 – 7.67 (m, 2H), 7.40 (d, *J* = 7.6 Hz, 2H), 7.36 – 7.31 (m, 2H), 7.30 (td, *J* = 7.4, 1.0 Hz, 2H), 7.25 (td, *J* = 6.9, 1.4 Hz, 1H), 3.60 (dd, *J* = 9.4, 7.2 Hz, 1H), 3.57 – 3.50 (m, 2H), 3.26 (ddd, *J* = 14.0, 8.3, 3.8 Hz, 1H), 2.94 (dd, *J* = 11.7, 9.3 Hz, 1H), 2.73 (dd, *J* = 10.9, 9.4 Hz, 1H), 2.45 (s, 3H), 2.37 – 2.26 (m, 1H), 2.12 (dd, *J* = 13.2, 3.7 Hz, 1H), 1.82 (dd, *J* = 13.8, 7.1 Hz, 1H), 1.70 (ddd, *J* = 11.6, 8.7, 3.8 Hz, 1H), 1.65 – 1.58 (m, 1H), 1.57 (d, *J* = 6.6 Hz, 1H), 1.54 – 1.45 (m, 2H), 1.44 (dd, *J* = 13.8, 3.4 Hz, 1H), 1.28 (s, 3H), 1.15 (dd, *J* = 13.2, 12.2 Hz, 1H). **^{13}C NMR** (151 MHz, Chloroform-*d*) δ 144.54, 143.54, 134.67, 129.91, 128.25, 127.72, 127.39, 125.84, 100.36, 75.03, 66.51, 52.94, 51.68, 49.02, 41.99, 33.58, 33.37, 32.01, 29.03, 21.70, 21.23. **HRMS** (ESI): m/z calcd for $C_{25}H_{31}NO_4SNa$ ($[M+Na]^+$): 464.1866, found: 464.1863.

(3a*R*, 11a*R*)-11-phenyl-5-(*p*-tolyl)-2-tosyldodecahydro-5, 11-epoxyoxecino [4, 5-*c*] pyrrole (7i)

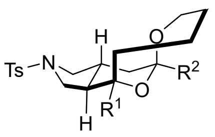

R1=Ph, R2=4-Me-Ph

Light yellow oil, $[\alpha]_D^{25}$ = -7.85(*c* 1.01, DCM), 28.5 mg, 95% yield, 96% ee, dr=20>1, determined by HPLC (Chiralcel column AD-H, hexane/i-PrOH = 80/20, flow rate 1.0 mL/min, UV detection at 254 nm), t_{major} = 7.8min, t_{minor} = 16.2min. **^{1}H NMR** (400 MHz, Chloroform-*d*) δ 7.66 (d, *J* = 8.1 Hz, 2H), 7.55 – 7.51 (m, 2H), 7.51 – 7.42 (m, 1H), 7.40 (t, *J* = 7.6 Hz, 2H), 7.36 – 7.21 (m, 4H), 7.19 (d, *J* = 7.9 Hz, 2H), 3.68 – 3.59 (m, 2H), 3.47 (dd, *J* = 9.2, 7.4 Hz, 1H), 3.41 (td, *J* = 12.1, 2.2 Hz, 1H), 3.22 (dd, *J* = 11.5, 9.2 Hz, 1H), 2.69 (dd, *J* = 10.9, 9.2 Hz, 1H), 2.66 – 2.43 (m, 1H), 2.42 (s, 3H), 2.36 (s, 3H), 2.28 – 2.20 (m, 3H), 1.75 (td, *J* = 11.8, 7.3 Hz, 1H), 1.59 – 1.41 (m, 3H), 1.27 (dt, *J* = 25.1, 13.0 Hz, 2H). **^{13}C NMR** (151 MHz, Chloroform-*d*) δ 146.16, 143.53, 141.97, 137.59, 134.70, 129.90, 129.13, 128.45, 127.31, 127.13, 125.82, 125.52, 101.25, 79.79, 66.90, 52.74, 52.49,

49.38, 42.05, 34.47, 32.00, 31.52, 21.67, 21.24, 20.99. **HRMS** (ESI): m/z calcd for $C_{31}H_{35}NO_4SNa$ ([M+Na]$^+$): 540.2179, found: 540.2179.

(3a*R*, 11a*R*)-11-phenyl-5-(*o*-tolyl)-2-tosyldodecahydro-5, 11-epoxyoxecino [4, 5-*c*] pyrrole (7j)

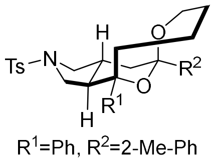

Light yellow oil, $[\alpha]_D^{25}$ =+ 8.01(*c* 0.62, DCM), 20.7 mg, 69% yield, 96% ee, dr > 20:1, determined by HPLC (Chiralcel column AD-H, hexane/i-PrOH = 80/20, flow rate 1.0 mL/min, UV detection at 254 nm), t_{major} = 32.5min, t_{minor} = 27.2min. **^{1}H NMR** (400 MHz, Chloroform-*d*) δ 7.84 (dd, *J* = 7.2, 2.1 Hz, 1H), 7.67 – 7.52 (m, 2H), 7.47 (d, *J* = 7.3 Hz, 2H), 7.35 (t, *J* = 7.7 Hz, 2H), 7.26 (dd, *J* = 14.0, 7.0 Hz, 1H), 7.23 – 7.15 (m, 3H), 7.15 – 7.11 (m, 1H), 7.12 – 7.04 (m, 1H), 3.58 (dd, *J* = 9.0, 6.8 Hz, 1H), 3.51 (d, *J* = 11.8 Hz, 1H), 3.44 – 3.29 (m, 2H), 3.14 (dd, *J* = 11.5, 9.2 Hz, 1H), 2.63 (t, *J* = 9.9 Hz, 1H), 2.55 – 2.45 (m, 1H), 2.43 (s, 3H), 2.35 (s, 3H), 2.30 – 2.11 (m, 2H), 1.71 (td, *J* = 11.7, 7.2 Hz, 1H), 1.67 – 1.30 (m, 4H), 1.24 (d, *J* = 13.0 Hz, 2H). **^{13}C NMR** (151 MHz, Chloroform-*d*) δ 146.27, 143.52, 142.14, 134.92, 134.68, 132.48, 129.90, 128.49, 127.92, 127.62, 127.31, 127.18, 125.93, 125.67, 101.34, 80.25, 65.65, 52.71, 52.65, 49.42, 38.86, 34.15, 31.83, 31.13, 21.67, 21.16, 20.48. **HRMS** (ESI): m/z calcd for $C_{31}H_{35}NO_4SNa$ ([M+Na]$^+$): 540.2179, found: 540.2177.

(3a*R*,11a*R*)-5-(4-ethylphenyl)-11-phenyl-2-tosyldodecahydro-5,11-methano-oxecino [4, 5-*c*] pyrrole (7k)

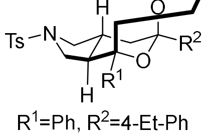

Light yellow oil, $[\alpha]_D^{25}$ = +38.45(*c* 1.42, DCM), 27.9 mg, 94% yield, 97% ee, dr=20>1, determined by HPLC (Chiralcel column AD-H, hexane/i-PrOH = 80/20, flow rate 1.0 mL/min, UV detection at 254 nm), t_{major} =7.5min, t_{minor} = 15.4min. **^{1}H NMR** (400 MHz, Chloroform-*d*) δ 7.66 (d, *J* = 8.0 Hz, 2H), 7.56 – 7.50 (m, 2H), 7.48 (d, *J* = 7.7 Hz, 2H), 7.40 (t, *J* = 7.6 Hz, 2H), 7.29 (t, *J* = 8.3 Hz, 3H), 7.21 (d, *J* = 8.0 Hz, 2H), 3.68 – 3.57 (m, 2H), 3.53 – 3.37 (m, 2H), 3.23 (dd, *J* = 11.5, 9.2 Hz, 1H), 2.74 – 2.61 (m, 3H), 2.62 – 2.43 (m, 1H), 2.42 (s, 3H), 2.32 – 2.17 (m, 3H), 1.75 (td, *J* = 11.8, 7.3 Hz, 1H), 1.59 (m, 2H), 1.49 – 1.38 (m, 1H), 1.32 (t, *J* = 12.7 Hz, 1H), 1.24 (t, *J* = 7.6 Hz, 4H). **^{13}C NMR** (101 MHz,

Chloroform-*d*) *δ* 146.17, 143.90, 143.52, 142.17, 134.68, 129.89, 128.44, 127.91, 127.30, 127.12, 125.86, 125.51, 101.27, 79.77, 66.93, 52.73, 52.48, 49.39, 42.06, 34.48, 32.02, 31.53, 28.62, 21.66, 20.97, 15.64. **HRMS** (ESI): m/z calcd for $C_{32}H_{37}NO_4SNa$ ($[M+Na]^+$): 554.2336, found: 554.2337.

((3a*R*,11a*R*)-5-(4-methoxyphenyl)-11-phenyl-2-tosyldodecahydro-5,11-epoxyo-xecino [4, 5-*c*] pyrrole (7l)

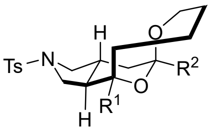

R1=Ph, R2=4-MeO-Ph

Light yellow oil, $[\alpha]_D^{25}$ = +41.25(*c* 1.31, DCM), 22.5 mg, 75% yield, 98% ee, dr>20:1, determined by HPLC (Chiralcel column AD-H, hexane/i-PrOH = 80/20, flow rate 1.0 mL/min, UV detection at 254 nm), t_{major} = 9.7min, t_{minor} = 20.0min. **^{1}H NMR** (400 MHz, Chloroform-*d*) *δ* 7.66 (d, *J* = 8.0 Hz, 2H), 7.55 – 7.45 (m, 4H), 7.40 (t, *J* = 7.6 Hz, 2H), 7.28 (d, *J* = 8.2 Hz, 3H), 6.94 – 6.87 (m, 2H), 3.82 (s, 3H), 3.68 – 3.56 (m, 2H), 3.51 – 3.36 (m, 2H), 3.22 (dd, *J* = 11.5, 9.2 Hz, 1H), 2.69 (dd, *J* = 10.8, 9.2 Hz, 1H), 2.65 – 2.43 (m, 1H), 2.42 (s, 3H), 2.28 – 2.20 (m, 3H), 1.74 (td, *J* = 11.8, 7.3 Hz, 1H), 1.59 (m, 2H), 1.50 – 1.40 (m, 1H), 1.31 (t, *J* = 12.7 Hz, 1H), 1.27 – 1.12 (m, 1H). **^{13}C NMR** (151 MHz, Chloroform-*d*) *δ* 159.21, 146.13, 143.51, 137.13, 134.70, 129.89, 128.45, 127.30, 127.14, 127.09, 125.50, 113.72, 101.12, 79.80, 66.75, 55.42, 52.71, 52.51, 49.36, 42.12, 34.49, 31.94, 31.52, 21.66, 20.98. **HRMS** (ESI): m/z calcd for $C_{31}H_{35}NO_5SNa$ ($[M+Na]^+$): 556.2134, found: 556.2133.

(3a*R*, 11a*R*)-5-(3-methoxyphenyl)-11-phenyl-2-tosyldodecahydro-5, 11 epoxy-oxecino [4, 5-*c*] pyrrole (7m)

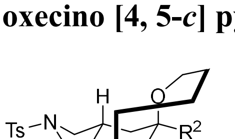

R1=Ph, R2=3-MeO-Ph

Light yellow oil, $[\alpha]_D^{25}$ = +21.62(*c* 0.60, DCM), 27.6 mg, 92% yield, 89% ee, dr>20:1, determined by HPLC (Chiralcel column AD-H, hexane/i-PrOH = 80/20, flow rate 1.0 mL/min, UV detection at 254 nm), t_{major} = 8.9min, t_{minor} = 15.3min. **^{1}H NMR** (400 MHz, Chloroform-*d*) *δ* 7.66 (d, *J* = 8.0 Hz, 2H), 7.52 (d, *J* = 7.4 Hz, 2H), 7.40 (t, *J* = 7.6 Hz, 2H), 7.29 (dd, *J* = 8.1, 3.2 Hz, 4H), 7.12 (d, *J* = 40.5 Hz, 2H), 6.85 (dd, *J* = 8.2, 2.6 Hz, 1H), 3.83 (s, 3H), 3.64 (dd, *J* = 9.1, 6.8 Hz, 2H), 3.52 – 3.36 (m, 2H), 3.23 (dd, *J* = 11.5, 9.2 Hz, 1H), 2.69 (dd, *J* = 10.8, 9.2 Hz, 1H), 2.65 – 2.44 (m, 1H), 2.42 (s, 3H), 2.24 (dt, *J* = 11.4, 3.7 Hz, 3H),

1.75 (td, J = 11.7, 7.3 Hz, 1H), 1.59 (m, 2H), 1.50 – 1.39 (m, 1H), 1.40 – 1.30 (m, 1H), 1.26 – 1.12 (m, 1H). **^{13}C NMR (**151 MHz, Chloroform-*d*) δ 159.77, 146.62, 146.08, 143.55, 134.69, 129.92, 129.55, 128.48, 127.32, 127.17, 125.51, 118.04, 113.04, 111.91, 101.15, 79.93, 67.08, 55.36, 52.73, 52.46, 49.38, 41.87, 34.45, 32.01, 31.51, 21.69, 20.98. **HRMS** (ESI): m/z calcd for $C_{31}H_{35}NO_5SNa$ ([M+Na]$^+$): 556.2134, found: 556.2130.

((3a*R*, 11a*R*)-11-phenyl-5-(thiophen-2-yl)-2-tosyldodecahydro-5, 11-epoxyoxecino [4, 5-*c*] pyrrole (7n)

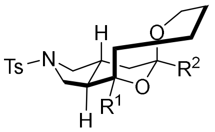

R1=Ph, R2=2-Thienyl

Light yellow oil, $[\alpha]_D^{25}$ = -3.04(*c* 0.48, DCM), 14.1 mg, 47% yield, 92% ee, dr>20:1, determined by HPLC (Chiralcel column AD-H, hexane/i-PrOH = 80/20, flow rate 1.0 mL/min, UV detection at 254 nm), t_{major} = 8.0min, t_{minor} = 14.7min. **^{1}H NMR** (400 MHz, Chloroform-*d*) δ 7.66 (d, J = 8.0 Hz, 2H), 7.52 (d, J = 7.3 Hz, 2H), 7.40 (t, J = 7.6 Hz, 2H), 7.34 – 7.23 (m, 4H), 7.06 (d, J = 3.6 Hz, 1H), 7.00 (dd, J = 5.0, 3.5 Hz, 1H), 3.71 – 3.62 (m, 3H), 3.45 (dd, J = 9.3, 7.4 Hz, 1H), 3.23 (dd, J = 11.5, 9.2 Hz, 1H), 2.71 (dd, J = 10.8, 9.2 Hz, 1H), 2.52 (dd, J = 7.8, 3.2 Hz, 1H), 2.43 (s, 3H), 2.37 (dd, J = 13.2, 3.7 Hz, 1H), 2.32 – 2.15 (m, 2H), 1.73 (td, J = 11.8, 7.4 Hz, 1H), 1.67 – 1.48 (m, 2H), 1.35 – 1.14 (m, 3H). **^{13}C NMR** (151 MHz, Chloroform-*d*) δ 148.83, 145.62, 143.57, 134.67, 129.91, 128.46, 127.30, 127.20, 126.89, 125.41, 124.96, 123.77, 100.16, 80.43, 67.00, 52.64, 52.44, 49.20, 42.32, 34.42, 31.95, 31.49, 21.66, 20.89. **HRMS** (ESI): m/z calcd for $C_{28}H_{31}NO_4S_2Na$ ([M+Na]$^+$): 532.1587, found: 532.1585.

((3a*R*,11a*R*)-5-(5-methylthiophen-2-yl)-11-phenyl-2-tosyldodecahydro-5,11-epoxyoxecino [4, 5-*c*] pyrrole (7o)

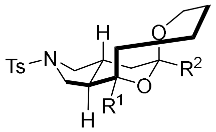

R1=Ph, R2=5-Me-2-Thienyl

Light yellow oil, $[\alpha]_D^{25}$ = -18.00 (*c* 1.58, DCM), 15 mg, 50% yield, 97% ee, dr>20:1, determined by HPLC (Chiralcel column AD-H, hexane/i-PrOH = 80/20, flow rate 1.0 mL/min, UV detection at 254 nm), t_{major} = 8.0min, t_{minor} = 18.3min. **^{1}H NMR** (400 MHz, Chloroform-*d*) δ 7.65 (d, J = 8.0 Hz, 2H), 7.49 (d, J = 7.3 Hz, 2H), 7.39 (t, J = 7.6 Hz, 2H), 7.27 (d, J = 8.2 Hz, 3H), 6.82 (d, J = 3.5 Hz, 1H), 6.65 – 6.59 (m, 1H), 3.75 – 3.60 (m, 3H), 3.43 (dd,

J = 9.2, 7.4 Hz, 1H), 3.20 (dd, *J* = 11.5, 9.3 Hz, 1H), 2.69 (dd, *J* = 10.8, 9.2 Hz, 1H), 2.47 (s, 3H), 2.42 (s, 3H), 2.32 (dd, *J* = 13.2, 3.8 Hz, 1H), 2.29 – 2.12 (m, 2H), 1.69 (td, *J* = 11.8, 7.4 Hz, 1H), 1.59 – 1.43 (m, 4H), 1.30 – 1.13 (m, 2H). **^{13}C NMR** (101 MHz, Chloroform-*d*) δ 146.10, 145.67, 143.55, 139.44, 134.66, 129.89, 128.43, 127.28, 127.14, 125.40, 124.90, 123.57, 100.04, 66.88, 52.66, 52.44, 49.21, 42.29, 34.41, 31.91, 31.50, 21.66, 20.89, 15.42. **HRMS** (ESI): m/z calcd for $C_{29}H_{33}NO_4S_2Na$ ($[M+Na]^+$): 546.1743, found: 546.1742.

((3a*R*,11a*R*)-5-(5-bromothiophen-2-yl)-11-phenyl-2-tosyldodecahydro-5,11-epoxyoxecino [4, 5-*c*] pyrrole (7p)

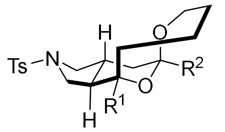

R1=Ph, R2=5-Br-2-Thienyl

Light yellow oil, $[\alpha]_D^{25}$ = +27.19(*c* 1.21, DCM), 29.1 mg, 97% yield, 83% ee, dr>20:1, determined by HPLC (Chiralcel column AD-H, hexane/Ethanol = 85/15, flow rate 1.0 mL/min, UV detection at 254 nm), t_{major} = 8.2min, t_{minor} = 8.9min. **^{1}H NMR** (400 MHz, Chloroform-*d*) δ 7.65 (d, *J* = 8.0 Hz, 2H), 7.41 (dt, *J* = 15.2, 7.6 Hz, 4H), 7.29 (dd, *J* = 10.9, 7.5 Hz, 3H), 6.93 (d, *J* = 3.8 Hz, 1H), 6.78 (d, *J* = 3.8 Hz, 1H), 3.65 (dq, *J* = 7.0, 2.8, 2.3 Hz, 3H), 3.42 (dd, *J* = 9.3, 7.4 Hz, 1H), 3.20 (dd, *J* = 11.5, 9.3 Hz, 1H), 2.91 – 2.66 (m, 1H), 2.69 – 2.41 (m, 1H), 2.42 (s, 3H), 2.31 (dd, *J* = 13.2, 3.7 Hz, 1H), 2.20 (qd, *J* = 14.5, 8.9 Hz, 2H), 1.69 (td, *J* = 11.7, 7.4 Hz, 1H), 1.54 (d, *J* = 30.7 Hz, 3H), 1.36 – 1.11 (m, 2H). **^{13}C NMR** (101 MHz, Chloroform-*d*) δ 150.11, 145.24, 143.60, 134.64, 129.91, 129.81, 128.52, 127.30, 127.28, 125.28, 123.96, 111.74, 99.91, 80.68, 67.11, 52.57, 52.38, 49.12, 42.04, 34.29, 31.84, 31.42, 21.67, 20.85. **HRMS** (ESI): m/z calcd for $C_{28}H_{30}BrNO_4S_2Na$ ($[M+Na]^+$): 610.0692, found: 610.0696.

((3a*R*,11a*R*)-5-(5-chlorothiophen-2-yl)-11-phenyl-2-tosyldodecahydro-5,11-epoxyoxecino [4, 5-*c*] pyrrole (7q)

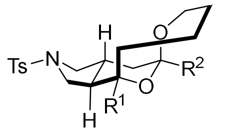

R1=Ph, R2=5-Cl-2-Thienyl

Light yellow oil, $[\alpha]_D^{25}$ = -3.04 (*c* 0.48, DCM), 26.7mg, 89% yield, 81% ee, dr>20:1, determined by HPLC (Chiralcel column AD-H, hexane/i-PrOH = 80/20, flow rate 1.0 mL/min, UV detection at 254 nm), t_{major} = 8.3min, t_{minor} = 12.2min. **^{1}H NMR** (600 MHz, Chloroform-*d*) δ 7.67 – 7.62 (m, 2H), 7.47 – 7.42 (m, 2H), 7.39 (t, *J* = 7.6 Hz, 2H), 7.29 (dd, *J* = 14.7, 7.7 Hz,

3H), 6.79 (d, J = 1.2 Hz, 2H), 3.71 – 3.63 (m, 3H), 3.42 (dd, J = 9.2, 7.5 Hz, 1H), 3.20 (dd, J = 11.5, 9.4 Hz, 1H), 2.69 (dd, J = 10.8, 9.2 Hz, 1H), 2.57 – 2.46 (m, 1H), 2.42 (s, 3H), 2.31 (dd, J = 13.3, 3.8 Hz, 1H), 2.24 (dd, J = 14.5, 10.2 Hz, 1H), 2.16 (dd, J = 14.6, 8.0 Hz, 1H), 1.69 (td, J = 11.8, 7.4 Hz, 1H), 1.65 – 1.42 (m, 3H), 1.30 – 1.16 (m, 2H). **^{13}C NMR** (151 MHz, Chloroform-*d*) δ 147.18, 145.26, 143.61, 134.66, 129.91, 129.45, 128.53, 127.30, 127.29, 126.05, 125.29, 122.95, 99.83, 80.68, 67.10, 52.58, 52.39, 49.12, 42.07, 34.31, 31.84, 31.42, 21.67, 20.85. **HRMS** (ESI): m/z calcd for $C_{28}H_{30}ClNO_4S_2Na$ ([M+Na]$^+$): 566.1197, found: 566.1197.

(4a*R*,12a*R*)-6-methyl-12-phenyl-2-tosyldodecahydro-1*H*-6,12-epoxyoxecino[5,4-*c*]pyridine (7r)

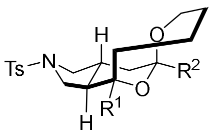

R1=Ph, R2= Me

Colorless oil, $[\alpha]_D^{25}$ = + 58.31 (*c* 1.08, DCM), 22.2 mg, 74% yield, 95% ee, determined by HPLC (Chiralcel column AD-H, hexane/i-PrOH = 90/10, flow rate 1.0 mL/min, UV detection at 254 nm), t_{major} = 12.0min, t_{minor} = 11.0min. **^{1}H NMR** (400 MHz, Acetone-d_6) δ 7.70 (d, J = 8.0 Hz, 2H), 7.45 (d, J = 7.3 Hz, 2H), 7.40 – 7.31 (m, 4H), 7.24 (t, J = 7.3 Hz, 1H), 3.85 (d, J = 11.7 Hz, 1H), 3.64 (dd, J = 9.4, 7.0 Hz, 2H), 3.26 – 3.16 (m, 2H), 2.76 (s, 1H), 2.41 (s, 3H), 2.27 (td, J = 8.6, 8.0, 4.6 Hz, 2H), 1.51 (td, J = 11.7, 11.2, 3.7 Hz, 4H), 1.44 (s, 3H), 1.35 (t, J = 12.6 Hz, 3H), 1.17 – 1.11 (m, 1H). **^{13}C NMR** (101 MHz, Acetone-d_6) δ 147.23, 144.21, 135.95, 130.56, 128.87, 128.19, 127.51, 126.27, 99.34, 79.58, 64.59, 53.73, 53.47, 49.92, 40.06, 34.50, 32.35, 31.98, 26.12, 21.93, 21.40. **HRMS** (ESI): m/z Calcd. For $C_{25}H_{31}NO_4SNa$ ([M+Na]$^+$): 464.1872, found: 464.1866 .

(3a*R*,4*S*,7*S*,8a*R*)-4-phenyl-2-tosyloctahydro-1*H*-4,7-epoxyoxepino[4,5-*c*]pyrrole (7s)

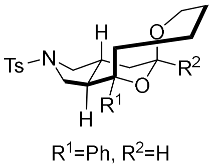

R1=Ph, R2=H

Colorless oil, $[\alpha]_D^{25}$ = + 57.35 (*c* 1.10, DCM), 27.3 mg, 91% yield, 91% ee, determined by HPLC (Chiralcel column AD-H, hexane/i-PrOH = 80/20, flow rate 1.0 mL/min, UV detection at 254 nm), t_{major} = 40.4min, t_{minor} = 31.6min. **^{1}H NMR** (400 MHz, Acetone-d_6) δ 7.74 – 7.64 (m, 2H), 7.44 (d, J = 7.7 Hz, 2H), 7.39 – 7.31 (m, 4H), 7.24 (t, J = 7.2 Hz, 1H), 5.08 (d, J = 3.6 Hz, 1H), 3.91 (d, J = 12.1 Hz, 1H), 3.64 (dd, J = 9.6, 7.1 Hz, 2H), 3.29 – 3.25 (m, 1H),

3.20 – 3.14 (m, 1H), 2.80 – 2.74 (m, 1H), 2.40 (s, 3H), 2.28 (dd, J = 14.4, 10.0 Hz, 1H), 2.19 (ddd, J = 11.7, 7.5, 4.2 Hz, 1H), 1.95 (dd, J = 12.9, 3.8 Hz, 1H), 1.64 – 1.30 (m, 6H), 1.19 (q, J = 6.6, 3.9 Hz, 1H). **^{13}C NMR** (101 MHz, Acetone-d_6) δ 147.22, 144.21, 135.89, 130.55, 128.88, 128.17, 127.53, 126.15, 98.05, 79.06, 69.87, 53.67, 53.53, 49.88, 33.87, 33.34, 32.36, 32.22, 21.70, 21.39. **HRMS** (ESI): m/z Calcd. For $C_{24}H_{29}NO_4SNa$ ($[M+Na]^+$): 450.1715, found: 450.1713.

(4a*R*, 12a*R*)-6, 12-diphenyl-2-tosyldodecahydro-1*H*-6, 12-epoxyoxecino [5,4-*c*] pyrrole (7t)

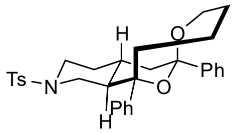

Light yellow oil, $[\alpha]_D^{25}$ = + 70.60 (*c* 1.10, DCM), 18.9 mg, 63% yield, 97% ee, dr>20:1, determined by HPLC (Chiralcel column AD-H, hexane/i-PrOH = 80/20, flow rate 1.0 mL/min, UV detection at 254 nm), t_{major} = 9.8min, t_{minor} = 5.9min. **^{1}H NMR** (600 MHz, Chloroform-*d*) δ 7.56 (d, J = 7.9 Hz, 4H), 7.49 (d, J = 8.0 Hz, 2H), 7.46 (t, J = 7.6 Hz, 2H), 7.36 (td, J = 7.7, 7.0, 3.9 Hz, 3H), 7.28 (dd, J = 7.8, 4.0 Hz, 3H), 3.81 (ddt, J = 12.0, 4.3, 2.1 Hz, 1H), 3.59 – 3.52 (m, 1H), 3.29 (td, J = 12.4, 2.2 Hz, 2H), 2.44 (s, 3H), 2.38 – 2.31 (m, 2H), 2.27 (dd, J = 14.7, 10.3 Hz, 1H), 2.15 – 2.02 (m, 3H), 1.76 – 1.65 (m, 2H), 1.60 (ddd, J = 16.4, 10.0, 3.3 Hz, 2H), 1.42 (ddd, J = 20.9, 9.6, 7.2 Hz, 2H), 1.38 – 1.29 (m, 1H), 1.26 – 1.16 (m, 1H). **^{13}C NMR** (151 MHz, Chloroform-*d*) δ 145.09, 144.12, 143.48, 133.98, 129.79, 128.56, 128.36, 127.74, 127.66 (2C), 126.99, 125.85, 100.99, 80.17, 66.75, 50.04, 46.43, 44.74, 31.79, 31.67, 30.14, 30.03, 29.85, 22.04, 21.72. **HRMS** (ESI): m/z calcd for $C_{31}H_{35}NO_4SNa$ ($[M+Na]^+$): 540.2179, found: 540.2177.

2.3 Synthesis and characterization of substrates **8a-n**

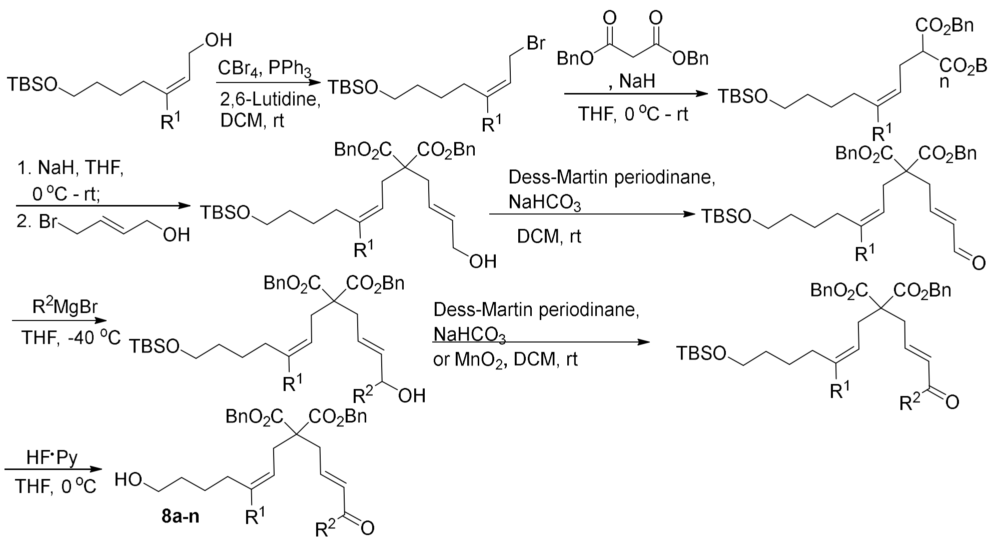

Dibenzyl-2-((*E*)-7-hydroxy-3-phenylhept-2-en-1-yl)-2-((*E*)-4-oxo-4-phenylbut-2-en-1-yl) malonate (8a)

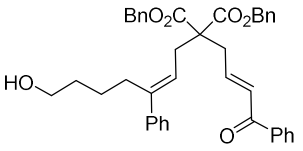

Light yellow oil. **^{1}H NMR** (400 MHz, Acetone-d_6) δ 8.06 – 7.85 (m, 2H), 7.65 (t, J = 7.4 Hz, 1H), 7.55 (t, J = 7.6 Hz, 2H), 7.45 – 7.34 (m, 8H), 7.34 – 7.23 (m, 7H), 7.13 (d, J = 15.3 Hz, 1H), 6.96 (dt, J = 15.2, 7.5 Hz, 1H), 5.57 (t, J = 7.4 Hz, 1H), 5.23 (s, 4H), 3.48 (t, J = 6.3 Hz, 2H), 3.06 (d, J = 7.5 Hz, 2H), 2.97 (d, J = 7.4 Hz, 2H), 2.54 (t, J = 7.7 Hz, 2H), 1.48 (tt, J = 9.8, 5.2 Hz, 2H), 1.38 (ddt, J = 14.6, 9.9, 5.3 Hz, 2H). **^{13}C NMR** (101 MHz, Acetone-d_6) δ 189.06, 169.97, 144.29, 142.72, 142.22, 137.65, 135.72, 132.82, 129.43, 128.64, 128.50, 128.45, 128.23 (2C), 128.21, 126.91, 126.41, 121.16, 67.04 (2C), 61.34, 57.65, 35.92, 32.65, 32.07, 24.97. **HRMS** (ESI): m/z calcd for $C_{40}H_{40}NO_6Na$ ([M+Na]$^+$): 639.2717, found: 639.2715.

Dibenzyl-2-((*E*)-4-(4-fluorophenyl)-4-oxobut-2-en-1-yl)-2-((*E*)-7-hydroxy-3-phenylhept-2-en-yl) malonate (8b)

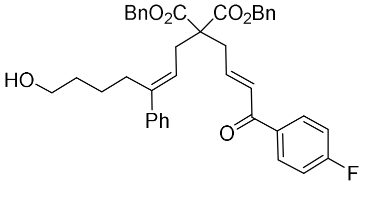

Light yellow oil. **^{1}H NMR** (400 MHz, Chloroform-d) δ 7.83 (dd, J = 8.5, 5.4 Hz, 2H), 7.36 – 7.21 (m, 13H), 7.22 – 7.15 (m, 2H), 7.09 (t, J = 8.5 Hz, 2H), 6.87 (dt, J = 15.0, 7.4 Hz, 1H), 6.74 (d, J = 15.3 Hz, 1H), 5.41 (t, J = 7.2 Hz, 1H), 5.20 – 5.07 (m, 4H), 3.51 (t, J = 6.4 Hz, 2H), 2.96 (d, J = 7.5 Hz, 2H), 2.91 (d, J = 7.3 Hz, 2H), 2.44 (t, J = 7.8 Hz, 2H), 1.48 (p, J = 6.7 Hz, 2H), 1.36 - 1.28

(m, 2H). **^{13}C NMR** (101 MHz, Chloroform-*d*) δ 188.63, 170.23, 165.70 (J = 255.5 Hz), 144.46, 142.89, 142.50, 135.17, 133.83 (J = 3.0 Hz), 131.33 (J = 9.1 Hz), 129.22, 128.66, 128.53, 128.41, 128.36, 127.18, 126.50, 121.09, 115.76 (J = 22.2 Hz), 67.54 (2C), 62.60, 57.76, 36.16, 32.57, 32.09, 24.83. **HRMS** (ESI): m/z calcd for $C_{40}H_{39}FO_6Na$ ($[M+Na]^+$): 657.2623, found: 657.2620.

Dibenzyl-2-((*E*)-7-hydroxy-3-phenylhept-2-en-1-yl)-2-((*E*)-4-(4-methoxyphenyl)-4-oxobut- 2-en-1-yl) malonate (8c)

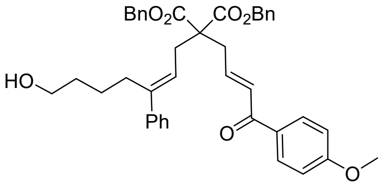

Light yellow oil. **^{1}H NMR** (400 MHz, Chloroform-*d*) δ 7.88 (d, J = 8.8 Hz, 2H), 7.31 (dq, J = 6.9, 3.9, 3.0 Hz, 13H), 7.24 (d, J = 8.4 Hz, 2H), 7.00 – 6.92 (m, 2H), 6.96 – 6.81 (m, 2H), 5.47 (t, J = 7.2 Hz, 1H), 5.24 – 5.12 (m, 4H), 3.91 (s, 3H), 3.55 (t, J = 6.4 Hz, 2H), 3.01 (d, J = 6.6 Hz, 2H), 2.96 (d, J = 7.3 Hz, 2H), 2.49 (t, J = 7.8 Hz, 2H), 1.52 (q, J = 7.0, 6.6 Hz, 2H), 1.43 – 1.33 (m, 2H). **^{13}C NMR (**101 MHz, Chloroform-*d*) δ 188.48, 170.27, 163.54, 144.35, 142.55, 141.48, 135.19, 131.03, 130.38, 129.39, 128.63, 128.47, 128.37, 128.32, 127.11, 126.49, 121.17, 113.86, 67.48 (2C), 62.56, 57.77, 55.55, 36.04, 32.56, 31.93, 24.82. **HRMS** (ESI): m/z calcd for $C_{41}H_{42}O_7Na$ ($[M+Na]^+$): 669.2823, found: 669.2827.

Dibenzyl-2-((*E*)-7-hydroxy-3-phenylhept-2-en-1-yl)-2-((*E*)-4-(3-methoxyphenyl)-4-oxobut- 2-en-1-yl) malonate (8d)

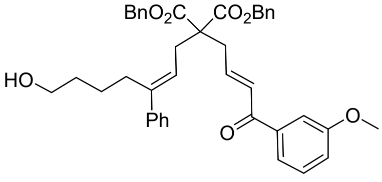

Light yellow oil. **^{1}H NMR** (400 MHz, Chloroform-*d*) δ 7.51 – 7.37 (m, 3H), 7.37 – 7.30 (m, 7H), 7.33 – 7.25 (m, 6H), 7.25 – 7.20 (m, 2H), 7.15 (dt, J = 7.7, 2.0 Hz, 1H), 6.92 (q, J = 7.4 Hz, 1H), 6.82 (d, J = 15.4 Hz, 1H), 5.46 (t, J = 7.2 Hz, 1H), 5.24 – 5.12 (m, 4H), 3.89 (s, 3H), 3.56 (d, J = 6.5 Hz, 2H), 3.01 (d, J = 7.3 Hz, 2H), 2.96 (d, J = 7.3 Hz, 2H), 2.49 (t, J = 7.8 Hz, 2H), 1.52 (dd, J = 8.7, 6.2 Hz, 2H), 1.43 – 1.32 (m, 2H). **^{13}C NMR** (101 MHz, Chloroform-*d*) δ 190.07, 170.26, 159.90, 144.44, 142.64, 142.54, 138.89, 135.18, 129.72, 129.61, 128.67, 128.53, 128.43, 128.36, 127.16, 126.52, 121.33, 121.13, 119.66, 112.90, 67.55 (2C),

62.62, 57.77, 55.55, 36.14, 32.57, 32.03, 24.83. **HRMS** (ESI): m/z calcd for $C_{41}H_{42}O_7Na$ ([M+Na]$^+$): 669.2823, found: 669.2828.

Dibenzyl-2-((*E*)-7-hydroxy-3-phenylhept-2-en-1-yl)-2-(*E*)-4-oxo-4-(*p*-tolyl) but-2-en-1-yl) malonate (8e)

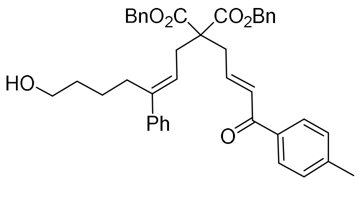

Light yellow oil. **^{1}H NMR** (400 MHz, Acetone-d_6) δ 7.85 (d, J = 8.1 Hz, 2H), 7.33 (dt, J = 7.8, 3.8 Hz, 12H), 7.30 – 7.19 (m, 5H), 7.09 (d, J = 15.3 Hz, 1H), 6.90 (dt, J = 15.1, 7.6 Hz, 1H), 5.53 (t, J = 7.3 Hz, 1H), 5.20 (s, 4H), 3.44 (q, J = 6.1 Hz, 2H), 3.05 – 2.98 (m, 2H), 2.93 (d, J = 7.4 Hz, 2H), 2.51 (t, J = 7.7 Hz, 2H), 2.40 (s, 3H), 1.51 – 1.39 (m, 2H), 1.41 – 1.30 (m, 2H). **^{13}C NMR** (101 MHz, Acetone-d_6) δ 189.34, 170.83, 145.12, 144.43, 143.58, 142.51, 136.58, 135.98, 130.28, 130.10, 129.45, 129.34, 129.07 (2C), 129.05, 127.75, 127.26, 122.02, 67.87 (2C), 62.19, 58.50, 36.73, 33.50, 32.88, 25.81, 21.56. **HRMS** (ESI): m/z calcd for $C_{41}H_{42}O_6Na$ ([M+Na]$^+$): 653.2874, found: 653.2877.

Dibenzyl-2-((*E*)-7-hydroxy-3-phenylhept-2-en-1-yl)-2-((*E*)-4-oxo-4-(*m*-tolyl) but-2-en-1-yl) malonate (8f)

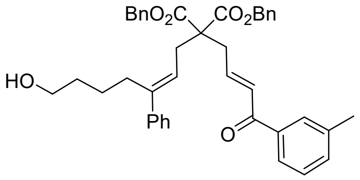

Light yellow oil. **^{1}H NMR** (400 MHz, Chloroform-*d*) δ 7.72 (s, 1H), 7.67 (d, J = 7.6 Hz, 1H), 7.50 – 7.37 (m, 2H), 7.41 – 7.29 (m, 8H), 7.32 – 7.21 (m, 7H), 6.92 (q, J = 7.3, 6.8 Hz, 1H), 6.86 (d, J = 15.4 Hz, 1H), 5.47 (t, J = 7.2 Hz, 1H), 5.26 – 5.13 (m, 4H), 3.58 (d, J = 6.6 Hz, 2H), 3.02 (d, J = 6.9 Hz, 2H), 2.97 (d, J = 7.3 Hz, 2H), 2.46 (s, 5H), 1.55 (q, J = 7.7, 7.2 Hz, 2H), 1.44 – 1.33 (m, 2H). **^{13}C NMR** (101 MHz, Chloroform-*d*) δ 190.46, 170.30, 144.44, 142.58, 142.41, 138.51, 137.58, 135.21, 133.81, 129.80, 129.30, 128.69, 128.55, 128.52, 128.44, 128.38, 127.18, 126.54, 125.97, 121.19, 67.57 (2C), 62.68, 57.79, 36.13, 32.60, 32.00, 24.85, 21.48. **HRMS** (ESI): m/z calcd for $C_{41}H_{42}O_6Na$ ([M+Na]$^+$): 653.2874, found: 653.2875.

Dibenzyl2-((*E*)-4-(3,5-dimethylphenyl)-4-oxobut-2-en-1-yl)-2-((*E*)-7-hydroxy-3-phenylhept-2-en-1-yl) malonate (8g)

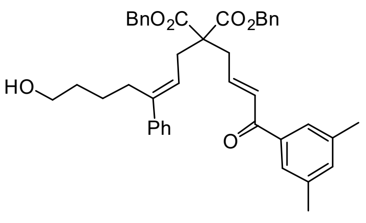

Colorless oil. ^{1}H NMR (400 MHz, Acetone-d_6) δ 7.56 (s, 2H), 7.32 (q, J = 3.8, 3.1 Hz, 7H), 7.29 (d, J = 6.4 Hz, 5H), 7.28 – 7.18 (m, 2H), 7.09 (d, J = 15.2 Hz, 1H), 6.92 – 6.84 (m, 1H), 5.53 (td, J = 7.5, 2.2 Hz, 1H), 5.20 (s, 4H), 3.49 – 3.39 (m, 2H), 3.02 (d, J = 7.6 Hz, 2H), 2.93 (dd, J = 7.5, 2.3 Hz, 2H), 2.50 (t, J = 7.7 Hz, 2H), 2.35 (s, 6H), 1.44 (q, J = 6.9, 6.5 Hz, 2H), 1.40 – 1.25 (m, 2H). **^{13}C NMR** (101 MHz, Acetone-d_6) δ 187.94, 169.94, 144.30, 142.90, 142.68, 138.52, 136.20, 135.71, 130.21, 129.03, 128.82, 128.49, 128.24, 128.23, 128.20, 126.91, 126.40, 121.12, 67.04 (2C), 61.32, 57.61, 35.94, 32.64, 32.11, 24.97, 20.39. **HRMS** (ESI): m/z Calcd. For $C_{42}H_{44}O_6Na$ ([M+Na]$^+$): 667.3030, found: 667.3036.

Dibenzyl2-((*E*)-4-(4-chlorophenyl)-4-oxobut-2-en-1-yl)-2-((*E*)-7-hydroxy-3-phenylhept-2-en-1-yl) malonate (8h)

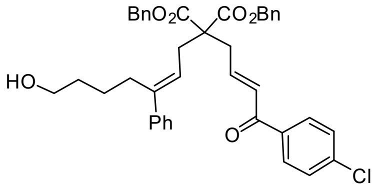

Colorless oil. **^{1}H NMR** (400 MHz, Chloroform-*d*) δ 7.79 (d, J = 8.3 Hz, 2H), 7.46 (d, J = 8.4 Hz, 2H), 7.42 – 7.28 (m, 13H), 7.30 – 7.21 (m, 2H), 6.94 (dt, J = 15.1, 7.5 Hz, 1H), 6.78 (d, J = 15.3 Hz, 1H), 5.48 (t, J = 7.2 Hz, 1H), 5.26 – 5.13 (m, 4H), 3.57 (t, J = 6.4 Hz, 2H), 3.02 (d, J = 7.5 Hz, 2H), 2.97 (d, J = 7.3 Hz, 2H), 2.50 (t, J = 7.8 Hz, 2H), 1.54 (d, J = 6.7 Hz, 2H), 1.39 (t, J = 7.7 Hz, 2H). **^{13}C NMR** (101 MHz, Chloroform-*d*) δ 189.01, 170.20, 144.46, 143.27, 142.48, 139.35, 135.78, 135.15, 130.12, 129.18, 128.94, 128.66, 128.54, 128.42, 128.35, 127.18, 126.48, 121.06, 67.54 (2C), 62.58, 57.74, 36.20, 32.55, 32.12, 24.82. **HRMS** (ESI): m/z Calcd. For $C_{40}H_{39}ClO_6Na$ ([M+Na]$^+$): 673.2327, found: 673.2325.

Dibenzyl-2-((*E*)-4-(3-chlorophenyl)-4-oxobut-2-en-1-yl)-2-((*E*)-7-hydroxy-3-phenylhept-2-en-yl) malonate (8i)

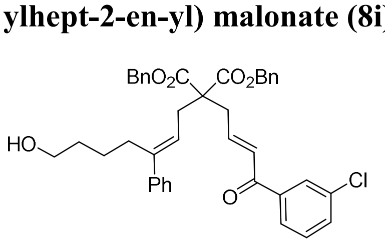

Light yellow oil. **^{1}H NMR** (400 MHz, Chloroform-*d*) δ 7.92 – 7.82 (m, 1H), 7.73 (d, J = 7.8 Hz, 1H), 7.58 (dd, J = 7.8, 2.1 Hz, 1H), 7.44 (d, J = 7.6 Hz, 1H), 7.44 – 7.25 (m, 13H), 7.25 (d, J = 8.0 Hz, 2H), 6.96 (dt, J = 15.2, 7.6 Hz, 1H), 6.77 (d, J = 15.3 Hz, 1H), 5.47 (t, J = 7.2 Hz, 1H), 5.29 – 5.16 (m, 4H), 3.58

(d, J = 6.5 Hz, 2H), 3.01 (d, J = 7.5 Hz, 2H), 2.97 (d, J = 7.2 Hz, 2H), 2.51 (t, J = 7.8 Hz, 2H), 1.60 – 1.48 (m, 2H), 1.45 – 1.32 (m, 2H). **^{13}C NMR** (101 MHz, Chloroform-*d*) δ 188.87, 170.20, 144.49, 143.70, 142.49, 139.08, 135.14, 134.92, 132.88, 129.98, 129.08, 128.78, 128.67, 128.56, 128.46, 128.37, 127.19, 126.77, 126.50, 121.06, 67.58 (2C), 62.61, 57.73, 36.20, 32.55, 32.14, 24.82. **HRMS** (ESI): m/z calcd for $C_{40}H_{39}O_6ClNa$ ([M+Na]$^+$): 673.2327, found: 673.2323.

Dibenzyl-2-((*E*)-4-(3-fluorophenyl)-4-oxobut-2-en-1-yl)-2-((*E*)-7-hydroxy-3-phenylhept-2-en-yl) malonate (8j)

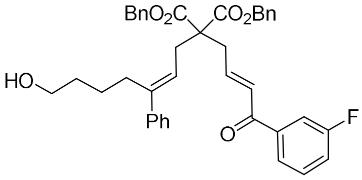

Light yellow oil. **^{1}H NMR** (400 MHz, Chloroform-*d*) δ 7.60 (d, J = 8.0 Hz, 1H), 7.53 (dt, J = 9.4, 2.1 Hz, 1H), 7.48 – 7.38 (m, 1H), 7.33 - 7.26 (m, 14H), 7.23 – 7.18 (m, 2H), 6.93 (dt, J = 15.1, 7.5 Hz, 1H), 6.75 (d, J = 15.3 Hz, 1H), 5.43 (t, J = 7.2 Hz, 1H), 5.19 (d, J = 12.1 Hz, 2H), 5.13 (d, J = 12.1 Hz, 2H), 3.54 (t, J = 6.5 Hz, 2H), 2.98 (d, J = 7.5 Hz, 2H), 2.93 (d, J = 7.2 Hz, 2H), 2.47 (t, J = 7.8 Hz, 2H), 1.57 – 1.43 (m, 2H), 1.43 – 1.28 (m, 2H). **^{13}C NMR** (101 MHz, Chloroform-*d*) δ 188.77 (J = 2.0 Hz), 170.14, 162.78 (J = 249.5 Hz), 144.42, 143.49, 142.43, 139.52 (J = 6.1 Hz), 135.08, 130.24 (J = 8.1 Hz), 129.04, 128.60, 128.49, 128.38, 128.30, 127.12, 126.43, 124.34 (J = 3.0 Hz), 121.00, 119.92 (J = 21.2 Hz), 115.40 (J = 22.2 Hz), 67.50 (2C), 62.54, 57.67, 36.11, 32.48, 32.05, 24.75. **HRMS** (ESI): m/z calcd for $C_{40}H_{39}O_6FNa$ ([M+Na]$^+$): 657.2623, found: 657.2621.

Dibenzyl-2-((*E*)-7-hydroxy-3-phenylhept-2-en-1-yl)-2-((*E*)-4-oxopent-2-en-1-yl) malonate (8k)

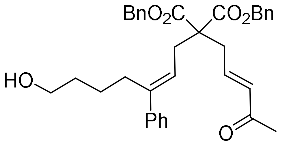

Light yellow oil. **^{1}H NMR** (400 MHz, Chloroform-*d*) δ 7.40 – 7.31 (m, 3H), 7.35 – 7.22 (m, 10H), 7.24 – 7.13 (m, 2H), 6.56 (dq, J = 15.4, 7.9 Hz, 1H), 6.01 (d, J = 15.9 Hz, 1H), 5.39 (t, J = 7.3 Hz, 1H), 5.19 (d, J = 12.2 Hz, 2H), 5.14 (d, J = 12.1 Hz, 2H), 3.56 (t, J = 6.4 Hz, 2H), 2.86 (td, J = 5.1, 2.5 Hz, 4H), 2.46 (t, J = 7.8 Hz, 2H), 2.08 (s, 3H), 1.57 – 1.41 (m, 2H), 1.40 – 1.28 (m, 2H). **^{13}C NMR** (101 MHz, Chloroform-*d*) δ 198.28, 170.24, 144.54, 142.52, 141.67, 135.19, 134.72, 128.75, 128.66, 128.59, 128.39, 127.22,

126.54, 121.03, 67.61 (2C), 62.70, 57.64, 36.10, 32.62, 32.30, 26.85, 24.81. **HRMS** (ESI): m/z Calcd. For $C_{35}H_{38}O_6Na$ ($[M+Na]^+$): 577.2561, found: 577.2561.

Dibenzyl2-((*E*)-7-hydroxy-3-phenylhept-2-en-1-yl)-2-((*E*)-4-oxohex-2-en-1-yl) malonate (8l)

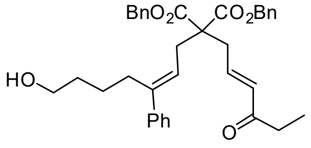

Colorless oil. ^{1}**H NMR** (400 MHz, Chloroform-*d*) δ 7.23 (q, J = 3.6, 3.1 Hz, 6H), 7.21 – 7.12 (m, 7H), 7.14 – 7.07 (m, 2H), 6.49 (dt, J = 15.4, 7.5 Hz, 1H), 5.93 (d, J = 15.8 Hz, 1H), 5.29 (t, J = 7.3 Hz, 1H), 5.09 (d, J = 12.2 Hz, 2H), 5.04 (d, J = 12.2 Hz, 2H), 3.46 (t, J = 6.4 Hz, 2H), 2.77 (t, J = 7.1 Hz, 4H), 2.36 (t, J = 7.7 Hz, 2H), 2.29 (q, J = 7.3 Hz, 2H), 1.44 – 1.35 (m, 2H), 1.29 – 1.21 (m, 2H), 0.94 (t, J = 7.3 Hz, 3H). ^{13}**C NMR** (101 MHz, Chloroform-*d*) δ 200.72, 170.24, 144.46, 142.52, 140.20, 135.19, 133.69, 128.70, 128.59, 128.55, 128.36, 127.17, 126.51, 121.06, 67.54 (2C), 62.64, 57.65, 35.99, 33.04, 32.60, 32.17, 24.81, 8.00. **HRMS** (ESI): m/z Calcd. For $C_{36}H_{40}O_6Na$ ($[M+Na]^+$): 591.2717, found: 591.2720.

Dibenzyl2-((*E*)-4-cyclopentyl-4-oxobut-2-en-1-yl)-2-((*E*)-7-hydroxy-3-phenyl-hept-2-en-1-yl) malonate (8m)

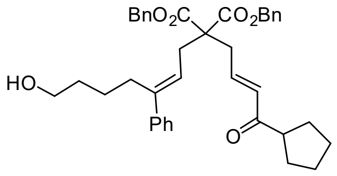

Colorless oil. ^{1}**H NMR** (400 MHz, Acetone-*d*$_6$) δ 7.34 (s, 9H), 7.34 – 7.08 (m, 6H), 6.68 (dt, J = 15.5, 7.6 Hz, 1H), 6.23 – 6.13 (m, 1H), 5.49 (t, J = 7.3 Hz, 1H), 5.19 (s, 4H), 3.46 (t, J = 6.0 Hz, 2H), 3.09 – 2.96 (m, 1H), 2.89 (dd, J = 7.4, 4.4 Hz, 4H), 2.52 (d, J = 7.8 Hz, 2H), 1.75 - 1.65 (m, 3H), 1.65 – 1.47 (m, 5H), 1.48 – 1.41 (m, 2H), 1.42 – 1.29 (m, 2H). ^{13}**C NMR** (101 MHz, Acetone-*d*$_6$) δ 201.49, 170.77, 140.71, 136.62, 134.15, 129.37, 129.15, 129.11, 129.06, 127.76, 127.24, 122.02, 67.87 (2C), 62.22, 58.45, 48.91, 36.57, 33.55, 32.81, 29.61, 26.72, 25.85. **HRMS** (ESI): m/z Calcd. For $C_{39}H_{44}O_6Na$ ($[M+Na]^+$): 631.3030, found: 631.3034.

Dibenzyl2-((*E*)-4-cyclohexyl-4-oxobut-2-en-1-yl)-2-((*E*)-7-hydroxy-3-phenylhept-2-en-1-yl) malonate (8n)

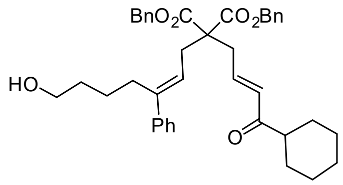

Colorless oil. **^{1}H NMR** (400 MHz, Acetone-d_6) δ 7.34 (s, 9H), 7.34 – 7.19 (m, 6H), 6.72 (dt, J = 15.4, 7.6 Hz, 1H), 6.24 (dt, J = 15.7, 1.4 Hz, 1H), 5.49 (t, J = 7.4 Hz, 1H), 5.19 (s, 4H), 3.47 (q, J = 6.3 Hz, 2H), 2.93 – 2.85 (m, 4H), 2.51 (t, J = 7.8 Hz, 3H), 1.78 – 1.69 (m, 4H), 1.69 – 1.59 (m, 1H), 1.54 – 1.42 (m, 2H), 1.42 – 1.33 (m, 2H), 1.34 – 1.21 (m, 4H), 1.24 – 1.12 (m, 1H). **^{13}C NMR** (101 MHz, Acetone-d_6) δ 202.06, 170.77, 145.09, 143.58, 140.28, 136.62, 133.11, 129.37, 129.10, 129.08, 129.06, 127.76, 127.24, 122.01, 67.84, 62.22, 58.46, 48.73, 36.48, 33.55, 32.77, 29.30, 26.64, 26.25, 25.86. **HRMS** (ESI): m/z Calcd. For $C_{40}H_{46}O_6Na$ ([M+Na]$^+$): 645.3187, found: 645.3186.

2.4 General procedure for IEDHDA-keto acetalization reactions with **8a-n**

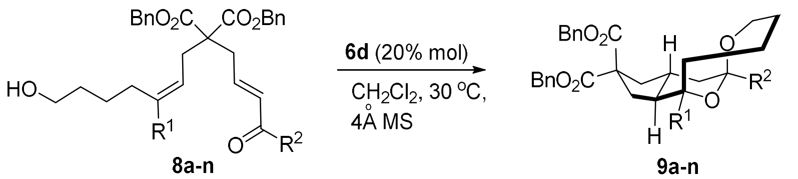

8a-n **9a-n**

Substrate **8a-n** (30 mg, 1.0 eq.) was dissolved in DCM (1.0 ml), then 4Å molecular sieves (60 mg) and catalyst **6d** (0.2 eq.) were added. The reaction mixture was stirred at 30 °C and monitored by TLC (generally for 48~60 hours). When the reaction was completed, the mixture was purified by silica gel column to afford products **9a-n**.

Benzyl (8a*S*, 10*R*, 11a*R*)-10-((benzylperoxy)-λ^2-methyl)-2, 8- diphenyl-dodecahydro-2, 8-epoxycyclopenta [*d*] oxecine-10-carboxylate (9a)

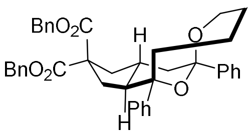

Light yellow oil, [α]$_D$^{25} = +19.75 (*c* 2.98, DCM), 28.2 mg, 94% yield, 96% ee, dr > 20:1, determined by HPLC (Chiralcel column AD-H, hexane/i-PrOH = 50/50, flow rate 1.0 mL/min, UV detection at 254 nm), t_{major} = 26.6 min, t_{minor} = 17.5 min. **^{1}H NMR** (600 MHz, Acetone-d_6) δ 7.74 – 7.69 (m, 2H), 7.65 (d, J = 7.6 Hz, 2H), 7.46 – 7.36 (m, 5H), 7.35 (dq, J = 7.3, 3.9, 2.9 Hz, 4H), 7.32 – 7.29 (m, 2H), 7.27 (dd, J = 3.8, 2.2 Hz, 3H), 7.24 – 7.18 (m, 2H), 5.21 (d, J = 12.4 Hz, 1H), 5.17 (d, J = 12.4 Hz, 1H), 5.08 – 5.00 (m, 2H), 3.60 (dt, J = 13.4, 3.3 Hz, 1H), 3.35 (td, J = 12.3, 2.3 Hz, 1H), 2.63 (dd, J = 12.9, 6.5 Hz, 1H), 2.49 – 2.41 (m, 2H), 2.38 (td, J = 13.9, 13.4, 7.1 Hz, 2H), 2.31 (dd, J = 13.2, 3.8

Hz, 1H), 2.15 (t, J = 12.9 Hz, 1H), 1.72 – 1.55 (m, 4H), 1.48 – 1.41 (m, 1H), 1.39 – 1.31 (m, 1H), 1.26 – 1.16 (m, 1H). **^{13}C NMR** (151 MHz, Acetone-d_6) δ 172.73, 172.33, 147.68, 146.64, 136.94, 136.85, 129.35, 129.27, 129.02 (2C), 128.95, 128.93, 128.88, 128.67, 128.25, 127.47, 126.75, 126.72, 102.26, 82.01, 67.80, 67.58, 67.24, 58.07, 54.51, 45.42, 40.66, 36.32, 35.85, 32.40, 31.87, 21.99. **HRMS** (ESI): m/z calcd for $C_{40}H_{40}O_6Na$ ([M+Na]$^+$): 639.2717, found: 639.2718.

Benzyl (8a*S*, 10*R*, 11a*R*)-10-((benzylperoxy)-λ^2-methyl)-2-(4-fluorophenyl)-8-phenyldodecahydro-2, 8-epoxycyclopenta [*d*] oxecine-10-carboxylate (9b)

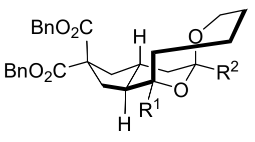

Light yellow oil, $[\alpha]_D^{25}$ = +18.69 (c 2.50, DCM), 28.5 mg, 95% yield, 97% ee, dr > 20:1, determined by HPLC (Chiralcel column AD-H, hexane/i-PrOH = 97/3, flow rate 1.0 mL/min, UV detection at 254 nm), t_{major} = 21.1min, t_{minor} = 14.4min. **^{1}H NMR** (600 MHz, Chloroform-d) δ 7.58 (t, J = 8.4 Hz, 4H), 7.38 (t, J = 7.8 Hz, 2H), 7.33 – 7.28 (m, 3H), 7.28 – 7.24 (m, 2H), 7.24 – 7.19 (m, 4H), 7.14 – 7.08 (m, 2H), 7.08 – 7.01 (m, 2H), 5.15 (d, J = 12.2 Hz, 1H), 5.09 (d, J = 12.1 Hz, 1H), 5.02 (d, J = 12.4 Hz, 1H), 4.97 (d, J = 12.4 Hz, 1H), 3.61 – 3.58(m, 1H), 3.38 (td, J = 12.2, 2.0 Hz, 1H), 2.57 (dd, J = 13.1, 6.5 Hz, 1H), 2.47 – 2.38 (m, 1H), 2.39 – 2.30 (m, 3H), 2.29 (dd, J = 13.3, 3.8 Hz, 1H), 2.16 (t, J = 12.9 Hz, 1H), 1.72 – 1.62 (m, 2H), 1.64 – 1.57 (m, 2H), 1.45 – 1.42 (m, 1H), 1.33 (t, J = 12.7 Hz, 1H), 1.28 – 1.19 (m, 1H). **^{13}C NMR** (151 MHz, Chloroform-d) δ 172.33, 172.03, 162.30 (J = 246.1 Hz), 146.48, 141.46 (J = 3.0 Hz), 135.53 (J = 7.6 Hz), 128.67, 128.60, 128.43, 128.30, 128.28, 128.24, 127.95, 127.80, 127.75, 126.85, 125.91, 115.09 (J = 21.1 Hz), 101.22, 81.50, 67.47, 67.28, 66.78, 57.53, 53.52, 44.86, 40.04, 35.74, 35.08, 31.69, 31.36, 21.22. **^{19}F NMR** (471 MHz, Chloroform-d) δ -115.45 (s, 1F). **HRMS** (ESI): m/z calcd for $C_{40}H_{39}O_6FNa$ ([M+Na]$^+$): 657.2623, found: 657.2623.

Benzyl (8a*S*, 10*R*, 11a*R*)-10-((benzylperoxy)-λ^2-methyl)-2-(4-methoxyphenyl)-8-phenyldodecahydro-2, 8-epoxycyclopenta [*d*] oxecine-10-carboxylate (9c)

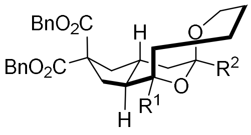

R¹=Ph,R²=4-MeO-Ph

Light yellow oil, [α]D [25] = +10.26 (*c* 2.50, DCM), 26.7 mg, 89% yield, 94% ee, dr > 20:1, determined by HPLC (Chiralcel column AD-H, hexane/i-PrOH = 97/3, flow rate 1.0 mL/min, UV detection at 254 nm), t_{major} = 16.8 min, t_{minor} = 12.9 min. **^{1}H NMR** (400 MHz, Chloroform-*d*) *δ* 7.71 – 7.64 (m, 2H), 7.59 (d, *J* = 8.2 Hz, 2H), 7.54 – 7.38 (m, 3H), 7.38 – 7.33 (m, 2H), 7.33 – 7.23 (m, 6H), 7.22 – 7.10 (m, 2H), 7.01 – 6.94 (m, 2H), 5.26 – 5.12 (m, 2H), 5.06 (q, *J* = 12.4 Hz, 2H), 3.88 (s, 3H), 3.67 (d, *J* = 11.8 Hz, 1H), 3.49 (td, *J* = 12.2, 2.0 Hz, 1H), 2.64 (dd, *J* = 13.0, 6.4 Hz, 1H), 2.61 – 2.45 (m, 1H), 2.45 – 2.31 (m, 4H), 2.24 (t, *J* = 12.8 Hz, 1H), 1.80 – 1.59 (m, 4H), 1.57 – 1.46 (m, 1H), 1.41 (t, *J* = 12.7 Hz, 1H), 1.29 (d, *J* = 19.5 Hz, 1H). **^{13}C NMR** (101 MHz, Chloroform-*d*) *δ* 172.36, 172.07, 159.03, 146.72, 137.91, 135.57, 135.52, 128.65, 128.62, 128.41, 128.27, 128.23, 128.21, 127.93, 127.21, 126.74, 125.95, 113.60, 101.40, 81.27, 67.44, 67.24, 66.65, 57.58, 55.39, 53.57, 44.93, 40.08, 35.76, 35.16, 31.75, 31.47, 21.22. **HRMS** (ESI): m/z calcd for $C_{41}H_{42}O_7Na$ ([M+Na]$^+$): 669.2828, found: 669.2827.

Benzyl (8a*S*, 10*R*, 11a*R*)-10-((benzylperoxy)-λ²-methyl)-2-(3-methoxyphenyl)-8-phenyldodecahydro-2, 8-epoxycyclopenta [*d*] oxecine-10-carboxylate (9d)

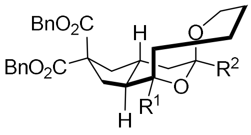

R¹=Ph,R²=3-MeO-Ph

Light yellow oil, [α]D [25] =+10.04 (*c* 2.33, DCM), 28.8 mg, 96% yield, 88 % ee, dr > 20:1, determined by HPLC (Chiralcel column AD-H, hexane/i-PrOH = 97/3, flow rate 1.0 mL/min, UV detection at 254 nm), t_{major} = 27.2 min, t_{minor} = 24.3 min. **^{1}H NMR** (600 MHz, Chloroform-*d*) *δ* 7.72 – 7.66 (m, 2H), 7.49 – 7.39 (m, 4H), 7.40 – 7.34 (m, 4H), 7.34 – 7.28 (m, 6H), 7.26 – 7.14 (m, 2H), 6.90 (dd, *J* = 8.3, 2.6 Hz, 1H), 5.22 (d, *J* = 12.2 Hz, 1H), 5.16 (d, *J* = 12.2 Hz, 1H), 5.09 (d, *J* = 12.4 Hz, 1H), 5.04 (d, *J* = 12.4 Hz, 1H), 3.90 (s, 3H), 3.71 (d, *J* = 12.1 Hz, 1H), 3.50 (td, *J* = 12.3, 2.0 Hz, 1H), 2.64 (dd, *J* = 13.1, 6.5 Hz, 1H), 2.54 – 2.43 (m, 1H), 2.42 (t, *J* = 6.1 Hz, 3H), 2.38 (dd, *J* = 13.3, 3.8 Hz, 1H), 2.25 (t, *J* = 12.9 Hz, 1H), 1.78 – 1.64 (m, 4H), 1.57 – 1.47 (m, 1H), 1.44 (t, *J* = 12.7 Hz, 1H), 1.35 – 1.29 (m, 1H). **^{13}C NMR** (151 MHz, Chloroform-*d*) *δ* 172.36, 172.06, 159.69, 147.37, 146.66, 135.56, 135.52, 129.38, 128.67, 128.60, 128.43, 128.29, 128.24, 128.23, 127.95, 126.76, 125.95, 118.44, 113.00, 111.77,

101.43, 81.38, 67.46, 67.26, 66.96, 57.55, 55.32, 53.49, 44.65, 40.08, 35.78, 35.10, 31.73, 31.52, 21.20. **HRMS** (ESI): m/z calcd for $C_{41}H_{42}O_7Na$ ($[M+Na]^+$): 669.2823, found: 669.2828.

Benzyl (8a*S*, 10*R*, 11a*R*)-10-((benzylperoxy)-λ²-methyl)-8-phenyl-2-(*p*-tolyl) dodecahydro-2, 8-epoxycyclopenta [*d*] oxecine-10-carboxylate (9e)

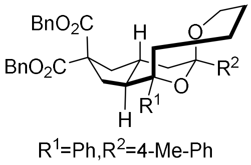

Light yellow oil, $[\alpha]_D^{25}$ = +16.57 (*c* 1.89, DCM), 25.2 mg, 84% yield, 97% ee, dr > 20:1, determined by HPLC (Chiralcel column AD-H, hexane/i-PrOH = 50/50, flow rate 1.0 mL/min, UV detection at 254 nm), t_{major} = 30.7 min, t_{minor} = 25.7 min. **^{1}H NMR** (400 MHz, Chloroform-*d*) δ 7.61 (d, *J* = 7.7 Hz, 2H), 7.49 (d, *J* = 7.7 Hz, 2H), 7.37 (t, *J* = 7.7 Hz, 2H), 7.29 (ddt, *J* = 11.7, 8.3, 6.3 Hz, 5H), 7.24 (d, *J* = 2.5 Hz, 3H), 7.21 (d, *J* = 10.4 Hz, 3H), 7.11 (dd, *J* = 7.3, 2.4 Hz, 2H), 5.12 (d, *J* = 11.8 Hz, 2H), 5.06 – 4.96 (m, 2H), 3.61 (d, *J* = 11.4 Hz, 1H), 3.42 (td, *J* = 12.2, 2.0 Hz, 1H), 2.57 (dd, *J* = 12.9, 6.4 Hz, 1H), 2.54 – 2.36 (m, 1H), 2.37-2.35(m, 5H), 2.36 – 2.25 (m, 2H), 2.18 (t, *J* = 12.9 Hz, 1H), 1.81 – 1.44 (m, 4H), 1.48 – 1.38 (m, 1H), 1.34 (t, *J* = 12.6 Hz, 1H), 1.31 – 1.17 (m, 1H). **^{13}C NMR** (101 MHz, Chloroform-*d*) δ 172.38, 172.09, 146.76, 142.72, 135.58, 135.53, 129.02, 128.67, 128.64, 128.60, 128.42, 128.29, 128.25, 128.22, 128.11, 127.95, 126.74, 125.98, 101.54, 81.27, 67.46, 67.26, 66.81, 57.58, 53.55, 44.86, 40.11, 35.79, 35.15, 31.76, 31.54, 21.26 (2C). **HRMS** (ESI): m/z calcd for $C_{41}H_{42}O_6Na$ ($[M+Na]^+$): 653.2874, found: 653.2877.

Benzyl (8a*S*, 10*R*, 11a*R*)-10-((benzylperoxy)-λ²-methyl)-8-phenyl-2-(*m*-tolyl) dodecahydro-2, 8-epoxycyclopenta [*d*] oxecine-10-carboxylate (9f)

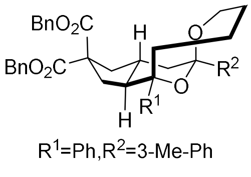

Light yellow oil, $[\alpha]_D^{25}$ = +13.92 (*c* 0.13, DCM), 20.4 mg, 68% yield, 87 % ee, dr > 20:1, determined by HPLC (Chiralcel column AD-H, hexane/i-PrOH = 95/5, flow rate 1.0 mL/min, UV detection at 254 nm), t_{major} = 8.4 min, t_{minor} = 9.3 min. **^{1}H NMR** (400 MHz, Chloroform-*d*) δ 7.54 (d, *J* = 7.7 Hz, 2H), 7.45 – 7.27 (m, 4H), 7.23 (dt, *J* = 7.4, 2.9 Hz, 5H), 7.16 (td, *J* = 7.0, 3.9 Hz, 6H), 7.03 (q, *J* = 3.4, 2.7 Hz, 2H), 5.04 (q, *J* = 12.3 Hz, 2H), 4.92 (q, *J* = 12.5 Hz, 2H), 3.55 (d, *J* = 11.5 Hz, 1H), 3.34 (td, *J* = 12.2, 2.1 Hz, 1H), 2.49 (dd,

J = 13.0, 6.5 Hz, 1H), 2.40 – 2.31 (m, 1H), 2.30 (s, 3H), 2.31 – 2.19 (m, 4H), 2.09 (t, J = 12.8 Hz, 1H), 1.67 – 1.47 (m, 4H), 1.41 – 1.31 (m, 1H), 1.33 – 1.16 (m, 1H), 1.19 – 1.10 (m, 1H). **^{13}C NMR** (151 MHz, Chloroform-*d*) δ 172.39, 172.09, 146.73, 145.59, 137.87, 135.58, 135.54, 133.60, 128.67, 128.65, 128.60, 128.43, 128.33, 128.29, 128.25, 128.23, 127.95, 126.76, 126.00, 123.13, 101.57, 81.32, 67.46, 67.26, 66.91, 57.57, 53.57, 44.84, 40.11, 35.80, 35.13, 31.75, 31.48, 21.86, 21.24. **HRMS** (ESI): m/z calcd for $C_{41}H_{42}O_6Na$ ([M+Na]$^+$): 653.2874, found: 653.2876.

Benzyl (8a*S*, 10*R*, 11a*R*)-10-((benzylperoxy)-λ^2-methyl)-2-(3, 5-dimethylphenyl)-8-phenyldodecahydro-2,8-epoxycyclopenta[*d*]oxecine-10-carboxylate (9g)

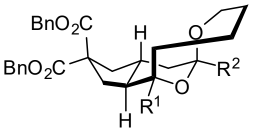

Colorless oil, $[\alpha]_D^{25}$ = +14.97 (*c* 1.14, DCM), 24.9 mg, 83% yield, 99% ee, determined by HPLC (Chiralcel column AD-H, hexane/i-PrOH = 90/10, flow rate 1.0 mL/min, UV detection at 254 nm), t_{major} = 4.5 min, t_{minor} = 5.0 min. **^{1}H NMR** (400 MHz, Chloroform-*d*) δ 7.56 (d, J = 7.8 Hz, 2H), 7.31 (d, J = 7.7 Hz, 2H), 7.24 (dt, J = 7.0, 3.0 Hz, 3H), 7.22 – 7.16 (m, 5H), 7.18 – 7.04 (m, 3H), 7.08 – 6.98 (m, 2H), 6.86 (s, 1H), 5.09 (d, J = 12.3 Hz, 1H), 5.04 (s, 1H), 4.96 (d, J = 12.5 Hz, 1H), 4.90 (d, J = 12.4 Hz, 1H), 3.56 (dd, J = 11.7, 3.4 Hz, 1H), 3.36 (td, J = 12.2, 2.0 Hz, 1H), 2.50 (dd, J = 13.0, 6.4 Hz, 1H), 2.44 – 2.29 (m, 1H), 2.27-2.25 (m, 9H), 2.21 (s, 1H), 2.10 (t, J = 12.8 Hz, 1H), 1.68 – 1.45 (m, 4H), 1.42 – 1.32 (m, 1H), 1.28 (t, J = 12.7 Hz, 1H), 1.19 (d, J = 6.3 Hz, 1H). **^{13}C NMR** (101 MHz, Chloroform-*d*) δ 172.39, 172.08, 146.78, 145.60, 137.76, 135.60, 135.56, 129.19, 128.67, 128.59, 128.42, 128.28, 128.23, 128.22, 127.94, 126.73, 126.02, 123.83, 101.61, 81.28, 67.45, 67.24, 66.90, 57.58, 53.59, 44.89, 40.13, 35.84, 35.13, 31.75, 31.46, 21.73 (2C), 21.26. **HRMS** (ESI): m/z Calcd. For $C_{42}H_{44}O_6Na$ ([M+Na]$^+$): 667.3030, found: 667.3037.

Benzyl (8a*S*, 10*R*, 11a*R*)-10-((benzylperoxy)-λ^2-methyl)-2-(4-chlorophenyl)-8-phenyldodecahydro-2, 8-epoxycyclopenta [*d*] oxecine-10-carboxylate (9h)

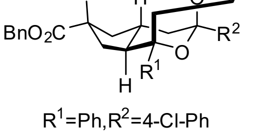

Colorless oil, $[\alpha]_D^{25}$ = +14.37 (*c* 1.21, DCM), 26.7 mg, 89% yield, 94% ee, dr>20:1, determined by HPLC (Chiralcel column AD-H, hexane/i-PrOH = 95/5, flow rate 1.0 mL/min, UV detection at 254

nm), t_{major} = 13.0min, t_{minor} = 11.0min. **^{1}H NMR** (400 MHz, Chloroform-*d*) δ 7.54 (d, *J* = 7.7 Hz, 2H), 7.49 (d, *J* = 8.0 Hz, 2H), 7.34 (t, *J* = 7.6 Hz, 2H), 7.33 – 7.23 (m, 5H), 7.26 – 7.13 (m, 5H), 7.11 – 7.04 (m, 3H), 5.11 (d, *J* = 12.3 Hz, 1H), 5.05 (d, *J* = 12.3 Hz, 1H), 4.98 (d, *J* = 12.4 Hz, 1H), 4.93 (d, *J* = 12.4 Hz, 1H), 3.56 (d, *J* = 11.0 Hz, 1H), 3.32 (td, *J* = 12.3, 2.2 Hz, 1H), 2.54 (dd, *J* = 13.0, 6.4 Hz, 1H), 2.50 – 2.33 (m, 1H), 2.34 – 2.28 (m, 3H), 2.25 (dd, *J* = 13.2, 3.7 Hz, 1H), 2.12 (t, *J* = 12.8 Hz, 1H), 1.70 – 1.49 (m, 4H), 1.45 – 1.36 (m, 1H), 1.28 (t, *J* = 12.7 Hz, 1H), 1.26 – 1.15 (m, 1H). **^{13}C NMR** (101 MHz, Chloroform-*d*) δ 172.33, 172.02, 146.43, 144.20, 135.56, 135.51, 133.45, 128.68, 128.61, 128.52, 128.44, 128.31, 128.30, 128.25, 127.96, 127.55, 126.88, 125.90, 101.19, 81.56, 67.48, 67.29, 66.89, 57.54, 53.51, 44.69, 40.06, 35.76, 35.05, 31.68, 31.38, 21.22. **HRMS** (ESI): m/z Calcd. For $C_{40}H_{39}ClO_6Na$ ([M+Na]$^+$): 673.2327, found: 673.2320.

Benzyl (8a*S*, 10*R*, 11a*R*)-10-((benzylperoxy)-λ^2-methyl)-2-(3-chlorophenyl)-8-phenyldodecahydro-2, 8-epoxycyclopenta [*d*] oxecine-10-carboxylate (9i)

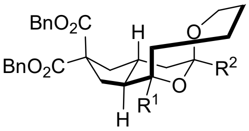

R^1=Ph,R^2=3-Cl-Ph

Light yellow oil, $[\alpha]_D^{25}$ = +8.98 (*c* 0.3, DCM), 28.8 mg, 96% yield, 85 % ee, dr > 20:1, determined by HPLC (Chiralcel column AD-H, hexane/i-PrOH = 95/5, flow rate 1.0 mL/min, UV detection at 254 nm), t_{major} = 11.7 min, t_{minor} = 10.6 min. **^{1}H NMR** (400 MHz, Chloroform-*d*) δ 7.65 (d, *J* = 7.4 Hz, 3H), 7.56 (d, *J* = 8.9 Hz, 1H), 7.51 – 7.38 (m, 3H), 7.37 (d, *J* = 2.3 Hz, 3H), 7.35 – 7.31 (m, 4H), 7.29 (d, *J* = 5.4 Hz, 3H), 7.29 – 7.04 (m, 2H), 5.22 (d, *J* = 12.3 Hz, 1H), 5.16 (d, *J* = 12.3 Hz, 1H), 5.09 (d, *J* = 12.6 Hz, 1H), 5.03 (d, *J* = 12.4 Hz, 1H), 3.70 (d, *J* = 11.8 Hz, 1H), 3.43 (td, *J* = 12.2, 2.1 Hz, 1H), 2.64 (dd, *J* = 13.0, 6.5 Hz, 1H), 2.57 – 2.43 (m, 1H), 2.40 (dd, *J* = 8.6, 4.7 Hz, 3H), 2.35 (dd, *J* = 13.2, 3.8 Hz, 1H), 2.22 (t, *J* = 12.8 Hz, 1H), 1.72 (dd, *J* = 21.5, 13.7 Hz, 4H), 1.56 – 1.46 (m, 1H), 1.39 (t, *J* = 12.7 Hz, 1H), 1.30 (q, *J* = 10.6, 9.6 Hz, 1H). **^{13}C NMR** (101 MHz, Chloroform-*d*) δ 172.32, 171.99, 147.77, 146.32, 135.54, 135.49, 134.30, 129.71, 128.67, 128.60, 128.44, 128.31 (2C), 128.24, 127.96, 127.78, 126.89, 126.40, 125.90, 124.27, 101.08, 81.64, 67.48, 67.29, 66.99, 57.51, 53.48, 44.62, 40.03, 35.74,

35.01, 31.64, 31.31, 21.22. **HRMS** (ESI): m/z calcd for $C_{40}H_{39}O_6ClNa$ ([M+Na]$^+$): 673.2327, found: 673.2325.

Benzyl (8a*S*, 10*R*, 11a*R*)-10-((benzylperoxy)-λ²-methyl)-2-(3-fluorophenyl)-8-phenyldodecahydro-2, 8-epoxycyclopenta [*d*] oxecine-10-carboxylate (9j)

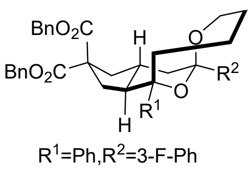

Light yellow oil, $[\alpha]_D^{25}$ = +19.38 (*c* 0.09, DCM), 24.0 mg, 80% yield, 88 % ee, dr > 20:1, determined by HPLC (Chiralcel column AD-H, hexane/i-PrOH = 95/5, flow rate 1.0 mL/min, UV detection at 254 nm), t_{major} = 13.1min, t_{minor} = 11.8min. **^{1}H NMR** (600 MHz, Chloroform-*d*) δ 7.64 (d, *J* = 7.6 Hz, 2H), 7.44 (t, *J* = 7.8 Hz, 3H), 7.42 – 7.34 (m, 4H), 7.35 – 7.30 (m, 2H), 7.30 – 7.18 (m, 5H), 7.19 – 7.14 (m, 2H), 7.03 (dt, *J* = 8.5, 4.2 Hz, 1H), 5.20 (d, *J* = 12.2 Hz, 1H), 5.15 (d, *J* = 12.2 Hz, 1H), 5.07 (d, *J* = 12.4 Hz, 1H), 5.02 (d, *J* = 12.4 Hz, 1H), 3.71 – 3.65 (m, 1H), 3.43 (td, *J* = 12.2, 2.0 Hz, 1H), 2.63 (dd, *J* = 13.1, 6.6 Hz, 1H), 2.47 (m, 1H), 2.43 – 2.35 (m, 3H), 2.34 (dd, *J* = 13.3, 3.8 Hz, 1H), 2.21 (t, *J* = 12.9 Hz, 1H), 1.78 – 1.69 (m, 2H), 1.68-1.64 (m, 2H), 1.53 – 1.46 (m, 1H), 1.39 (t, *J* = 12.7 Hz, 1H), 1.33 – 1.25 (m, 1H). **^{13}C NMR** (151 MHz, Chloroform-*d*) δ 172.33, 172.00, 163.02 (*J* = 246.1 Hz), 148.40 (*J* = 6.0 Hz), 146.37, 135.52 (*J* = 7.6 Hz), 129.88 (*J* = 7.6 Hz), 128.67, 128.60, 128.44, 128.31(2C), 128.25, 127.96, 126.88, 125.90, 121.65, 114.48 (*J* = 21.1 Hz), 113.30 (*J* = 22.7 Hz), 101.08, 81.60, 67.48, 67.29, 66.99, 57.52, 53.49, 44.56, 40.04, 35.73, 35.02, 31.66, 31.35, 21.21. **^{19}F NMR** (471 MHz, Chloroform-d) δ -113.07 (s, 1F). **HRMS** (ESI): m/z calcd for $C_{40}H_{39}O_6FNa$ ([M+Na]$^+$): 657.2623, found: 657.2627.

Benzyl (8a*S*, 10*R*, 11a*R*)-10-((benzylperoxy)-λ²-methyl)-2-methyl-8-phenyldodecahydro-2, 8-epoxycyclopenta [*d*] oxecine-10-carboxylate (9k)

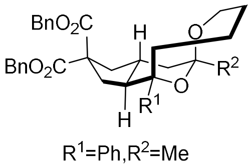

Light yellow oil, $[\alpha]_D^{25}$ = + 34.57 (*c* 0.10, DCM), 27.0 mg, 90% yield, 98% ee, dr > 20:1, determined by HPLC (Chiralcel column AD-H, hexane/i-PrOH = 95/5, flow rate 1.0 mL/min, UV detection at 254 nm), t_{major} = 8.5min, t_{minor} = 9.3min. **^{1}H NMR** (500 MHz, Chloroform-*d*) δ 7.49 – 7.43 (m, 2H), 7.36 – 7.29 (m, 5H), 7.29 – 7.20 (m, 6H), 7.13 (dd, *J* = 7.5, 2.0 Hz, 2H), 5.16 (d, *J* = 12.3 Hz, 1H), 5.10 (d, *J* = 12.3 Hz, 1H), 5.04 (d, *J* = 12.4 Hz,

1H), 4.98 (d, J = 12.4 Hz, 1H), 3.95 – 3.86 (m, 1H), 3.73 – 3.65 (m, 1H), 2.57 (dd, J = 13.0, 6.5 Hz, 1H), 2.34 – 2.19 (m, 4H), 2.18 – 2.08 (m, 2H), 1.71 (t, J = 12.5 Hz, 1H), 1.60 – 1.52 (m, 2H), 1.53 -1.43 (m, 4H), 1.35 (t, J = 12.6 Hz, 1H), 1.26 – 1.19 (m, 1H). **^{13}C NMR** (126 MHz, Chloroform-*d*) δ 172.42, 172.14, 146.52, 135.60, 135.56, 128.65, 128.59, 128.40, 128.26, 128.22, 128.07, 127.93, 126.60, 125.81, 98.86, 80.38, 67.41, 67.23, 64.22, 57.59, 53.72, 42.49, 40.24, 35.64, 34.49, 31.88, 31.05, 26.24, 21.41. **HRMS** (ESI): m/z calcd for $C_{35}H_{38}O_6Na$ ([M+Na]$^+$): 577.2561, found: 577.2561.

Benzyl (8a*S*, 10*R*, 11a*R*)-10-((benzylperoxy)-λ^2-methyl)-2-ethyl-8- phenyldo-decahydro-2, 8-epoxycyclopenta [*d*] oxecine-10-carboxylate (9l)

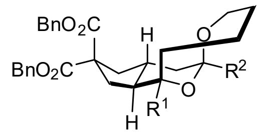

R^1=Ph,R^2=Et

Colorless oil, $[\alpha]_D^{25}$ = +27.74 (*c* 0.70, DCM), 18.9 mg, 63% yield, 99% ee, dr>20:1, determined by HPLC (Chiralcel column AD-H, hexane/i-PrOH = 98/2, flow rate 1.0 mL/min, UV detection at 254 nm), t_{major} = 18.3min, t_{minor} = 17.5min. **^{1}H NMR** (400 MHz, Chloroform-*d*) δ 7.51 (d, J = 7.6 Hz, 2H), 7.42 – 7.31 (m, 8H), 7.29 (t, J = 6.3 Hz, 3H), 7.25 – 7.13 (m, 2H), 5.21 (d, J = 12.4 Hz, 1H), 5.14 (d, J = 12.3 Hz, 1H), 5.10 (d, J = 12.4 Hz, 1H), 5.03 (d, J = 12.4 Hz, 1H), 3.90 (ddd, J = 13.2, 9.5, 4.1 Hz, 1H), 3.77 – 3.68 (m, 1H), 2.62 (dd, J = 13.0, 6.5 Hz, 1H), 2.34 – 2.28 (m, 4H), 2.18 (ddd, J = 13.0, 8.4, 4.4 Hz, 2H), 2.08 – 1.99(m, 1H), 1.85 – 1.67 (m, 2H), 1.69 – 1.56 (m, 3H), 1.57 – 1.51 (m, 1H), 1.38 – 1.23 (m, 2H), 1.04 – 0.92 (m, 3H). **^{13}C NMR** (101 MHz, Chloroform-*d*) δ 172.42, 172.16, 146.65, 135.62, 135.58, 128.66, 128.60, 128.40, 128.27, 128.23, 128.07, 127.94, 126.59, 125.83, 100.90, 80.30, 67.41, 67.23, 63.55, 57.63, 53.92, 40.38, 39.04, 35.69, 34.30, 31.94, 31.22, 31.16, 21.43, 8.50. **HRMS** (ESI): m/z Calcd. For $C_{36}H_{40}O_6Na$ ([M+Na]$^+$): 591.2717, found: 591.2714.

Benzyl (8a*S*, 10*R*, 11a*R*)-10-((benzylperoxy)-λ^2-methyl)-2-cyclopentyl-8-phenyldodecahydro-2, 8-epoxycyclopenta [*d*] oxecine-10-carboxylate (9m)

Colorless oil, $[\alpha]_D^{25}$ = +41.24 (*c* 0.15, DCM), 13.5 mg, 45% yield, 97% ee, dr>20:1, determined by HPLC (Chiralcel column AD-H, hexane/i-PrOH = 95/5, flow rate 1.0

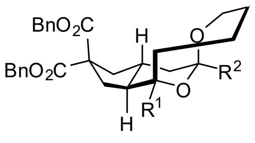

R^1=Ph,R^2=Cyclopentyl

mL/min, UV detection at 254 nm), t_{major} = 8.0 min, t_{minor} = 7.3 min. **^{1}H NMR** (600 MHz, Acetone-d_6) δ 7.54 – 7.48 (m, 2H), 7.38 – 7.30 (m, 7H), 7.31 – 7.26 (m, 3H), 7.26 – 7.21 (m, 1H), 7.23 – 7.18 (m, 2H), 5.19 (d, J = 12.3 Hz, 1H), 5.14 (d, J = 12.4 Hz, 1H), 5.04 (d, J = 2.8 Hz, 2H), 4.02 – 3.94 (m, 1H), 3.60 – 3.54 (m, 1H), 2.62 (dd, J = 12.8, 6.5 Hz, 1H), 2.37 – 2.16 (m, 4H), 2.05 (d, J = 13.1 Hz, 1H), 1.93 (dd, J = 12.9, 4.2 Hz, 1H), 1.91 – 1.82 (m, 1H), 1.69 (dd, J = 12.9, 12.0 Hz, 1H), 1.65 – 1.53 (m, 7H), 1.55 – 1.48 (m, 1H), 1.48 – 1.38 (m, 2H), 1.38 – 1.27 (m, 3H), 1.20 – 1.11 (m, 1H). **^{13}C NMR** (151 MHz, Acetone-d_6) δ 172.79, 172.40, 147.77, 136.95, 136.87, 129.33, 129.27, 129.00, 128.92, 128.88, 128.71, 128.68, 127.20, 126.55, 103.57, 80.79, 67.73, 67.54, 63.31, 58.08, 54.89, 45.64, 41.12, 36.24, 34.91, 34.35, 32.59, 31.51, 29.28, 27.58, 26.94, 26.78, 22.21. **HRMS** (ESI): m/z Calcd. For $C_{39}H_{44}O_6Na$ ($[M+Na]^+$): 631.3030, found: 631.3032.

Benzyl (8a*S*, 10*R*, 11a*R*)-10-((benzylperoxy)-λ^2-methyl)-2-cyclohexyl-8-phenyldodecahydro-2, 8-epoxycyclopenta [*d*] oxecine-10-carboxylate (9n)

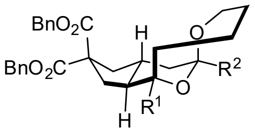

R^1=Ph,R^2=Cyclohexyl

Colorless oil, $[\alpha]_D^{25}$ = +24.08 (*c* 0.25, DCM), 15.0 mg, 50% yield, 98% ee, dr>20:1, determined by HPLC (Chiralcel column AD-H, hexane/i-PrOH = 90/10, flow rate 1.0 mL/min, UV detection at 254 nm), t_{major} = 5.6 min, t_{minor} = 4.8 min. **^{1}H NMR** (600 MHz, Acetone-d_6) δ 7.55 – 7.50 (m, 2H), 7.39 – 7.35 (m, 1H), 7.35 – 7.30 (m, 6H), 7.30 – 7.25 (m, 3H), 7.28 – 7.18 (m, 3H), 5.19 (d, J = 12.4 Hz, 1H), 5.14 (d, J = 12.4 Hz, 1H), 5.08 – 5.00 (m, 2H), 3.90 (td, J = 11.6, 2.1 Hz, 1H), 3.61 – 3.54 (m, 1H), 2.61 (dd, J = 12.9, 6.5 Hz, 1H), 2.37 – 2.19 (m, 4H), 2.05 (d, J = 13.3 Hz, 1H), 1.89 – 1.74 (m, 3H), 1.71 – 1.63 (m, 4H), 1.63 – 1.49 (m, 3H), 1.49 – 1.34 (m, 3H), 1.33 – 1.26 (m, 2H), 1.25 – 1.07 (m, 3H), 1.07 – 0.97 (m, 1H). **^{13}C NMR** (151 MHz, Acetone-d_6) δ 172.77, 172.41, 147.80, 136.95, 129.33, 129.27, 128.99, 128.92, 128.88, 128.72, 128.68, 126.61, 103.15, 80.92, 67.73, 67.54, 63.02, 58.08, 54.89, 44.87, 41.09, 36.34, 35.23, 34.85, 32.46, 31.77, 27.84 (2C), 27.53, 27.43, 27.40, 22.20. **HRMS** (ESI): m/z Calcd. For $C_{40}H_{46}O_6Na$ ($[M+Na]^+$): 645.3187, found: 645.3188.

2.5 Synthesis and characterization of substrates **10a-p**

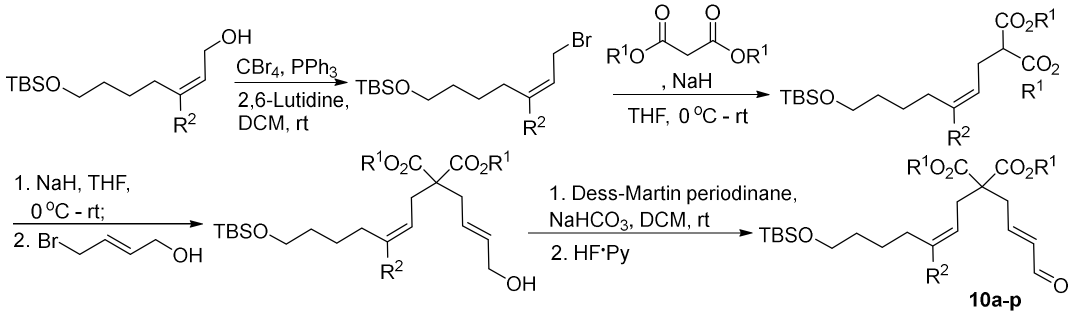

10a-p

Dibenzyl-2-((*E*)-7-hydroxy-3-phenylhept-2-en-1-yl)-2-((*E*)-4-oxobut-2-en-1-yl) malonate (10a)

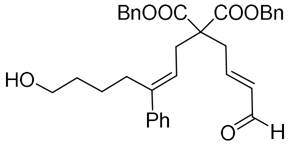

Colorless oil. **^{1}H NMR** (400 MHz, Acetone-d_6) δ 9.43 (d, J = 7.8 Hz, 1H), 7.34 (s, 9H), 7.30 – 7.19 (m, 6H), 6.83 (dt, J = 15.2, 7.5 Hz, 1H), 6.12 (dd, J = 15.5, 7.8 Hz, 1H), 5.49 (t, J = 7.4 Hz, 1H), 5.20 (s, 4H), 3.47 (q, J = 6.0 Hz, 2H), 3.03 – 2.99 (m, 2H), 2.92 (d, J = 7.4 Hz, 2H), 2.51 (t, J = 7.8 Hz, 2H), 1.50 – 1.44 (m, 2H), 1.39 – 1.33 (m, 2H). **^{13}C NMR** (101 MHz, Acetone-d_6) δ 193.87, 170.66, 152.36, 145.30, 143.56, 136.79, 136.56, 129.38, 129.20, 129.17, 129.08, 127.80, 127.25, 121.88, 67.96, 62.19, 58.30, 36.73, 33.48, 32.91, 30.36, 25.82. **HRMS** (ESI): m/z Calcd. For $C_{36}H_{36}O_6Na$ ([M+Na]$^+$): 563.2404, found: 563.2403.

Bis(4-fluorobenzyl)-2-((*E*)-7-hydroxy-3-phenylhept-2-en-1-yl)-2-((*E*)-4-oxobut-2-en-1-yl)malonate (10b)

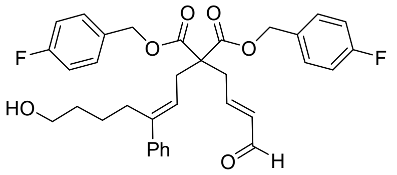

Colorless oil. **^{1}H NMR** (400 MHz, Acetone-d_6) δ 9.44 (d, J = 7.8 Hz, 1H), 7.41 – 7.35 (m, 4H), 7.30 – 7.23 (m, 5H), 7.09 (td, J = 8.9, 2.7 Hz, 4H), 6.84 (dt, J = 15.3, 7.5 Hz, 1H), 6.10 (dd, J = 15.5, 7.8 Hz, 1H), 5.46 (t, J = 7.4 Hz, 1H), 5.19 (s, 4H), 3.47 (d, J = 5.7 Hz, 2H), 3.00 (d, J = 7.5 Hz, 2H), 2.91 (d, J = 7.4 Hz, 2H), 2.51 (t, J = 7.8 Hz, 2H), 1.50 – 1.45 (m, 2H), 1.36 (q, J = 6.7, 5.3 Hz, 2H). **^{13}C NMR** (101 MHz, Acetone-d_6) δ 193.85, 170.61, 164.51 (J = 245.4 Hz), 152.25, 145.31, 143.51, 136.80, 132.73 (J = 3.2 Hz), 131.55 (J = 8.1 Hz), 129.07, 127.84, 127.22, 121.83, 116.11 (J = 21.2 Hz), 67.22, 62.19, 58.26, 36.73, 33.46, 32.91, 30.34, 25.83. **HRMS** (ESI): m/z Calcd. For $C_{34}H_{34}F_2O_6Na$ ([M+Na]$^+$): 599.2216, found:599.2217.

Bis(4-chlorobenzyl)-2-((*E*)-7-hydroxy-3-phenylhept-2-en-1-yl)-2-((*E*)-4-oxobut-2-en-1-yl)malonate (10c)

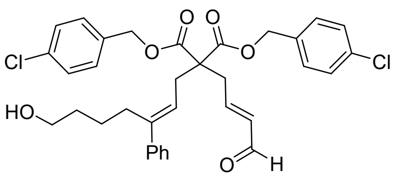

Colorless oil. **^{1}H NMR** (400 MHz, Acetone-d_6) δ 9.46 (d, J = 7.8 Hz, 1H), 7.44 – 7.38 (m, 1H), 7.35 (s, 6H), 7.33 – 7.27 (m, 3H), 7.25 (d, J = 7.1 Hz, 3H), 6.92 – 6.82 (m, 1H), 6.21 – 6.03 (m, 1H), 5.47 (t, J = 7.3 Hz, 1H), 5.20 (s, 4H), 3.47 (q, J = 5.9 Hz, 2H), 3.02 (dd, J = 7.5, 1.4 Hz, 2H), 2.92 (d, J = 7.5 Hz, 2H), 2.51 (t, J = 7.8 Hz, 2H), 1.47 (dd, J = 8.5, 6.3 Hz, 2H), 1.35 (dd, J = 10.0, 6.0 Hz, 2H). **^{13}C NMR** (101 MHz, Acetone-d6) δ 193.86, 170.58, 152.20, 145.36, 143.51, 136.86, 135.46, 134.60, 130.98, 129.46, 129.10, 127.86, 127.23, 121.80, 67.14, 62.19, 58.31, 36.75, 33.46, 32.93, 30.34, 25.82. **HRMS** (ESI): m/z Calcd. For $C_{34}H_{34}Cl_2O_6Na$ ([M+Na]$^+$): 631.1625, found: 631.1621.

Bis(4-bromobenzyl)-2-((*E*)-7-hydroxy-3-phenylhept-2-en-1-yl)-2-((*E*)-4-oxobut-2-en-1-yl)malonate (10d)

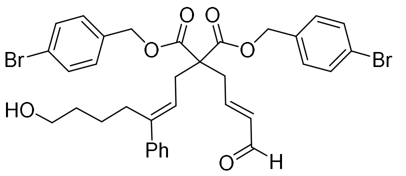

Colorless oil. **^{1}H NMR** (400 MHz, Acetone-d6) δ 9.47 (d, J = 7.8 Hz, 1H), 7.53 – 7.46 (m, 4H), 7.31 – 7.24 (m, 9H), 6.85 (dd, J = 15.4, 7.6 Hz, 1H), 6.19 – 6.08 (m, 1H), 5.47 (t, J = 7.4 Hz, 1H), 5.18 (s, 4H), 3.47 (d, J = 5.8 Hz, 2H), 3.04 – 3.00 (m, 2H), 2.92 (d, J = 7.4 Hz, 2H), 2.51 (t, J = 7.7 Hz, 2H), 1.49 – 1.45 (m, 2H), 1.38 – 1.33 (m, 2H). **^{13}C NMR** (101 MHz, Acetone-d6) δ 193.85, 170.57, 152.19, 145.36, 143.52, 136.87, 135.92, 132.48, 131.25, 129.12, 127.86, 127.24, 122.79, 121.80, 67.18, 62.19, 36.75, 33.47, 32.93, 30.35, 29.26, 25.82. **HRMS** (ESI): m/z Calcd. For $C_{34}H_{34}Br_2O_6Na$ ([M+Na]$^+$): 719.0614, found:719.0618.

Bis(4-methylbenzyl)-2-((*E*)-7-hydroxy-3-phenylhept-2-en-1-yl)-2-((*E*)-4-oxobut-2-en-1-yl)malonate (10e)

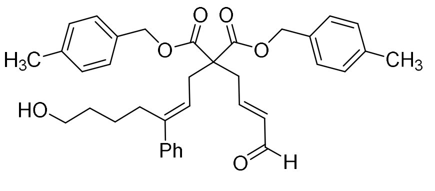

Colorless oil. ^{1}H NMR (400 MHz, Acetone-d_6) δ 9.42 (d, J = 7.8 Hz, 1H), 7.40 – 7.20 (m, 9H), 7.15 (d, J = 7.9 Hz, 4H), 6.81 (dt, J = 15.2, 7.5 Hz, 1H), 6.12 –6.05 (m, 1H), 5.47 (t, J = 7.4 Hz, 1H), 5.14 (s, 4H), 3.47 (q, J = 6.1 Hz, 2H), 2.98

(dd, J = 7.5, 1.4 Hz, 2H), 2.90 (d, J = 7.4 Hz, 2H), 2.50 (t, J = 7.8 Hz, 2H), 2.32 (s, 6H), 1.51 – 1.44 (m, 2H), 1.39 – 1.33 (m, 2H). **^{13}C NMR** (101 MHz, Acetone-d_6) δ 193.81, 170.66, 152.37, 145.22, 143.56, 138.90, 136.73, 133.56, 129.97, 129.41, 129.05, 127.77, 127.24, 121.90, 67.87, 62.20, 58.23， 36.70, 33.50, 32.87, 30.36, 25.82, 21.21. **HRMS** (ESI): m/z Calcd. For $C_{34}H_{40}O_6Na$ ([M+Na]$^+$): 591.2717, found: 591.2717.

Bis(4-methoxybenzyl)-2-((*E*)-7-hydroxy-3-phenylhept-2-en-1-yl)-2-((*E*)-4-oxobut-2-en-1-yl)malonate (10f)

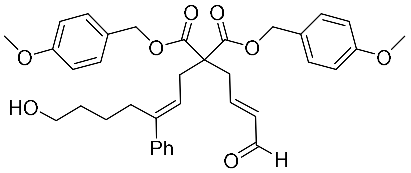

Colorless oil. **^{1}H NMR** (500 MHz, Acetone-d6) δ 9.42 (d, J = 7.8 Hz, 1H), 7.30 – 7.23 (m, 9H), 6.90 – 6.87 (m, 4H), 5.11 (s, 1H), 6.10 –6.04 (m, 1H), 5.44 (t, J = 7.4 Hz, 1H), 5.13 – 5.08 (m, 4H), 3.79 (s, 6H), 3.49 – 3.44 (m, 2H), 2.97 – 2.93 (m, 2H), 2.86 (d, J = 7.3 Hz, 2H), 2.53 – 2.45 (m, 2H), 1.49 – 1.44 (m, 2H), 1.36 –1.32 (m, 2H). **^{13}C NMR** (126 MHz, Acetone-d6) δ 193.89, 170.72, 160.83, 152.48, 145.18, 143.57, 136.74, 131.16, 129.08, 128.52, 127.79, 127.26, 121.96, 114.72, 67.77, 62.21, 58.20, 55.55, 36.71, 33.51, 32.86, 30.34, 25.84. **HRMS** (ESI): m/z Calcd. For $C_{36}H_{40}O_8Na$ ([M+Na]$^+$): 623.2615, found: 623.2614.

Bis(4-(trifluoromethyl)benzyl)-2-((*E*)-7-hydroxy-3-phenylhept-2-en-1-yl)-2-((*E*)-4-oxobut-2-en-1-yl)malonate (10g)

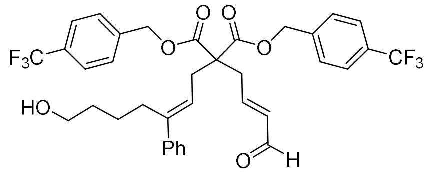

Colorless oil. **^{1}H NMR** (400 MHz, Acetone-d_6) δ 9.48 (d, J = 7.8 Hz, 1H), 7.68 – 7.61 (m, 4H), 7.56 (d, J = 8.0 Hz, 4H), 7.28 (d, J = 3.3 Hz, 5H), 6.91 (dt, J = 15.2, 7.5 Hz, 1H), 6.16 (dd, J = 15.5, 7.8 Hz, 1H), 5.53 (t, J = 7.4 Hz, 1H), 5.34 (s, 4H), 3.47 (q, J = 6.2 Hz, 2H), 3.08 (d, J = 7.5 Hz, 2H), 2.97 (d, J = 7.4 Hz, 2H), 2.53 (t, J = 7.8 Hz, 2H), 1.47 (dd, J = 8.4, 6.3 Hz, 2H), 1.37 (td, J = 8.8, 8.4, 4.1 Hz, 2H). **^{13}C NMR** (101 MHz, Acetone-d6) δ 193.86, 170.59, 152.08, 145.50, 143.53, 141.10, 136.96, 130.61 (J = 33.3 Hz), 129.44 (2C), 129.10, 127.89, 127.24, 126.24, 121.77, 67.09, 62.18, 58.44, 36.81, 33.45, 33.00, 30.38, 25.84. **HRMS** (ESI): m/z Calcd. For $C_{36}H_{34}F_6O_6H$ ([M-H]$^-$): 675.2187, found: 675.2187.

Bis(4-fluorophenyl)-2-((*E*)-7-hydroxy-3-phenylhept-2-en-1-yl)-2-((*E*)-4-oxobut-2-

en-1-yl)malonate (10h)

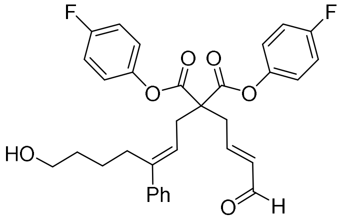

Colorless oil. ^{1}H NMR (400 MHz, Acetone-d_6) δ 9.65 (d, J = 7.8 Hz, 1H), 7.43 (d, J = 8.3 Hz, 2H), 7.35 (t, J = 7.5 Hz, 2H), 7.30 – 7.22 (m, 9H), 7.17 (dt, J = 15.2, 7.4 Hz, 1H), 6.40 (dd, J = 15.5, 7.8 Hz, 1H), 5.79 (t, J = 7.4 Hz, 1H), 3.54 – 3.45 (m, 2H), 3.33 (d, J = 7.5 Hz, 2H), 3.21 (d, J = 7.4 Hz, 2H), 2.69 (t, J = 7.6 Hz, 2H), 1.58 – 1.50 (m, 2H), 1.49 – 1.44 (m, 2H). **^{13}C NMR** (101 MHz, Acetone-d6) δ 194.13, 169.58, 161.41 (J = 243.4 Hz), 151.54, 147.47 (J = 3.0 Hz), 146.16, 143.55, 137.42, 129.27, 128.08, 127.33, 124.01 (J = 9.1 Hz), 121.36, 117.14 (J = 24.2 Hz), 62.19, 58.56, 36.80, 33.45, 33.05, 30.57, 25.96. **HRMS** (ESI): m/z Calcd. For $C_{32}H_{30}F_2O_6Na$ ([M+Na]$^+$): 571.1903, found: 571.1905.

Bis(4-chlorophenyl)-2-((*E*)-7-hydroxy-3-phenylhept-2-en-1-yl)-2-((*E*)-4-oxobut-2-en-1-yl)malonate (10i)

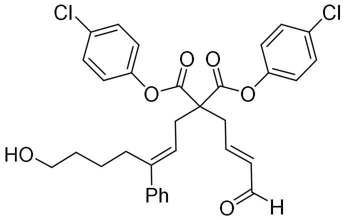

Colorless oil. ^{1}H NMR (400 MHz, Acetone-d_6) δ 9.64 (d, J = 7.7 Hz, 1H), 7.54 – 7.47 (m, 4H), 7.42 (d, J = 7.0 Hz, 2H), 7.34 (t, J = 7.5 Hz, 2H), 7.30 – 7.21 (m, 5H), 7.16 (dt, J = 15.2, 7.4 Hz, 1H), 6.39 (dd, J = 15.5, 7.8 Hz, 1H), 5.78 (t, J = 7.4 Hz, 1H), 3.48 (q, J = 6.0 Hz, 2H), 3.35 – 3.31 (m, 2H), 3.21 (d, J = 7.4 Hz, 2H), 2.68 (t, J = 7.6 Hz, 2H), 1.57 – 1.49 (m, 2H), 1.46 –1.40 (m, 2H). **^{13}C NMR** (101 MHz, Acetone-d6) δ 193.26, 168.45, 150.57, 149.25, 145.38, 142.65, 136.59, 131.36, 129.74, 128.42, 127.25, 126.48, 123.18, 120.41, 61.36, 57.81, 35.94, 32.58, 32.21, 29.71, 25.10. **HRMS** (ESI): m/z Calcd. For $C_{32}H_{30}Cl_2O_6Na$ ([M+Na]$^+$): 603.1312, found: 603.1318.

Bis(4-bromophenyl)-2-((*E*)-7-hydroxy-3-phenylhept-2-en-1-yl)-2-((*E*)-4-oxobut-2-en-1-yl)malonate (10j)

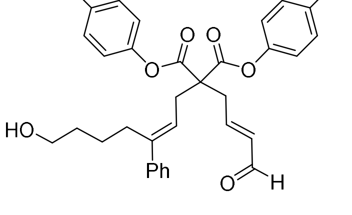

Colorless oil. ^{1}H NMR (400 MHz, Acetone-d_6) δ 9.64 (d, J = 7.8 Hz, 1H), 7.70 – 7.59 (m, 4H), 7.42 (d, J = 7.2 Hz, 2H), 7.34 (t, J = 7.5 Hz, 2H), 7.31 – 7.24 (m, 1H), 7.23 – 7.10 (m, 5H), 6.39 (dd, J = 15.5, 7.8 Hz, 1H), 5.78 (t, J =

7.4 Hz, 1H), 3.49 (q, J = 5.9 Hz, 2H), 3.36 – 3.30 (m, 2H), 3.21 (d, J = 7.4 Hz, 2H), 2.68 (t, J = 7.6 Hz, 2H), 1.52 (dt, J = 12.6, 5.8 Hz, 2H), 1.48 – 1.41 (m, 2H), **^{13}C NMR** (101 MHz, Acetone) δ 194.08, 169.21, 151.38, 150.62, 146.22, 143.50, 137.43, 133.61, 129.25, 128.07, 127.32, 124.43, 121.24, 119.90, 62.17, 58.66, 36.75, 33.42, 33.03, 30.54, 25.94. **HRMS** (ESI): m/z Calcd. For $C_{32}H_{30}O_6Br_2Na$ ([M+Na]$^+$): 691.0301, found: 691.0307.

Dibenzyl-2-((*E*)-3-(3-chlorophenyl)-7-hydroxyhept-2-en-1-yl)-2-((*E*)-4-oxobut-2-en-1-yl) malonate (10k)

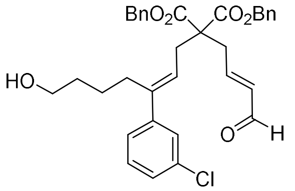

Colorless oil. **^{1}H NMR** (400 MHz, Acetone-d_6) δ 9.43 (d, J = 7.8 Hz, 1H), 7.40 – 7.22 (m, 13H), 7.22 – 7.17 (m, 1H), 6.83 (dt, J = 15.2, 7.5 Hz, 1H), 6.16 – 6.09 (m, 1H), 5.54 (t, J = 7.4 Hz, 1H), 5.20 (s, 4H), 3.47 (q, J = 5.9 Hz, 2H), 3.02 (dd, J = 7.5, 1.4 Hz, 2H), 2.92 (d, J = 7.4 Hz, 2H), 2.50 (t, J = 7.8 Hz, 2H), 1.47 (dd, J = 8.7, 6.1 Hz, 2H), 1.34 (dt, J = 15.0, 7.2 Hz, 2H). **^{13}C NMR** (101 MHz, Acetone) δ 193.03, 169.75, 151.45, 144.94, 143.25, 135.99, 135.68, 133.71, 129.90, 128.55, 128.39, 128.36, 126.90, 126.34, 125.04, 122.54, 67.16, 61.28, 57.36, 35.87, 32.51, 32.06, 29.37, 24.86. **HRMS** (ESI): m/z Calcd. For $C_{34}H_{35}ClO_6Na$ ([M+Na]$^+$): 597.2014, found: 597.2015.

Dibenzyl-2-((*E*)-3-(4-chlorophenyl)-7-hydroxyhept-2-en-1-yl)-2-((*E*)-4-oxobut-2-en-1-yl) malonate (10l)

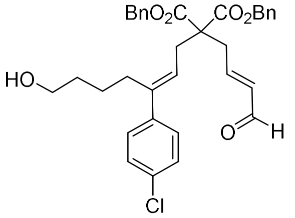

Colorless oil. **^{1}H NMR** (400 MHz, Acetone-d_6) δ 9.42 (d, J = 7.8 Hz, 1H), 7.36 – 7.23 (m, 14H), 6.81 (dt, J = 15.2, 7.5 Hz, 1H), 6.15 – 6.07 (m, 1H), 5.50 (t, J = 7.4 Hz, 1H), 5.19 (s, 4H), 3.46 (q, J = 6.0 Hz, 2H), 3.02 – 2.98 (m, 2H), 2.90 (d, J = 7.4 Hz, 2H), 2.48 (t, J = 7.8 Hz, 2H), 1.47 – 1.41 (m, 2H), 1.32 (dd, J = 10.5, 5.5 Hz, 2H). **^{13}C NMR** (101 MHz, Acetone-d_6) δ 193.87, 170.61, 152.29, 144.05, 142.26, 136.82, 136.54, 133.06, 129.40, 129.25, 129.20, 129.09, 128.92, 122.76, 67.98, 62.12, 58.21, 36.75, 33.37, 32.91, 30.16, 25.71. **HRMS** (ESI): Calcd. For $C_{34}H_{35}ClO_6Na$ ([M+Na]$^+$): 597.2014, found:597.2012.

Dibenzyl-2-((*E*)-7-hydroxy-3-(m-tolyl)hept-2-en-1-yl)-2-((*E*)-4-oxobut-2-en-1-yl)malonate (10m)

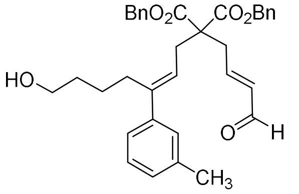

Colorless oil. **^{1}H NMR** (400 MHz, Acetone-*d*$_6$) δ 9.43 (d, *J* = 7.8 Hz, 1H), 7.34 (br, 9H), 7.22 – 7.04 (m, 5H), 6.83 (dt, *J* = 15.2, 7.4 Hz, 1H), 6.12 (ddd, *J* = 15.6, 7.7, 1.5 Hz, 1H), 5.48 (t, *J* = 7.3 Hz, 1H), 5.20 (br, 4H), 3.47 (d, *J* = 6.0 Hz, 2H), 3.01 (dd, *J* = 7.5, 1.4 Hz, 2H), 2.92 (d, *J* = 7.4 Hz, 2H), 2.50 (t, *J* = 7.8 Hz, 2H), 2.30 (s, 3H), 1.49 – 1.44 (m, 2H), 1.35 (dt, *J* = 8.8, 3.3 Hz, 2H). **^{13}C NMR** (101 MHz, Acetone-*d*$_6$) δ 193.87, 170.66, 152.38, 145.48, 143.62, 138.37, 136.77, 136.55, 129.38, 129.16 (2C), 129.11, 128.97, 128.51, 127.90, 124.45, 121.59, 67.93, 62.20, 58.28, 36.69, 33.47, 30.42, 32.88, 25.81, 21.52. **HRMS** (ESI): m/z Calcd. For $C_{35}H_{38}O_6Na$ ([M+Na]$^+$): 577.2561, found: 577.2565.

Dibenzyl-2-((*E*)-7-hydroxy-3-(p-tolyl)hept-2-en-1-yl)-2-((*E*)-4-oxobut-2-en-1-yl)malonate (10n)

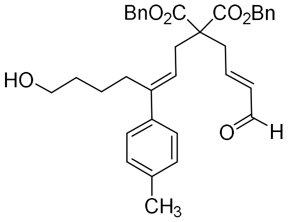

Colorless oil. **^{1}H NMR** (400 MHz, Acetone-*d*$_6$) δ 9.43 (d, *J* = 7.8 Hz, 1H), 7.34 (s, 9H), 7.19 – 7.08 (m, 5H), 6.83 (dt, *J* = 15.2, 7.4 Hz, 1H), 6.17 – 6.05 (m, 1H), 5.46 (t, *J* = 7.4 Hz, 1H), 5.19 (s, 4H), 3.47 (d, *J* = 5.9 Hz, 2H), 3.00 (dd, *J* = 7.6, 1.3 Hz, 2H), 2.91 (d, *J* = 7.4 Hz, 2H), 2.49 (t, *J* = 7.8 Hz, 2H), 2.30 (s, 3H), 1.49 – 1.44 (m, 2H), 1.37 – 1.32 (m, 2H). **^{13}C NMR** (101 MHz, Acetone-*d*$_6$) δ 193.86, 170.67, 152.39, 145.11, 140.61, 137.13, 136.77, 136.56, 129.70, 129.38, 129.19, 129.17, 127.13, 121.03, 67.93, 62.20, 58.30, 36.69, 33.49, 32.86, 30.29, 25.82, 21.03. **HRMS** (ESI): m/z Calcd. For $C_{35}H_{38}O_6Na$ ([M+Na]$^+$): 577.2561, found:577.2564.

Dibenzyl-2-((*E*)-7-hydroxy-3-(3-methoxyphenyl)hept-2-en-1-yl)-2-((*E*)-4-oxobut-2-en-1-yl)malonate (10o)

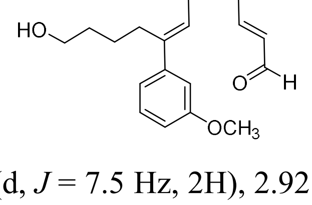

Colorless oil. **^{1}H NMR** (400 MHz, Acetone-*d*$_6$) δ 9.43 (d, *J* = 7.8 Hz, 1H), 7.34 (s, 9H), 7.21 (t, *J* = 8.1 Hz, 1H), 6.92 – 6.64 (m, 5H), 6.12 (dd, *J* = 15.6, 7.8 Hz, 1H), 5.52 (t, *J* = 7.4 Hz, 1H), 5.20 (s, 4H), 3.78 (s, 3H), 3.51 – 3.45 (m, 2H), 3.01 (d, *J* = 7.5 Hz, 2H), 2.92 (d, *J* = 7.4 Hz, 2H), 2.50 (t, *J* = 7.8 Hz, 2H), 1.47 (q, *J* = 6.9

Hz, 2H), 1.35 (dd, J = 10.7, 5.3 Hz, 2H). **^{13}C NMR** (101 MHz, Acetone-d_6) δ 193.87, 170.64, 160.61, 152.36, 145.27, 145.11, 136.78, 136.53, 130.05, 129.37, 129.16 (2C), 121.92, 119.69, 113.25, 112.88, 67.94, 62.18, 58.27, 55.40, 36.68, 33.46, 32.84, 30.4, 25.79. **HRMS** (ESI): m/z Calcd. For $C_{35}H_{38}O_7Na$ ([M+Na]$^+$): 593.2510, found: 593.2511.

Dibenzyl-2-((*E*)-3-(4-bromophenyl)-7-hydroxyhept-2-en-1-yl)-2-((*E*)-4-oxobut-2-en-1-yl) malonate (10p)

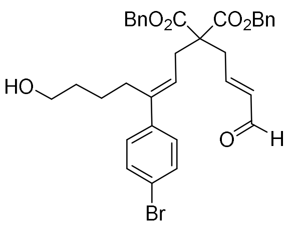

Colorless oil. **^{1}H NMR** (400 MHz, Acetone-d_6) δ 9.43 (d, J = 7.8 Hz, 1H), 7.46 (d, J = 8.5 Hz, 1H), 7.34 (s, 11H), 7.30 – 7.08 (m, 2H), 6.82 (dt, J = 15.2, 7.5 Hz, 1H), 6.12 (dd, J = 15.5, 7.8 Hz, 1H), 5.52 (t, J = 7.4 Hz, 1H), 5.20 (s, 4H), 3.47 (q, J = 6.1 Hz, 2H), 3.04 – 2.97 (m, 2H), 2.91 (d, J = 7.4 Hz, 2H), 2.49 (t, J = 7.8 Hz, 2H), 1.50 – 1.42 (m, 2H), 1.34 (dd, J = 10.4, 5.4 Hz, 2H). **^{13}C NMR** (101 MHz, Acetone-d_6) δ 193.82, 170.56, 152.25, 144.06, 142.64, 136.78, 136.48, 132.04, 129.36, 129.23, 129.21, 129.17, 122.78, 121.17, 67.96, 62.10, 58.17, 36.73, 33.34, 32.91, 30.08, 25.68. **HRMS** (ESI): m/z Calcd. For $C_{34}H_{35}BrO_6Na$ ([M+Na]$^+$): 641.1508, found: 641.1509.

2.6 General procedure for IEDHDA-keto acetalization reactions with **10a-p**

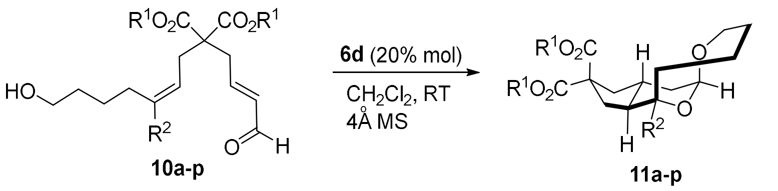

Substrate **10a-p** (30 mg, 1.0 eq.) was dissolved in DCM (1.0 mL), then 4Å molecular sieves (60 mg) followed by catalyst **6d** (0.2 eq.) were added. The reaction was stirred at 30 °C and monitored by TLC (generally for 60 hours). When the reaction was completed, the mixture was directly purified by silica gel column to afford products **11a-p**.

Dibenzyl(8a*S*,11a*R*)-8-phenyldecahydro-2,8-epoxycyclopenta[*d*]oxecine-10,10(*4H*) –dicarboxylate (11a)

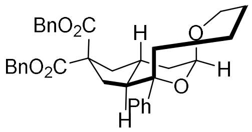

White solid, $[\alpha]_D^{25}$ = + 41.83 (*c* 1.00, DCM), 95% yield, 14.3 mg, 94%ee, determined by HPLC (Chiralcel column AD-H, hexane/i-PrOH = 90/10, flow rate 1.0 mL/min, UV detection at 254 nm), t_{major} = 7.3 min ,t_{minor} = 7.8 min. **^{1}H NMR** (800 MHz, Chloroform-*d*) δ 7.47 – 7.44 (m, 2H), 7.34 – 7.29 (m, 5H), 7.27 – 7.22 (m, 6H), 7.12 (dt, *J* = 6.7, 1.6 Hz, 2H), 5.15 (d, *J* = 12.3 Hz, 1H), 5.13 (d, *J* = 3.6 Hz, 1H), 5.09 (d, *J* = 12.2 Hz, 1H), 5.03 (d, *J* = 12.5 Hz, 1H), 4.98 (d, *J* = 12.4 Hz, 1H), 4.00 – 3.96 (m, 1H), 3.72 (td, *J* = 12.2, 2.1 Hz, 1H), 2.58 (dd, *J* = 13.1, 6.5 Hz, 1H), 2.32 – 2.26 (m, 2H), 2.26 – 2.16 (m, 2H), 2.11 (t, *J* = 12.9 Hz, 1H), 2.07 (ddd, *J* = 13.0, 3.7, 1.0 Hz, 1H), 1.71 (t, *J* = 12.6 Hz, 1H), 1.66 – 1.59 (m, 2H), 1.56 – 1.50 (m, 2H), 1.47 (td, *J* = 12.7, 3.8 Hz, 1H), 1.30 – 1.27 (m, 1H). **^{13}C NMR** (201 MHz, Chloroform-*d*) δ 172.40, 172.12, 146.43, 135.61, 135.56, 128.67, 128.61, 128.42, 128.29, 128.24, 128.15, 127.95, 126.68, 125.73, 97.66, 80.09, 69.45, 67.43, 67.26, 57.23, 53.84, 40.24, 36.13, 35.67, 33.31, 31.71, 31.41, 21.22. **HRMS** (ESI): m/z Calcd.For $C_{34}H_{36}O6Na$ ($[M+Na]^+$): 563.2404, found: 563.2403.

Bis(4-fluorobenzyl)(8a*S*,11a*R*)-8-phenyldecahydro-2,8-epoxycyclopenta[*d*] oxe-cine -10,10(4*H*)-dicarboxylate (11b)

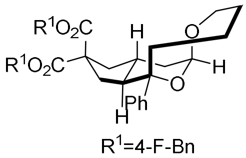

White solid, $[\alpha]_D^{25}$ = +35.31 (*c* 1.16, DCM), 24.6 mg, 82% yield, 95% ee, determined by HPLC (Chiralcel column AD-H, hexane/i-PrOH = 90/10, flow rate 1.0 mL/min, UV detection at 254 nm), t_{major} = 8.4 min, t_{minor} = 9.3 min. **^{1}H NMR** (400 MHz, Chloroform-*d*) δ 7.51 (d, *J* = 7.7 Hz, 2H), 7.38 (t, *J* = 7.6 Hz, 2H), 7.34 – 7.23 (m, 3H), 7.14 (dd, *J* = 8.5, 5.4 Hz, 2H), 7.05 (t, *J* = 8.7 Hz, 2H), 6.97 (t, *J* = 8.7 Hz, 2H), 5.19 (d, *J* = 3.5 Hz, 1H), 5.13 (q, *J* = 12.2 Hz, 2H), 5.07 – 4.95 (m, 2H), 4.09 – 4.00 (m, 1H), 3.77 (td, *J* = 11.9, 2.2 Hz, 1H), 2.59 (dd, *J* = 13.0, 6.4 Hz, 1H), 2.40 – 2.19 (m, 4H), 2.21 – 2.08 (m, 2H), 1.81 – 1.46 (m, 5H), 1.41 – 1.27 (m, 2H). **^{13}C NMR** (101 MHz, Chloroform-*d*) δ 172.25, 171.97, 162.8 (*J* = 248.5 Hz), 162.7 (*J* = 248.5 Hz), 146.38, 131.39 (*J* = 4.0 Hz), 131.32 (*J* = 3.0 Hz), 130.30, (*J* = 8.1 Hz), 130.00 (*J* = 8.1 Hz), 128.15, 126.70, 125.70, 115.61 (*J* = 22.2 Hz), 115.55 (*J* = 21.21 Hz), 97.64, 80.03, 69.47, 66.71, 66.53, 57.17, 53.82, 40.19, 36.12, 35.66, 33.31, 31.70, 31.40, 21.21. **^{19}F NMR** (471 MHz, Chloroform-d) δ -113.27

(s, 1F), -113.41 (s, 1F). **HRMS** (ESI): m/z Calcd. For $C_{34}H_{34}O_6F_2Na$ ($[M+Na]^+$): 599.2216, found: 599.2212.

Bis(4-chlorobenzyl)(8aS,11a*R*)-8-phenyldecahydro-2,8-epoxycyclopenta[*d*] oxecine-10,10(4*H*)-dicarboxylate (11c)

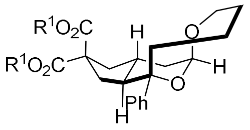

R¹=4-Cl-Bn

White solid, $[\alpha]_D^{25}$ = +34.07 (*c* 1.06, DCM), 22.2 mg, 74% yield, 94% ee, determined by HPLC (Chiralcel column AD-H, hexane/i-PrOH = 80/20, flow rate 1.0 mL/min, UV detection at 254 nm), t_{major} = 6.8 min, t_{minor} = 7.8 min. **^{1}H NMR** (400 MHz, Chloroform-*d*) *δ* 7.42 (d, *J* = 7.4 Hz, 2H), 7.42 (d, *J* = 7.4 Hz, 2H), 7.30 (dd, *J* = 8.4, 6.6 Hz, 2H), 7.27 – 7.21 (m, 3H), 7.20 – 7.11 (m, 4H), 7.00 (d, *J* = 8.3 Hz, 2H), 5.10 (d, *J* = 3.6 Hz, 1H), 5.11 – 4.97 (m, 2H), 5.01 – 4.86 (m, 2H), 4.00 – 3.91 (m, 1H), 3.69 (td, *J* = 11.9, 2.3 Hz, 1H), 2.51 (dd, *J* = 13.0, 6.4 Hz, 1H), 2.31 – 2.00 (m, 6H), 1.67 (t, *J* = 12.6 Hz, 1H), 1.57 (dq, *J* = 15.8, 7.7, 5.4 Hz, 2H), 1.55 – 1.46 (m, 1H), 1.44 (td, *J* = 12.7, 3.7 Hz, 1H), 1.23 (m, 2H). **^{13}C NMR** (101 MHz, Chloroform-*d*) *δ* 172.17, 171.88, 146.33, 134.45, 134.29, 133.95, 133.89, 129.71, 129.41, 128.88, 128.82, 128.16, 126.72, 125.68, 97.62, 80.01, 69.47, 66.62, 66.44, 57.13, 53.81, 40.19, 36.09, 35.67, 33.31, 31.69, 31.38, 21.19. **HRMS** (ESI): m/z Calcd. For $C_{34}H_{34}O_6Cl_2Na$ ($[M+Na]^+$): 631.1625, found: 631.1624.

Bis(4-bromobenzyl)(8aS,11a*R*)-8-phenyldecahydro-2,8-epoxycyclopenta[*d*] oxecine-10,10(4*H*)-dicarboxylate (11d)

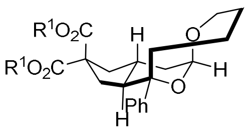

R¹=4-Br-Bn

White solid, $[\alpha]_D^{25}$ = +33.33 (*c* 0.96, DCM), 22.0 mg, 73% yield, 95% ee, determined by HPLC (Chiralcel column AD-H, hexane/i-PrOH = 80/20, flow rate 1.0 mL/min, UV detection at 254 nm), t_{major} = 7.5 min, t_{minor} = 8.9 min. **^{1}H NMR** (400 MHz, Chloroform-*d*) *δ* 7.47 (dd, *J* = 8.0, 3.5 Hz, 4H), 7.43 – 7.32 (m, 4H), 7.28 (d, *J* = 6.9 Hz, 1H), 7.14 (d, *J* = 8.1 Hz, 2H), 7.00 (d, *J* = 8.0 Hz, 2H), 5.16 (d, *J* = 3.3 Hz, 1H), 5.15 – 5.02 (m, 2H), 5.03 – 4.90 (m, 2H), 4.02 (d, *J* = 11.6 Hz, 1H), 3.75 (t, *J* = 11.9 Hz, 1H), 2.57 (dd, *J* = 13.0, 6.4 Hz, 1H), 2.38 – 2.16 (m, 4H), 2.12 (t, *J* = 12.7 Hz, 2H), 1.74 (t, *J* = 12.7 Hz, 1H), 1.69 – 1.44 (m, 4H), 1.31 (d, *J* = 10.1 Hz, 2H). **^{13}C NMR** (101 MHz, Chloroform-*d*) *δ* 172.15, 171.87, 146.35, 134.48, 134.42, 131.87, 131.80, 129.99, 129.70, 128.16, 126.73,

125.69, 122.63, 122.47, 97.63, 80.02, 69.48, 66.65, 66.47, 57.14, 53.83, 40.21, 36.11, 35.70, 33.33, 31.70, 31.40, 21.21. **HRMS** (ESI): m/z Calcd. For $C_{34}H_{34}O_6Br_2Na$ ($[M+Na]^+$): 719.0614, found: 719.0611.

Bis(4-methylbenzyl)(8aS,11a*R*)-8-phenyldecahydro-2,8-epoxycyclopenta[*d*] oxecine-10,10(4*H*)-dicarboxylate (11e)

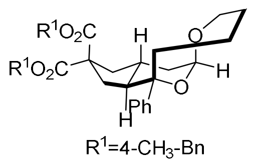

White solid, $[\alpha]_D^{25}$ = +34.78 (*c* 1.27, DCM), 26.4 mg, 88% yield, 95% ee, determined by HPLC (Chiralcel column AD-H, hexane/i-PrOH = 95/5, flow rate 1.0 mL/min, UV detection at 254 nm), t_{major} = 8.7 min, t_{minor} = 9.9 min. **^{1}H NMR** (400 MHz, Chloroform-*d*) δ 7.46 (d, *J* = 7.7 Hz, 2H), 7.33 (t, *J* = 7.6 Hz, 2H), 7.23 (t, *J* = 7.3 Hz, 1H), 7.14 (d, *J* = 3.0 Hz, 4H), 7.09 – 6.91 (m, 4H), 5.13 (s, 1H), 5.12 – 5.01 (m, 2H), 5.04 – 4.92 (m, 2H), 3.98 (dd, *J* = 11.0, 3.7 Hz, 1H), 3.72 (td, *J* = 12.0, 2.2 Hz, 1H), 2.56 (dd, *J* = 13.0, 6.3 Hz, 1H), 2.35 (s, 3H), 2.33 (s, 3H), 2.32 – 2.14 (m, 4H), 2.17 – 2.02 (m, 2H), 1.76 – 1.41 (m, 5H), 1.36 – 1.21 (m, 2H). **^{13}C NMR** (101 MHz, Chloroform-*d*) δ 172.41, 172.14, 146.45, 138.17, 138.02, 132.64, 132.58, 129.29, 129.24, 128.38, 128.10 (2C), 126.62, 125.72, 97.65, 80.09, 69.42, 67.37, 67.20, 57.22, 53.80, 40.20, 36.13, 35.61, 33.28, 31.71, 31.43, 21.35, 21.32, 21.23. **HRMS** (ESI): m/z Calcd. For $C_{36}H_{40}O_6Na$ ($[M+Na]^+$): 591.2717, found: 591.2713.

Bis(4-methoxybenzyl)(8aS,11a*R*)-8-phenyldecahydro-2,8-epoxycyclopenta[*d*] oxecine-10,10(4*H*)-dicarboxylate (11f)

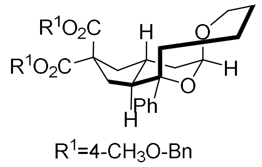

White solid, $[\alpha]_D^{25}$ = +30.56 (*c* 1.00, DCM), 23.3 mg, 78% yield, 94% ee, determined by HPLC (Chiralcel column AD-H, hexane/i-PrOH = 80/20, flow rate 1.0 mL/min, UV detection at 254 nm), t_{major} = 7.5 min, t_{minor} = 8.5 min. **^{1}H NMR** (400 MHz, Chloroform-*d*) δ 7.48 – 7.41 (m, 2H), 7.32 (t, *J* = 7.6 Hz, 2H), 7.27 – 7.14 (m, 3H), 7.09 – 7.02 (m, 2H), 6.92 – 6.80 (m, 2H), 6.84 – 6.70 (m, 2H), 5.12 (d, *J* = 3.4 Hz, 1H), 5.04 (q, *J* = 12.0 Hz, 2H), 4.93 (q, *J* = 12.1 Hz, 2H), 4.02 – 3.93 (m, 1H), 3.80 (s, 3H), 3.78 (s, 3H), 3.71 (td, *J* = 11.9, 2.2 Hz, 1H), 2.53 (dd, *J* = 12.9, 6.4 Hz, 1H), 2.22 (m, 4H), 2.14 – 2.01 (m, 2H), 1.76 – 1.38 (m, 6H), 1.34 – 1.20 (m, 1H). ^{13}C NMR (101 MHz, Chloroform-*d*) δ 172.33,

172.05, 159.60, 159.48, 146.32, 129.98, 129.68, 127.98, 127.63, 127.57, 126.50, 125.59, 113.85, 113.80, 97.52, 79.95, 69.30, 67.11, 66.94, 57.08, 55.26, 55.23, 53.66, 40.04, 35.99, 35.44, 33.13, 31.58, 31.29, 21.09. **HRMS** (ESI): m/z Calcd. For $C_{36}H_{40}O_8Na$ ([M+Na]$^+$): 623.2615, found: 623.2613.

Bis(4-trifluoromethylbenzyl)(8aS,11a*R*)-8-phenyldecahydro-2,8 epoxycyclo-penta[*d*] oxecine- 10,10(4*H*)-dicarboxylate (11g)

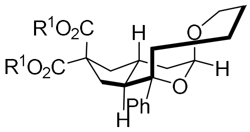

R1=4-CF3-Bn

White solid, $[\alpha]_D^{25}$ = +31.80 (*c* 1.27, DCM), 26.3 mg, 88% yield, 94% ee, determined by HPLC (Chiralcel column AD-H, hexane/i-PrOH = 80/20, flow rate 1.0 mL/min, UV detection at 254 nm), t_{major} = 5.4 min, t_{minor} =6.2 min. **^{1}H NMR** (400 MHz, Chloroform-*d*) δ 7.48 (d, *J* = 8.0 Hz, 2H), 7.39 (dd, *J* = 8.2, 2.6 Hz, 4H), 7.32 – 7.21 (m, 4H), 7.21 – 7.09 (m, 3H), 5.17 – 5.04 (m, 3H), 5.04 – 4.92 (m, 2H), 3.91 (dd, *J* = 11.0, 3.4 Hz, 1H), 3.64 (td, *J* = 11.9, 2.3 Hz, 1H), 2.51 (dd, *J* = 13.0, 6.4 Hz, 1H), 2.28 – 2.14 (m, 3H), 2.13 – 1.98 (m, 3H), 1.68 (t, *J* = 12.6 Hz, 1H), 1.51 (m, 4H), 1.46 – 1.33 (m, 1H), 1.28 – 1.14 (m, 1H). **^{13}C NMR** (101 MHz, Chloroform-*d*) δ 172.07, 171.79, 146.33, 139.40, 139.35, 130.80, 130.48,128.21, 128.19, 127.88, 126.77, 125.73, 125.69, 125.65, 125.64, 125.60, 97.63, 79.99, 69.51, 66.49, 66.29, 57.18, 53.88, 40.28, 36.11, 35.82, 33.38, 31.69, 31.41, 21.20. **^{19}F NMR** (376 MHz, Chloroform-d) δ -62.74 (s, 3F), -62.76 (s, 3F). **HRMS** (ESI): m/z Calcd. For $C_{36}H_{34}O_6F_6Na$ ([M+Na]$^+$): 699.2152, found: 699.2155.

Bis(4-fluorophenyl)-(8a*S*,11a*R*)-8-phenyldecahydro-2,8-epoxycyclopenta[d] oxec-ine-10,10(4*H*)-dicarboxylate (11h)

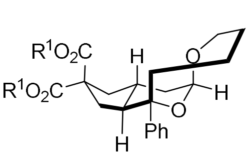

R1=4-F-Ph

Yellow solid, $[\alpha]_D^{25}$ = +49.38 (*c* 1.08, DCM), 28.3 mg, 94% yield, 94% ee, determined by HPLC (Chiralcel column AD-H, hexane/i-PrOH = 80/20, flow rate 1.0 mL/min, UV detection at 254 nm), t_{major} = 6.4min, t_{minor} = 5.6min. **^{1}H NMR** (400 MHz, Chloroform-*d*) δ 7.56 – 7.49 (m, 2H), 7.36 (t, *J* = 7.7 Hz, 2H), 7.25 (t, *J* = 7.3 Hz, 1H), 7.10 (d, *J* = 4.0 Hz, 4H), 7.06 – 6.88 (m, 4H), 5.19 (d, *J* = 3.4 Hz, 1H), 4.02 (dt, *J* = 11.4, 3.3 Hz, 1H), 3.76 (td, *J* = 11.9, 2.3 Hz, 1H), 2.81 (dd, *J* = 13.1, 6.4 Hz, 1H), 2.47 (dd, *J* = 13.2, 7.3 Hz, 1H), 2.43 – 2.35 (m, 1H), 2.35 – 2.25 (m, 1H), 2.18 (dd, *J* = 12.9,

3.7 Hz, 1H), 2.07 – 1.93 (m, 1H), 1.79 – 1.52 (m, 5H), 1.62 (s, 1H), 1.38 – 1.24 (m, 2H). **^{13}C NMR** (101 MHz, Chloroform-*d*) *δ* 171.02, 170.68, 161.77 (*J* = 7.1 Hz), 159.34 (*J* = 8.1 Hz), 146.59 (*J* = 2.0 Hz), 146.49 (*J* = 3.0 Hz), 146.21, 128.26, 126.86, 125.72, 122.70 (*J* = 8.1 Hz), 122.62 (*J* = 8.1 Hz), 116.53 (*J* = 10.1 Hz), 116.29 (*J* = 10.1 Hz), 97.63, 80.04, 69.56, 57.26, 54.01, 40.47, 36.17, 35.92, 33.45, 31.68, 31.39, 21.24. **^{19}F NMR** (471 MHz, Chloroform-d) *δ* -116.12 (s, 1F), -116.20 (s, 1F). **HRMS** (ESI): m/z Calcd. For $C_{32}H_{30}F_2O_6Na$ ($[M+Na]^+$): 571.1903, found: 571.1905.

Bis(4-chlorophenyl)(8a*S*,11a*R*)-8-phenyldecahydro-2,8-epoxycyclopenta[d] oxecine -10,10(4*H*)-dicarboxylate (11i)

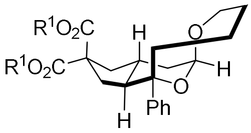

R^1=4-Cl-Ph

Yellow solid, $[\alpha]_D^{25}$ = +53.40 (*c* 1.05, DCM), 25.5 mg, 85% yield, 94% ee, determined by HPLC (Chiralcel column AD-H, hexane/i-PrOH = 90/10, flow rate 1.0 mL/min, UV detection at 254 nm), t_{major} = 11.2min, t_{minor} = 9.5min. **^{1}H NMR** (400 MHz, Chloroform-*d*) *δ* 7.54 (d, *J* = 7.8 Hz, 2H), 7.44 – 7.24 (m, 7H), 7.10 (d, *J* = 8.3 Hz, 2H), 6.98 (d, *J* = 8.3 Hz, 2H), 5.22 (d, *J* = 3.5 Hz, 1H), 4.04 (d, *J* = 11.4 Hz, 1H), 3.78 (t, *J* = 11.8 Hz, 1H), 2.83 (dd, *J* = 13.1, 6.3 Hz, 1H), 2.54 – 2.27 (m, 4H), 2.20 (dd, *J* = 12.9, 3.7 Hz, 1H), 2.00 (t, *J* = 12.6 Hz, 1H), 1.80 – 1.62 (m, 3H), 1.59 (dd, *J* = 11.4, 4.3 Hz, 2H), 1.33 (dd, *J* = 22.1, 7.5 Hz, 2H). **^{13}C NMR** (101 MHz, Chloroform-*d*) *δ* 170.69, 170.34, 149.21, 149.11, 146.18, 131.88, 131.79, 129.84, 129.75, 128.27, 126.88, 125.71, 122.63, 122.55, 97.62, 80.02, 69.57, 57.33, 54.00, 40.45, 36.15, 35.90, 33.46, 31.67, 31.39, 21.23. **HRMS** (ESI): m/z Calcd. For $C_{32}H_{30}Cl_2O_6Na$ ($[M+Na]^+$): 603.1312, found: 603.1312.

Bis(4-bromophenyl)(8a*S*,11a*R*)-8-phenyldecahydro-2,8-epoxycyclopenta[d] oxecine -10,10(4*H*)-dicarboxylate (11j)

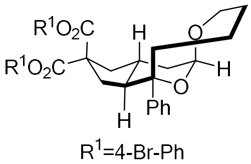

R^1=4-Br-Ph

Yellow solid, $[\alpha]_D^{25}$ = +50.90 (*c* 1.02, DCM), 27.9 mg, 93% yield, 93% ee,determined by HPLC (Chiralcel column AD-H, hexane/i-PrOH = 90/10, flow rate 1.0 mL/min, UV detection at 254 nm), t_{major} = 13.1min, t_{minor} = 11.7min. **^{1}H NMR** (400 MHz, Chloroform-*d*) *δ* 7.36 – 7.29 (m, 4H), 7.25 (d, *J* = 8.0 Hz, 2H), 7.16 (t, *J* = 7.6 Hz, 2H), 7.09 – 7.01 (m, 1H), 6.81 (d, *J* = 8.0 Hz, 2H), 6.69 (d, *J* = 8.0 Hz, 2H), 4.99 (d, *J* = 3.4 Hz, 1H), 3.82 (dt, *J* = 11.6, 3.1

Hz, 1H), 3.55 (td, J = 12.0, 2.4 Hz, 1H), 2.60 (dd, J = 13.1, 6.3 Hz, 1H), 2.31 – 2.04 (m, 5H), 1.98 (dd, J = 12.9, 3.7 Hz, 1H), 1.85 – 1.71 (m, 1H), 1.58 – 1.31 (m, 4H), 1.20 – 1.10 (m, 1H), 1.10 – 1.02 (m, 1H). **^{13}C NMR** (101 MHz, Chloroform-*d*) δ 170.63, 170.28, 149.80, 149.70, 146.22, 132.88, 132.78, 128.31, 126.93, 125.75, 123.08, 123.00, 119.62, 119.53, 97.66, 80.06, 69.61, 57.39, 54.04, 40.49, 36.19, 35.94, 33.50, 31.71, 31.43, 21.27. **HRMS** (ESI): m/z Calcd. For $C_{32}H_{30}Br_2O_6Na$ ([M+Na]$^+$): 691.0301, found: 691.0301.

Dibenzyl(8a*S*,11a*R*)-8-(3-chlorophenyl)decahydro-2,8-epoxycyclopenta[d] oxecine-10,10(4*H*)-dicarboxylate (11k)

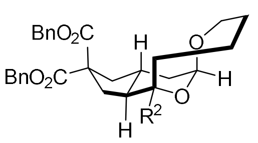

R_2=3-Cl-Ph

Colorless oil, $[\alpha]_D{}^{25}$ = +40.88 (*c* 0.78, DCM), 19.5 mg, 65% yield, 98% ee, determined by HPLC (Chiralcel column AD-H, hexane/i-PrOH = 98/2, flow rate 1.0 mL/min, UV detection at 254 nm), t_{major} = 26.1 min, t_{minor} = 22.9 min. **^{1}H NMR** (400 MHz, Chloroform-*d*) δ 7.51 (s, 1H), 7.33 – 7.29 (m, 2H), 7.29 – 7.18 (m, 10H), 7.14 (d, J = 4.4 Hz, 1H), 5.16 (d, J = 12.3 Hz, 1H), 5.12 – 5.07 (m, 2H), 5.06 – 4.96 (m, 2H), 3.98 (dd, J = 11.2, 3.7 Hz, 1H), 3.70 (td, J = 11.9, 2.2 Hz, 1H), 2.58 (dd, J = 13.0, 6.4 Hz, 1H), 2.28 (dd, J = 13.1, 7.1 Hz, 1H), 2.22 – 2.14 (m, 3H), 2.13 – 2.07 (m, 1H), 2.07 – 2.03 (m, 1H), 1.70 (t, J = 12.6 Hz, 1H), 1.65 – 1.53 (m, 3H),1.51 – 1.38 (m, 2H),1.26 – 1.18 (m, 1H). **^{13}C NMR** (101 MHz, Chloroform-*d*) δ 172.30, 172.04, 148.79, 135.55, 135.49, 134.28, 129.33, 128.68, 128.62, 128.45, 128.35, 128.25, 127.98, 126.84, 126.27, 123.78, 97.63, 79.80, 69.50, 67.48, 67.32, 57.19, 53.57, 40.14, 35.99, 35.55, 33.34, 31.59 (2C), 21.05. **HRMS** (ESI): m/z Calcd. For $C_{34}H_{35}ClO_6Na$ ([M+Na]$^+$): 597.2014, found: 597.2016.

Dibenzyl(8a*S*,11a*R*)-8-(4-chlorophenyl)decahydro-2,8-epoxycyclopenta[d] oxecine-10,10(4*H*)-dicarboxylate (11l)

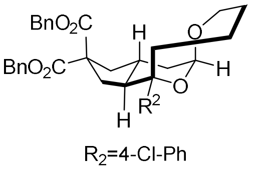

R_2=4-Cl-Ph

Colorless oil, $[\alpha]_D{}^{25}$ = +39.78(*c* 0.69, DCM), 23.7 mg, 79% yield, 99% ee, determined by HPLC (Chiralcel column AD-H, hexane/i-PrOH = 90/10, flow rate 1.0 mL/min, UV detection at 254 nm), t_{major} = 9.8 min, t_{minor} = 7.3 min. **^{1}H NMR** (400 MHz, Chloroform-*d*) δ 7.36 (d, J = 8.5 Hz, 2H), 7.32 – 7.18 (m, 11H), 7.11 (d, J = 6.1 Hz,

1H), 5.13 (d, J = 12.3 Hz, 1H), 5.07 (d, J = 12.1 Hz, 2H), 5.01 (d, J = 12.4 Hz, 1H), 4.97 (d, J = 12.4 Hz, 1H), 3.96 (dd, J = 11.3, 3.5 Hz, 1H), 3.73 – 3.62 (m, 1H), 2.56 (dd, J = 13.0, 6.4 Hz, 1H), 2.26 – 2.10 (m, J = 6.6 Hz, 4H), 2.11 – 2.00 (m, 2H), 1.69 (t, J = 12.5 Hz, 1H), 1.66 – 1.48 (m, 3H), 1.51 – 1.37 (m, 2H), 1.24 – 1.15 (m, 1H). **^{13}C NMR** (101 MHz, Chloroform-*d*) δ 171.97, 171.74, 144.77, 135.23, 135.15, 132.11, 128.36, 128.30, 128.12, 128.04, 127.92, 127.90, 127.69, 126.94, 97.33, 79.46, 69.16, 67.14, 66.99, 56.84, 53.40, 39.77, 35.72, 35.29, 32.96, 31.27, 31.12, 20.75. **HRMS** (ESI): m/z Calcd. For $C_{34}H_{35}ClO_6Na$ ([M+Na]$^+$): 597.2014, found: 597.2012.

Dibenzyl(8a*S*,11a*R*)-8-(m-tolyl)decahydro-2,8-epoxycyclopenta[d]oxecine-10,10-(4*H*)-dicarboxylate (11m)

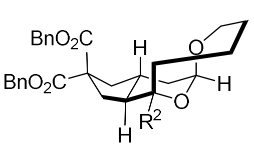

Colorless oil, $[\alpha]_D^{25}$ = +37.82 (*c* 0.80, DCM), 23.8 mg, 79% yield, 91% ee, determined by HPLC (Chiralcel column AD-H, hexane/i-PrOH = 90/10, flow rate 1.0 mL/min, UV detection at 254 nm), t_{major} = 6.5min, t_{minor} = 6.0min. **^{1}H NMR** (400 MHz, Chloroform-*d*) δ 7.37 – 7.31 (m, 4H), 7.31 – 7.25 (m, 5H), 7.24 (d, J = 4.7 Hz, 2H), 7.23 – 7.13 (m, 2H), 7.11 – 7.04 (m, 1H), 5.23 – 5.13 (m, 2H), 5.12 (d, J = 12.3 Hz, 1H), 5.07 (d, J = 12.4 Hz, 1H), 5.01 (d, J = 12.4 Hz, 1H), 4.06 – 3.97 (m, 1H), 3.75 (td, J = 12.0, 2.3 Hz, 1H), 2.59 (dd, J = 12.9, 6.3 Hz, 1H), 2.39 (s, 3H), 2.36 – 2.22 (m, 4H), 2.21 – 2.01 (m, 3H), 1.76 (t, J = 12.6 Hz, 1H), 1.71 – 1.46 (m, 4H), 1.37 – 1.26 (m, 1H).**^{13}C NMR** (101 MHz, Chloroform-*d*) δ 172.42, 172.11, 146.35, 137.60, 135.60, 135.55, 128.65, 128.59, 128.40, 128.27, 128.22, 127.93 (2C), 127.45, 126.50, 122.70, 97.63, 80.11, 69.41, 67.41, 67.23, 57.22, 53.84, 40.26, 36.12, 35.64, 33.27, 31.72, 31.32, 21.95, 21.27. **HRMS** (ESI): m/z Calcd. For $C_{35}H_{38}O_6Na$ ([M+Na]$^+$): 577.2561, found: 577.2565.

Dibenzyl(8a*S*,11a*R*)-8-(p-tolyl)decahydro-2,8-epoxycyclopenta[d]oxecine-10,10-(4*H*)-dicarboxylate (11n)

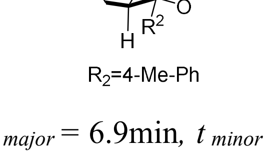

Colorless oil, $[\alpha]_D^{25}$ = +36.04 (*c* 0.83, DCM), 21.2 mg, 71% yield, 97% ee,determined by HPLC (Chiralcel column AD-H, hexane/i-PrOH = 90/10, flow rate 1.0 mL/min, UV detection at 254 nm), t_{major} = 6.9min, t_{minor} = 6.0min. **^{1}H NMR** (400 MHz, Chloroform-*d*) δ 7.39 – 7.31 (m,

5H), 7.27 (d, J = 6.4 Hz, 5H), 7.15 (d, J = 8.0 Hz, 4H), 5.21 – 5.13 (m, 2H), 5.11 (d, J = 12.3 Hz, 1H), 5.04 (q, J = 12.4 Hz, 2H), 4.00 (dt, J = 11.3, 3.0 Hz, 1H), 3.73 (td, J = 11.9, 2.2 Hz, 1H), 2.59 (dd, J = 13.0, 6.4 Hz, 1H), 2.36 (s, 3H), 2.34 – 2.16 (m, 4H), 2.14 – 2.02 (m, 2H), 1.74 (t, J = 12.5 Hz, 1H), 1.66 – 1.56 (m, 4H), 1.55 – 1.43 (m, 1H), 1.36 – 1.26 (m, 1H). **^{13}C NMR** (101 MHz, Chloroform-*d*) δ 172.41, 172.11, 143.48, 135.56, 128.84, 128.65, 128.58, 128.39, 128.26, 128.21, 127.95, 125.64, 97.64, 80.00, 69.40, 67.40, 67.22, 57.21, 53.96, 40.25, 36.14, 35.65, 33.26, 31.73, 31.35, 21.27, 21.08. **HRMS** (ESI): m/z Calcd. For $C_{35}H_{38}O_6Na$ ([M+Na]$^+$): 577.2561, found: 597.2568.

Dibenzyl(8a*S*,11a*R*)-8-(3-methoxyphenyl)decahydro-2,8-epoxycyclopenta[d] oxecine-10,10(4*H*)-dicarboxylate (11o)

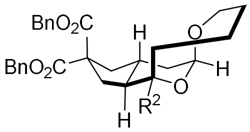

R2=3-MeO-Ph

Colorless oil, [α]$_D$^{25} = +37.33 (*c* 0.91, DCM), 20.8 mg, 69% yield, 93% ee, determined by HPLC (Chiralcel column AD-H, hexane/i-PrOH = 90/10, flow rate 1.0 mL/min, UV detection at 254 nm), t_{major} =7.1min, t_{minor} = 6.1min. **^{1}H NMR** (400 MHz, Chloroform-*d*) δ 7.30 (q, J = 3.7 Hz, 3H), 7.28 – 7.18 (m, 6H), 7.15 – 7.02 (m, 3H), 6.99 (d, J = 7.8 Hz, 1H), 6.77 (dd, J = 8.2, 2.6 Hz, 1H), 5.18 – 5.08 (m, 2H), 5.12 – 5.00 (m, 2H), 4.97 (d, J = 12.4 Hz, 1H), 4.01 – 3.92 (m, 1H), 3.80 (s, 3H), 3.70 (td, J = 12.0, 2.2 Hz, 1H), 2.56 (dd, J = 13.0, 6.3 Hz, 1H), 2.32 – 2.18 (m, 4H), 2.13 – 2.02 (m, 2H), 1.70 (t, J = 12.5 Hz, 1H), 1.61 – 1.40 (m, 5H), 1.29 (dd, J = 11.9, 7.5 Hz, 1H). **^{13}C NMR** (101 MHz, Chloroform-*d*) δ 172.39, 172.10, 159.56, 148.34, 135.60, 135.56, 129.00, 128.66, 128.59, 128.40, 128.28, 128.23, 127.95, 118.12, 112.39, 111.36, 97.61, 80.01, 69.44, 67.42, 67.24, 57.21, 55.32, 53.65, 40.25, 36.07, 35.70, 33.31, 31.69, 31.53, 21.20. **HRMS** (ESI): m/z Calcd. For $C_{35}H_{38}O_7Na$ ([M+Na]$^+$): 593.2510, found: 593.2512.

Dibenzyl(8a*S*,11a*R*)-8-(4-bromophenyl)decahydro-2,8-epoxycyclopenta[d] oxecine-10,10(4*H*)-dicarboxylate (11p)

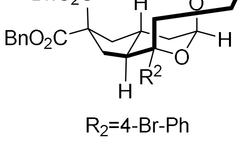

R2=4-Br-Ph

Colorless oil,[α]$_D$^{25} = +33.85 (*c* 1.08, DCM), 23.4 mg, 78% yield, 99% ee, determined by HPLC (Chiralcel column AD-H, hexane/i-PrOH = 90/10, flow rate 1.0 mL/min, UV detection at 254 nm), t_{major} = 11.1min, t_{minor} = 7.6min.^{1}H NMR (400 MHz, Chloroform-*d*) δ 7.39

(d, J = 8.4 Hz, 2H), 7.31 – 7.26 (m, 5H), 7.25 – 7.17 (m, 5H),7.14 – 7.03 (m, 2H), 5.11 (d, J = 12.3 Hz, 1H), 5.05 (d, J = 12.3 Hz, 2H), 5.04 – 4.92 (m, 2H), 3.94 (dd, J = 11.3, 3.7 Hz, 1H), 3.71 – 3.60 (m, 1H), 2.54 (dd, J = 13.0, 6.4 Hz, 1H), 2.25 – 2.05 (m, 4H), 2.05 – 1.99 (m, 2H), 1.61 (dt, J = 52.0, 12.7 Hz, 4H), 1.49 – 1.35 (m, 2H), 1.25 – 1.14 (m, 1H).**^{13}C NMR** (101 MHz, Chloroform-*d*) δ 172.31, 172.07, 145.67, 135.58, 135.50, 131.21, 128.71, 128.66, 128.48, 128.40, 128.27, 128.04, 127.68, 120.68, 97.67, 79.84, 69.52, 67.51, 67.36, 57.19, 53.68, 40.12, 36.07, 35.64, 33.31, 31.62, 31.47, 21.10. **HRMS** (ESI): m/z Calcd. For $C_{34}H_{35}BrO_6Na$ ([M+Na]$^+$): 641.1509, found: 641.1506.

2.7 Procedure for derivatization of IEDHDA products

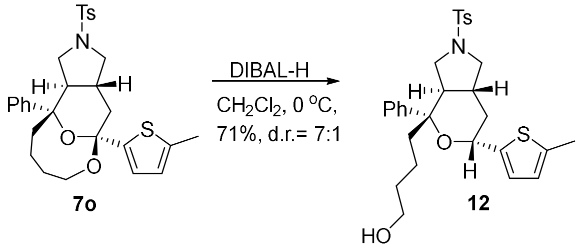

DIBAL-H (0.28 ml, 1.0 M in Toluene, 20.0 eq,) was added to a solution of **7o** (1.0 eq, 11.0 mg) in dry DCM at 0 °C. When the reaction completed by TLC analysis, then, the mixture was quenched with saturated potassium and sodium tartrate solution, and washed with brine, extracted with ether. The organic layer was dried over Na_2SO_4, filtered, and concentrated in *vacuo*. The residue was purified by flash column chromatography on silica gel to afford alcohol **12** (7.8 mg, 71%).

4-((3a*R*,4*S*,6*R*,7a*R*)-6-(5-methylthiophen-2-yl)-4-phenyl-2 tosyloctahydro-pyrano[3,4-*c*]pyrrol-4-yl)butan-1-ol (12)

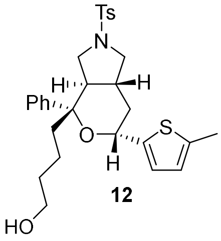

Colorless oil, $[\alpha]_D^{25}$ = + 5.28 (*c* 0.78, Acetone), 71% yield, 97% ee, determined by HPLC (Chiralcel column AD-H, hexane/i-PrOH = 80/20, flow rate 1.0 mL/min, UV detection at 254 nm), t_{major} = 36.8min, t_{minor} = 14.2min. **^{1}H NMR** (600 MHz, Chloroform-*d*) δ 7.67 – 7.62 (m, 2H), 7.38 – 7.29 (m, 4H), 7.28 – 7.21 (m, 3H), 6.74 (d, J = 3.4 Hz, 1H), 6.63 – 6.59 (m, 1H), 4.78 (dd, J = 11.3, 2.3 Hz, 1H), 3.69 (dd, J = 9.2, 6.9 Hz, 1H), 3.52 (t, J = 6.6 Hz, 2H), 3.40 (dd, J = 9.3, 7.5 Hz, 1H), 3.26 (dd, J = 11.6, 9.4 Hz, 1H), 2.71 (dd, J = 10.6, 9.3 Hz, 1H), 2.47 (d, J = 1.1 Hz, 3H), 2.41 (s, 3H), 2.27 – 2.16 (m, 2H),

2.15 – 2.10 (m, 1H), 1.83 – 1.75 (m, 1H), 1.65 – 1.58 (m, 1H), 1.52 – 1.46 (m, 1H), 1.48 – 1.41 (m, 1H), 1.43 – 1.36 (m, 1H), 1.37 – 1.26 (m, 1H), 1.01 – 0.89 (m, 1H). ^{13}C NMR (151 MHz, Chloroform-*d*) *δ* 144.88, 143.61, 143.59, 139.27, 134.77, 129.88, 128.46, 127.29, 126.97, 124.71, 124.58, 123.09, 79.90, 68.65, 62.89, 52.93, 52.71, 49.03, 37.90, 36.96, 32.93, 28.00, 21.68, 18.75, 15.50. **HRMS** (ESI): m/z calcd for $C_{29}H_{35}NO_4S_2Na$ ($[M+Na]^+$): 548.1900, found: 548.1901.

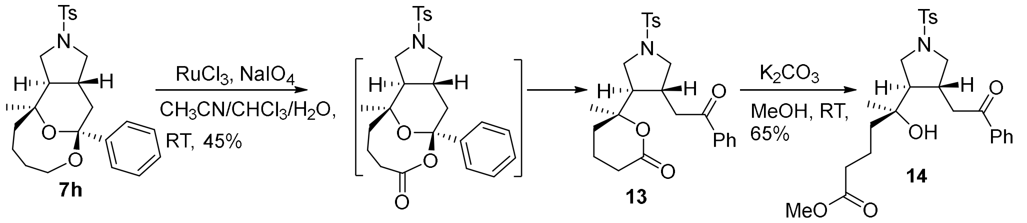

Compound **7h** (9.0 mg, 1.0 eq) was dissolved in a mixture solution of chloroform (0.06 ml), acetonitrile (0.31 ml), and water (0.53 ml), then ruthenium chloride (1.3 mg, 0.3 eq) and sodium periodate (131.0 mg, 30 eq) were added respectively, and the resulting mixture was stirred at 0 °C about 20 minutes. Then the mixture was filtered, quenched with water, extracted with ethyl acetate three times. The residue was purified by flash column chromatography on silica gel to provide **13** (4.2 mg, 45% yield).

Potassium Carbonate (24.0 mg, 20.0 eq) was added to a solution of **13** (4.0 mg, 1.0 eq) in MeOH (0.5 ml). The reaction was stirred overnight at room temperature. When the reaction was completed, which was quenched by water, extracted by ether, and concentrated under reduced pressure to obtain a crude product. The residue was purified by flash column chromatography on silica gel to afford compound **14** (2.8 mg, 65% yield).

(3a*R*,11a*R*)-11-methyl-5-phenyl-2-tosyldecahydro-5,11-epoxyoxecino[4,5-*c*]pyrrol-7(1*H*)-one (13)

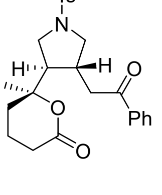

Light yellow oil, $[\alpha]_D^{25}$ = +7.36 (*c* 0.28, Acetone), 45% yield, 93% ee, determined by HPLC (Chiralcel column AD-H, hexane/i-PrOH = 80/20, flow rate 1.0 mL/min, UV detection at 254 nm), t_{major} = 39.3min, t_{minor} = 30.6min. **^{1}H NMR** (400 MHz, Chloroform-*d*) *δ* 7.88 (d, *J* = 7.7 Hz, 2H), 7.69 (d, *J* = 7.9 Hz, 2H), 7.57 (t, *J* = 7.3 Hz, 1H), 7.45 (t, *J* = 7.6 Hz, 2H), 7.33 (d, *J* =

7.9 Hz, 2H), 3.52 (dd, J = 10.0, 7.4 Hz, 1H), 3.35 (t, J = 9.4 Hz, 1H), 3.24 (dd, J = 17.7, 4.3 Hz, 1H), 3.09 (dd, J = 10.4, 6.0 Hz, 1H), 2.99 (dd, J = 17.7, 9.3 Hz, 1H), 2.85 (dd, J = 9.9, 5.4 Hz, 1H), 2.82 – 2.73 (m, 1H), 2.58 – 2.47 (m, 1H), 2.43 (s, 3H), 2.41 – 2.23 (m, 2H), 1.89 (dd, J = 11.2, 6.5 Hz, 2H), 1.75 (td, J = 13.2, 11.3, 6.6 Hz, 2H), 1.31 (s, 3H). **^{13}C NMR** (151 MHz, Chloroform-*d*) δ 198.39, 170.46, 144.06, 136.45, 133.65, 131.95, 129.89, 128.86, 128.15, 128.10, 85.36, 54.25, 53.54, 48.70, 44.12, 34.95, 29.55, 29.03, 25.00, 21.74, 16.60. **HRMS** (ESI): m/z calcd for $C_{25}H_{29}NO_5SNa$ ([M+Na]$^+$): 478.1659, found: 478.1657.

Methyl5-hydroxy-5-((3*R*,4*R*)-4-(2-oxo-2-phenylethyl)-1-tosylpyrrolidin-3-yl)hex-anoate (14)

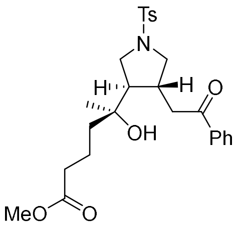

Light yellow oil, $[\alpha]_D^{25}$ = +22.26 (*c* 0.14, Acetone), 65% yield, 93% ee, determined by HPLC (Chiralcel column AD-H, hexane/i-PrOH = 50/50, flow rate 0.6 mL/min, UV detection at 254 nm), t_{major} = 16.4min, t_{minor} = 10.9min. **^{1}H NMR** (600 MHz, Acetone-d_6) δ 7.99 – 7.94 (m, 2H), 7.73 – 7.68 (m, 2H), 7.65 – 7.59 (m, 1H), 7.55 – 7.48 (m, 2H), 7.42 (d, J = 7.9 Hz, 2H), 3.60 (s, 3H), 3.38 – 3.27 (m, 3H), 3.11 (dd, J = 10.0, 7.1 Hz, 1H), 3.07 (dd, J = 17.9, 10.2 Hz, 1H), 2.92 (dd, J = 10.0, 4.8 Hz, 1H), 2.75 – 2.67 (m, 1H), 2.42 (s, 3H), 2.27 (td, J = 7.4, 1.7 Hz, 2H), 2.20 – 2.13 (m, 1H), 1.73 – 1.57 (m, 2H), 1.41 (dd, J = 9.3, 7.6 Hz, 2H), 1.12 (s, 3H). **^{13}C NMR** (151 MHz, Acetone-d_6) δ 199.47, 174.07, 144.34, 137.88, 133.91, 133.74, 130.46, 129.46, 128.84, 128.77, 72.98, 55.09, 53.50, 51.49, 49.58, 45.02, 40.27, 35.54, 34.67, 25.10, 21.43, 20.12. **HRMS** (ESI): m/z calcd for $C_{26}H_{33}NO_6SNa$ ([M+Na]$^+$): 510.1921, found: 510.1927.

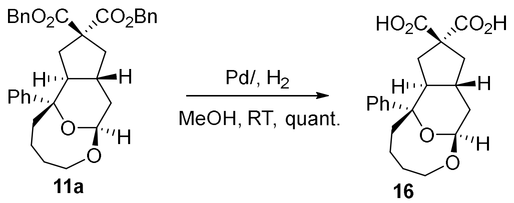

Compound **11a** (10.0 mg, 0.02 mmol) was dissolved in MeOH (0.6 ml), then Palladium on activated carbon was added. The flask was vacuumed and recharged with hydrogen in a balloon three times. The mixture was stirred overnight at room temperature, then

filtered and concentrated under reduced pressure to get **16** (6.5 mg, quant.) without further purification.

(8a*S*, 11a*R*)-8-phenyldecahydro-2,8-epoxycyclopenta[*d*]oxecine-10,10(4*H*)-dicarboxylic acid (16)

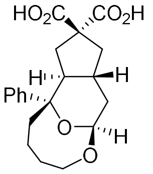

Light yellow oil, $[\alpha]_D{}^{25}$ = +26.11 (*c* 0.88, DCM), quant. yield. **^{1}H NMR** (600 MHz, Acetone-d_6) δ 7.57 – 7.52 (m, 2H), 7.34 (t, *J* = 7.8 Hz, 2H), 7.25 – 7.19 (m, 1H), 5.12 (d, *J* = 3.7 Hz, 1H), 3.97 – 3.91 (m, 1H), 3.67 (td, *J* = 12.0, 2.2 Hz, 1H), 2.58 (dd, *J* = 12.8, 6.4 Hz, 1H), 2.40 (dd, *J* = 14.4, 10.2 Hz, 1H), 2.32 (dd, *J* = 13.0, 7.0 Hz, 1H), 2.29 – 2.16 (m, 2H), 2.12 – 2.07 (m, 1H), 1.68 – 1.53 (m, 2H), 1.52 – 1..45 (m, 2H), 1.27 (td, *J* = 9.5, 3.8 Hz, 4H).**^{13}C NMR** (151 MHz, Acetone-d_6) δ 174.39, 174.00, 147.82, 128.66, 127.18, 126.52, 98.46, 80.59, 69.76, 56.97, 54.84, 41.06, 36.76, 36.52, 34.13, 32.43, 32.19, 21.86. **HRMS** (ESI): m/z Calcd. $C_{20}H_{23}O_6$ ($[M-H]^-$): 359.1500, found: 359.1500.

(8a*S*,10*R*,11a*R*)-2-methyl-8-phenyldodecahydro-2,8-epoxycyclopenta[*d*]oxecine-10-carboxylic acid (17)

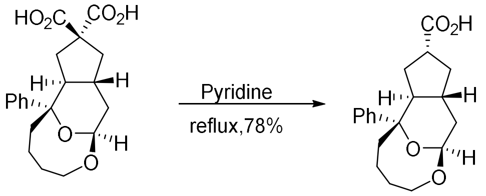

16 17

A sealed tube was charged with **16** (8.0 mg, 0.02 mmol) and pyridine (0.6 mL), then heated at 118 °C and stirred for 6 hours. Then pyridine was evaporated under high vacuum and then the residue was purified by silica gel chromatography to get the **17** (5.3 mg, 78% yield).

(2S,8S,8aS,10R,11aR)-8-phenyldodecahydro-2,8-epoxycyclopenta[d]oxecine-10-carboxylic acid (17)

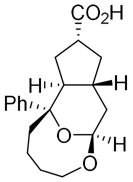

Colorless oil, $[\alpha]_D{}^{25}$ = +32.27 (*c* 0.53, DCM), 78% yield, 95% ee, determined by HPLC (Chiralcel column AD-H, hexane/i-PrOH（0.1%TFA）= 97/3, flow rate 1.0 mL/min, UV detection at 210 nm), t_{major} = 9.4min, t_{minor} = 8.8min. **^{1}H NMR** (800 MHz, Acetone-d_6) *δ* 7.55 – 7.50 (m, 2H), 7.35 – 7.29 (m,

2H), 7.22 – 7.18 (m, 1H), 5.11 (d, J = 3.8 Hz, 1H), 3.94 (ddt, J = 11.8, 4.0, 1.9 Hz, 1H), 3.67 (tt, J = 12.0, 2.9 Hz, 1H), 2.38 (dd, J = 14.4, 10.3 Hz, 1H), 2.25-2.19 (m, 2H), 2.15 – 2.07 (m, 1H), 1.89 – 1.83 (m, 1H), 1.82 (dd, J = 12.5, 11.1 Hz, 1H), 1.62 – 1.50 (m, 2H), 1.49 – 1.41 (m, 2H), 1.39 – 1.31 (m, 1H), 1.31 – 1.21 (m, 4H). ^{13}C NMR (201 MHz, Acetone-d_6) δ 177.55, 148.09, 128.59, 127.08, 126.53, 98.50, 80.87, 69.72, 54.85, 39.91, 37.12, 36.57, 34.76, 32.51, 32.18, 30.96, 21.91.**HRMS** (ESI): m/z Calcd. For $C_{19}H_{23}O_4$ ([M-H]$^-$): 315.1602, found: 315.1605.

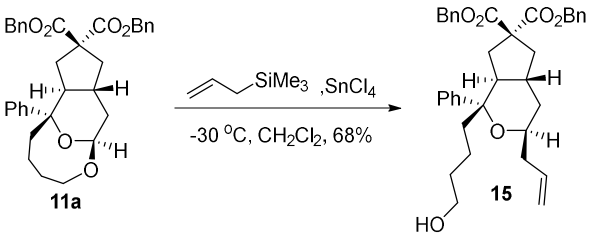

A flame dried round bottom flask equipped with a stirring bar was charged with CH_2Cl_2 (0.4 mL), **11a** (4.4 mg, 0.01 mmol) and allyltrimethylsilane (0.1 mL, 0.20 mmol). The reaction was then cooled to -30°C and $SnCl_4$ (1.0 M in methylene chloride, 0.1mL, 0.1 mmol) was slowly added dropwise. The resulting mixture was stirred for 6 h at this temperature and terminated by addition of a saturated aqueous $NaHCO_3$ solution which was extracted with Et_2O.The organic phases were combined, dried over Na_2SO_4, filtered and concentrated under reduced pressure and the residue was purified by silica gel chromatography to get product **15** (3.2mg, 68% yield).

Dibenzyl(1*S*,3*S*,4a*R*,7a*S*)-3-allyl-1-pentyl-1-phenylhexahydrocyclopenta[*c*]pyran-6,6(1*H*)-dicarboxylate (15)

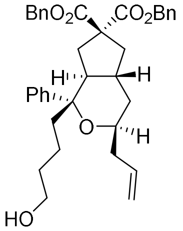

Colorless oil, $[\alpha]_D^{25}$ = -13.01(c 0.28, DCM), 68% yield, 95% ee, determined by HPLC (Chiralcel column AD-H, hexane/i-PrOH = 90/10, flow rate 1.0 mL/min, UV detection at 220 nm), t_{major} = 14.8min, t_{minor} = 10.6min. **^{1}H NMR** (600 MHz, Acetone-d_6) δ 7.42 – 7.26 (m, 14H), 7.25 – 7.17 (m, 1H), 5.93 - 5.86 (m, 1H), 5.18 (d, J = 3.4 Hz, 2H), 5.15 – 5.11 (m, 3H), 5.09 – 5.03 (m, 2H), 3.50 (dq, J = 9.5, 6.3 Hz, 1H), 3.42 – 3.39 (m, 2H), 3.32 – 3.29 (m, 3H), 2.67 (dd, J = 13.2, 6.8 Hz, 1H), 2.43 – 2.35 (m, 2H), 2.26 (dt, J = 13.7, 6.7 Hz, 1H), 1.83 – 1.69 (m, 3H), 1.56 – 1.51 (m, 1H), 1.38 (d, J = 3.6 Hz, 1H), 1.31 (d, J =

22.4 Hz, 3H).^{13}**C NMR** (151 MHz, Acetone-*d*$_6$) δ 172.61, 172.58, 147.99, 136.96, 136.94, 135.98, 129.34, 129.31, 129.00, 128.95, 128.86, 128.80, 128.73, 127.17, 126.39, 117.21, 81.21, 68.84, 67.69, 67.65, 62.44, 59.81, 50.55, 41.85, 40.19, 38.75, 37.11, 37.03, 36.42, 34.26, 20.48. **HRMS** (ESI): m/z calcd for $C_{37}H_{42}O_6Na$ ([M+Na]$^+$): 605.2874, found: 605.2871.

2.8 Synthesis and characterization of **19**

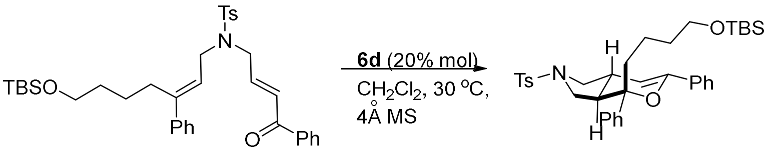

Substrate **18** (50.0 mg) was dissolved in 1.0 ml DCM, then 120 mg 4Å MS and 20 mol% **6d** were added. The reaction was then warmed up to 30°C and stirred for 48 hours. When the reaction was completed, the reaction mixture was purified by silica gel column to afford product **19** (20.1mg, 41% yield).

4-(4-((tert-butyldimethylsilyl)oxy)butyl)-4,6-diphenyl-2-tosyl-1,2,3,3a,4,7a-hexa-hydropyrano[3,4-*c*]pyrrole (19)

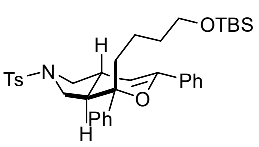

Corlorless oil, $[\alpha]_D^{25}$ = -37.90 (*c* 1.80, Acetone), 41% yield, 49% ee, determined by HPLC (Chiralcel column AD-H, hexane/i-PrOH = 80/20, flow rate 1.0 mL/min, UV detection at 254 nm), t_{major} = 8.0min , t_{minor} =10.7min. ^{1}**H NMR** (500 MHz, Acetone-*d*$_6$) δ 7.73 – 7.67 (m, 4H), 7.50 – 7.45 (m, 2H), 7.42 (dd, *J* = 8.6, 7.0 Hz, 2H), 7.39 – 7.34 (m, 4H), 7.34 – 7.29 (m, 2H), 5.59 (d, *J* = 1.8 Hz, 1H), 3.83 (dd, *J* = 9.0, 7.0 Hz, 1H), 3.50 (t, *J* = 5.7 Hz, 2H), 3.42 (dd, *J* = 11.9, 9.5 Hz, 1H), 3.21 (dd, *J* = 9.4, 7.1 Hz, 1H), 2.87 (dd, *J* = 11.3, 9.0 Hz, 1H), 2.70 – 2.59 (m, 1H), 2.39 (s, 3H), 2.13 – 2.07 (m, 1H), 1.96 – 1.81 (m, 2H), 1.48 – 1.36 (m, 3H), 1.22 – 1.06 (m, 1H), 0.78 (s, 9H), -0.07 (d, *J* = 8.6 Hz, 6H). ^{13}**C NMR** (126 MHz, Acetone-*d*$_6$) δ 151.80, 144.29, 144.24, 136.52, 135.85, 130.54, 129.27, 129.10, 129.08, 128.07, 127.85, 125.57, 125.55, 95.18, 83.30, 63.16, 53.02, 52.79, 49.50, 36.59, 33.58, 30.88, 26.18, 21.35, 20.40, 18.63, -5.27, -5.29. **HRMS** (ESI): m/z calcd for $C_{36}H_{47}NO_4SSiNa$ ([M+Na]$^+$): 640.2887, found: 640.2889.

2.9 Syntheses of substrate **5u-w**

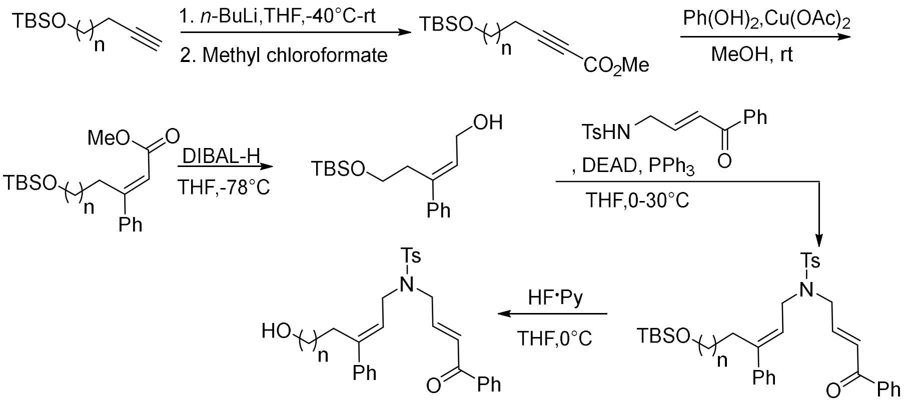

5u-w

N-((*E*)-6-hydroxy-3-phenylbut-2-en-1-yl)-4-methyl-*N*-((*E*)-4-oxo-4-phenylbut-2-en-1-yl) benzenesulfonamide (5u)

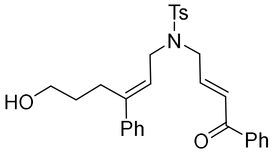

Light yellow oil. **^{1}H NMR** (400 MHz, Chloroform-*d*) δ 7.82 (d, *J* = 7.6 Hz, 2H), 7.73 (d, *J* = 8.2 Hz, 2H), 7.53 (t, *J* = 7.4 Hz, 1H), 7.40 (t, *J* = 7.6 Hz, 2H), 7.27 (d, *J* = 8.1 Hz, 3H), 7.22 (d, *J* = 10.0 Hz, 3H), 7.20 – 7.13 (m, 1H), 7.00 (d, *J* = 15.5 Hz, 1H), 6.82 (dt, *J* = 15.5, 5.2 Hz, 1H), 5.48 (t, *J* = 6.8 Hz, 1H), 4.08 (t, *J* = 6.5 Hz, 4H), 3.48 (t, *J* = 6.0 Hz, 2H), 2.52 (t, *J* = 7.5 Hz, 2H), 2.37 (s, 3H), 1.55 – 1.44 (m, 2H). **^{13}C NMR** (101 MHz, Chloroform-*d*) δ 190.22, 144.38, 143.75, 142.69, 141.37, 137.21, 136.85, 133.15, 129.95, 128.67, 128.40, 127.59, 127.53, 127.27, 127.01, 126.34, 122.60, 61.61, 48.58, 45.98, 31.16, 25.78, 21.55. **HRMS** (ESI+): m/z Calcd. For $C_{29}H_{31}NO_4SNa$ ($[M+Na]^+$): 512.1866, found $[M+Na]^+$: 512.1867.

N-((*E*)-5-hydroxy-3-phenylbut-2-en-1-yl)-4-methyl-*N*-((*E*)-4-oxo-4-phenylbut-2-en-1-yl) benzenesulfonamide (5v)

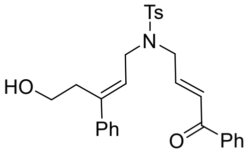

Light yellow oil. **^{1}H NMR** (400 MHz, Chloroform-*d*) δ 7.95 – 7.88 (m, 2H), 7.82 (d, *J* = 8.1 Hz, 2H), 7.61 (t, *J* = 7.4 Hz, 1H), 7.48 (t, *J* = 7.7 Hz, 2H), 7.36 (d, *J* = 8.1 Hz, 2H), 7.31 (q, *J* = 4.5, 3.5 Hz, 3H), 7.27 (dd, *J* = 7.4, 2.4 Hz, 2H), 7.11 (d, *J* = 15.5 Hz, 1H), 6.92 (dt, *J* = 15.4, 5.2 Hz, 1H), 5.70 (t, *J* = 6.9 Hz, 1H), 4.17 (t, *J* = 6.4 Hz, 4H), 3.61 (t, *J* = 6.6 Hz, 2H), 2.77 (t, *J* = 6.6 Hz, 2H), 2.45 (s, 3H). **^{13}C NMR** (101 MHz, Chloroform-*d*) δ 190.17, 143.71, 142.85, 141.13, 141.08, 137.13, 136.69, 133.08, 129.90, 128.60, 128.36 (2C),

127.57, 127.46, 127.21, 126.34, 124.69, 60.72, 48.70, 46.24, 33.10, 21.48. **HRMS** (ESI+): m/z Calcd. For $C_{28}H_{29}NO_4SNa$ ([M+Na] $^+$): 498.1710, found [M+Na]$^+$: 498.1710.

***N*-((Z)-4-hydroxy-3-phenylbut-2-en-1-yl)-4-methyl-*N*-((*E*)-4-oxo-4-phenylbut-2-en-1-yl) benzenesulfonamide (5w)**

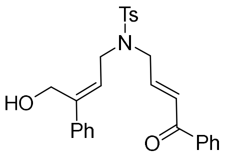

Light yellow oil. **^{1}H NMR** (400 MHz, Chloroform-*d*) δ 7.87 – 7.80 (m, 2H), 7.73 (d, J = 8.0 Hz, 2H), 7.55 (t, J = 7.4 Hz, 1H), 7.42 (t, J = 7.6 Hz, 2H), 7.29 (s, 4H), 7.28 – 7.21 (m, 3H), 7.02 (d, J = 15.5 Hz, 1H), 6.82 (dt, J = 15.4, 5.4 Hz, 1H), 5.69 (t, J = 7.3 Hz, 1H), 4.48 (s, 2H), 4.14 – 4.07 (m, 4H), 2.39 (s, 3H). **^{13}C NMR** (101 MHz, Chloroform-*d*) δ 190.13, 143.82, 142.89, 142.54, 139.94, 137.09, 136.66, 133.12, 129.94, 128.64, 128.62, 128.45, 127.77, 127.71, 127.23, 126.35, 125.09, 59.25, 48.88, 45.74, 21.50. **HRMS** (ESI+): m/z Calcd. For $C_{27}H_{27}NO_4SNa$ ([M+Na]$^+$): 484.1553, found[M+Na]$^+$: 484.1551.

2.10 Synthesis and characterization of **7u-w**

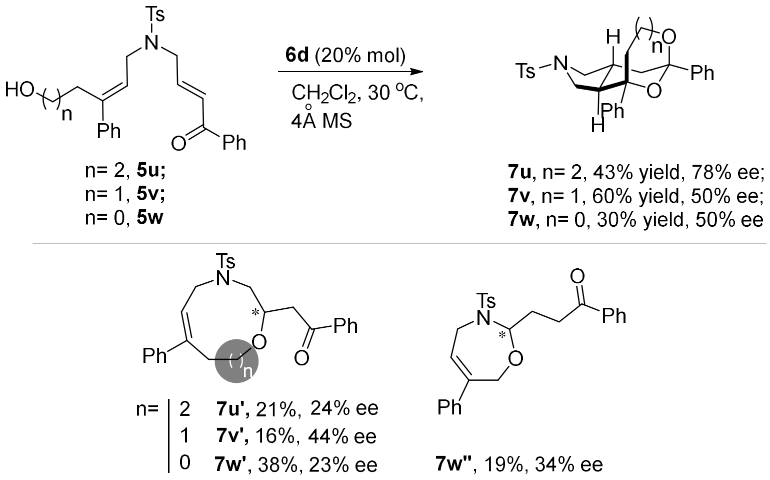

Substrate **5u-w** (30.0 mg) was dissolved in 1.0 ml DCM, then 60 mg 4Å MS and 20 mol% **6d** were added. The reaction was then warmed up to 30ºC and stirred for 48 hours. When the reaction was completed, the reaction mixture was purified by silica gel column to afford product **7u-w** as well as the by products **7u'-w'** and **7w''**.

(3a*R*, 5*R*, 10*S*, 10a*R*)-5, 10-diphenyl-2-tosyldodecahydro-1*H*-5, 10-epoxyoxocino [4, 5-*c*] pyrrole (7u)

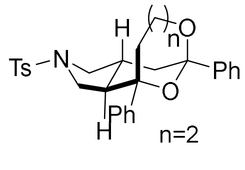

n=2, light yellow oil, 12.9 mg, $[\alpha]_D^{25}$ = + 40.09 (*c* 1.10, DCM), 43% yield, 78% ee, determined by HPLC (Chiralcel column AD-H, hexane/i-PrOH = 80/20, flow rate 1.0 mL/min, UV detection at 254 nm), t_{major} = 9.6min, t_{minor} = 23.6min. **^{1}H NMR** (400 MHz, Acetone-d_6) δ 7.77 (d, J = 8.0 Hz, 2H), 7.64 (d, J = 7.7 Hz, 4H), 7.53 – 7.39 (m, 6H), 7.34 (dt, J = 14.2, 7.2 Hz, 2H), 3.70 (dd, J = 9.3, 7.0 Hz, 1H), 3.62 – 3.49 (m, 2H), 3.47 (dd, J = 9.4, 7.4 Hz, 1H), 3.39 (dd, J = 11.6, 9.4 Hz, 1H), 2.88 – 2.80 (m, 1H), 2.69 – 2.63 (m, 1H), 2.61 – 2.42 (m, 1H), 2.44 (s, 3H), 2.20 (dd, J = 13.0, 3.9 Hz, 1H), 2.12 – 2.06 (m, 1H), 1.80 (td, J = 11.8, 7.3 Hz, 1H), 1.64 (s, 2H), 1.41 (t, J = 12.4 Hz, 1H). **^{13}C NMR** (151 MHz, Acetone-d_6) δ 147.32, 144.70, 144.22, 135.86, 130.56, 129.03, 128.97, 128.60, 128.16, 127.69, 126.52, 126.00, 102.71, 80.68, 60.14, 53.41, 53.00, 49.75, 41.40, 35.98, 30.03, 23.73, 21.39. **HRMS** (ESI): m/z Calcd. For $C_{29}H_{31}O_4SNa$ ([M+Na]$^+$): 512.1866, found: 512.1863.

(3a*R*, 5*R*, 9*S*, 9a*R*)-5, 9-diphenyl-2-tosyldodecahydro-5, 9-epoxyoxocino [4, 5-*c*] pyrrole (7v)

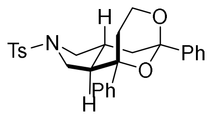

n=1, light yellow oil, 18.0 mg, $[\alpha]_D^{25}$ = + 17.89 (*c* 1.40, DCM), 60% yield, 50% ee, dr > 20:1, determined by HPLC (Chiralcel column AD-H, hexane/i-PrOH = 80/20, flow rate 1.0 mL/min, UV detection at 254 nm), t_{major} = 30.5min, t_{minor} = 24.1min. **^{1}H NMR** (400 MHz, Acetone-d_6) δ 7.72 (d, J = 8.1 Hz, 2H), 7.66 – 7.56 (m, 2H), 7.46 (d, J = 7.4 Hz, 2H), 7.44 – 7.35 (m, 6H), 7.35 – 7.20 (m, 2H), 3.79 – 3.71 (m, 1H), 3.71 – 3.61 (m, 2H), 3.31 – 3.15 (m, 2H), 2.90 (dd, J = 11.2, 9.3 Hz, 1H), 2.54 – 2.46 (m, 1H), 2.44 – 2.37 (m, 4H), 2.11 – 2.02 (m, 2H), 1.69 (td, J = 11.8, 7.4 Hz, 1H), 1.52 (t, J = 12.2 Hz, 1H). **^{13}C NMR** (151 MHz, Chloroform-*d*) δ 147.06, 143.67, 143.60, 134.92, 130.01, 128.69, 128.39, 128.36, 127.44, 127.38, 125.03, 124.00, 98.78, 75.78, 56.50, 52.84, 52.21, 48.81, 40.39, 35.09, 32.39, 21.77. **HRMS** (ESI): m/z Calcd. For $C_{28}H_{29}O_4SNa$ ([M+Na]$^+$): 498.1710, found: 498.1710.

(3a*R*, 4*S*, 7a*R*, 8a*R*)-4, 7-diphenyl-2-tosyldodecahydro-1*H*-4, 7-epoxyoxocino [4, 5-*c*] pyrrole (7w)

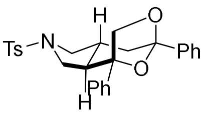

n=0, light yellow oil, 9.0 mg, $[\alpha]_D^{25}$ = + 32.27 (*c* 0.14, Acetone), 30% yield, 52% ee, dr > 20:1, determined by HPLC (Chiralcel column AD-H, hexane/i-PrOH = 50/50, flow rate 1.0 mL/min, UV detection at 254 nm), t_{major} = 24.3 min, t_{minor} = 30.6 min. **^{1}H NMR** (400 MHz, Chloroform-*d*) δ 7.68 (d, *J* = 8.0 Hz, 2H), 7.59 – 7.52 (m, 2H), 7.35 (dtd, *J* = 14.7, 8.3, 3.2 Hz, 8H), 7.23 – 7.17 (m, 2H), 4.36 (d, *J* = 7.3 Hz, 1H), 3.86 (d, *J* = 7.3 Hz, 1H), 3.68 (dd, *J* = 9.0, 6.4 Hz, 1H), 3.26 (dd, *J* = 9.1, 6.9 Hz, 1H), 3.05 (dd, *J* = 11.7, 9.1 Hz, 1H), 2.94 (dd, *J* = 11.0, 8.9 Hz, 1H), 2.45 (s, 3H), 2.35 – 2.20 (m, 2H), 2.05 (td, *J* = 11.8, 7.0 Hz, 1H), 1.90 – 1.78 (m, 1H). **^{13}C NMR** (151 MHz, Acetone-d_6) δ 144.35, 141.75, 140.86, 136.20, 130.70, 129.49, 129.29, 128.92, 128.82, 128.23, 126.27, 125.20, 109.30, 85.70, 71.67, 52.60, 52.46, 49.24, 39.55, 38.28, 21.49. **HRMS** (ESI): m/z Calcd. For $C_{27}H_{27}O_4SNa$ ([M+Na]$^+$): 484.1553, found: 484.1551.

(*E*)-1-phenyl-2-(7-phenyl-4-tosyl-3,4,5,8,9,10-hexahydro-2*H*-1,4-oxazecin-2-yl)-ethan-1-one (7u')

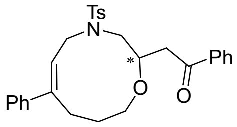

7u'

Light yellow oil, $[\alpha]_D^{25}$ = -10.72 (*c* 0.20, DCM), 6.3 mg, 21% yield, 24% ee, determined by HPLC (Chiralcel column AD-H, hexane/i-PrOH = 80/20, flow rate 1.0 mL/min, UV detection at 254 nm), t_{major} = 20.9min, t_{minor} = 23.0min. **^{1}H NMR** (400 MHz, Acetone-d_6) δ 8.01 (d, *J* = 7.0 Hz, 2H), 7.80 (d, *J* = 8.2 Hz, 2H), 7.64 (t, *J* = 7.5 Hz, 1H), 7.54 (t, *J* = 7.6 Hz, 2H), 7.43 (d, *J* = 8.0 Hz, 2H), 7.36 – 7.22 (m, 5H), 5.43 (dd, *J* = 11.6, 4.6 Hz, 1H), 4.42 – 4.34 (m, 1H), 4.23 (dd, *J* = 14.7, 4.7 Hz, 1H), 4.11 (dd, *J* = 6.9, 3.6 Hz, 1H), 3.75 (d, *J* = 14.7 Hz, 1H), 3.53 – 3.40 (m, 3H), 3.38 (d, *J* = 7.0 Hz, 1H), 3.32 – 3.26 (m, 1H), 3.19 (dd, *J* = 17.3, 5.4 Hz, 1H), 2.92 – 2.87 (m, 1H), 2.43 (s, 3H), 1.72 – 1.61 (m, 1H), 1.30 (d, *J* = 13.0 Hz, 1H). **^{13}C NMR** (151 MHz, Acetone-d_6) δ 197.88, 144.19, 143.72, 142.21, 138.29, 133.83, 130.71, 129.46, 129.29, 128.88, 128.38, 127.96, 127.20, 124.91, 76.48, 65.53, 47.37, 41.82, 25.98, 24.53, 21.40. **HRMS** (ESI): m/z Calcd. For $C_{29}H_{31}NO_4SNa$ ([M+Na]$^+$): 512.1866, found: 512.1869.

(*E*)-1-phenyl-2-(7-phenyl-4-tosyl-2,3,4,5,8,9-hexahydro-1,4-oxazonin-2-yl)ethan-1-one (7v')

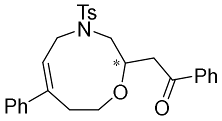

7v'

Light yellow oil, $[\alpha]_D^{25}$ = -8.48 (*c* 0.50, DCM), 4.8 mg, 16% yield, 44% ee, determined by HPLC (Chiralcel column AD-H, hexane/i-PrOH = 50/50, flow rate 0.7 mL/min, UV detection at 254 nm), t_{major} = 22.1min, t_{minor} = 25.0min. **^{1}H NMR** (600 MHz, Chloroform-*d*) δ 7.98 (d, *J* = 6.7 Hz, 2H), 7.71 (d, *J* = 8.2 Hz, 2H), 7.57 (t, *J* = 7.4 Hz, 1H), 7.47 (t, *J* = 7.7 Hz, 2H), 7.28 (dd, *J* = 12.1, 7.7 Hz, 5H), 7.15 (d, *J* = 6.6 Hz, 2H), 5.37 (dd, *J* = 10.9, 5.6 Hz, 1H), 4.42 (dd, *J* = 14.5, 5.6 Hz, 1H), 4.28 (dd, *J* = 14.4, 10.8 Hz, 1H), 4.24 (td, *J* = 5.0, 2.5 Hz, 1H), 4.07 – 4.04 (m, 1H), 3.44 – 3.35 (m, 4H), 3.19 (dd, *J* = 17.2, 5.2 Hz, 1H), 2.82 (dd, *J* = 15.2, 9.2 Hz, 1H), 2.66 (dd, *J* = 15.1, 5.8 Hz, 1H), 2.41 (s, 3H). **^{13}C NMR** (151 MHz, Chloroform-*d*) δ 197.97, 145.06, 143.45, 143.32, 137.42, 137.19, 133.31, 129.97, 128.72, 128.52, 128.37, 127.65, 127.16, 126.37, 122.75, 77.18, 69.16, 47.84, 47.78, 41.86, 32.99, 21.65.**HRMS** (ESI): m/z Calcd. For $C_{28}H_{29}NO_4SNa$ ($[M+Na]^+$): 498.1710, found: 498.1710.

(*S,Z*)-1-phenyl-2-(7-phenyl-4-tosyl-3,4,5,8-tetrahydro-2*H*-1,4-oxazocin-2-yl)-ethan-1-one (7w')

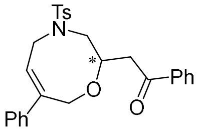

7w'

White solid, $[\alpha]_D^{25}$ = +0.48 (*c* 0.58, DCM), 11.4 mg, 38% yield, 23% ee, determined by HPLC (Chiralcel column AD-H, hexane/i-PrOH = 80/20, flow rate 1 mL/min, UV detection at 254 nm), t_{major} = 28.6min, t_{minor} = 33.4min.**^{1}H NMR** (600 MHz, Acetone-*d*$_6$) δ 8.04 (dd, *J* = 8.4, 1.3 Hz, 2H), 7.78 (d, *J* = 8.3 Hz, 2H), 7.69 – 7.59 (m, 1H), 7.58 – 7.50 (m, 2H), 7.40 (d, *J* = 7.6 Hz, 2H), 7.34 – 7.25 (m, 3H), 7.14 – 7.07 (m, 2H), 5.75 – 5.71 (m, 1H), 4.78 (dd, *J* = 17.3, 2.3 Hz, 1H), 4.48 (dt, *J* = 17.4, 1.4 Hz, 1H), 4.37 – 4.33 (m, 2H), 4.24 (dd, *J* = 14.6, 8.3 Hz, 1H), 3.80 (dd, *J* = 14.0, 2.2 Hz, 1H), 3.46 (dd, *J* = 17.2, 7.2 Hz, 1H), 3.30 (dd, *J* = 17.1, 5.3 Hz, 1H), 3.22 (dd, *J* = 13.9, 7.2 Hz, 1H), 2.43 (s, 3H). **^{13}C NMR** (151 MHz, Acetone-*d*$_6$) δ 198.03, 145.19, 144.13, 141.16, 138.41, 138.11, 134.00, 130.63, 129.52, 129.19, 128.96, 128.57, 128.11, 127.10, 122.50, 77.70, 72.53, 50.34, 44.65, 43.15, 21.40. **HRMS** (ESI): m/z Calcd. For $C_{27}H_{27}NO_4SNa$ ($[M+Na]^+$): 484.1553, found: 484.1554.

1-phenyl-3-(6-phenyl-3-tosyl-2,3,4,7-tetrahydro-1,3-oxazepin-2-yl)propan-1-one (7w”)

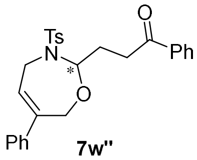

7w"

Light yellow oil, $[\alpha]_D^{25}$ = +2.59 (*c* 0.19, DCM), 5.7 mg, 19% yield, 34% ee, determined by HPLC (Chiralcel column AD-H, hexane/i-PrOH = 92/8, flow rate 1 mL/min, UV detection at 254 nm), t_{major} = 42.2min, t_{minor} = 44.5min. **^{1}H NMR** (600 MHz, Acetone-d_6) δ 8.02 – 7.98 (m, 2H), 7.78 (d, J = 8.3 Hz, 2H), 7.62 (t, J = 7.4 Hz, 1H), 7.53 (t, J = 7.7 Hz, 2H), 7.27 – 7.21 (m, 5H), 6.95 – 6.91 (m, 2H), 5.77 (dt, J = 6.7, 2.5 Hz, 1H), 5.39 (t, J = 6.3 Hz, 1H), 4.49 (d, J = 16.5 Hz, 1H), 4.36 (dd, J = 18.1, 6.6 Hz, 1H), 4.21 (d, J = 18.1 Hz, 1H), 3.90 (d, J = 16.5 Hz, 1H), 3.24 – 3.13 (m, 2H), 2.27 (s, 3H), 2.22 (dd, J = 13.5, 6.7 Hz, 1H), 2.12 – 2.07 (m, 1H). ^{13}C NMR (151 MHz, Acetone-d_6) δ 199.33, 144.27, 141.46, 139.76, 139.24, 138.14, 133.69, 130.32, 129.44, 128.98, 128.74, 128.34, 128.20, 126.61, 125.25, 90.07, 71.32, 41.26, 34.50, 21.33. **HRMS** (ESI): m/z Calcd. For $C_{27}H_{27}NO_4SNa$ ($[M+Na]^+$): 484.1553, found: 484.1557.

3. Supplementary Discussion

3.1. DFT calculation

3.1.1 Method

The density functional theory (DFT)[1] calculations were performed by using the *Gaussian 09* program[2]. The geometric structures of all involved transition states were optimized by using the B3LYP[3] density functional, combined with the 6-31G(d, p)[4-5] basis set. The solvent effects of dichloromethane were simulated by the SMD model[6]. The harmonic frequency calculations were conducted at the same level to corroborate each transition state has one and only one imaginary frequency and other structures have no imaginary frequency. Based on the optimized structures, all energies were refined by conducting single point energy calculations at the M06-2X/def2-TZVP/$SMD_{CH_2Cl_2}$[7-8] level of theory, with the empirical dispersion corrections using the original D3 damping function added[9-10].

3.1.2 Results for reaction mechanism

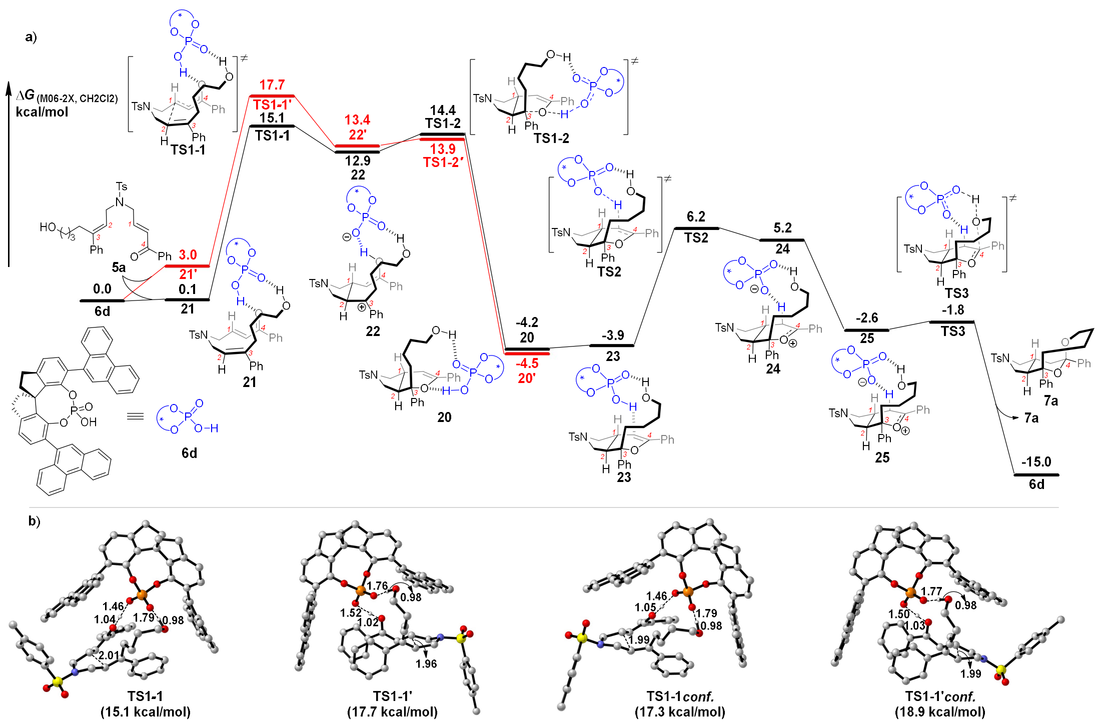

Supplementary Figure 1. Computational results for the cyclization mechanism of 5a into 7a. a) Gibbs free energy profile. Energy values are given in kcal/mol and represent the relative free energies calculated by the M06-2X method in

dichloromethane. The red line represents the pathway of formation of enantiomer of **7a**, and the black line indicates the pathway of formation of product **7a**. **b**) Optimized structures of two typical conformations of **TS1-1** and **TS1-1'**. The values of bond length are given in angstrom. **TS1-1*conf.*** represents the less stable conformer of **TS1-1** due to the different orientation of Ts group; **TS1-1'*conf.*** represents the less stable conformer of **TS1-1'** due to the different orientation of Ts group.

As shown in Supplementary Figure 1, the reaction is initiated by combination of substrate **5a** with catalyst **6d** through hydrogen bonds formation, followed by a stepwise formal HDA process *via* transition state **TS1-1** and **TS1-2** successively. Due to activation of the organophosphonic acid, the C_1-C_2 bond forms ahead of the C_3-O bond. The computational results indicate that the energy barrier *via* **TS1-1**, which is the transition state resulting from an attack of the *Si* face of *tri*-sustituted alkene onto the *Si* face of the conjugated enone alkene moiety, is 15.1 kcal/mol. The barrier *via* **TS1-1'** which corresponds to the '*Re*-*Re*' combination is 17.7 kcal/mol (*i.e.* 2.6 kcal/mol higher). For the following C_3-O bond formation, the energy barrier is 1.5 kcal/mol via **TS1-2** and 0.5 kcal/mol via **TS1-2'**, indicating a very fast process in both cases. Since the whole HDA process is significantly exothermic, the enantioselectivity should be determined by the C_1-C_2 bond formation step, and the relatively lower barrier *via* **TS1-1** suggests that the '*Si*-*Si*' combination should be more favor. This result is well consistent with the experimental results. In addition, for both **TS1-1** and **TS1-1'**, two typical conformations are considered, *i.e.* the *p*-methylphenyl of Ts orientated towards the phenanthrene group (**TS1-1** and **TS1-1'*conf.***) or that orientated away from the phenanthrene group (**TS1-1*conf.*** and **TS1-1'**). The computational results disclose that the relatively more stable conformation are **TS1-1**and **TS1-1'**, respectively.

The hydrogen bond transfer from PO-H···O in **20** to PO-H···π in **23** leads to slight rise of energy, and the energy barrier of the following protonation of the vinyl ether moiety is found to be 10.1 kcal/mol. The corresponding oxonium intermediate **24** is instable and will be quickly transformed into intermediate **25**, with heat released by 7.8 kcal/mol. Finally, a facile synergetic process of proton abstraction by the phosphate moiety and

attack of the hydroxyl group to the C_4 atom via **TS3** results in formation of ketal **7a** and delivers of catalyst **6d**.

3.1.3 Energies of all the optimized structures.

Supplementary Table 1. Absolute Energies, Thermal Corrections to Free Energies and Imaginary Frequency of the Transition State.

Geometry	*E*(elec-B3LYP)[1]	*G*(corr-B3LYP)[2]	*E*(elec-M06-2X)[3]	Imaginary Frequency[4]
**6d**	-2375.96718	0.56707	-2375.90863	
**5a**	-1916.89463	0.49333	-1916.82443	
**21**	-4292.88046	1.09089	-4292.76338	
**TS1-1**	-4292.85300	1.09491	-4292.74354	-343.3*i*
**TS1-1*conf.***	-4292.85371	1.09522	-4292.74033	-337.0*i*
**22**	-4292.85794	1.09823	-4292.75041	
**TS1-2**	-4292.85004	1.09518	-4292.74495	-201.2*i*
**20**	-4292.87344	1.10147	-4292.78074	
**21'**	-4292.87630	1.09260	-4292.76051	
**TS1-1'**	-4292.85351	1.09700	-4292.74152	-282.4*i*
**TS1-1'*conf.***	-4292.85032	1.09394	-4292.73654	-307.0*i*
**22'**	-4292.85650	1.09661	-4292.74798	
**TS1-2'**	-4292.84963	1.09528	-4292.74571	-185.0*i*
**20'**	-4292.87257	1.09845	-4292.77833	
**23**	-4292.87555	1.09681	-4292.77563	
**TS2**	-4292.86033	1.09304	-4292.75575	-1370.7*i*
**24**	-4292.87045	1.09715	-4292.76144	
**25**	-4292.87670	1.10132	-4292.77805	
**TS3**	-4292.87468	1.10220	-4292.77775	-155.0*i*
**7a**	-1916.92057	0.51193	-1916.86693	

[1]Electronic energy calculated by B3LYP in CH_2Cl_2as the solvent. [2]Thermal correction to Gibbs free energy calculated by B3LYP in CH_2Cl_2as the solvent. [3]Electronic energy calculated by M06-2X in CH_2Cl_2 as the solvent. [4]Imaginary frequency for each transition state calculated by B3LYP.

Supplementary Figures

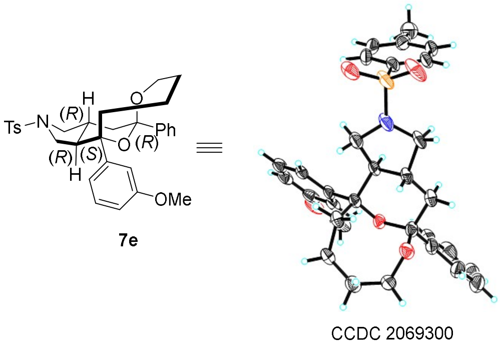

Supplementary Figure 2. **The Crystal Structure of 7e**. The structures of **7e** has been deposited under deposition number CDCC-2069300.

Supplementary Table 2. Crystal data and structure refinement for 7e.

Identification code	global
Empirical formula	C31 H37 N O6 S
Formula weight	551.67
Temperature	100(2) K
Wavelength	1.54178 Å
Crystal system	Trigonal
Space group	P3221
Unit cell dimensions	a = 14.0296(8) Å α = 90°<br>b = 14.0296(8) Å β = 90°<br>c = 28.5892(15)Å γ = 120°.
Volume	4873.3(6) $Å^3$
Z	6
Density (calculated)	1.128 Mg/m^3
Absorption coefficient	1.204 mm^{-1}
F(000)	1764
Crystal size	0.230 x 0.140 x 0.130 mm^3
Theta range for data collection	3.64 to 72.48°.
Index ranges	-17<=h<=17, -17<=k<=17, -34<=l<=35
Reflections collected	75898
Independent reflections	5520 [R(int) = 0.0733]
Completeness to theta = 72.48°	86.0 %
Absorption correction	Semi-empirical from equivalents
Max. and min. transmission	0.86 and 0.68
Refinement method	Full-matrix least-squares on F^2
Data / restraints / parameters	5520 / 378 / 356
Goodness-of-fit on F^2	1.397
Final R indices [I>2sigma(I)]	R1 = 0.1079, wR2 = 0.3059
R indices (all data)	R1 = 0.1257, wR2 = 0.3190
Absolute structure parameter	0.065(8)
Extinction coefficient	0.0010(6)
Largest diff. peak and hole	0.931 and -0.373 e.$Å^{-3}$

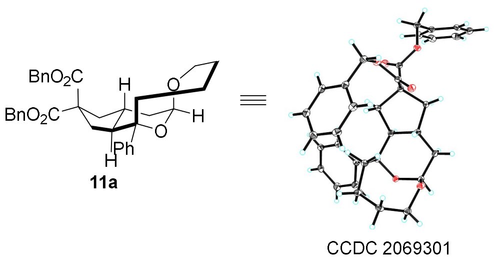

Supplementary Figure 3. **The Crystal Structure of 11a**. The structures of **11a** has been deposited under deposition number CDCC-2069301.

Supplementary Table 3. **Crystal data and structure refinement for 11a**.

Identification code	global
Empirical formula	C34 H36 O6
Formula weight	540.63
Temperature	100(2) K
Wavelength	1.54178 Å
Crystal system	Orthorhombic
Space group	$P2_12_12_1$
Unit cell dimensions	a = 8.4909(3) Å α = 90°<br>b = 10.2366(3)Å β = 90°<br>c = 31.1212(10)Å γ = 90°
Volume	2704.99(15) Å^3
Z	4
Density (calculated)	1.328 Mg/m^3
Absorption coefficient	0.725 mm^{-1}
F(000)	1152
Crystal size	0.230 x 0.140 x 0.140 mm^3
Theta range for data collection	2.84 to 72.45°.
Index ranges	-10<=h<=10, -12<=k<=12, -38<=l<=36
Reflections collected	21094
Independent reflections	5263 [R(int) = 0.2066]
Completeness to theta = 72.45°	98.8 %
Absorption correction	Semi-empirical from equivalents
Max. and min. transmission	0.91 and 0.64
Refinement method	Full-matrix least-squares on F^2
Data / restraints / parameters	5263 / 0 / 361
Goodness-of-fit on F^2	1.108
Final R indices [I>2sigma(I)]	R1 = 0.0574, wR2 = 0.1415
R indices (all data)	R1 = 0.0857, wR2 = 0.1574
Absolute structure parameter	-0.07(14)
Largest diff. peak and hole	0.508 and -0.491 e.Å^{-3}

HPLC Chromatograms

Supplementary Figure 4. HPLC Trace of 7a.

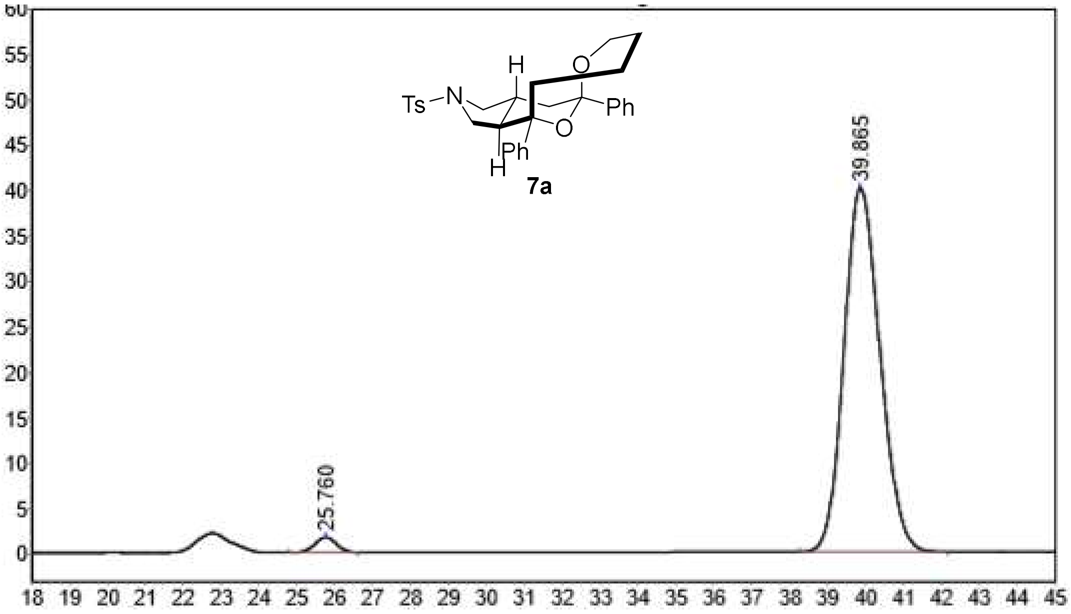

#	RT (time)	Heigh (mV*sec)	Area (mv)	Area (%)
1	25.760	1636.297	65535.344	2.4129
2	39.865	40019.262	2650478.750	97.5871

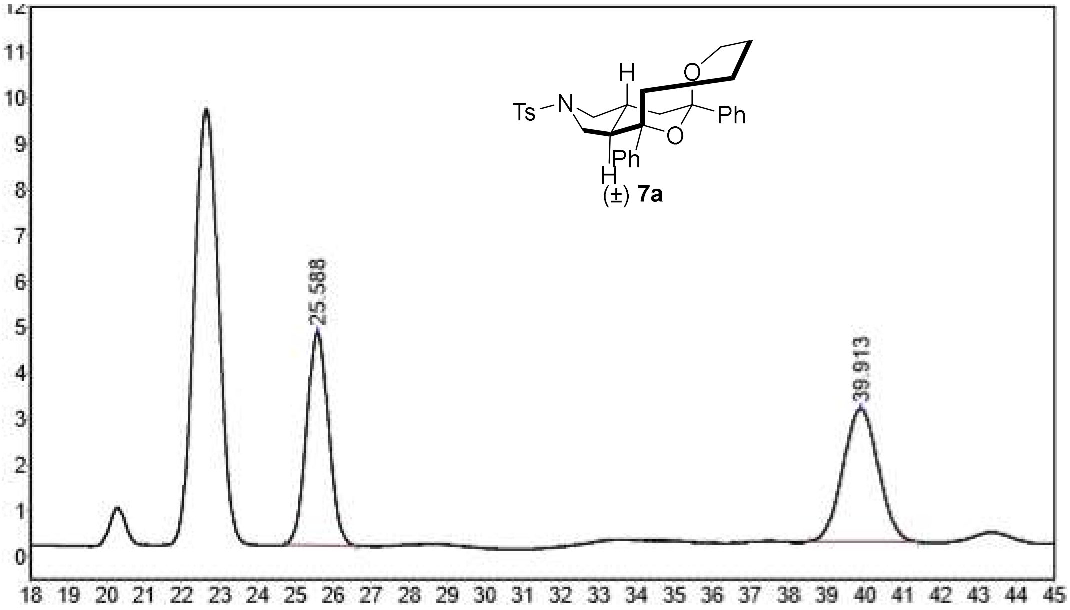

#	RT (time)	Heigh (mV*sec)	Area (mv)	Area (%)
1	25.588	4644.963	187092.500	49.5642
2	39.913	2914.000	190382.844	50.4358

Supplementary Figure 5. HPLC Trace of 7b.

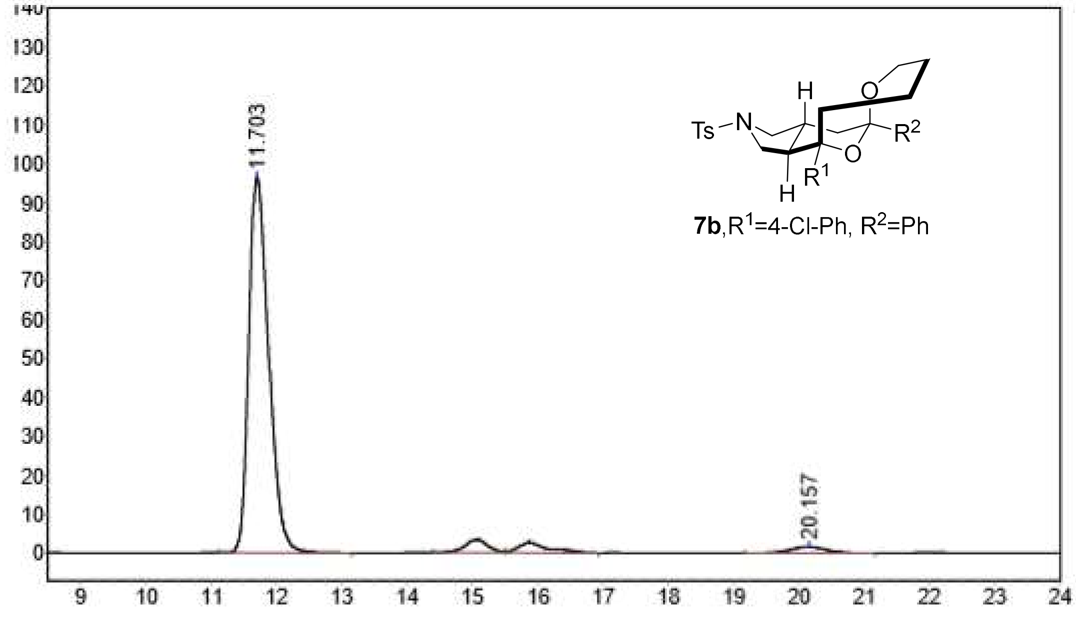

#	RT (time)	Heigh (mV*sec)	Area (mv)	Area (%)
1	11.703	96046.492	2052805.125	89.1746
2	20.157	1670.967	64793.938	2.8147

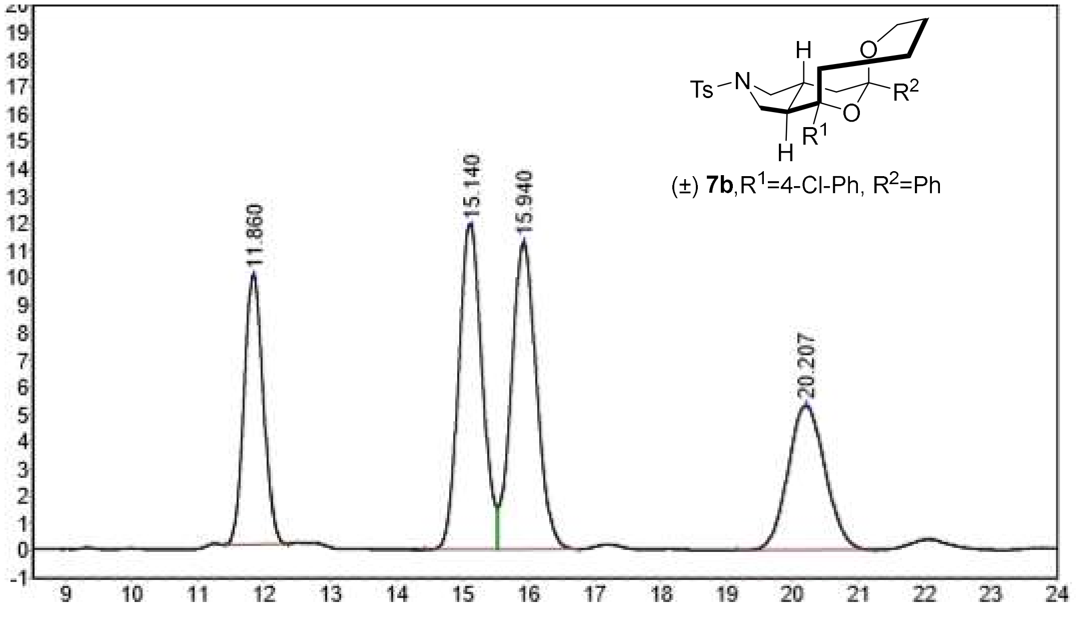

#	RT (time)	Heigh (mV*sec)	Area (mv)	Area (%)
1	11.860	10065.637	209532.656	20.7195
2	15.140	11918.038	295513.719	29.2217
3	15.940	11266.302	299506.563	29.6166
4	20.207	5290.359	206727.891	20.4422

Supplementary Figure 6. HPLC Trace of 7c.

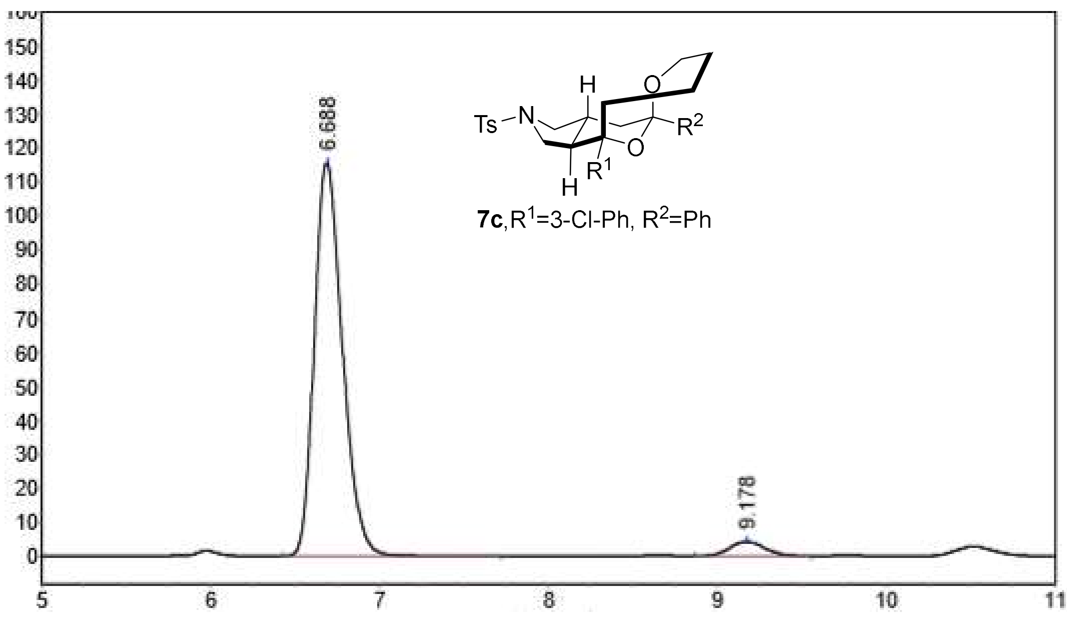

(min)

#	RT (time)	Heigh (mV*sec)	Area (mv)	Area (%)
1	6.688	115318.664	1335629.750	95.4497
2	9.178	4232.250	63672.711	4.5503

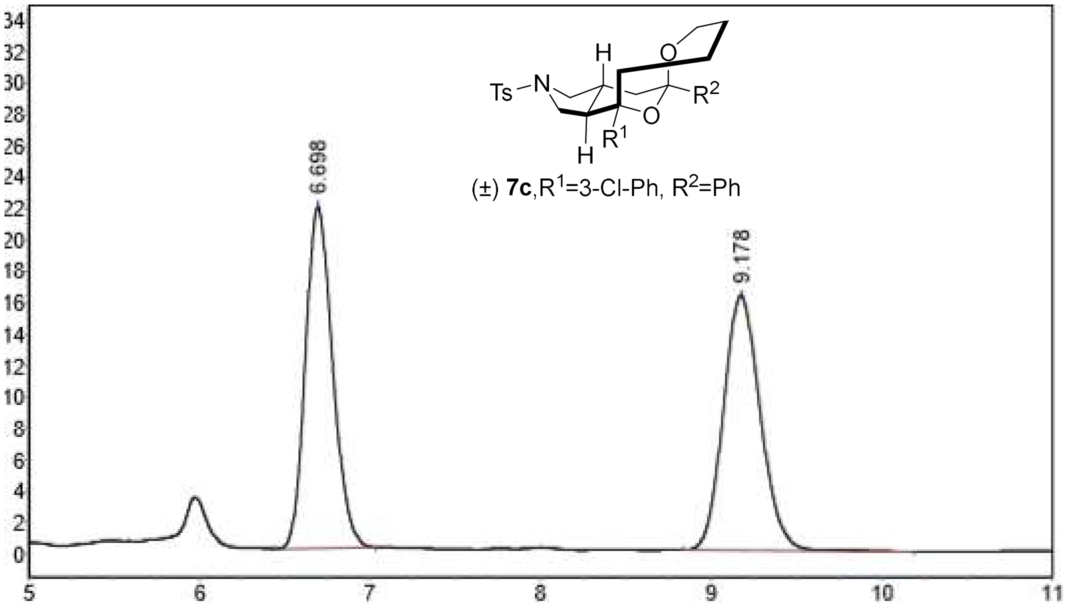

(min)

#	RT (time)	Heigh (mV*sec)	Area (mv)	Area (%)
1	6.698	21762.145	241419.156	49.3536
2	9.178	16254.285	247742.750	50.6464

Supplementary Figure 7. HPLC Trace of 7d.

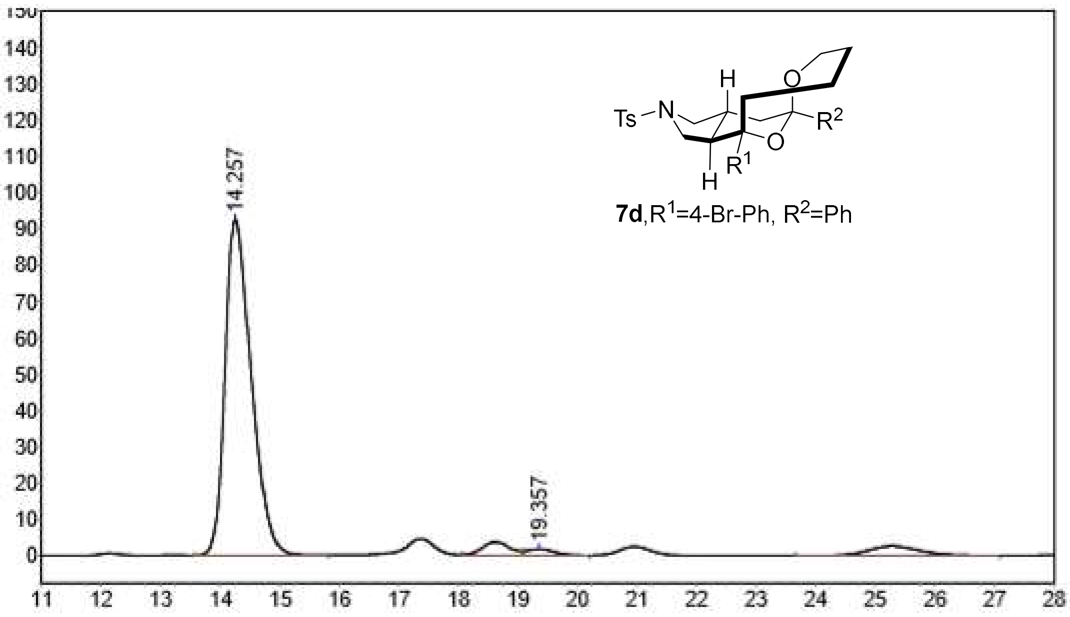

#	RT (time)	Heigh (mV*sec)	Area (mv)	Area (%)
1	14.257	92395.414	2831128.000	89.1253
2	19.357	1822.757	59178.617	1.8630

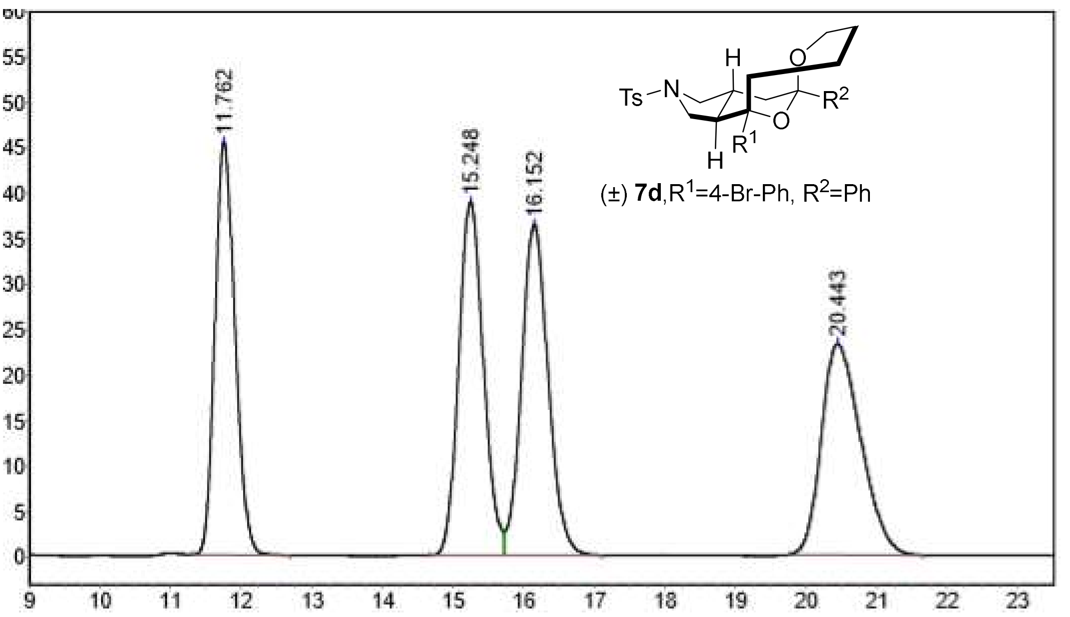

#	RT (time)	Heigh (mV*sec)	Area (mv)	Area (%)
1	11.762	45415.203	901936.938	24.2796
2	15.248	38907.980	947118.313	25.4959
3	16.152	36401.418	958843.063	25.8115
4	20.443	23272.688	906888.188	24.4129

Supplementary Figure 8. HPLC Trace of 7e.

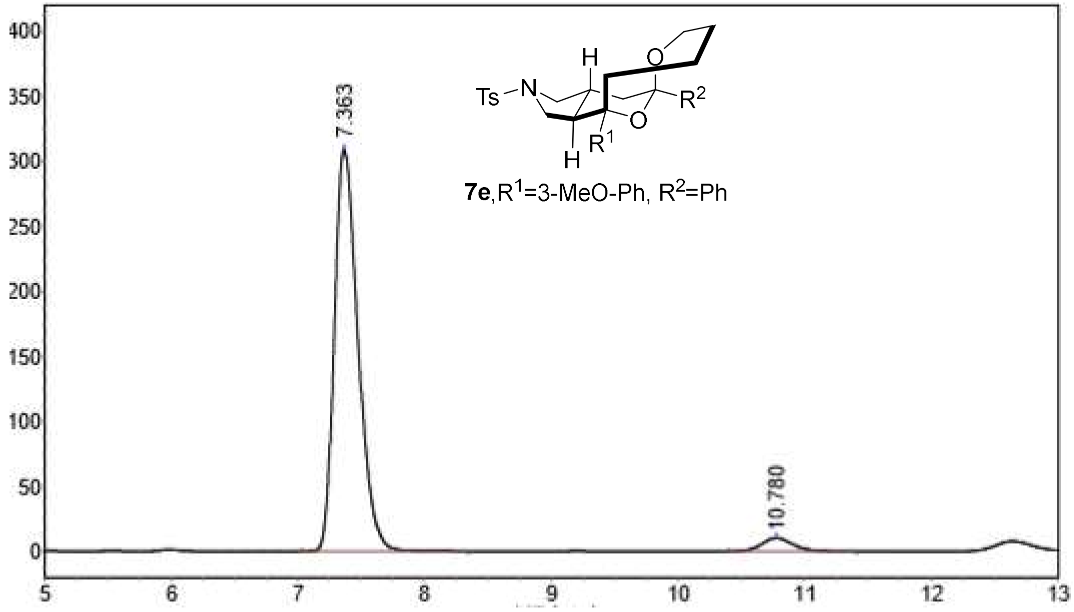

#	RT (time)	Heigh (mV*sec)	Area (mv)	Area (%)
1	7.363	308592.250	4016179.000	95.7605
2	10.780	10017.296	177805.406	4.2395

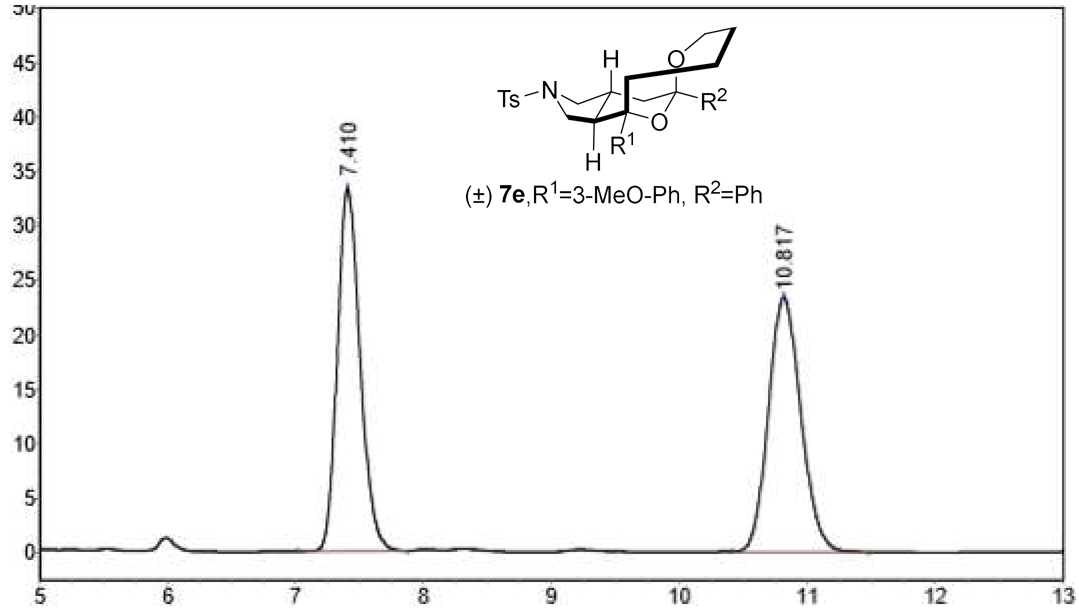

#	RT (time)	Heigh (mV*sec)	Area (mv)	Area (%)
1	7.410	33238.711	412479.688	49.8627
2	10.817	23347.115	414750.875	50.1373

Supplementary Figure 9. HPLC Trace of 7f.

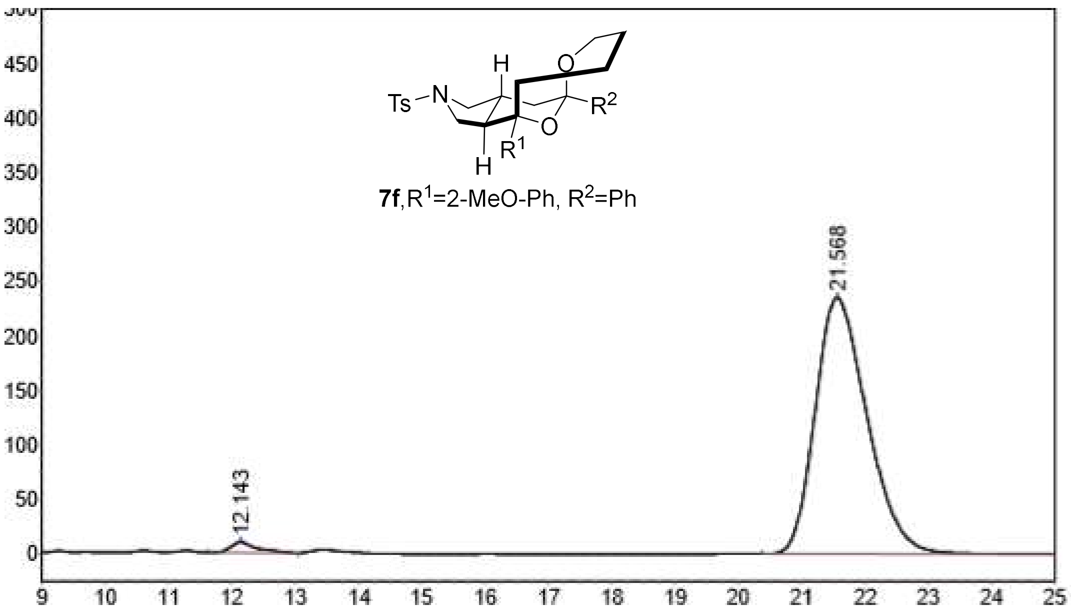

#	RT (time)	Heigh (mV*sec)	Area (mv)	Area (%)
1	12.143	9568.901	289181.656	2.1083
2	21.568	234950.766	13427056.000	97.8917

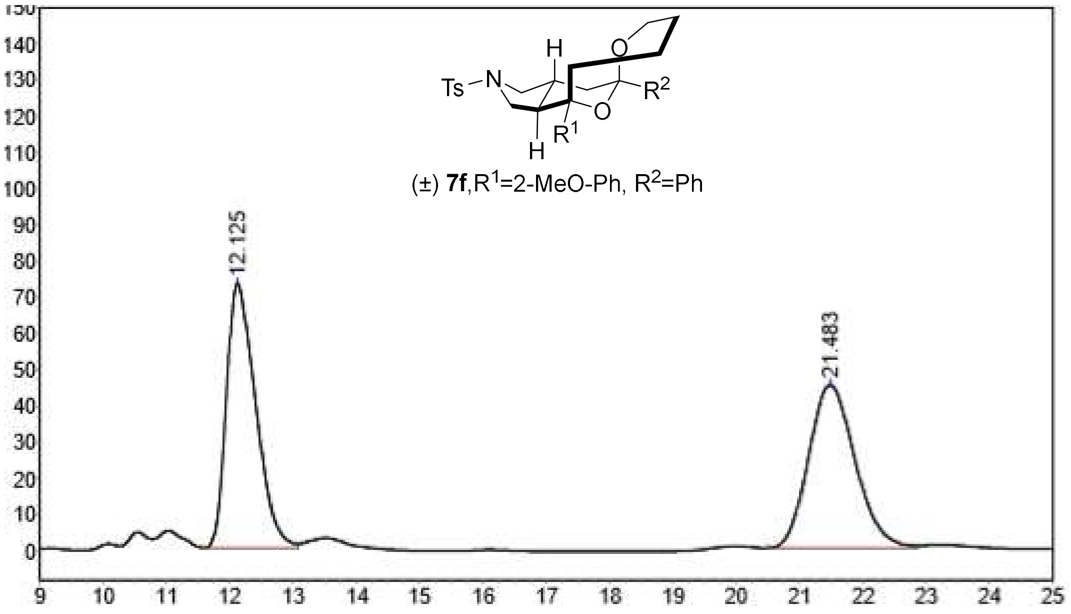

#	RT (time)	Heigh (mV*sec)	Area (mv)	Area (%)
1	12.125	72850.000	2340052.500	50.4413
2	21.483	45102.000	2299106.750	49.5587

Supplementary Figure 10. HPLC Trace of 7g.

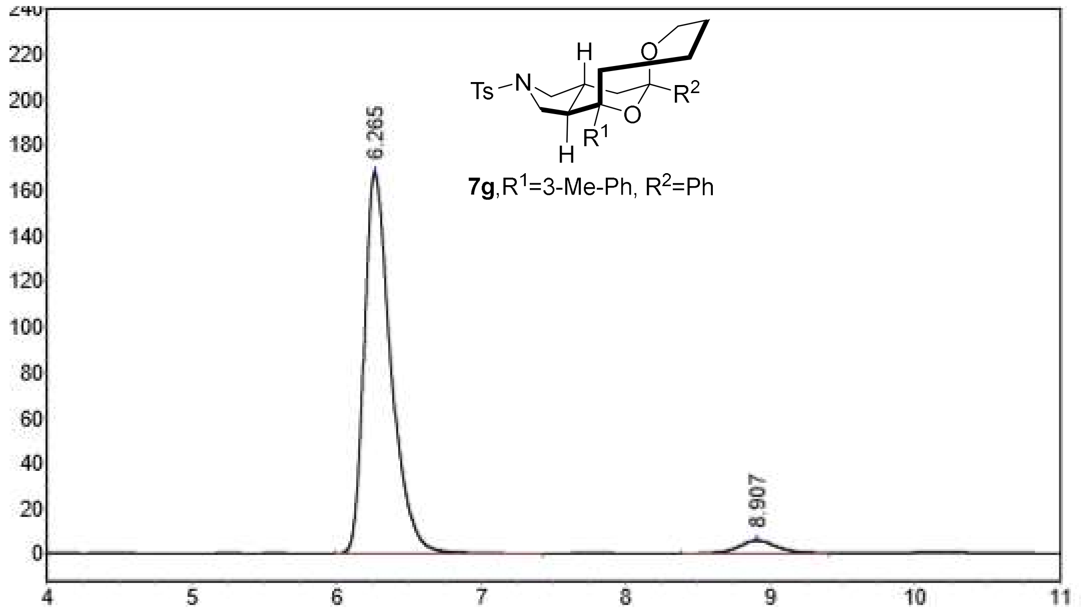

#	RT (time)	Heigh (mV*sec)	Area (mv)	Area (%)
1	6.265	167922.094	2160883.000	95.0182
2	8.907	5710.000	113294.500	4.9818

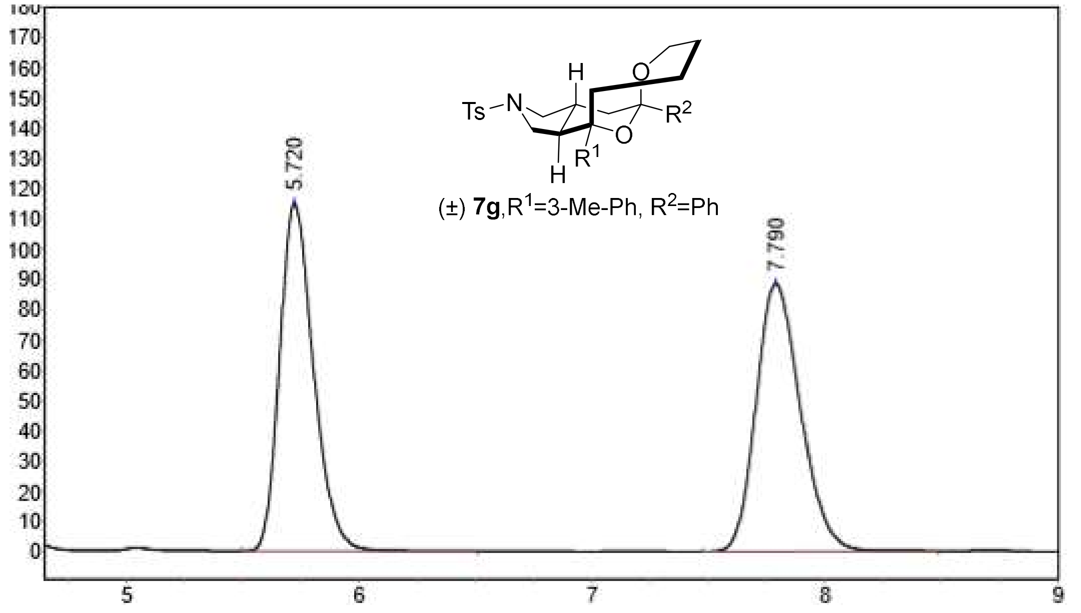

#	RT (time)	Heigh (mV*sec)	Area (mv)	Area (%)
1	5.720	114898.984	1159603.625	49.7582
2	7.790	88413.023	1170875.250	50.2418

Supplementary Figure 11. HPLC Trace of 7h.

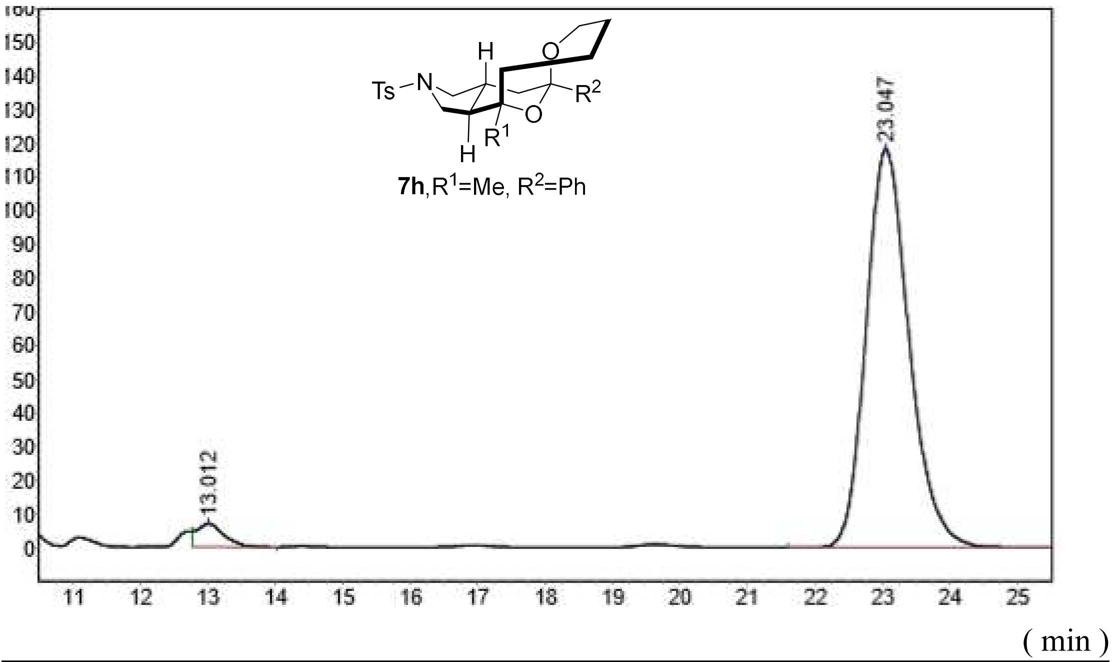

#	RT (time)	Heigh (mV*sec)	Area (mv)	Area (%)
1	13.012	6728.737	190452.000	3.5097
2	23.047	118178.352	5236017.500	96.4903

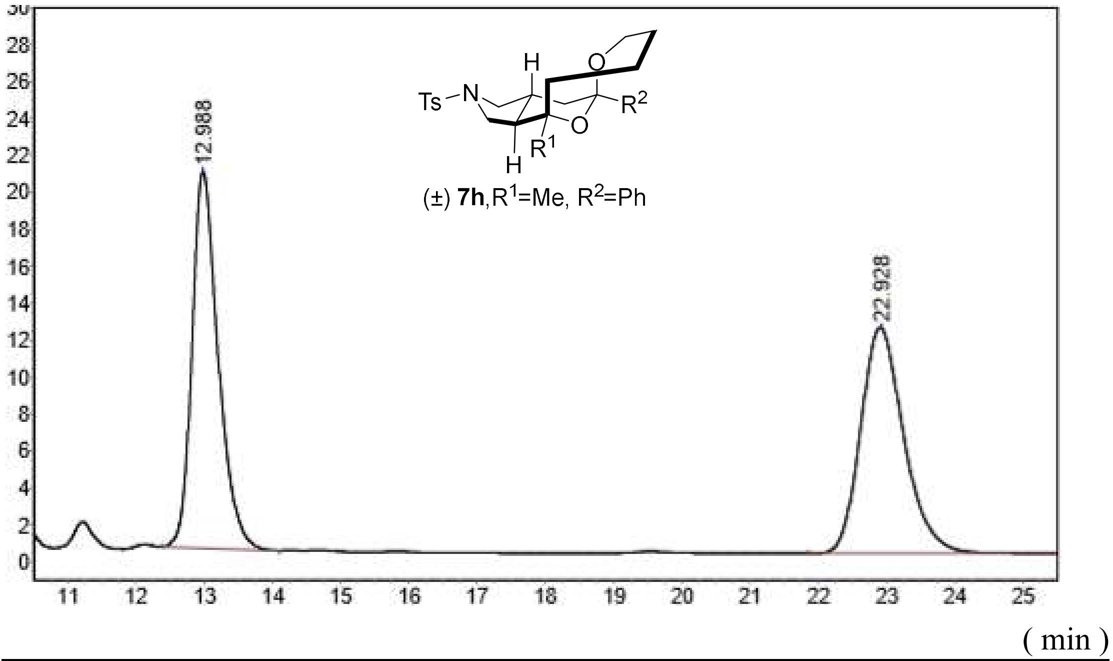

#	RT (time)	Heigh (mV*sec)	Area (mv)	Area (%)
1	12.988	20417.750	548484.813	50.4194
2	22.928	12225.031	539359.188	49.5806

Supplementary Figure 12. HPLC Trace of 7i.

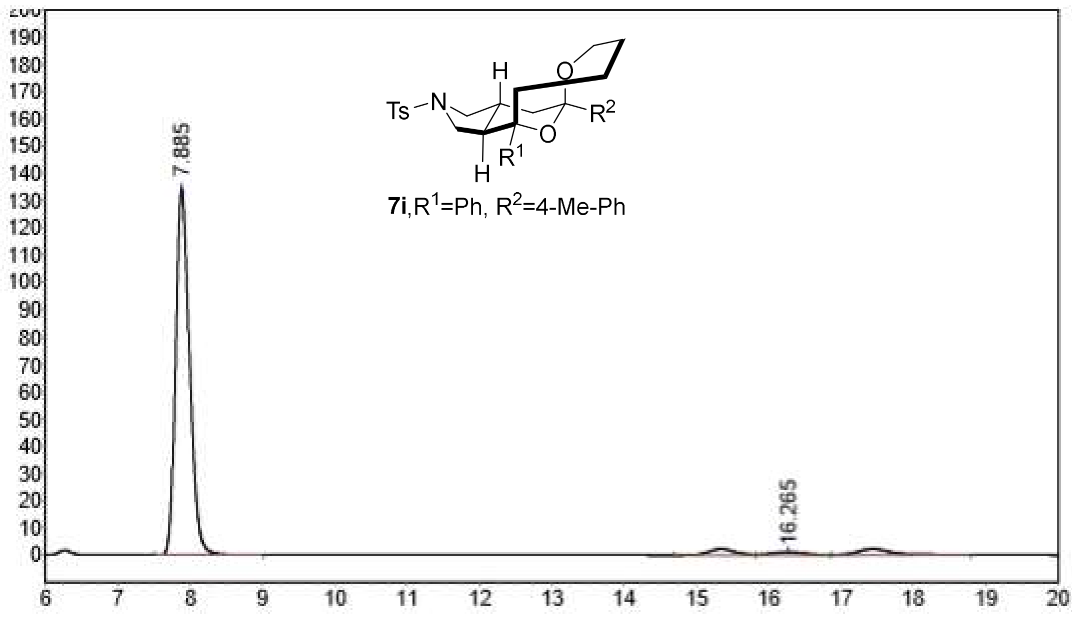

#	RT (time)	Heigh (mV*sec)	Area (mv)	Area (%)
1	7.885	134038.578	1805328.625	91.1656
2	16.265	1225.496	38438.988	1.9411

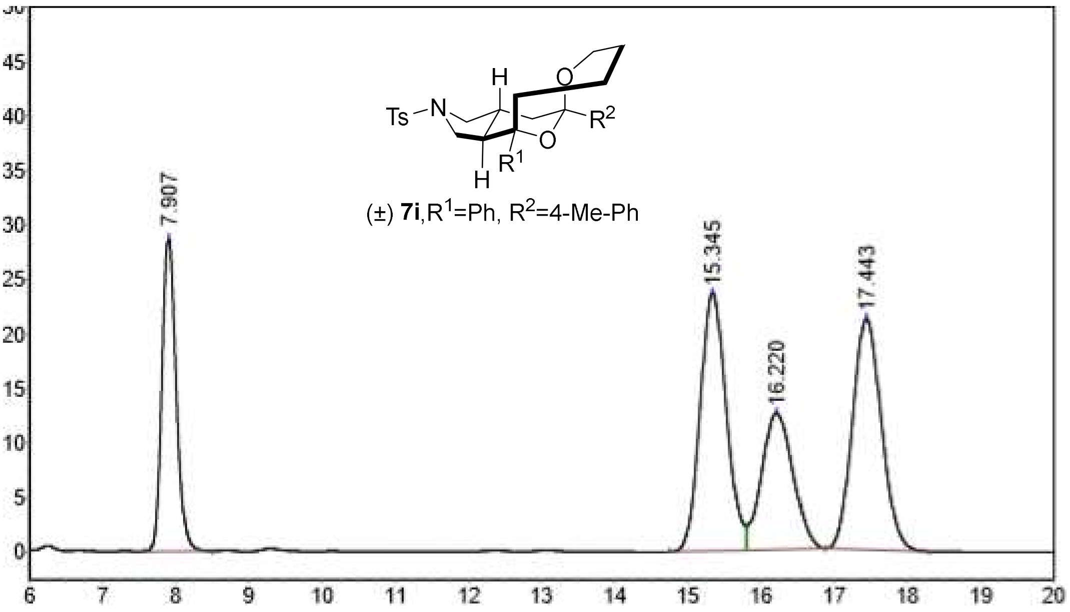

#	RT (time)	Heigh (mV*sec)	Area (mv)	Area (%)
1	7.907	28643.492	385873.594	19.9762
2	15.345	23676.375	589674.250	30.5266
3	16.220	12506.903	372843.469	19.3016
4	17.443	21183.756	583279.875	30.1956

Supplementary Figure 13. HPLC Trace of 7j.

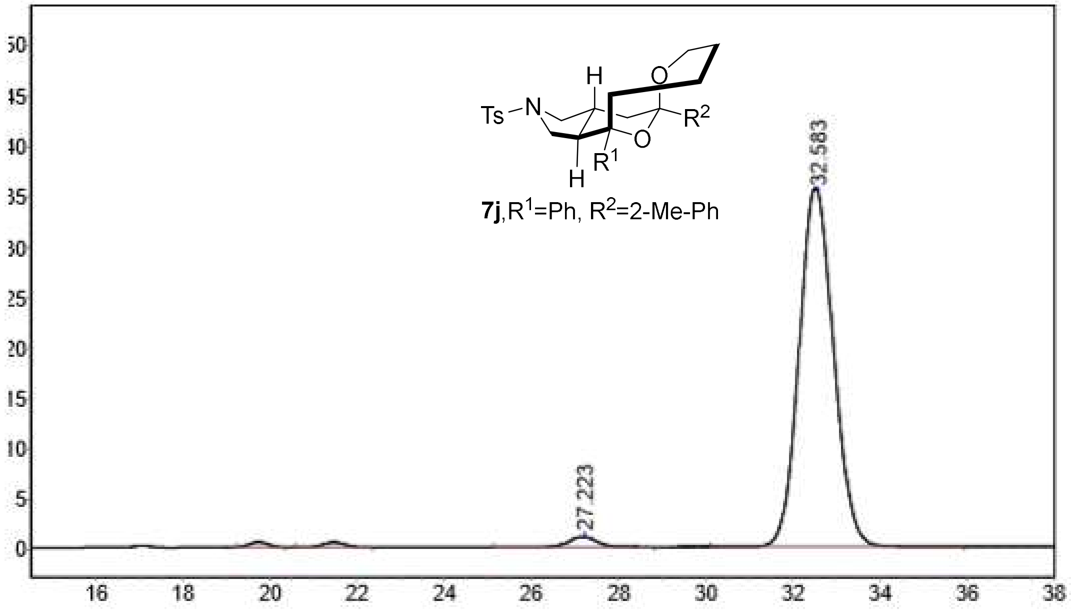

#	RT (time)	Heigh (mV*sec)	Area (mv)	Area (%)
1	27.223	986.097	44681.094	2.1952
2	32.583	35444.438	1956021.000	96.0984

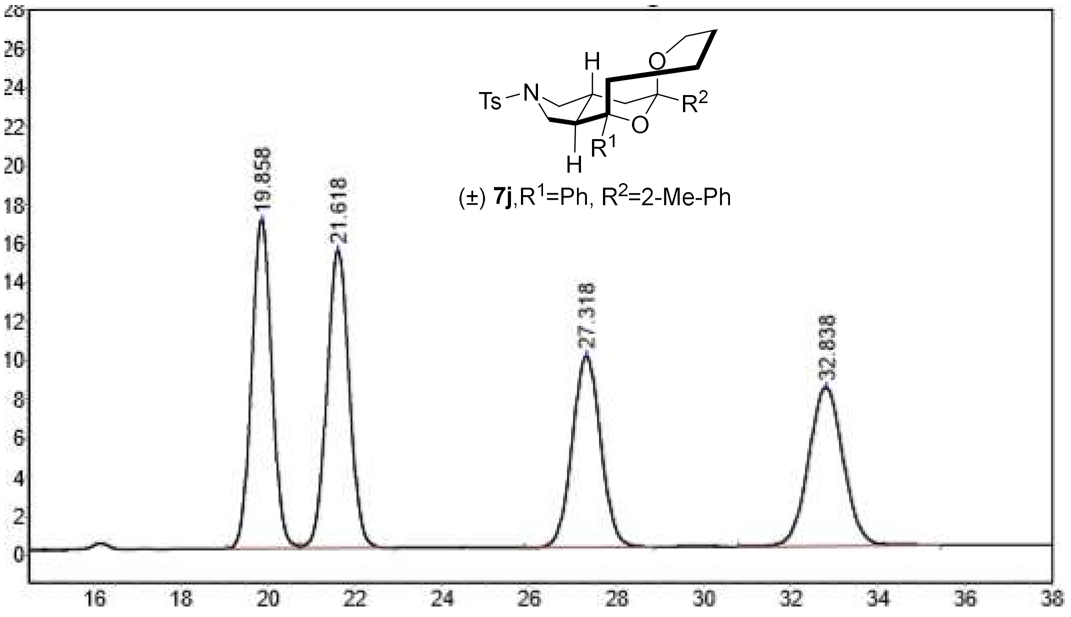

#	RT (time)	Heigh (mV*sec)	Area (mv)	Area (%)
1	19.858	16812.535	540792.063	27.2727
2	21.618	15249.796	544437.938	27.4565
3	27.318	9796.811	445180.406	22.4509
4	32.838	8124.336	452498.813	22.8199

Supplementary Figure 14. HPLC Trace of 7k.

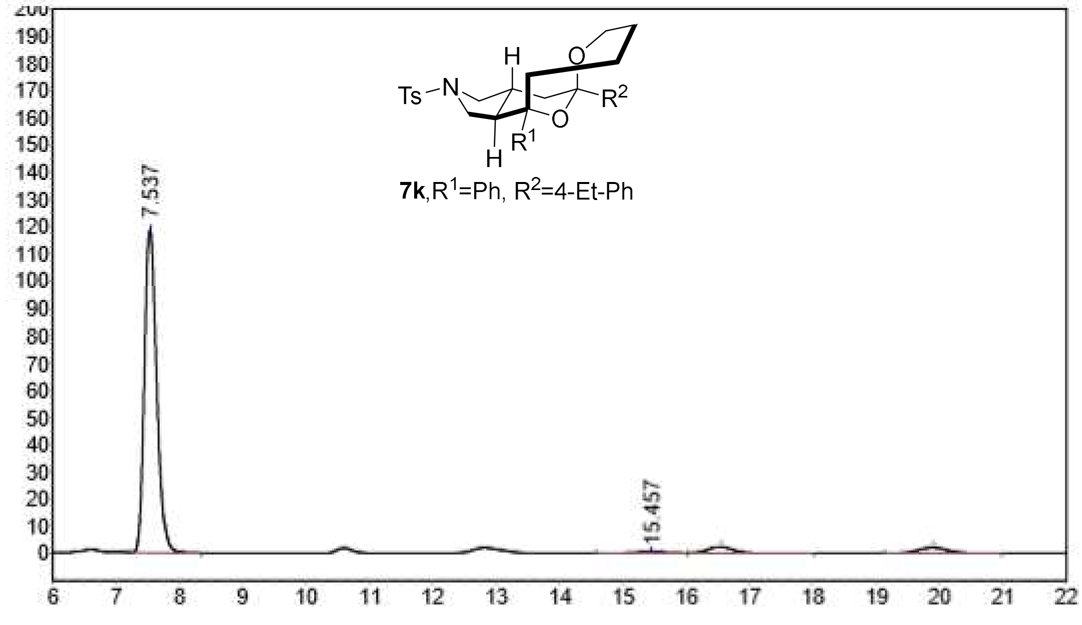

#	RT (time)	Heigh (mV*sec)	Area (mv)	Area (%)
1	7.537	118422.320	1589707.125	90.6637
2	15.457	560.319	20744.352	1.1831

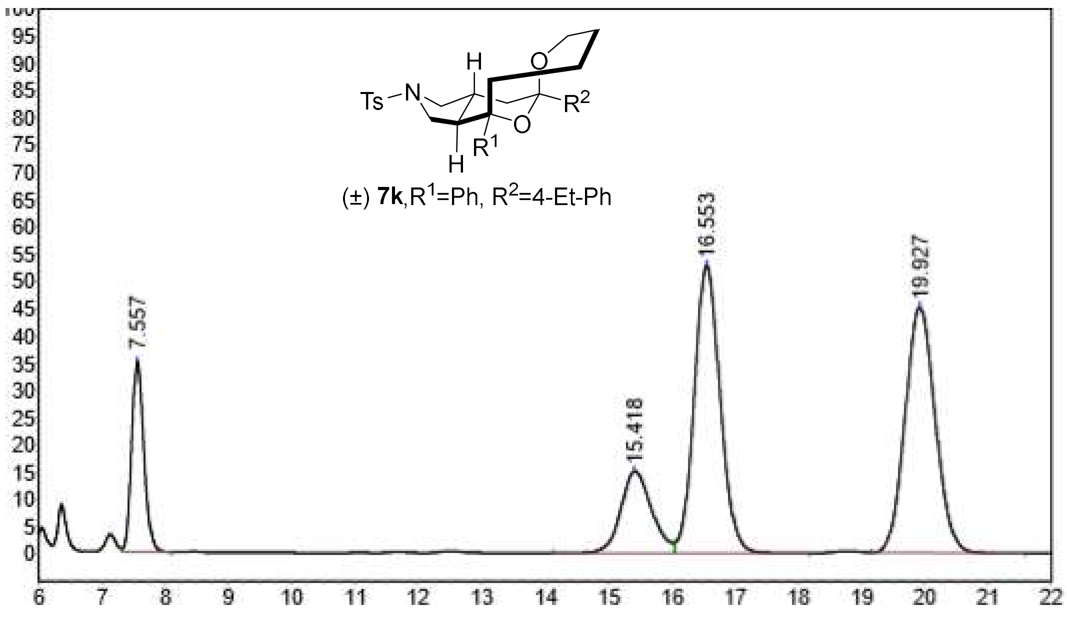

#	RT (time)	Heigh (mV*sec)	Area (mv)	Area (%)
1	7.557	34768.000	469493.313	11.7795
2	15.418	15059.796	512669.063	12.8628
3	16.553	52777.164	1506597.375	37.8002
4	19.927	45018.598	1496922.500	37.5575

Supplementary Figure 15. HPLC Trace of 7l.

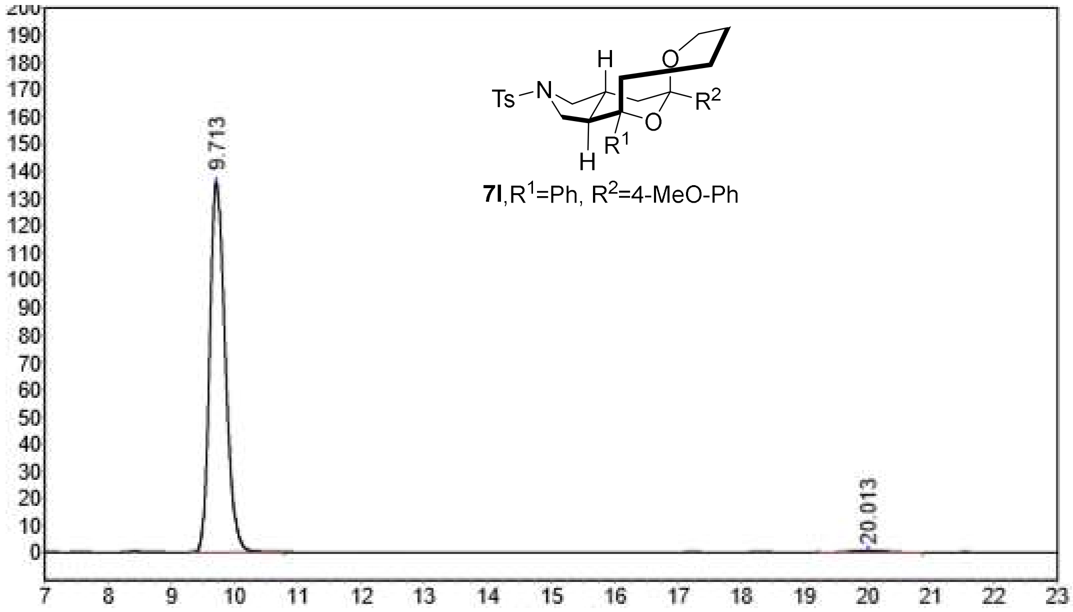

#	RT (time)	Heigh (mV*sec)	Area (mv)	Area (%)
1	9.713	135770.156	2332171.250	98.8725
2	20.013	705.008	26595.355	1.1275

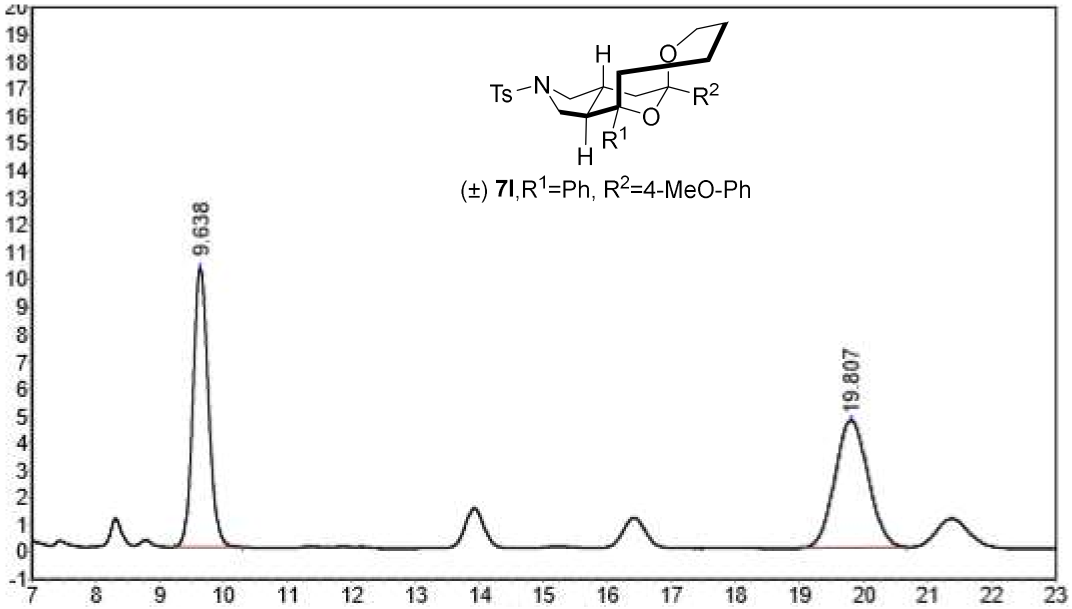

#	RT (time)	Heigh (mV*sec)	Area (mv)	Area (%)
1	9.638	10220.459	171167.500	50.3247
2	19.807	4677.254	168958.406	49.6753

Supplementary Figure 16. HPLC Trace of 7m.

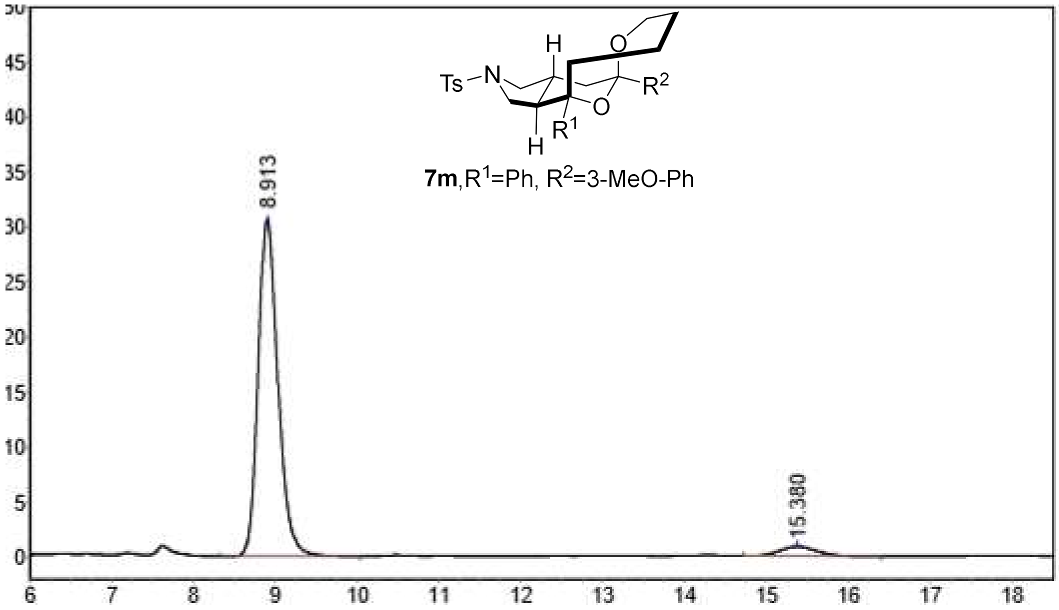

(min)

#	RT (time)	Heigh (mV*sec)	Area (mv)	Area (%)
1	8.913	30554.063	518356.688	94.4985
2	15.380	914.000	30177.801	5.5015

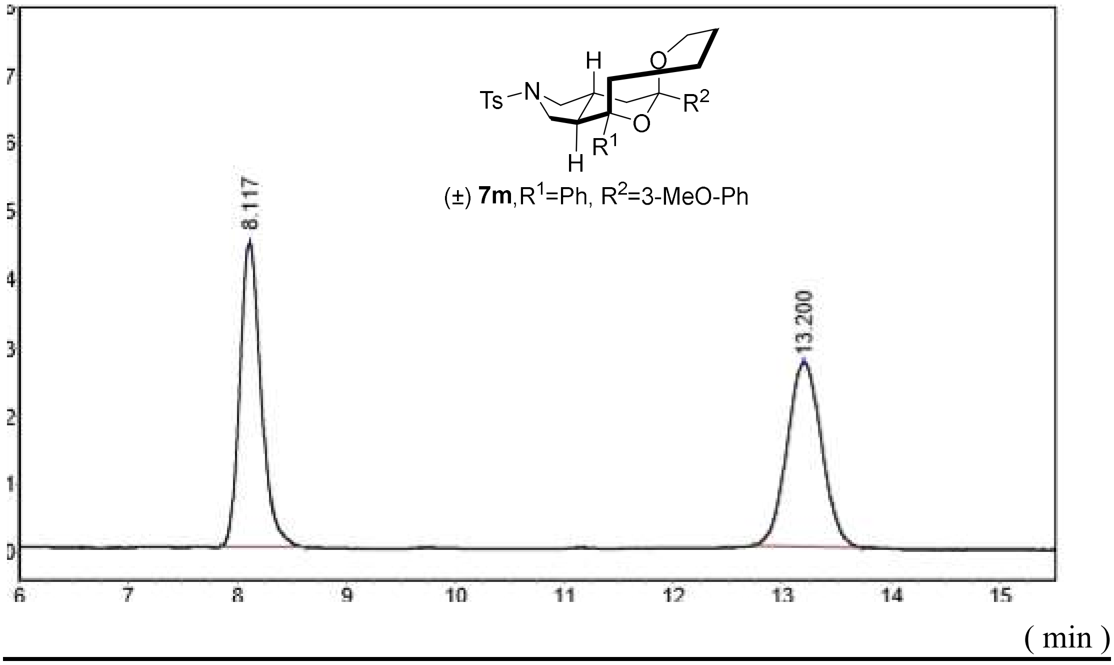

(min)

#	RT (time)	Heigh (mV*sec)	Area (mv)	Area (%)
1	8.117	4455.653	60278.301	50.8056
2	13.200	2687.010	58366.801	49.1944

Supplementary Figure 17. HPLC Trace of 7n.

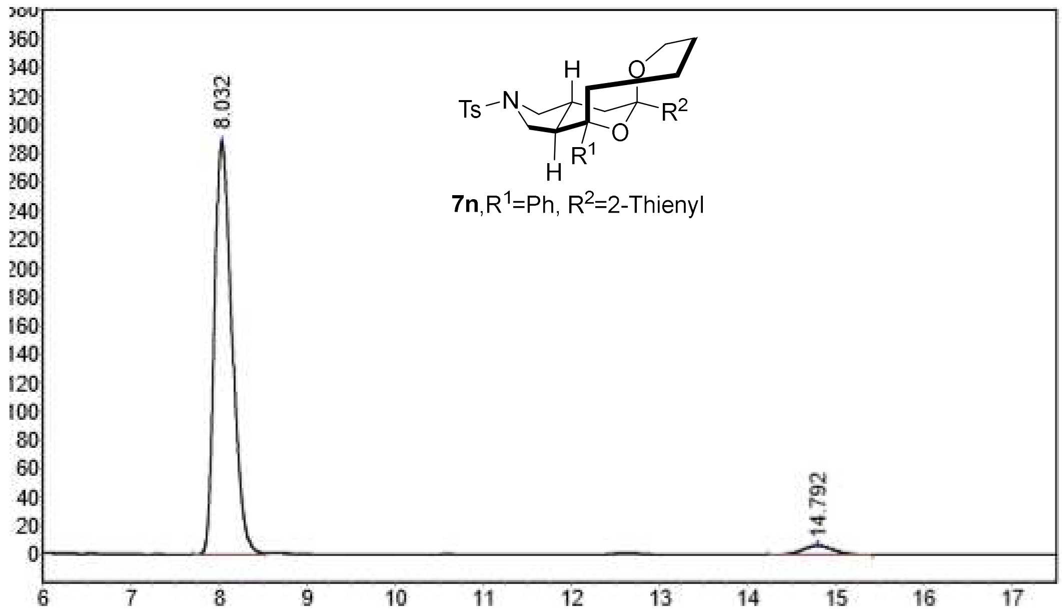

(min)

#	RT (time)	Heigh (mV*sec)	Area (mv)	Area (%)
1	8.032	287633.000	4009881.250	96.2773
2	14.792	6616.255	155048.156	3.7227

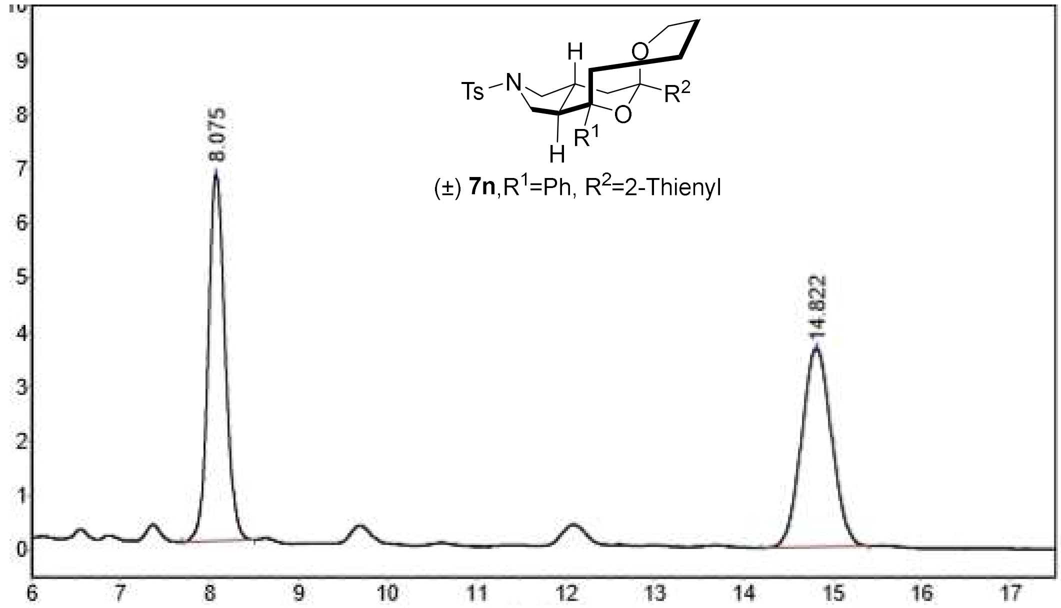

(min)

#	RT (time)	Heigh (mV*sec)	Area (mv)	Area (%)
1	8.075	6737.364	89893.734	50.7718
2	14.822	3656.287	87160.617	49.2282

Supplementary Figure 18. HPLC Trace of 7o.

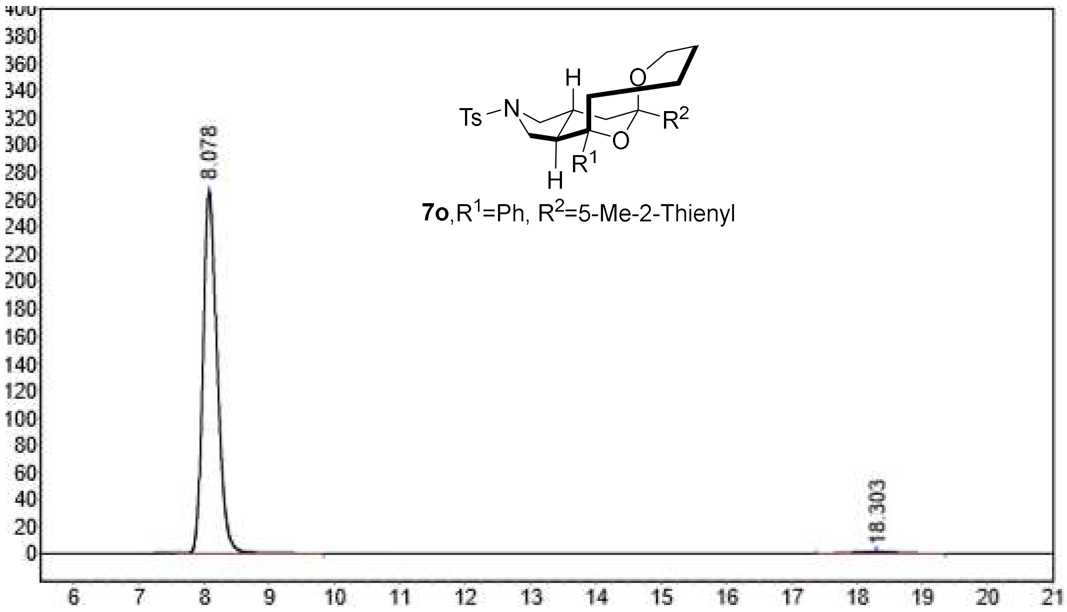

(min)

#	RT (time)	Heigh (mV*sec)	Area (mv)	Area (%)
1	8.078	264882.750	4035793.500	98.7493
2	18.303	1276.406	51117.059	1.2508

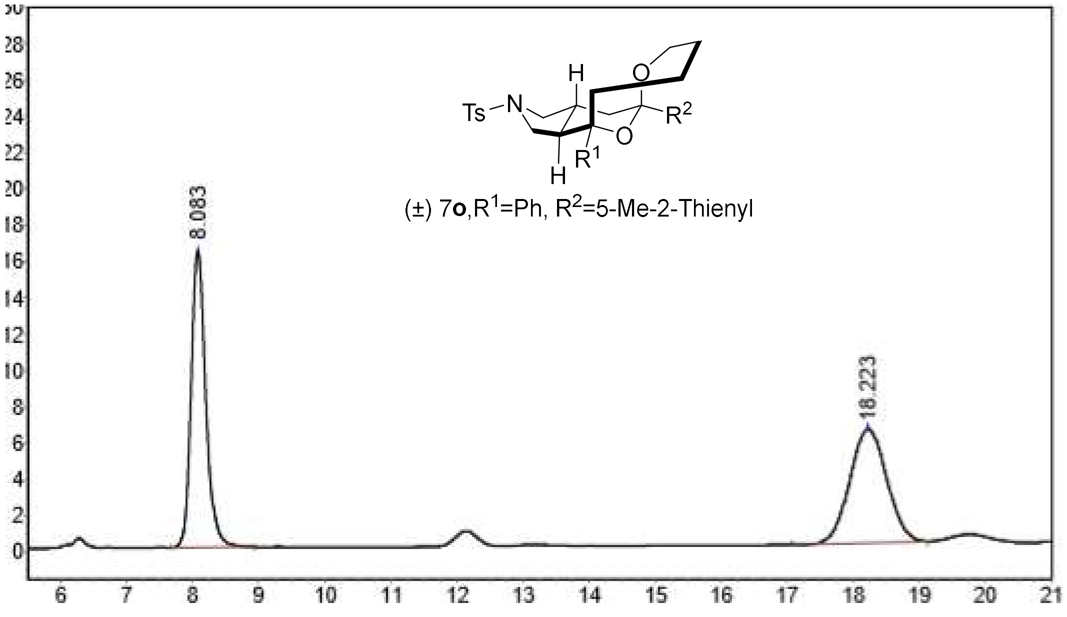

(min)

#	RT (time)	Heigh (mV*sec)	Area (mv)	Area (%)
1	8.083	16273.510	250968.297	50.6415
2	18.223	6282.927	244610.203	49.3585

Supplementary Figure 19. HPLC Trace of 7p.

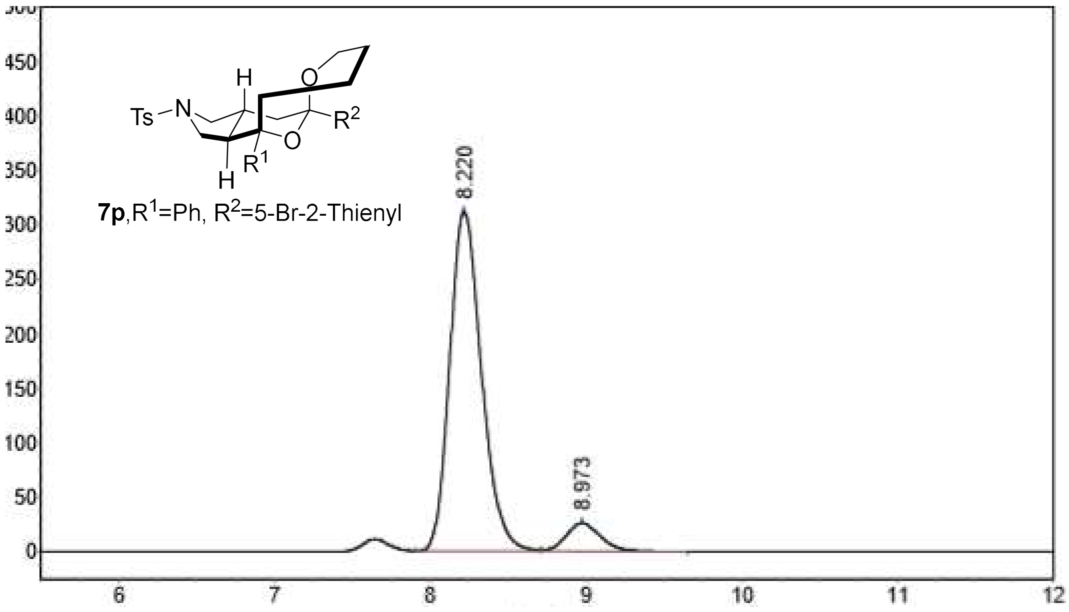

#	RT (time)	Heigh (mV*sec)	Area (mv)	Area (%)
1	8.220	311540.844	4438612.500	91.5930
2	8.973	25884.406	407404.625	8.4070

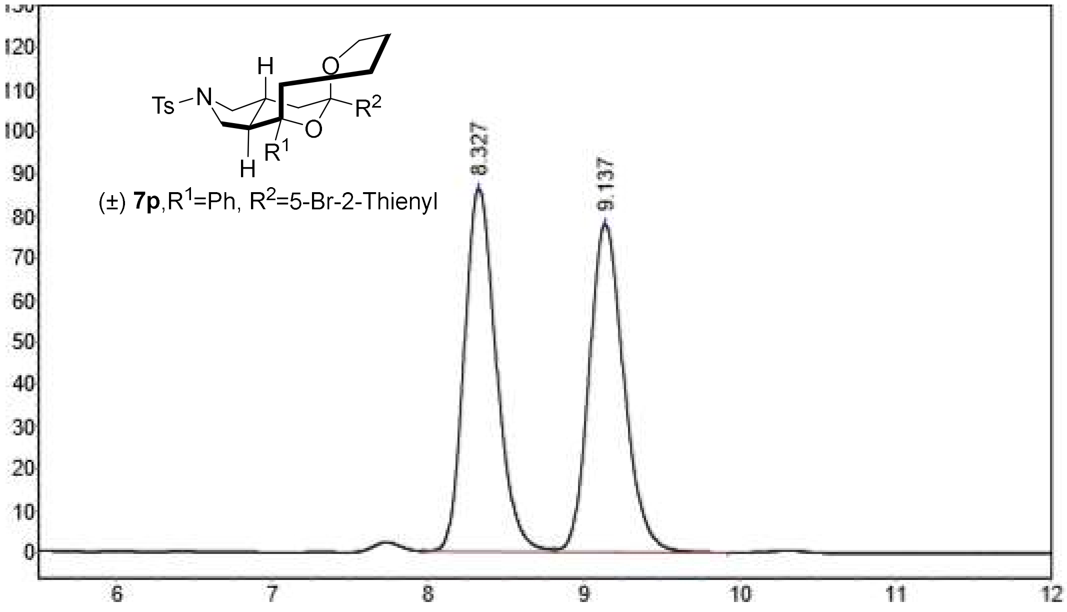

#	RT (time)	Heigh (mV*sec)	Area (mv)	Area (%)
1	8.327	86244.078	1233742.875	50.4711
2	9.137	77864.625	1210713.750	49.5290

Supplementary Figure 20. HPLC Trace of 7q.

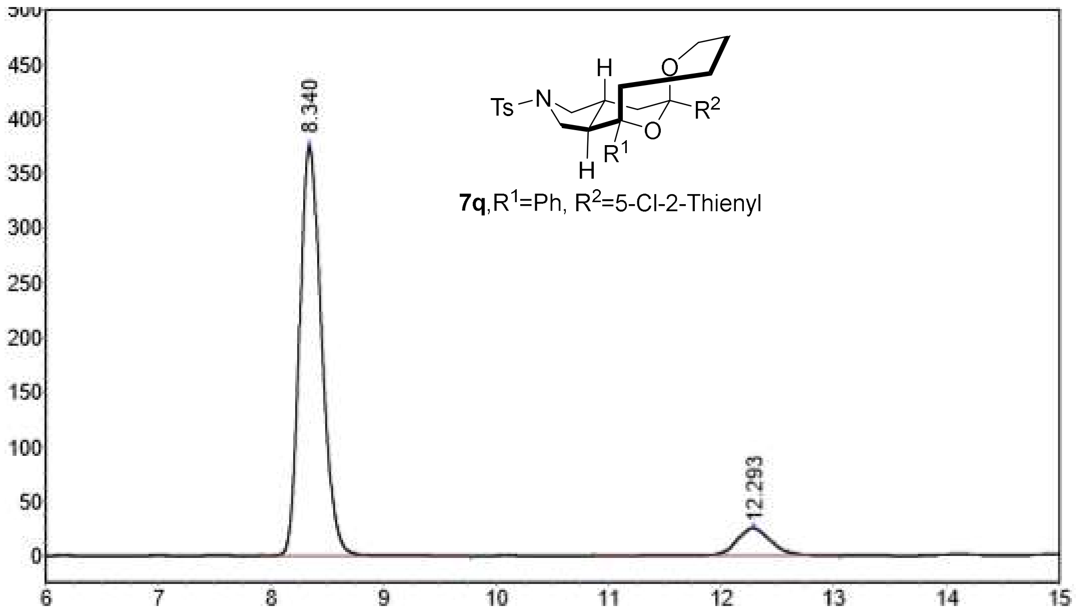

(min)

#	RT (time)	Heigh (mV*sec)	Area (mv)	Area (%)
1	8.340	374908.000	5106014.000	90.3376
2	12.293	25013.543	546134.875	9.6624

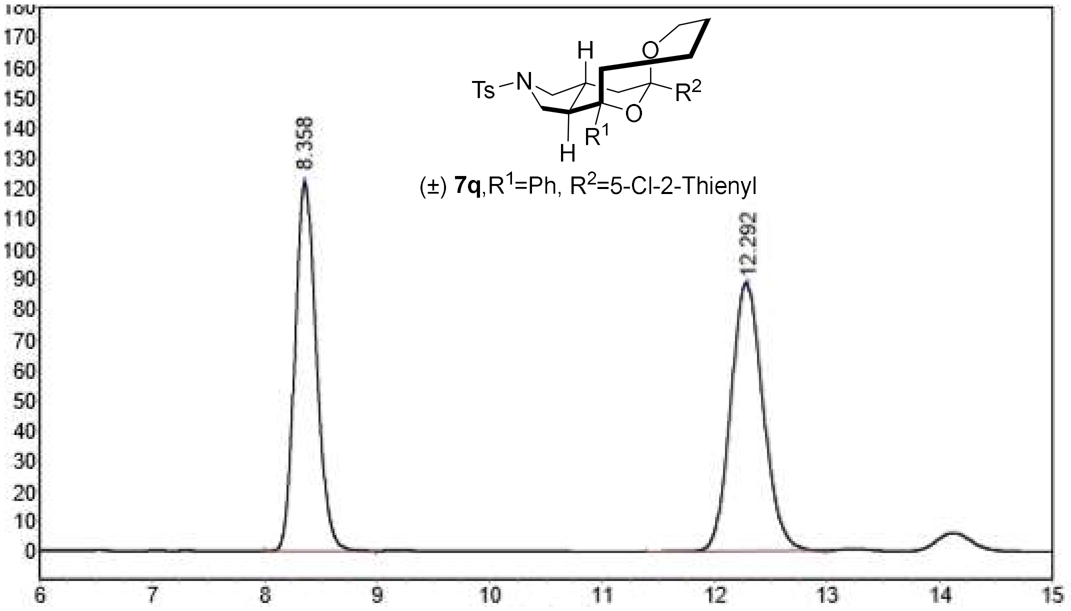

(min)

#	RT (time)	Heigh (mV*sec)	Area (mv)	Area (%)
1	8.358	121853.516	1625545.875	47.7493
2	12.292	88212.969	1778790.750	52.2507

Supplementary Figure 21. HPLC Trace of 7r.

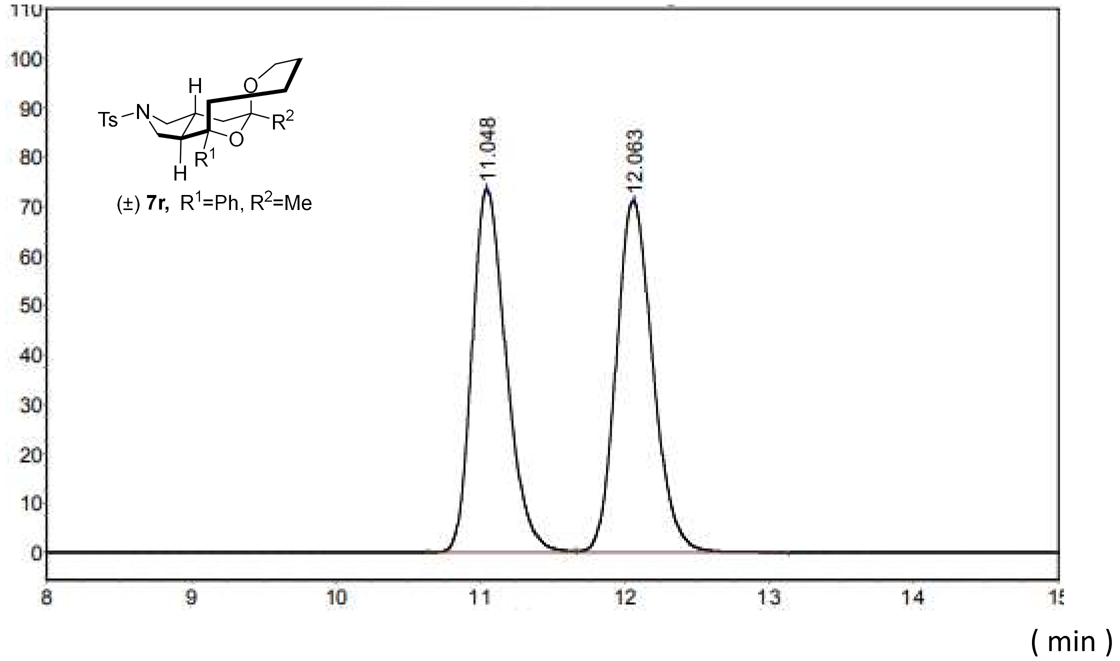

#	RT (time)	Heigh (mV*sec)	Area (mv)	Area (%)
1	11.048	73364.000	1205820.000	49.0302
2	12.063	71057.000	1253520.625	50.9698

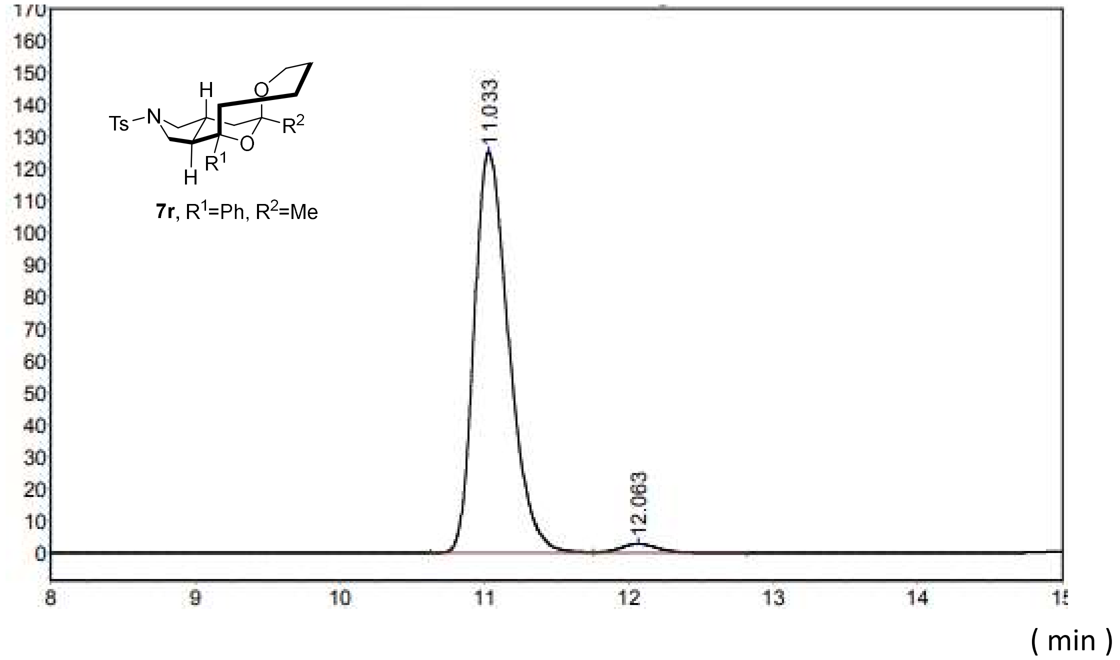

#	RT (time)	Heigh (mV*sec)	Area (mv)	Area (%)
1	11.033	125095.773	2090030.375	97.5104
2	12.063	2951.119	53360.926	2.4896

Supplementary Figure 22. HPLC Trace of 7s.

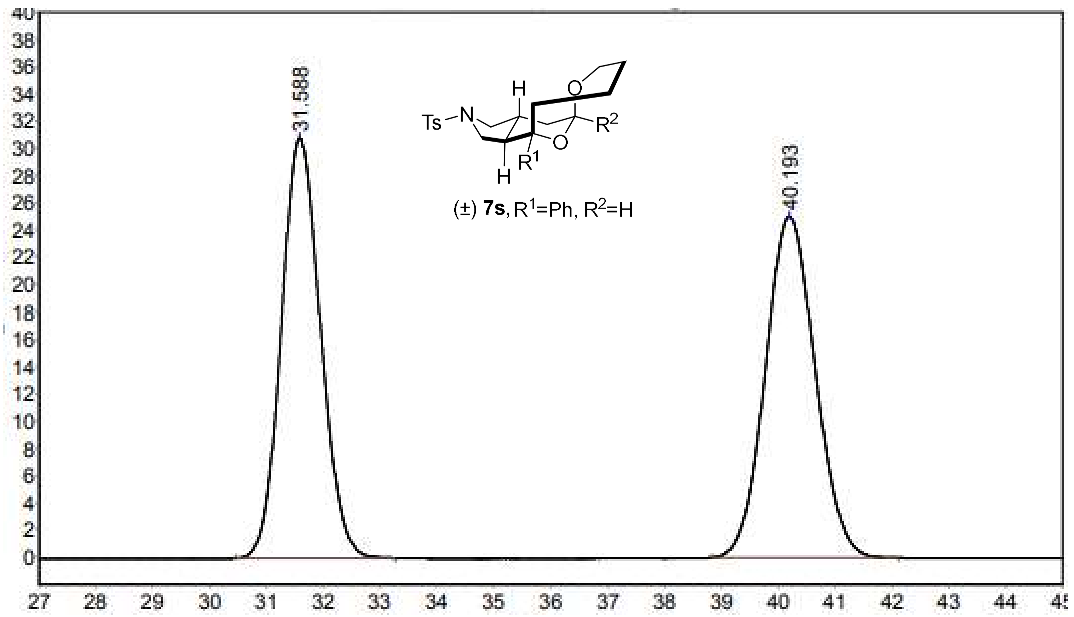

#	RT (time)	Heigh (mV*sec)	Area (mv)	Area (%)
1	31.588	30819.459	1481446.375	49.2005
2	40.193	25008.178	1529591.625	50.7995

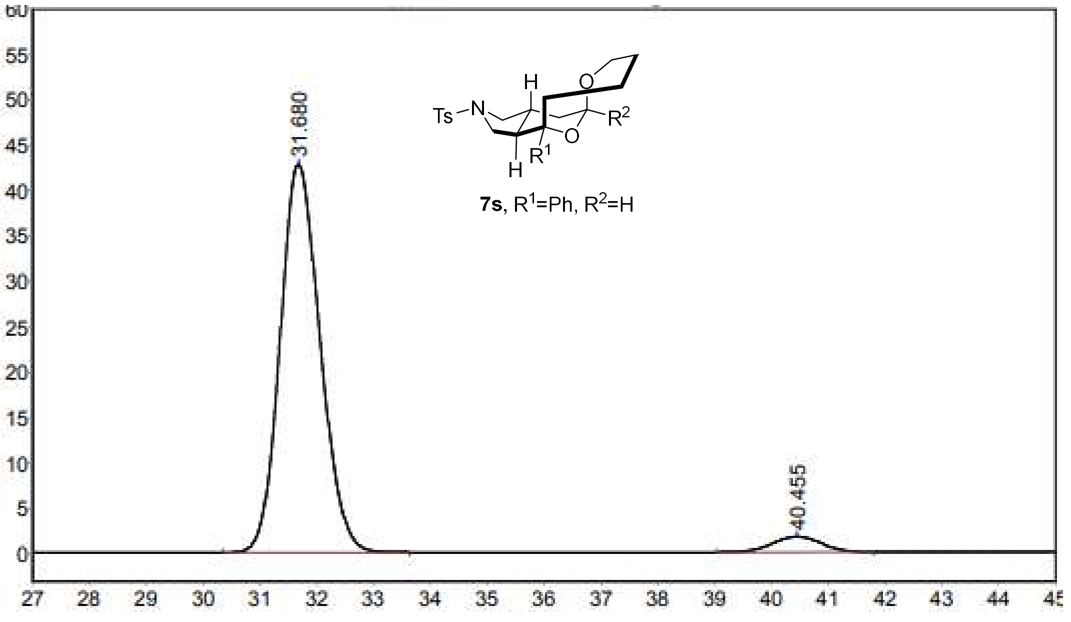

#	RT (time)	Heigh (mV*sec)	Area (mv)	Area (%)
1	31.680	42673.906	2090440.750	95.2907
2	40.455	1702.432	103309.359	4.7093

Supplementary Figure 23. HPLC Trace of 7t.

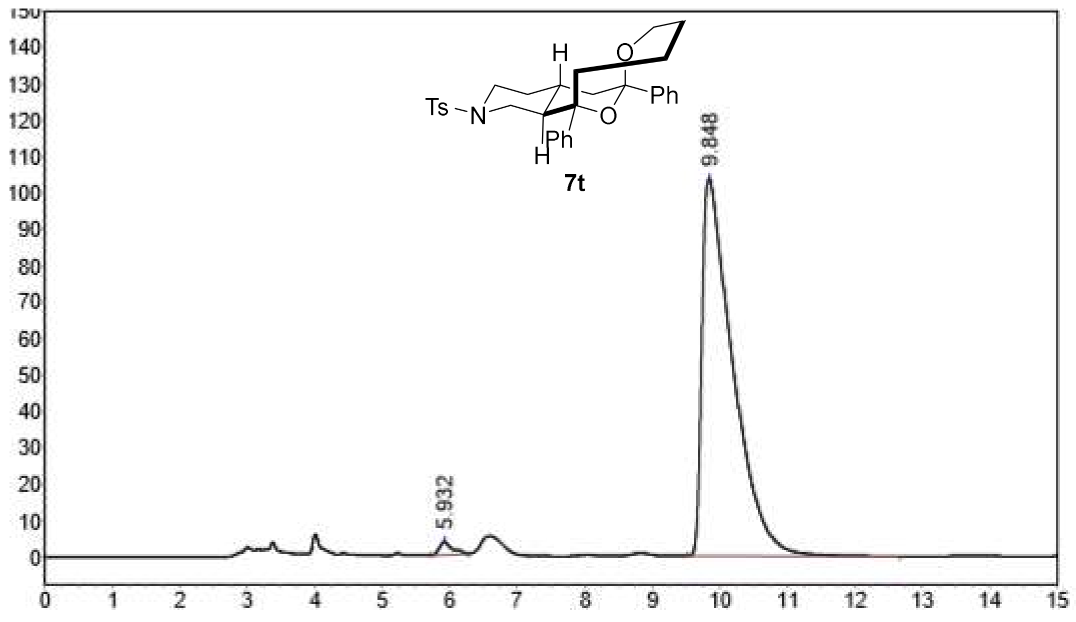

#	RT (time)	Heigh (mV*sec)	Area (mv)	Area (%)
1	5.932	3505.559	48298.199	1.4450
2	9.848	103611.445	3294130.500	98.5550

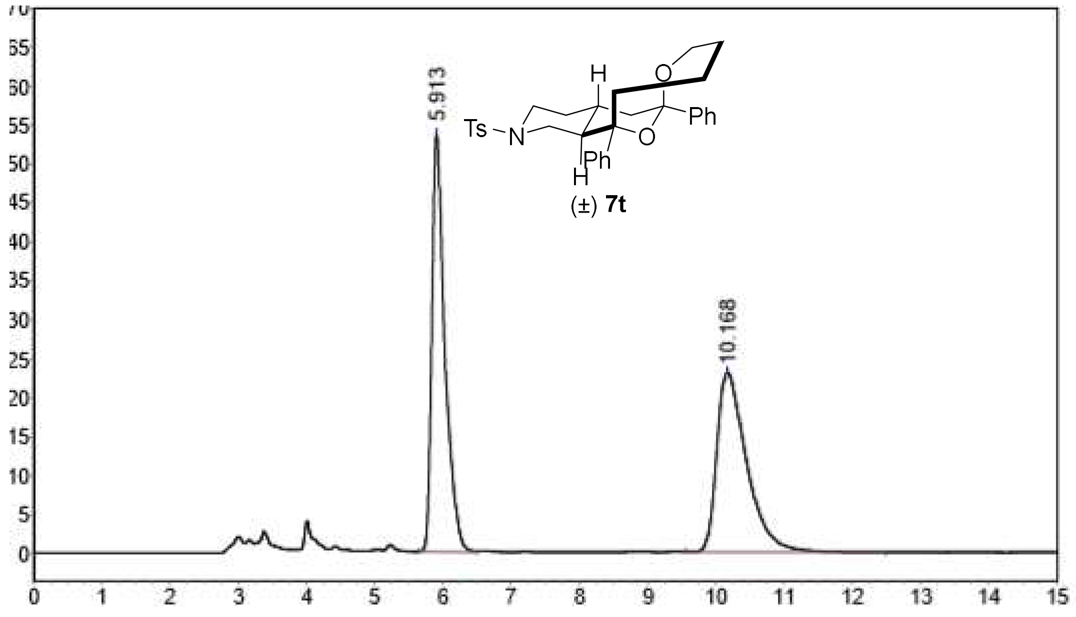

#	RT (time)	Heigh (mV*sec)	Area (mv)	Area (%)
1	5.913	53609.449	710535.938	49.9289
2	10.168	23124.129	712558.688	50.0711

Supplementary Figure 24. HPLC Trace of 9a.

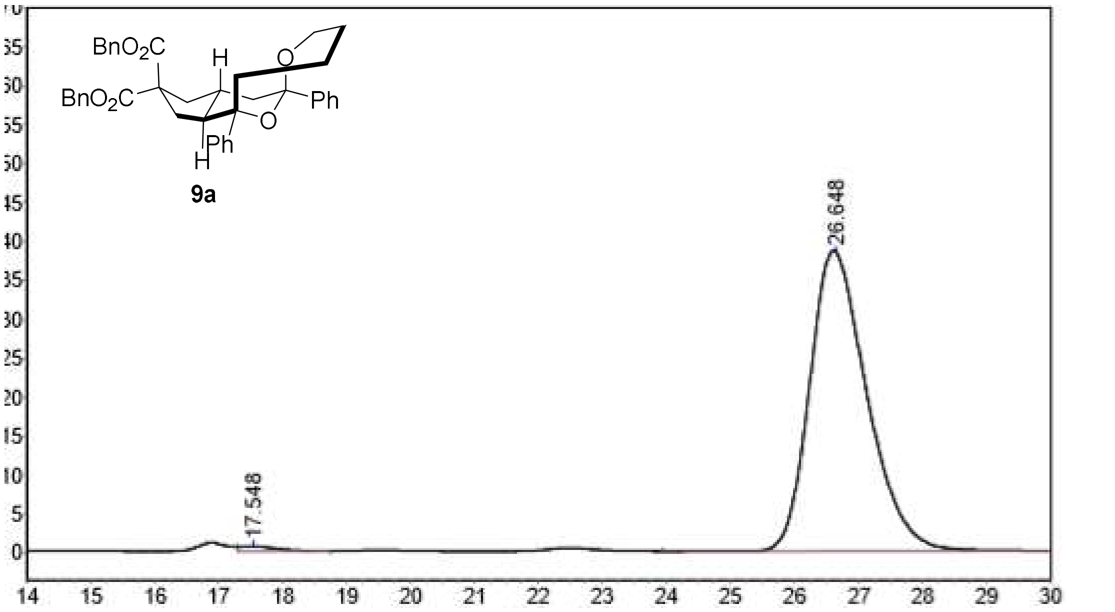

(min)

#	RT (time)	Heigh (mV*sec)	Area (mv)	Area (%)
1	17.548	659.669	25870.082	1.0546
2	26.648	38275.594	2391379.500	97.4884

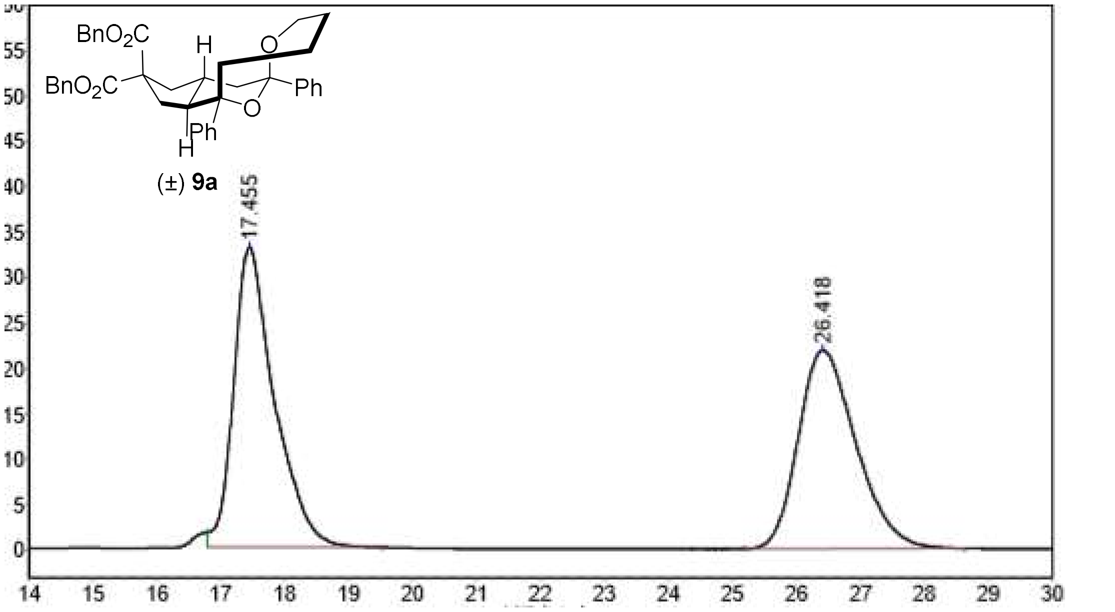

(min)

#	RT (time)	Heigh (mV*sec)	Area (mv)	Area (%)
1	17.455	32982.000	1461828.625	51.5295
2	26.418	21902.957	1375050.375	48.4705

Supplementary Figure 25. HPLC Trace of 9b.

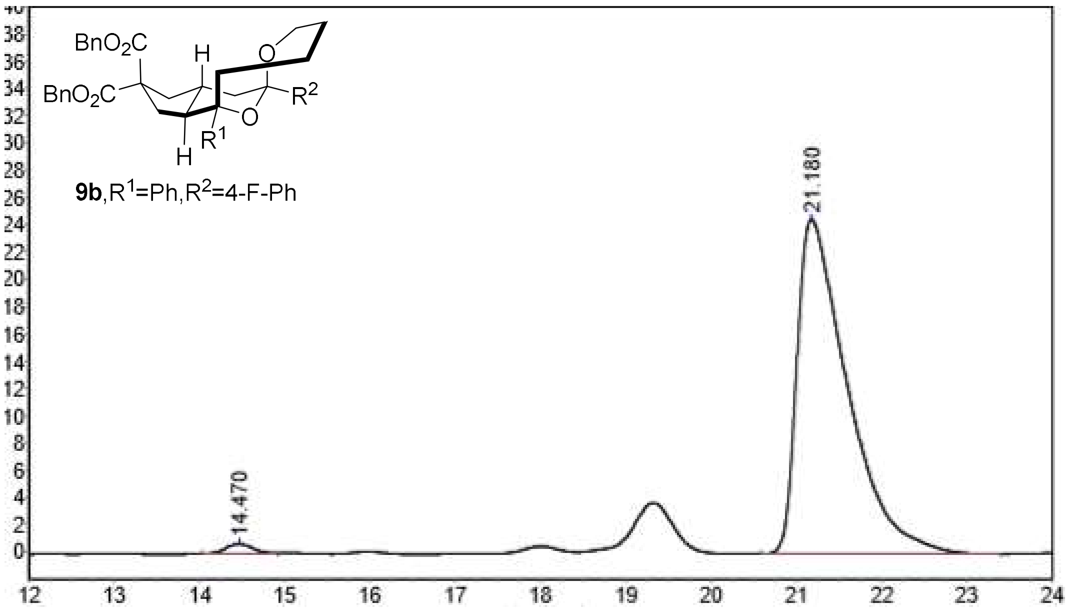

#	RT (time)	Heigh (mV*sec)	Area (mv)	Area (%)
1	14.470	755.000	16896.000	1.6506
2	21.180	24514.596	1006724.000	98.3494

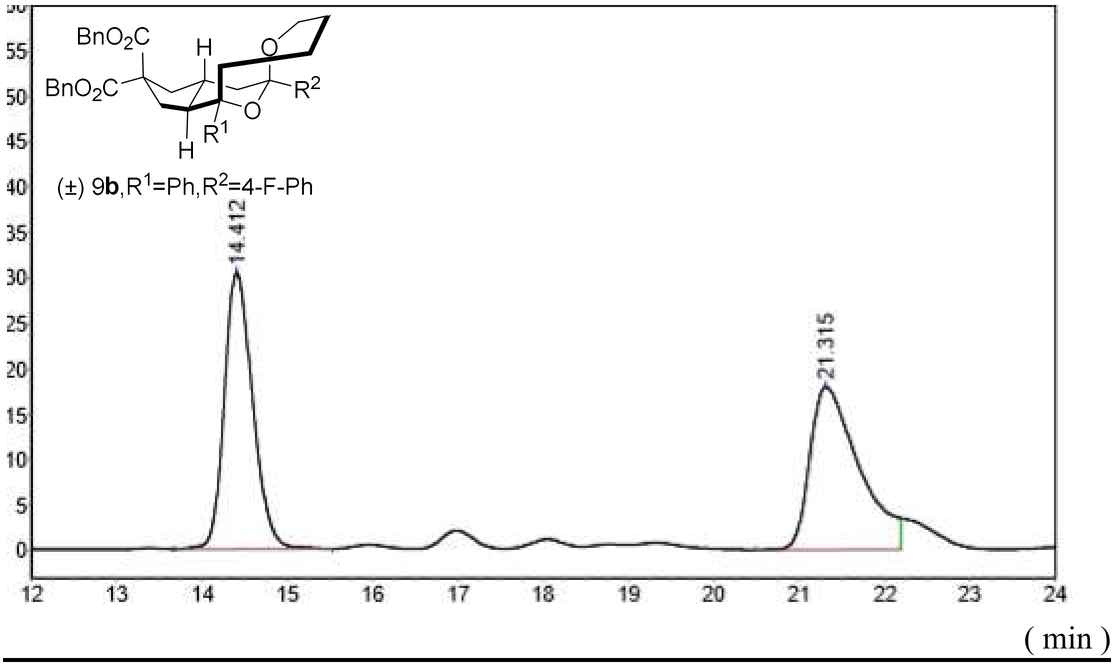

#	RT (time)	Heigh (mV*sec)	Area (mv)	Area (%)
1	14.412	30380.723	705111.313	49.6994
2	21.315	17939.518	713640.438	50.3006

Supplementary Figure 26. HPLC Trace of 9c.

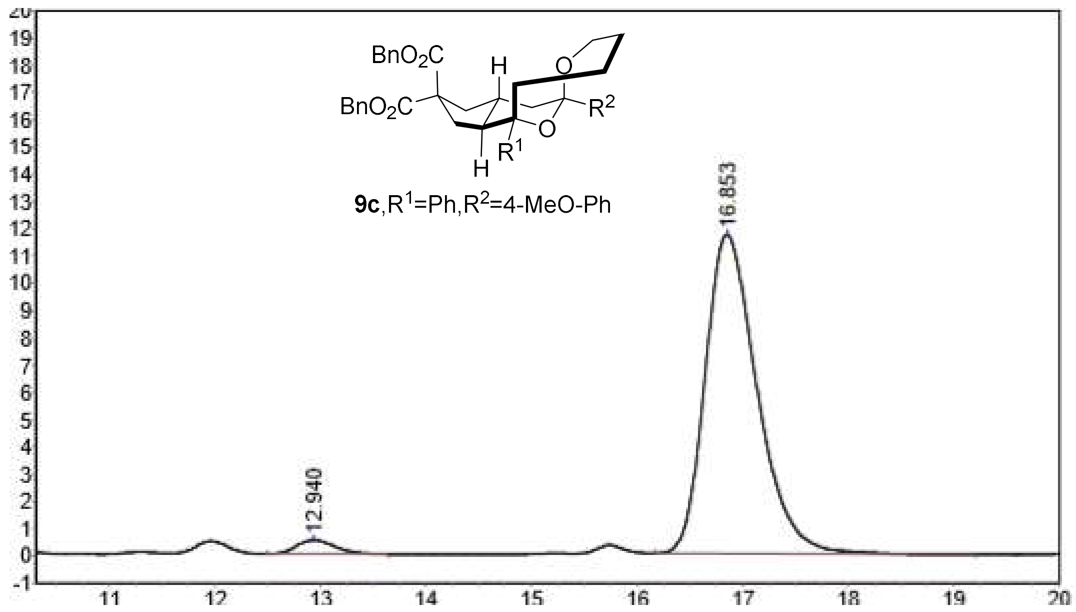

#	RT (time)	Heigh (mV*sec)	Area (mv)	Area (%)
1	12.940	507.000	13302.999	3.2153
2	16.853	11682.649	400442.625	96.7847

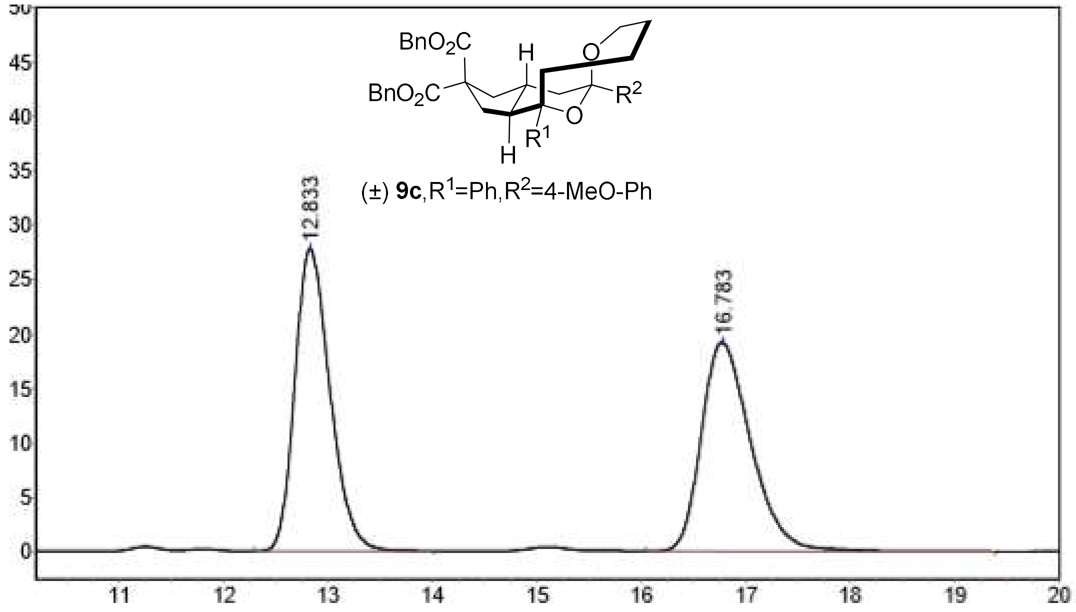

#	RT (time)	Heigh (mV*sec)	Area (mv)	Area (%)
1	12.833	27753.125	652175.500	50.6179
2	16.783	19203.631	636253.438	49.3821

Supplementary Figure 27. HPLC Trace of 9d.

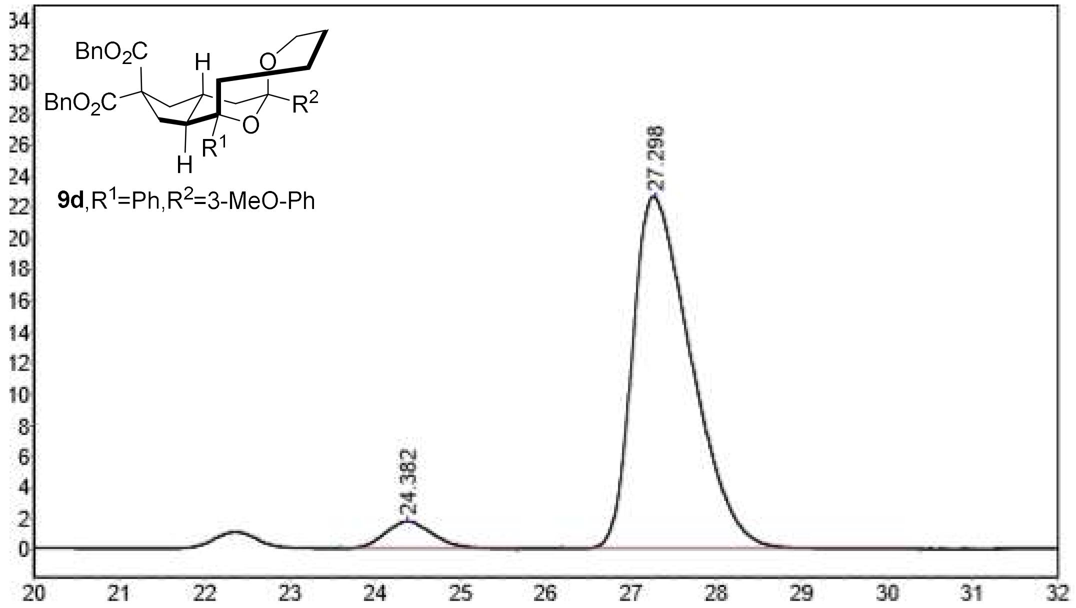

#	RT (time)	Heigh (mV*sec)	Area (mv)	Area (%)
1	24.382	1728.526	68893.703	5.9698
2	27.298	22591.096	1085145.375	94.0302

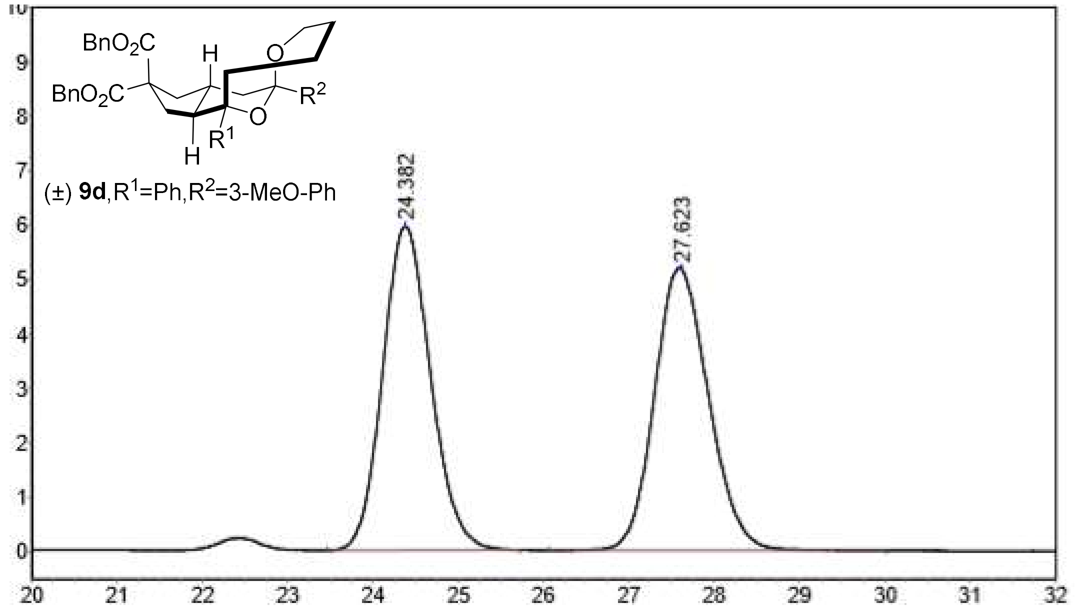

#	RT (time)	Heigh (mV*sec)	Area (mv)	Area (%)
1	24.382	5938.159	232907.750	50.3642
2	27.623	5177.975	229539.594	49.6358

Supplementary Figure 28. HPLC Trace of 9e.

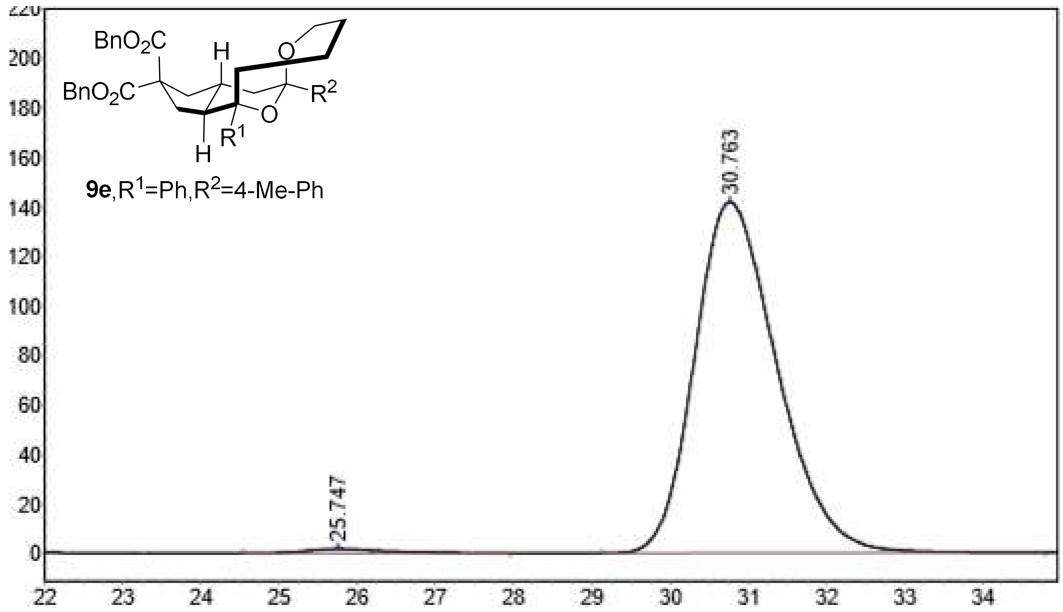

#	RT (time)	Heigh (mV*sec)	Area (mv)	Area (%)
1	25.747	1838.688	138434.906	1.3098
2	30.763	141662.422	10430542.000	98.6902

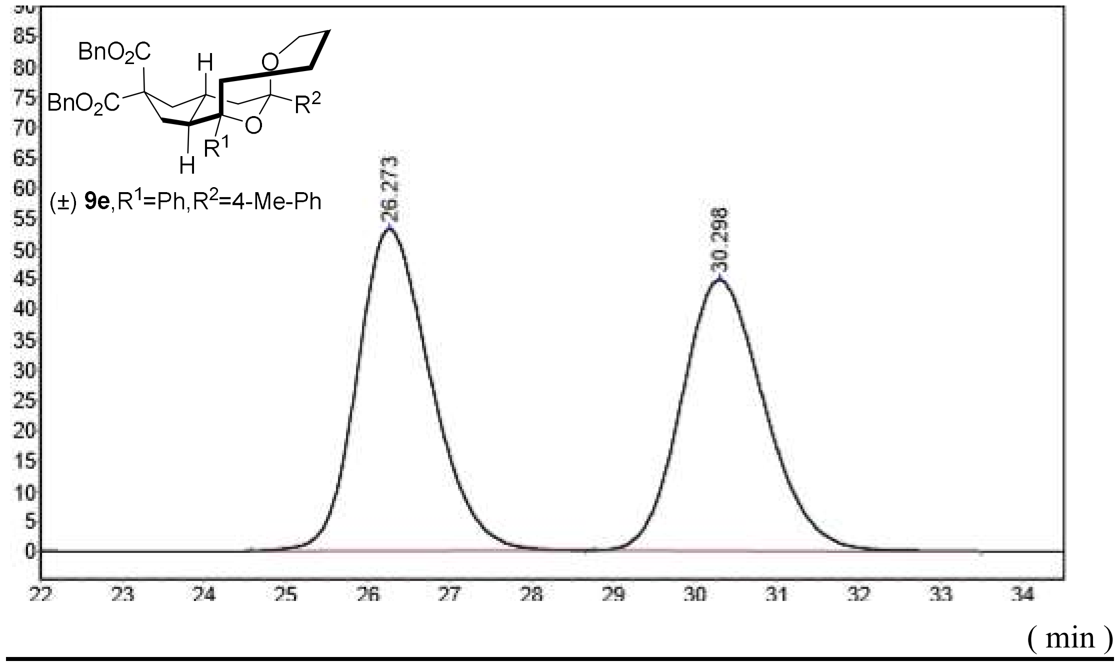

#	RT (time)	Heigh (mV*sec)	Area (mv)	Area (%)
1	26.273	53111.258	3323172.250	51.4049
2	30.298	44658.422	3141532.250	48.5951

Supplementary Figure 29. HPLC Trace of 9f.

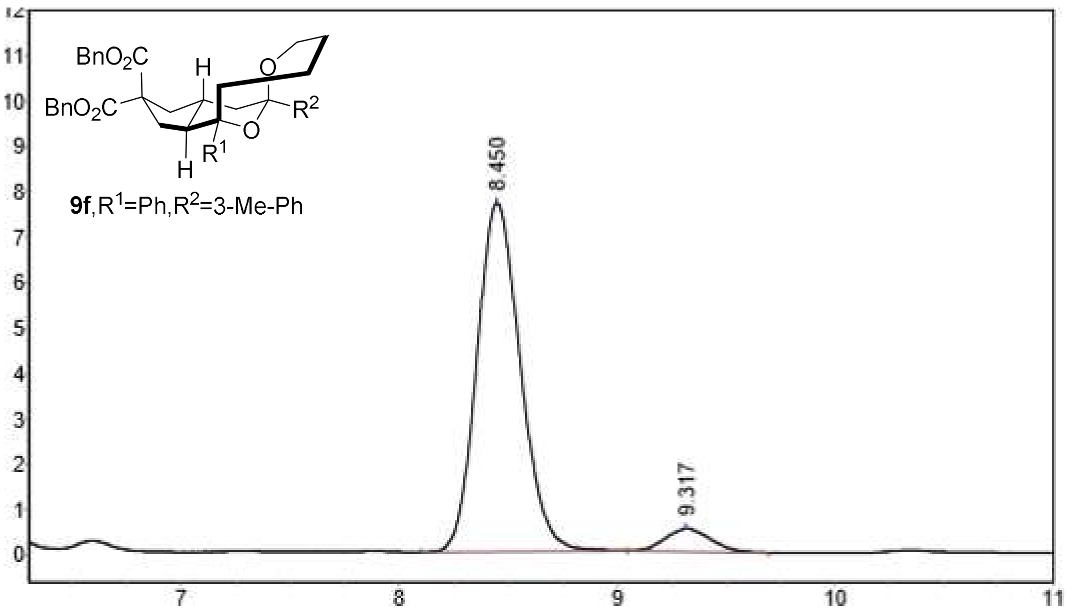

#	RT (time)	Heigh (mV*sec)	Area (mv)	Area (%)
1	8.450	7671.066	106649.750	93.6732
2	9.317	498.673	7203.300	6.3268

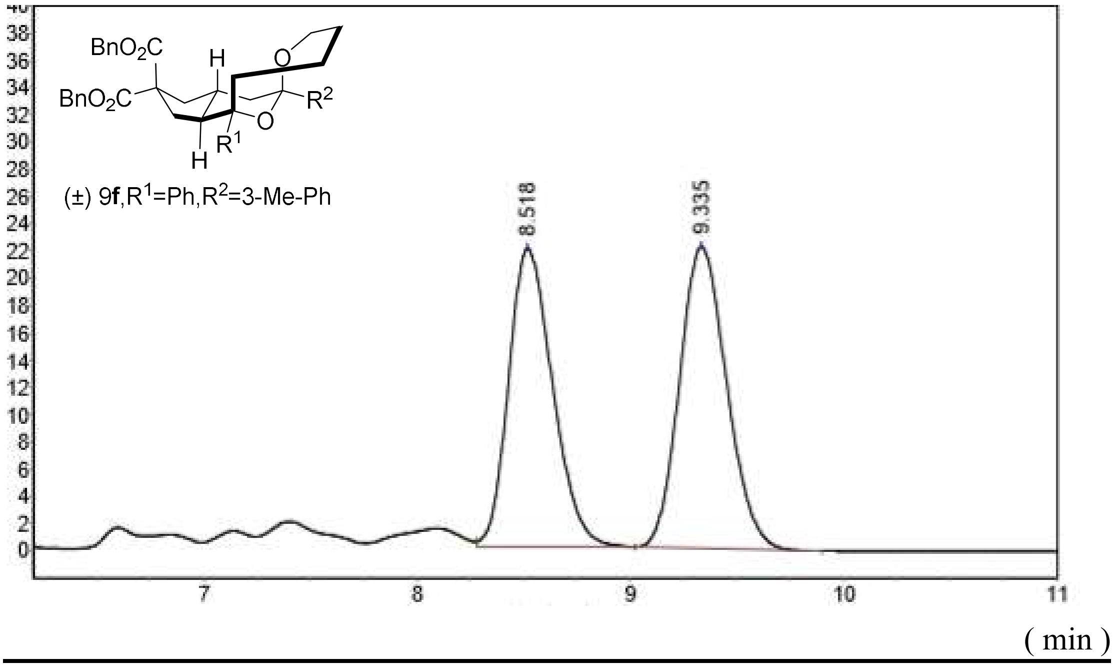

#	RT (time)	Heigh (mV*sec)	Area (mv)	Area (%)
1	8.518	21879.000	314960.594	48.0708
2	9.335	22123.076	340240.563	51.9292

Supplementary Figure 30. HPLC Trace of 9g.

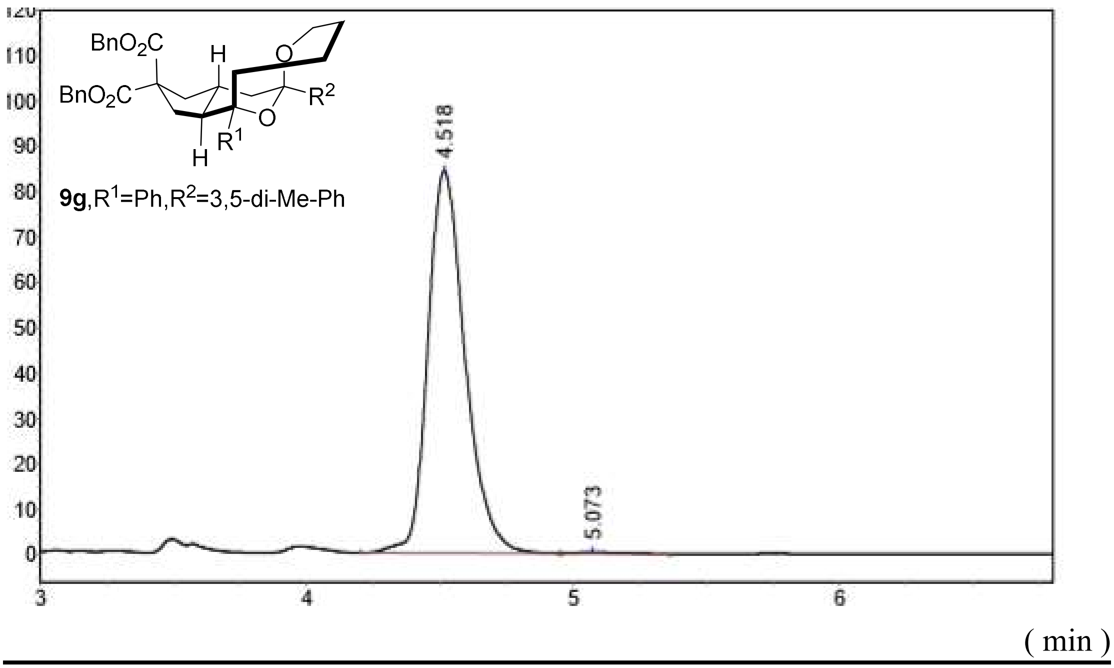

#	RT (time)	Heigh (mV*sec)	Area (mv)	Area (%)
1	4.518	84238.656	821081.750	99.4132
2	5.073	396.000	4846.159	0.5868

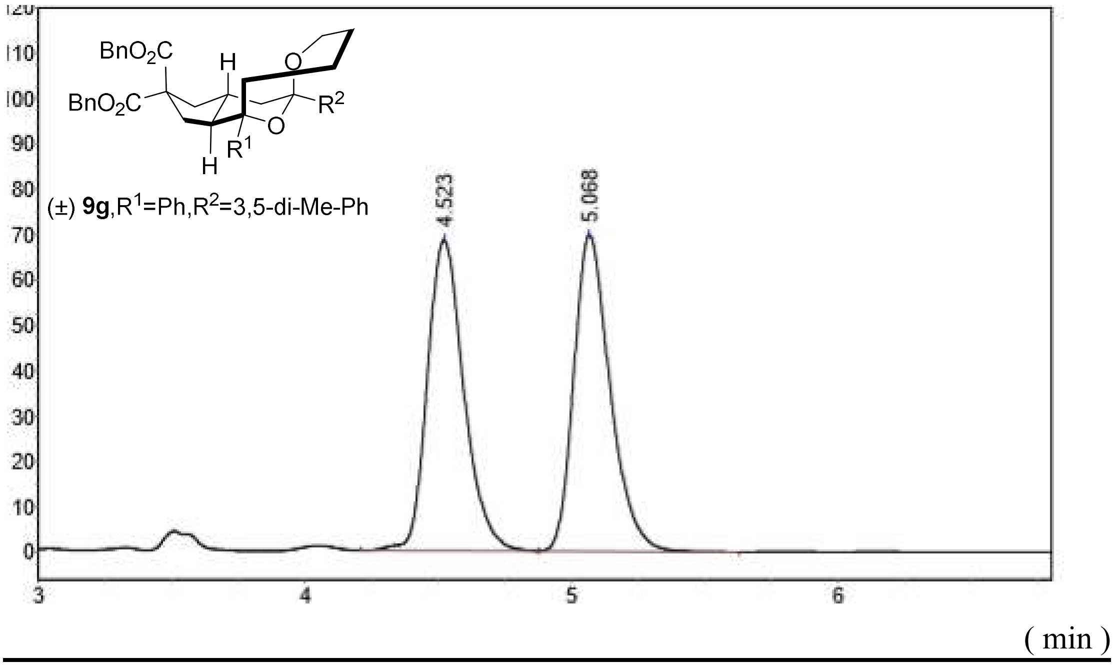

#	RT (time)	Heigh (mV*sec)	Area (mv)	Area (%)
1	4.523	68512.430	654931.438	49.6507
2	5.068	69649.563	664146.563	50.3493

Supplementary Figure 31. HPLC Trace of 9h.

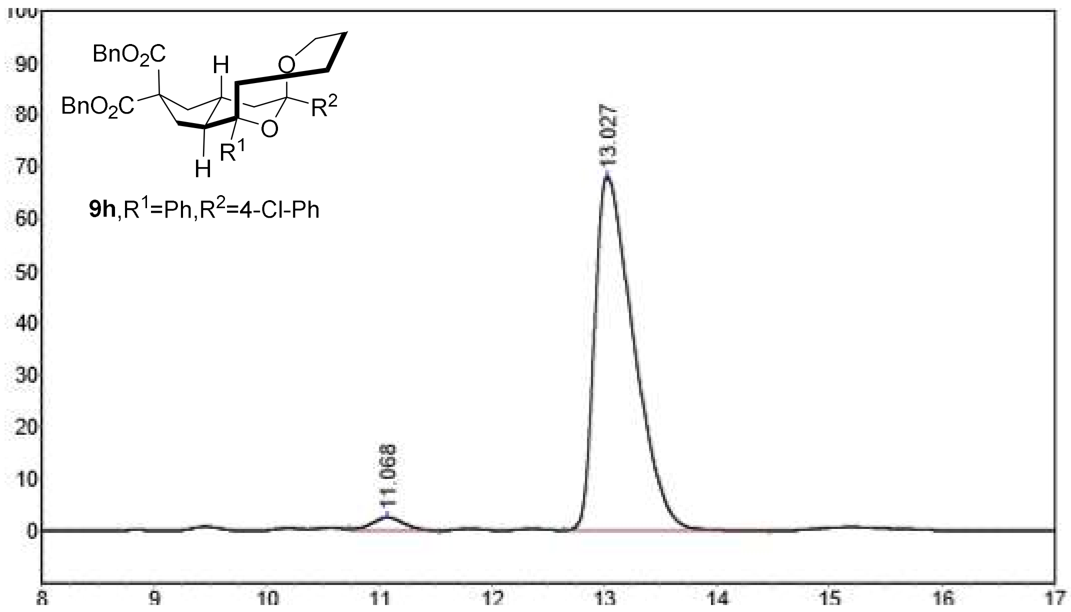

#	RT (time)	Heigh (mV*sec)	Area (mv)	Area (%)
1	11.068	2530.000	49473.801	2.9327
2	13.027	68026.570	1637496.375	97.0673

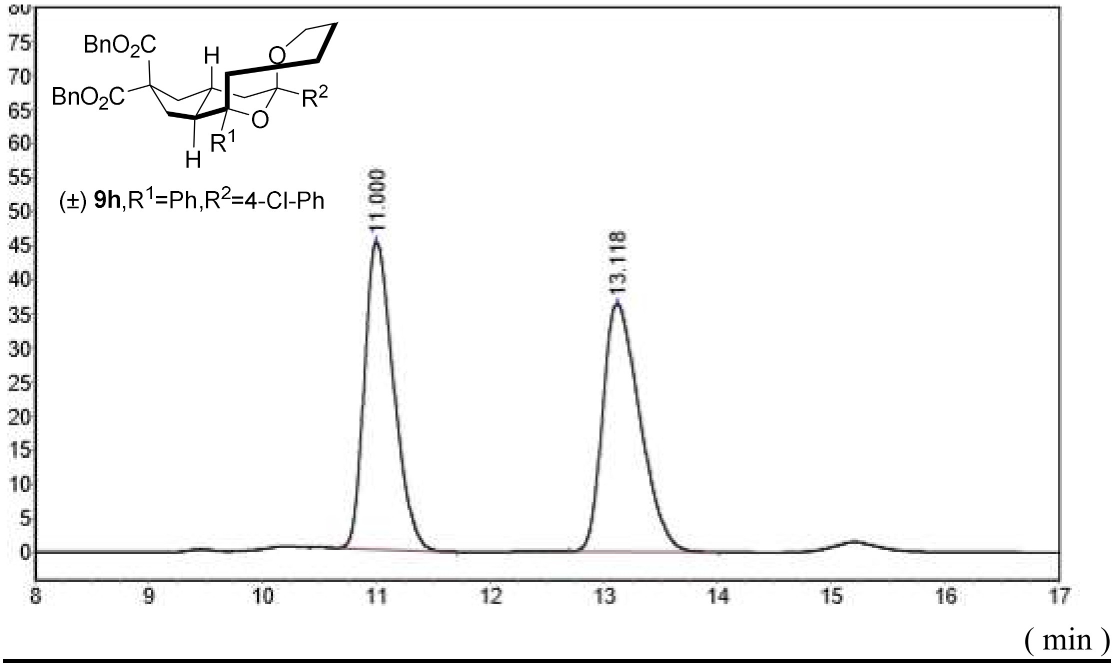

#	RT (time)	Heigh (mV*sec)	Area (mv)	Area (%)
1	11.000	45034.953	802370.875	49.2725
2	13.118	36352.504	826063.313	50.7275

Supplementary Figure 32. HPLC Trace of 9i.

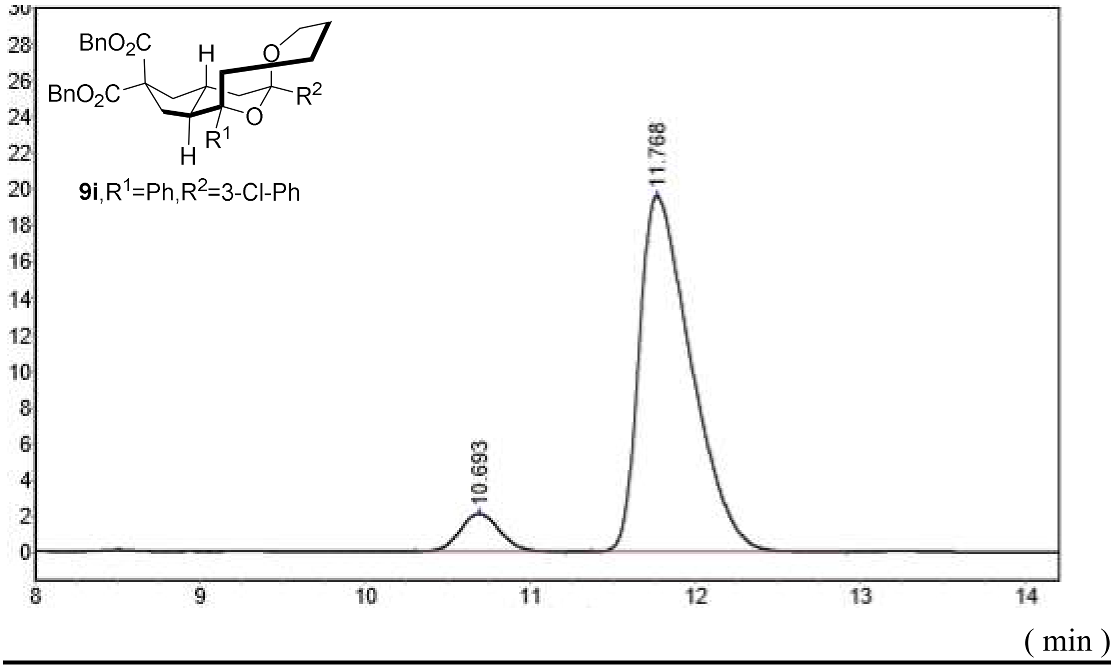

#	RT (time)	Heigh (mV*sec)	Area (mv)	Area (%)
1	10.693	2086.286	35211.000	7.6040
2	11.768	19555.000	427848.313	92.3960

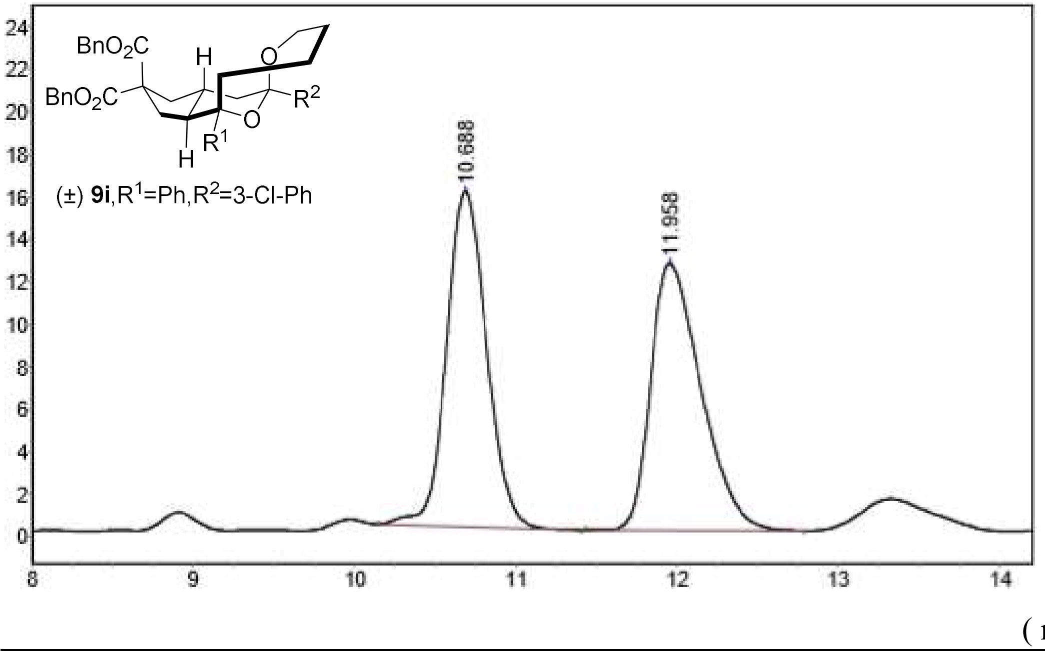

#	RT (time)	Heigh (mV*sec)	Area (mv)	Area (%)
1	10.688	15764.969	272068.938	50.0976
2	11.958	12538.660	271008.844	49.9024

Supplementary Figure 33. HPLC Trace of 9j.

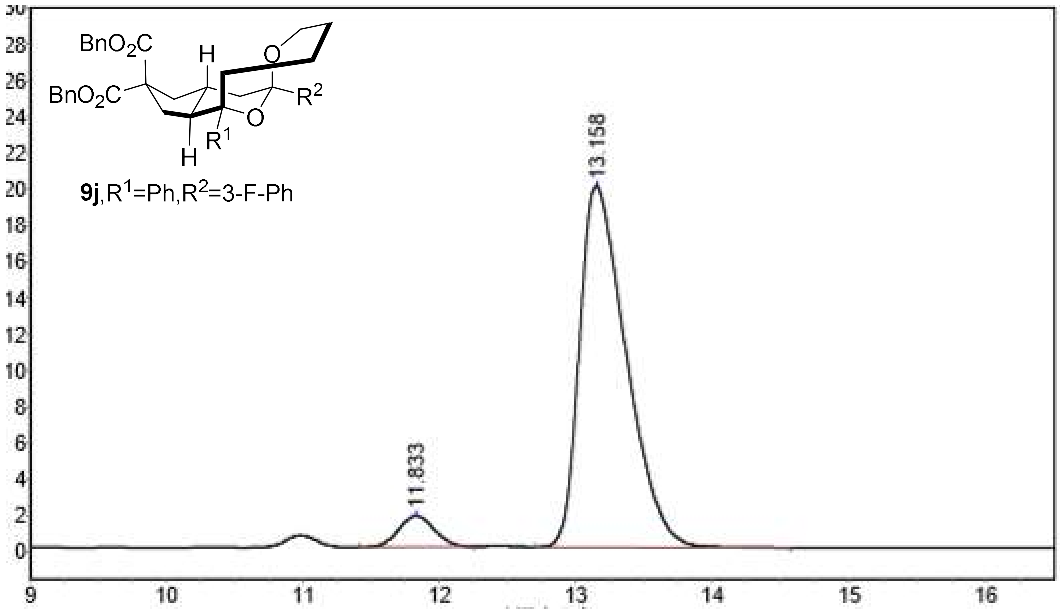

(min)

#	RT (time)	Heigh (mV*sec)	Area (mv)	Area (%)
1	11.833	1711.000	31248.400	6.1897
2	13.158	19899.053	473594.594	93.8103

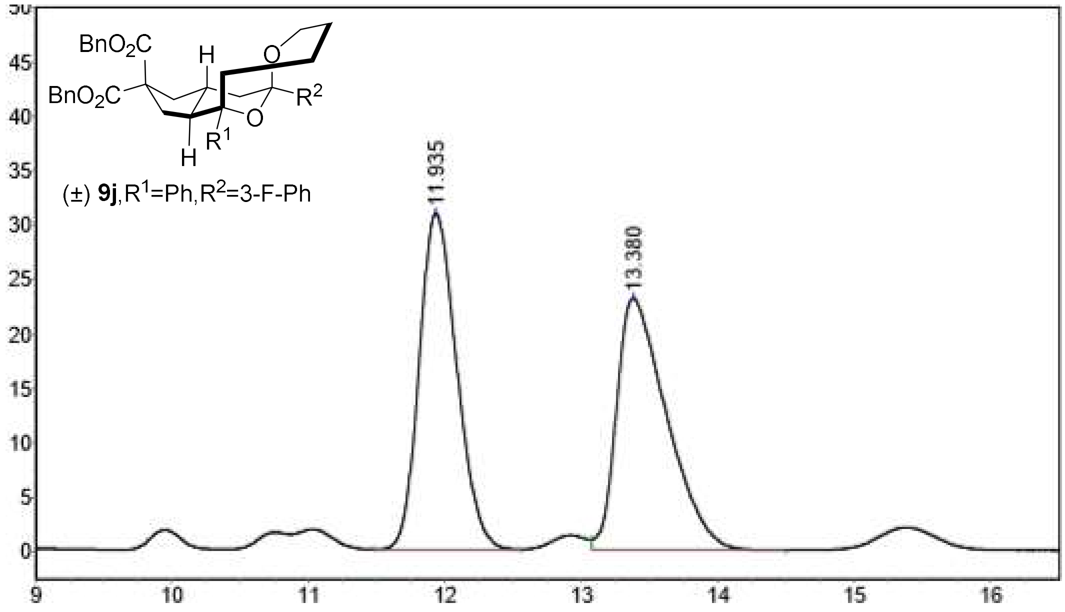

(min)

#	RT (time)	Heigh (mV*sec)	Area (mv)	Area (%)
1	11.935	30868.838	591146.563	50.0625
2	13.380	23064.684	589671.438	49.9375

Supplementary Figure 34. HPLC Trace of 9k.

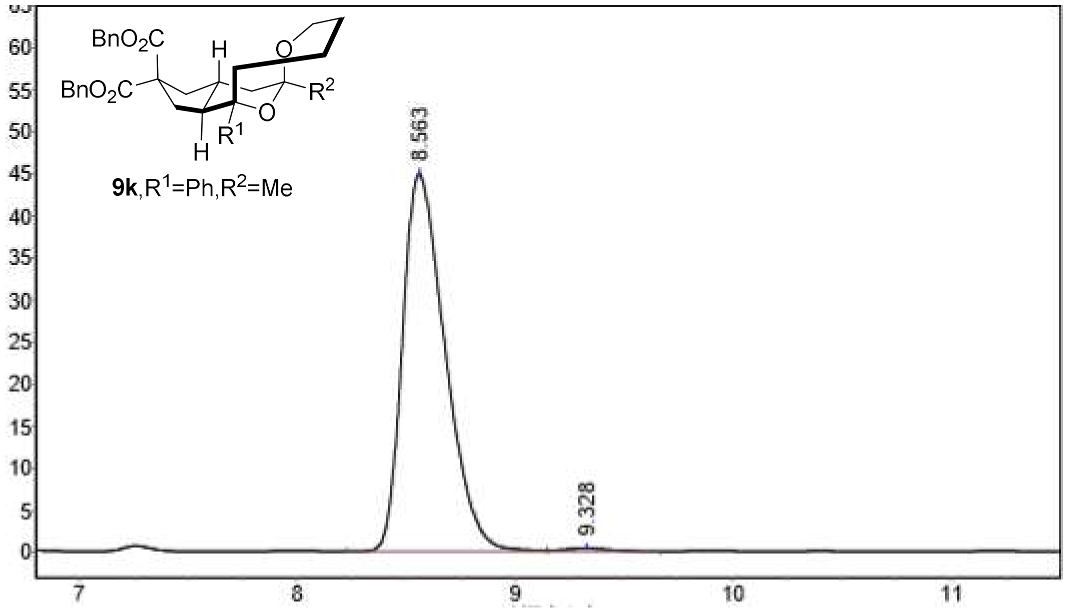

(min)

#	RT (time)	Heigh (mV*sec)	Area (mv)	Area (%)
1	8.563	44882.535	612058.563	99.0635
2	9.328	389.472	5785.967	0.9365

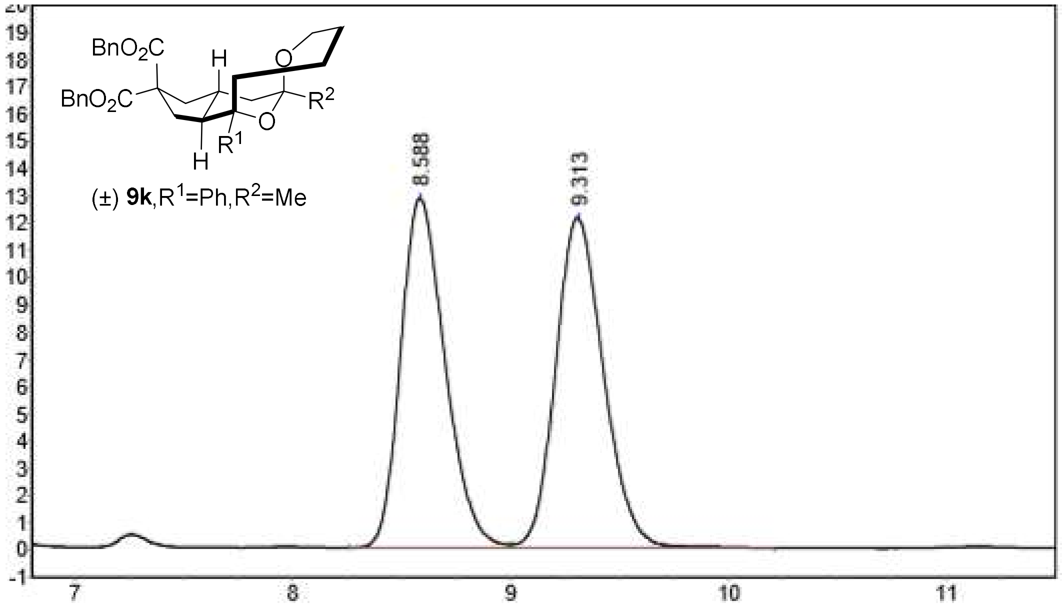

(min)

#	RT (time)	Heigh (mV*sec)	Area (mv)	Area (%)
1	8.588	12810.675	181222.516	49.5446
2	9.313	12086.158	184553.734	50.4554

Supplementary Figure 35. HPLC Trace of 9l.

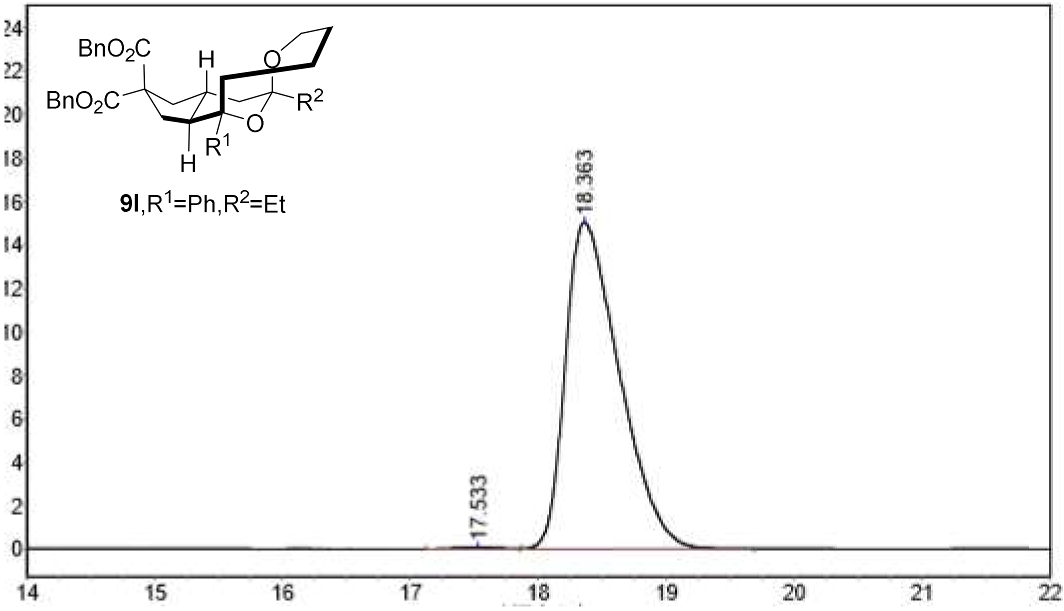

(min)

#	RT (time)	Heigh (mV*sec)	Area (mv)	Area (%)
1	17.533	88.078	2032.931	0.4582
2	18.363	15001.188	441676.688	99.5418

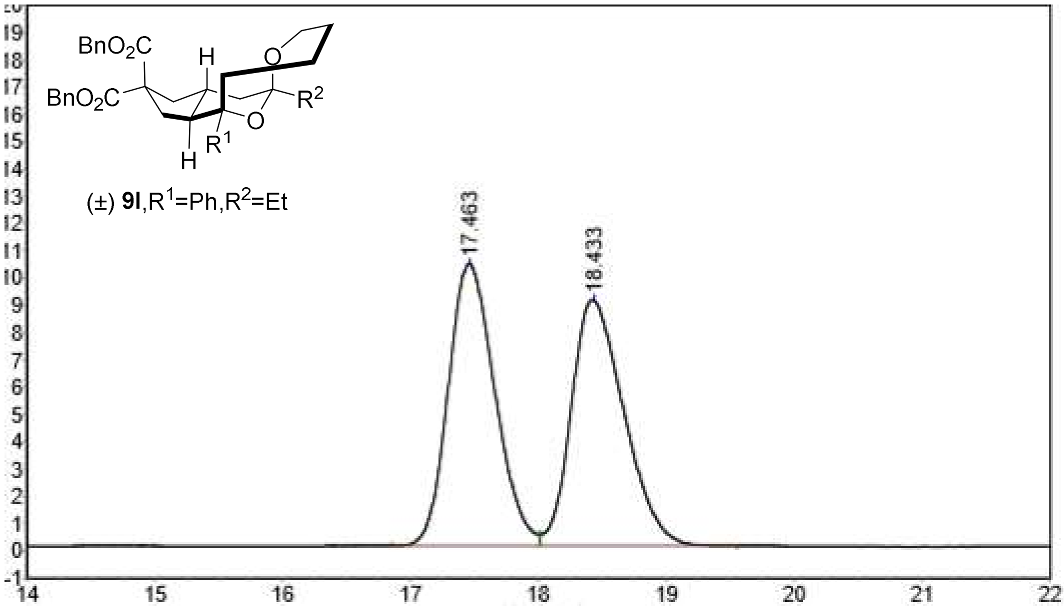

(min)

#	RT (time)	Heigh (mV*sec)	Area (mv)	Area (%)
1	17.463	10318.291	261793.203	50.4723
2	18.433	8994.835	256893.453	49.5277

Supplementary Figure 36. HPLC Trace of 9m.

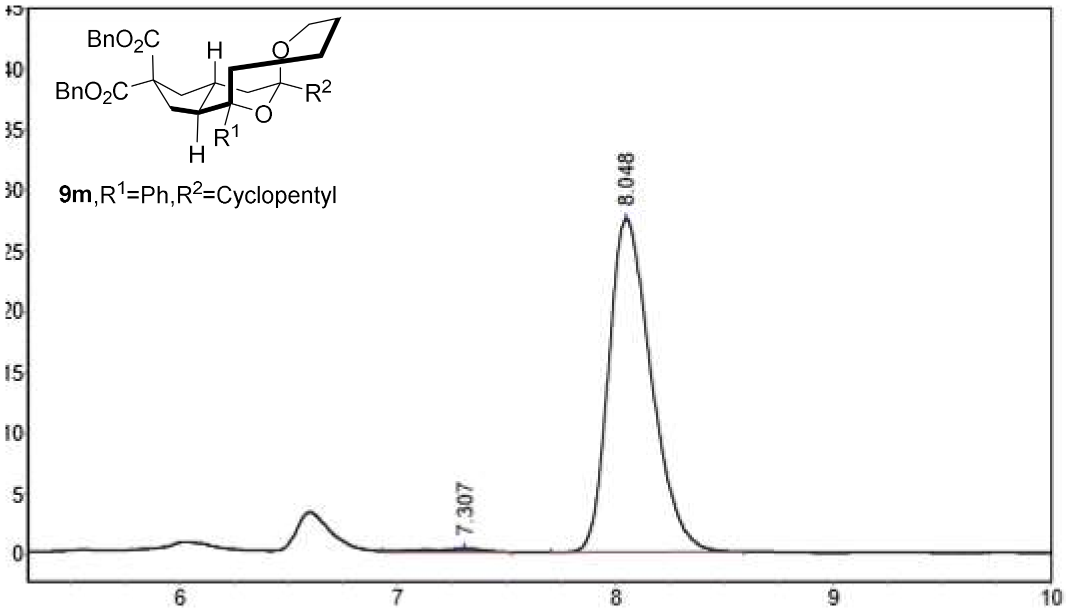

#	RT (time)	Heigh (mV*sec)	Area (mv)	Area (%)
1	7.307	285.000	5591.001	1.4750
2	8.048	27438.822	373464.781	98.5250

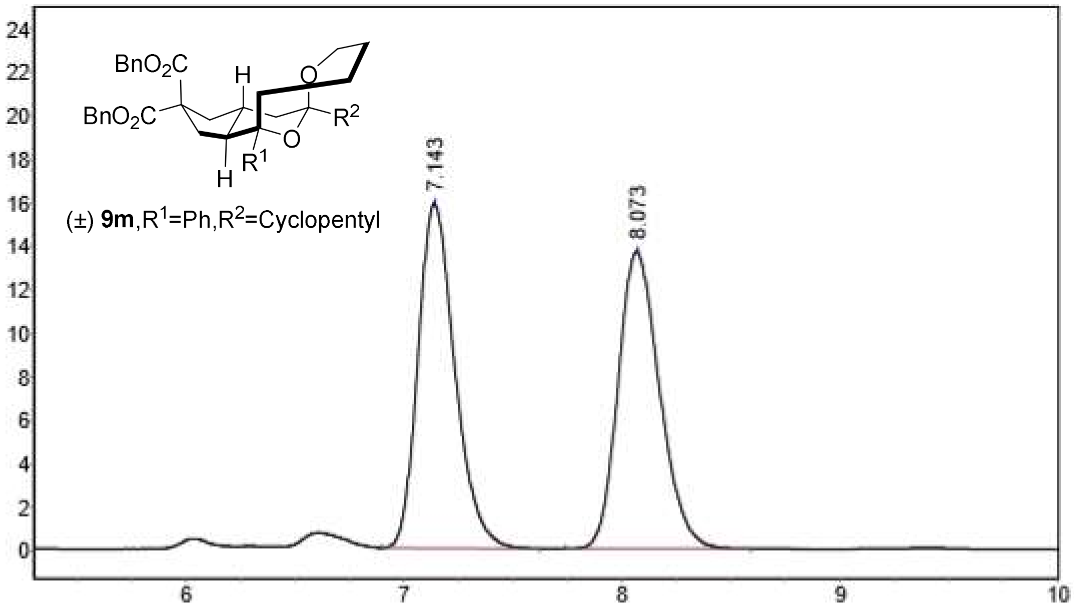

#	RT (time)	Heigh (mV*sec)	Area (mv)	Area (%)
1	7.143	15792.489	185821.000	50.7131
2	8.073	13619.553	180595.094	49.2869

Supplementary Figure 37. HPLC Trace of 9n.

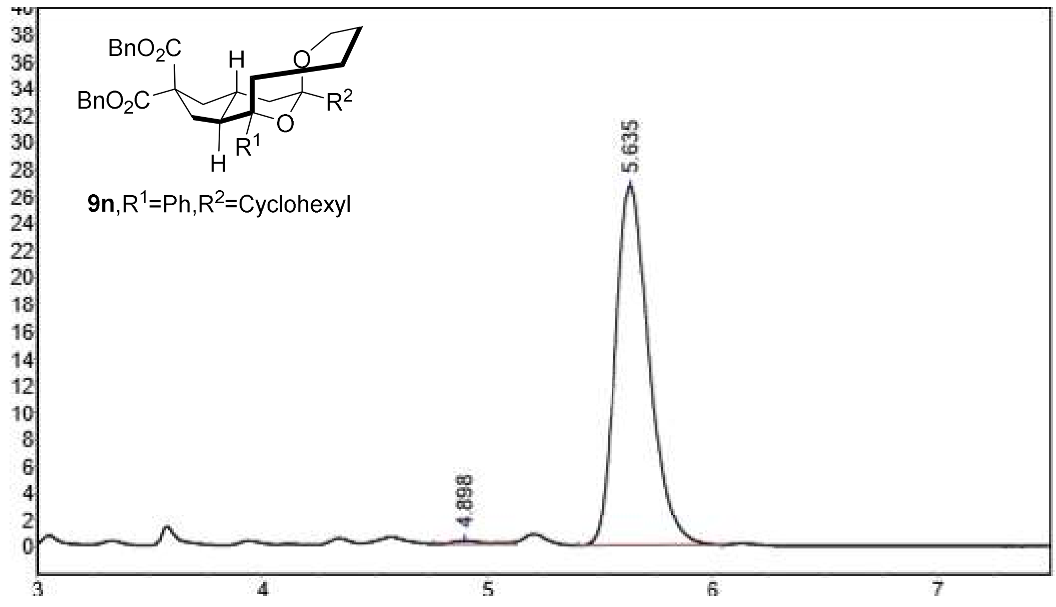

#	RT (time)	Heigh (mV*sec)	Area (mv)	Area (%)
1	4.898	259.000	2818.700	1.0060
2	5.635	26649.115	277370.313	98.9940

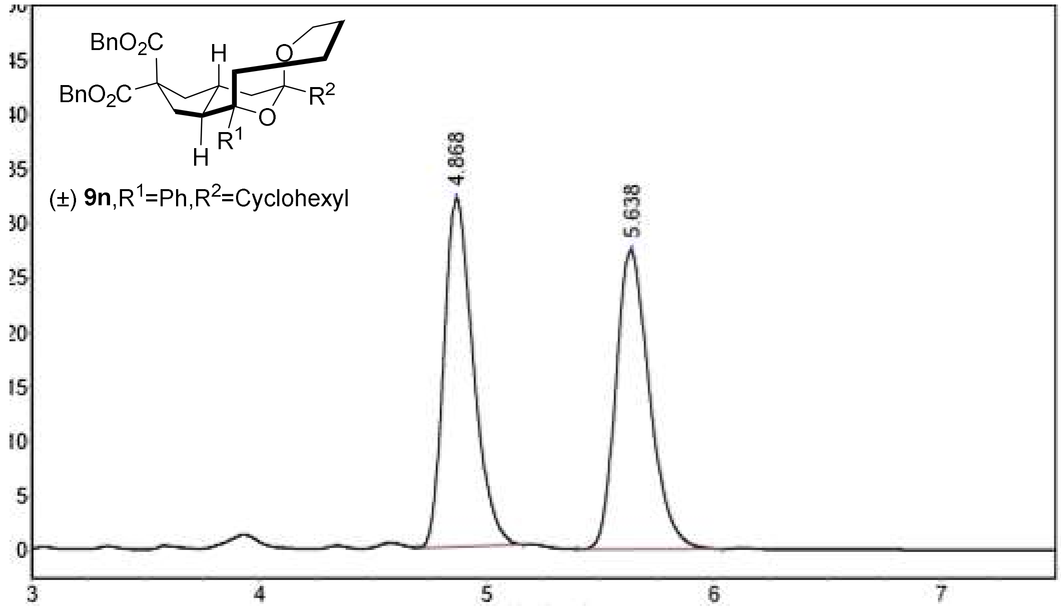

#	RT (time)	Heigh (mV*sec)	Area (mv)	Area (%)
1	4.868	31839.721	286330.750	50.3250
2	5.638	27377.234	282632.188	49.6750

Supplementary Figure 38. HPLC Trace of 11a.

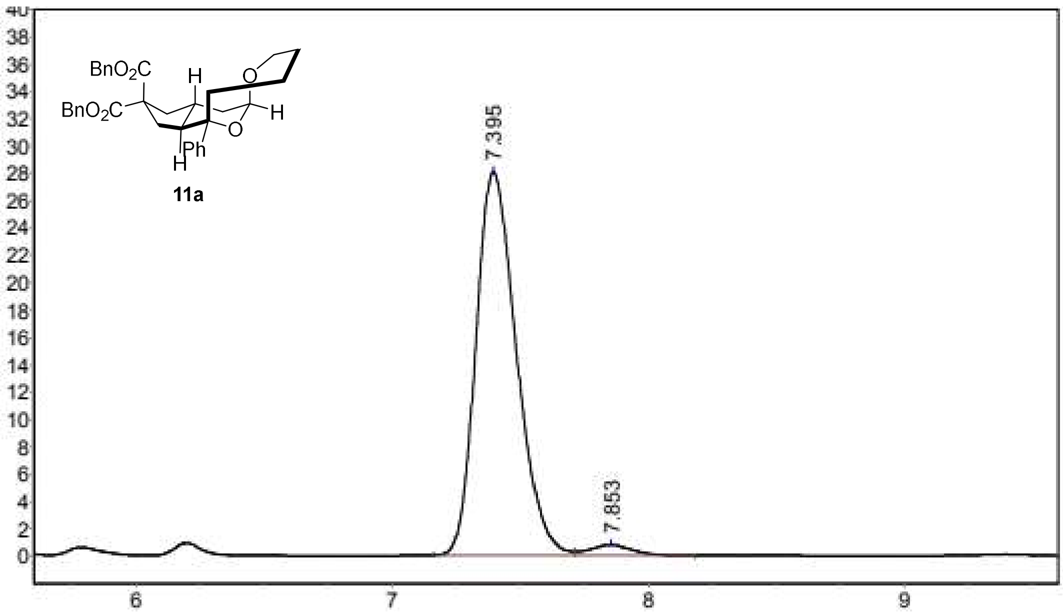

(min)

#	RT (time)	Heigh (mV*sec)	Area (mv)	Area (%)
1	7.395	28039.188	307174.563	97.2253
2	7.853	751.339	8766.476	2.7747

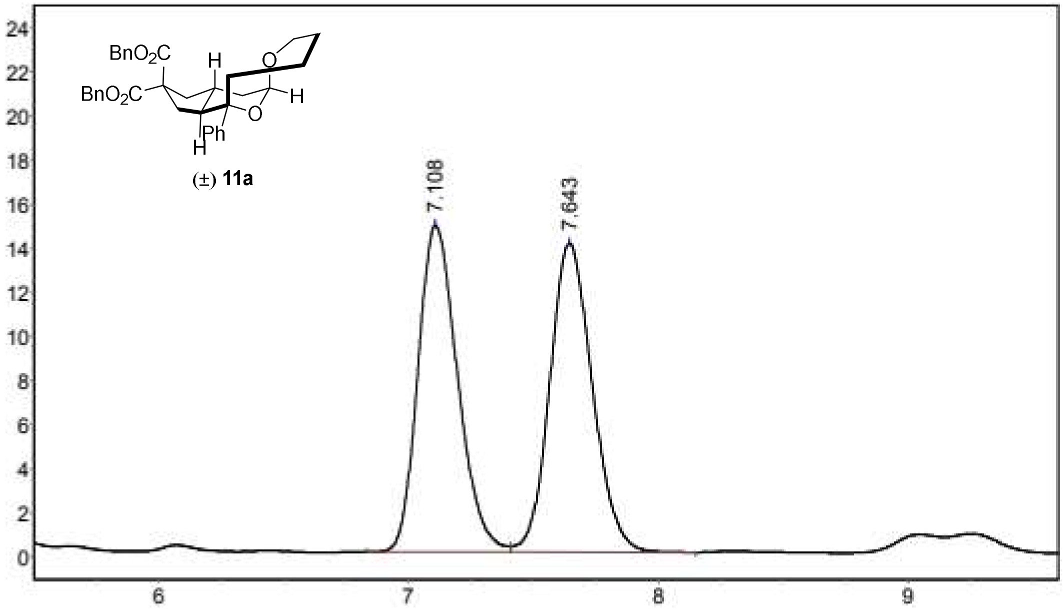

(min)

#	RT (time)	Heigh (mV*sec)	Area (mv)	Area (%)
1	7.108	14799.019	163219.828	49.6301
2	7.643	13977.783	165652.672	50.3699

Supplementary Figure 39. HPLC Trace of 11b.

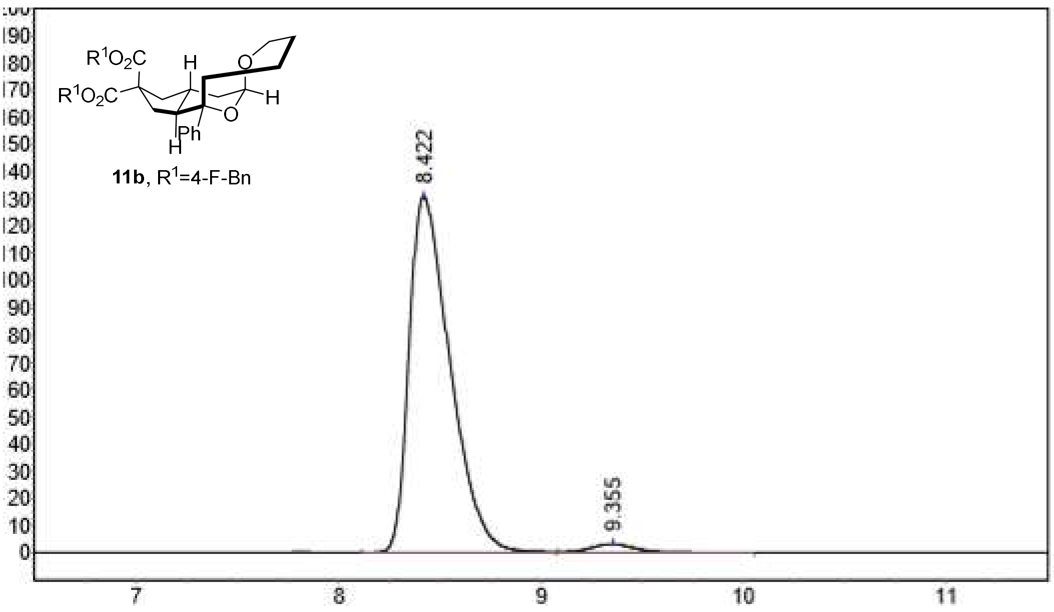

#	RT (time)	Heigh (mV*sec)	Area (mv)	Area (%)
1	8.422	130801.891	1840626.625	97.4219
2	9.355	3212.526	48708.551	2.5781

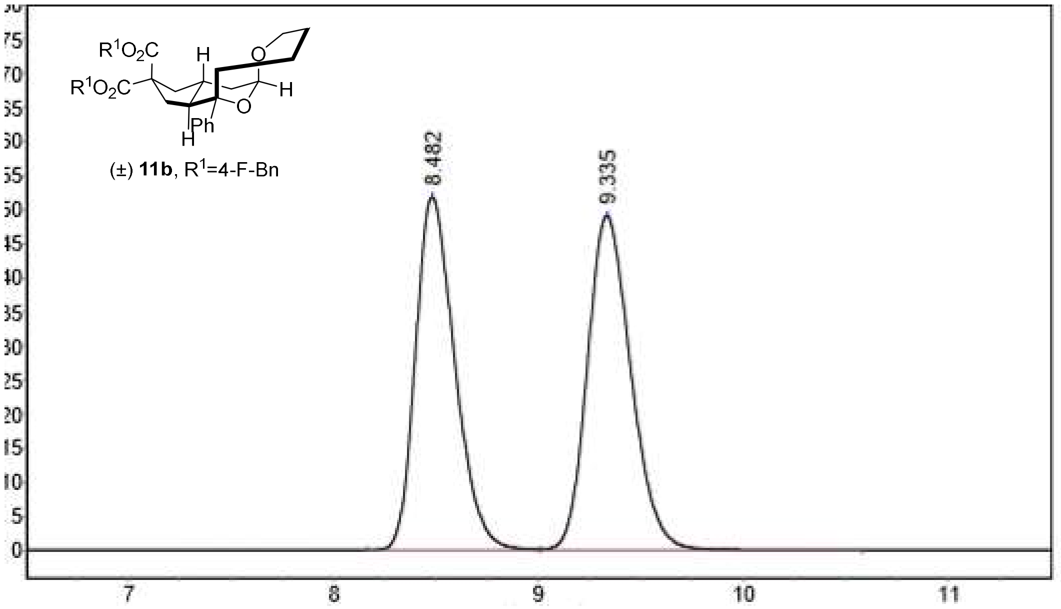

#	RT (time)	Heigh (mV*sec)	Area (mv)	Area (%)
1	8.482	51686.227	692009.063	48.7829
2	9.355	48976.230	726538.938	51.2171

Supplementary Figure 40. HPLC Trace of 11c.

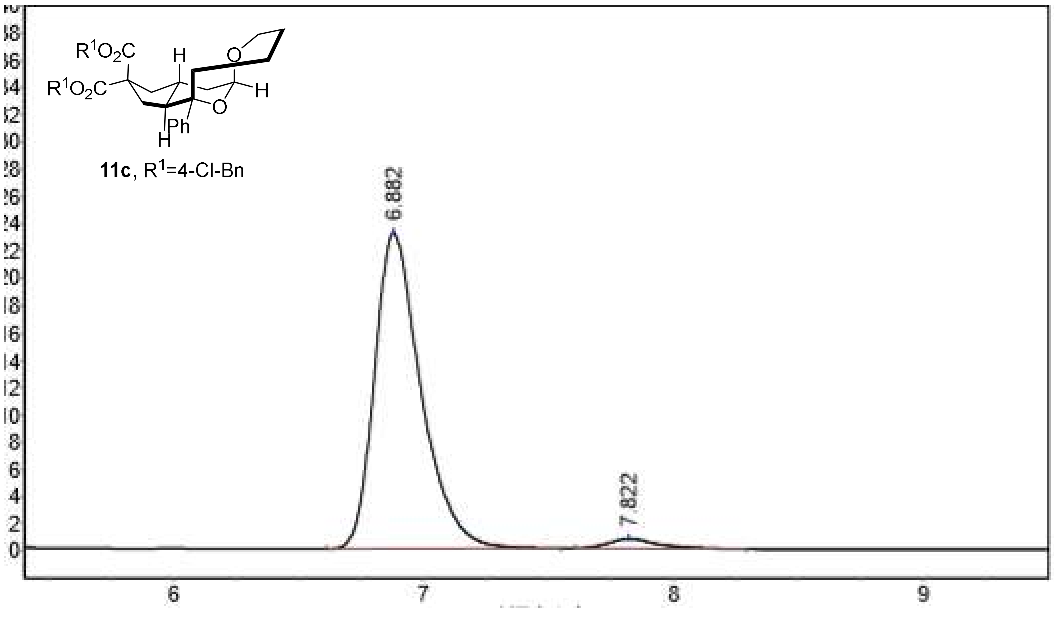

(min)

#	RT (time)	Heigh (mV*sec)	Area (mv)	Area (%)
1	6.882	23141.979	300265.313	96.7606
2	7.822	678.194	10052.600	3.2395

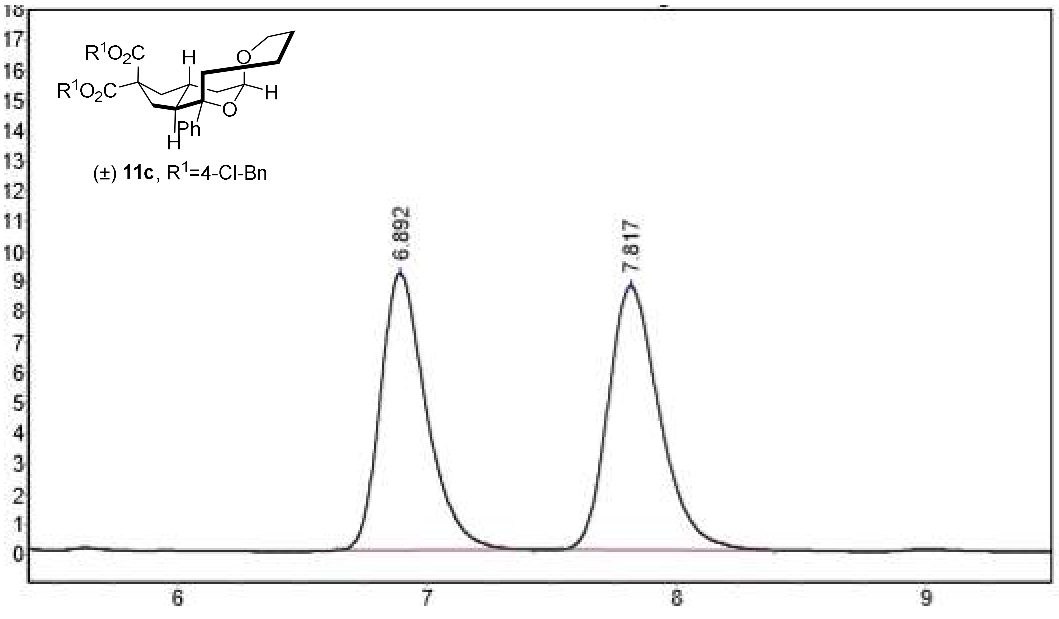

(min)

#	RT (time)	Heigh (mV*sec)	Area (mv)	Area (%)
1	6.892	9122.381	116346.953	48.3406
2	7.817	8683.532	124334.898	51.6594

Supplementary Figure 41. HPLC Trace of 11d.

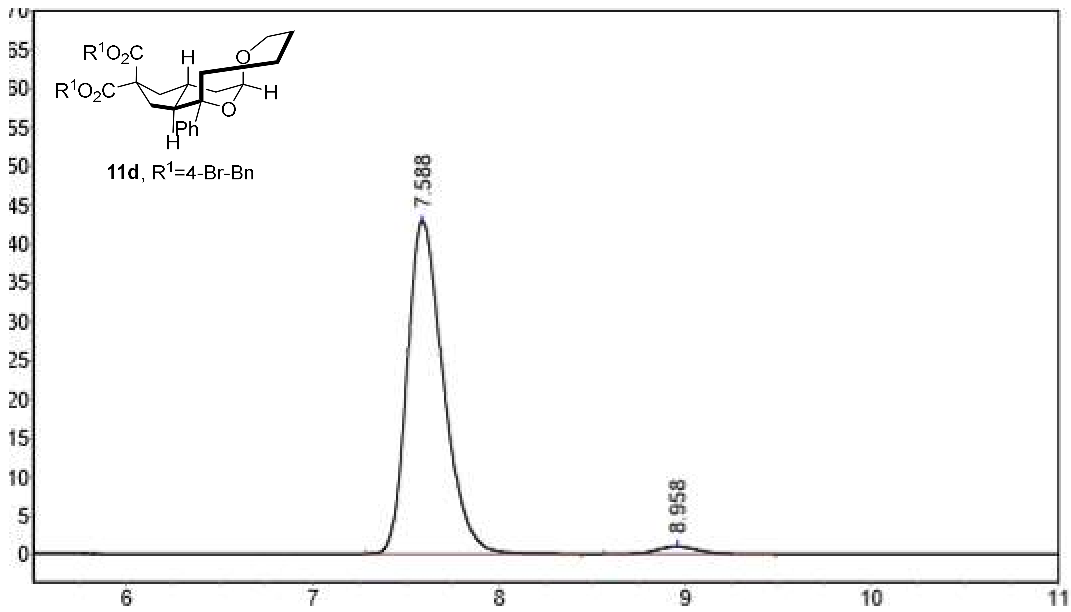

(min)

#	RT (time)	Heigh (mV*sec)	Area (mv)	Area (%)
1	7.588	42835.250	578729.125	97.3428
2	8.958	1018.986	15798.000	2.6572

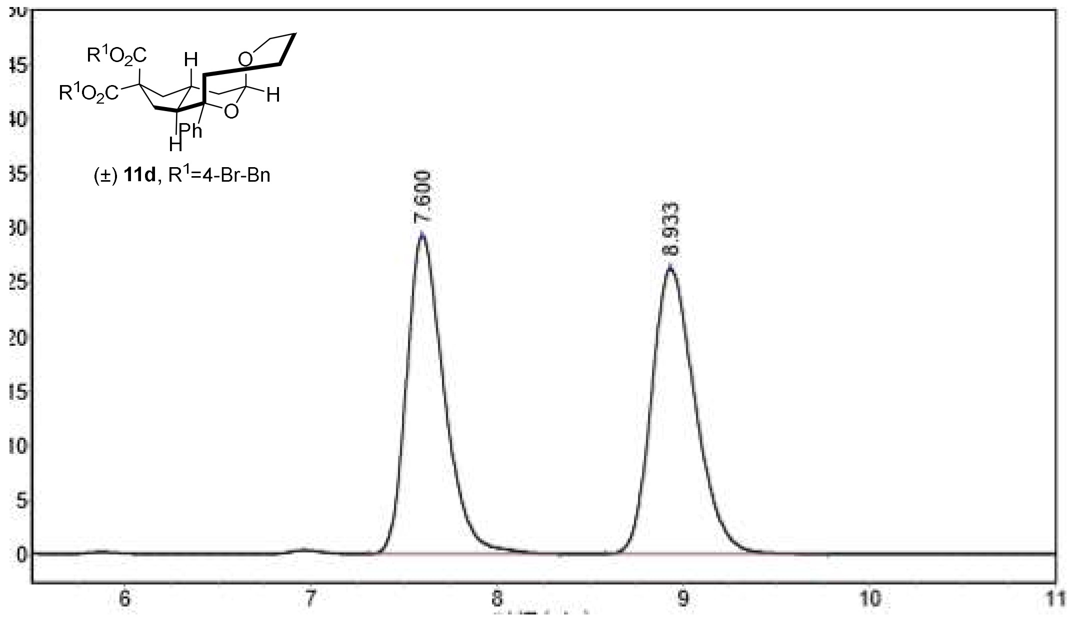

(min)

#	RT (time)	Heigh (mV*sec)	Area (mv)	Area (%)
1	7.600	29122.617	407531.938	48.9138
2	8.933	26134.049	425631.500	51.0862

Supplementary Figure 42. HPLC Trace of 11e.

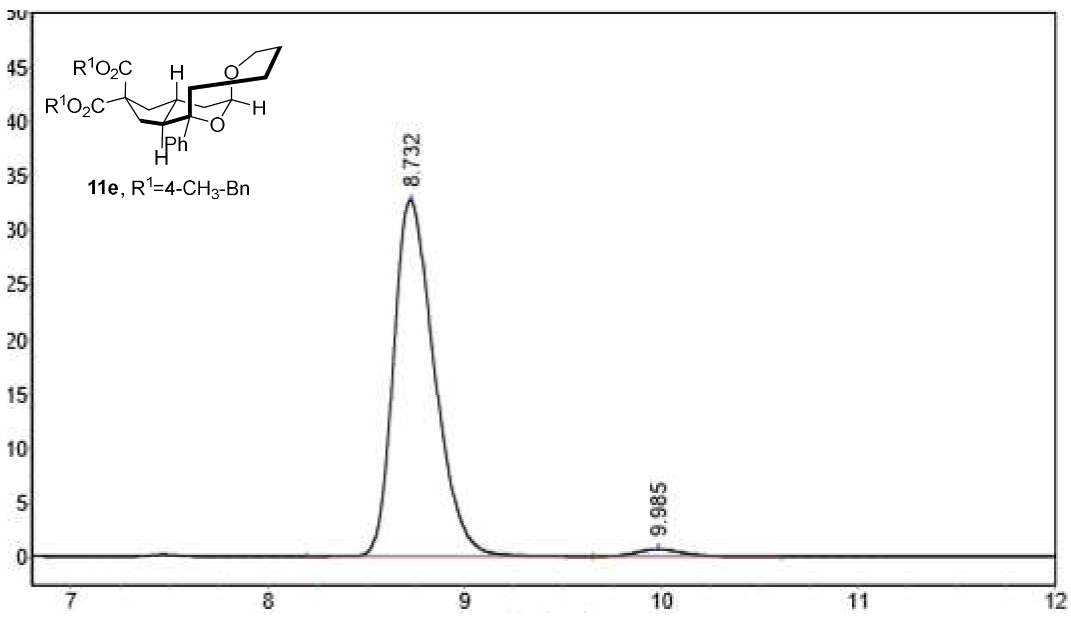

(min)

#	RT (time)	Heigh (mV*sec)	Area (mv)	Area (%)
1	8.732	32654.967	471733.219	97.5550
2	9.985	699.989	11823.179	2.4450

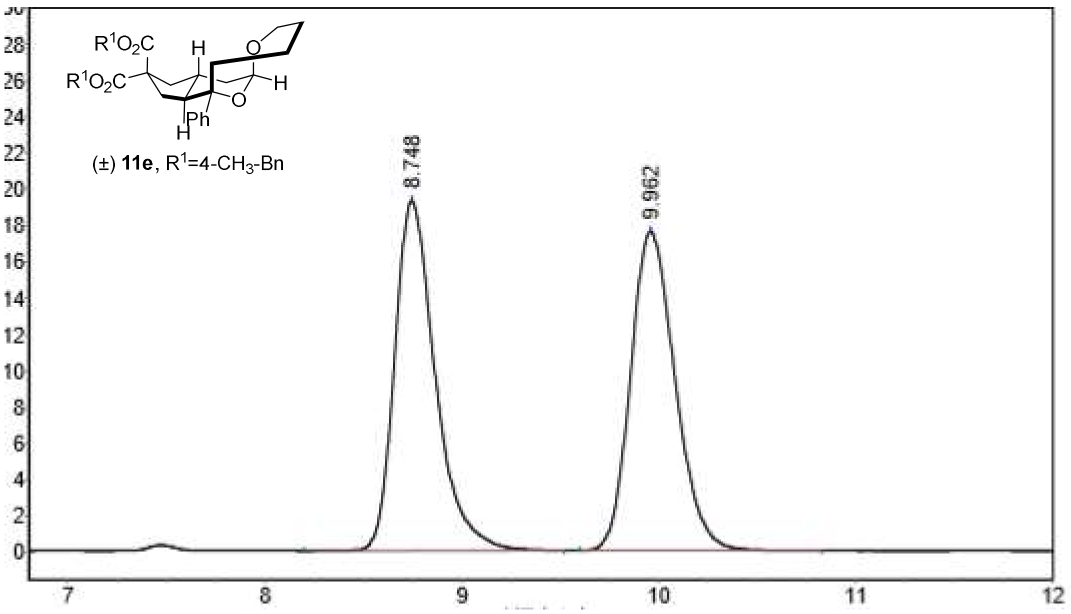

(min)

#	RT (time)	Heigh (mV*sec)	Area (mv)	Area (%)
1	8.748	19243.545	276861.906	50.0621
2	9.962	17533.467	276174.813	49.9379

Supplementary Figure 43. HPLC Trace of 11f.

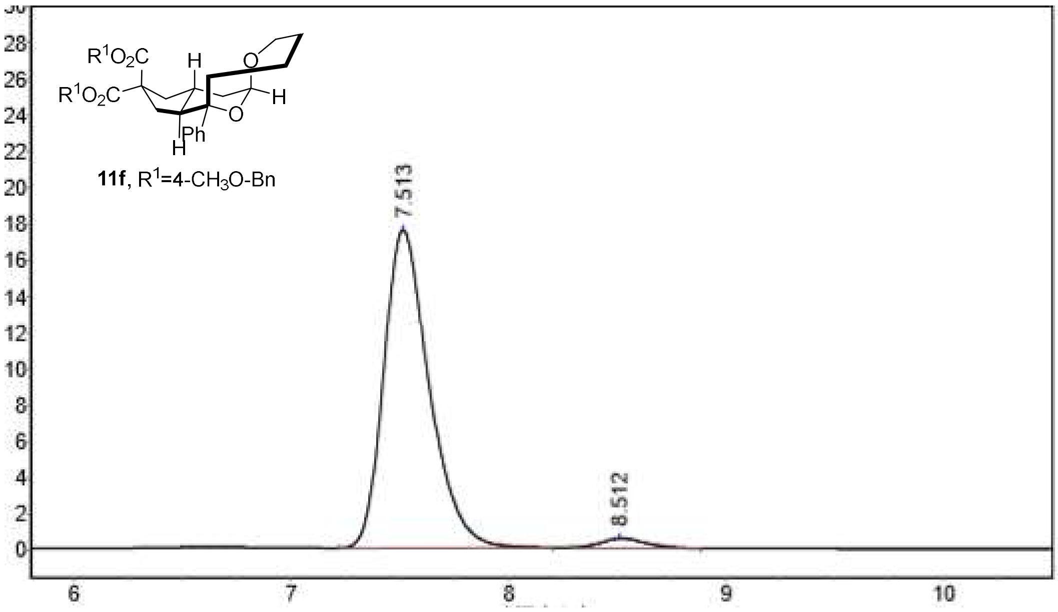

(min)

#	RT (time)	Heigh (mV*sec)	Area (mv)	Area (%)
1	7.513	17524.836	255932.406	96.9586
2	8.512	518.000	8028.000	3.0414

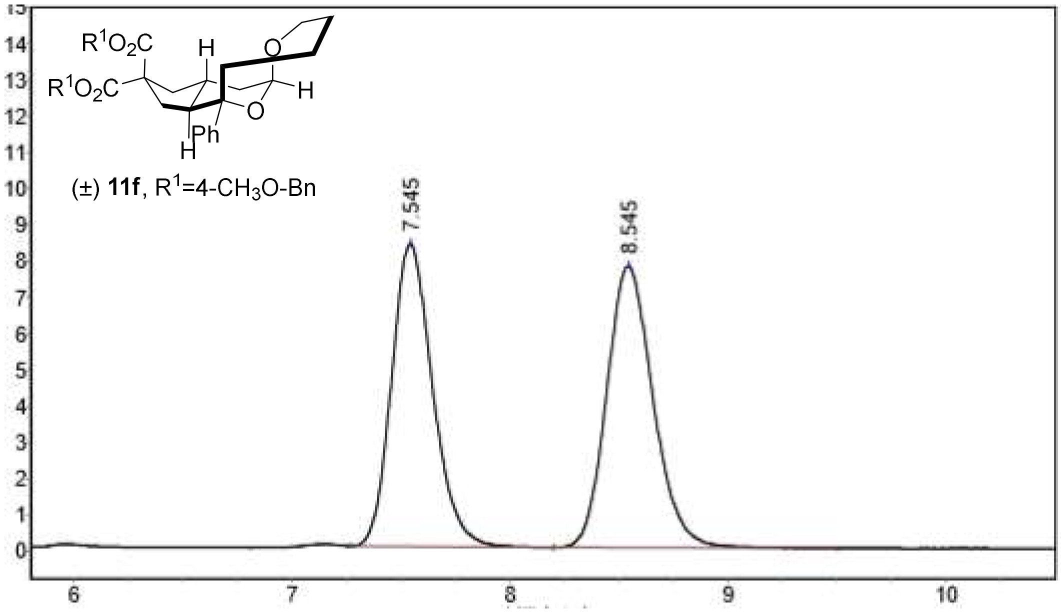

(min)

#	RT (time)	Heigh (mV*sec)	Area (mv)	Area (%)
1	7.545	8349.405	109938.484	48.8571
2	8.545	7739.708	115081.898	51.1429

Supplementary Figure 44. HPLC Trace of 11g.

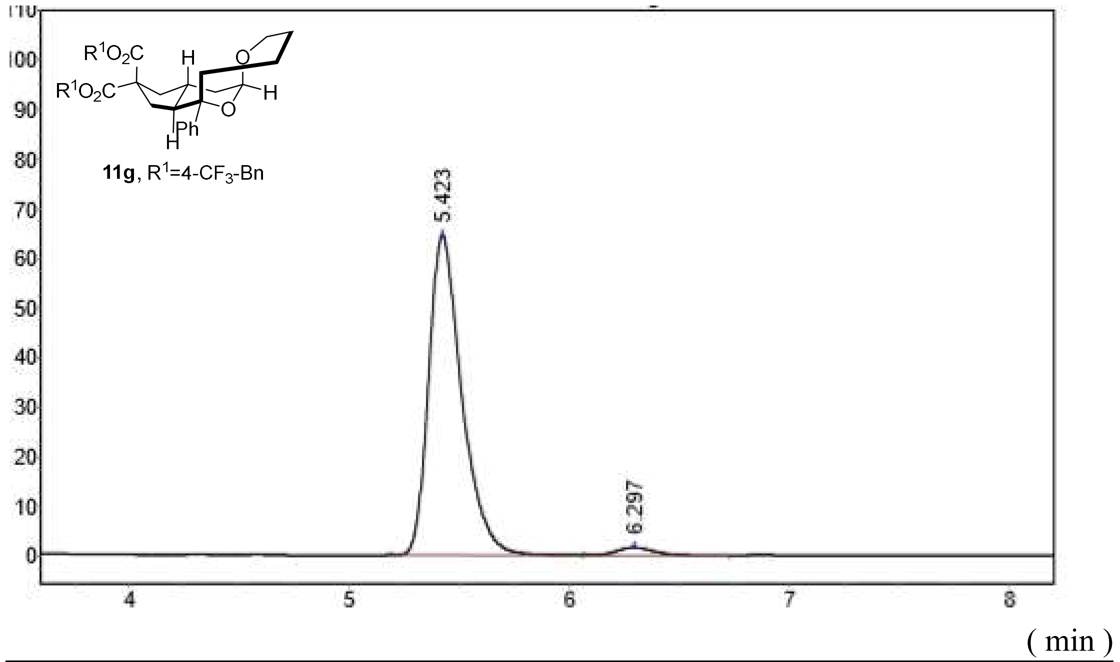

#	RT (time)	Heigh (mV*sec)	Area (mv)	Area (%)
1	5.423	64504.789	675513.250	97.1657
2	6.297	1672.394	19704.496	2.8343

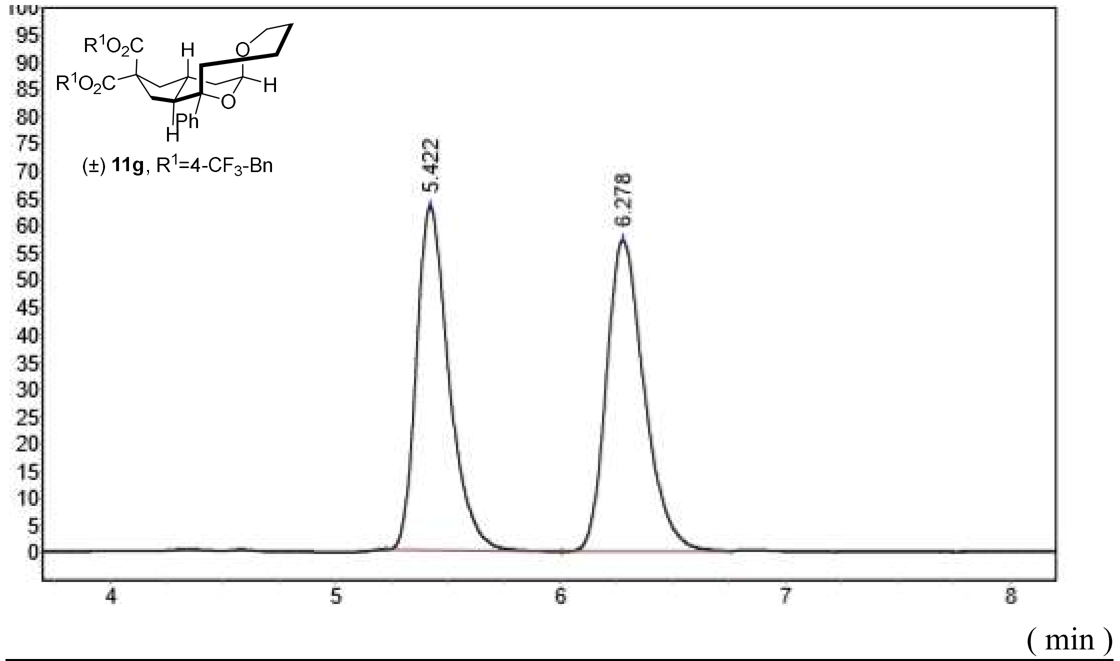

#	RT (time)	Heigh (mV*sec)	Area (mv)	Area (%)
1	5.422	63160.543	633363.313	48.5419
2	6.278	57211.840	671413.438	51.4581

Supplementary Figure 45. HPLC Trace of 11h.

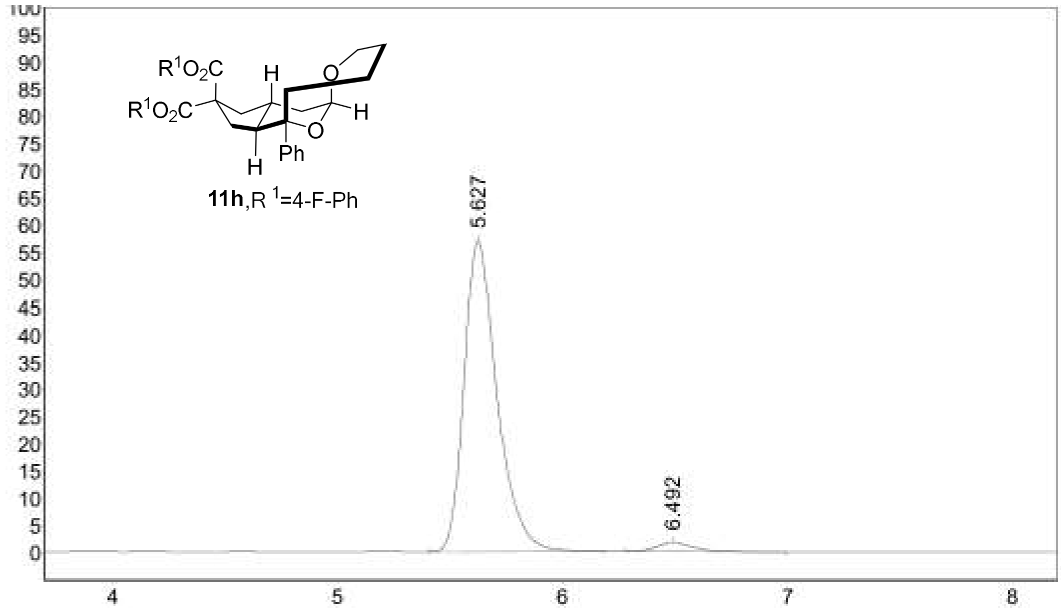

(min)

#	RT (time)	Heigh (mV*sec)	Area (mv)	Area (%)
1	5.627	56962.402	583418.625	96.8055
2	6.492	1699.185	19252.000	3.1944

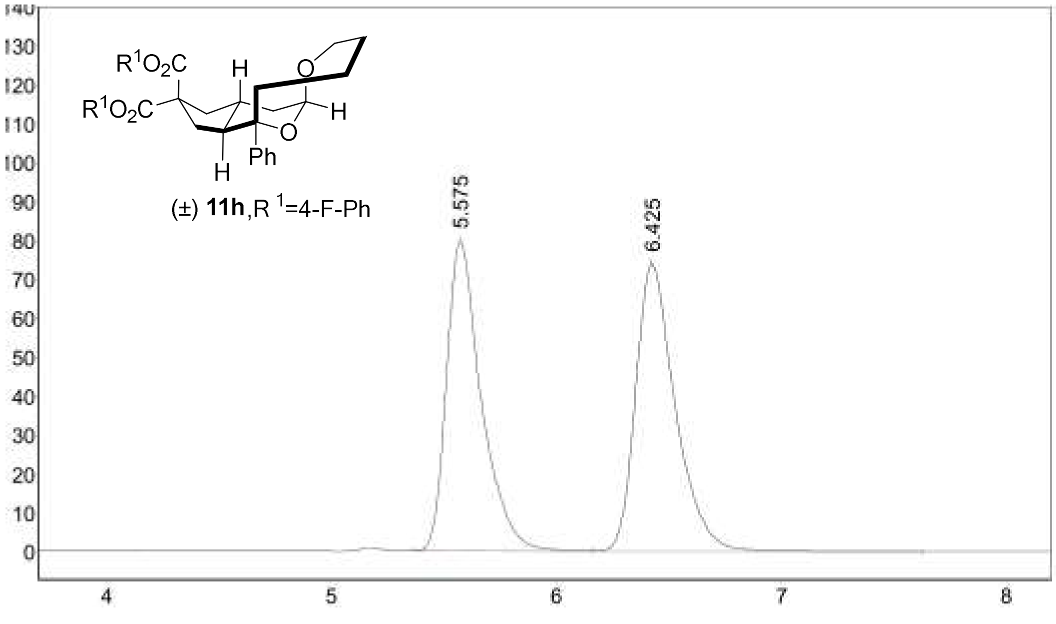

(min)

#	RT (time)	Heigh (mV*sec)	Area (mv)	Area (%)
1	5.575	79547.141	858080.813	48.6771
2	6.425	73598.266	904719.688	51.3229

Supplementary Figure 46. HPLC Trace of 11i.

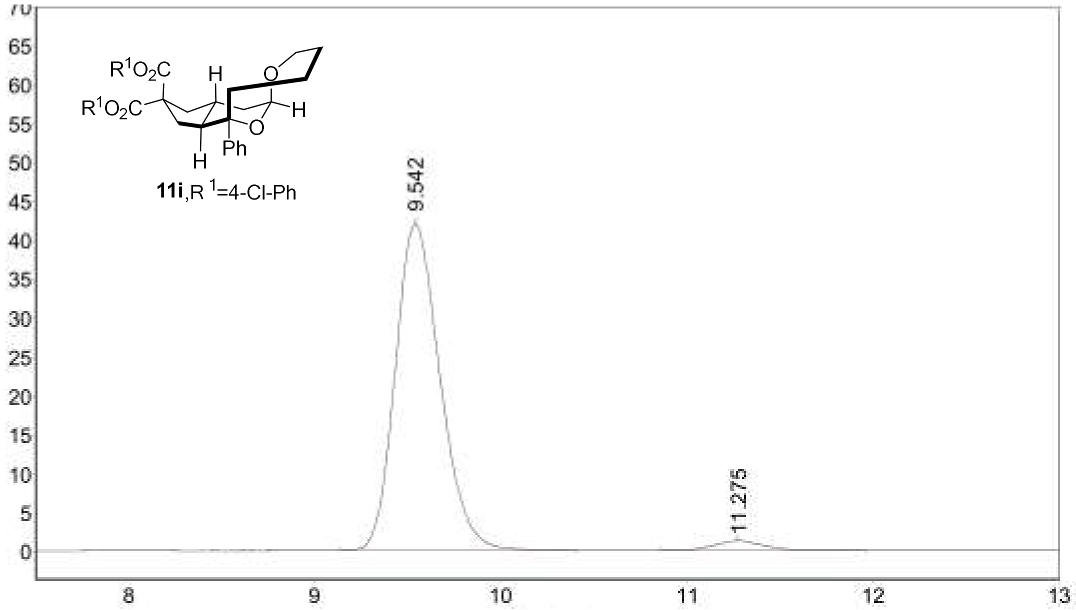

(min)

#	RT (time)	Heigh (mV*sec)	Area (mv)	Area (%)
1	9.542	41939.211	703192.688	96.8327
2	11.275	1201.867	23000.900	3.1673

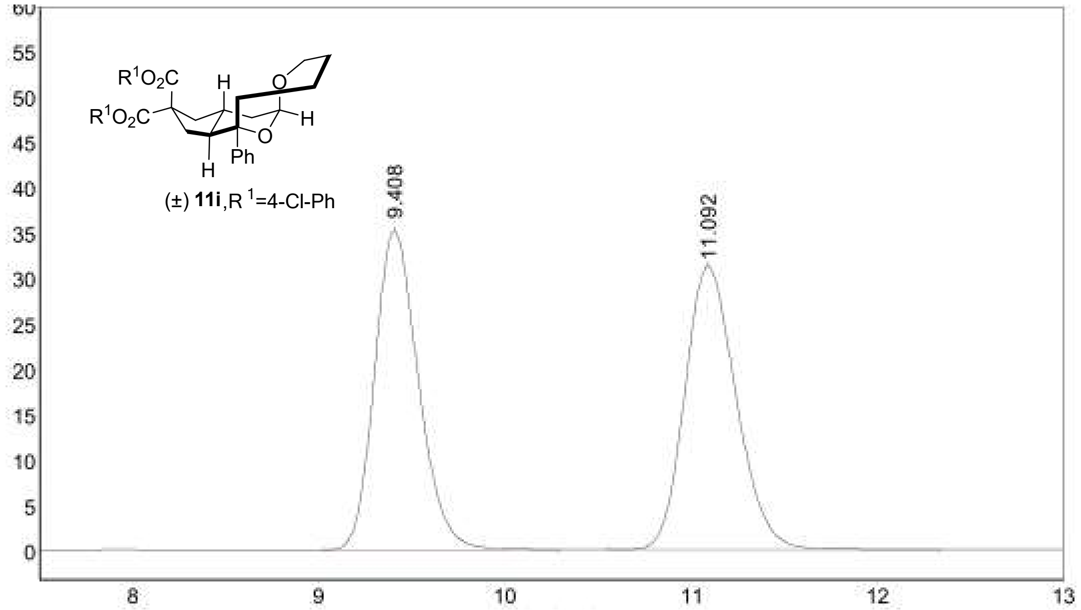

(min)

#	RT (time)	Heigh (mV*sec)	Area (mv)	Area (%)
1	9.408	35085.434	576964.875	49.0874
2	11.092	31103.963	598416.813	50.9125

Supplementary Figure 47. HPLC Trace of 11j.

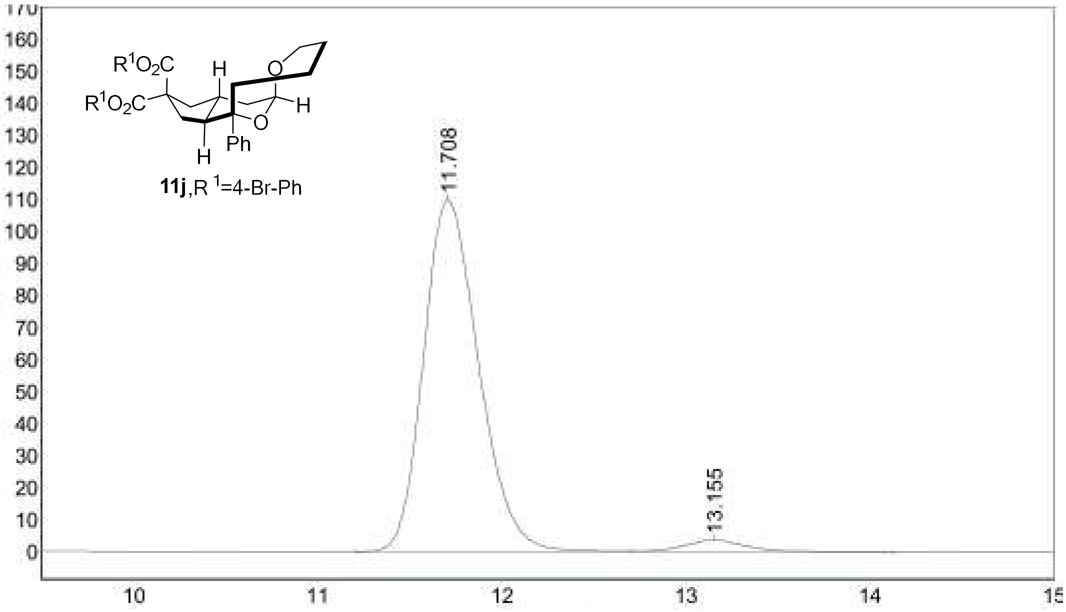

(min)

#	RT (time)	Heigh (mV*sec)	Area (mv)	Area (%)
1	11.708	109651.313	2283687.500	96.6851
2	13.155	3405.332	78298.070	3.3149

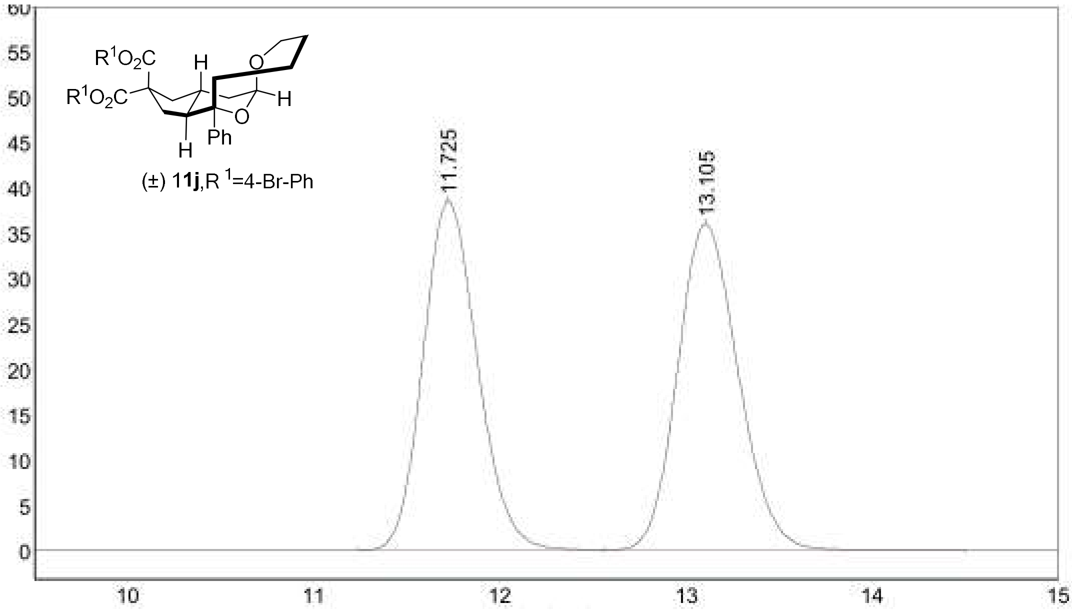

(min)

#	RT (time)	Heigh (mV*sec)	Area (mv)	Area (%)
1	11.725	38480.043	786205.875	48.9788
2	13.105	35897.000	818990.063	51.0212

Supplementary Figure 48. HPLC Trace of 11k.

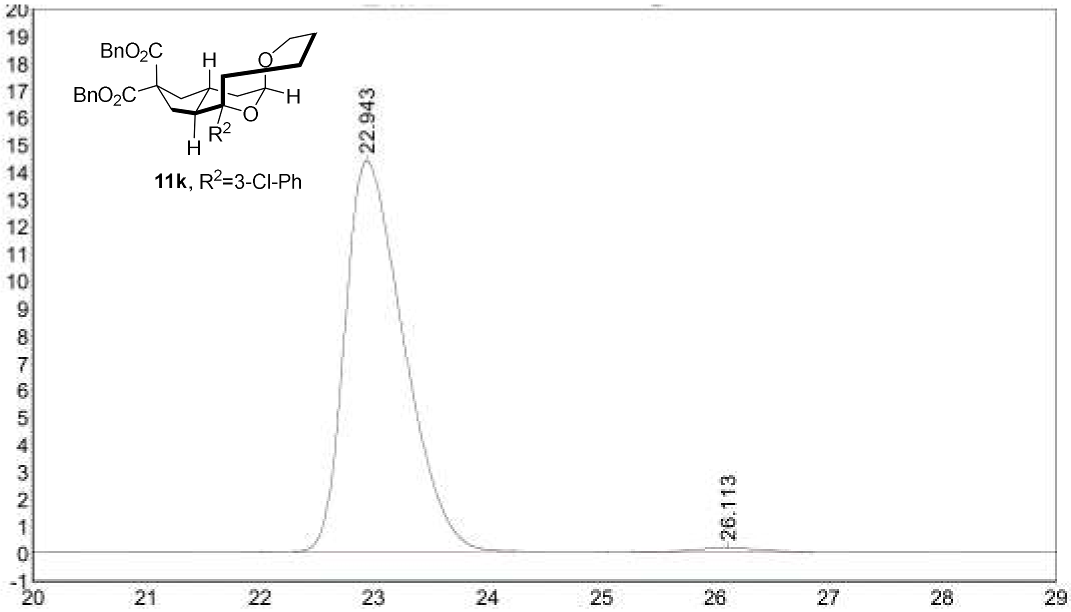

(min)

#	RT (time)	Heigh (mV*sec)	Area (mv)	Area (%)
1	22.943	14356.430	515117.688	98.8506
2	26.113	158.810	5989.600	1.1494

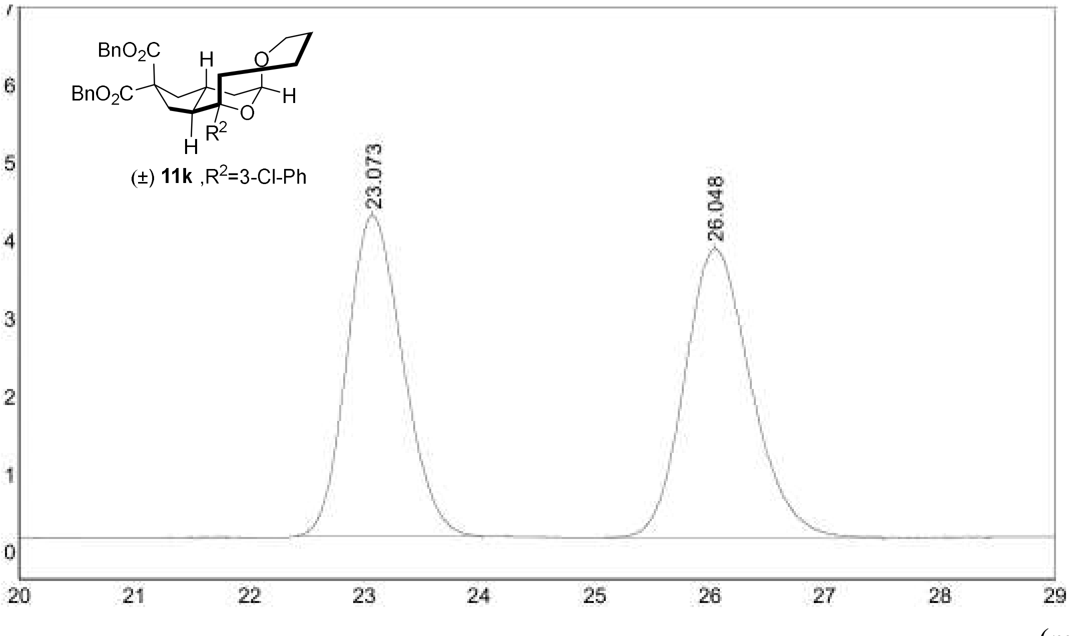

(min)

#	RT (time)	Heigh (mV*sec)	Area (mv)	Area (%)
1	23.073	4115.016	139158.797	48.2719
2	26.048	3707.825	149122.406	51.7281

Supplementary Figure 49. HPLC Trace of 11l.

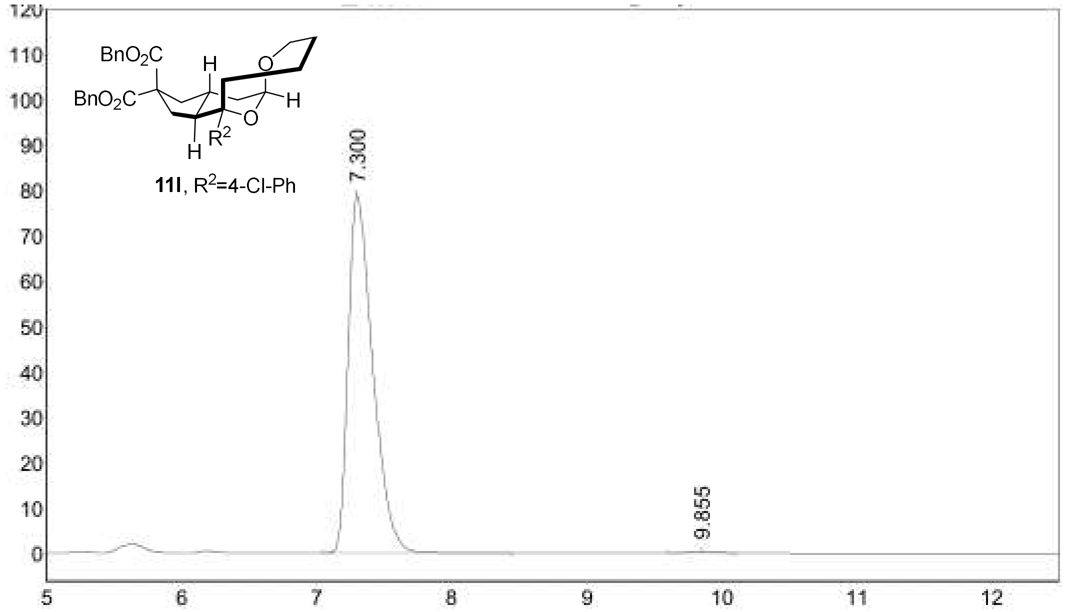

(min)

#	RT (time)	Heigh (mV*sec)	Area (mv)	Area (%)
1	7.300	78805.625	1001150.875	99.4729
2	9.855	360.000	5304.700	0.5271

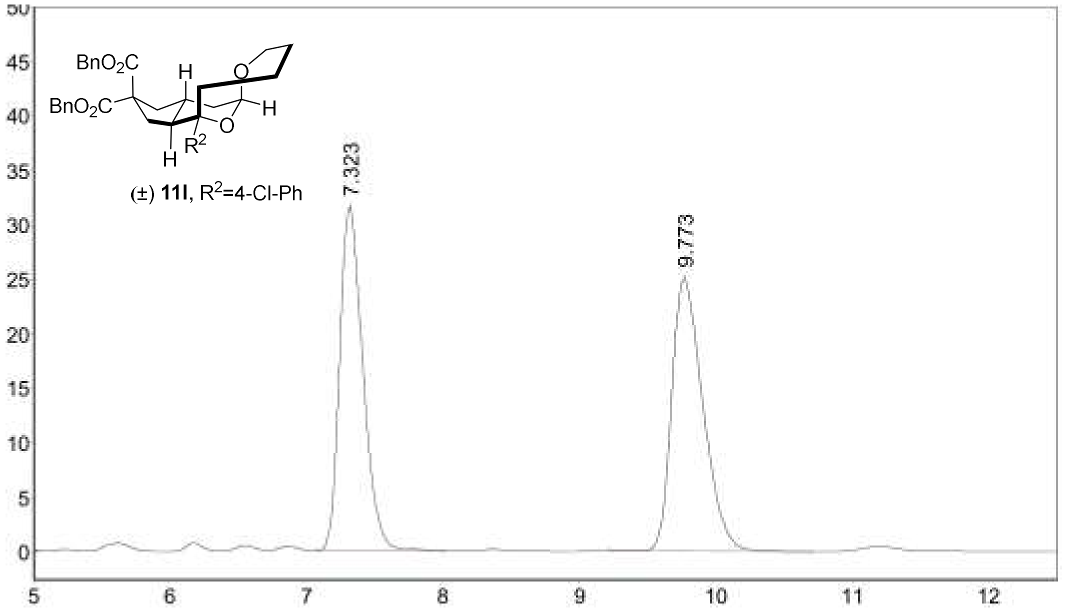

(min)

#	RT (time)	Heigh (mV*sec)	Area (mv)	Area (%)
1	7.323	31488.750	375734.438	48.4781
2	9.773	24959.006	399326.188	51.5219

Supplementary Figure 50. HPLC Trace of 11m.

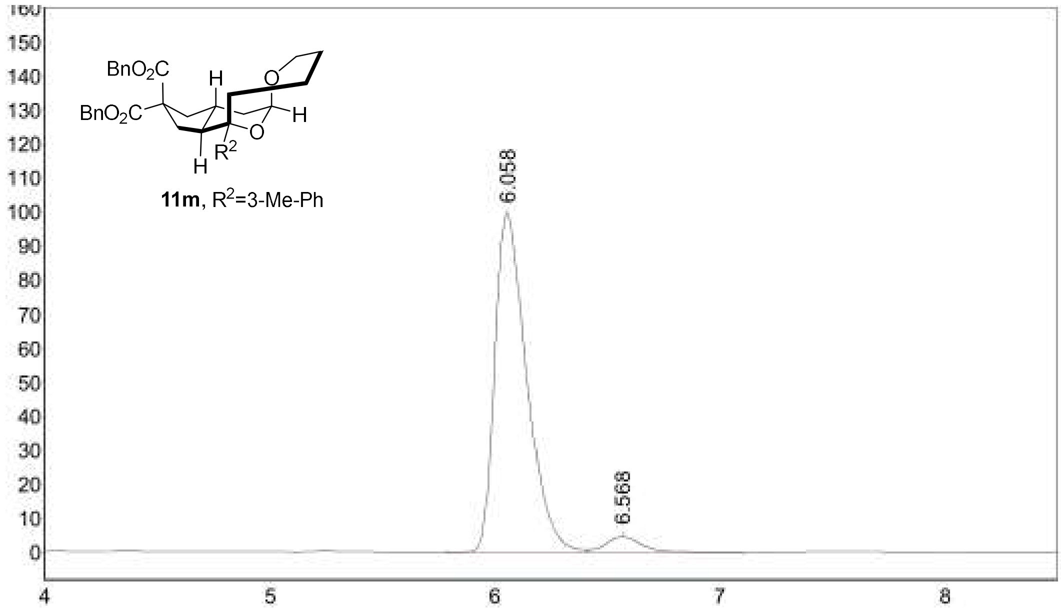

#	RT (time)	Heigh (mV*sec)	Area (mv)	Area (%)
1	6.058	99036.125	975537.063	95.3517
2	6.568	448.908	47555.988	4.6483

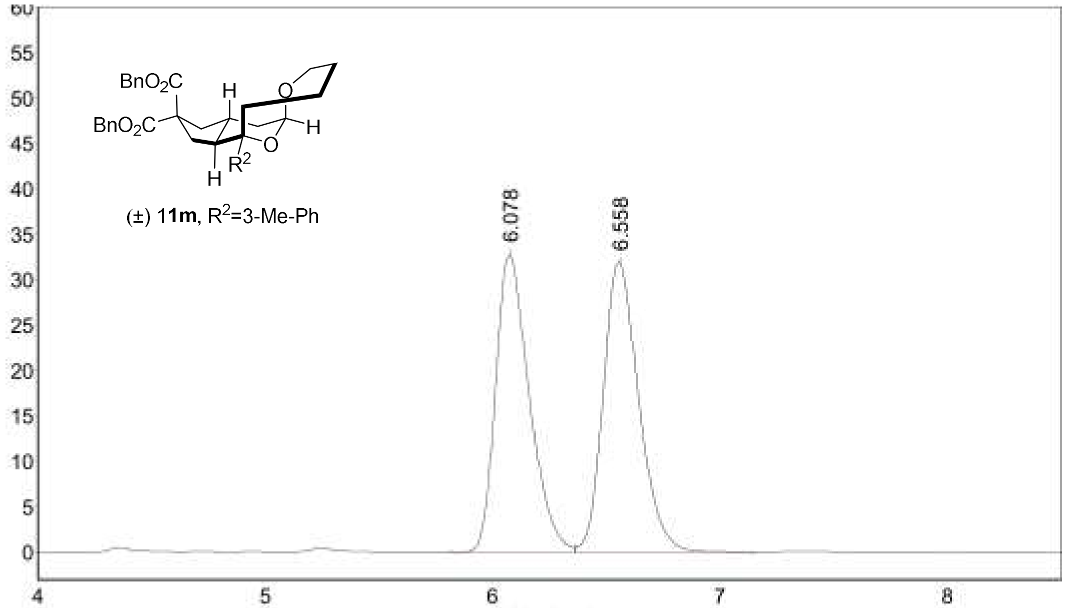

#	RT (time)	Heigh (mV*sec)	Area (mv)	Area (%)
1	6.078	32730.631	335988.313	49.1928
2	6.558	31860.105	347015.343	50.8072

Supplementary Figure 51. HPLC Trace of 11n.

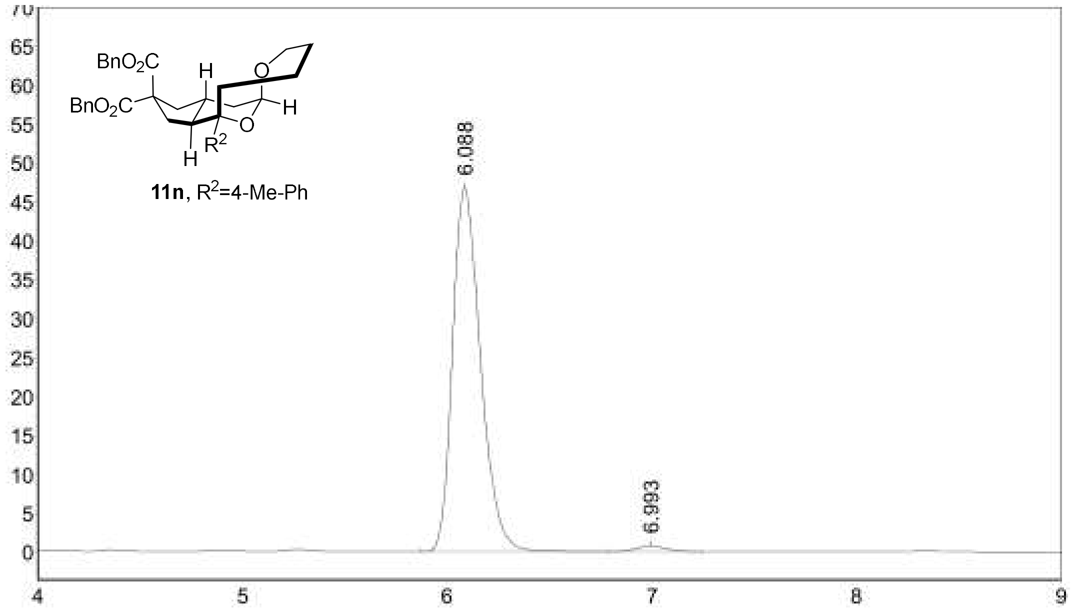

(min)

#	RT (time)	Heigh (mV*sec)	Area (mv)	Area (%)
1	6.088	46735.824	445419.094	98.3608
2	6.993	726.714	7422.950	1.6392

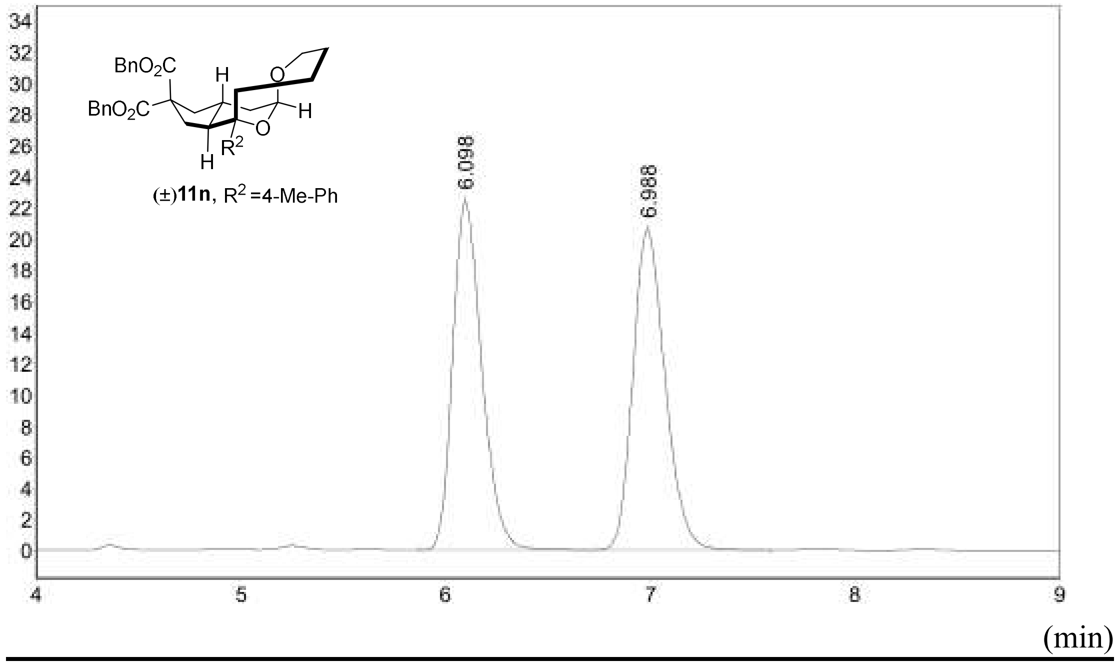

(min)

#	RT (time)	Heigh (mV*sec)	Area (mv)	Area (%)
1	6.098	22349.379	221624.000	49.6601
2	6.988	20481.586	224657.406	50.3399

Supplementary Figure 52. HPLC Trace of 11o.

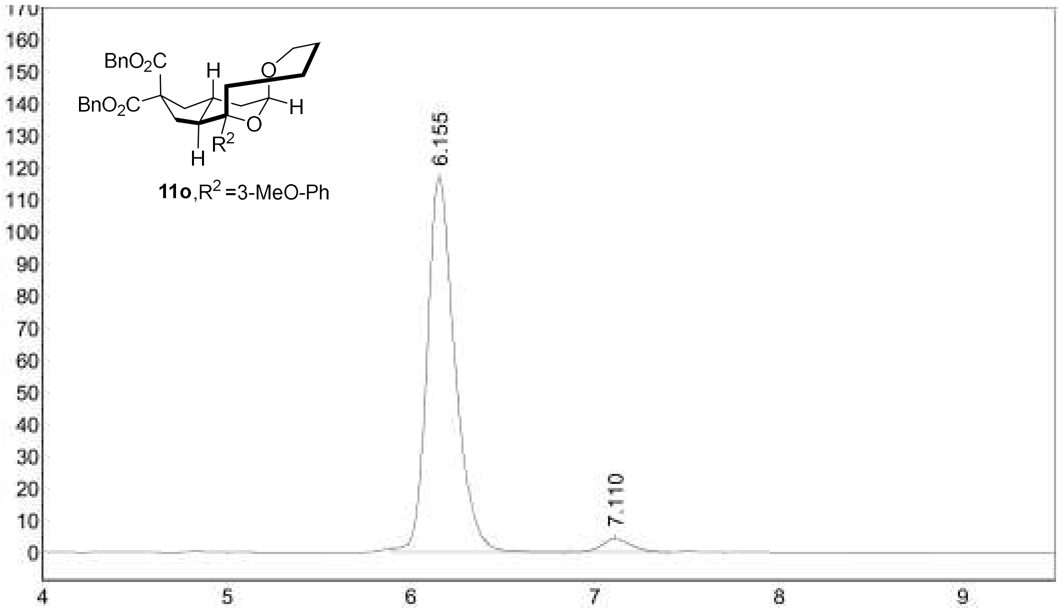

(min)

#	RT (time)	Heigh (mV*sec)	Area (mv)	Area (%)
1	6.155	116380.000	1228598.750	96.4137
2	7.110	3947.000	45699.699	3.5863

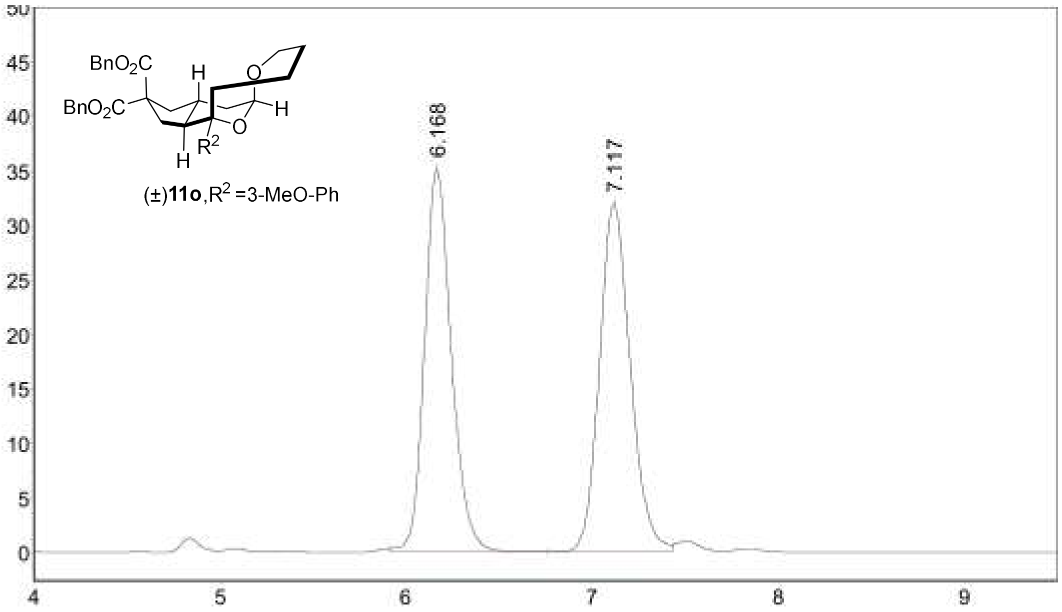

(min)

#	RT (time)	Heigh (mV*sec)	Area (mv)	Area (%)
1	6.168	35052.000	353265.094	48.8436
2	7.117	31821.000	369993.313	51.1565

Supplementary Figure 53. HPLC Trace of 11p.

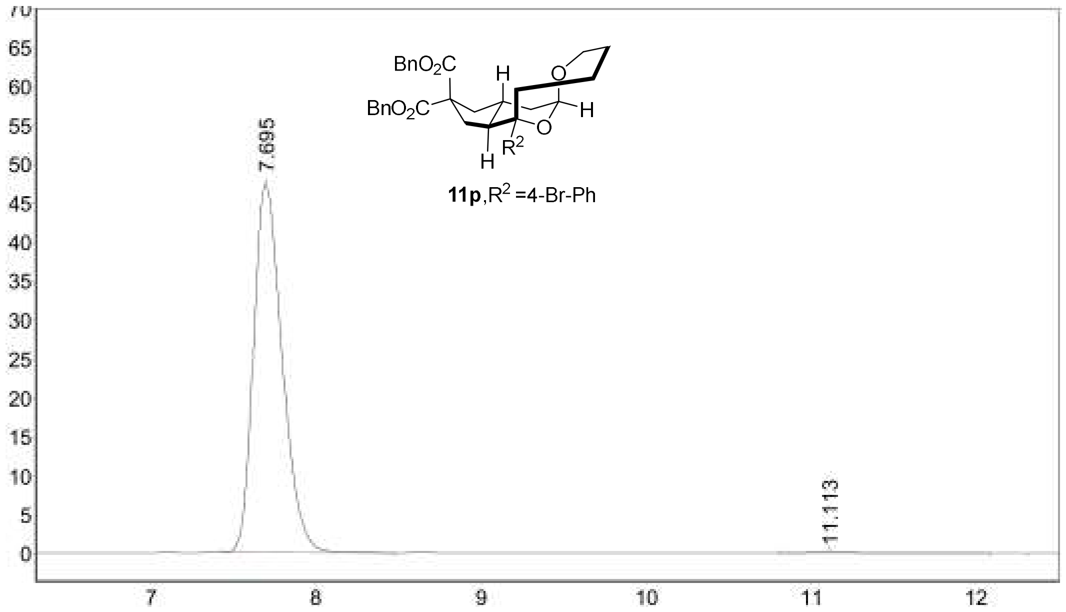

(min)

#	RT (time)	Heigh (mV*sec)	Area (mv)	Area (%)
1	7.695	47265.000	571960.625	99.3661
2	11.113	190.668	3649.025	0.6339

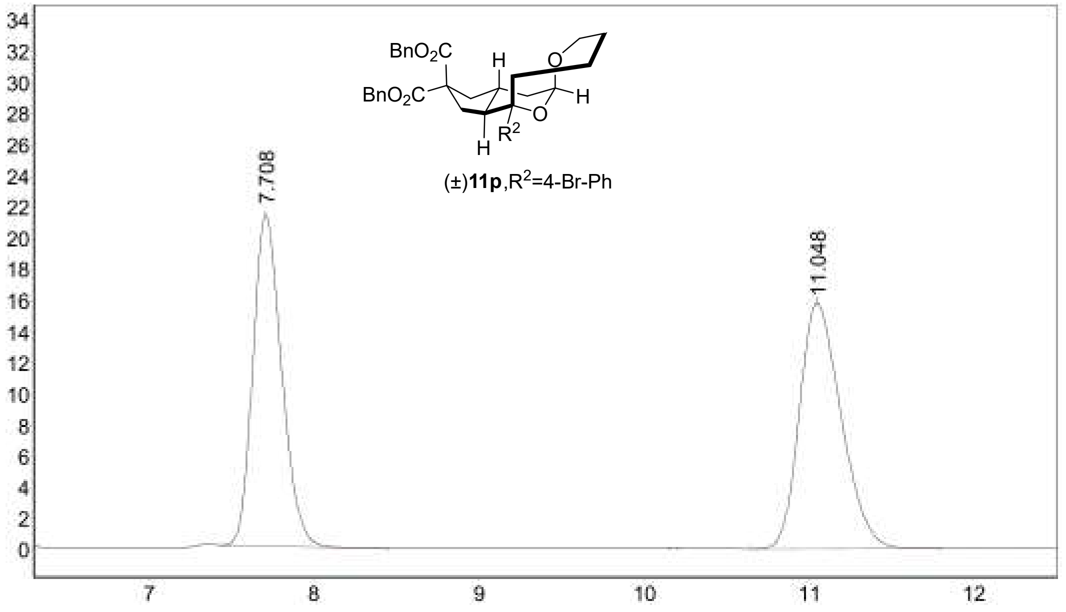

(min)

#	RT (time)	Heigh (mV*sec)	Area (mv)	Area (%)
1	7.708	21294.555	263482.656	48.6857
2	11.048	15760.000	277707.813	51.3142

Supplementary Figure 54. HPLC Trace of 12.

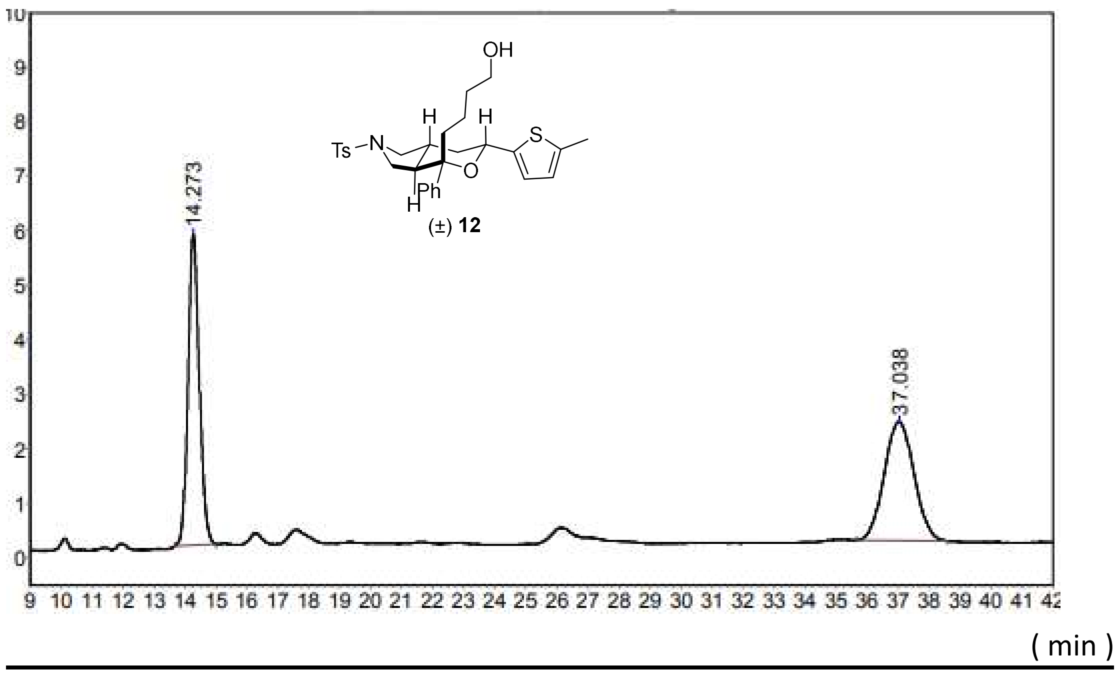

#	RT (time)	Heigh (mV*sec)	Area (mv)	Area (%)
1	14.273	5696.362	144991.703	49.7701
2	37.038	2187.060	146331.406	50.2299

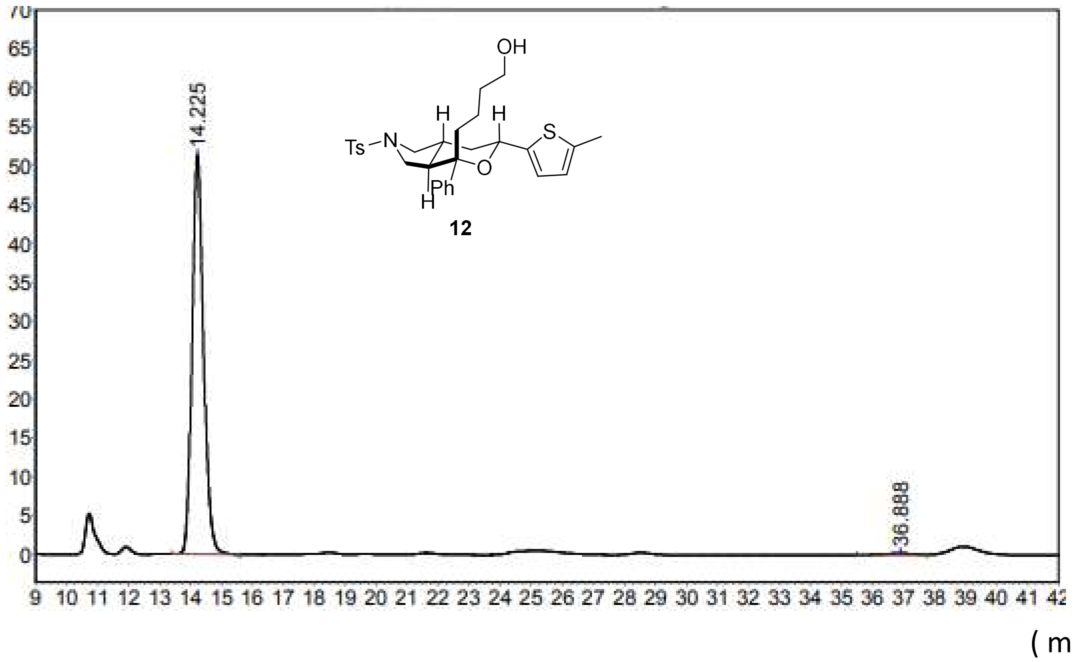

#	RT (time)	Heigh (mV*sec)	Area (mv)	Area (%)
1	14.225	51217.250	1327800.875	98.7664
2	36.888	245.998	16584.514	1.2336

Supplementary Figure 55. HPLC Trace of 13.

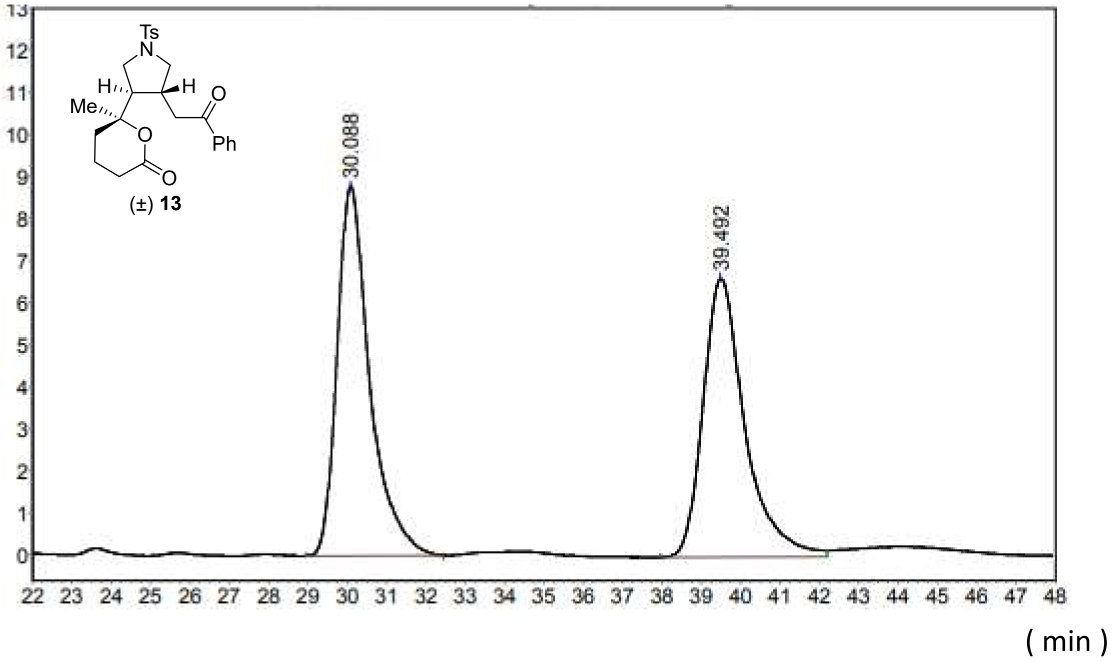

#	RT (time)	Heigh (mV*sec)	Area (mv)	Area (%)
1	30.088	8785.856	507828.188	50.8947
2	39.492	6616.829	489973.188	49.1053

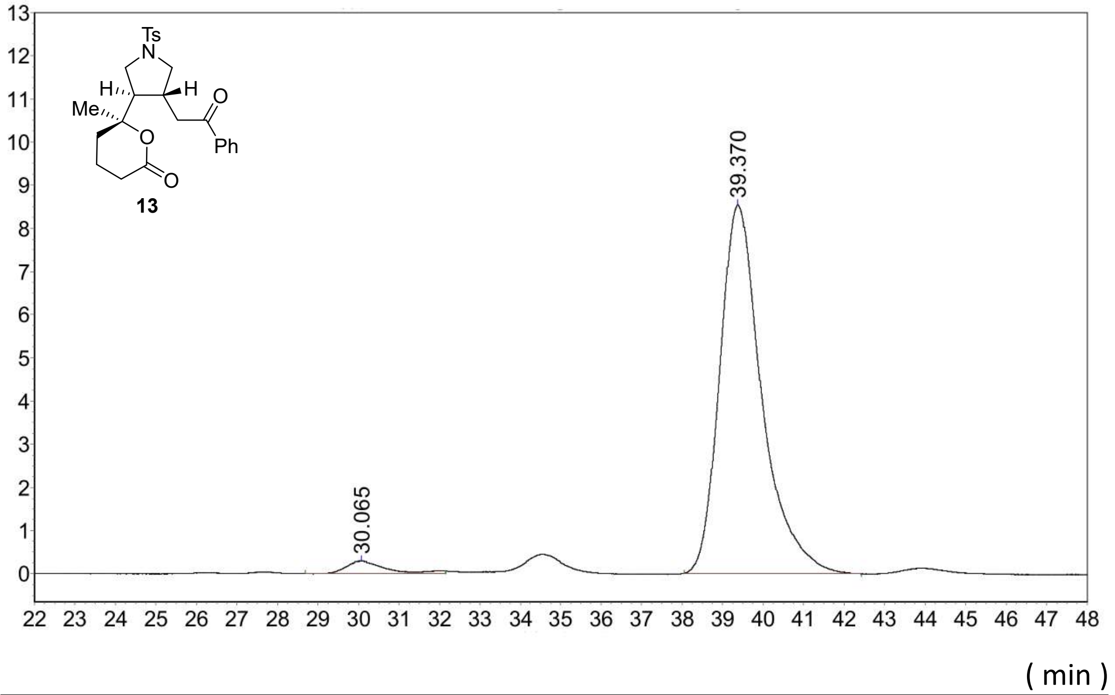

#	RT (time)	Heigh (mV*sec)	Area (mv)	Area (%)
1	30.065	289.000	20771.000	3.2600
2	39.370	8533.952	616383.438	96.7400

Supplementary Figure 56. HPLC Trace of 14.

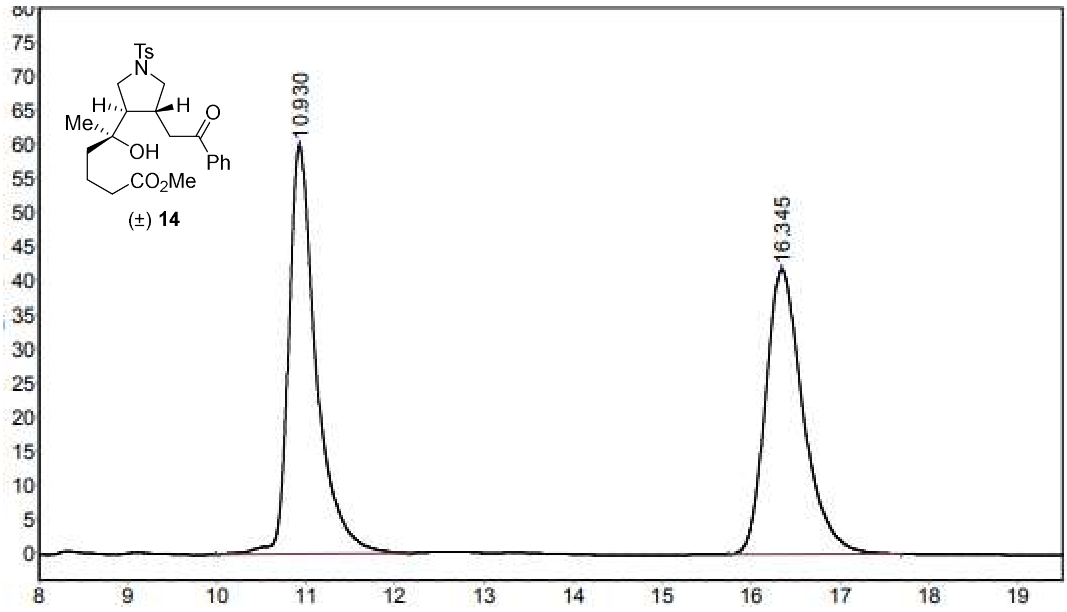

(min)

#	RT (time)	Heigh (mV*sec)	Area (mv)	Area (%)
1	10.930	59896.027	1292338.250	51.2300
2	16.345	41693.930	1230281.500	48.7700

(min)

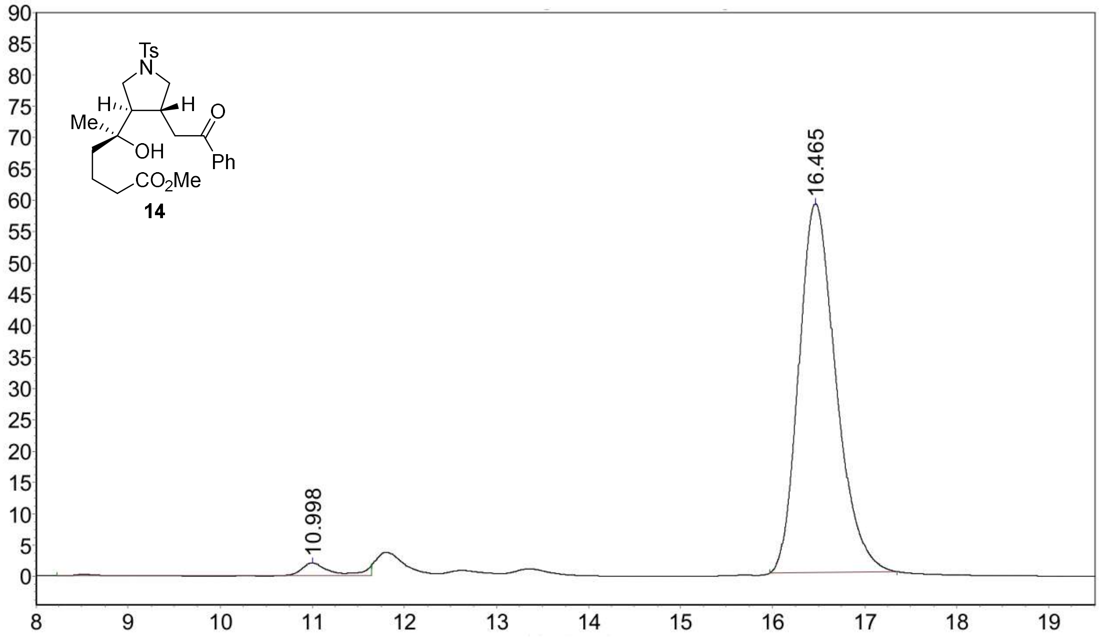

#	RT (time)	Heigh (mV*sec)	Area (mv)	Area (%)
1	10.998	2022.000	55397.305	3.2531
2	16.465	58748.246	1647489.625	96.7469

Supplementary Figure 57. HPLC Trace of 15.

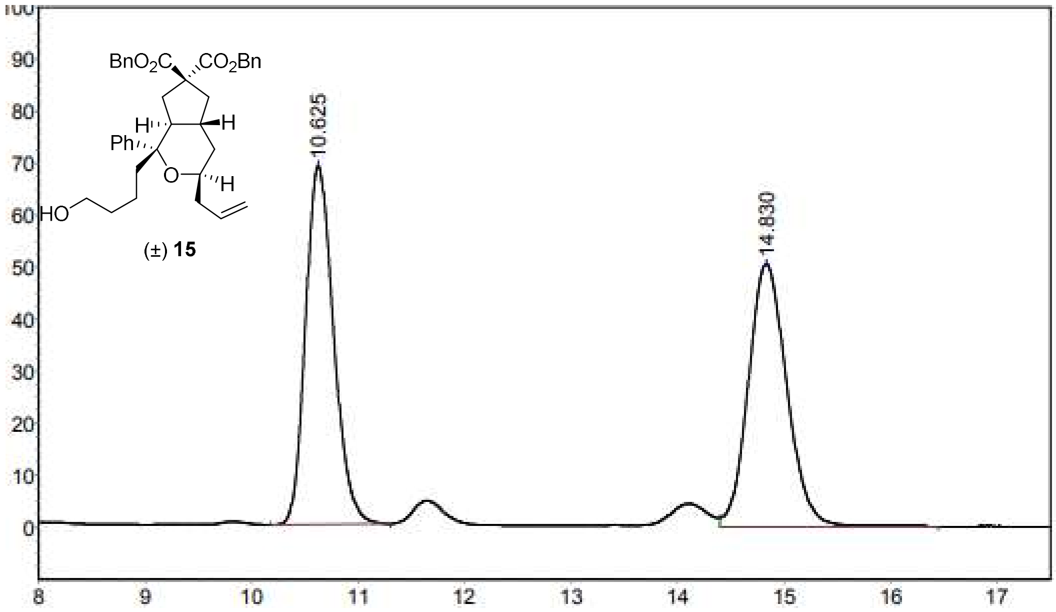

#	RT (time)	Heigh (mV*sec)	Area (mv)	Area (%)
1	10.625	68898.828	1266957.625	50.1303
2	14.830	50305.000	1260369.125	49.8697

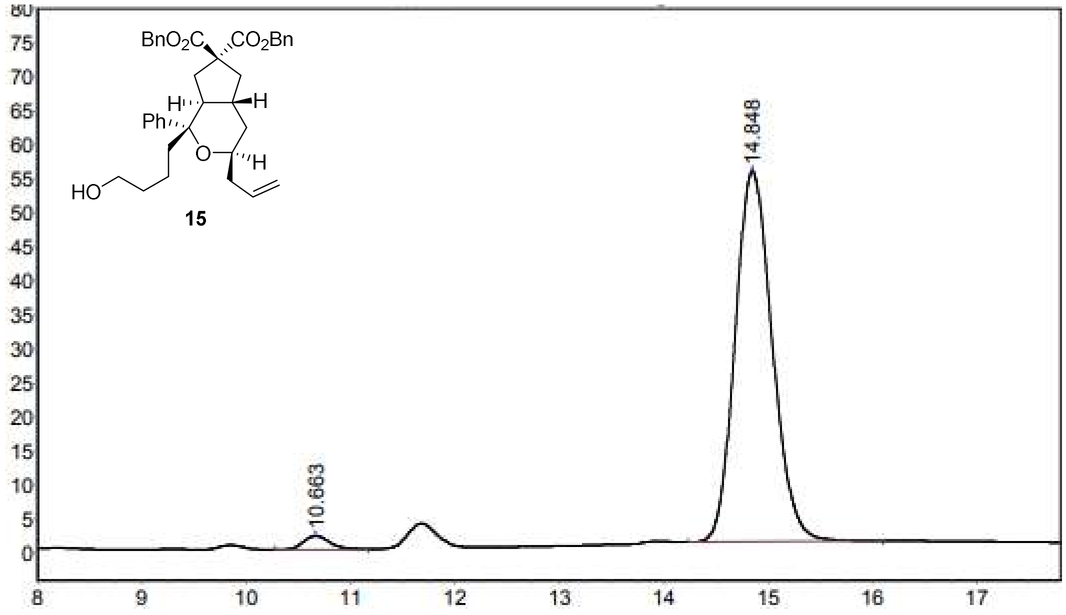

#	RT (time)	Heigh (mV*sec)	Area (mv)	Area (%)
1	10.663	2001.000	37246.797	2.6811
2	14.848	54490.359	1351984.875	97.3189

Supplementary Figure 58. HPLC Trace of 17.

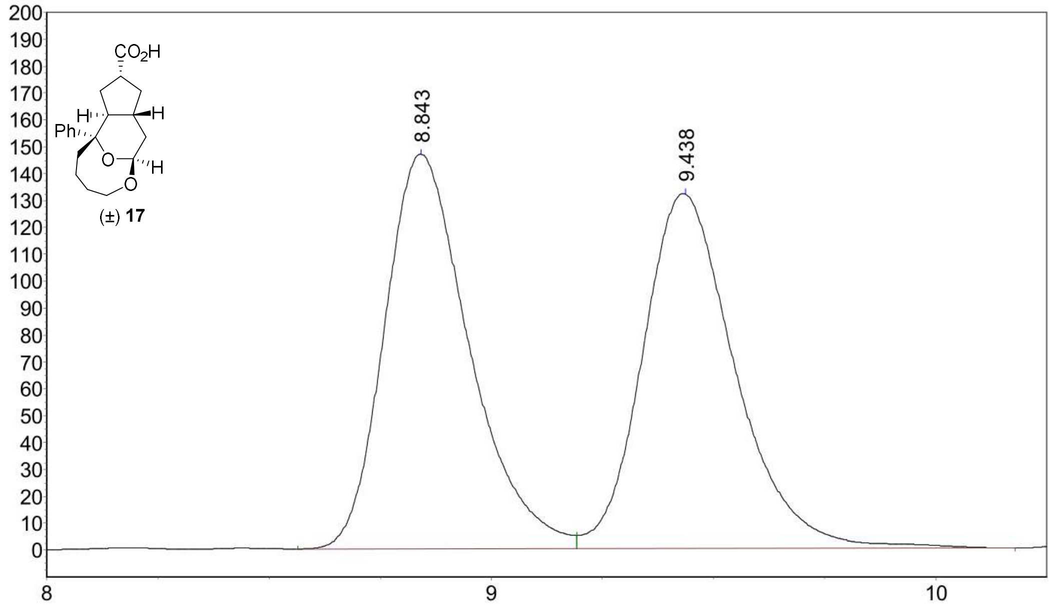

#	RT (time)	Heigh (mV*sec)	Area (mv)	Area (%)
1	8.843	146760.000	1949628.500	50.4851
2	9.438	132011.359	1912159.875	49.5149

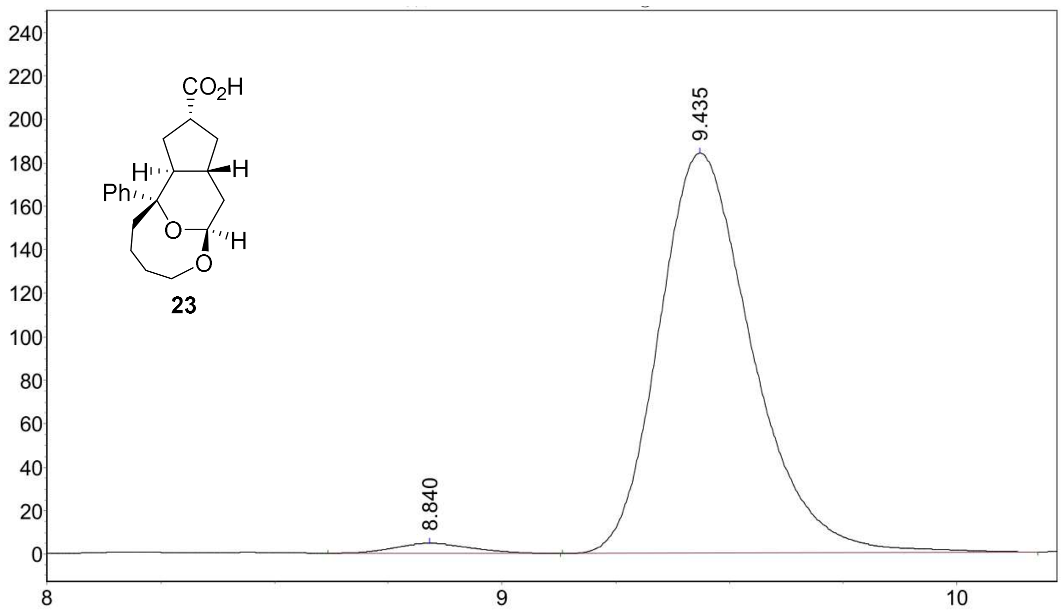

#	RT (time)	Heigh (mV*sec)	Area (mv)	Area (%)
1	8.840	4659.000	59222.402	2.2119
2	9.435	184005.516	2618194.750	97.7881

Supplementary Figure 59. HPLC Trace of 19.

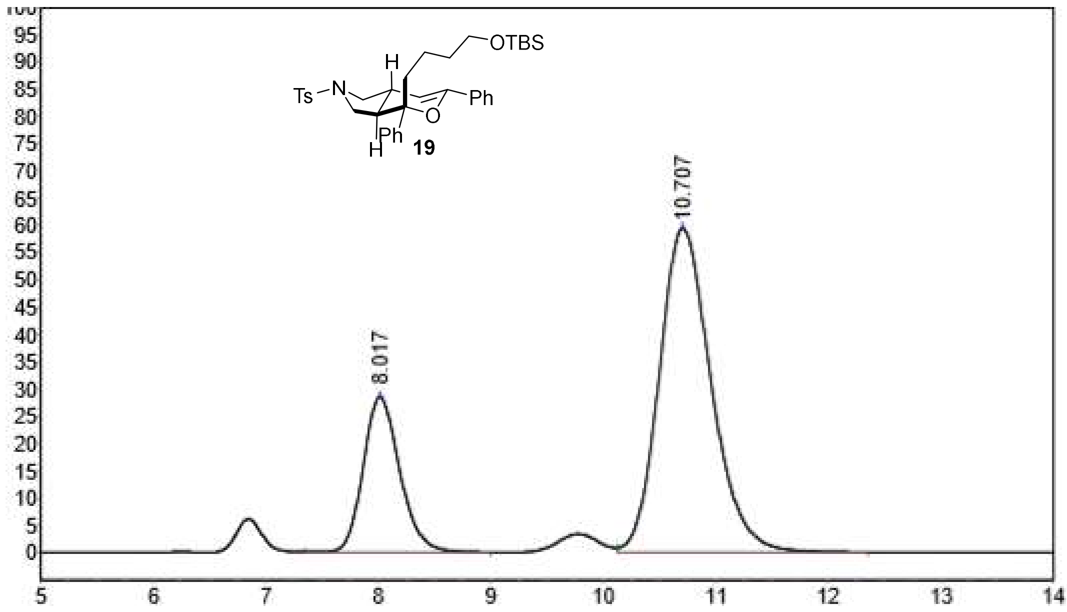

#	RT (time)	Heigh (mV*sec)	Area (mv)	Area (%)
1	8.017	28537.000	648954.250	25.3392
2	10.707	59456.000	1912110.375	74.6608

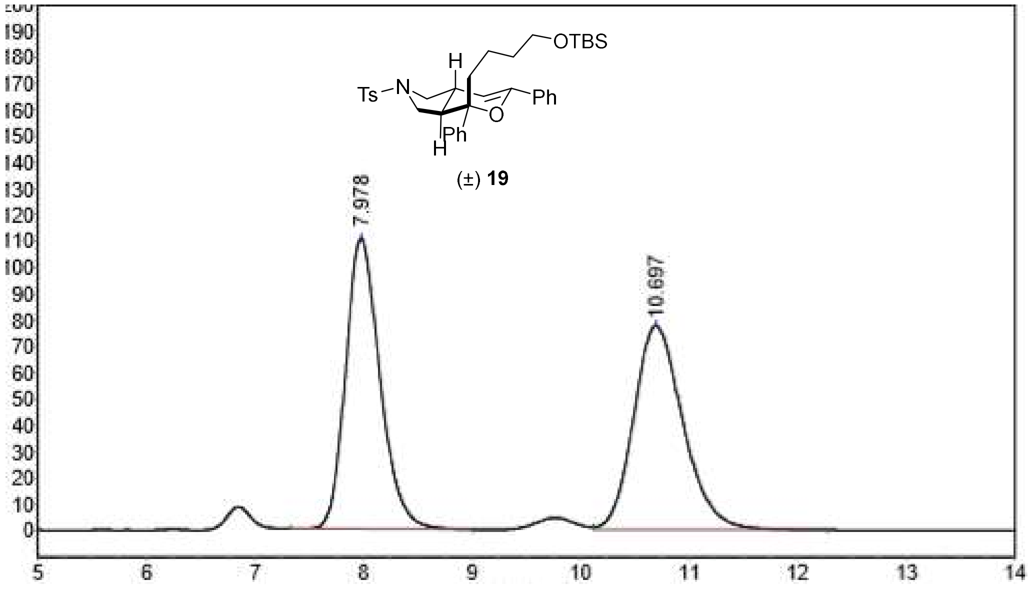

#	RT (time)	Heigh (mV*sec)	Area (mv)	Area (%)
1	7.978	110463.453	2439072.500	49.6616
2	10.697	77819.000	2472317.000	50.3384

Supplementary Figure 60. HPLC Trace of 7u.

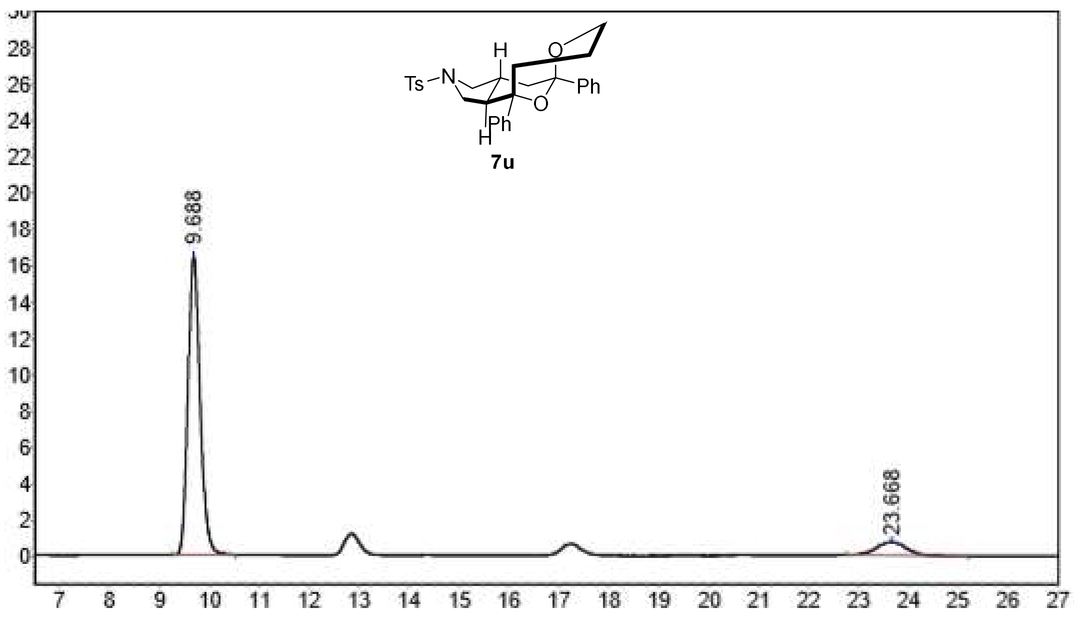

#	RT (time)	Heigh (mV*sec)	Area (mv)	Area (%)
1	9.688	16397.666	276763.656	89.1517
2	23.668	746.590	33677.746	10.8483

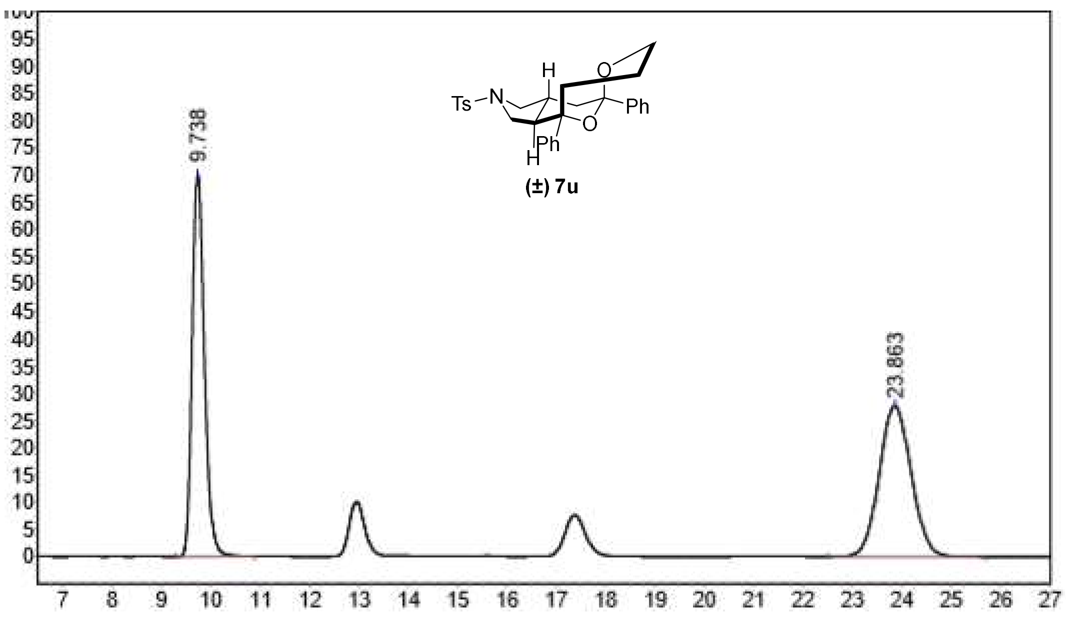

#	RT (time)	Heigh (mV*sec)	Area (mv)	Area (%)
1	9.738	70008.000	1202262.375	49.0294
2	23.863	27734.199	1249861.750	50.9706

Supplementary Figure 61. HPLC Trace of 7v.

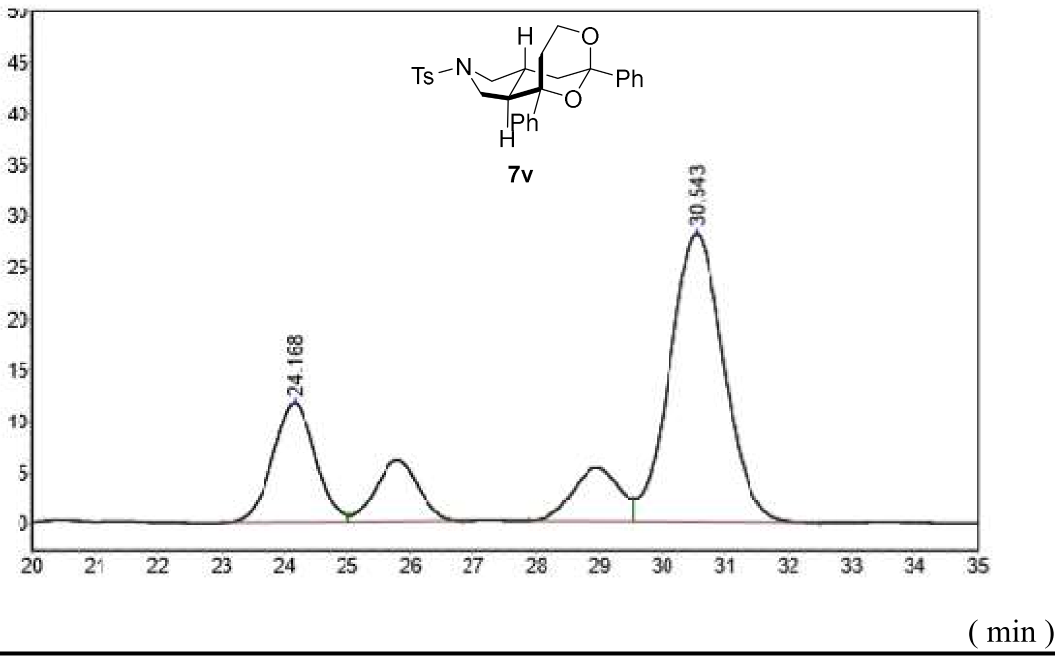

#	RT (time)	Heigh (mV*sec)	Area (mv)	Area (%)
1	24.168	11653.496	549605.438	19.8074
2	30.543	28083.139	1669950.500	60.1839

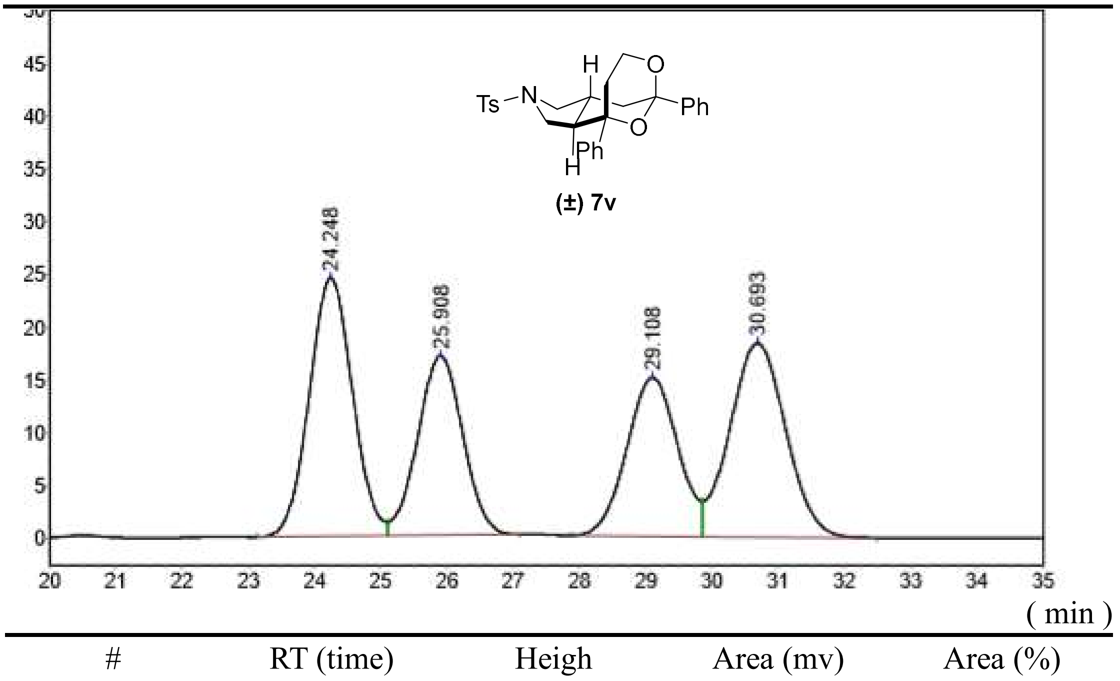

#	RT (time)	Heigh (mV*sec)	Area (mv)	Area (%)
1	24.248	24448.572	1110375.875	29.2448
2	25.908	16986.744	804225.875	21.1815
3	29.108	15009.981	804800.625	21.1966
4	30.693	18364.178	1077431.750	28.3771

Supplementary Figure 62. HPLC Trace of 7w.

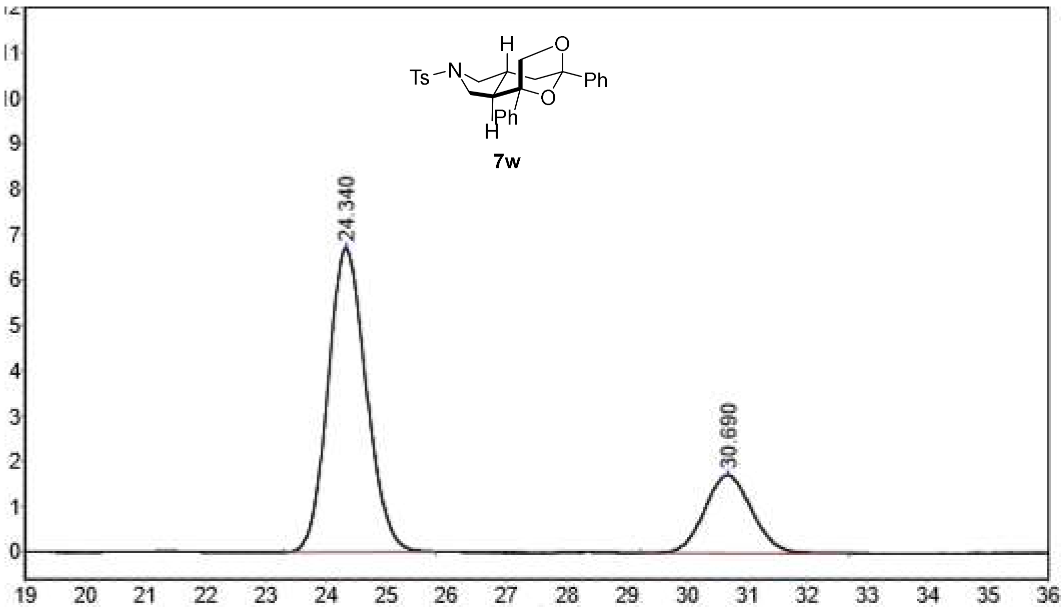

(min)

#	RT (time)	Heigh (mV*sec)	Area (mv)	Area (%)
1	24.340	6693.421	295977.500	75.7863
2	30.690	1717.115	94564.797	24.2137

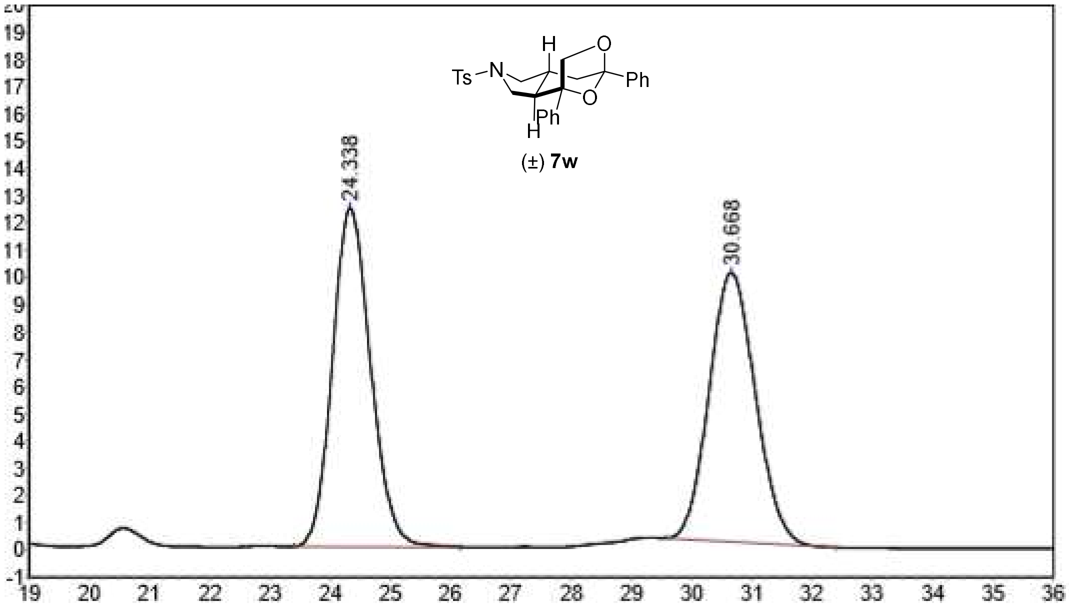

(min)

#	RT (time)	Heigh (mV*sec)	Area (mv)	Area (%)
1	24.338	12427.058	548980.000	50.9566
2	30.668	9870.736	528368.000	49.0434

Supplementary Figure 63. HPLC Trace of 7u'.

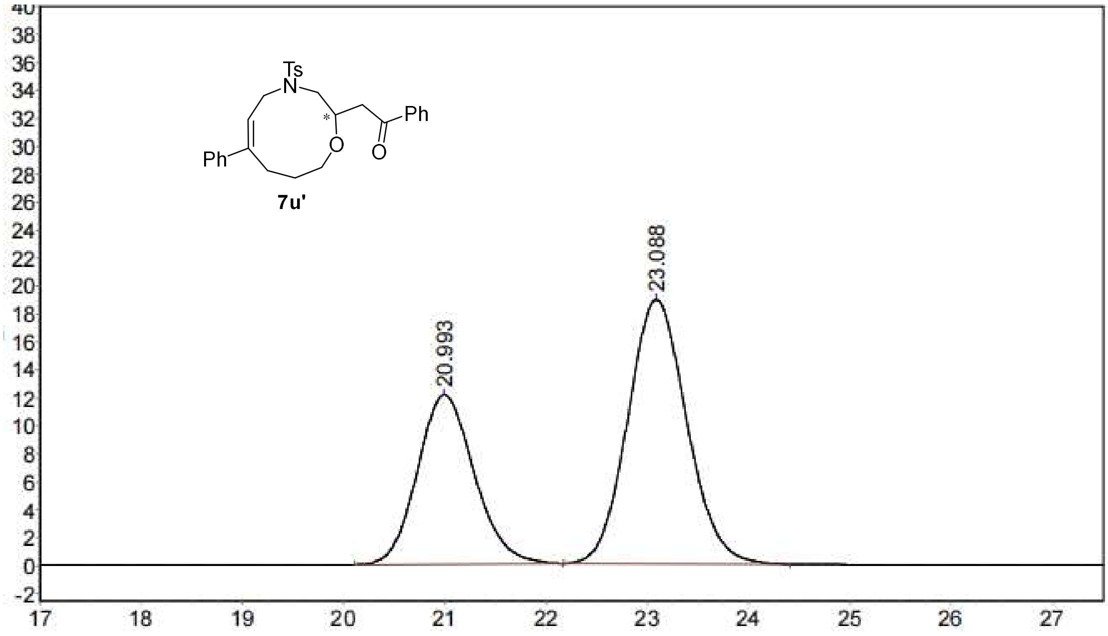

(min)

#	RT (time)	Heigh (mV*sec)	Area (mv)	Area (%)
1	20.993	12094.643	471216.344	38.2453
2	23.088	18852.799	760874.313	61.7547

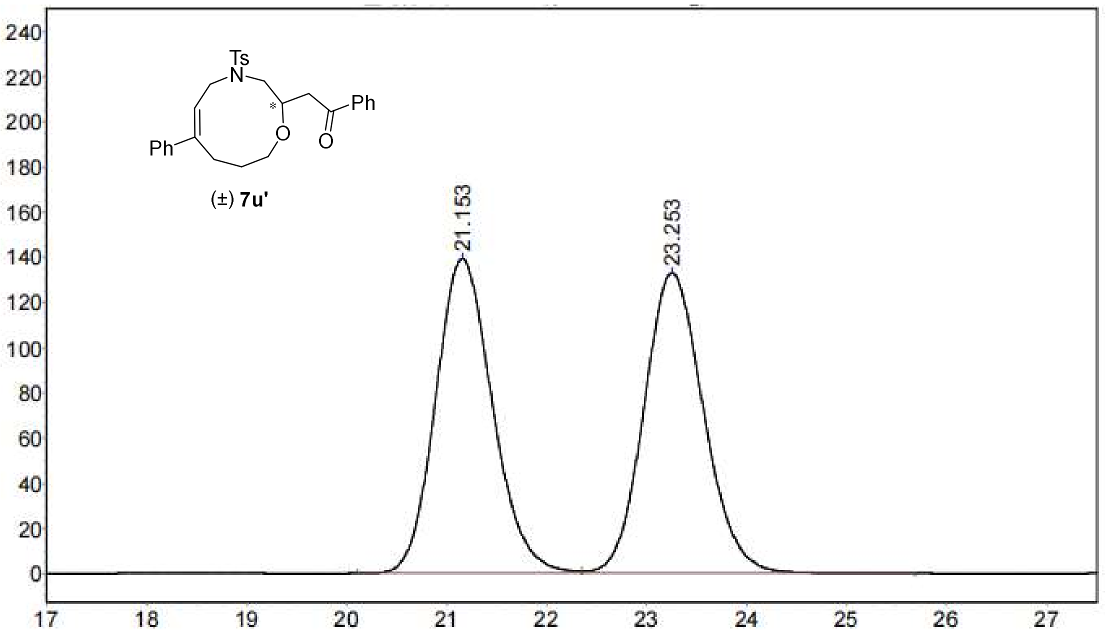

(min)

#	RT (time)	Heigh (mV*sec)	Area (mv)	Area (%)
1	21.153	139214.063	5503952.000	49.9411
2	23.253	132867.219	5516928.500	50.0589

Supplementary Figure 64. HPLC Trace of 7v'.

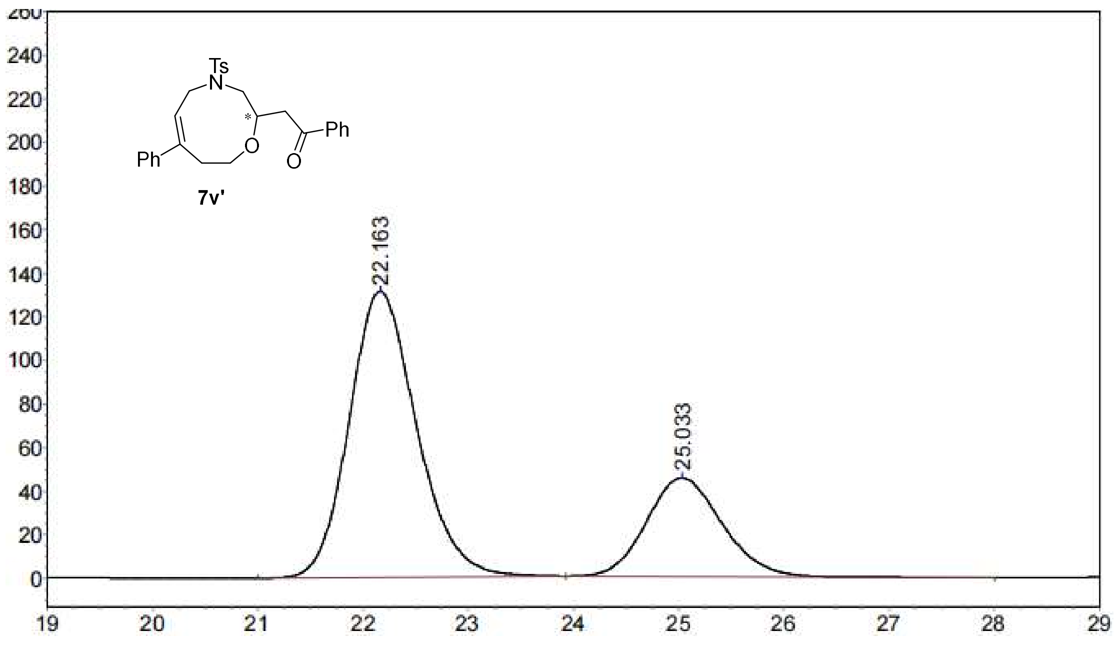

#	RT (time)	Heigh (mV*sec)	Area (mv)	Area (%)
1	22.163	131237.188	5845473.000	72.0286
2	25.033	45211.727	2270010.000	27.9713

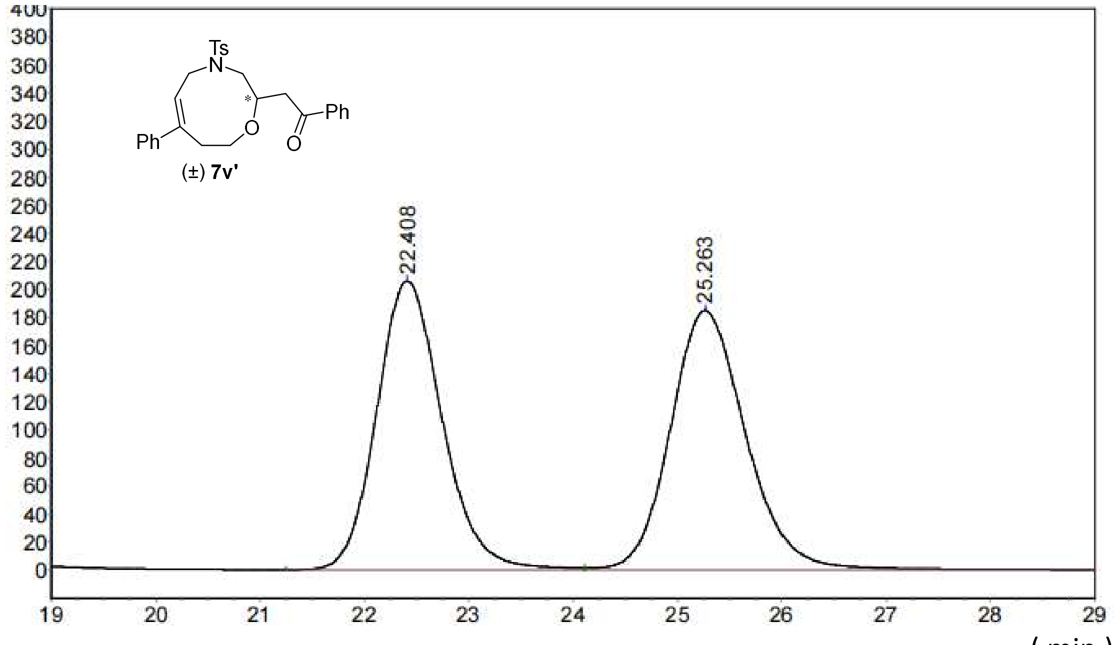

#	RT (time)	Heigh (mV*sec)	Area (mv)	Area (%)
1	22.408	205948.906	8978625.000	49.1441
2	25.263	184554.422	9291363.000	50.8559

Supplementary Figure 65. HPLC Trace of 7w'.

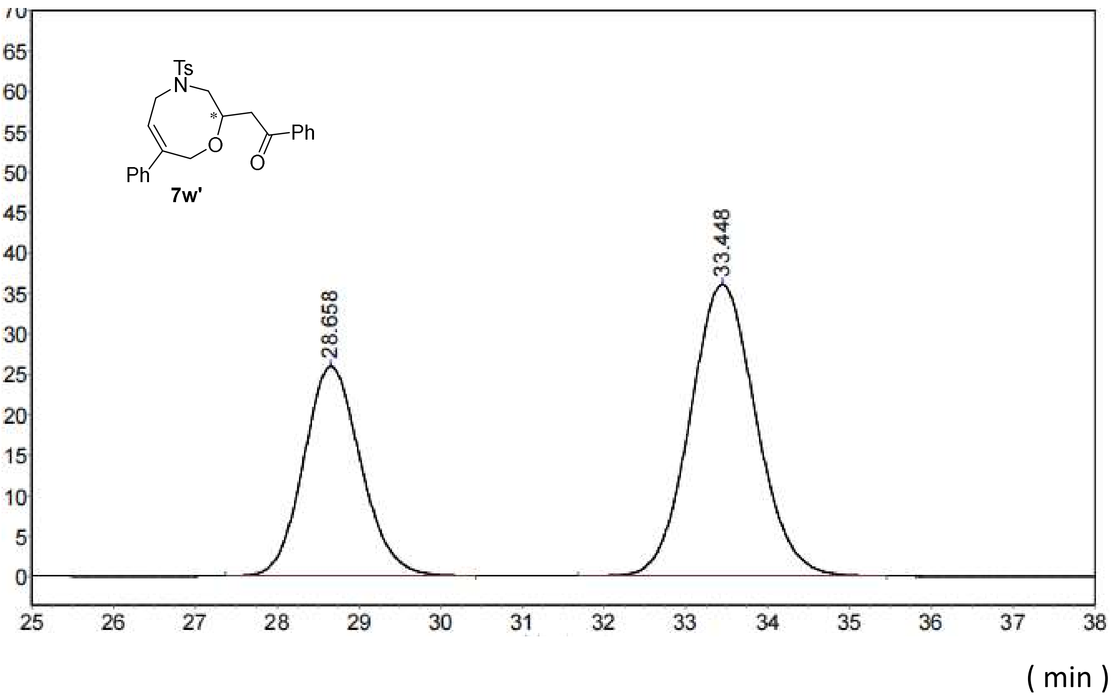

#	RT (time)	Heigh (mV*sec)	Area (mv)	Area (%)
1	28.658	25968.953	1264101.375	38.4576
2	33.448	36095.984	2022895.500	61.5424

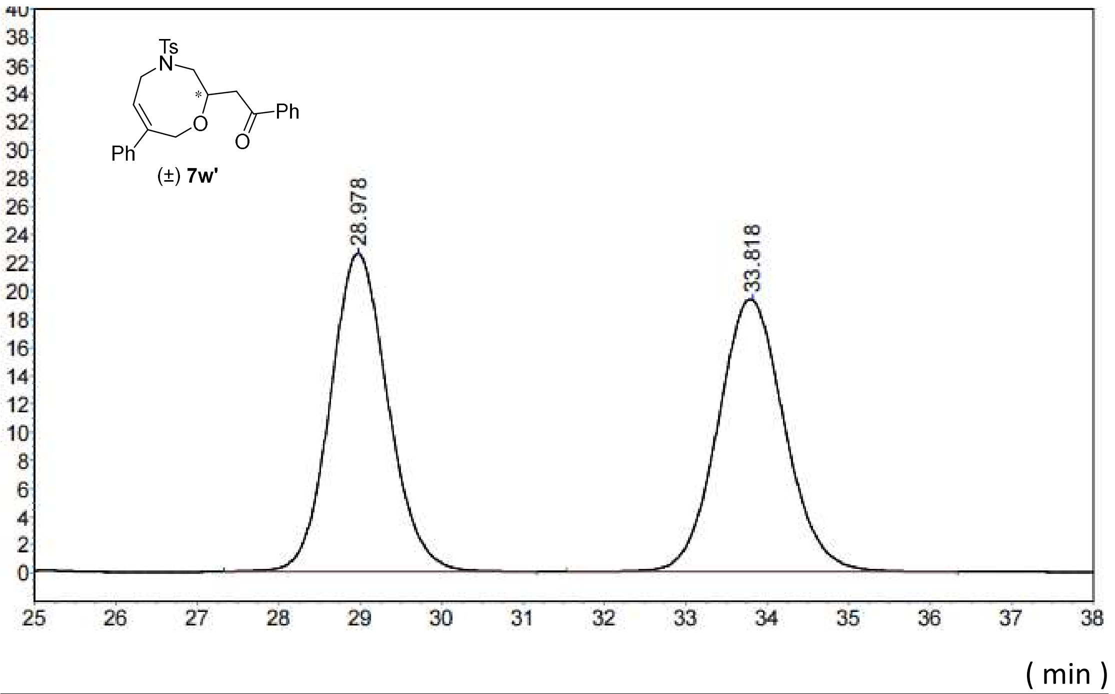

#	RT (time)	Heigh (mV*sec)	Area (mv)	Area (%)
1	28.978	22560.080	1115406.750	50.2522
2	33.818	19339.740	1104211.375	49.7478

Supplementary Figure 66. HPLC Trace of 7w”.

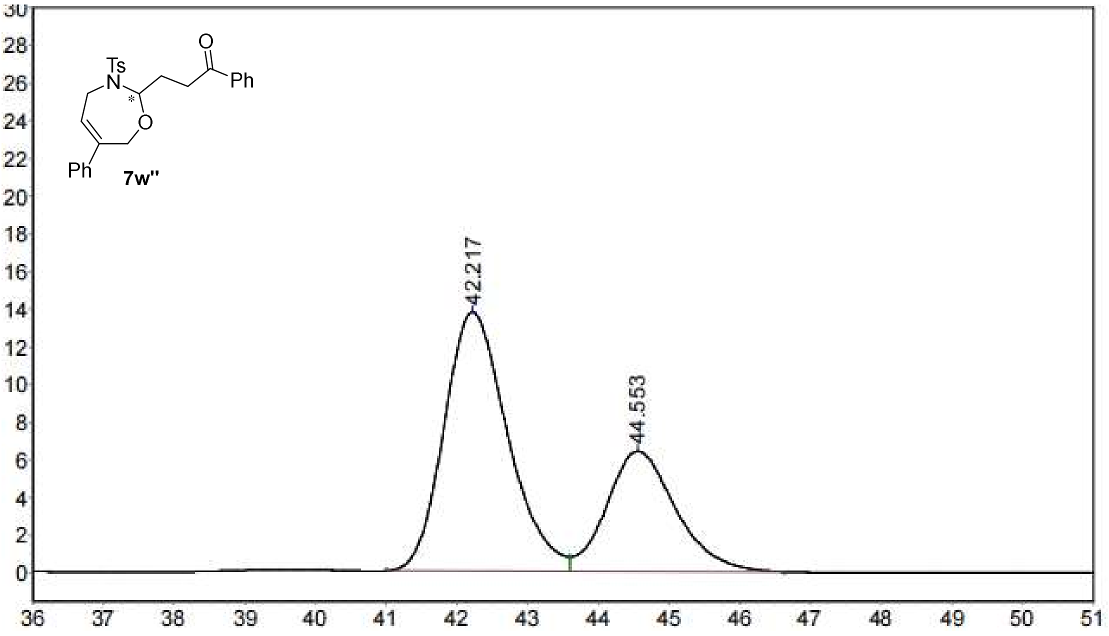

#	RT (time)	Heigh (mV*sec)	Area (mv)	Area (%)
1	42.217	13752.183	858801.313	66.3774
2	44.553	6387.825	435014.656	33.6226

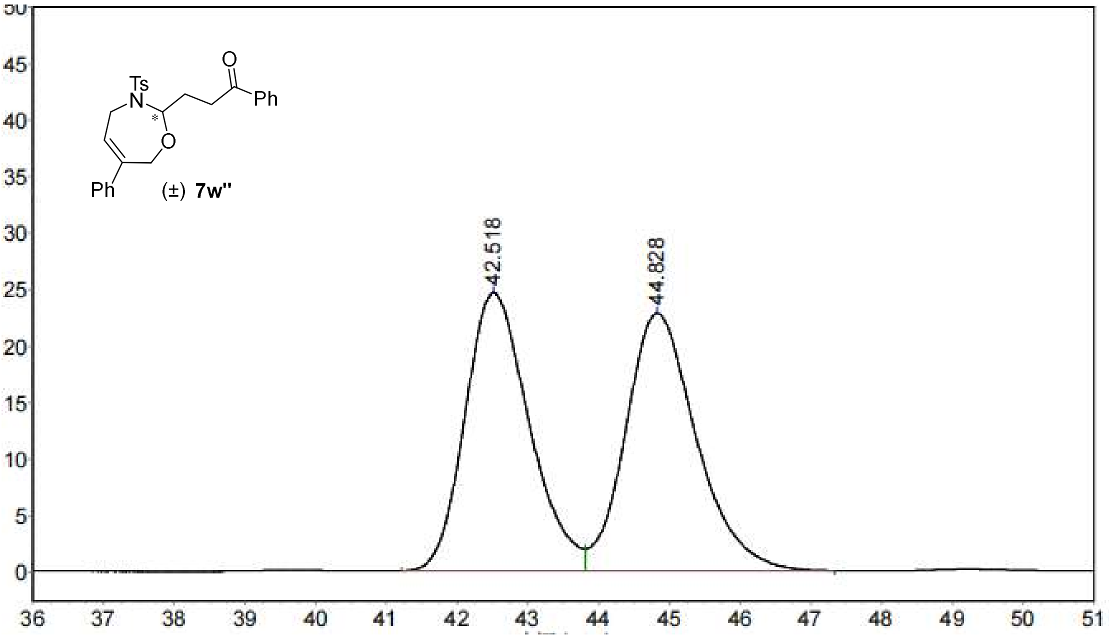

#	RT (time)	Heigh (mV*sec)	Area (mv)	Area (%)
1	42.518	24602.016	1549907.000	49.4813
2	44.828	22814.730	1582400.000	50.5187

^{1}H NMR、^{13}C NMR and ^{19}F NMR spectra

Supplementary Figure 67. ^{1}H NMR spectrum of 5a.

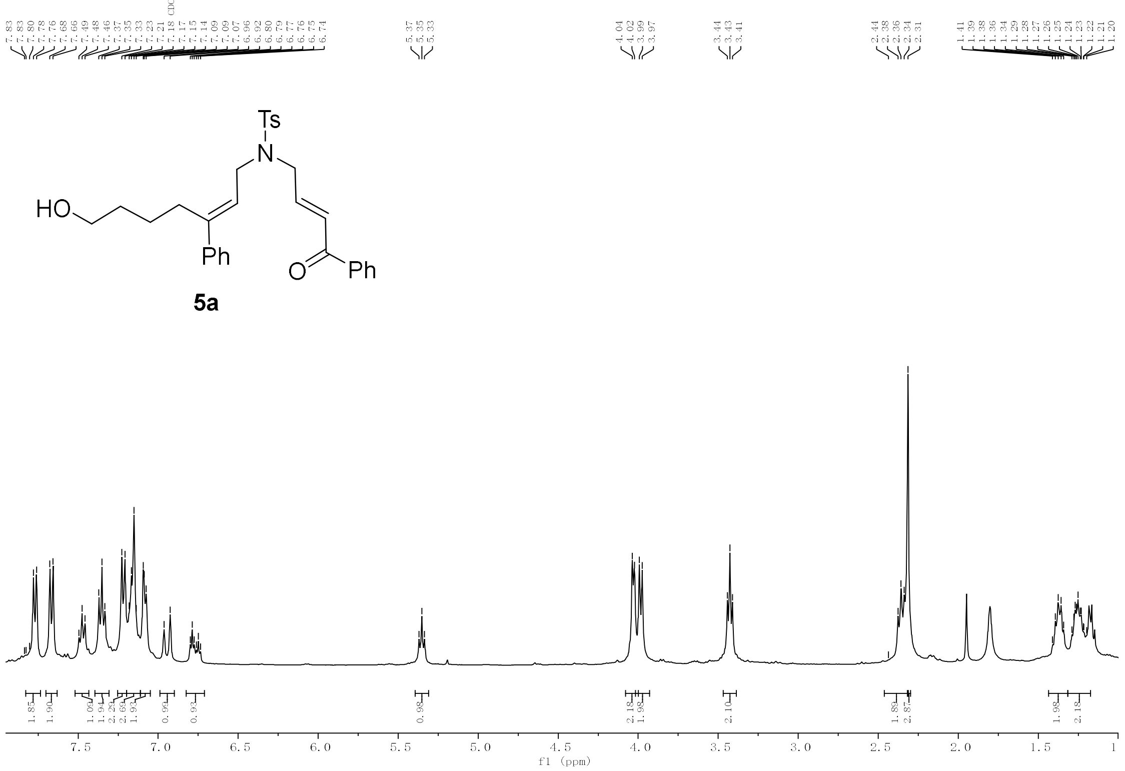

Supplementary Figure 68. ^{13}C NMR spectrum of 5a.

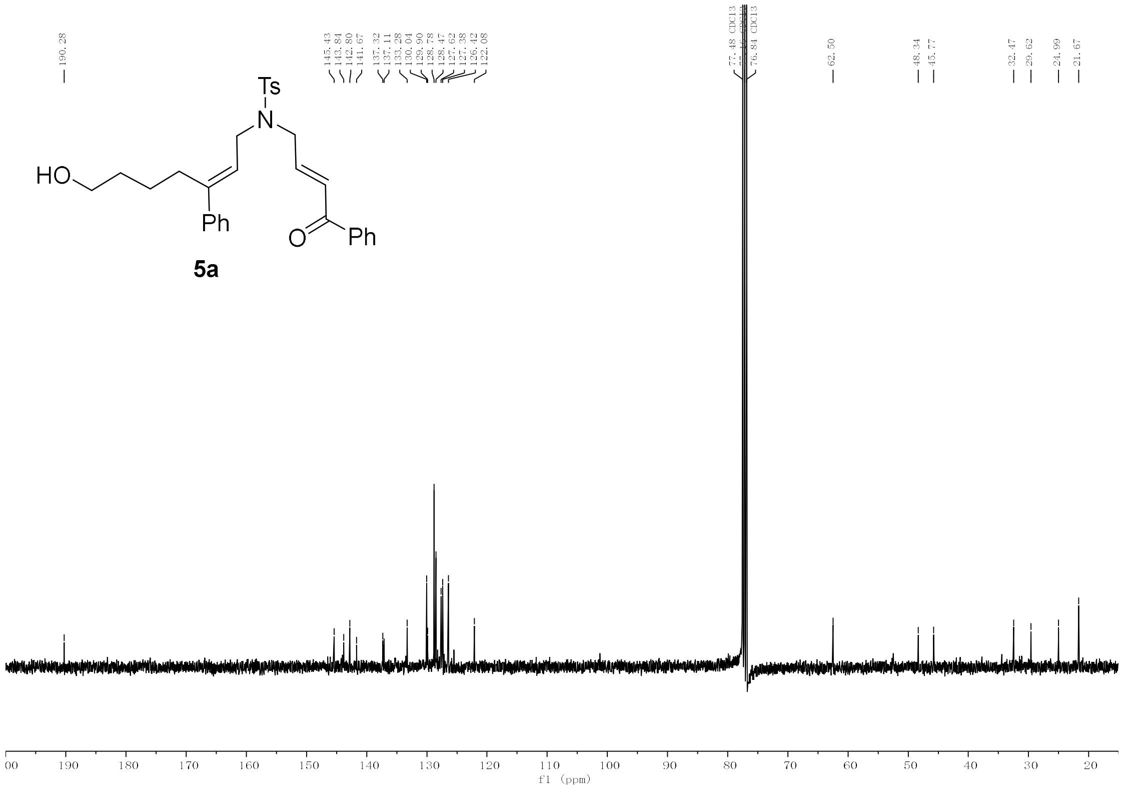

Supplementary Figure 69. ^{1}H NMR spectrum of 5b.

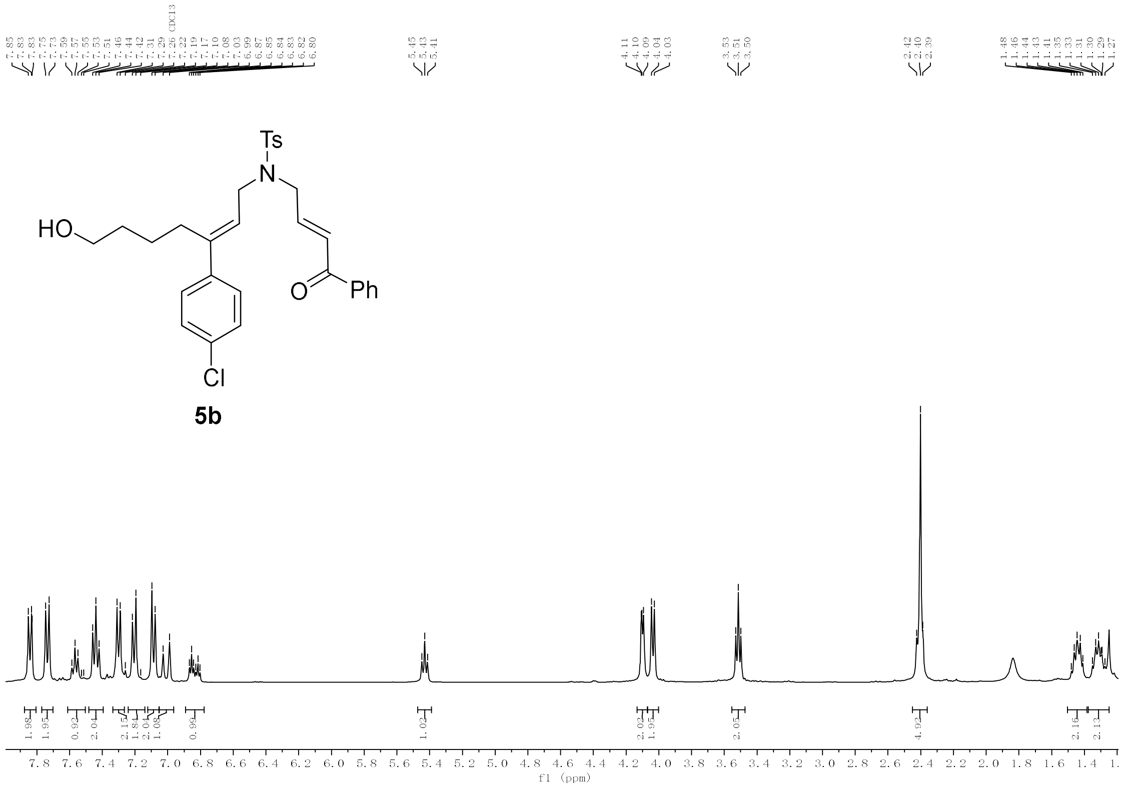

Supplementary Figure 70. ^{13}C NMR spectrum of 5b.

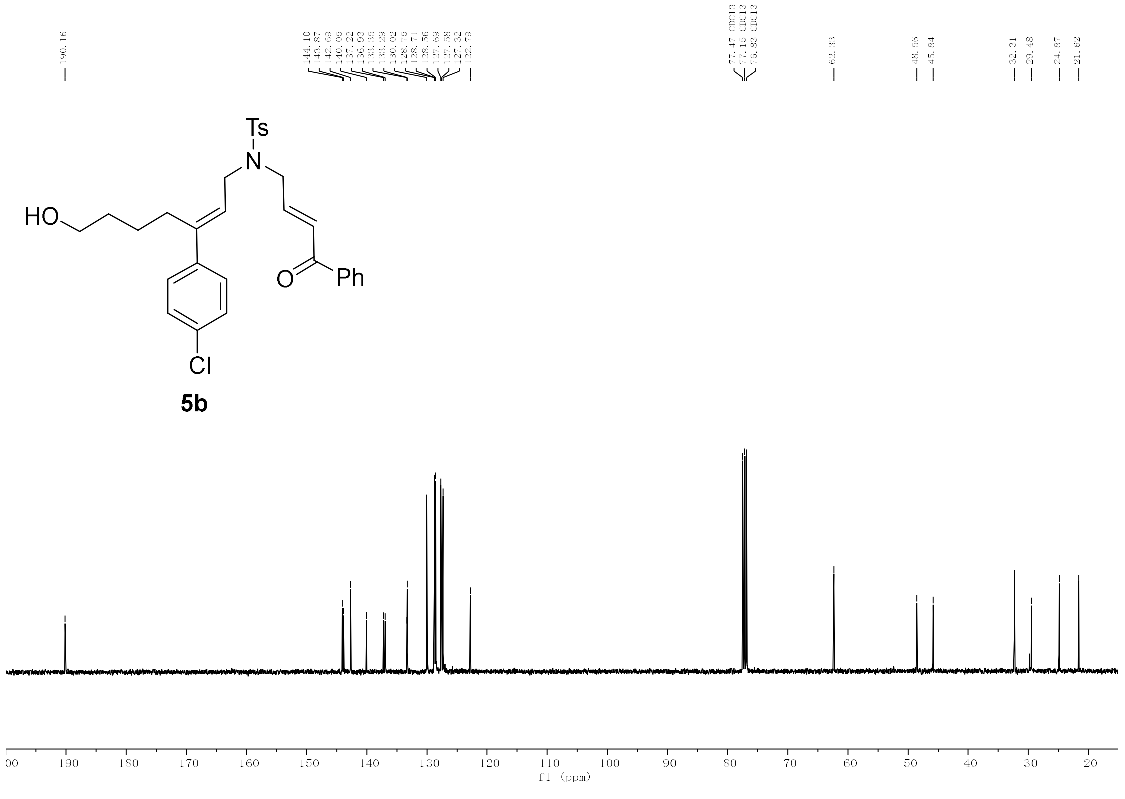

Supplementary Figure 71. ^{1}H NMR spectrum of 5c.

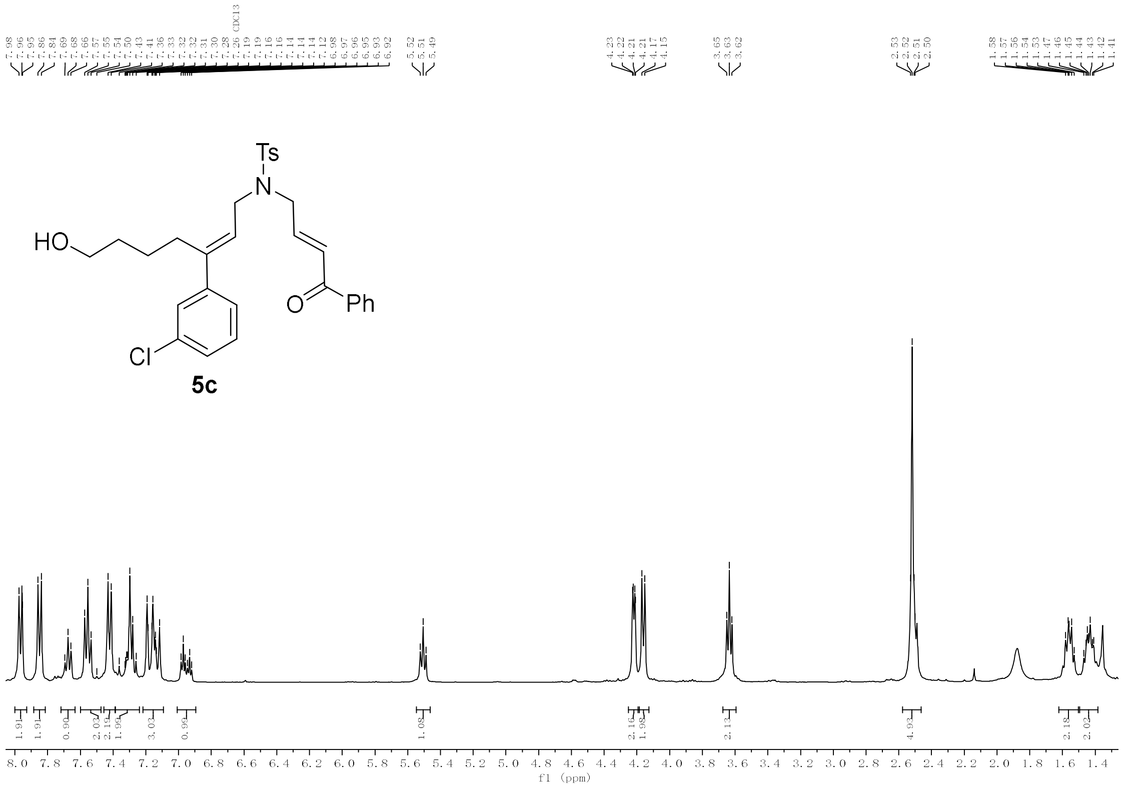

Supplementary Figure 72. ^{13}C NMR spectrum of 5c.

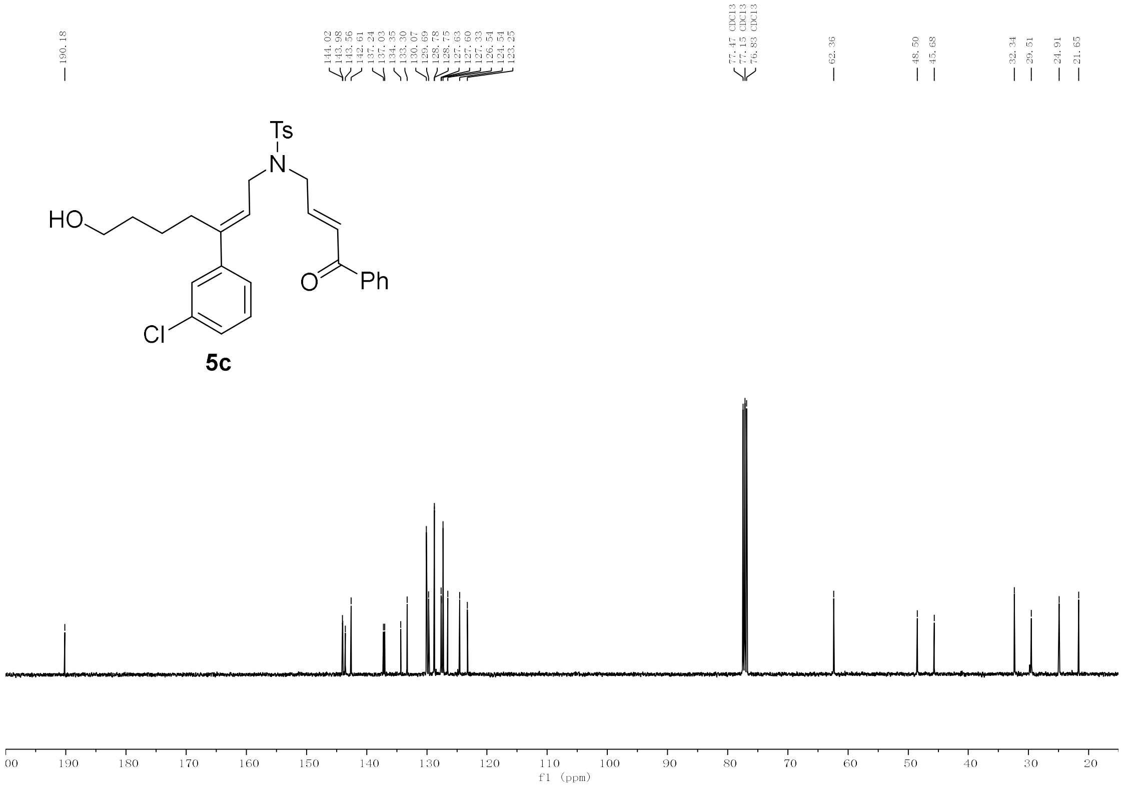

Supplementary Figure 73. ^{1}H NMR spectrum of 5d.

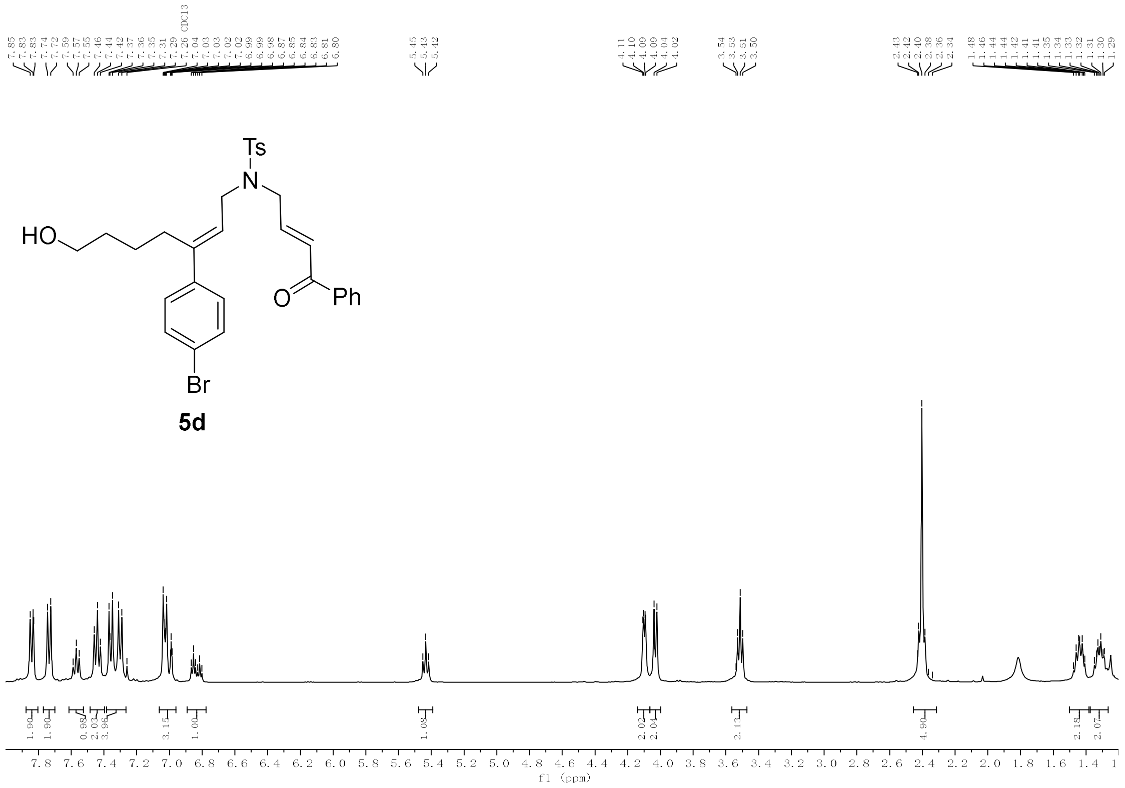

Supplementary Figure 74. ^{13}C NMR spectrum of 5d.

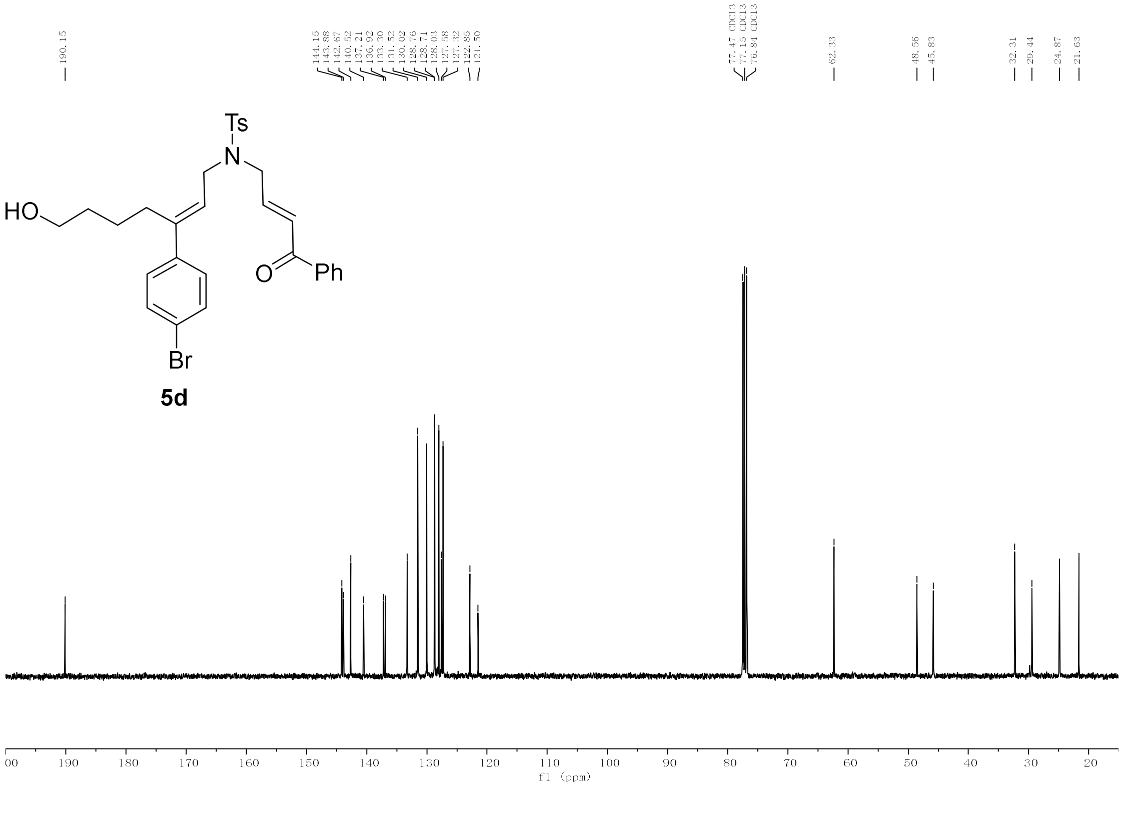

Supplementary Figure 75. ^{1}H NMR spectrum of 5e.

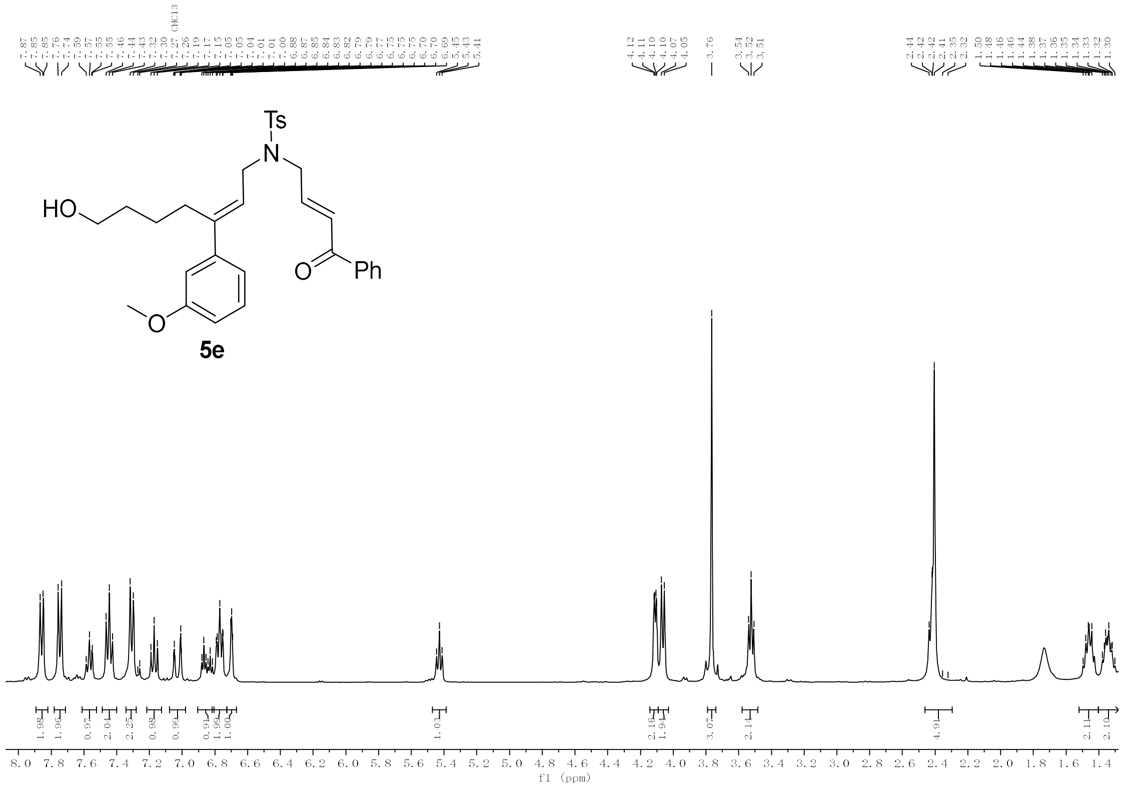

Supplementary Figure 76. ^{13}C NMR spectrum of 5e.

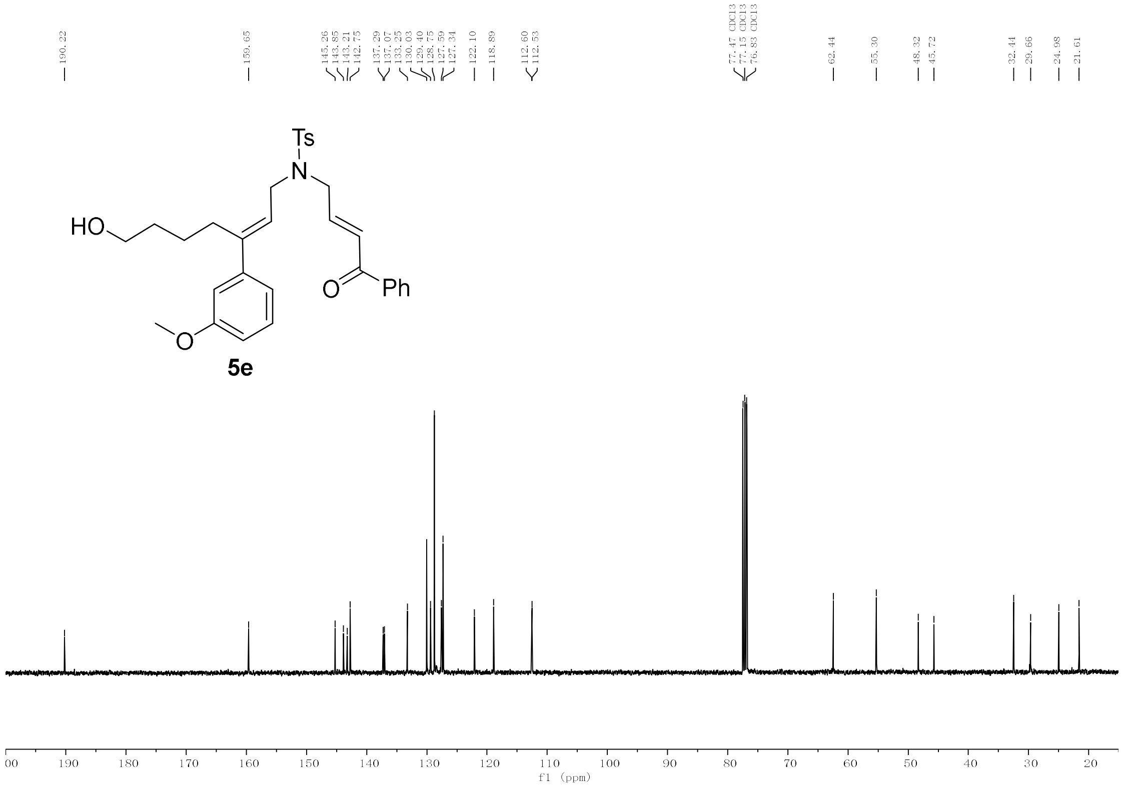

Supplementary Figure 77. ^{1}H NMR spectrum of 5f.

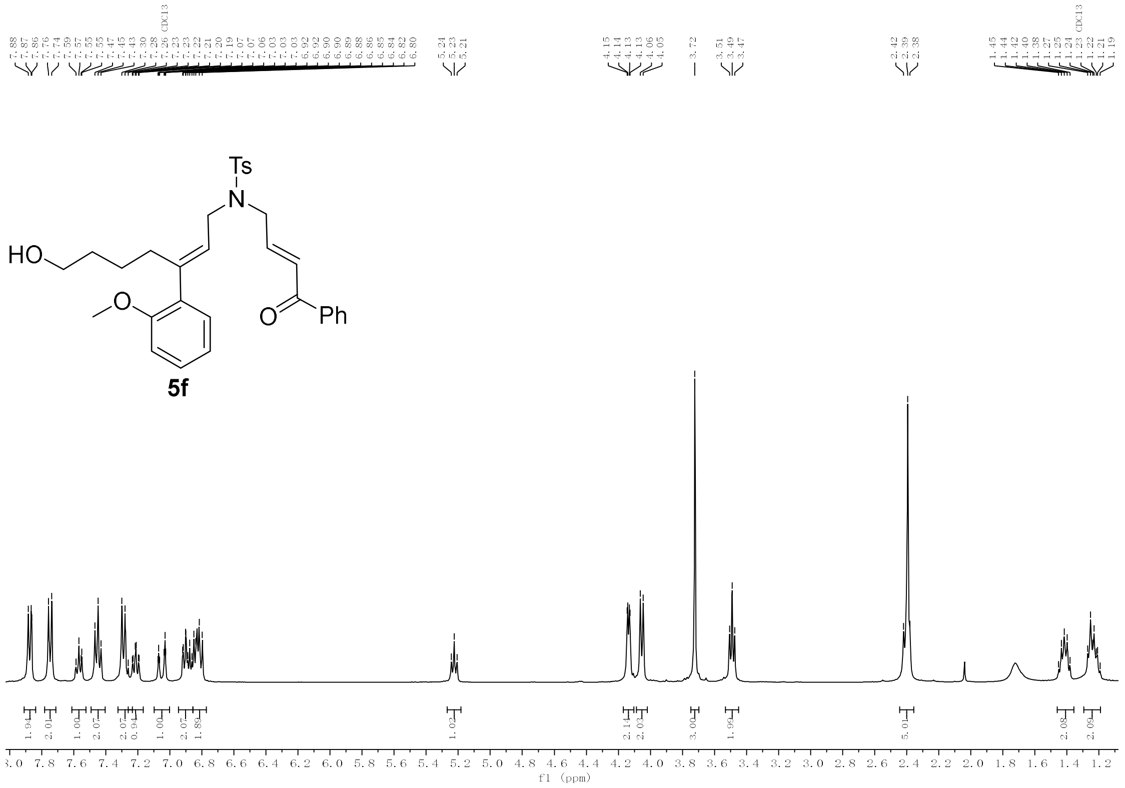

Supplementary Figure 78. ^{13}C NMR spectrum of 5f.

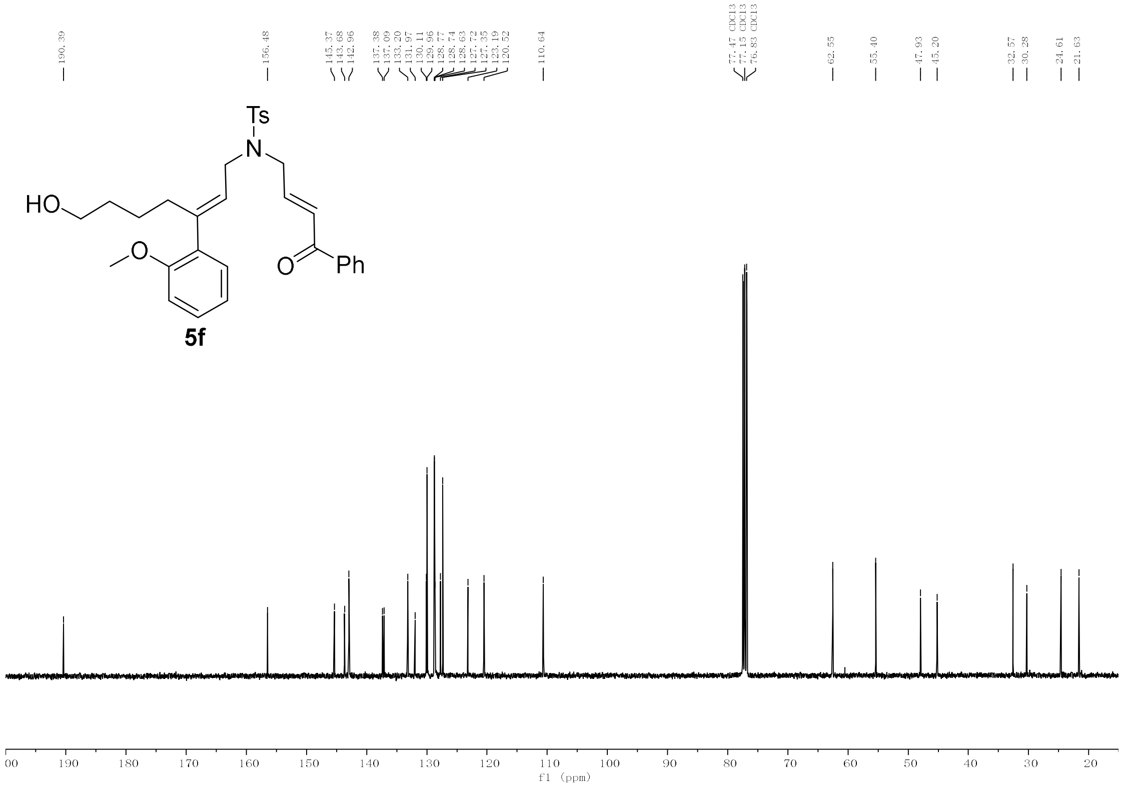

Supplementary Figure 79. ^{1}H NMR spectrum of 5g.

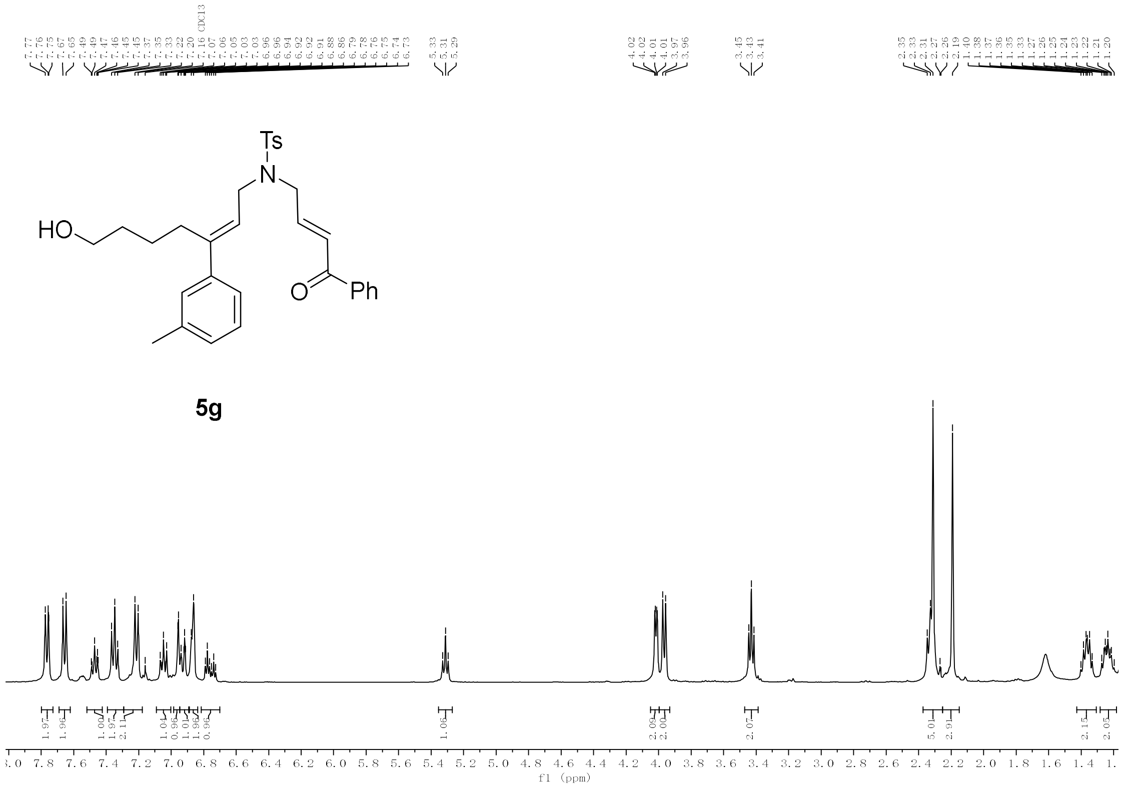

Supplementary Figure 80. ^{13}C NMR spectrum of 5g.

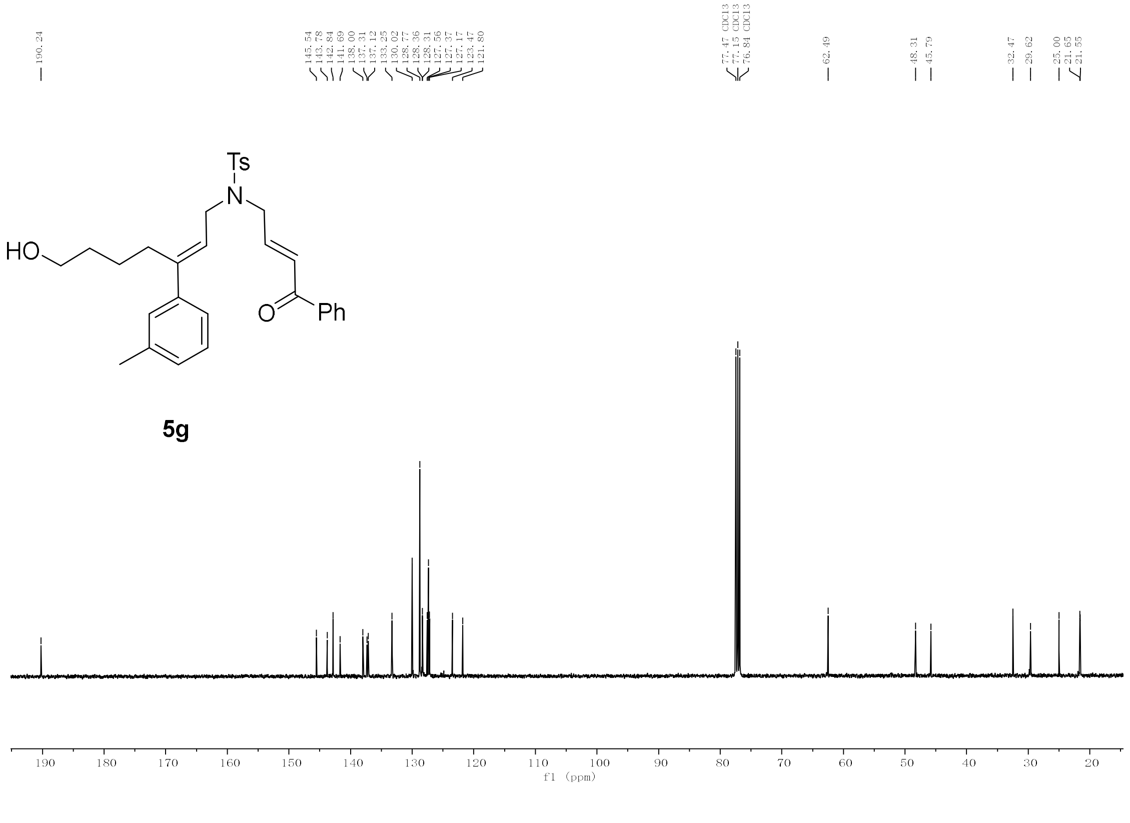

Supplementary Figure 81. ^{1}H NMR spectrum of 5h.

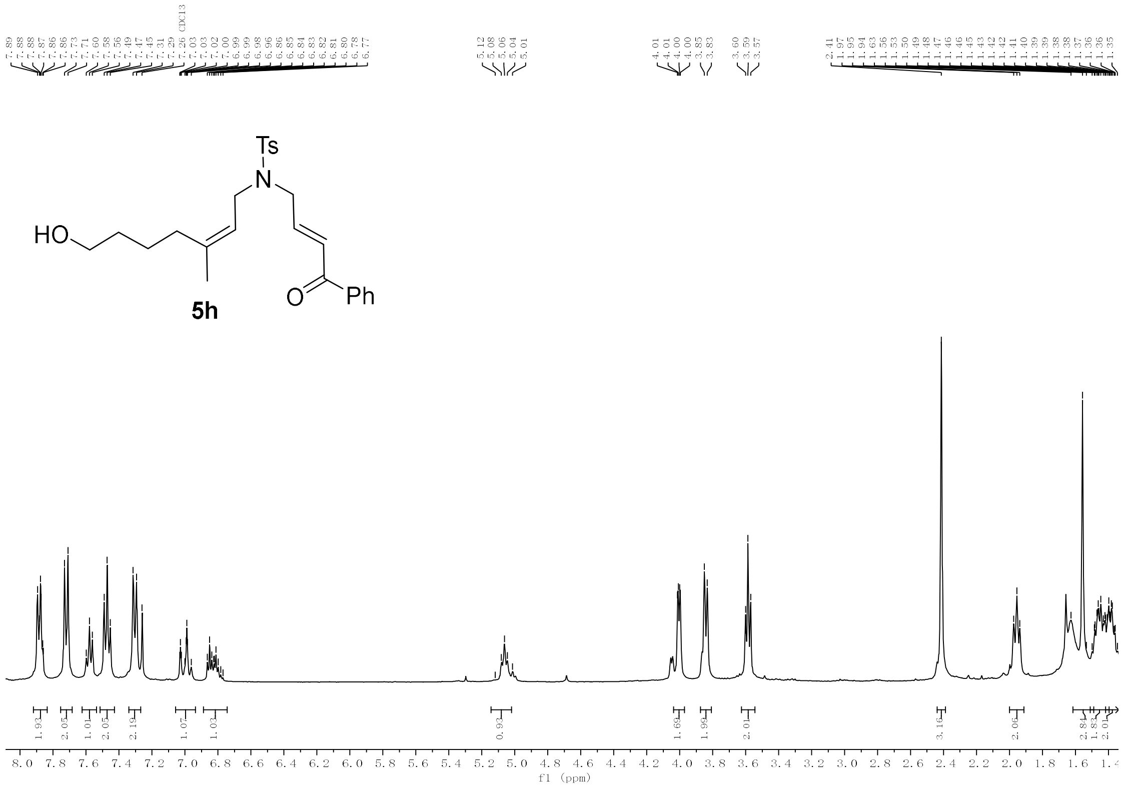

Supplementary Figure 82. ^{13}C NMR spectrum of 5h.

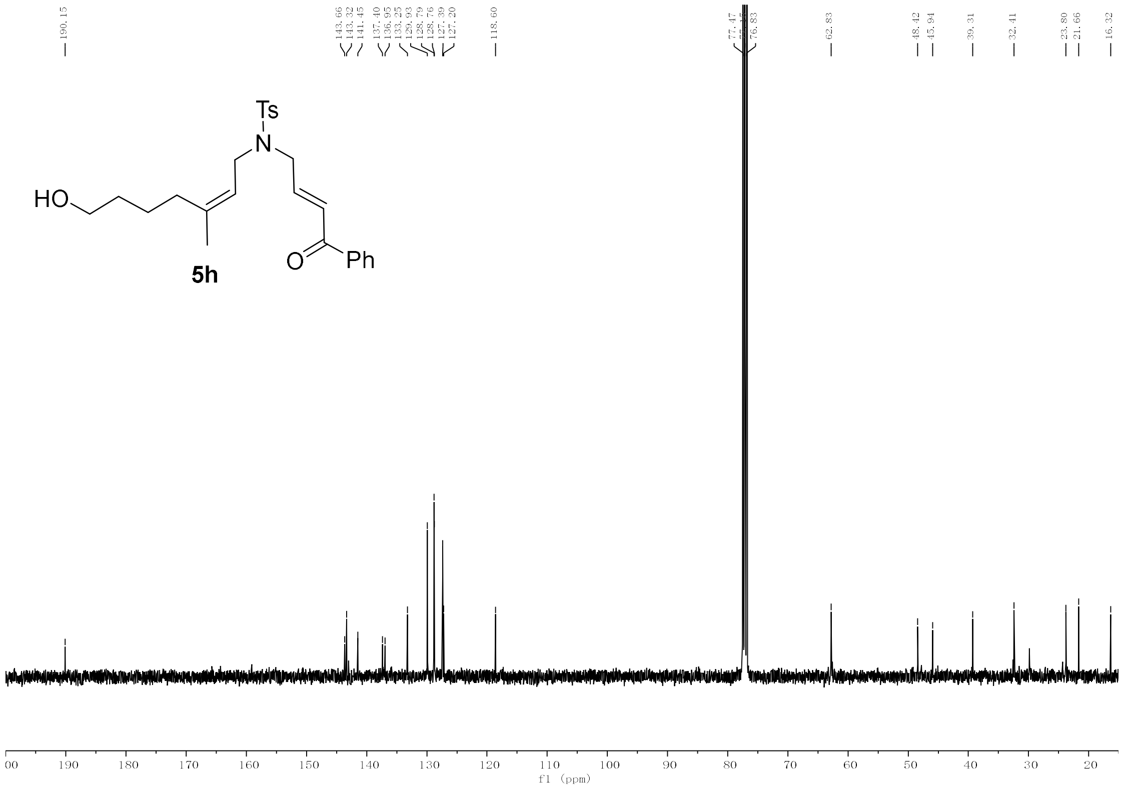

Supplementary Figure 83. ^{1}H NMR spectrum of 5i.

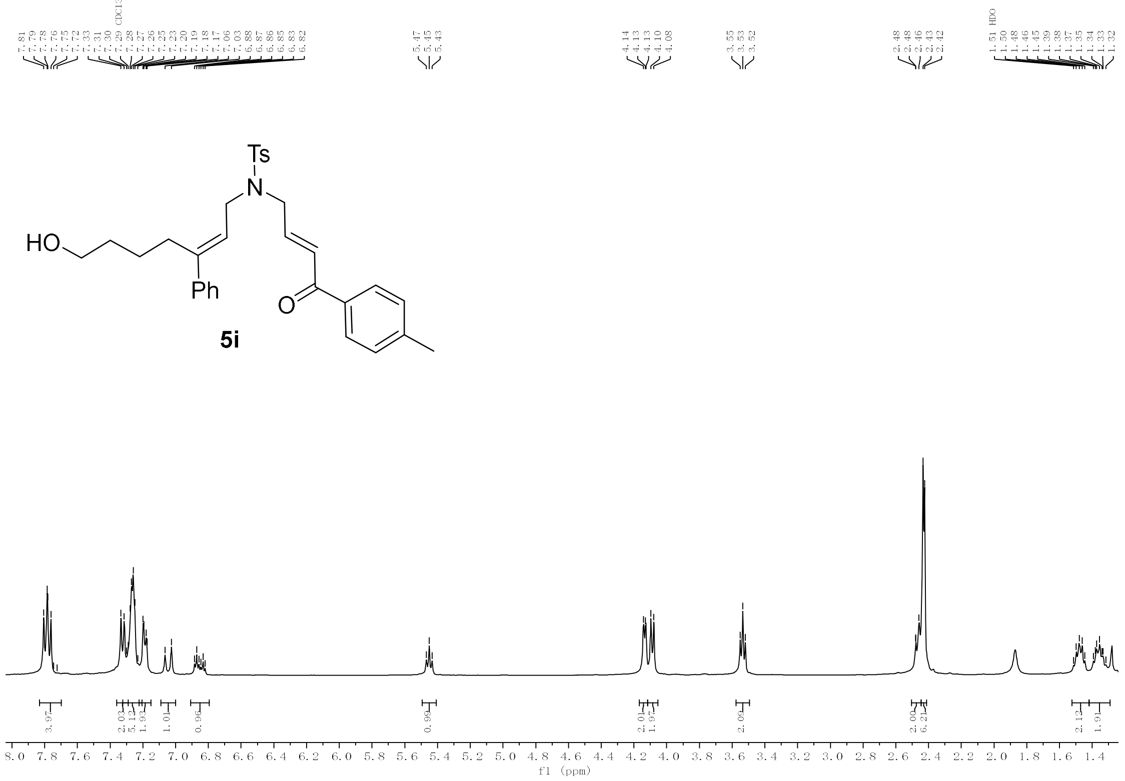

Supplementary Figure 84. ^{13}C NMR spectrum of 5i.

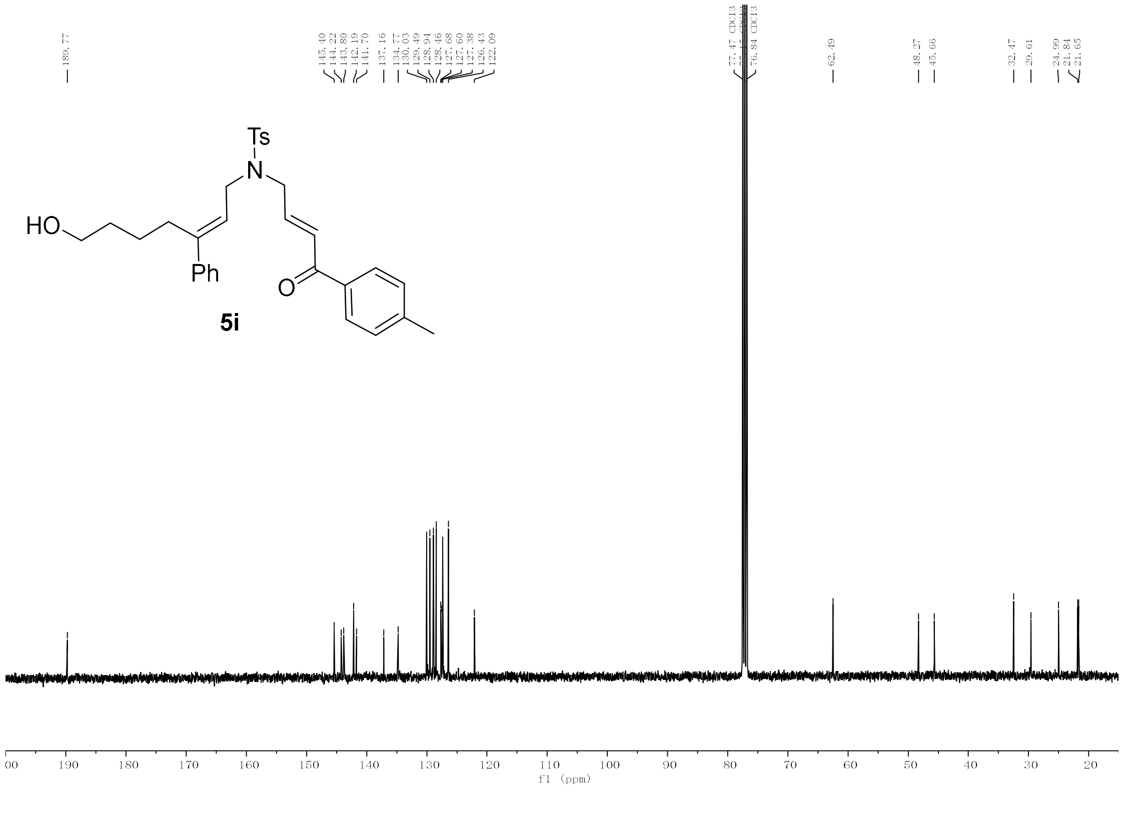

Supplementary Figure 85. ^{1}H NMR spectrum of 5j.

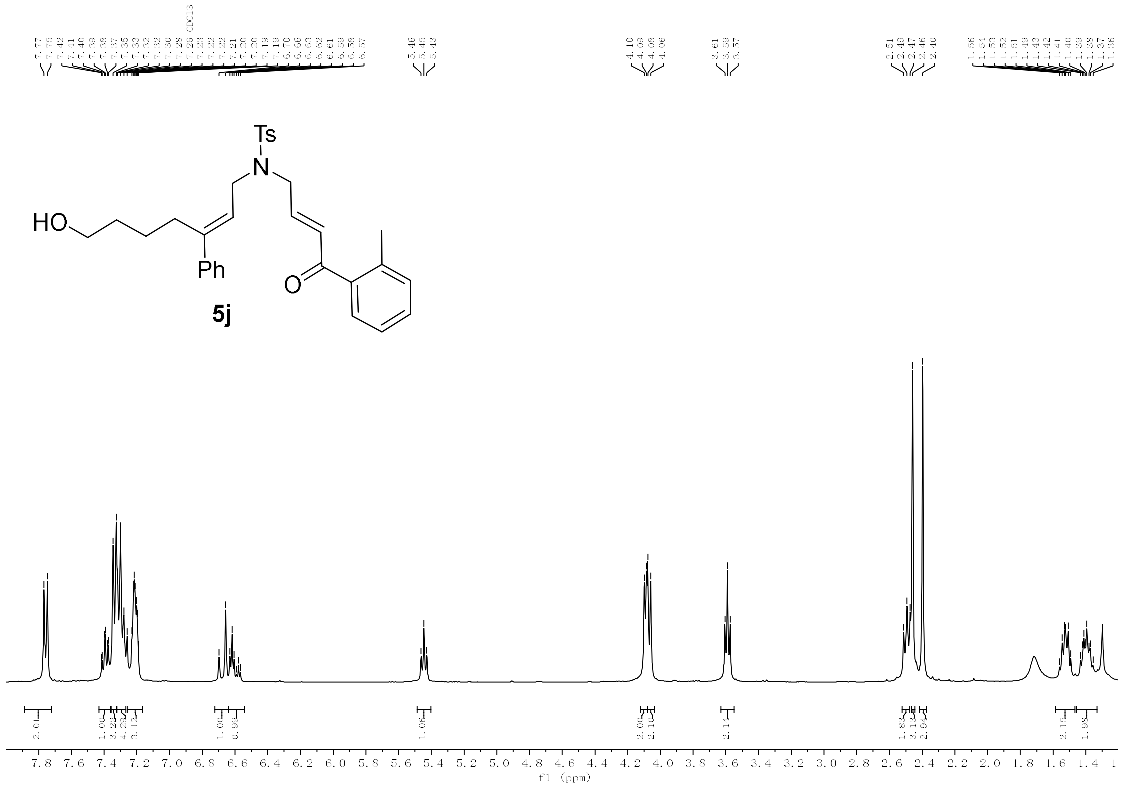

Supplementary Figure 86. ^{13}C NMR spectrum of 5j.

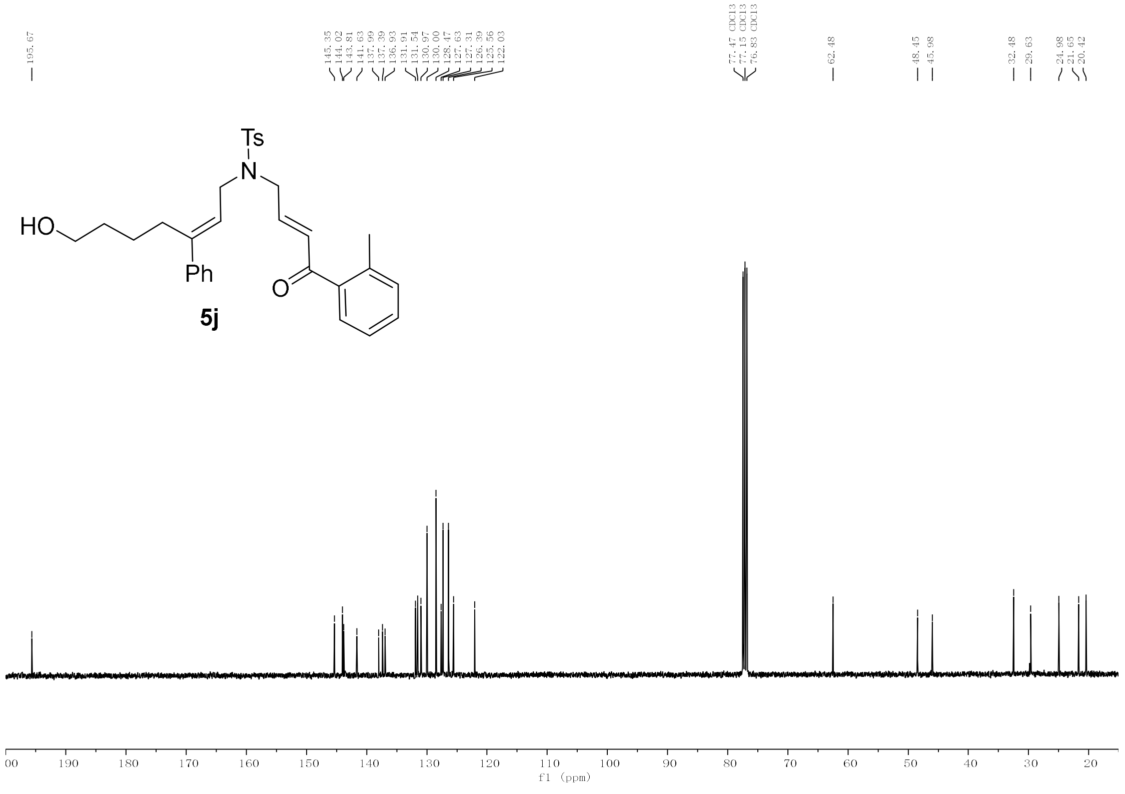

Supplementary Figure 87. ^{1}H NMR spectrum of 5k.

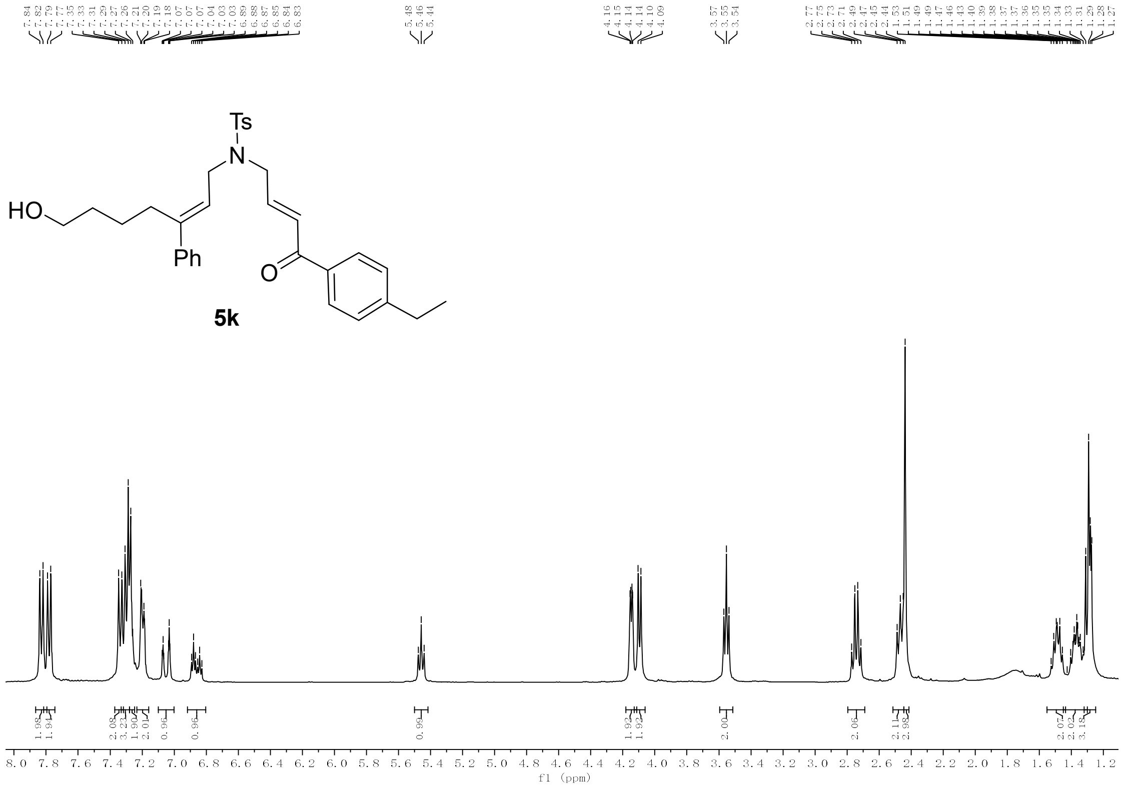

Supplementary Figure 88. ^{13}C NMR spectrum of 5k.

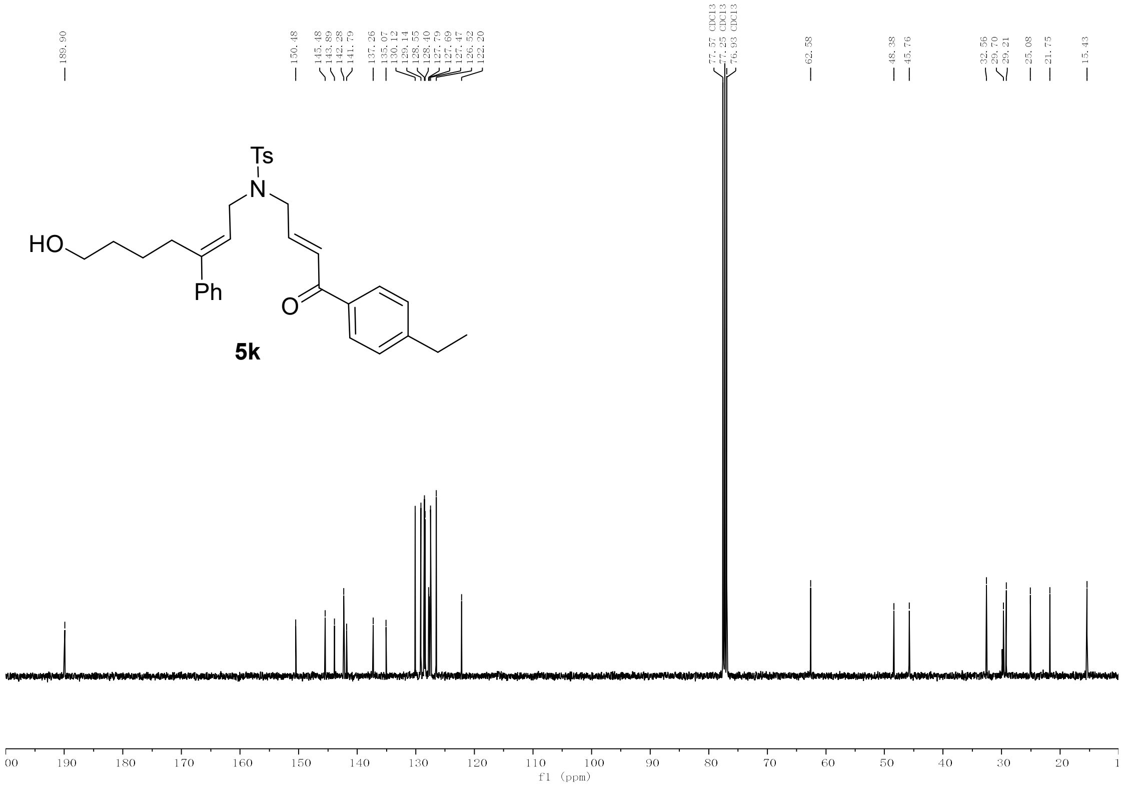

Supplementary Figure 89. ^{1}H NMR spectrum of 5l.

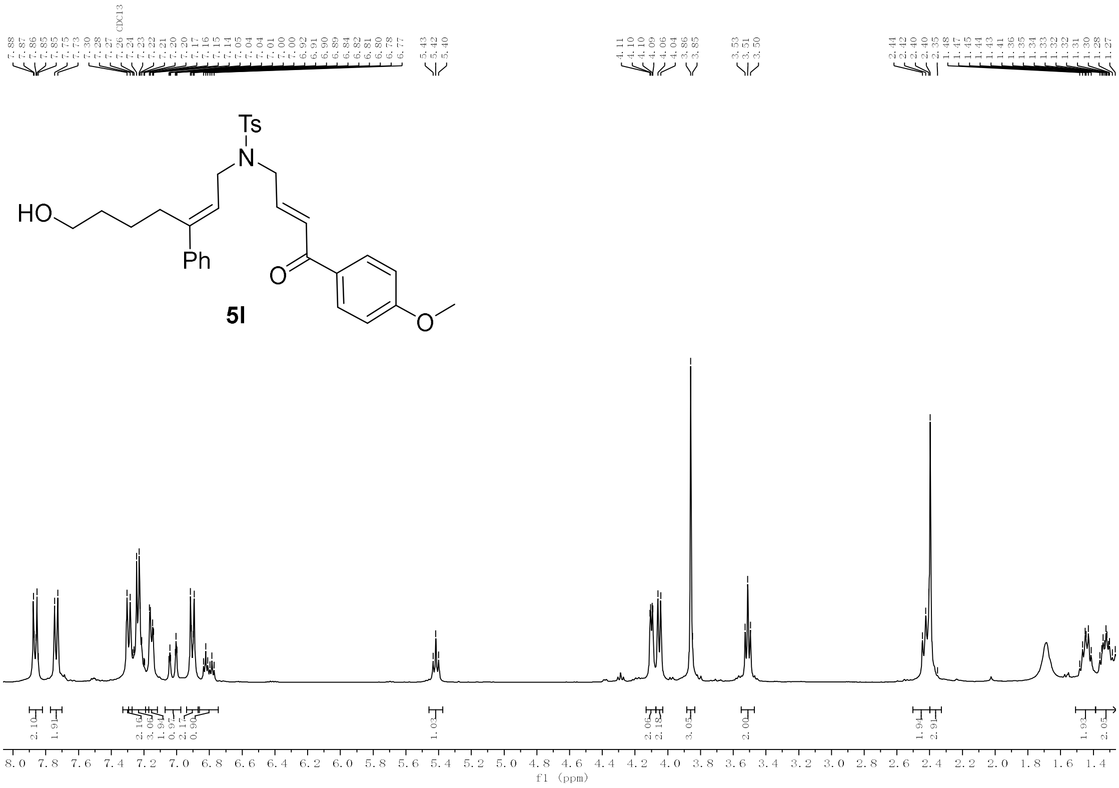

Supplementary Figure 90. ^{13}C NMR spectrum of 5l.

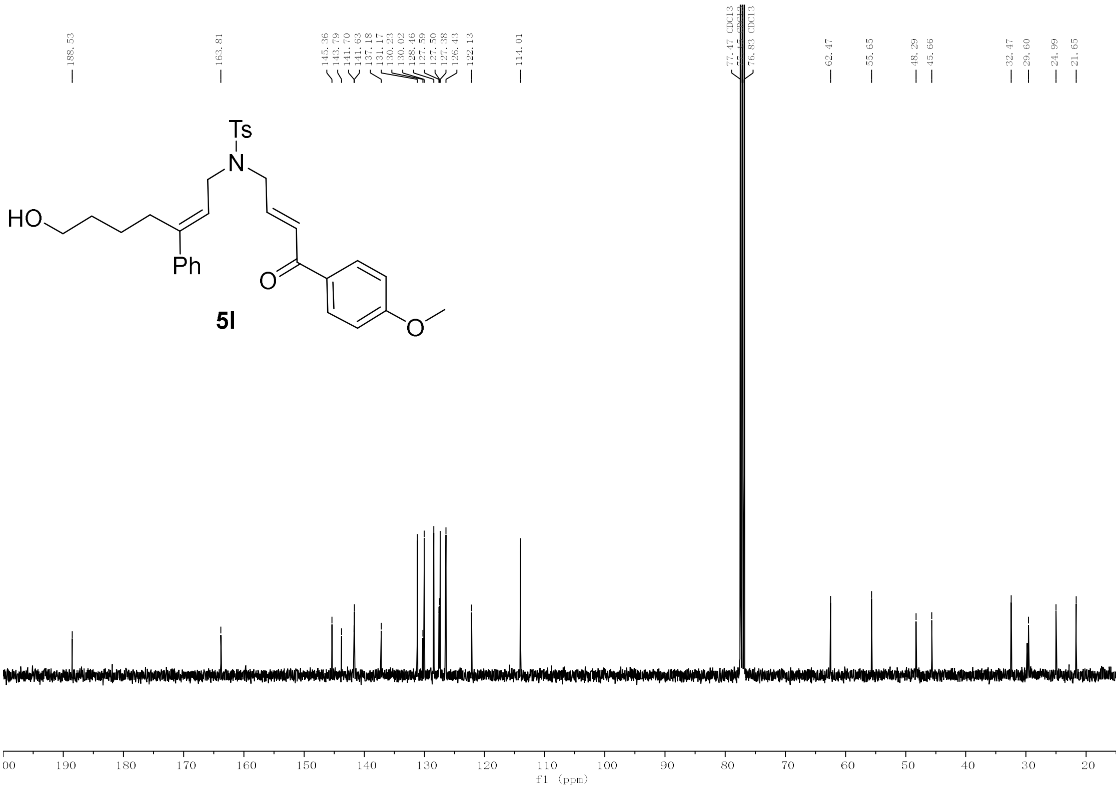

Supplementary Figure 91. ^{1}H NMR spectrum of 5m.

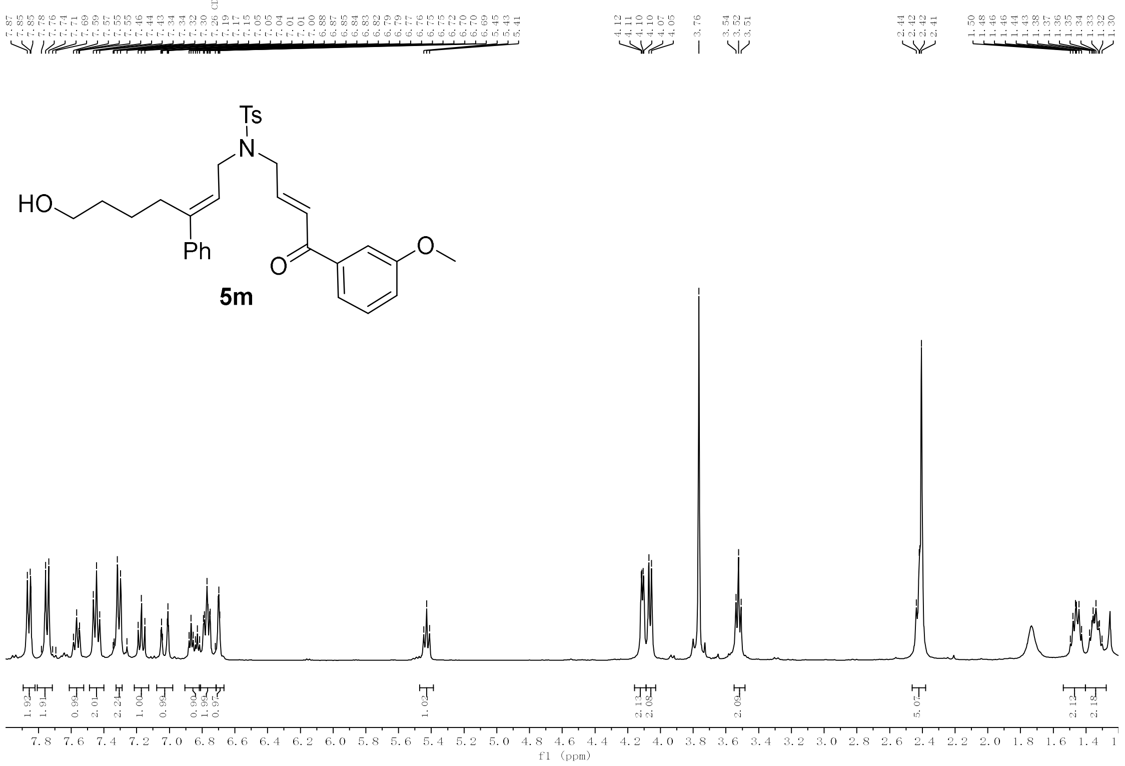

Supplementary Figure 92. ^{13}C NMR spectrum of 5m.

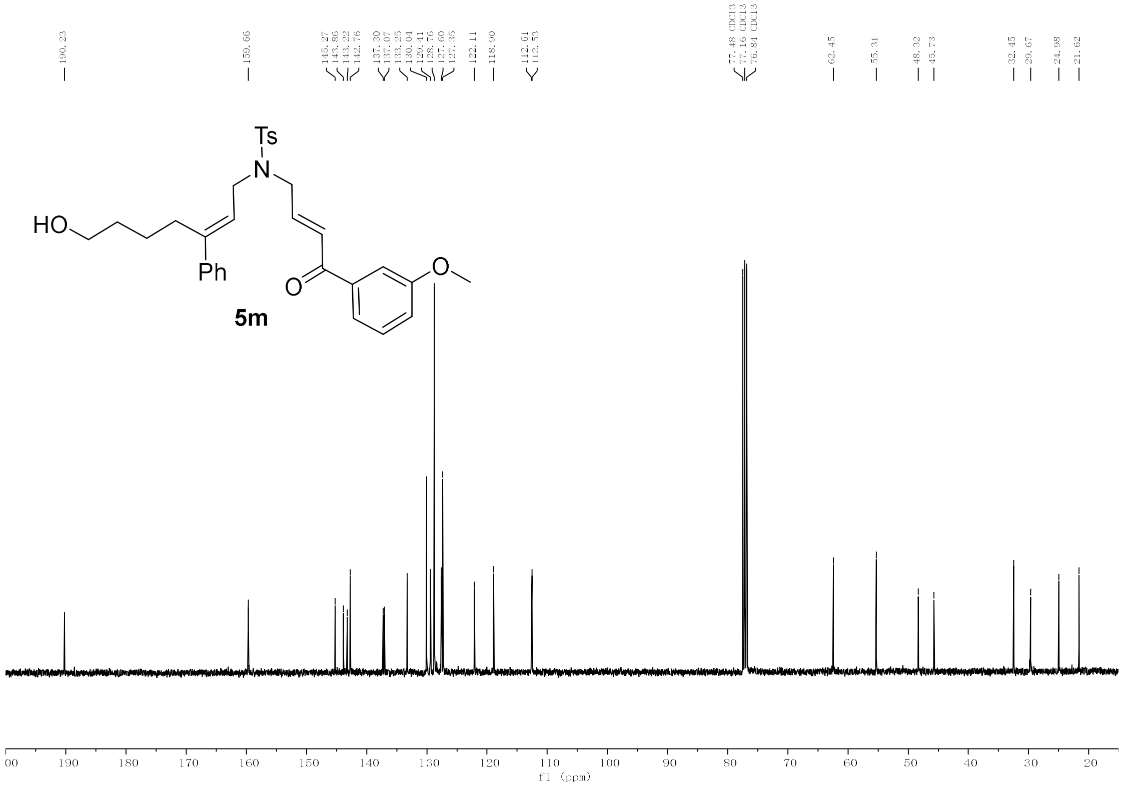

Supplementary Figure 93. ^{1}H NMR spectrum of 5n.

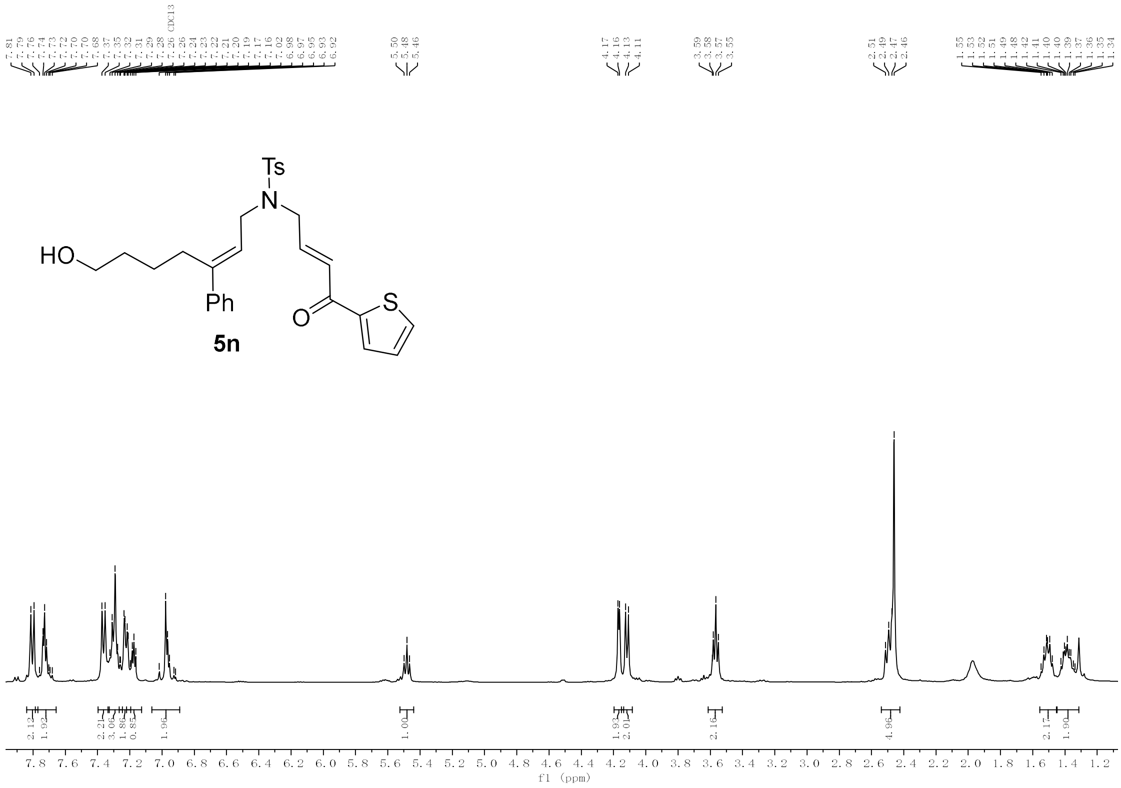

Supplementary Figure 94. ^{13} C NMR spectrum of 5n.

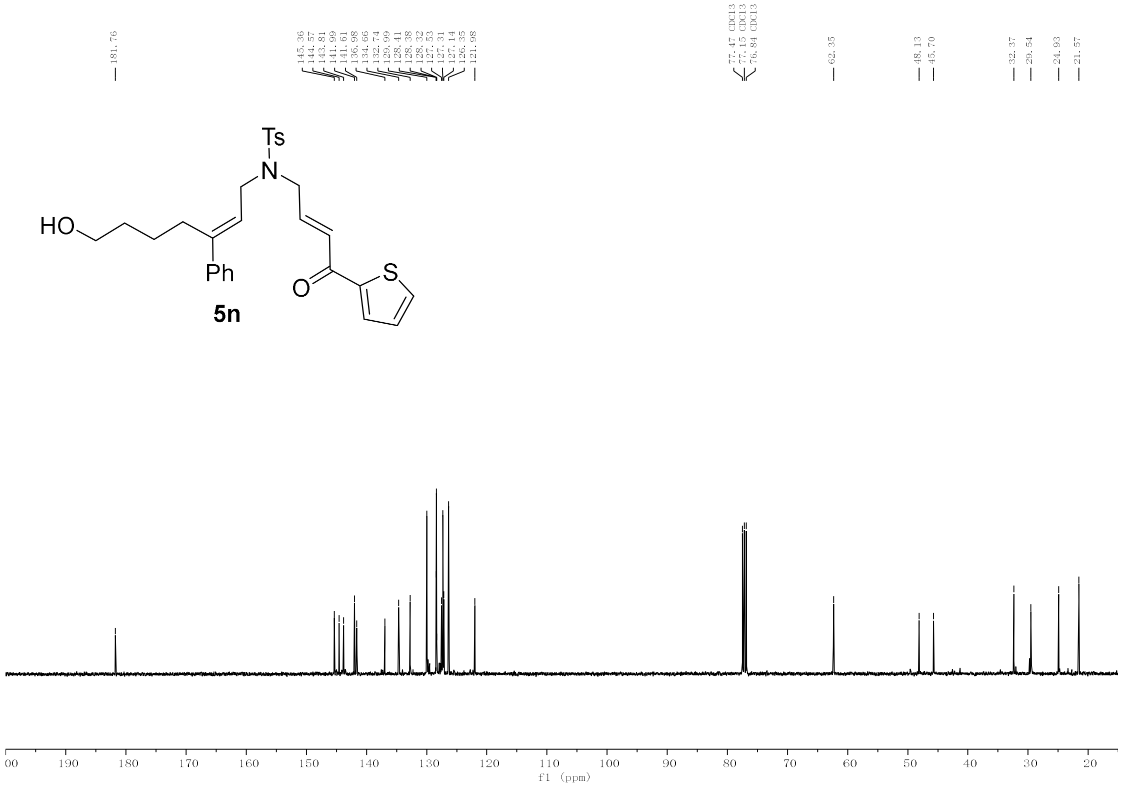

Supplementary Figure 95. ^{1}H NMR spectrum of 5o.

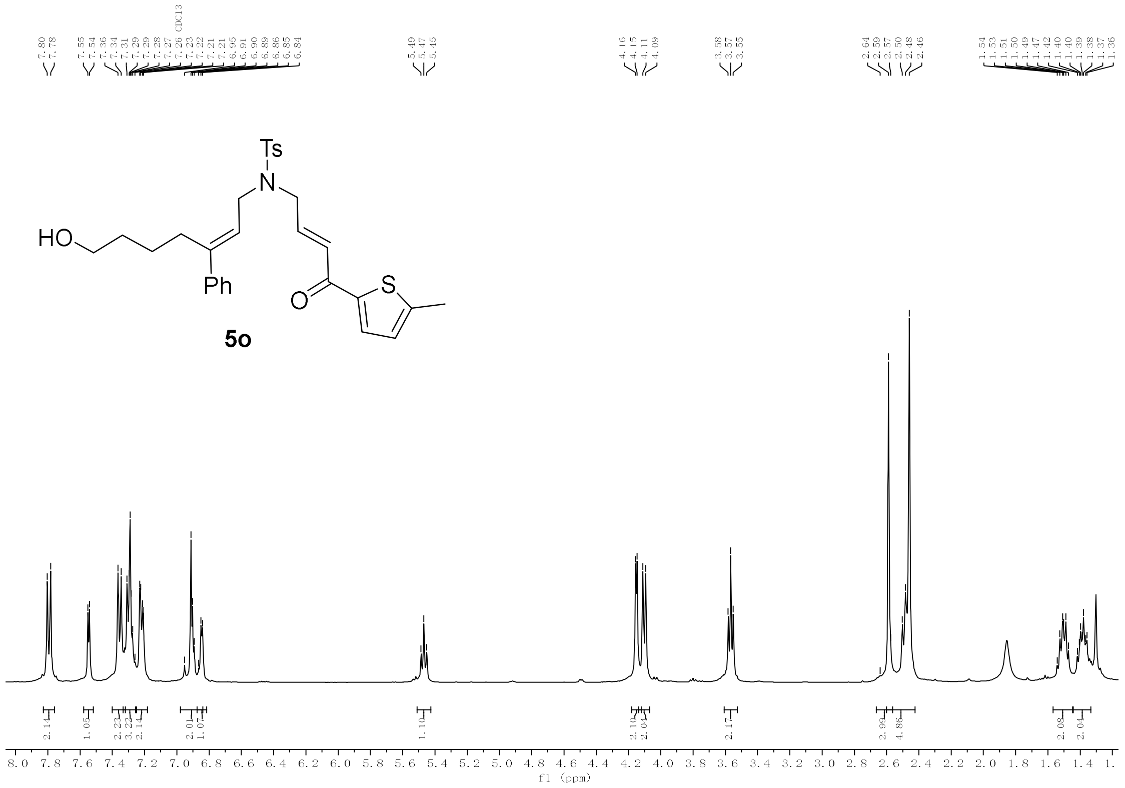

Supplementary Figure 96. ^{13}C NMR spectrum of 5o.

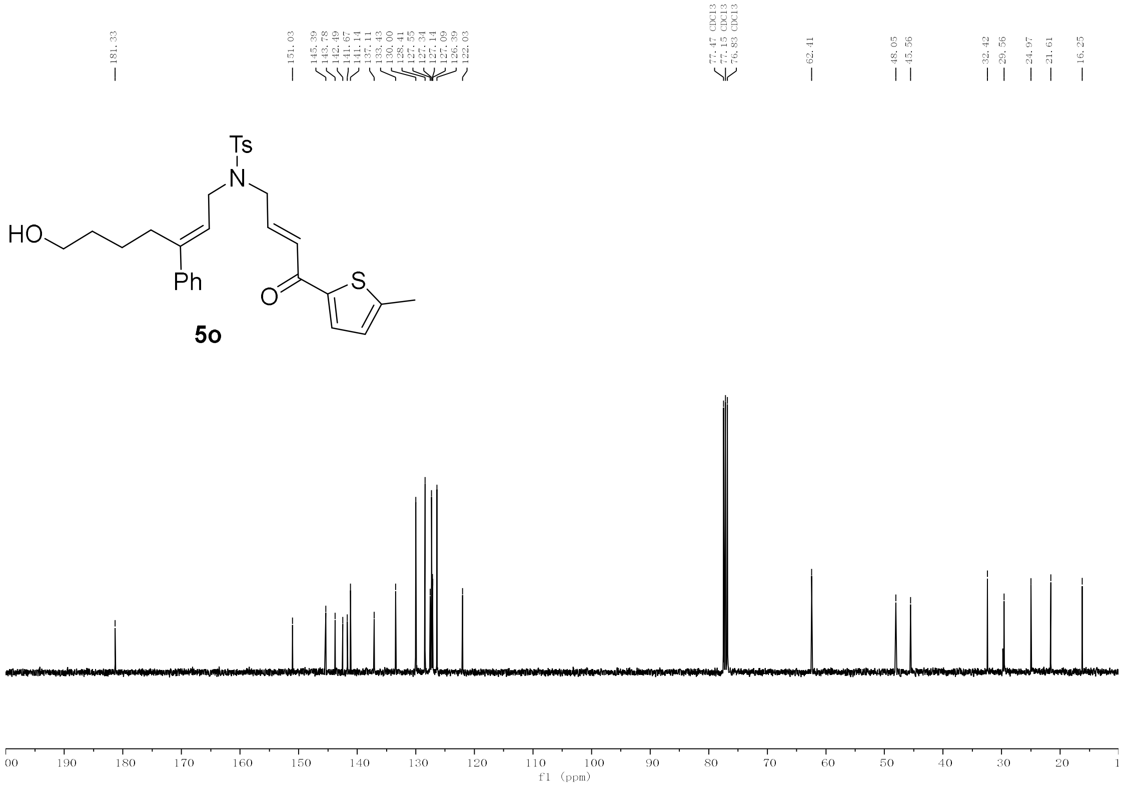

Supplementary Figure 97. ^{1}H NMR spectrum of 5p.

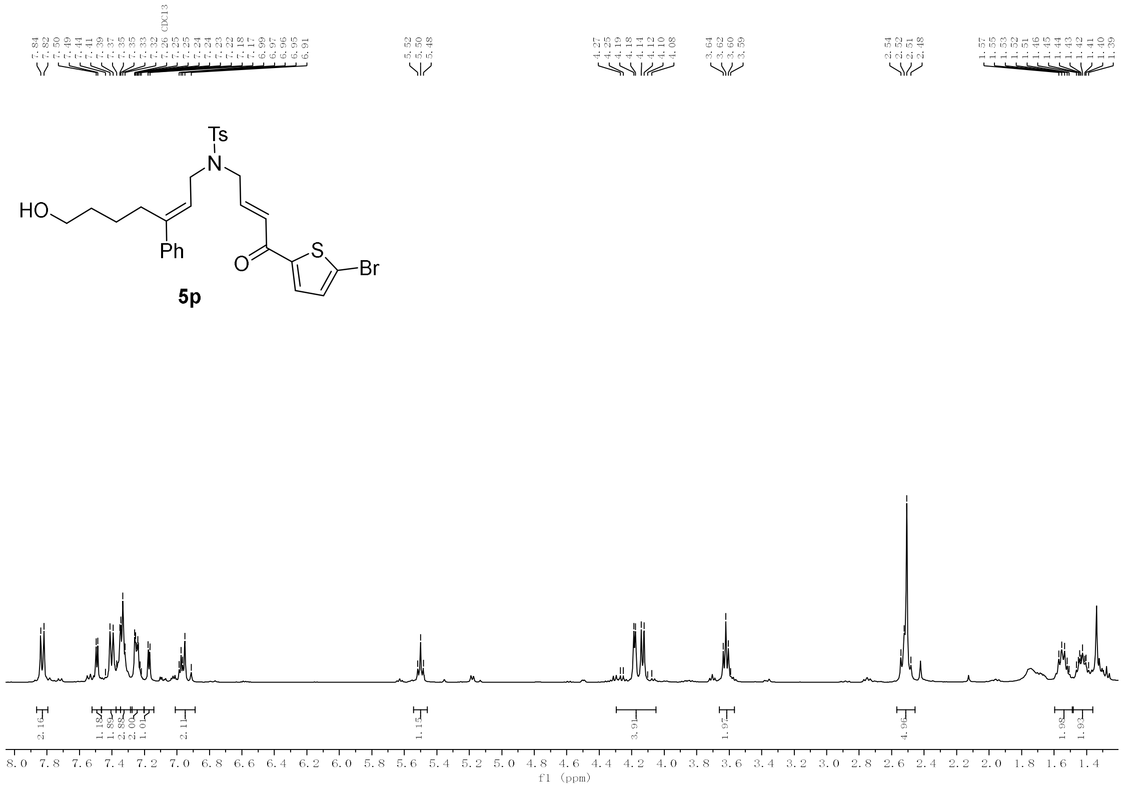

Supplementary Figure 98. ^{13}C NMR spectrum of 5p.

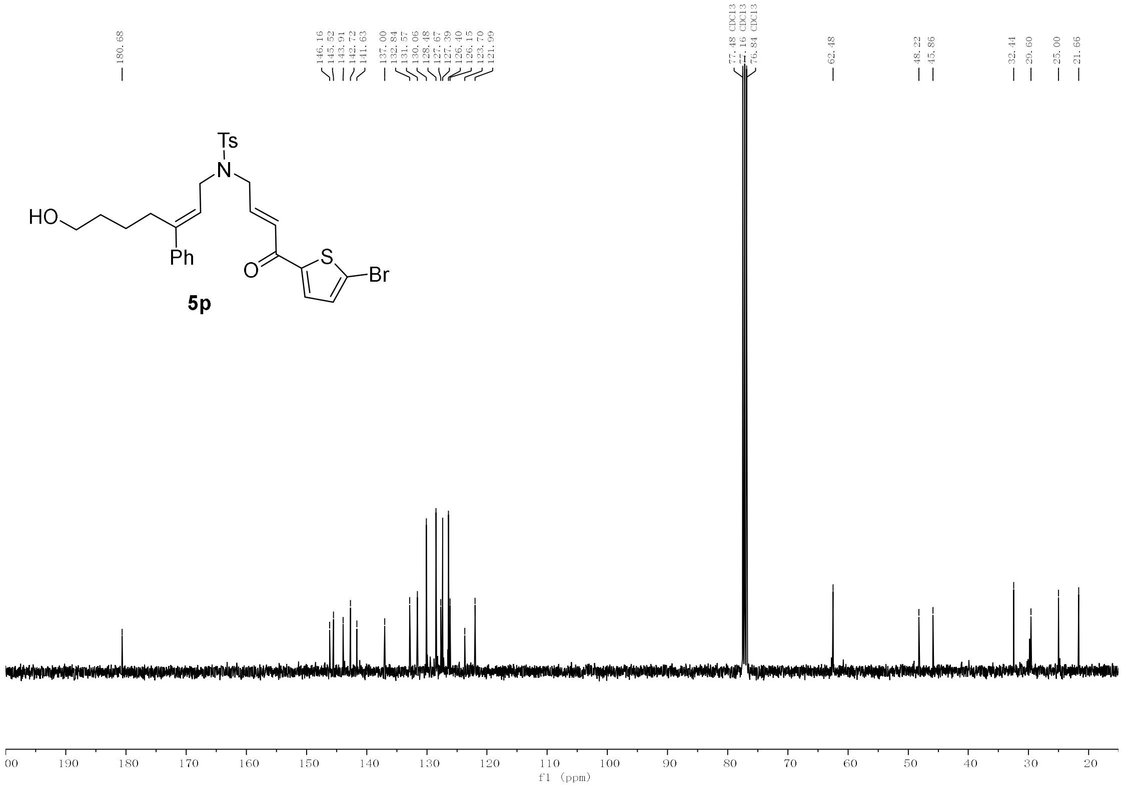

Supplementary Figure 99. ^{1}H NMR spectrum of 5q.

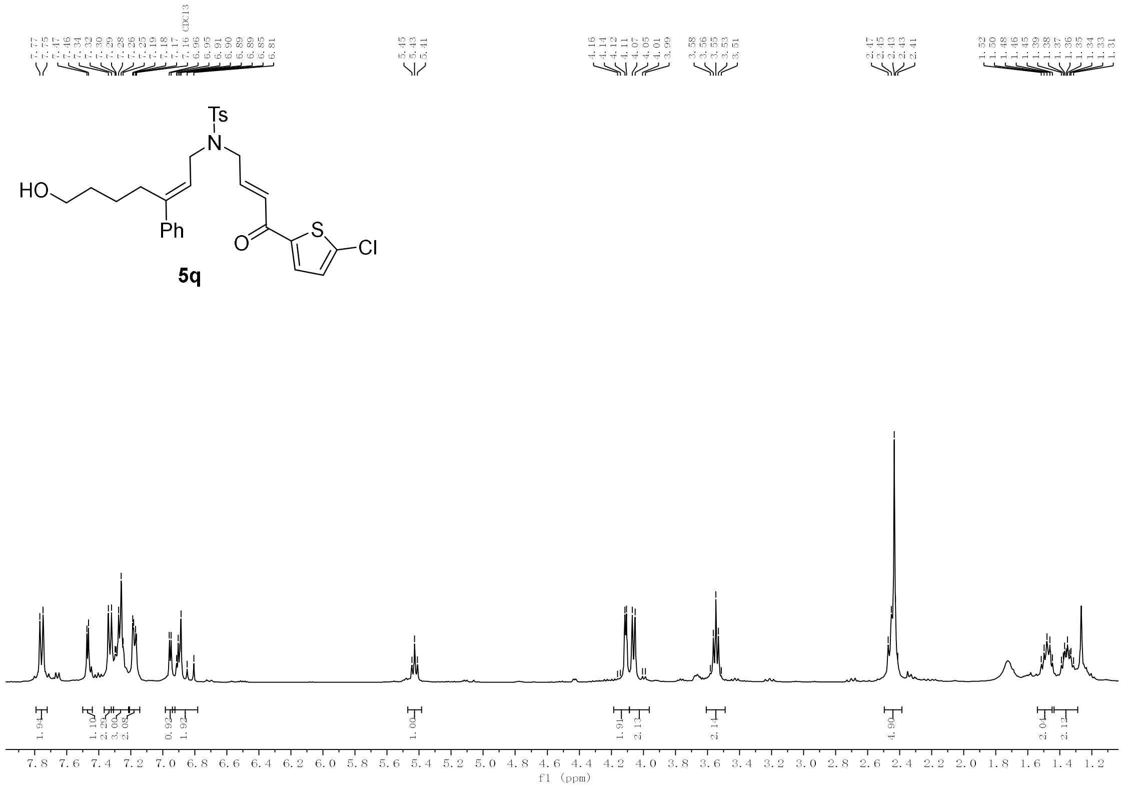

Supplementary Figure 100. ^{13}C NMR spectrum of 5q.

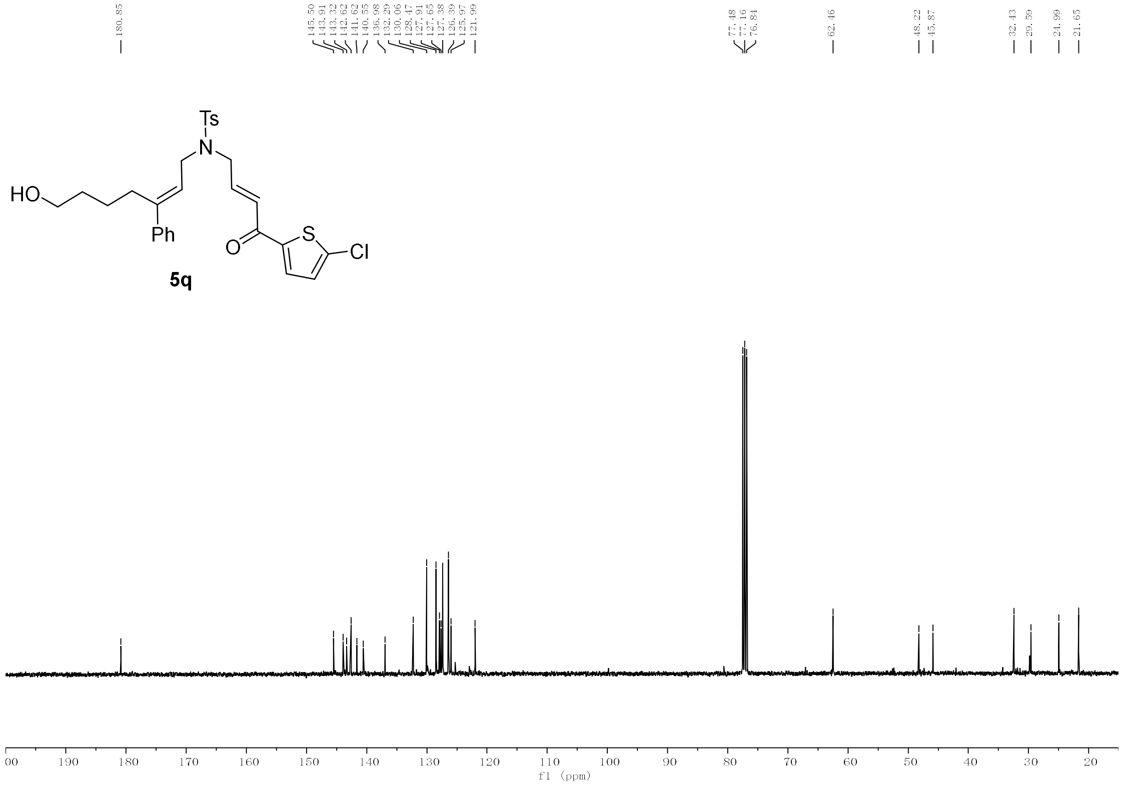

Supplementary Figure 101. ^{1}H NMR spectrum of 5r.

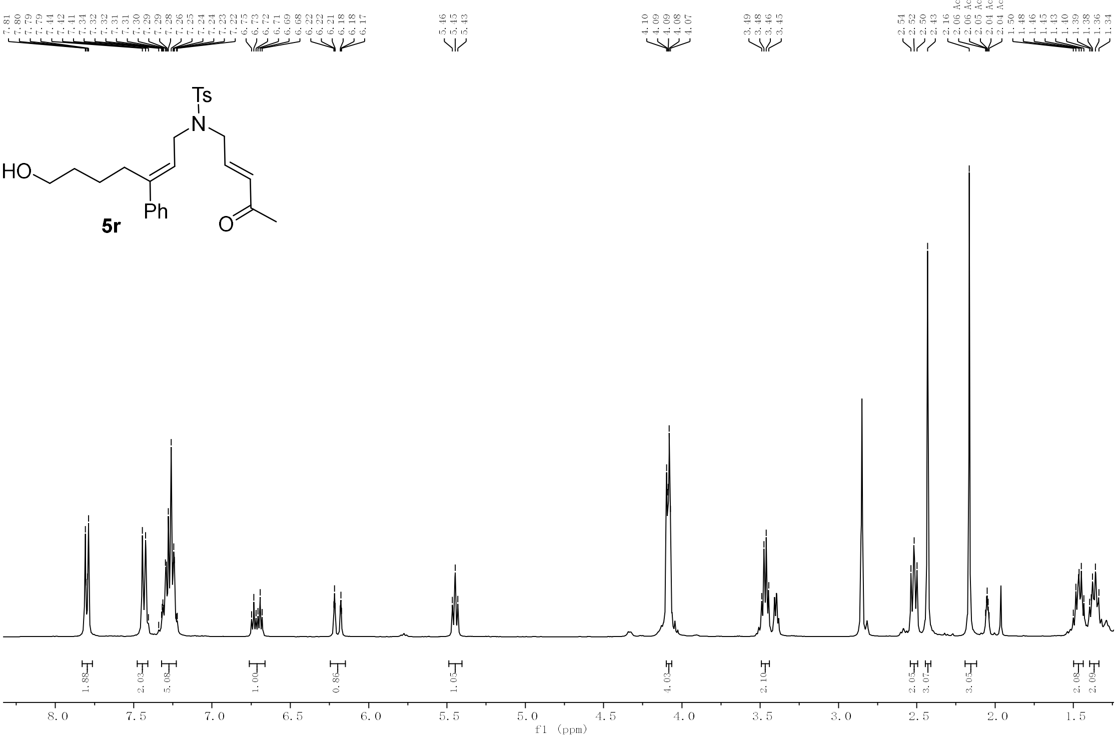

Supplementary Figure 102. ^{13}C NMR spectrum of 5r.

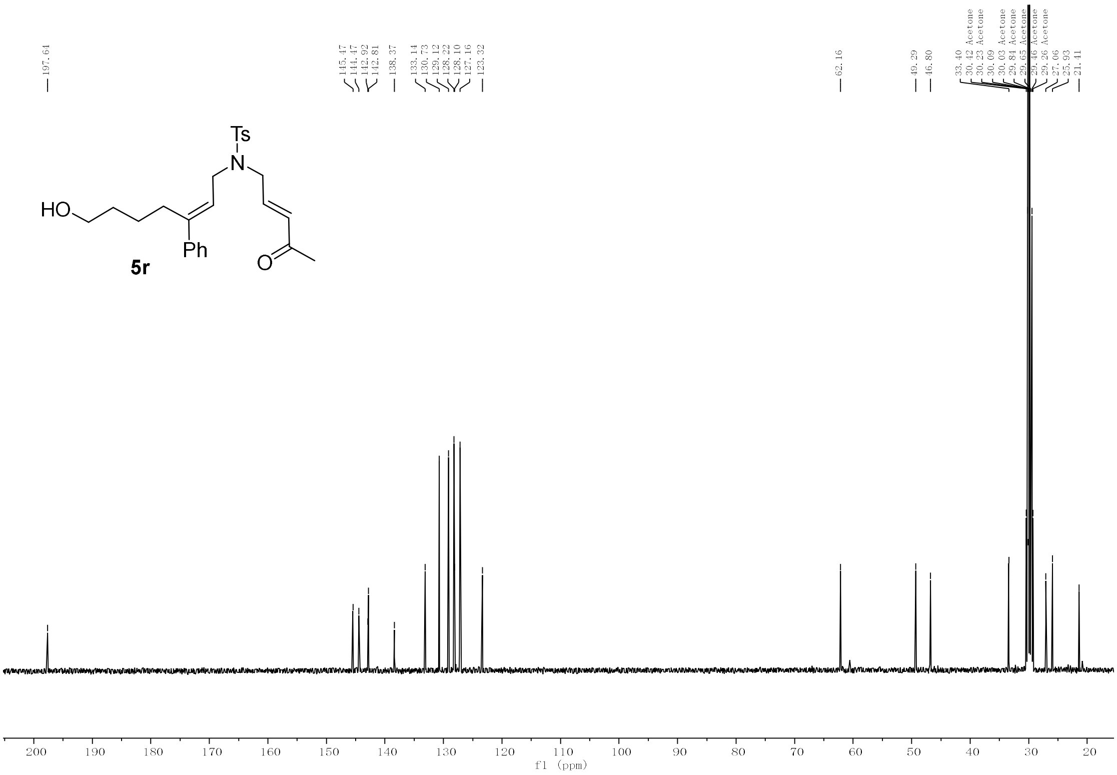

Supplementary Figure 103. ^{1}H NMR spectrum of 5s.

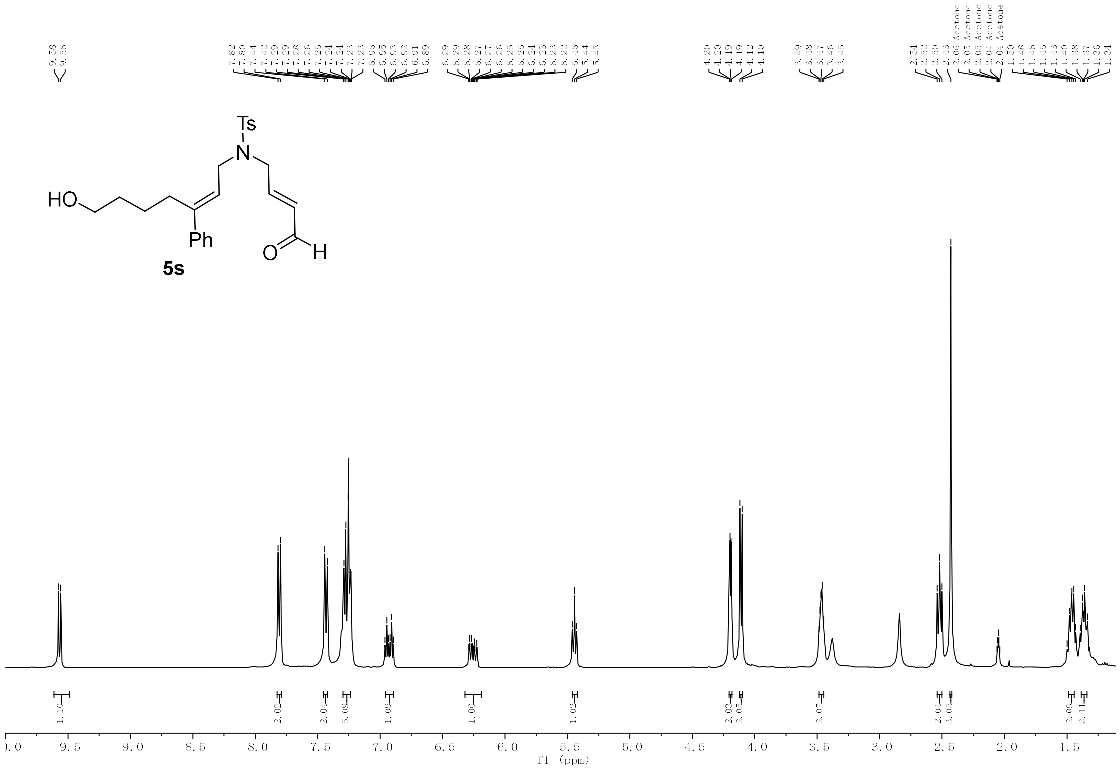

Supplementary Figure 104. ^{13}C NMR spectrum of 5s.

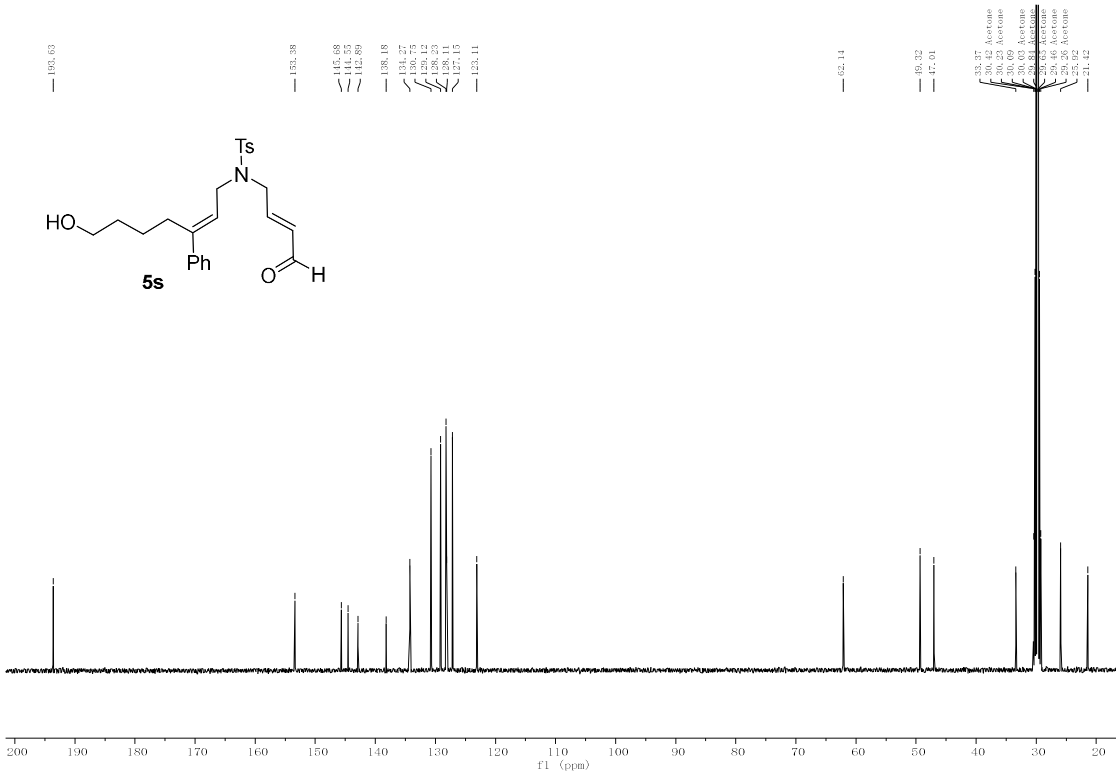

Supplementary Figure 105. ^{1}H NMR spectrum of 5t.

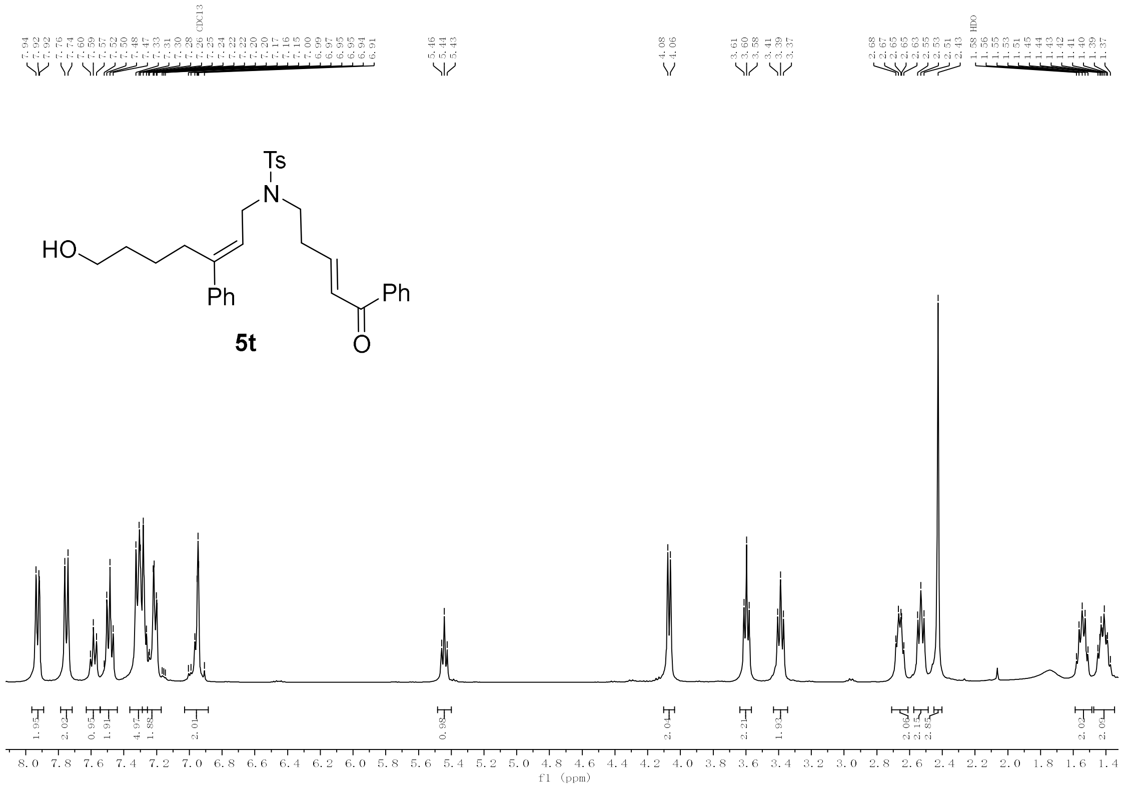

Supplementary Figure 106. ^{13}C NMR spectrum of 5t.

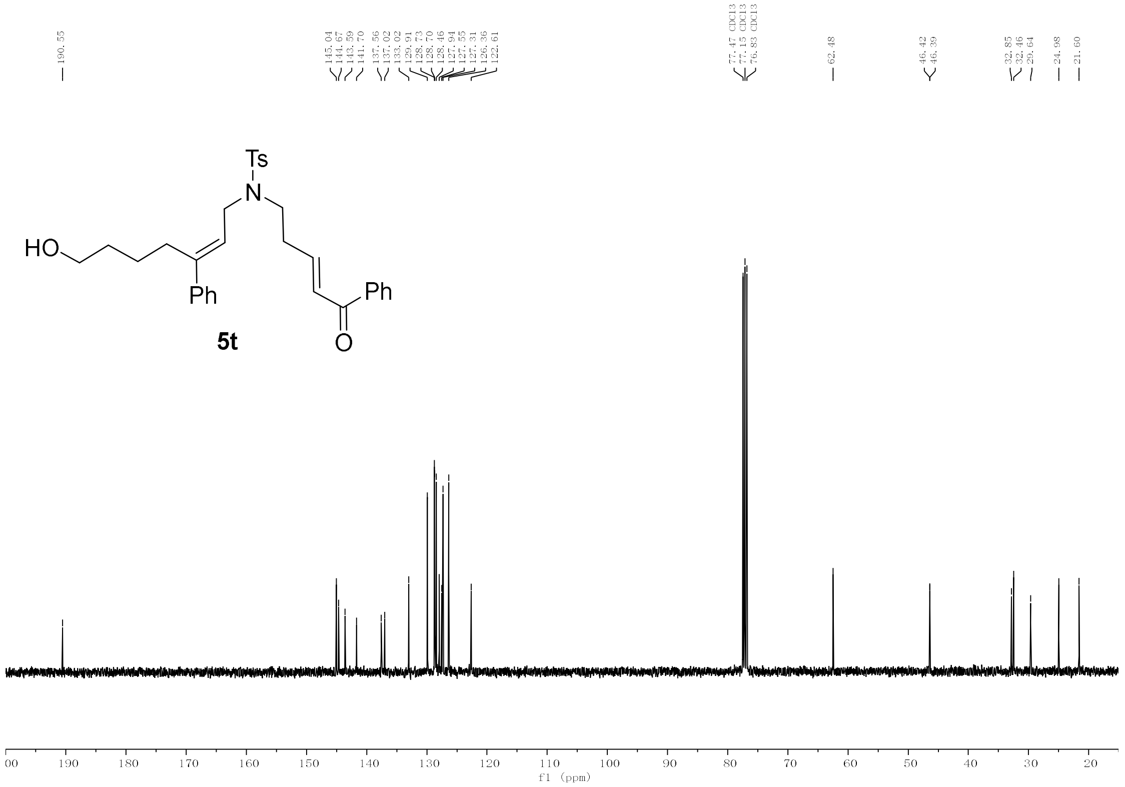

Supplementary Figure 107. ^{1}H NMR spectrum of 7a.

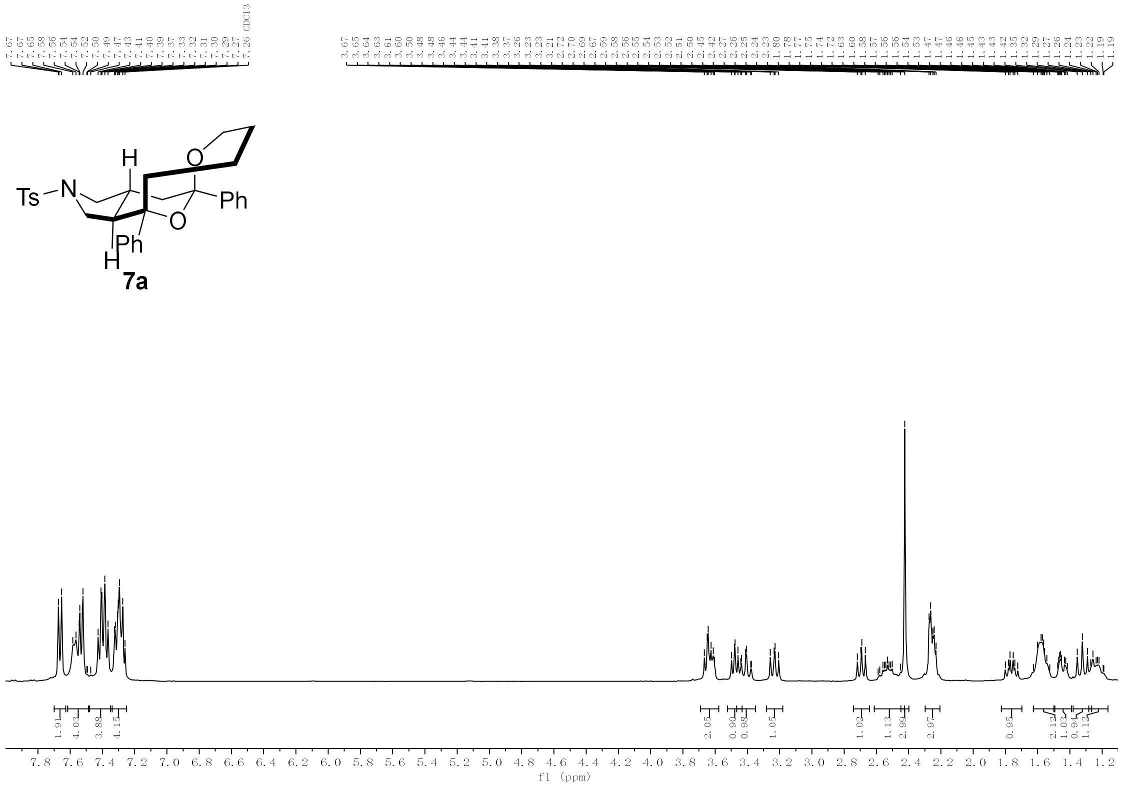

Supplementary Figure 108. ^{13}C NMR spectrum of 7a.

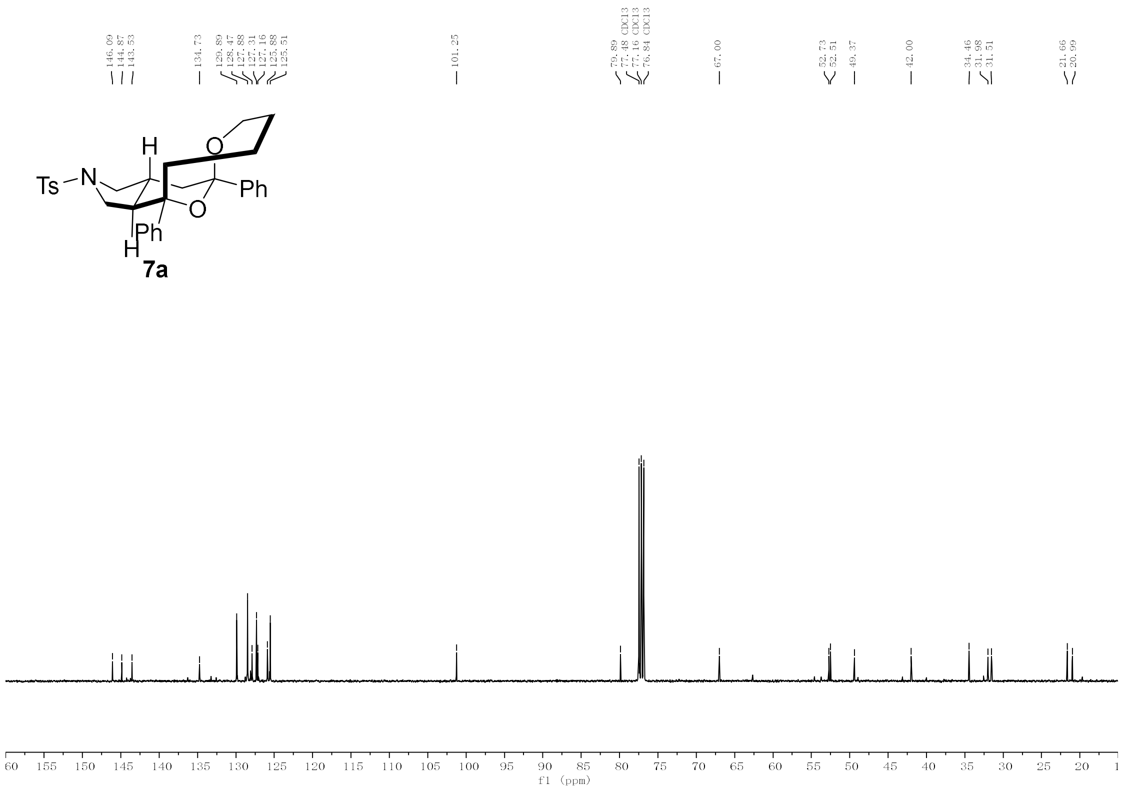

Supplementary Figure 109. [1] H NMR spectrum of 7b.

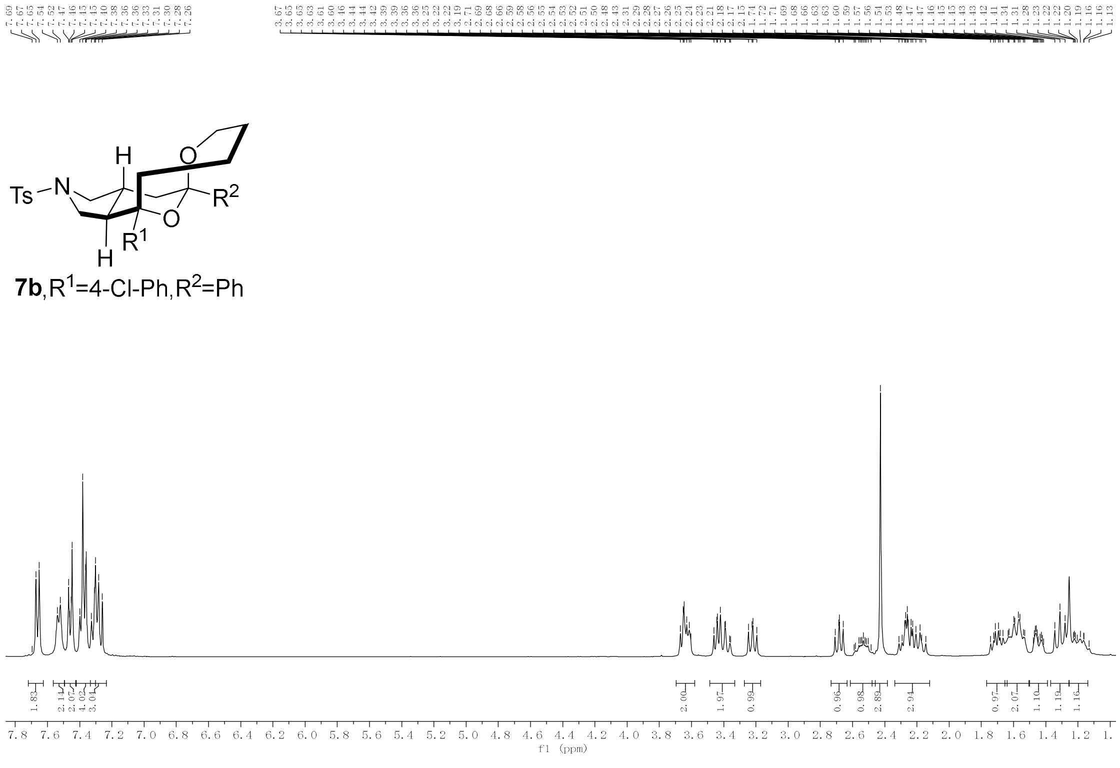

Supplementary Figure 110. [13] C NMR spectrum of 7b.

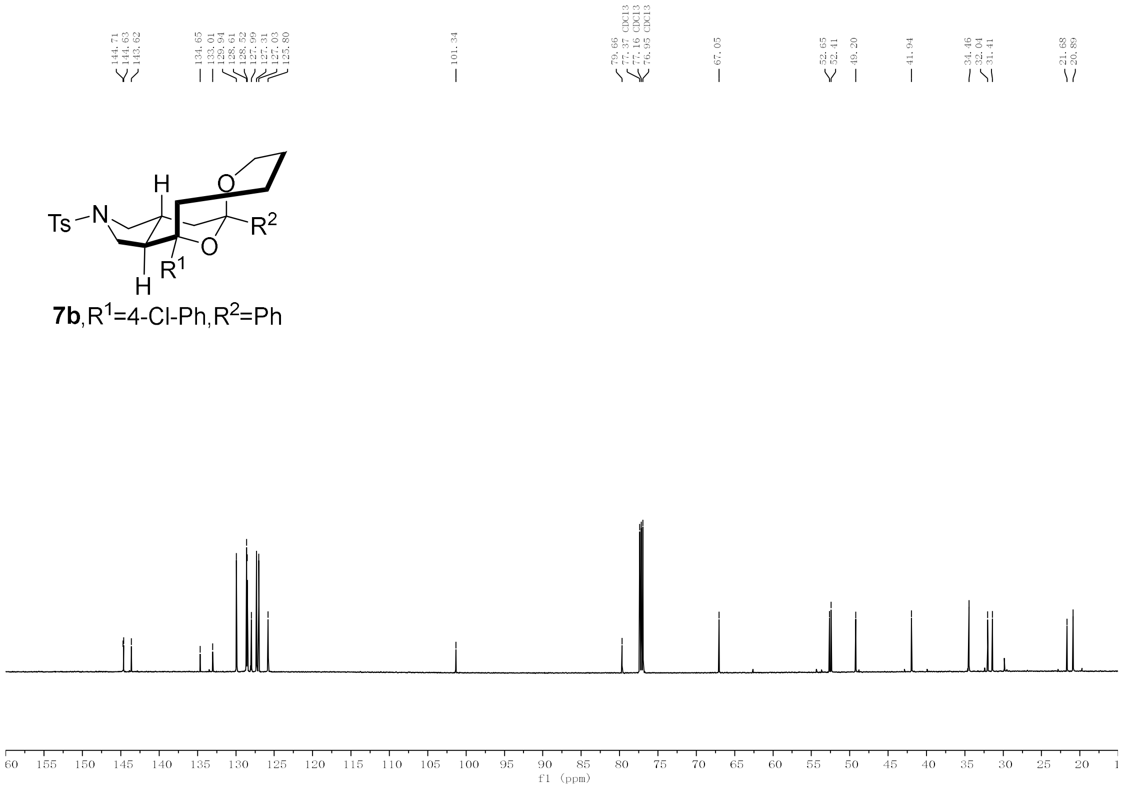

Supplementary Figure 111. ^{1}H NMR spectrum of 7c.

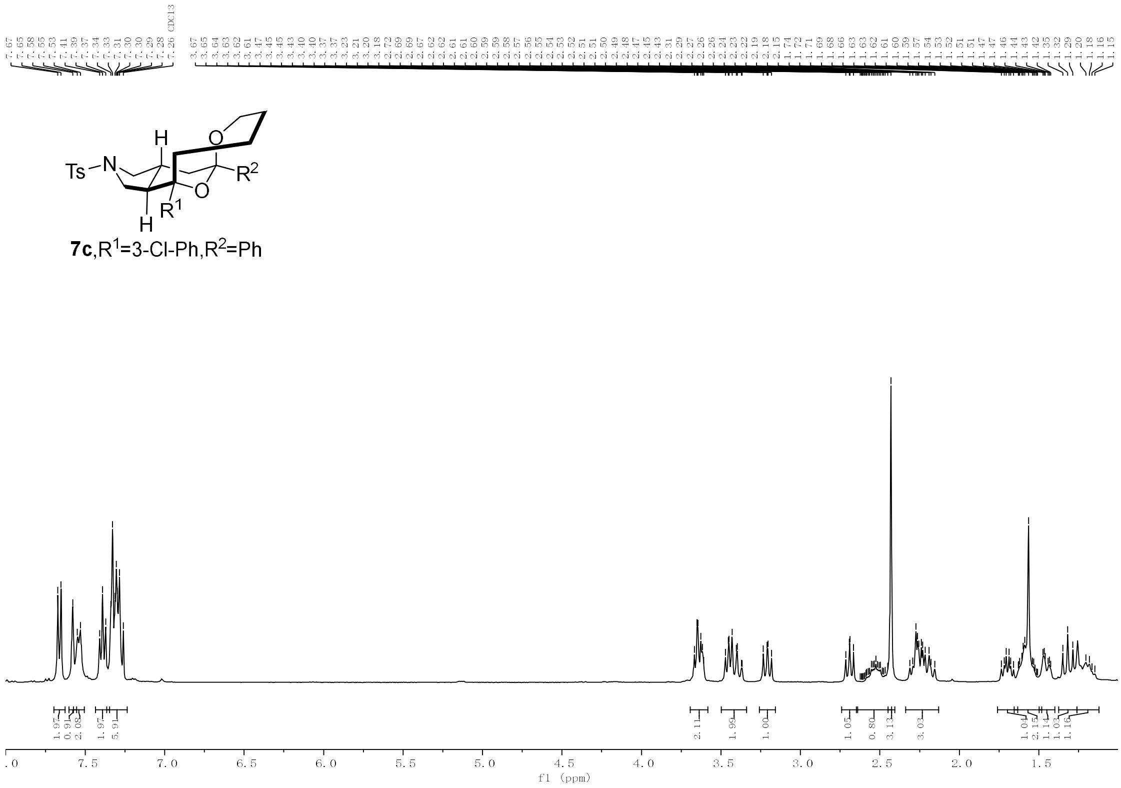

Supplementary Figure 112. ^{13}C NMR spectrum of 7c.

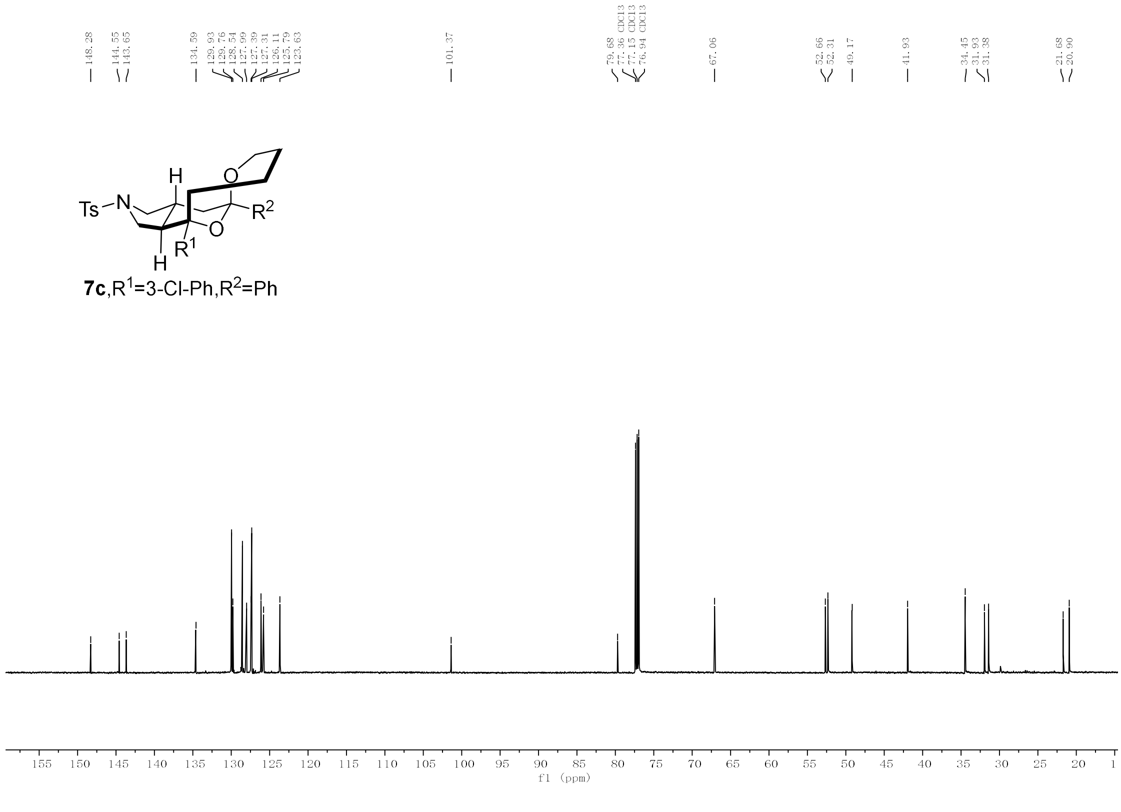

Supplementary Figure 113. ^{1}H NMR spectrum of 7d.

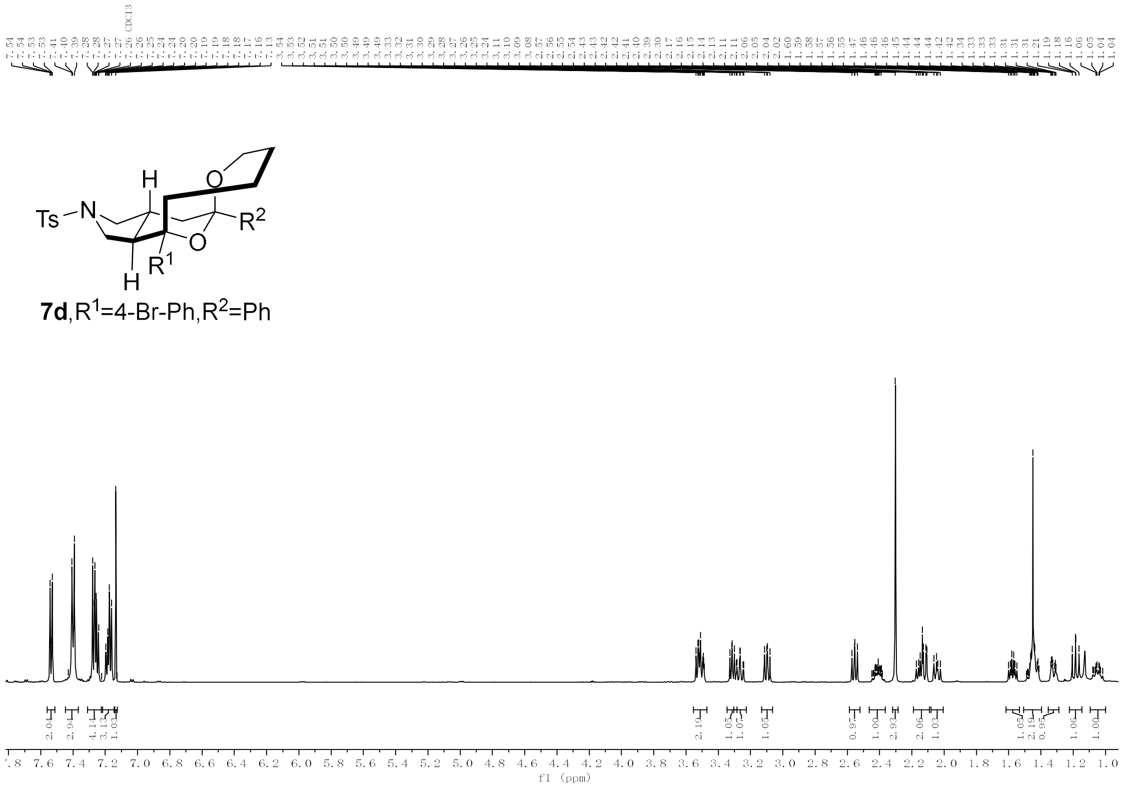

Supplementary Figure 114. ^{13}C NMR spectrum of 7d.

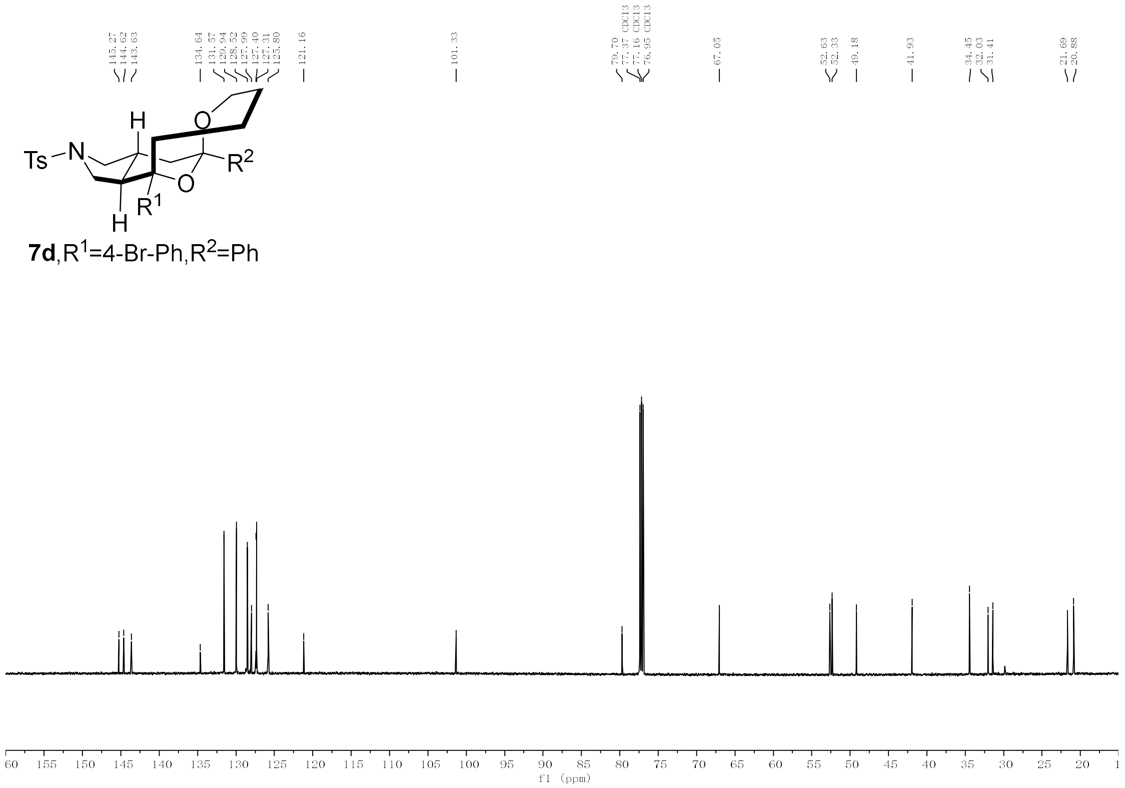

Supplementary Figure 115. ^{1}H NMR spectrum of 7e.

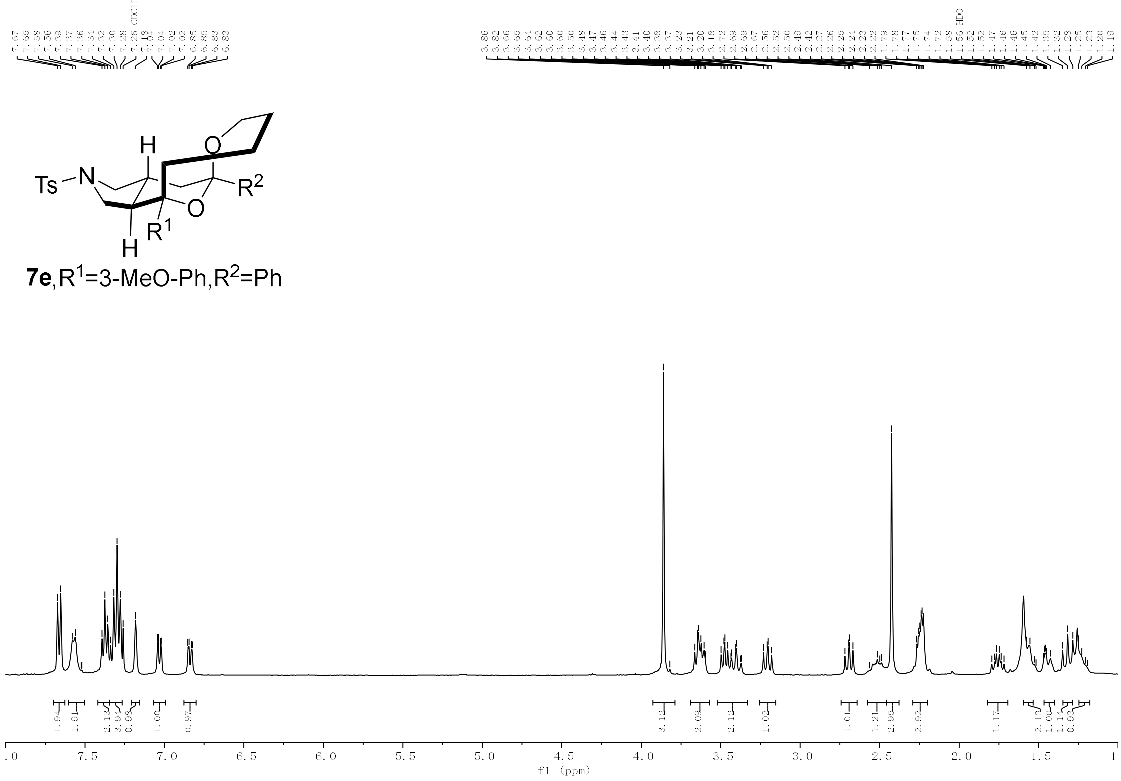

Supplementary Figure 116. ^{13}C NMR spectrum of 7e.

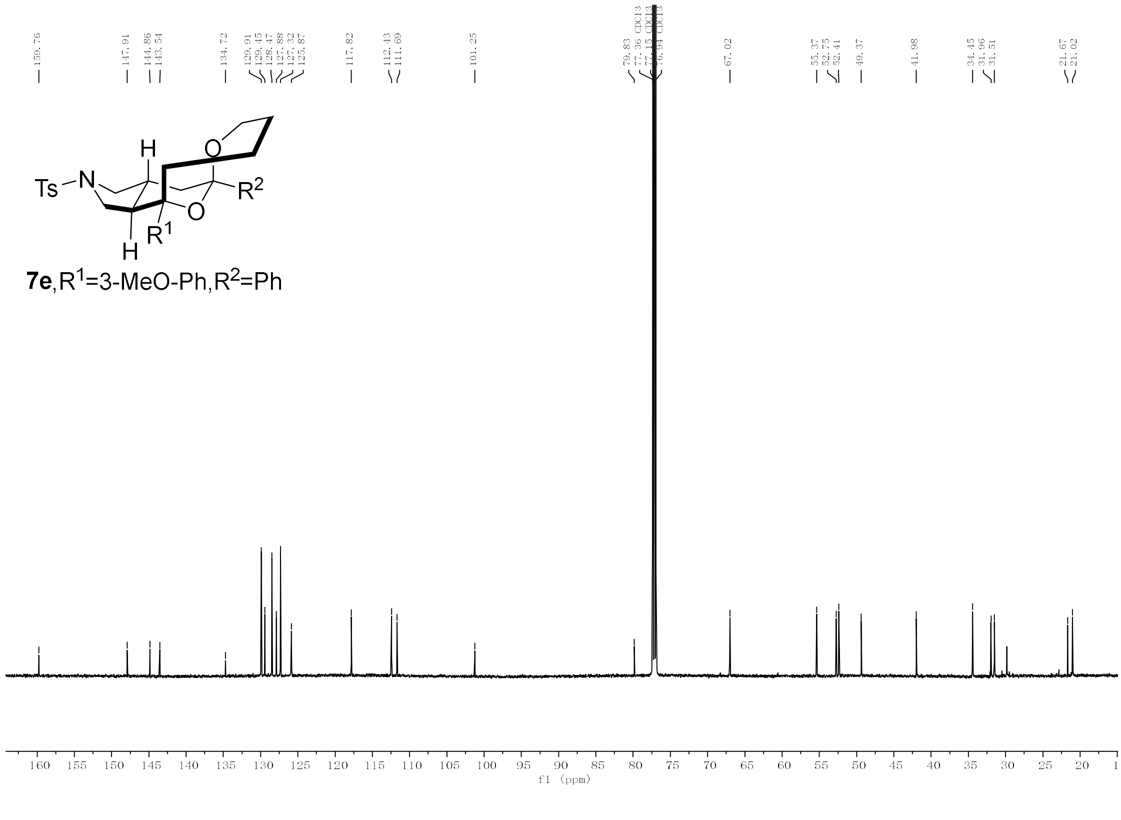

Supplementary Figure 117. ^{1}H NMR spectrum of 7f.

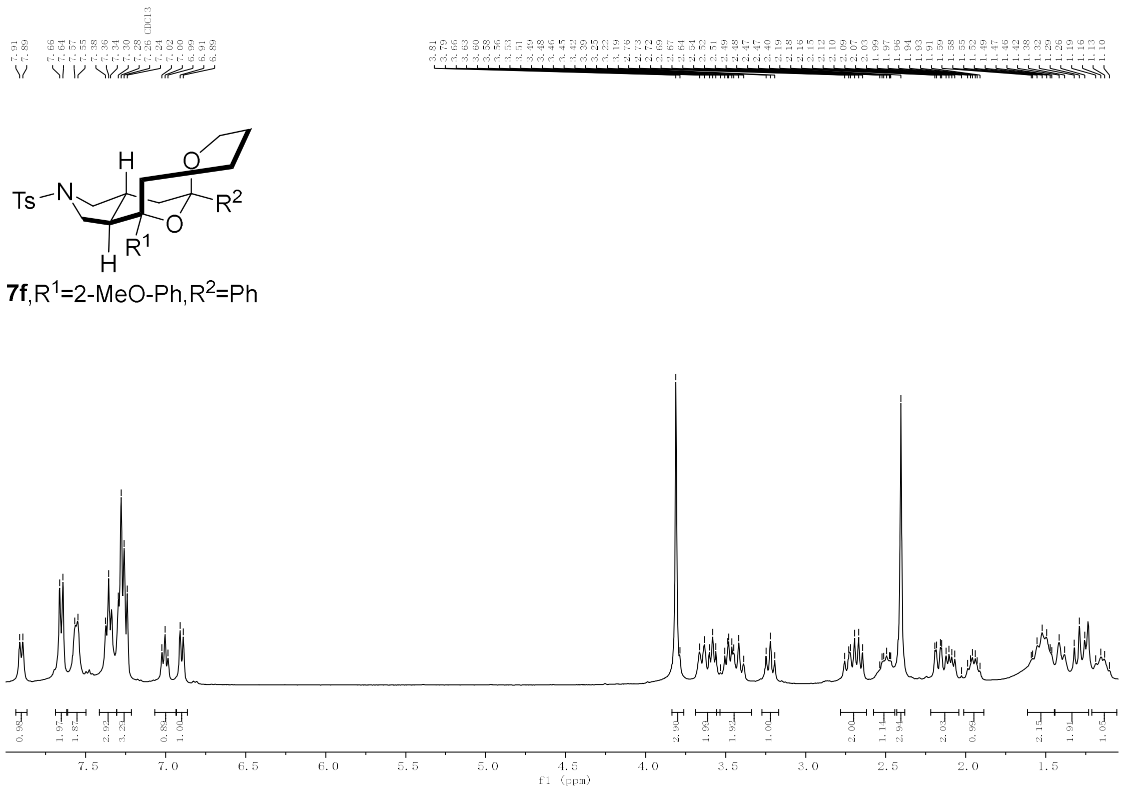

Supplementary Figure 118. ^{13}C NMR spectrum of 7f.

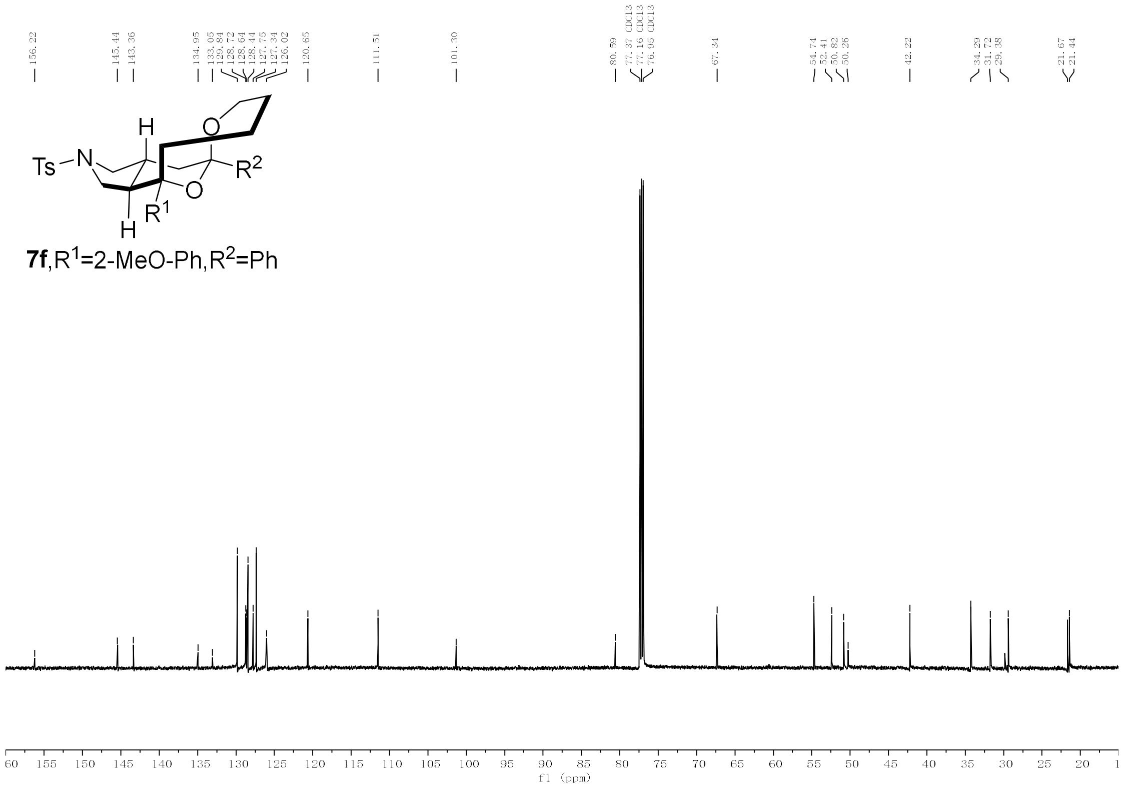

Supplementary Figure 119. ^{1}H NMR spectrum of 7g.

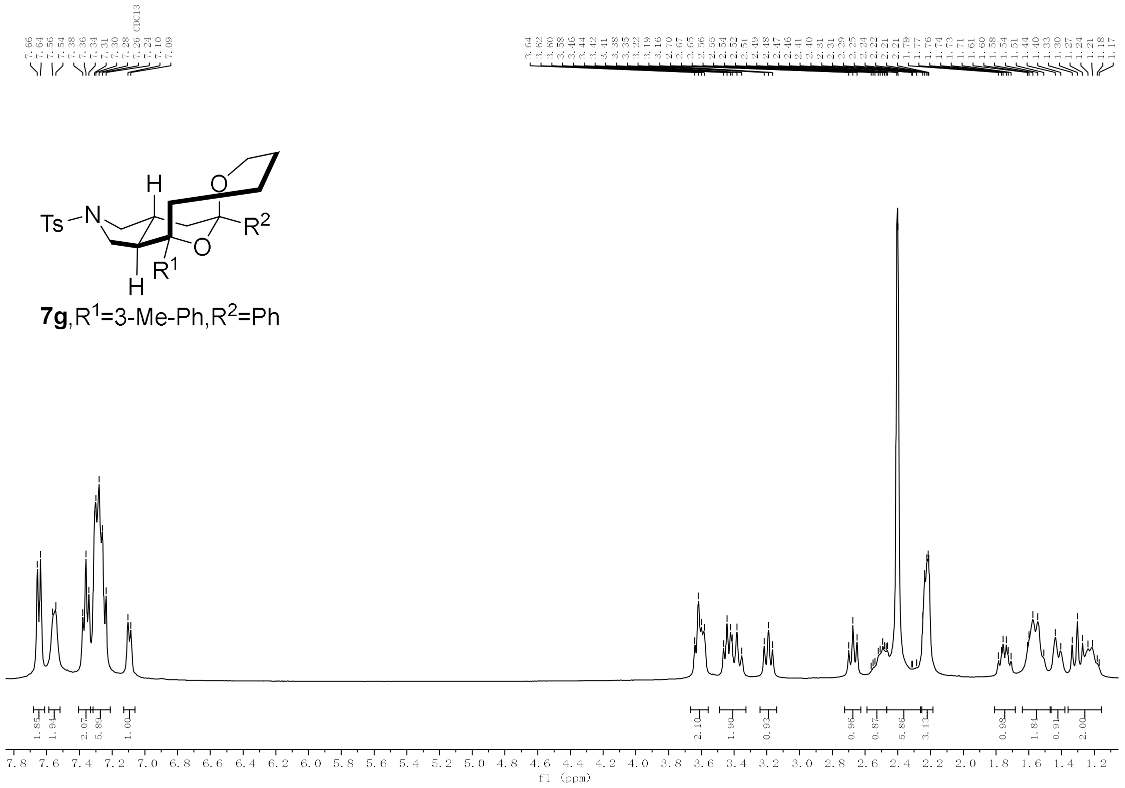

Supplementary Figure 120. ^{13}C NMR spectrum of 7g.

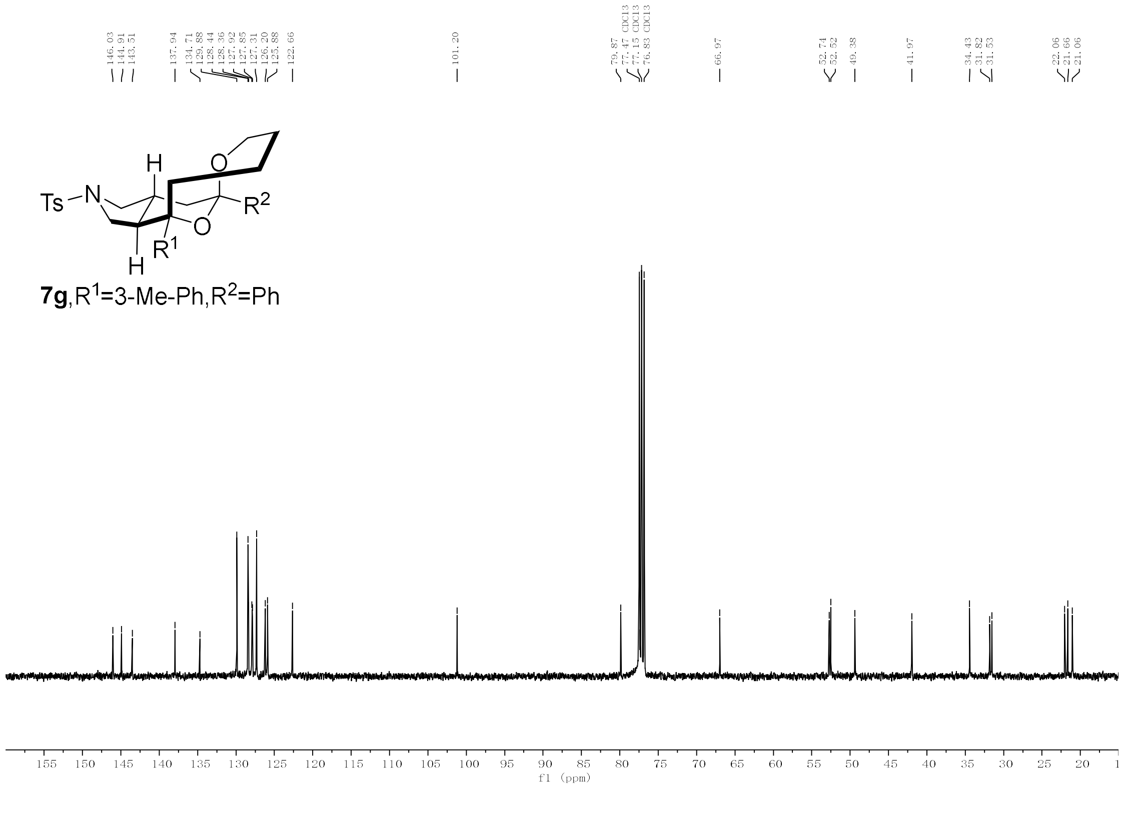

Supplementary Figure 121. ^{1}H NMR spectrum of 7h.

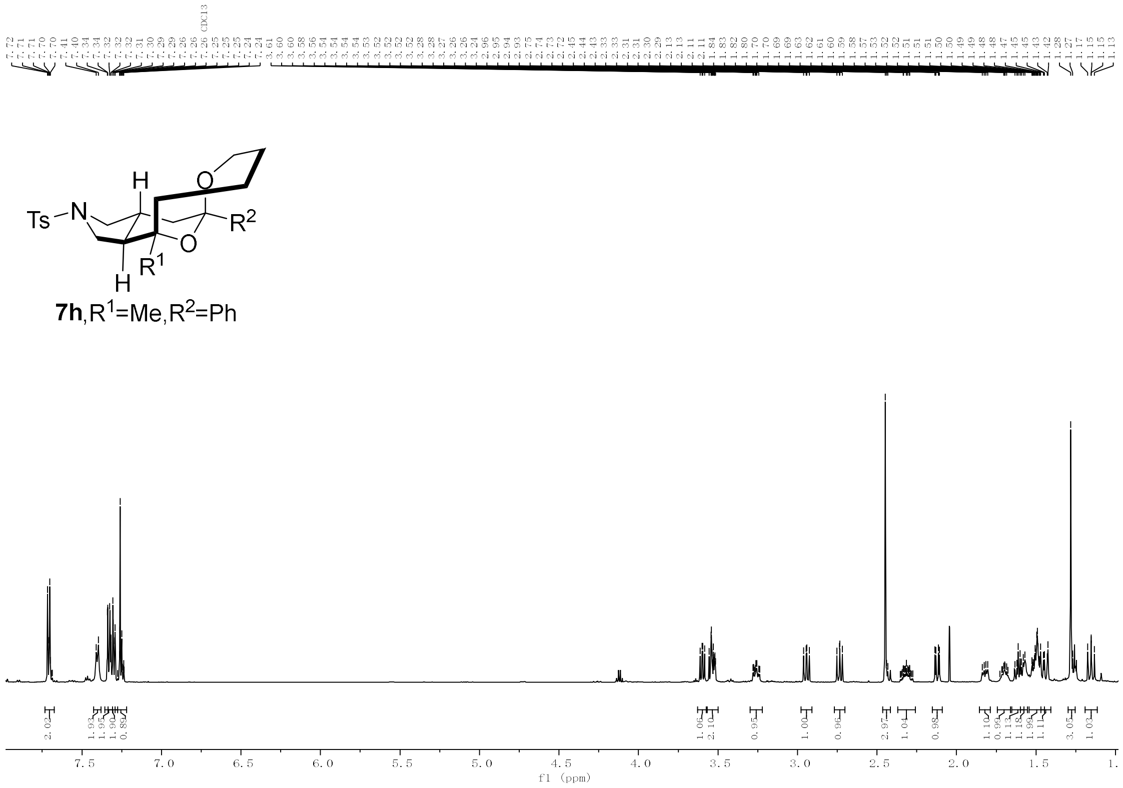

Supplementary Figure 122. ^{13}C NMR spectrum of 7h.

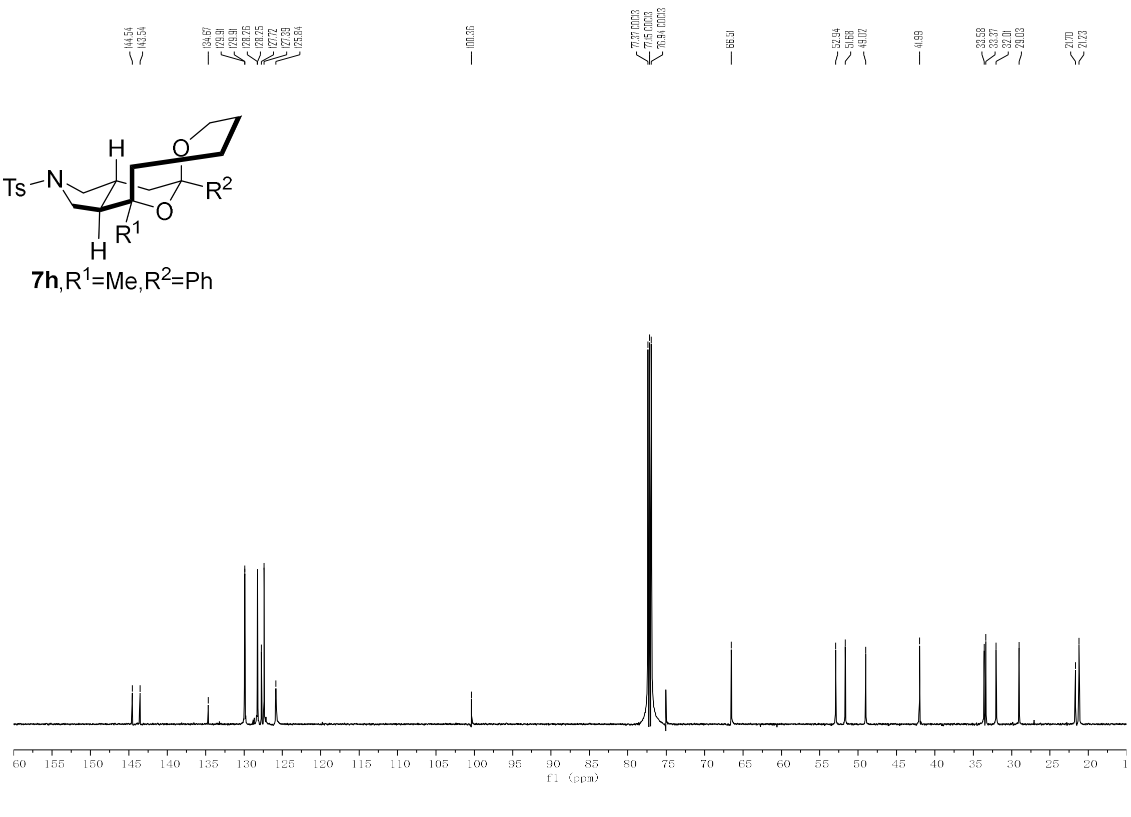

Supplementary Figure 123. ^{1}H NMR spectrum of 7i.

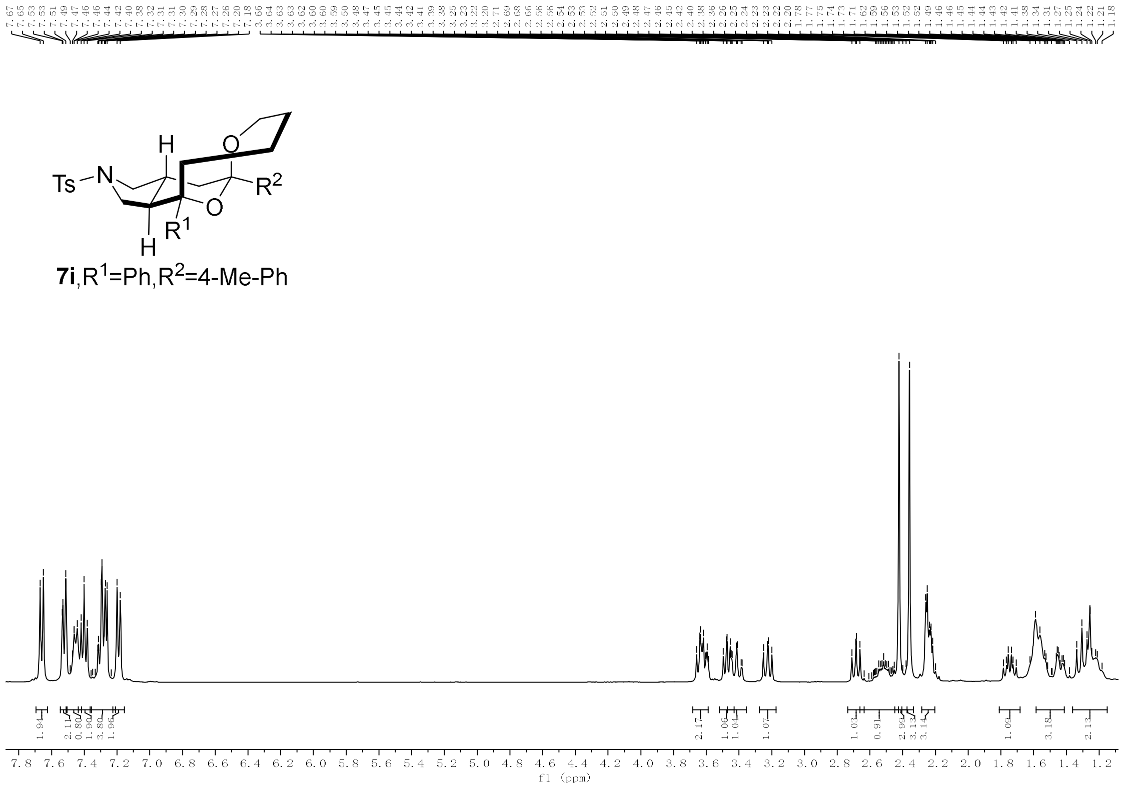

Supplementary Figure 124. ^{13}C NMR spectrum of 7i.

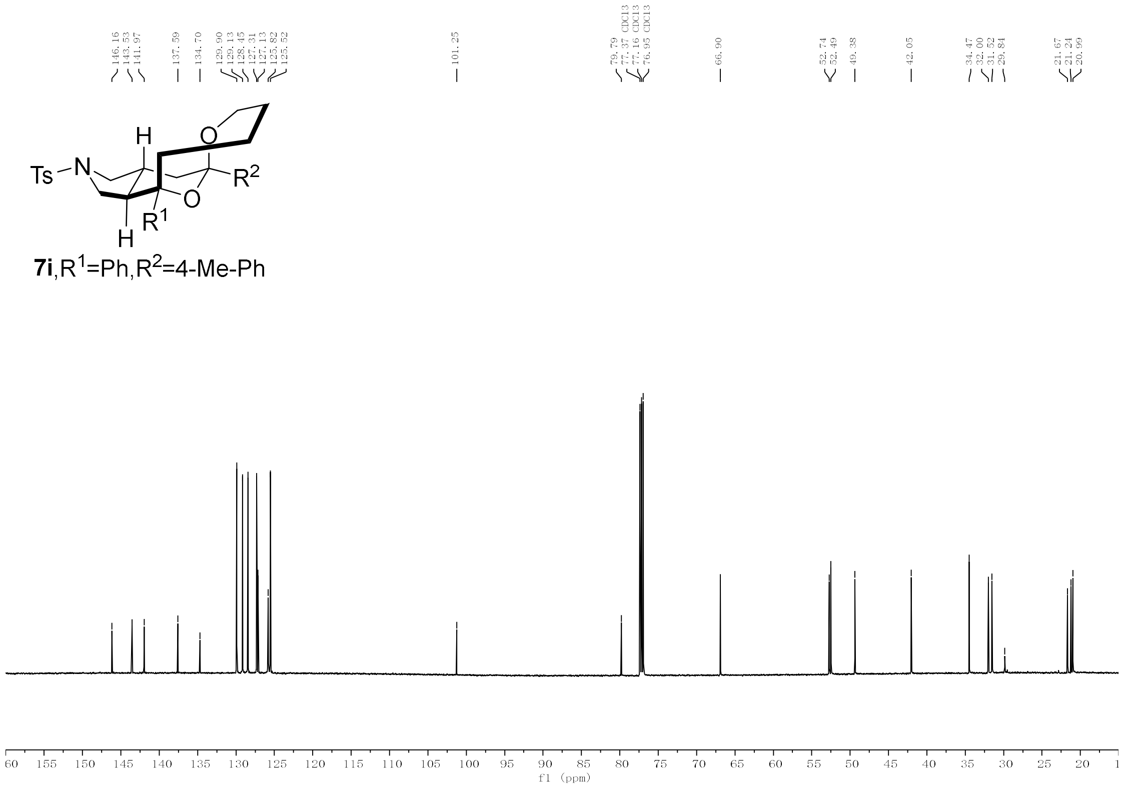

Supplementary Figure 125. ^{1}H NMR spectrum of 7j.

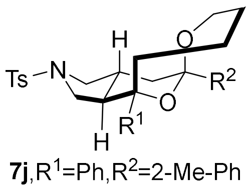

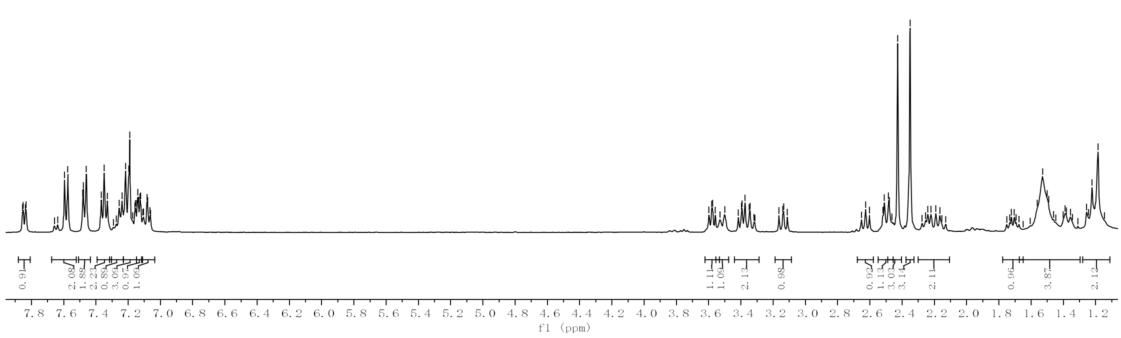

Supplementary Figure 126. ^{13}C NMR spectrum of 7j.

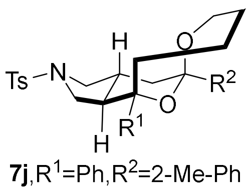

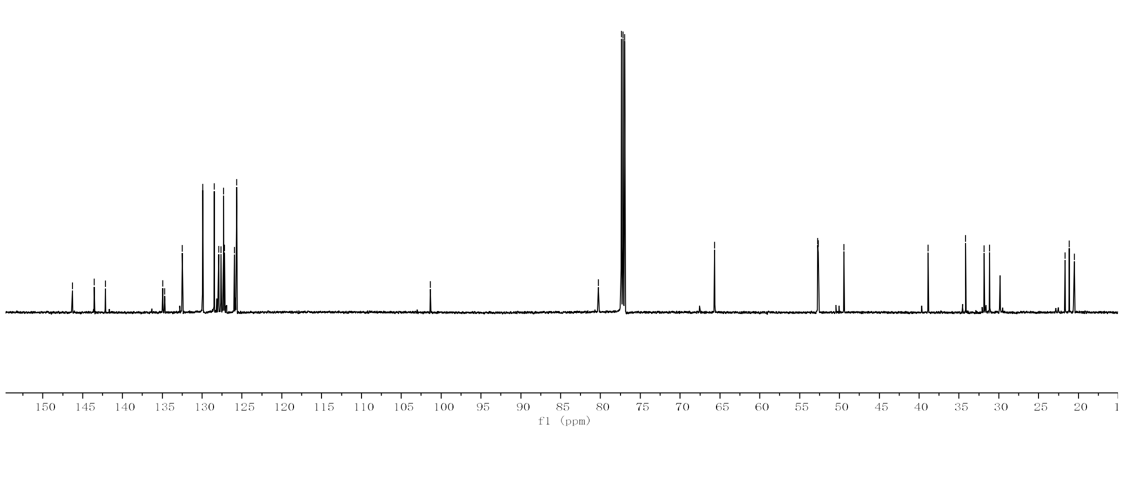

Supplementary Figure 127. ^{1}H NMR spectrum of 7k.

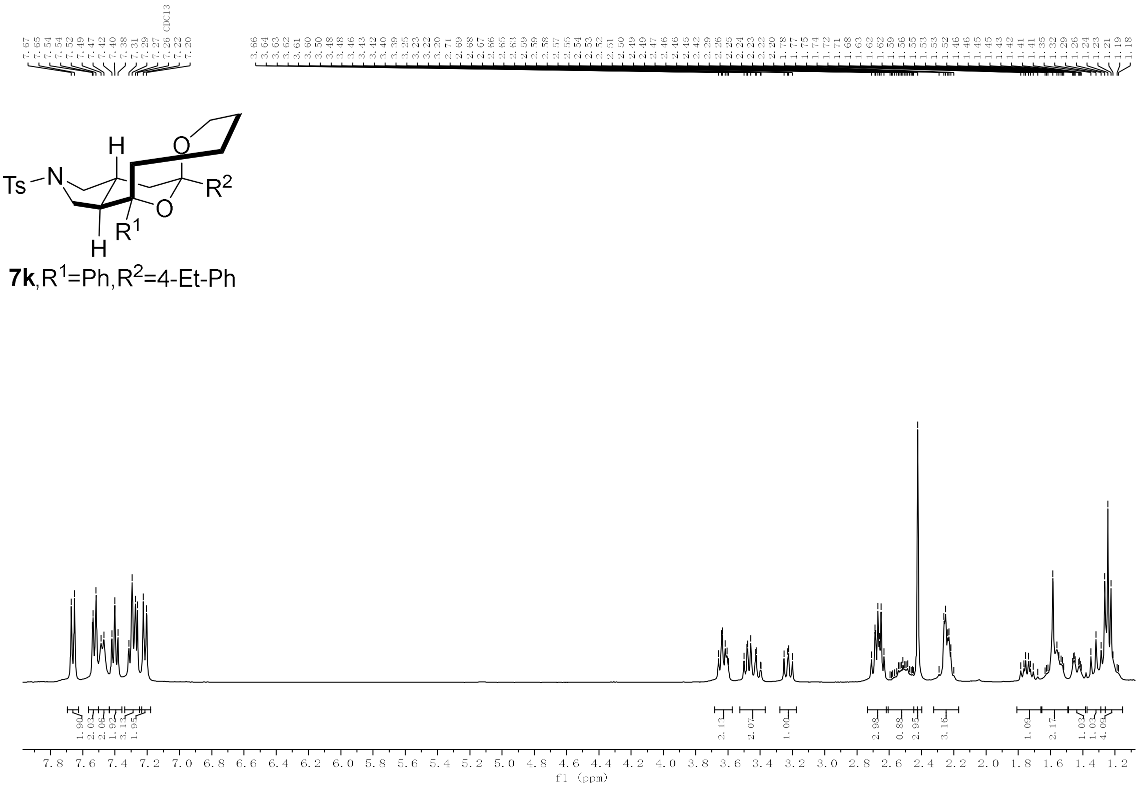

Supplementary Figure 128. ^{13}C NMR spectrum of 7k.

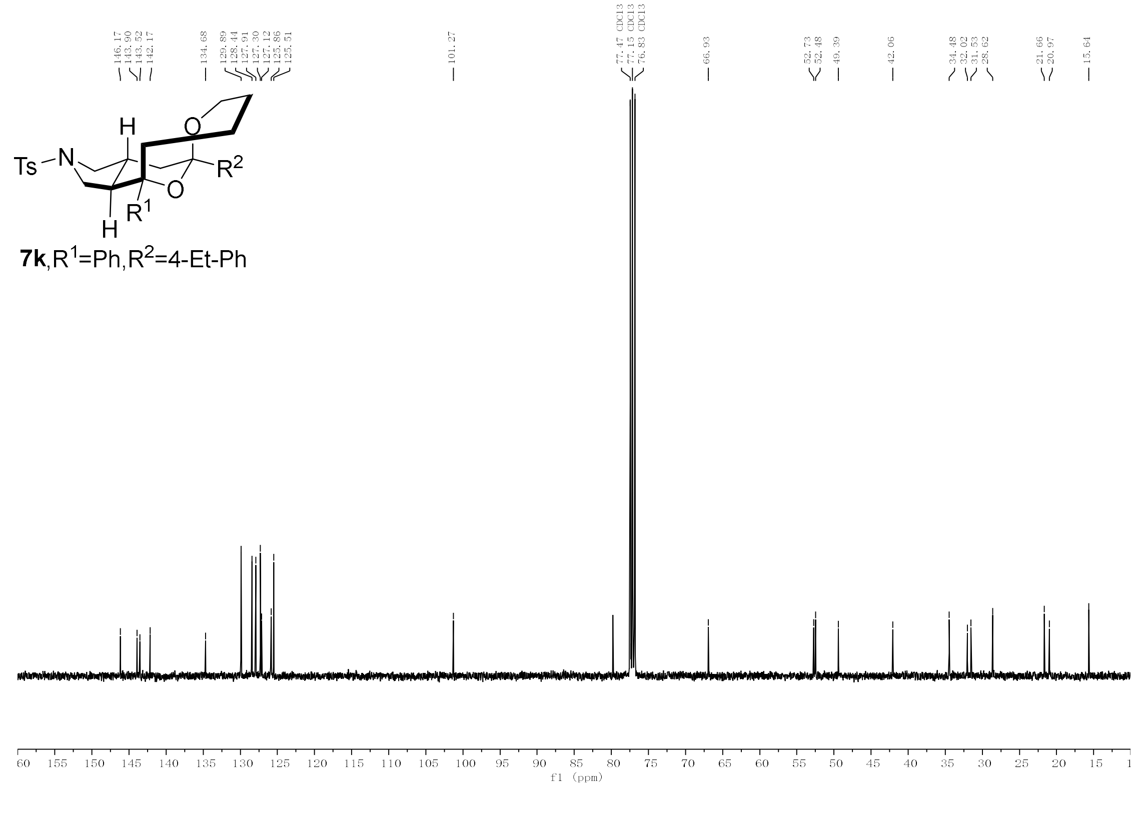

Supplementary Figure 129. ^{1}H NMR spectrum of 7l.

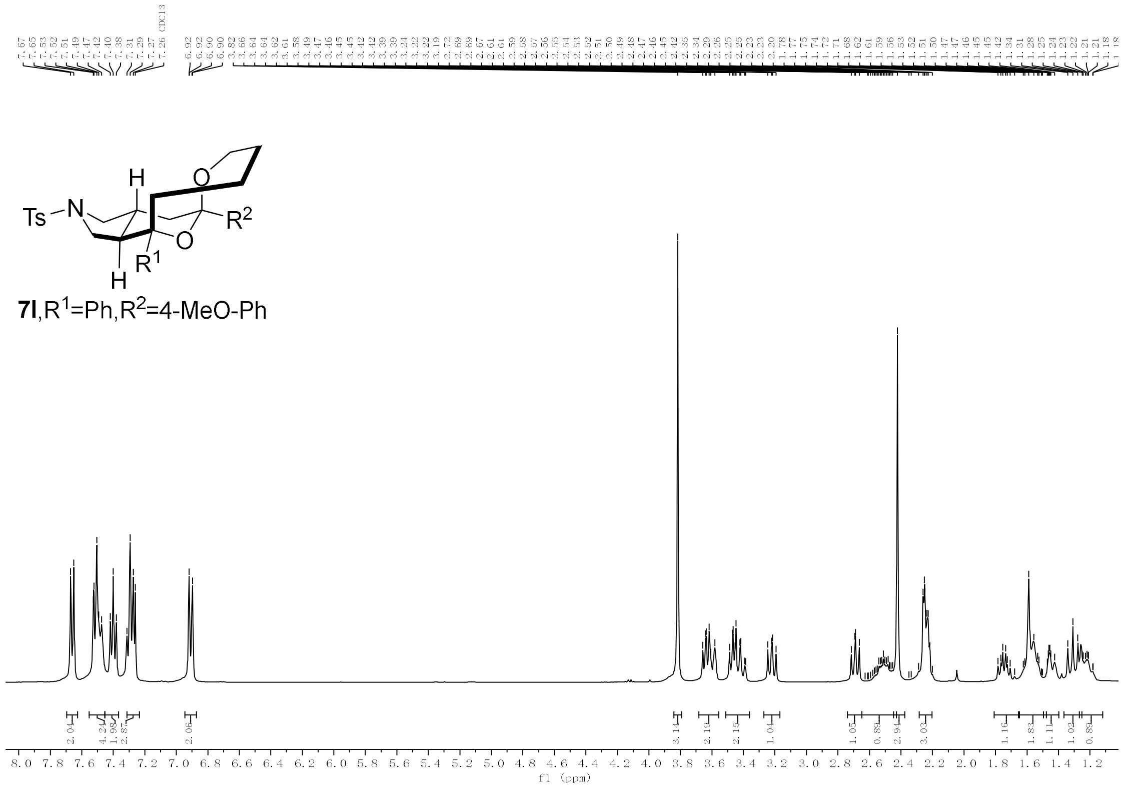

Supplementary Figure 130. ^{13}C NMR spectrum of 7l.

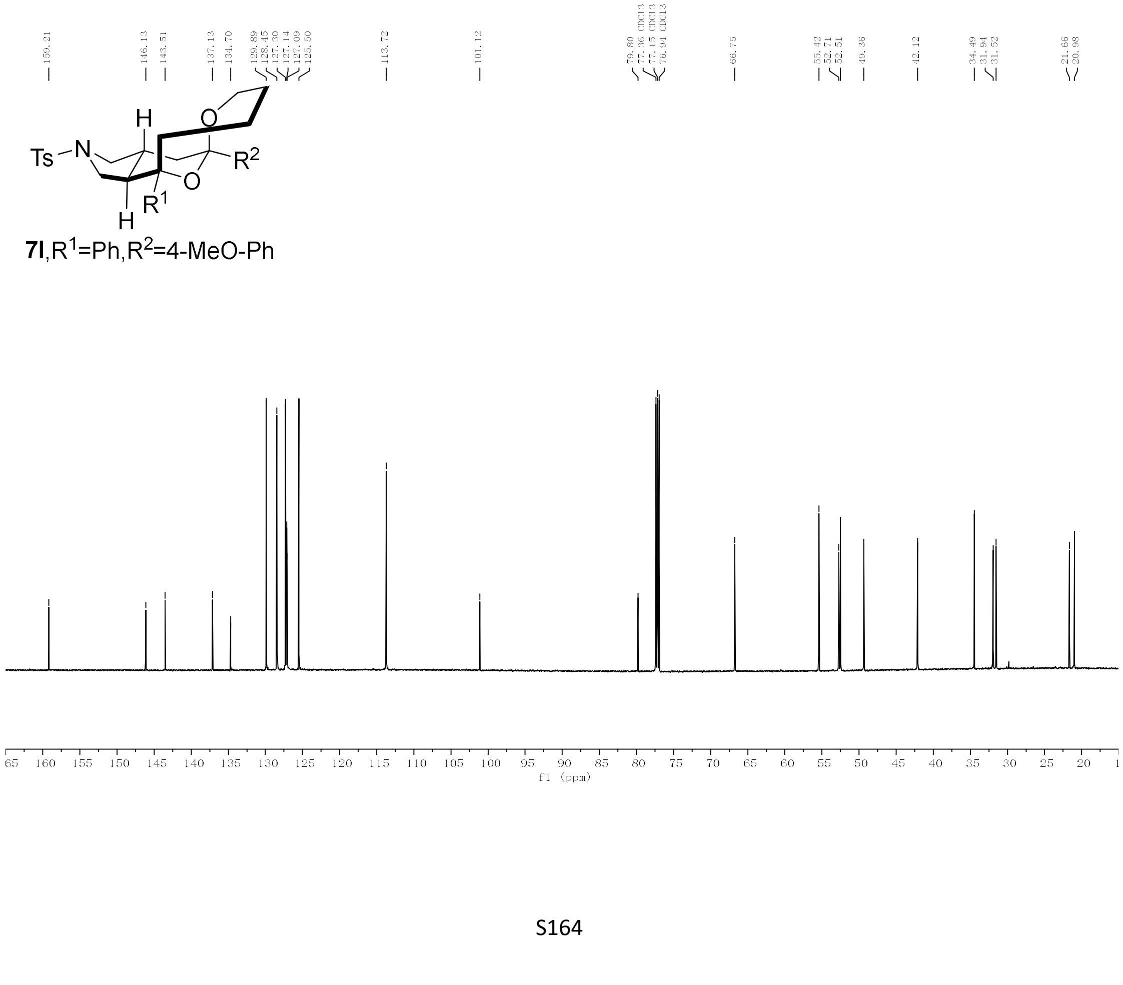

Supplementary Figure 131. ^{1}H NMR spectrum of 7m.

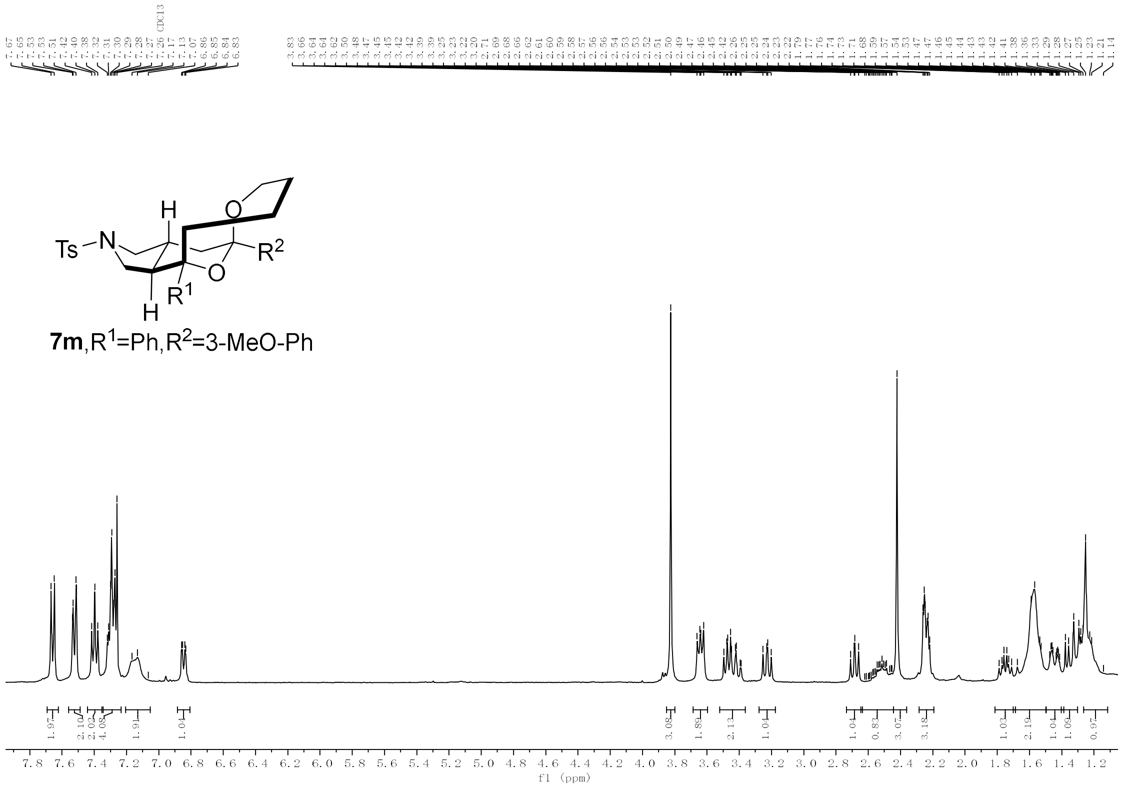

Supplementary Figure 132. ^{13}C NMR spectrum of 7m.

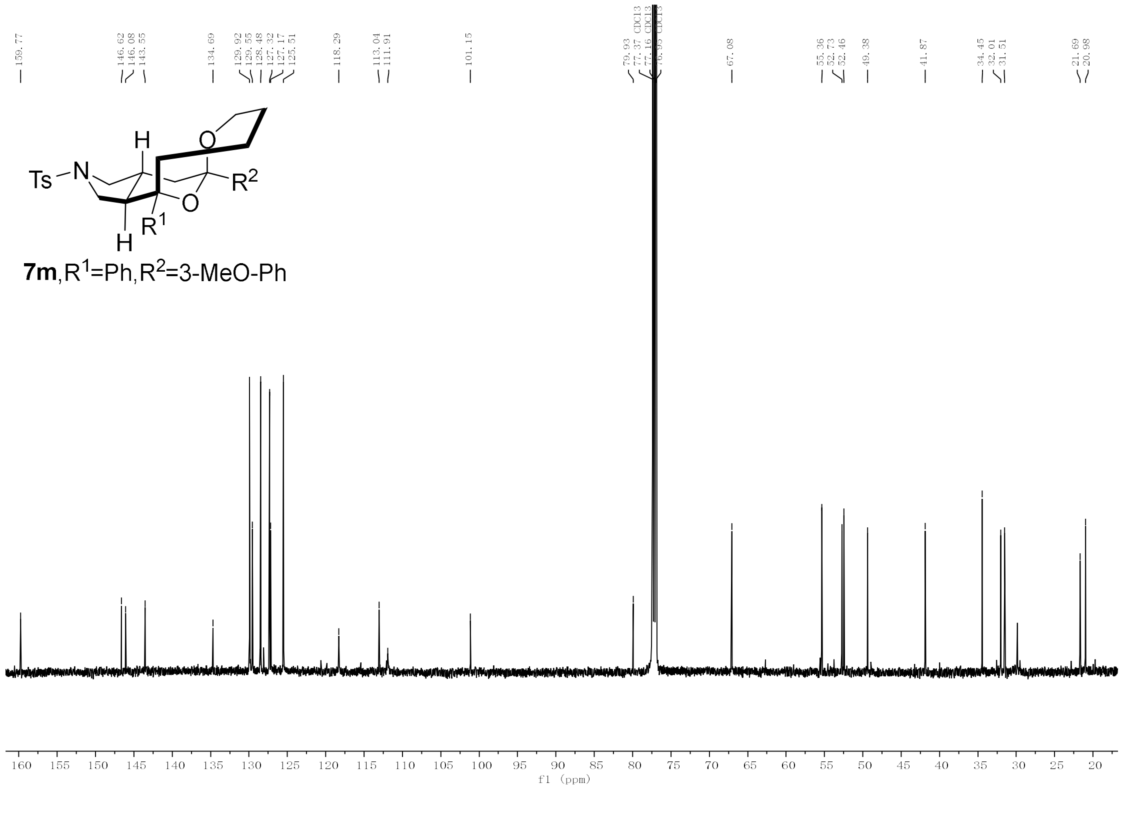

Supplementary Figure 133. ^{1}H NMR spectrum of 7n.

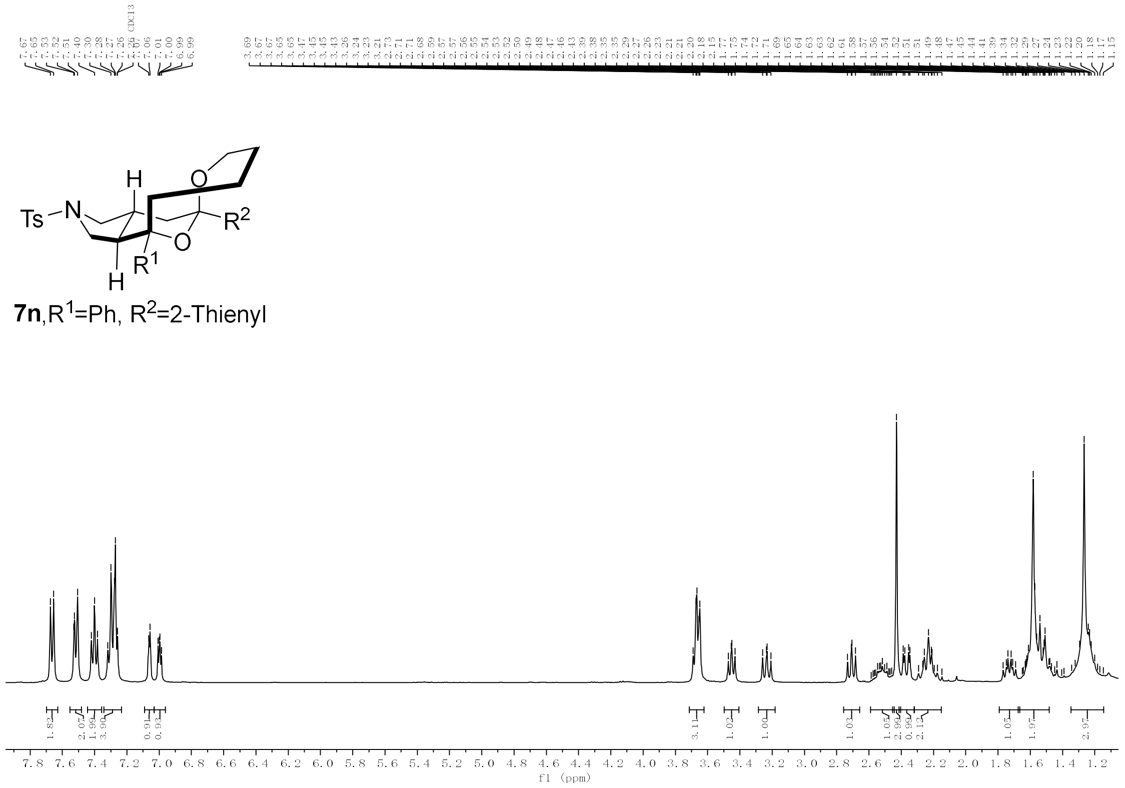

Supplementary Figure 134. ^{13}C NMR spectrum of 7n.

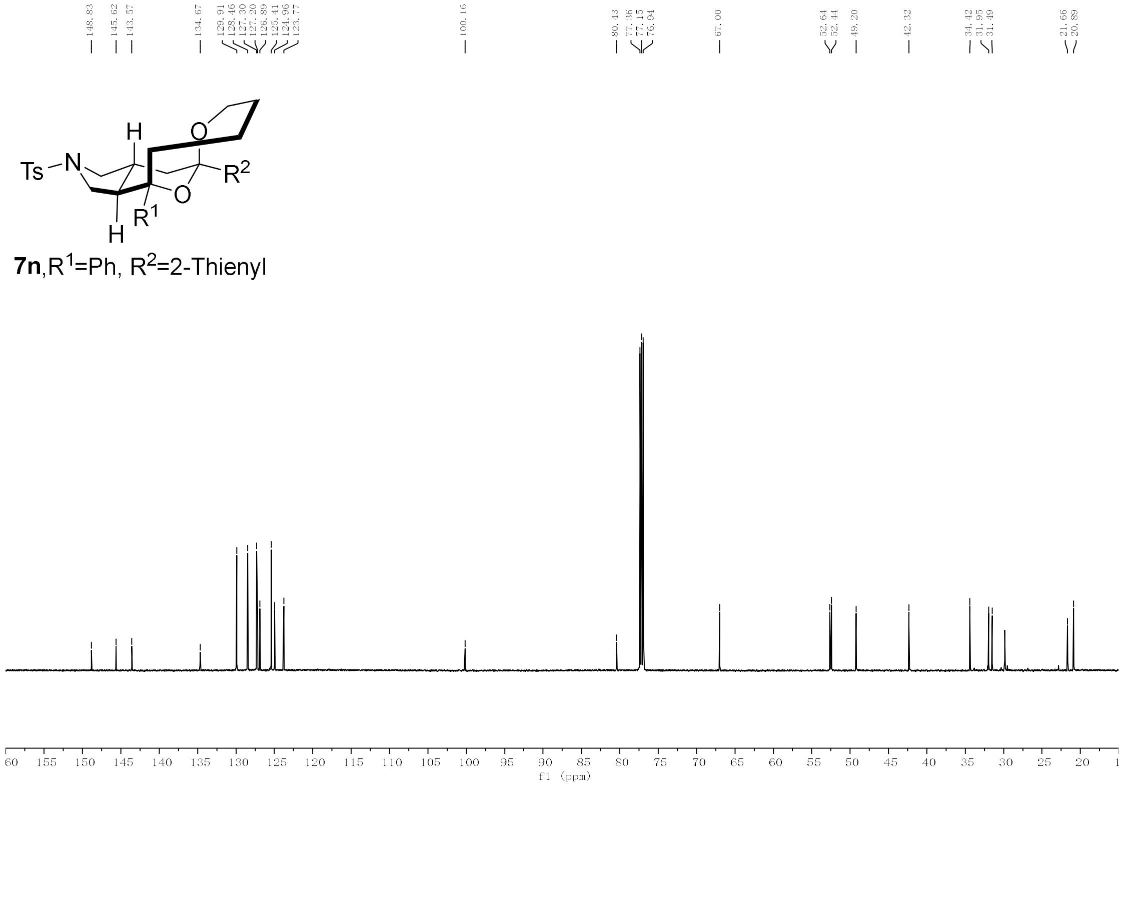

Supplementary Figure 135. ^{1}H NMR spectrum of 7o.

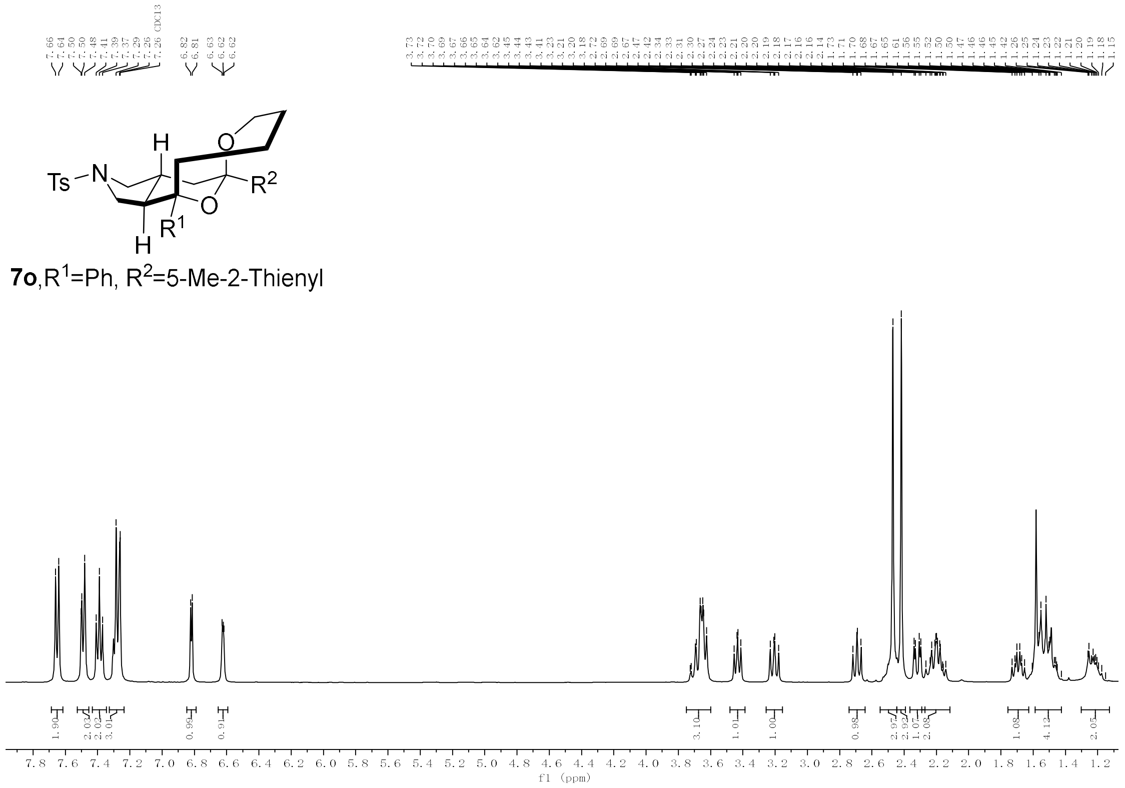

Supplementary Figure 136. ^{13}C NMR spectrum of 7o.

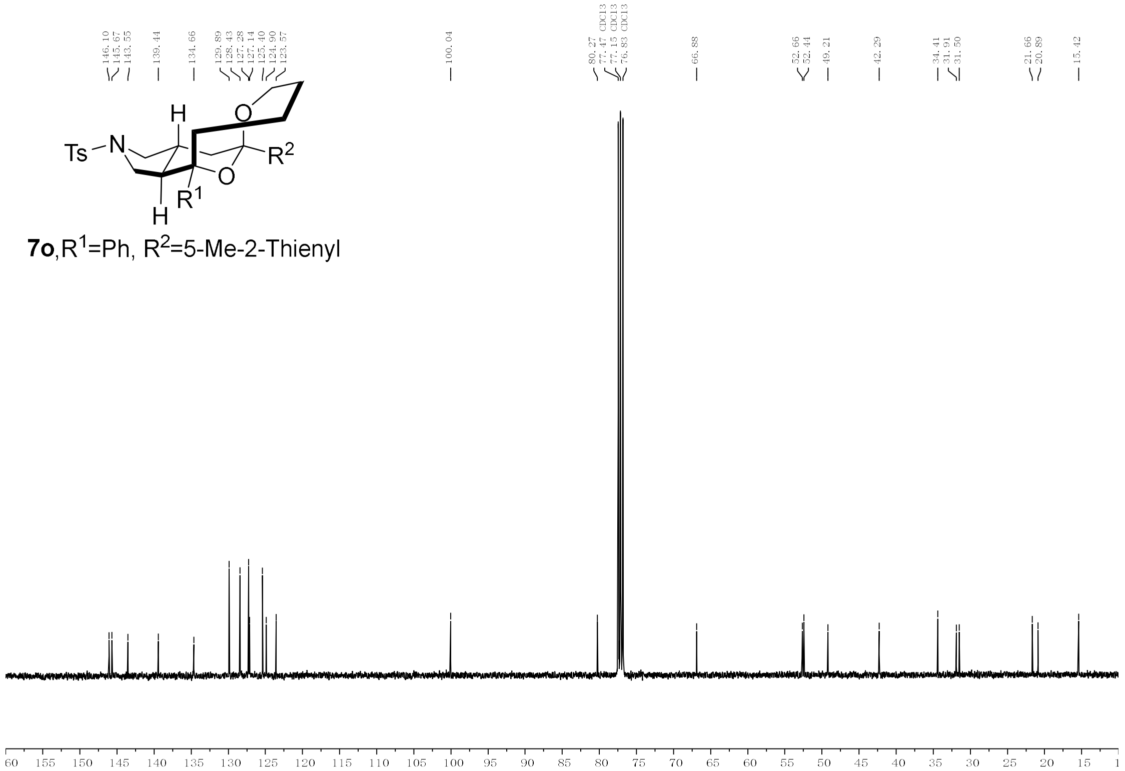

Supplementary Figure 137. ^{1}H NMR spectrum of 7p.

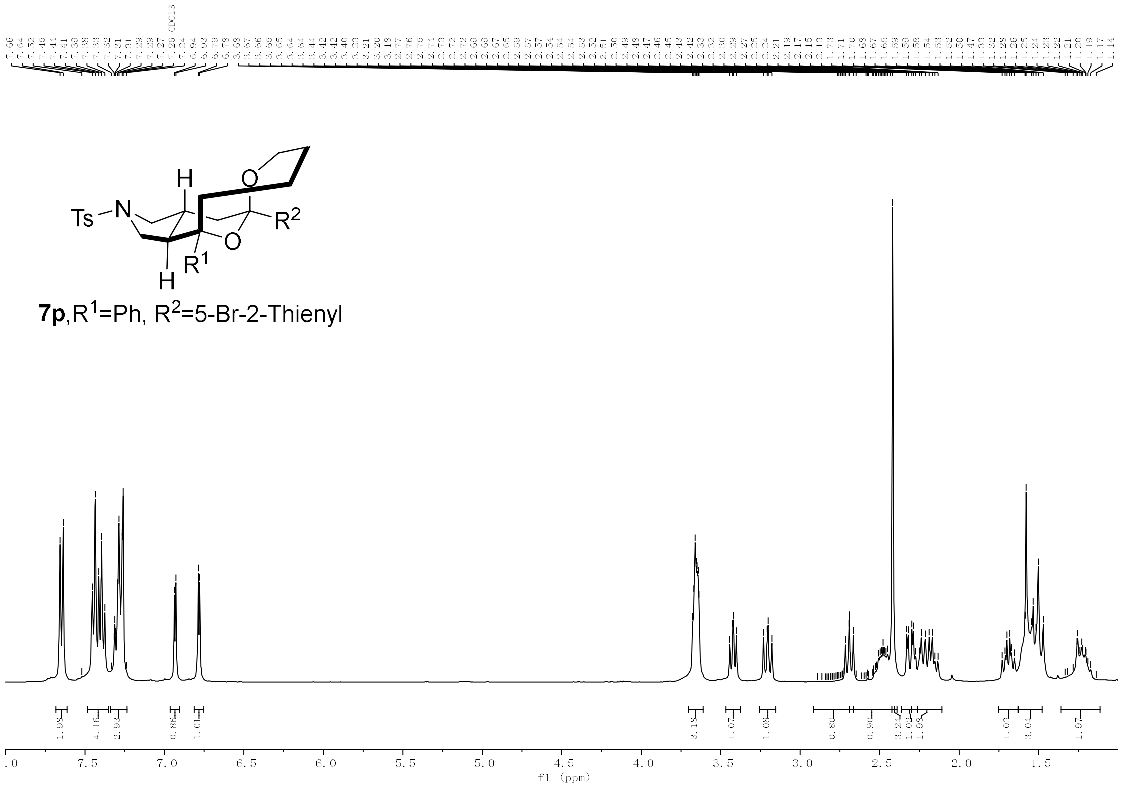

Supplementary Figure 138. ^{13}C NMR spectrum of 7p.

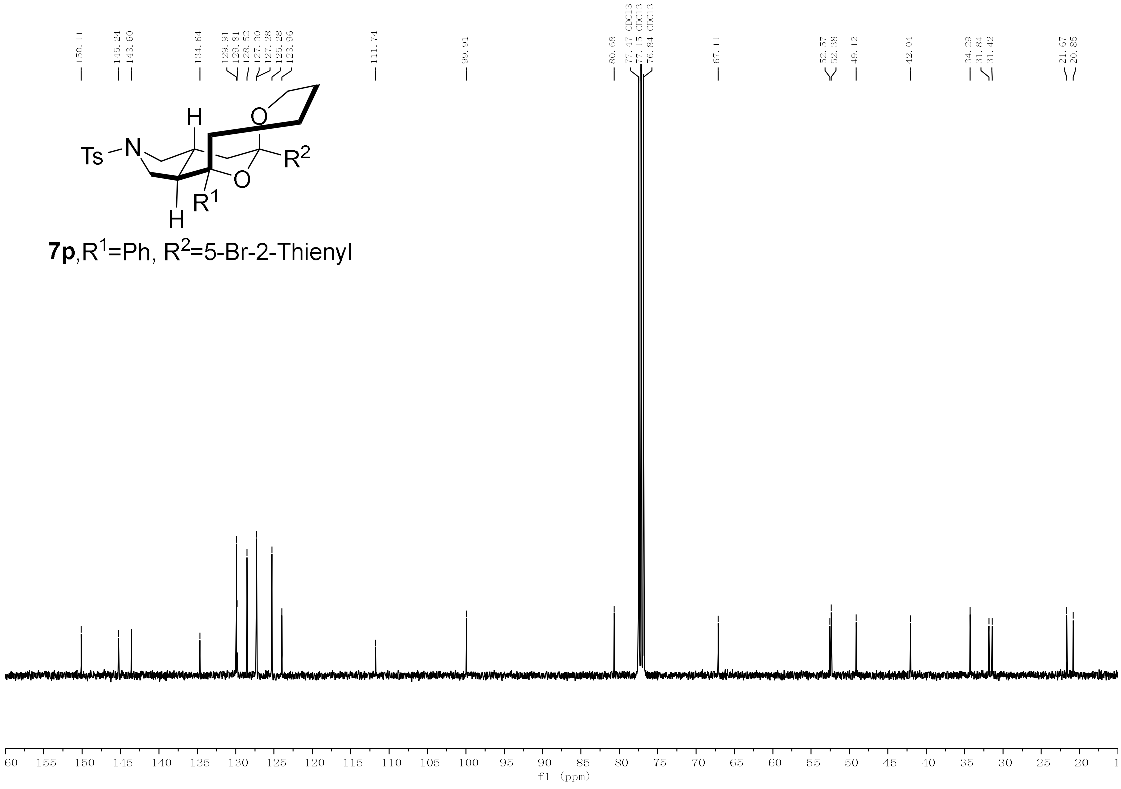

Supplementary Figure 139. ^{1}H NMR spectrum of 7q.

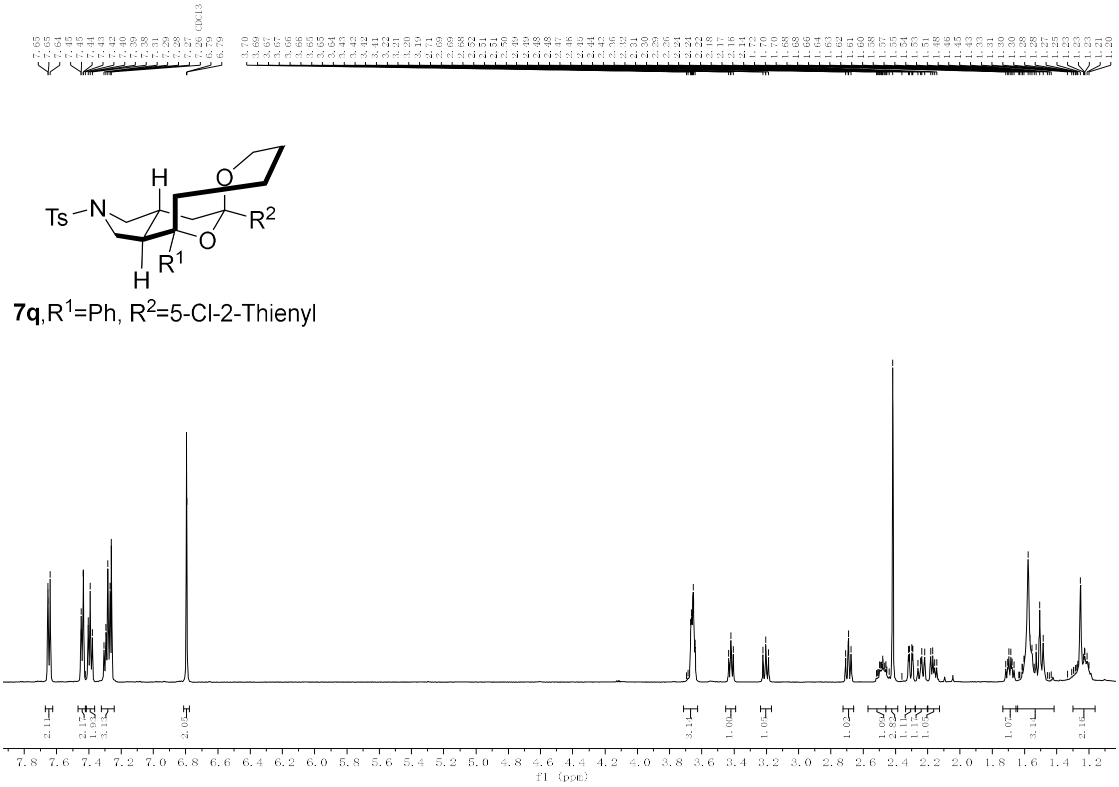

Supplementary Figure 140. ^{13}C NMR spectrum of 7q.

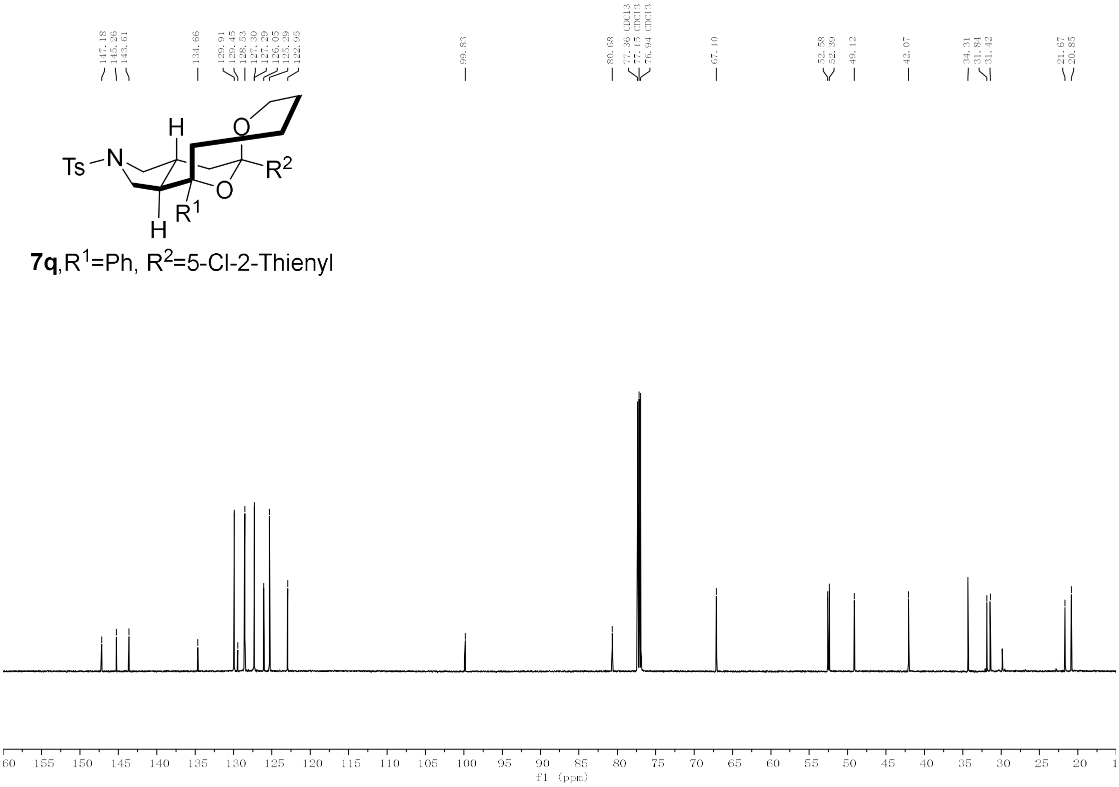

Supplementary Figure 141. ^{1}H NMR spectrum of 7r.

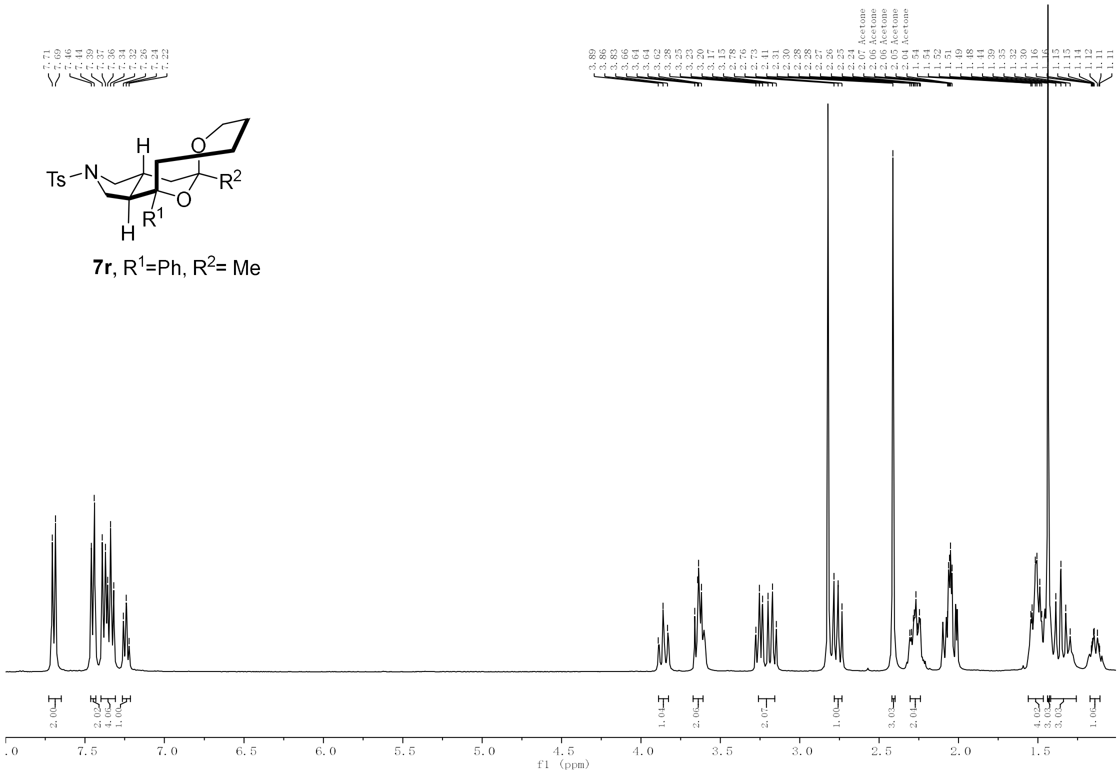

Supplementary Figure 142. ^{13}C NMR spectrum of 7r.

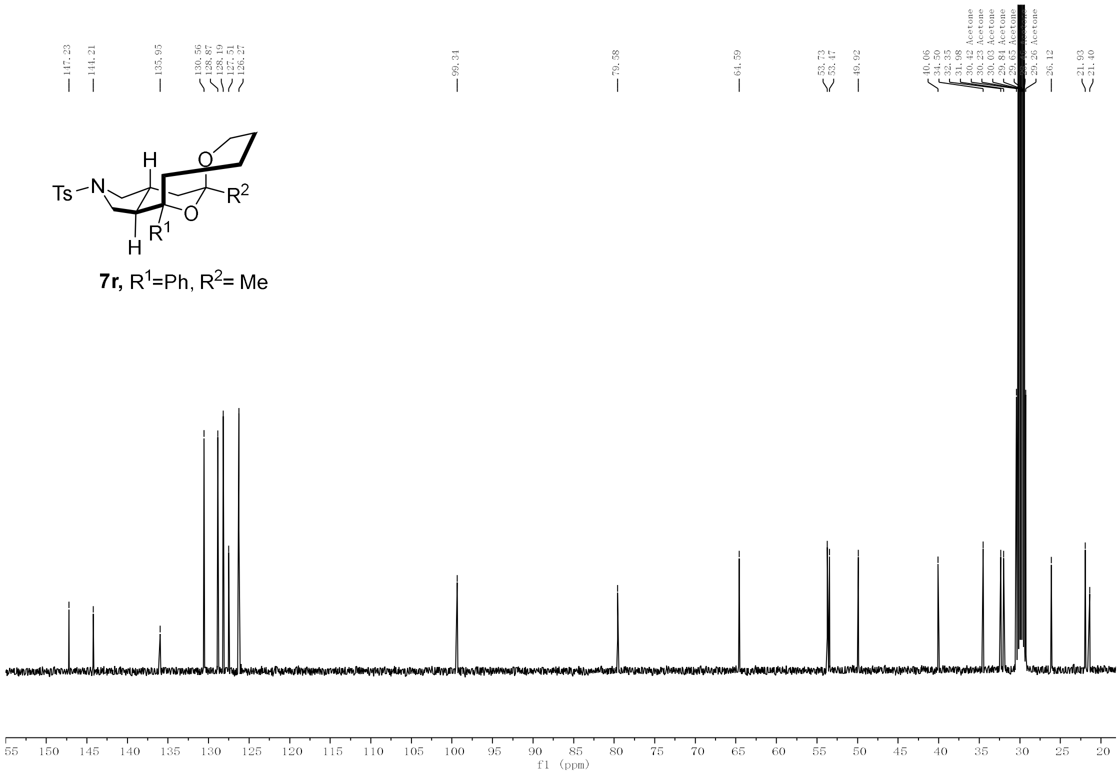

Supplementary Figure 143. ^{1}H NMR spectrum of 7s.

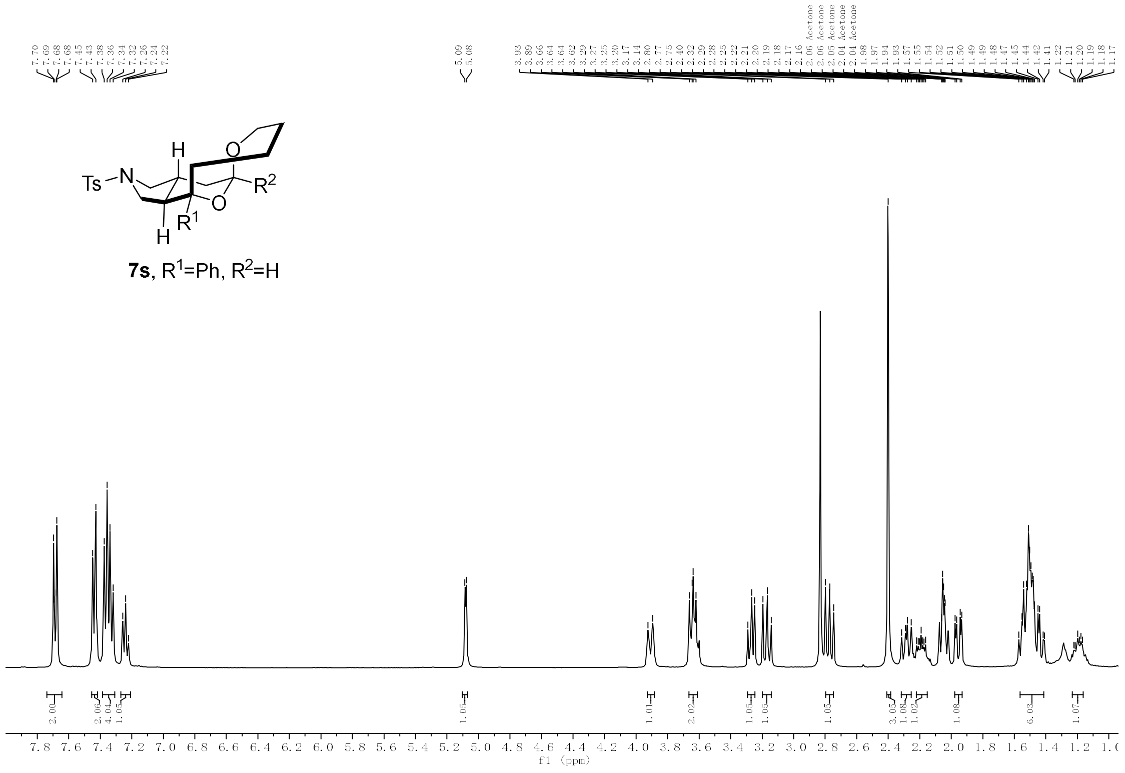

Supplementary Figure 144. ^{13}C NMR spectrum of 7s.

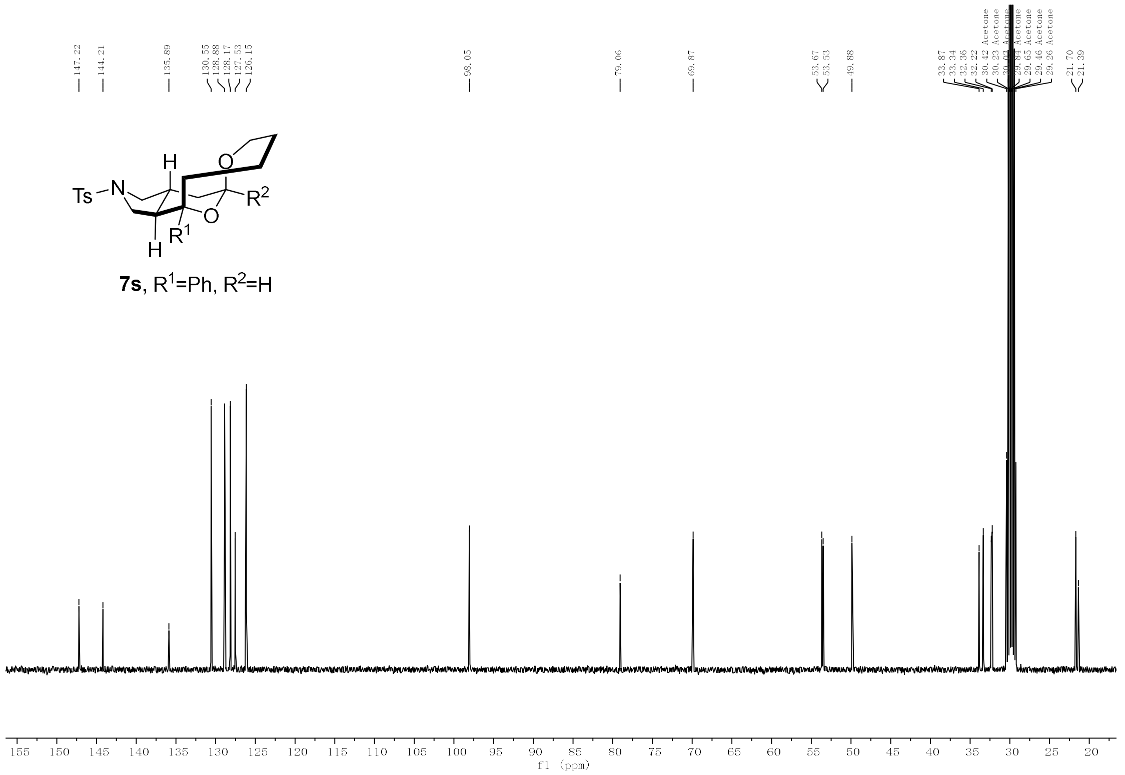

Supplementary Figure 145: [1] H NMR spectrum of 7t.

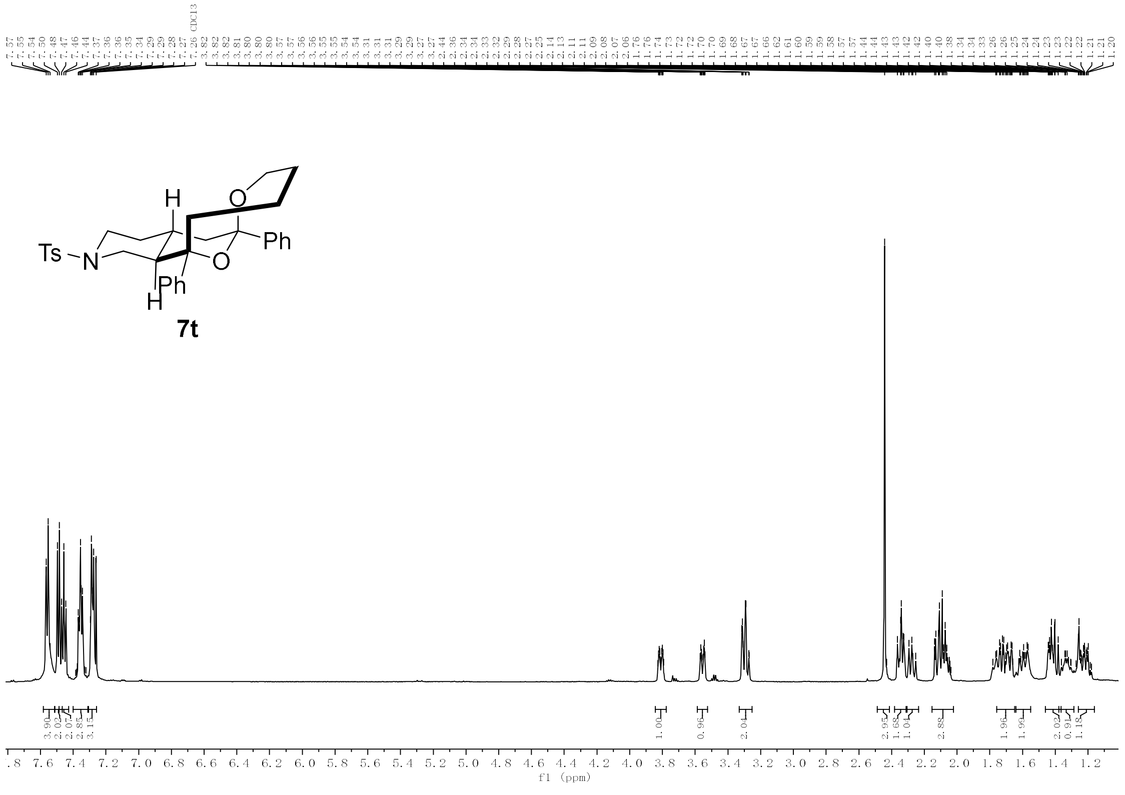

Supplementary Figure 146: [13] C NMR spectrum of 7t.

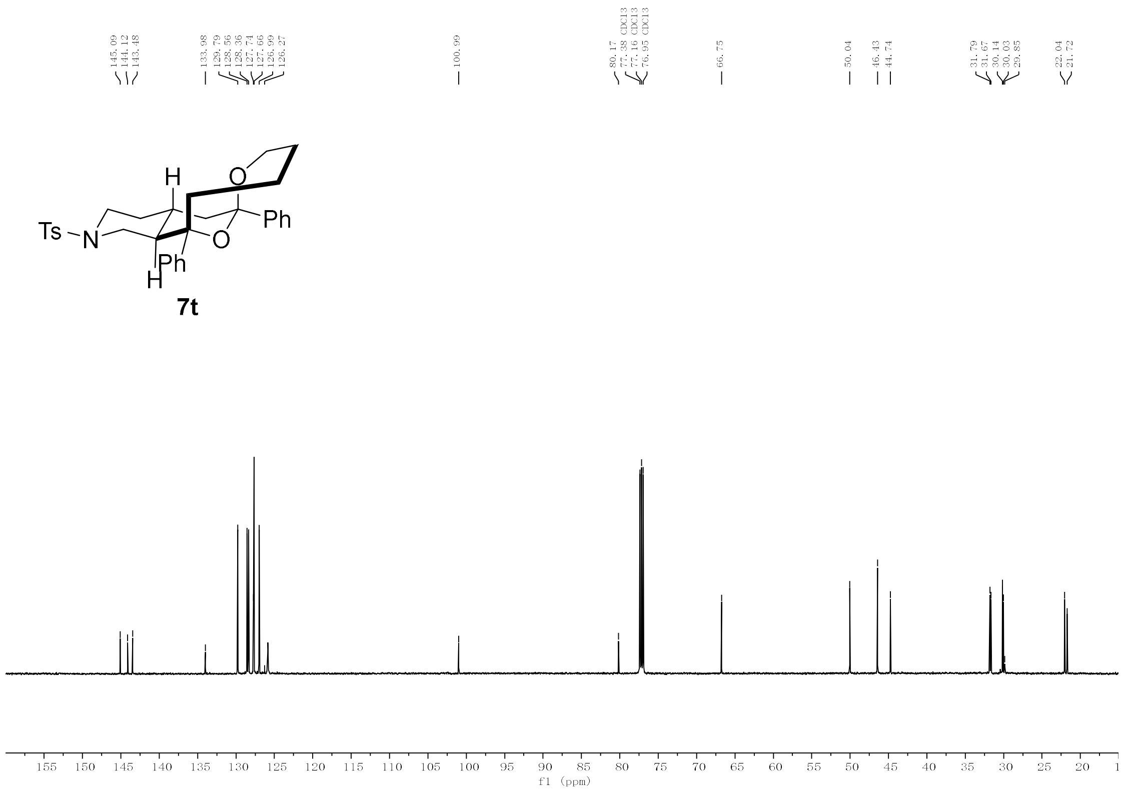

Supplementary Figure 147. ^{1}H NMR spectrum of 8a.

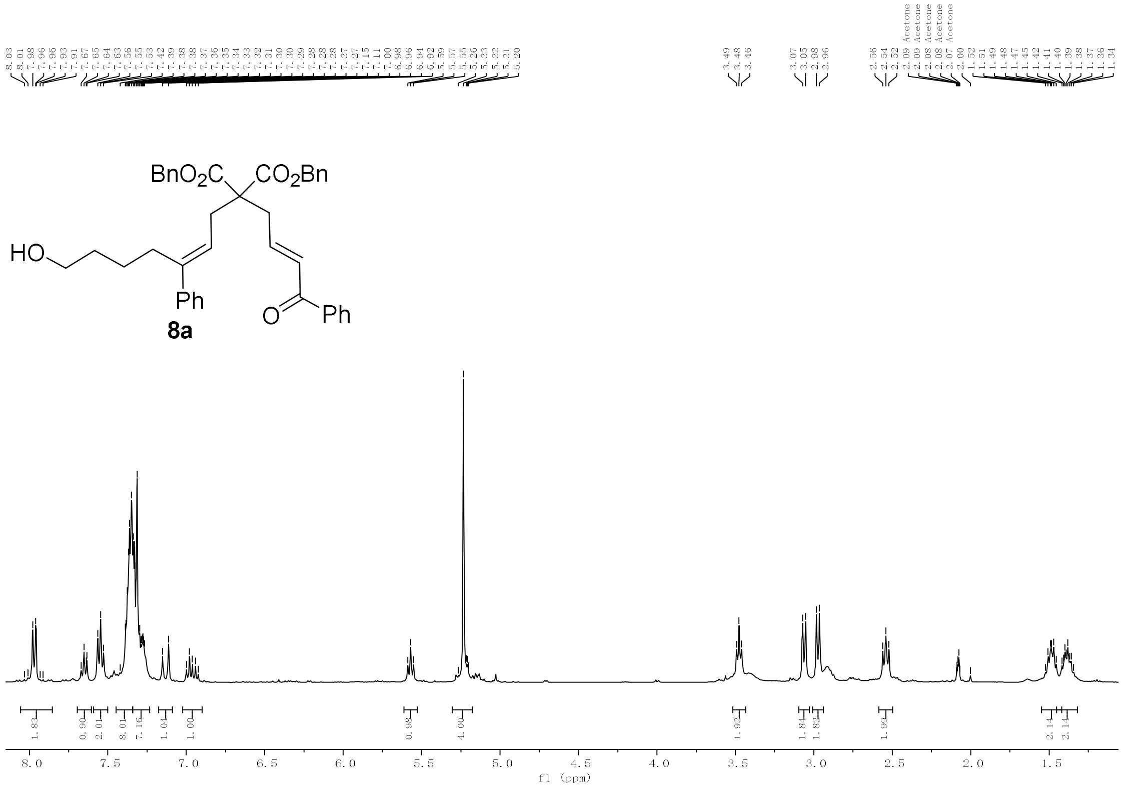

Supplementary Figure 148. ^{13}C NMR spectrum of 8a.

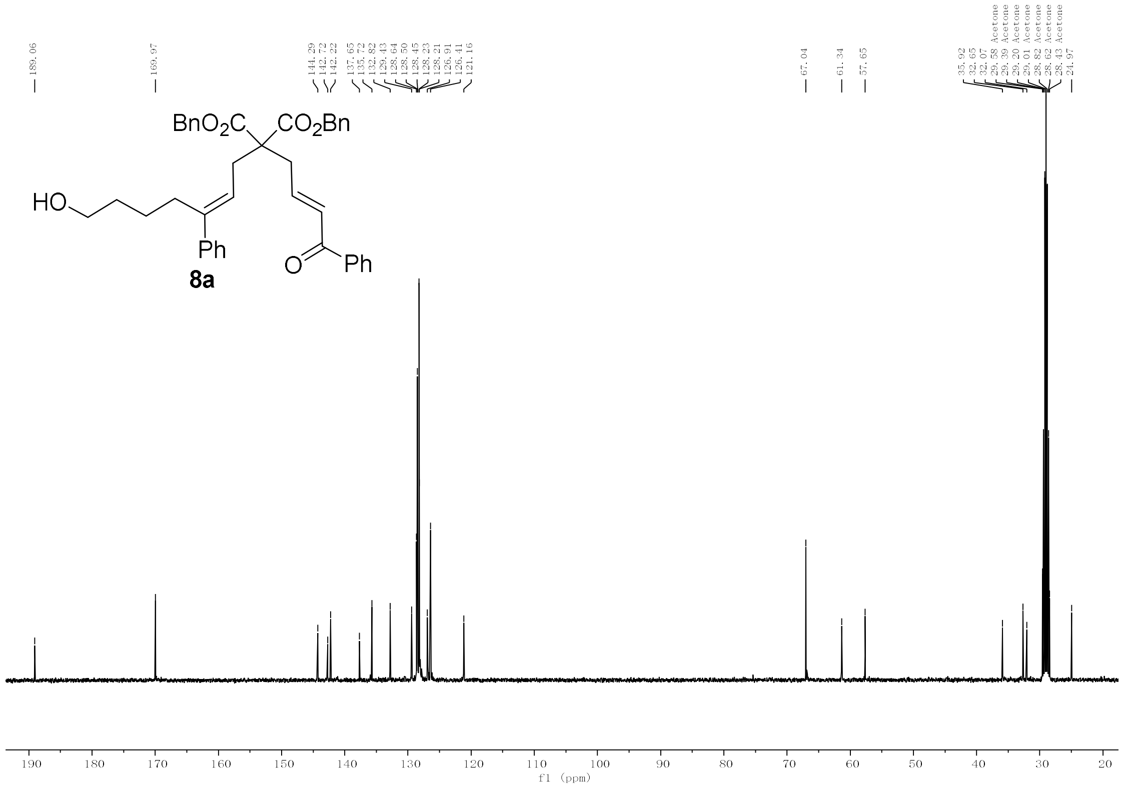

Supplementary Figure 149. ^{1}H NMR spectrum of 8b.

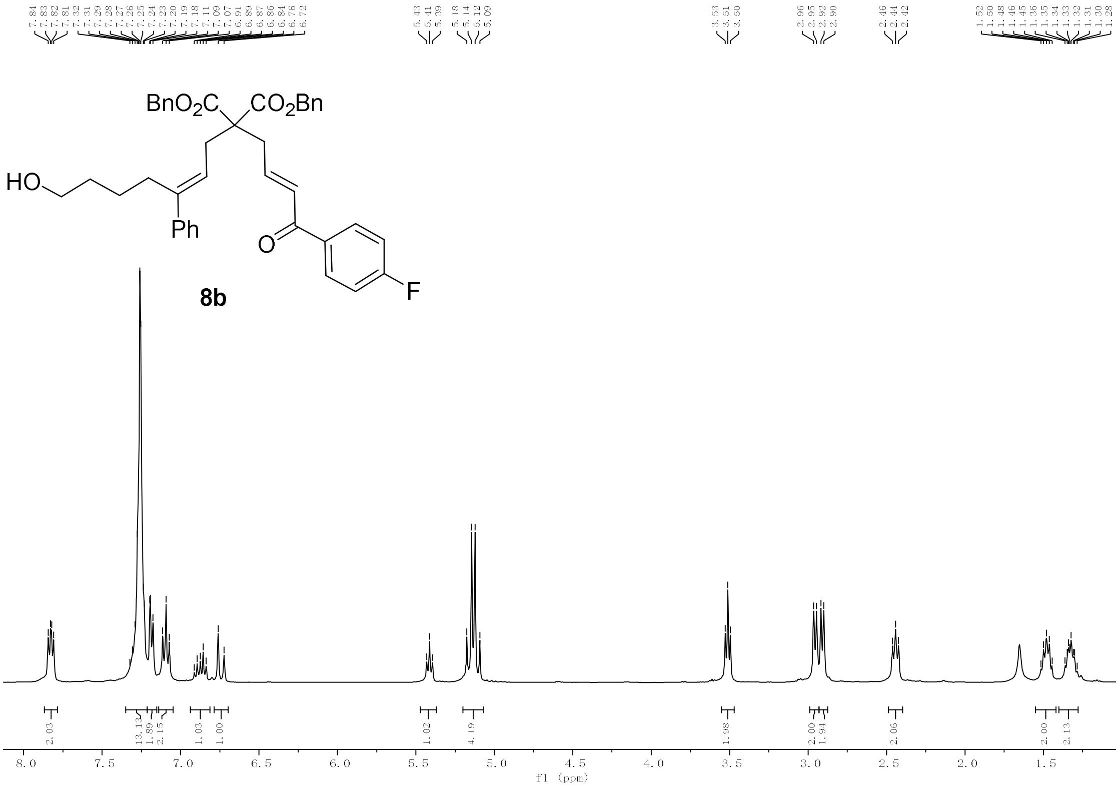

Supplementary Figure 150. ^{13}C NMR spectrum of 8b.

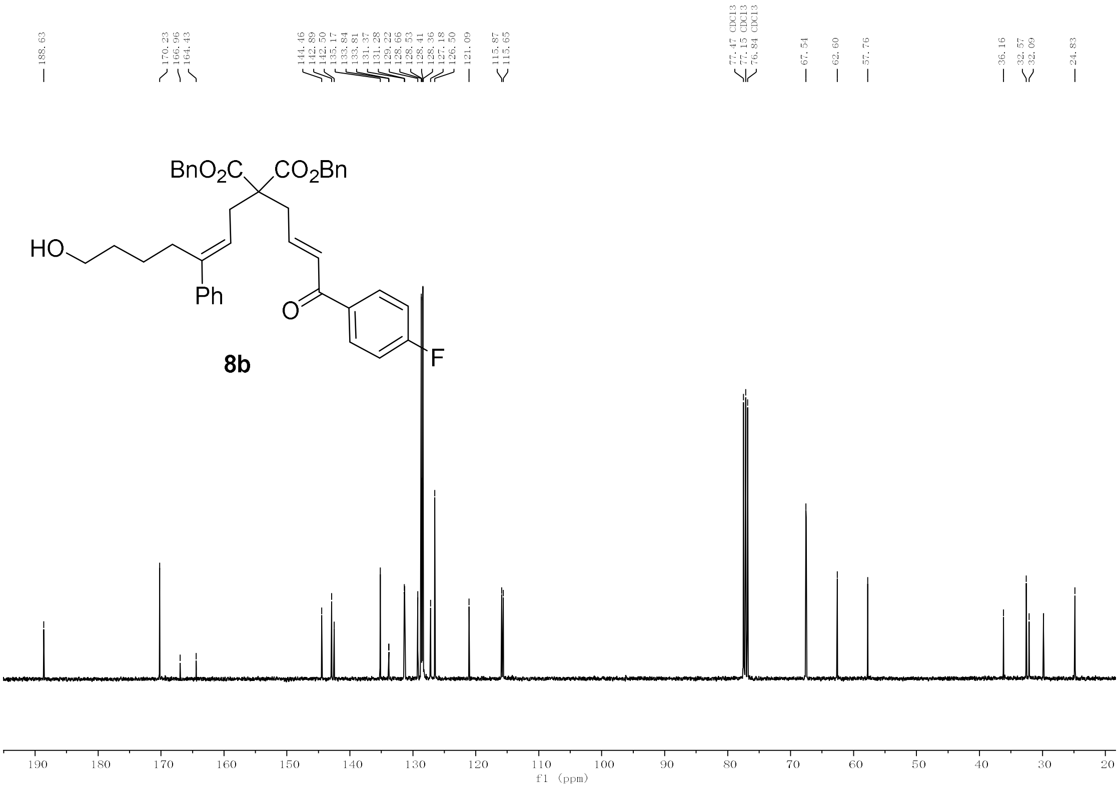

Supplementary Figure 151. ¹ H NMR spectrum of 8c.

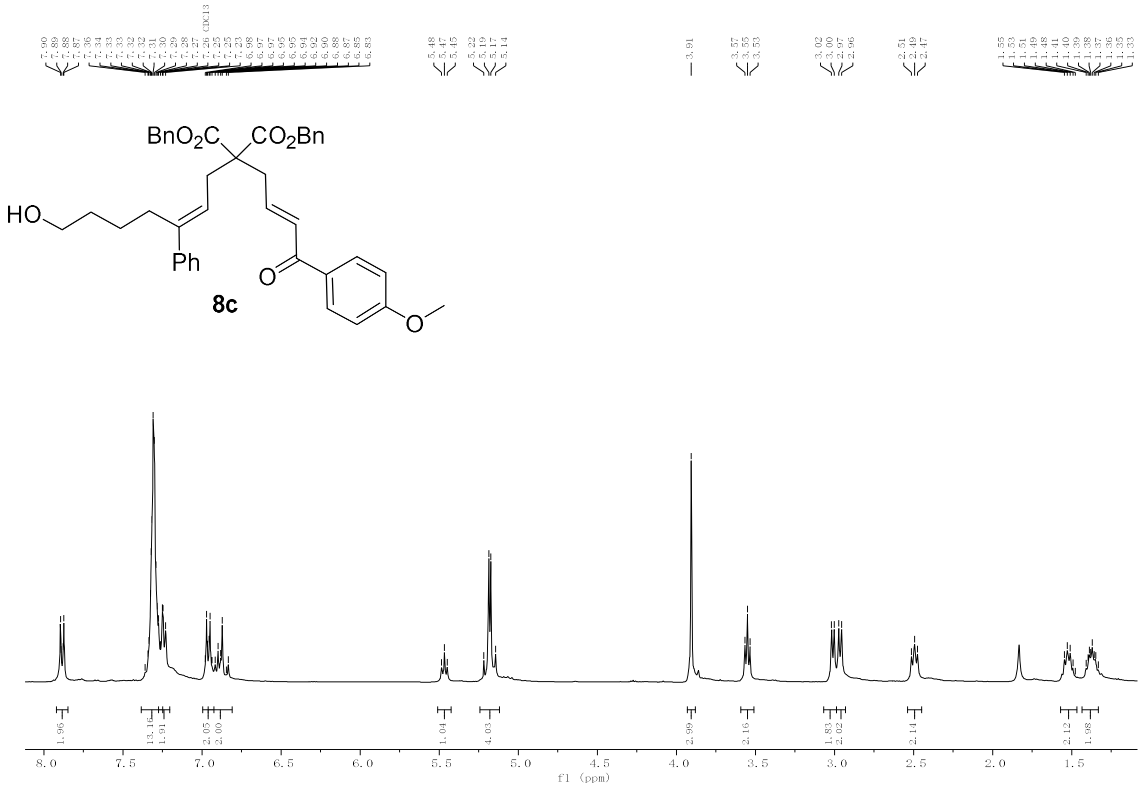

Supplementary Figure 152. ¹³ C NMR spectrum of 8c.

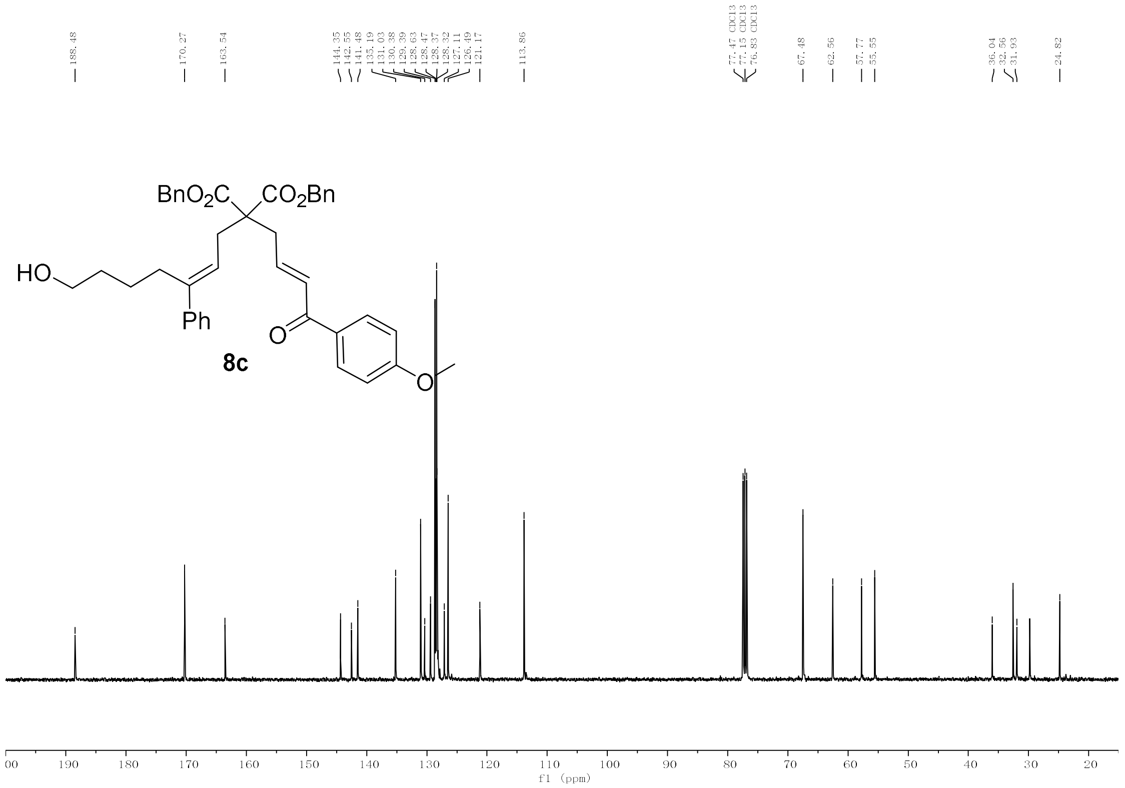

Supplementary Figure 153. ^{1}H NMR spectrum of 8d.

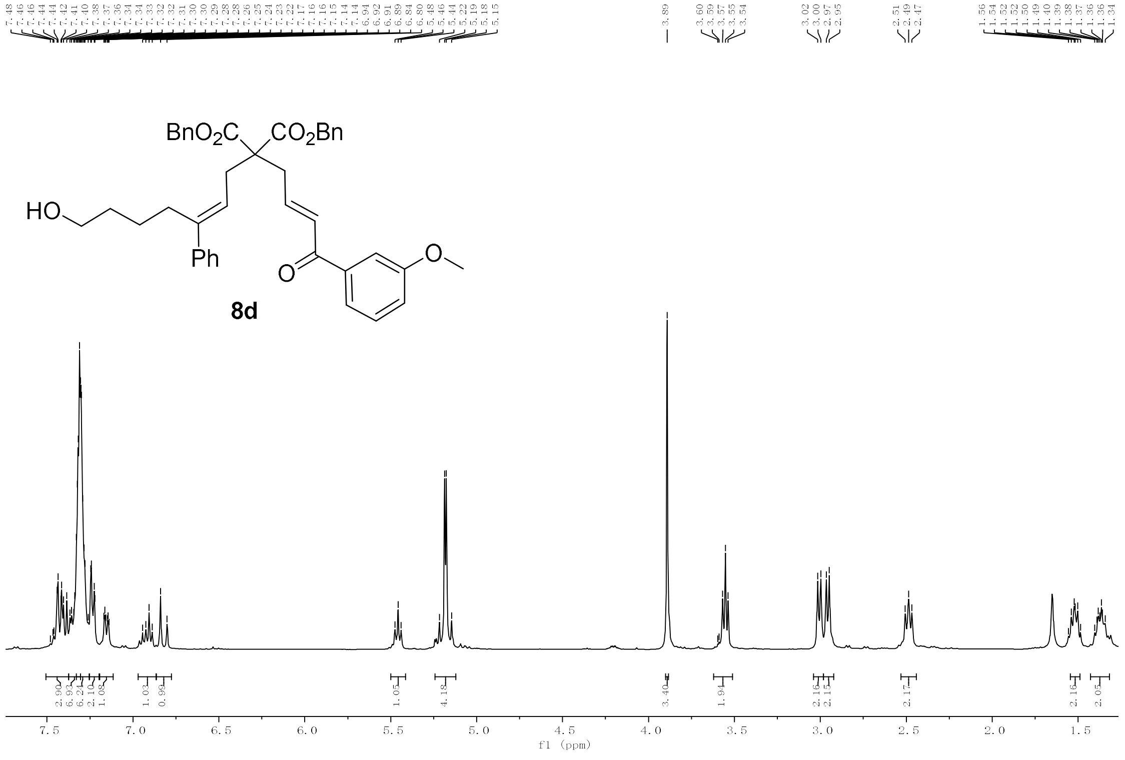

Supplementary Figure 154. ^{13}C NMR spectrum of 8d.

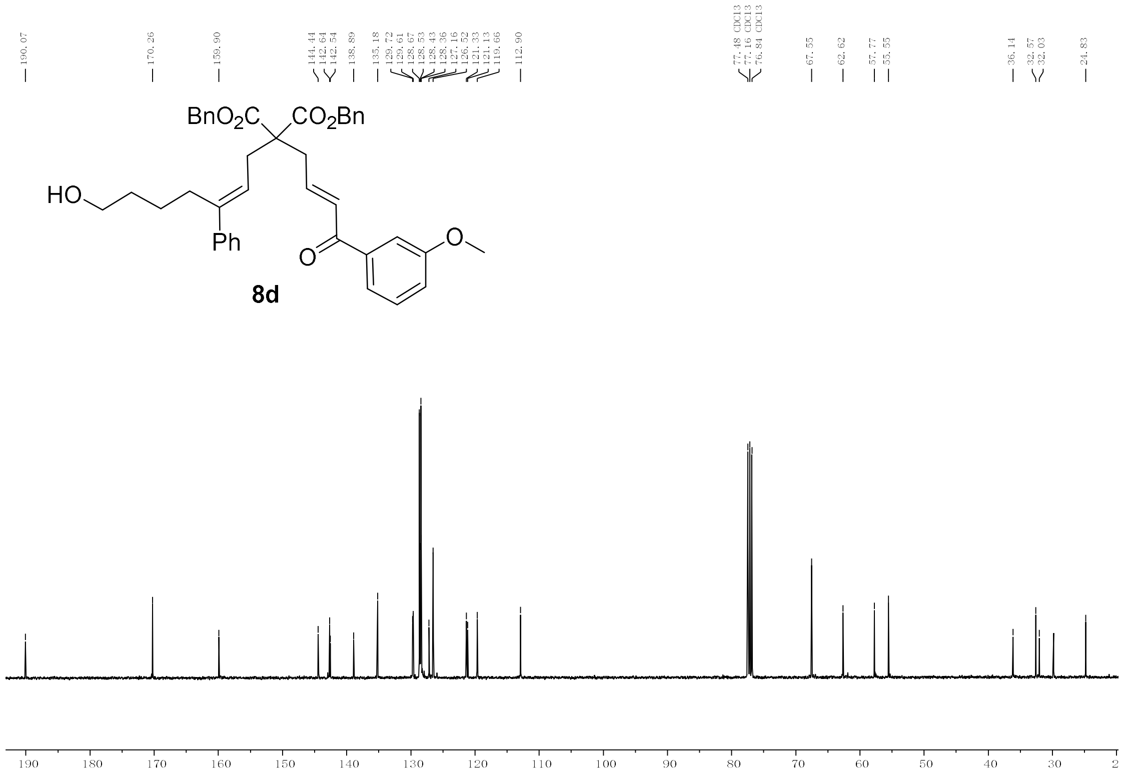

Supplementary Figure 155. ^{1}H NMR spectrum of 8e.

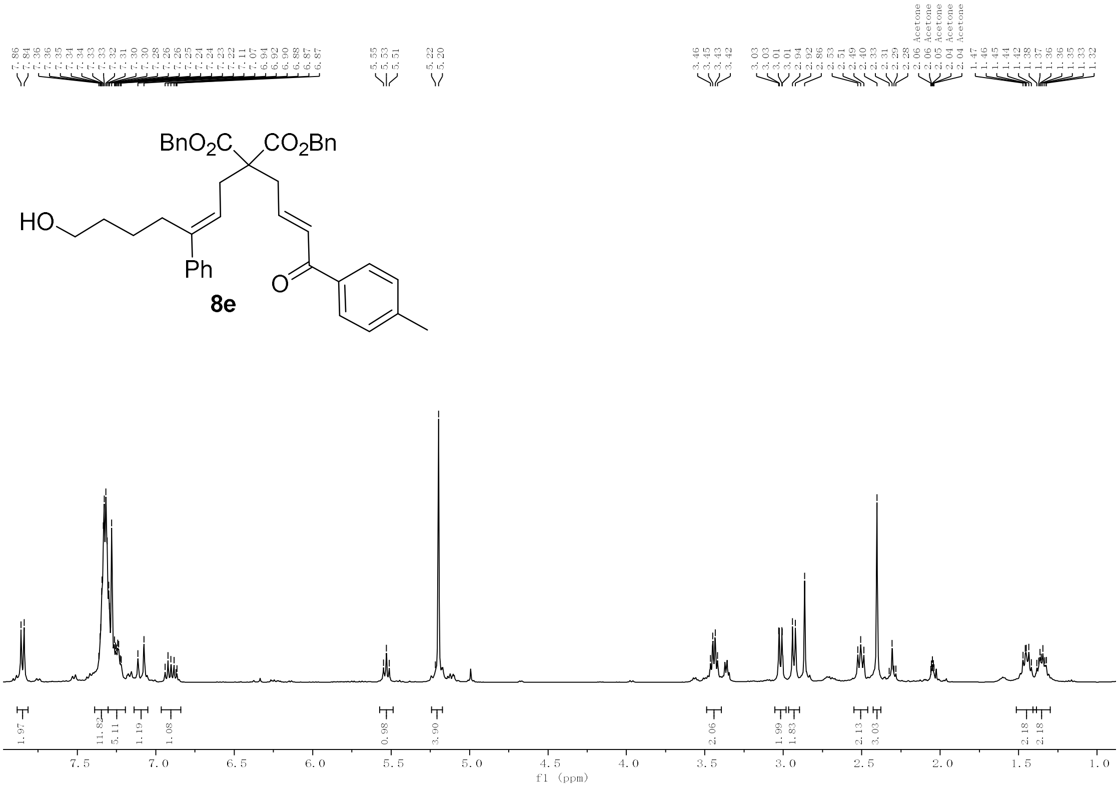

Supplementary Figure 156. ^{13}C NMR spectrum of 8e.

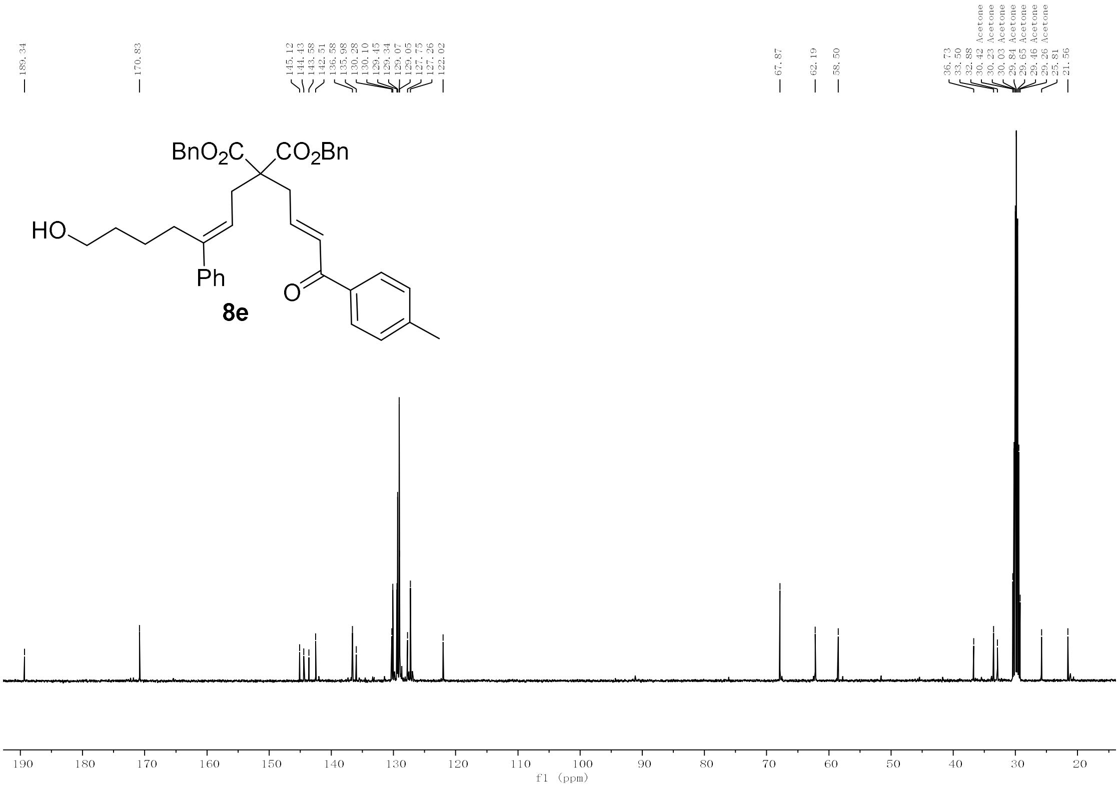

Supplementary Figure 157. [1] H NMR spectrum of 8f.

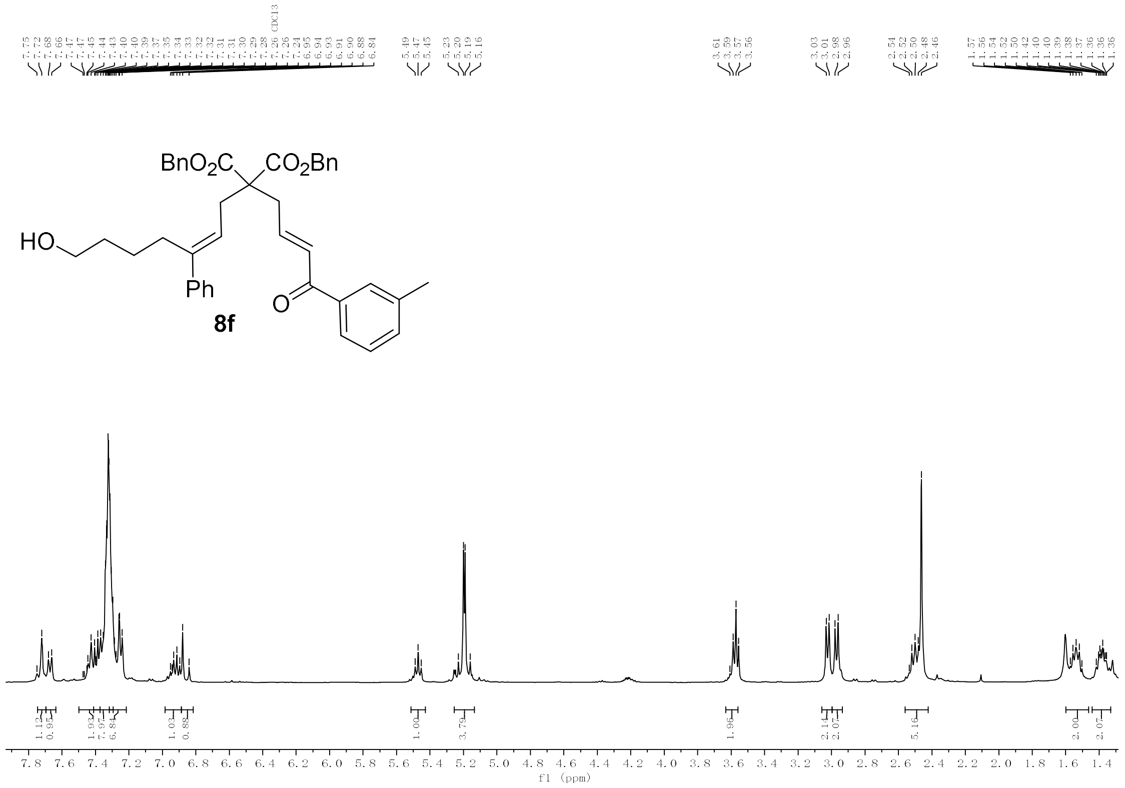

Supplementary Figure 158. [13] C NMR spectrum of 8f.

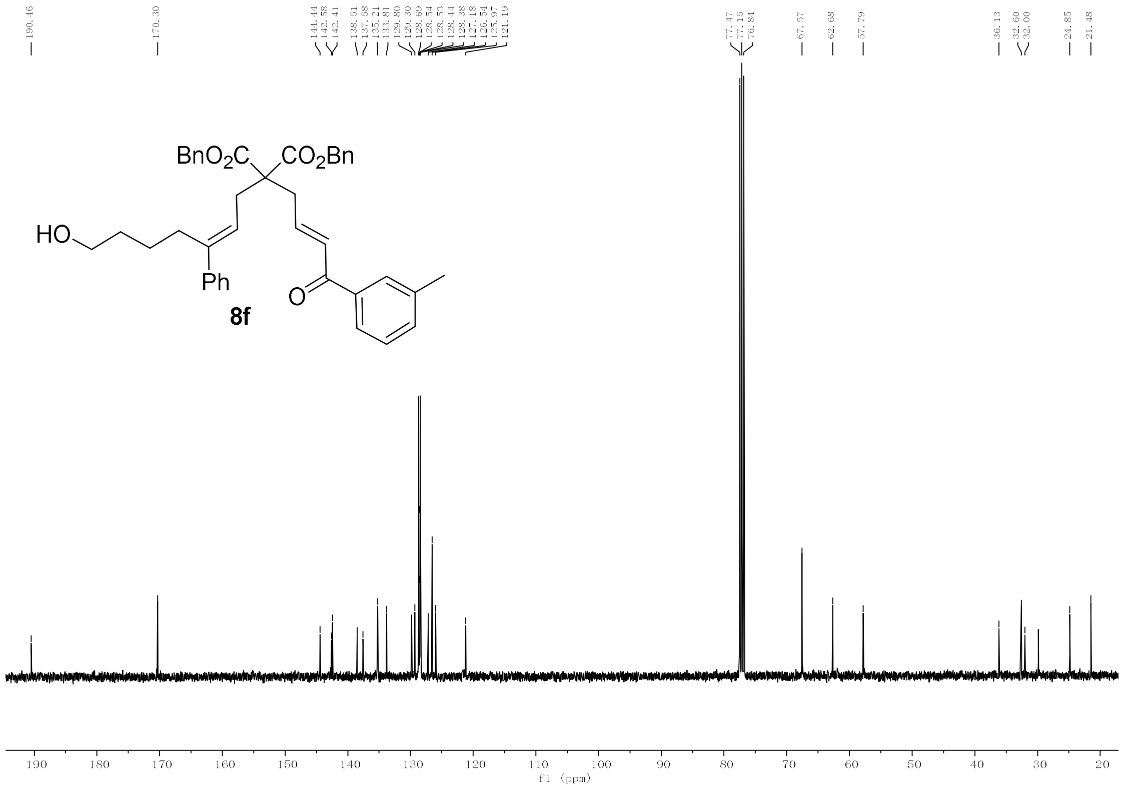

Supplementary Figure 159. [1] H NMR spectrum of 8g.

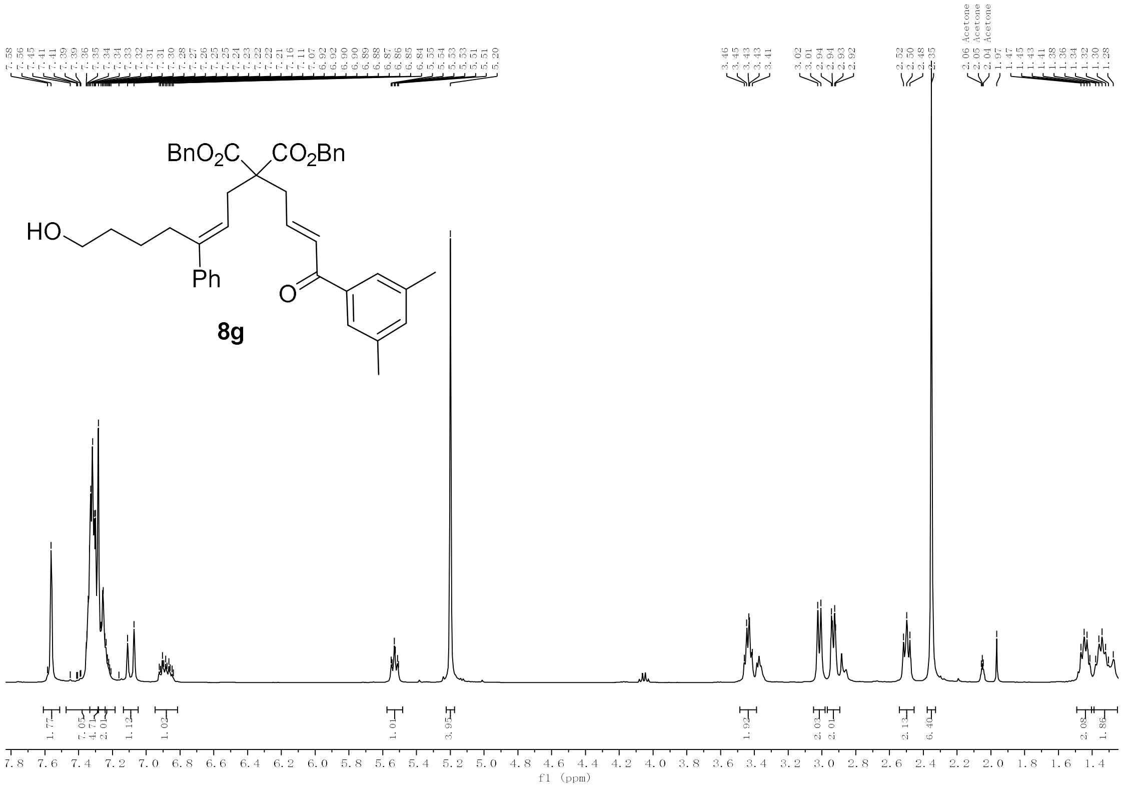

Supplementary Figure 160. [13] C NMR spectrum of 8g.

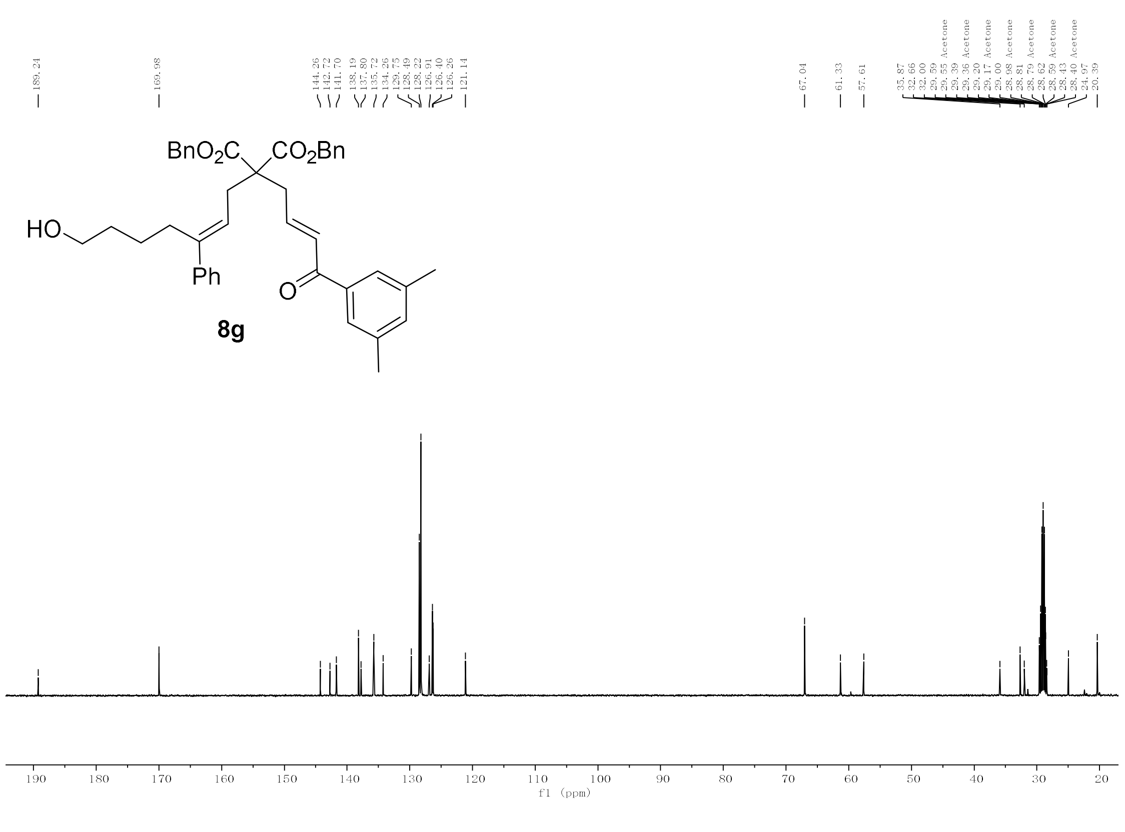

Supplementary Figure 161. [1] H NMR spectrum of **8h**.

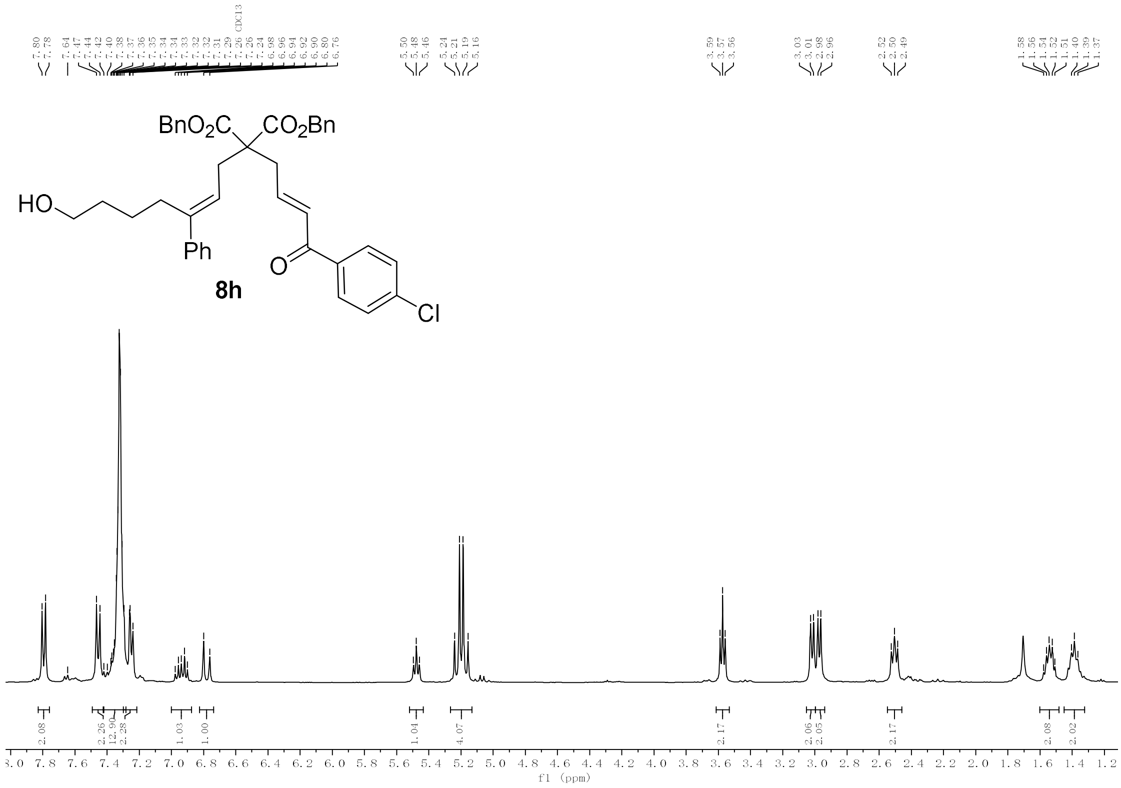

Supplementary Figure 162. [13] C NMR spectrum of **8h**.

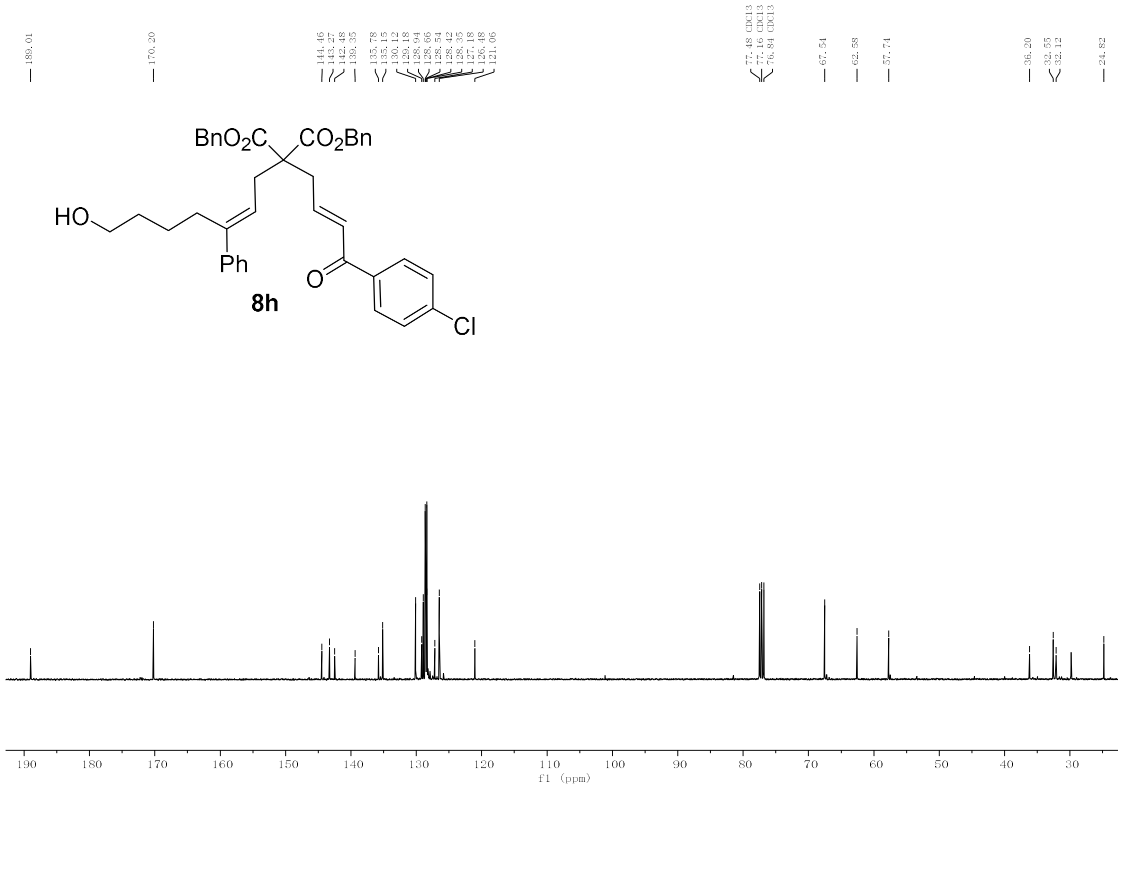

Supplementary Figure 163. ^{1}H NMR spectrum of 8i.

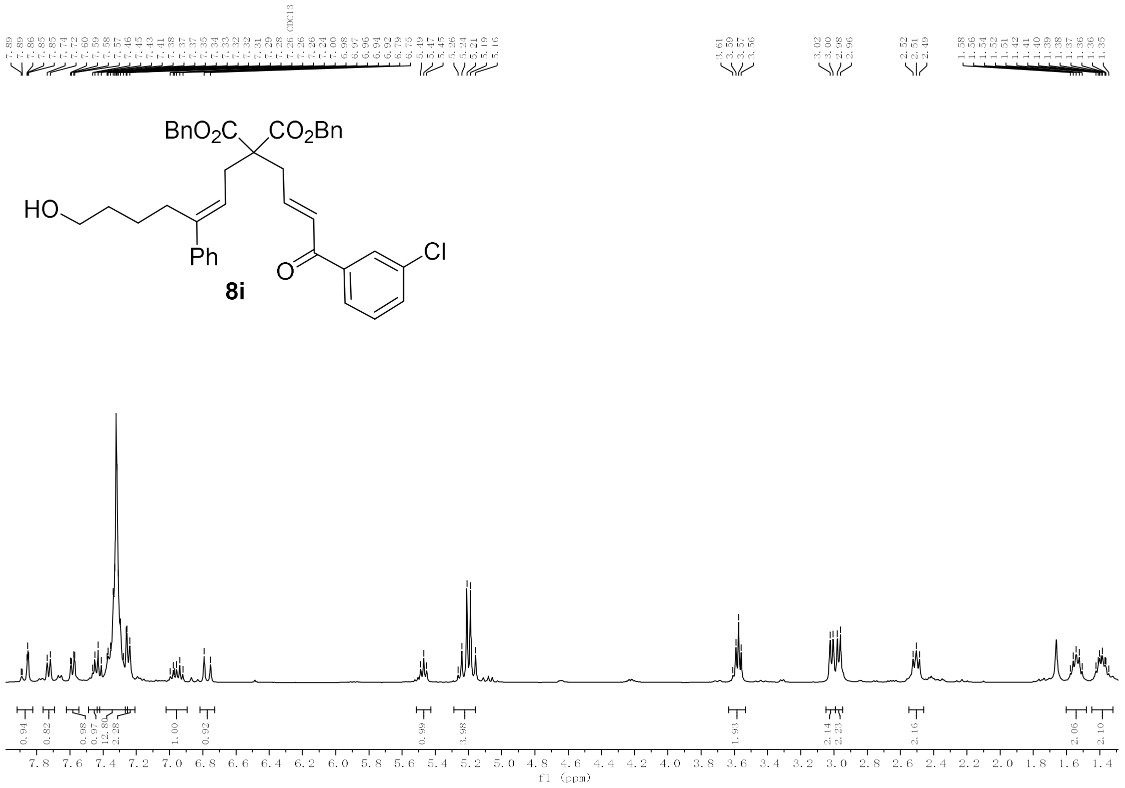

Supplementary Figure 164. ^{13}C NMR spectrum of 8i.

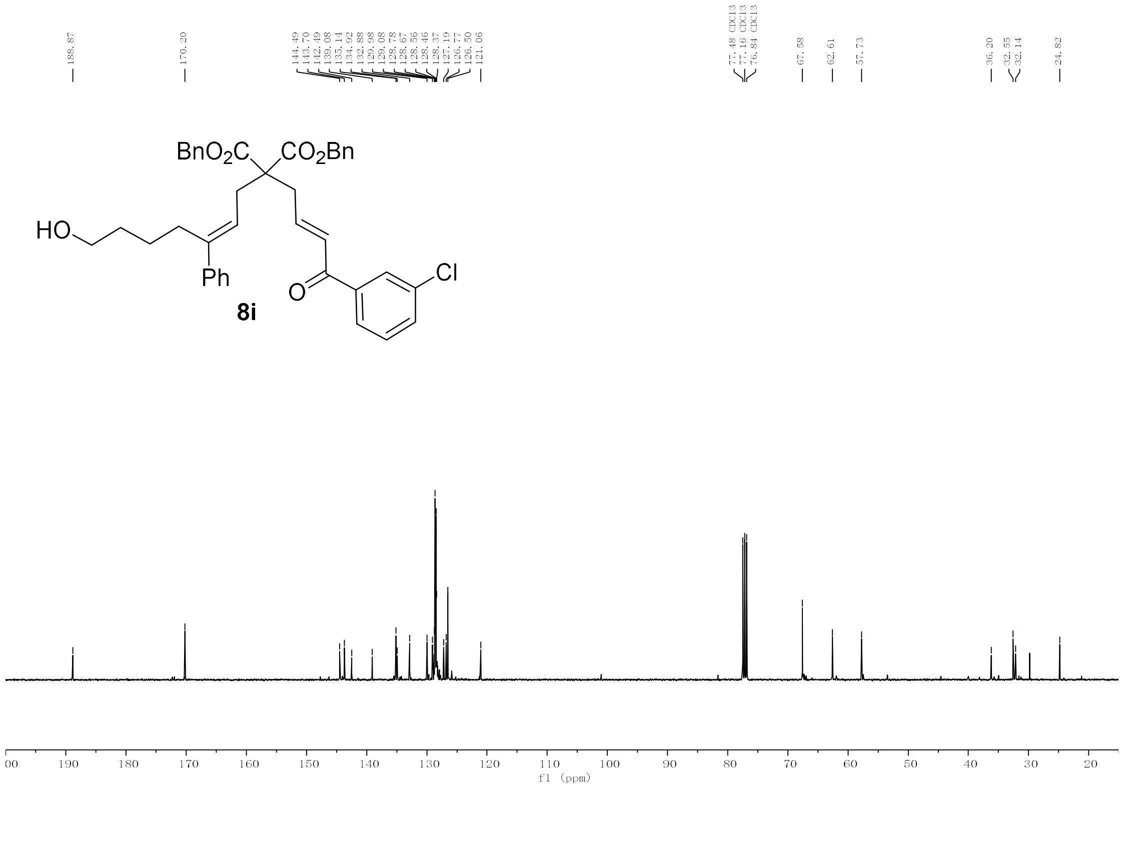

Supplementary Figure 165. ^{1}H NMR spectrum of 8j.

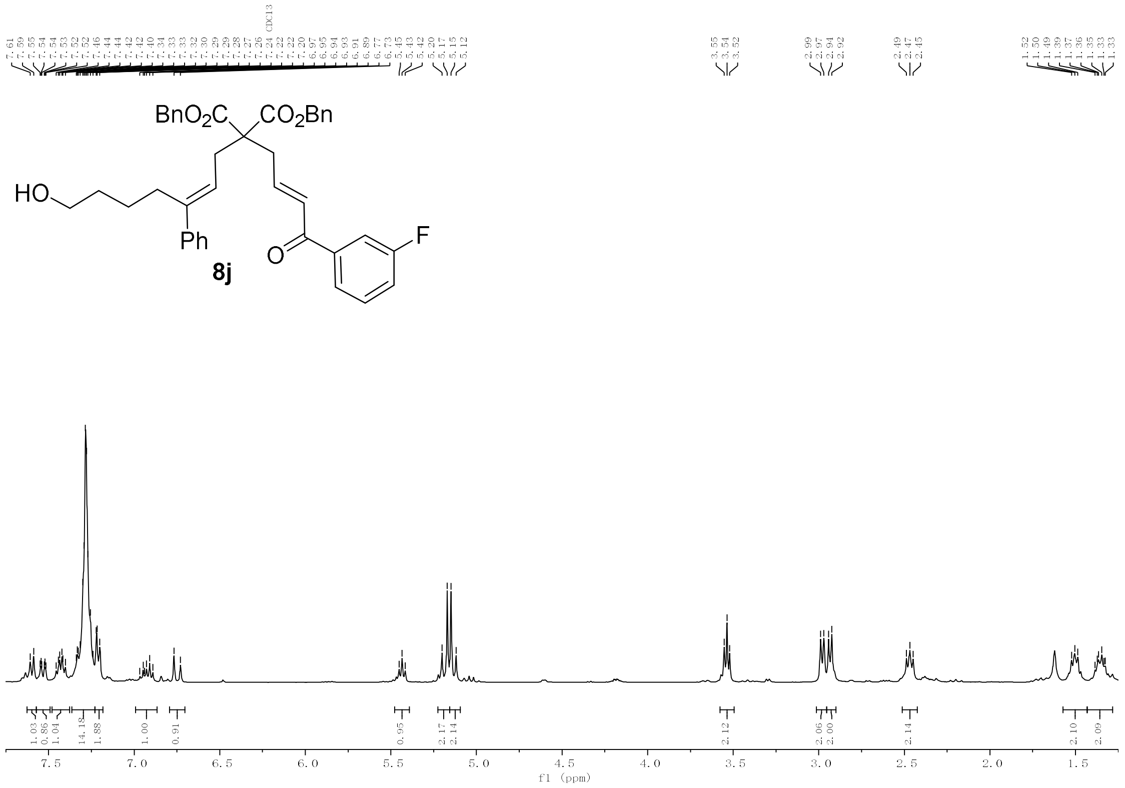

Supplementary Figure 166. ^{13}C NMR spectrum of 8j.

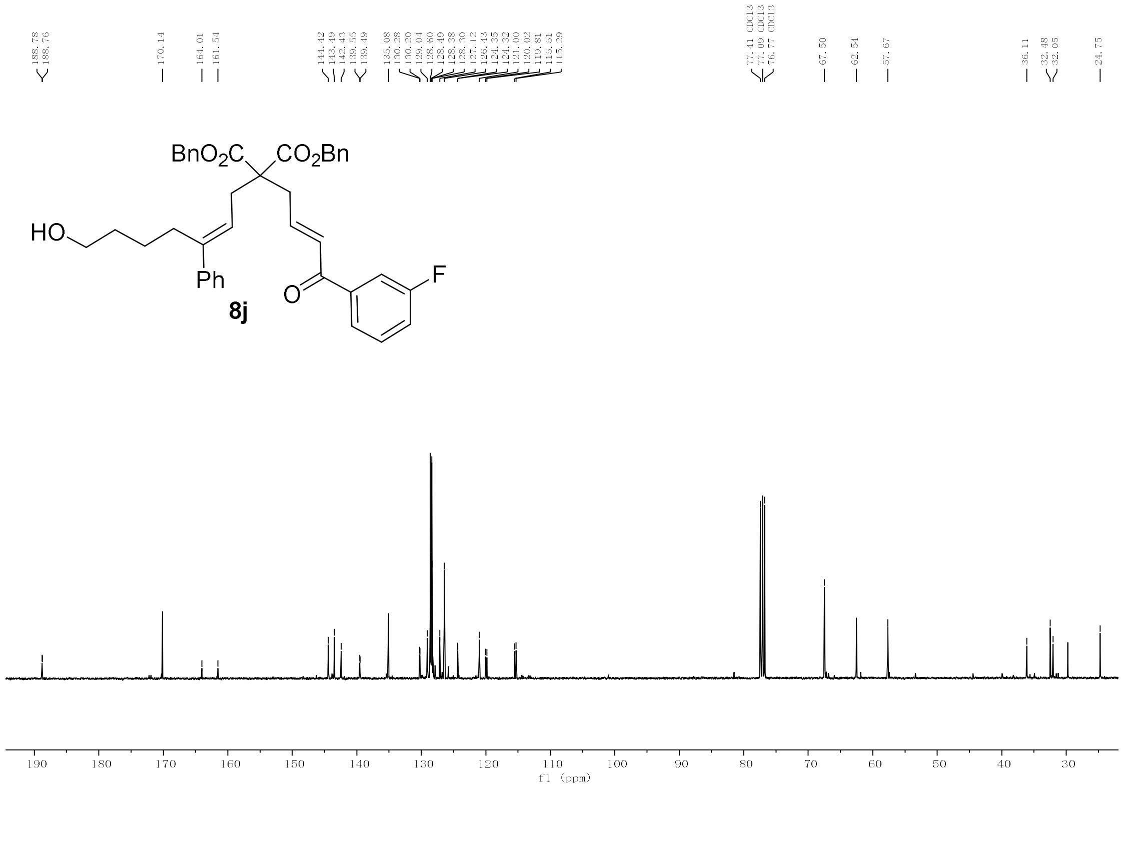

Supplementary Figure 167. [1] H NMR spectrum of 8k.

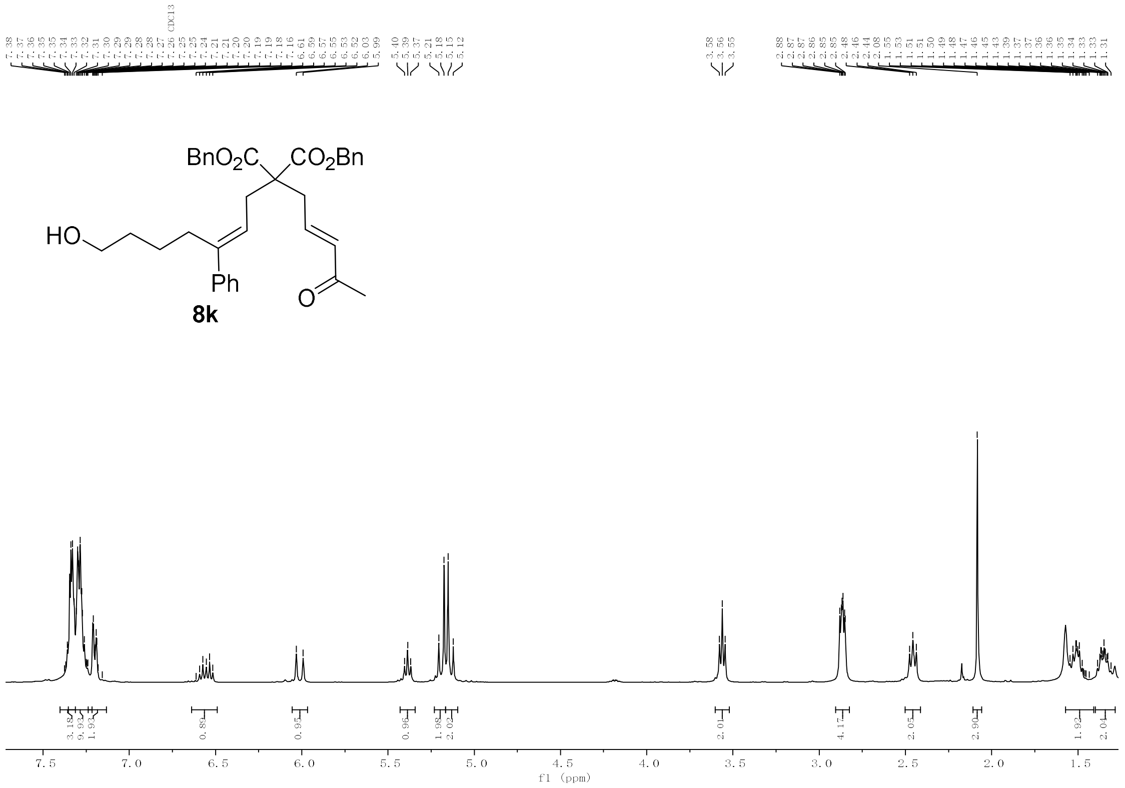

Supplementary Figure 168.[13] C NMR spectrum of 8k.

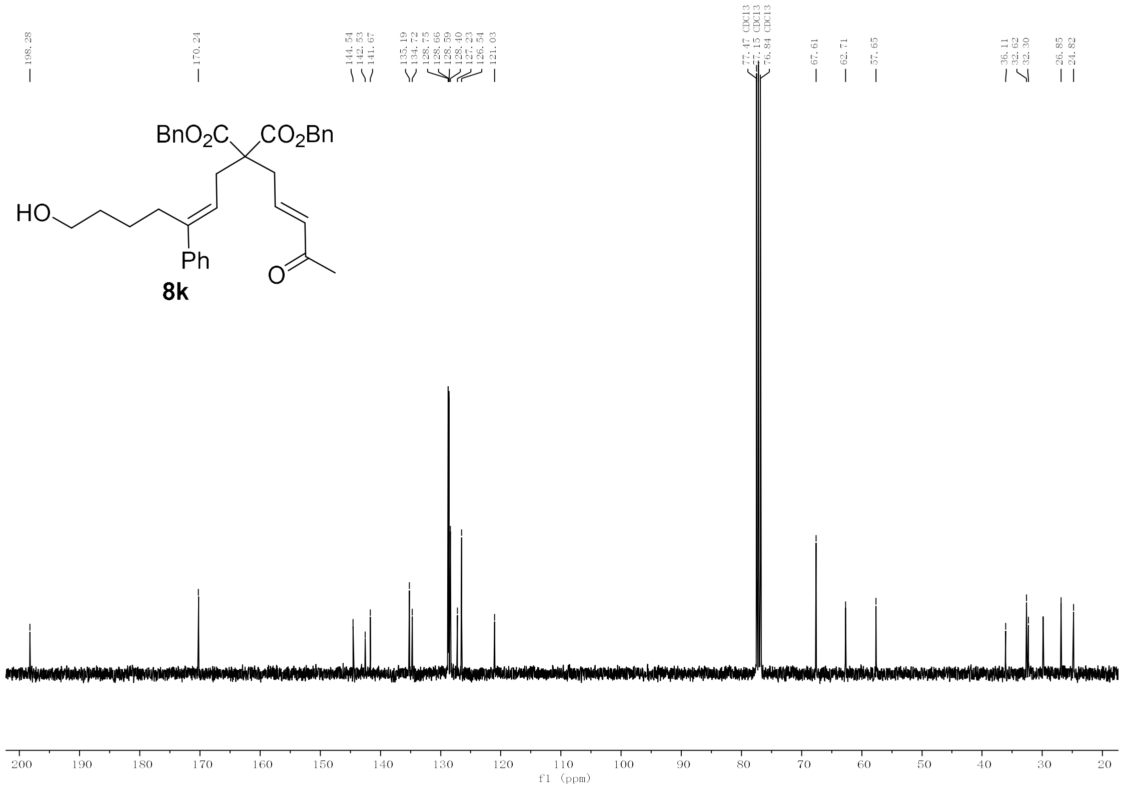

Supplementary Figure 169. ^{1}**H NMR spectrum of 8l**.

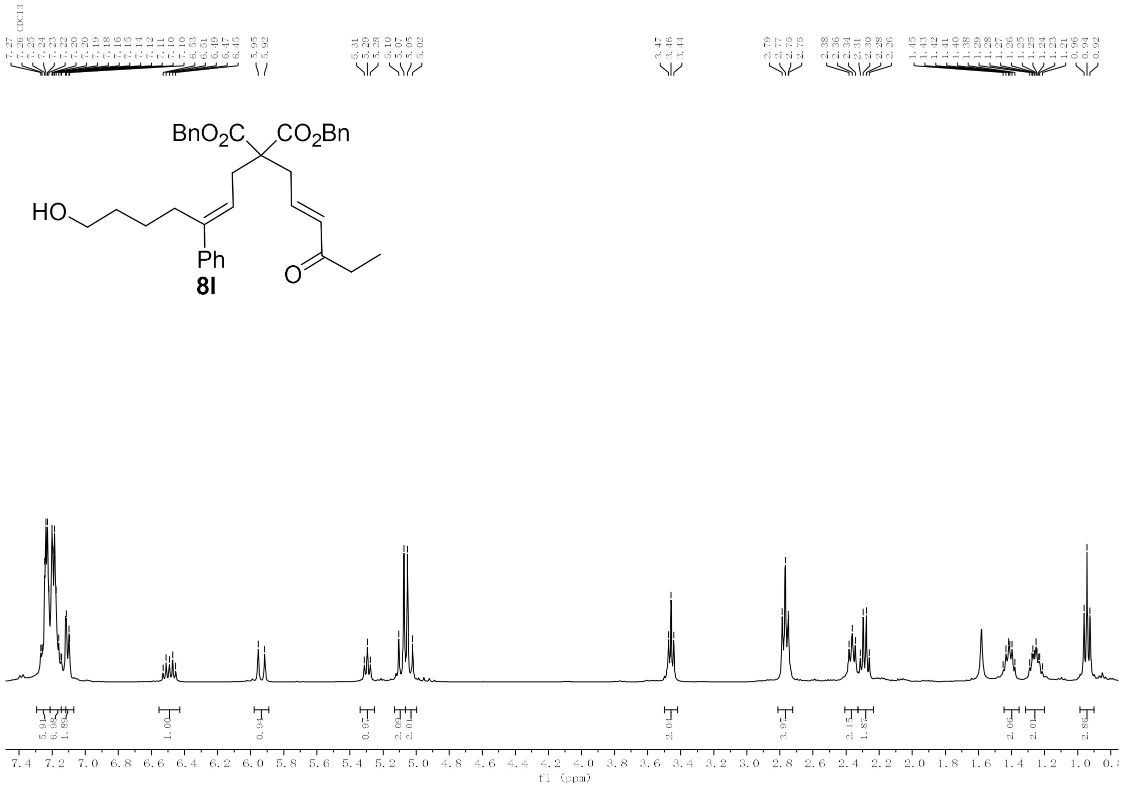

Supplementary Figure 170. ^{13}**C NMR spectrum of 8l**.

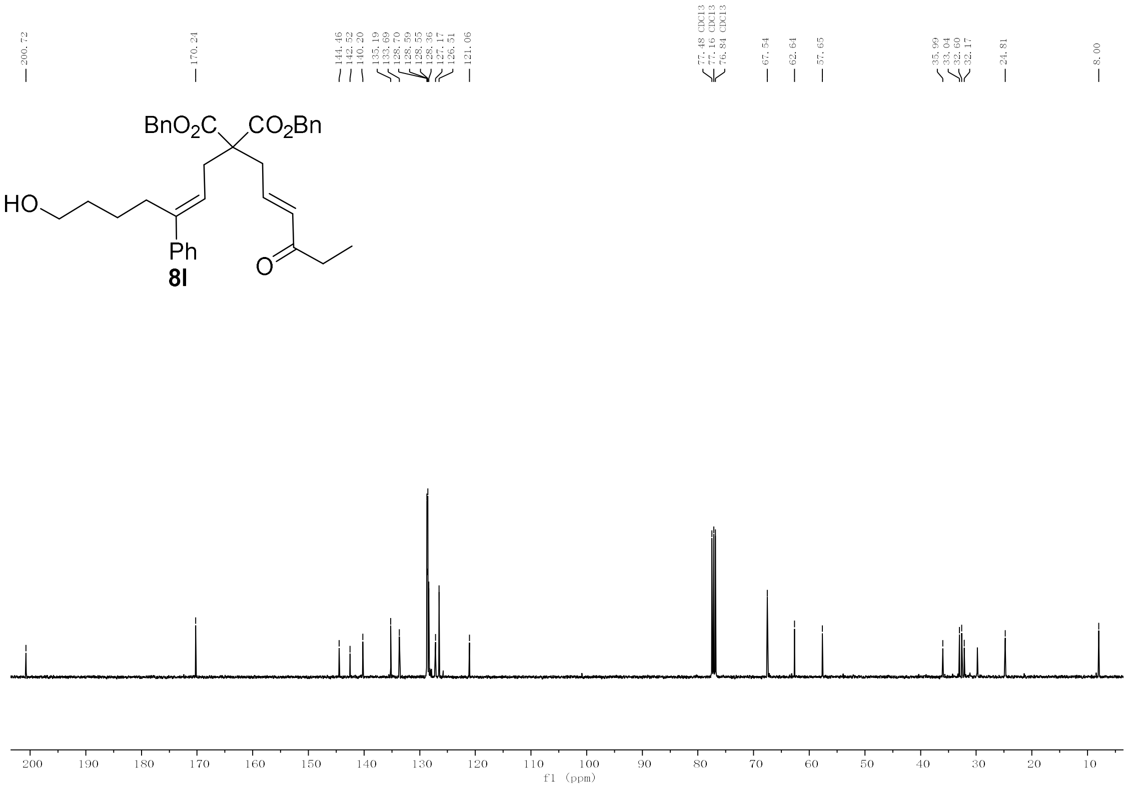

Supplementary Figure 171. [1] H NMR spectrum of 8m.

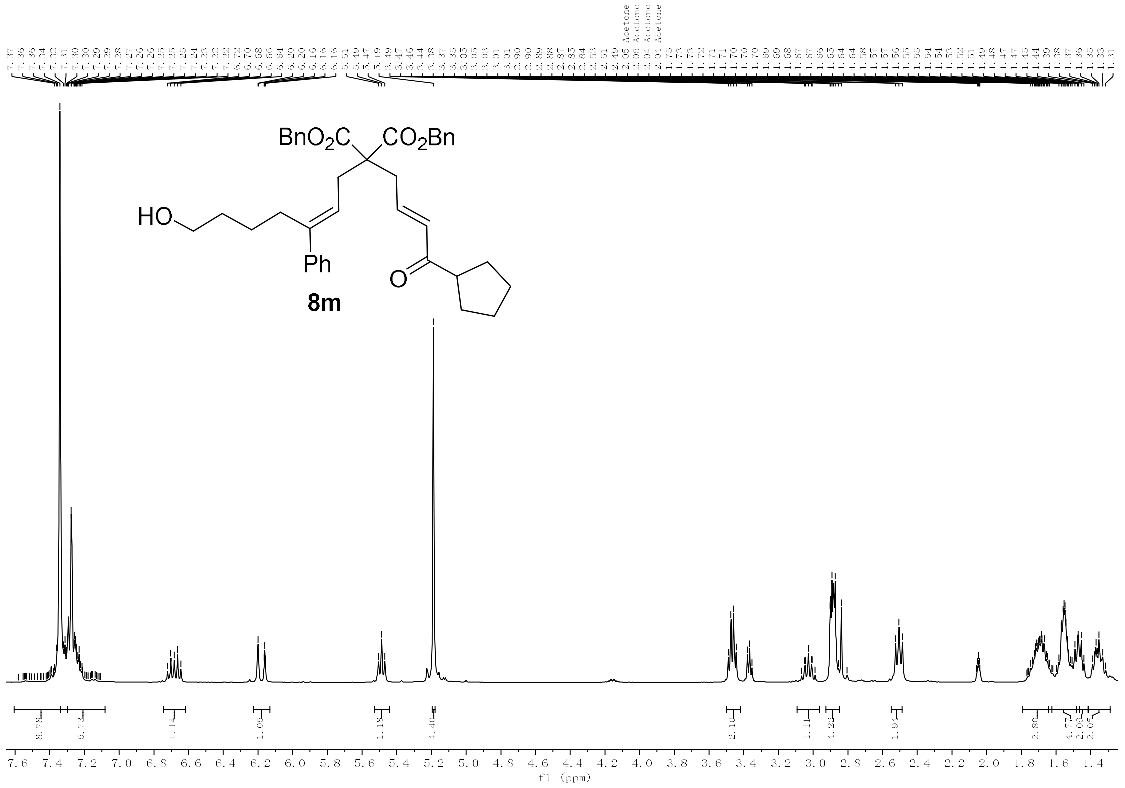

Supplementary Figure 172. [13] C NMR spectrum of 8m.

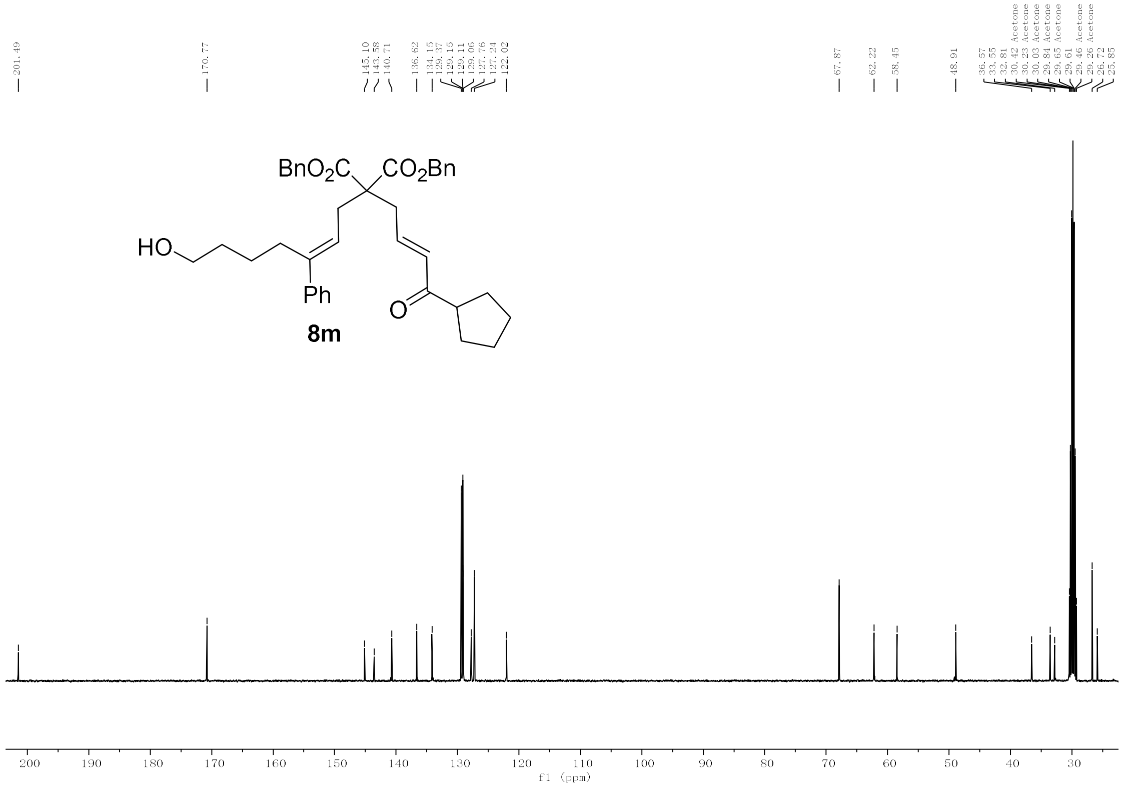

Supplementary Figure 173. [1] H NMR spectrum of 8n.

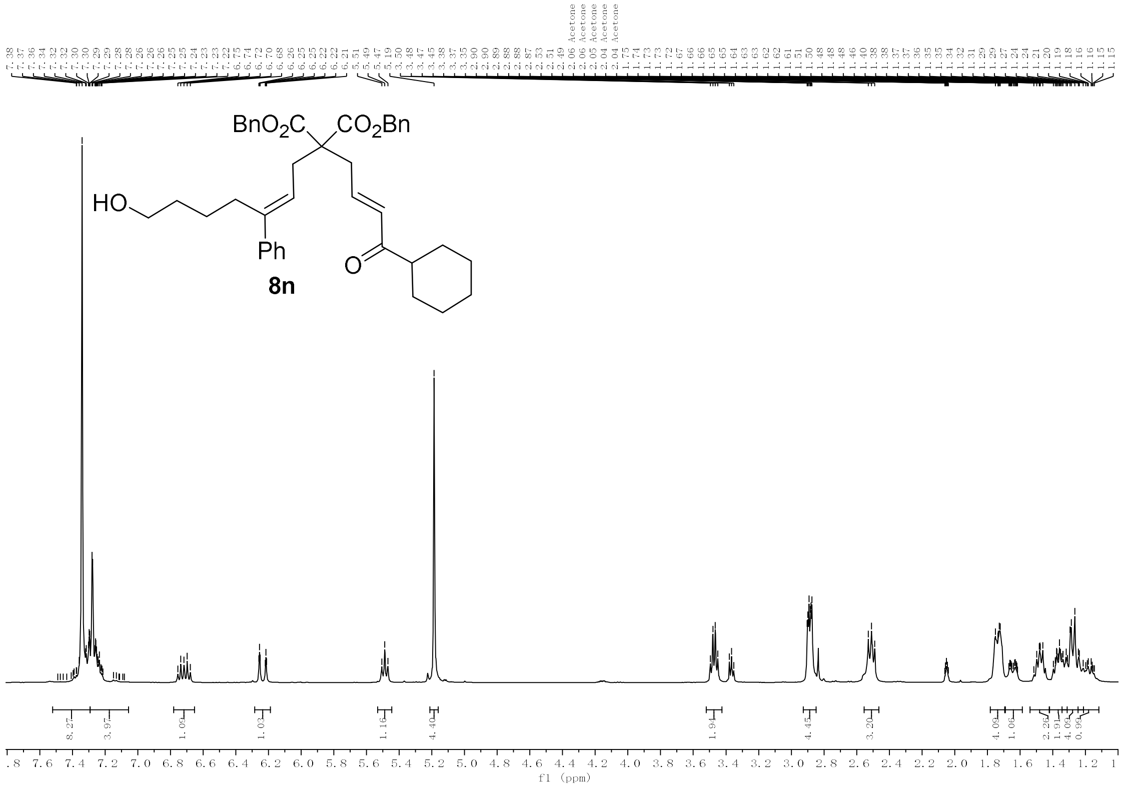

Supplementary Figure 174. [13] C NMR spectrum of 8n.

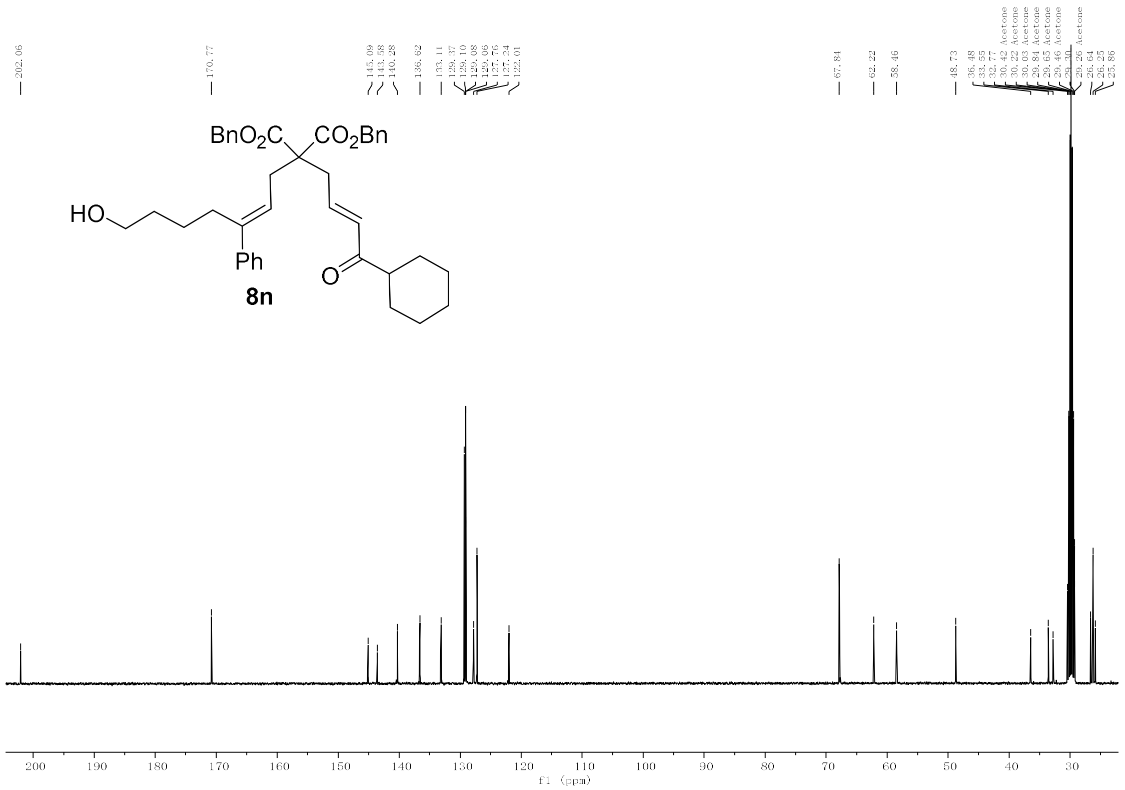

Supplementary Figure 175. ^{1} H NMR spectrum of 9a.

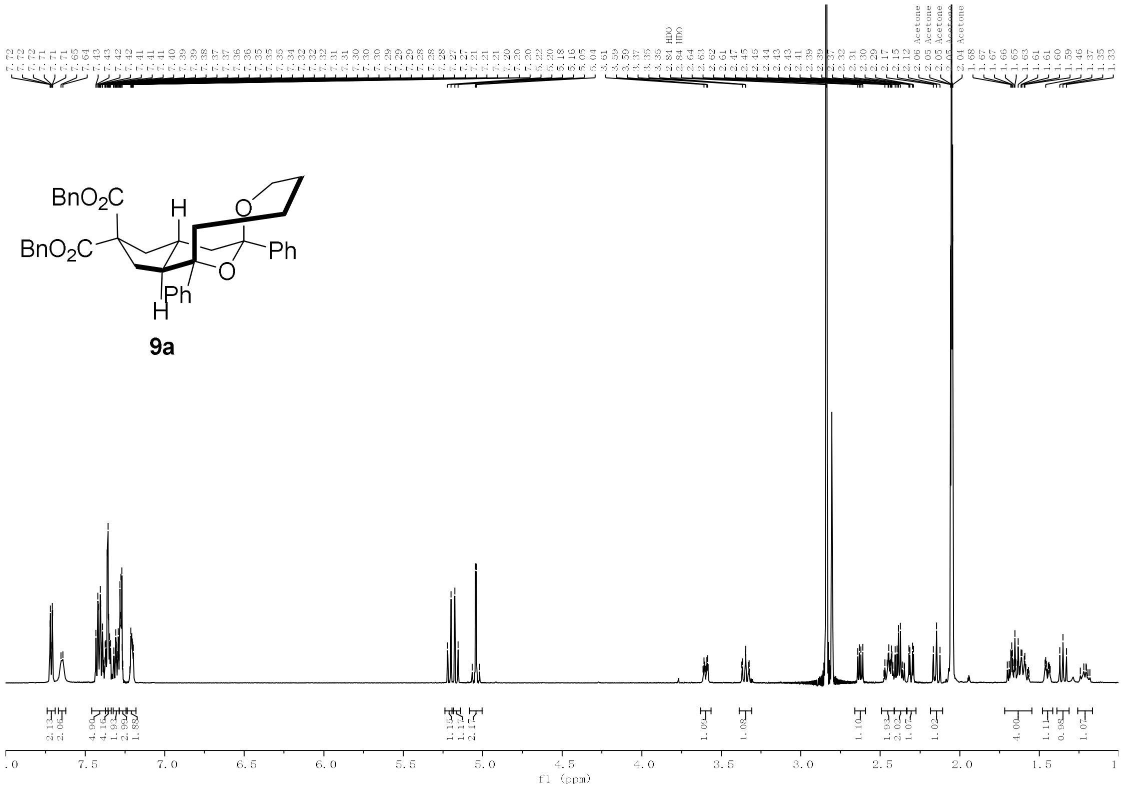

Supplementary Figure 176. ^{13} C NMR spectrum of 9a.

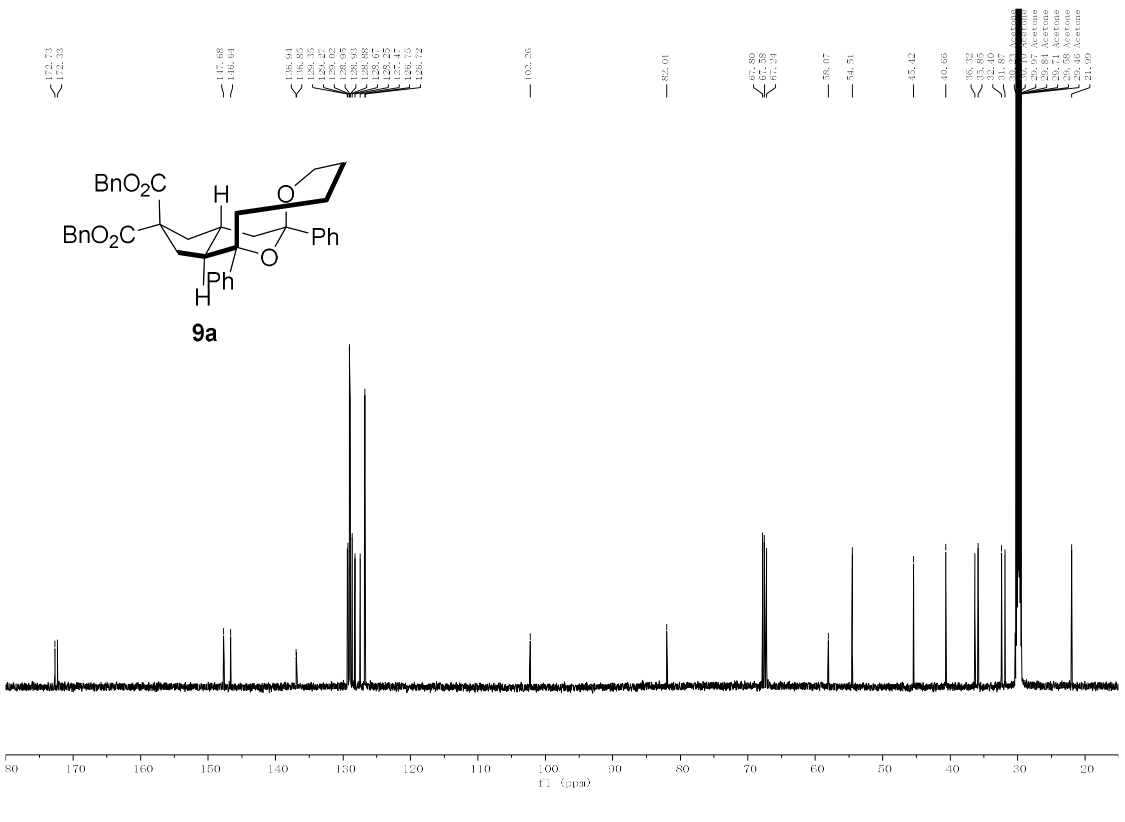

Supplementary Figure 177. ^{1}H NMR spectrum of 9b.

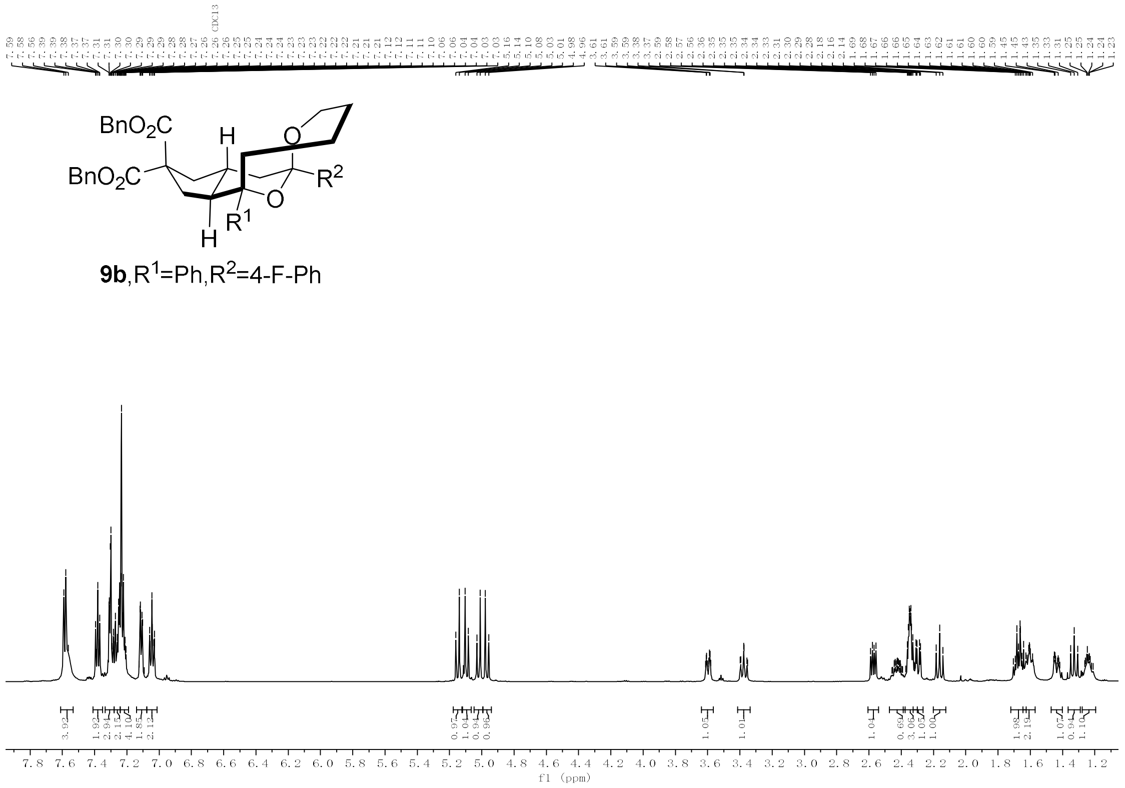

Supplementary Figure 178. ^{13}C NMR spectrum of 9b.

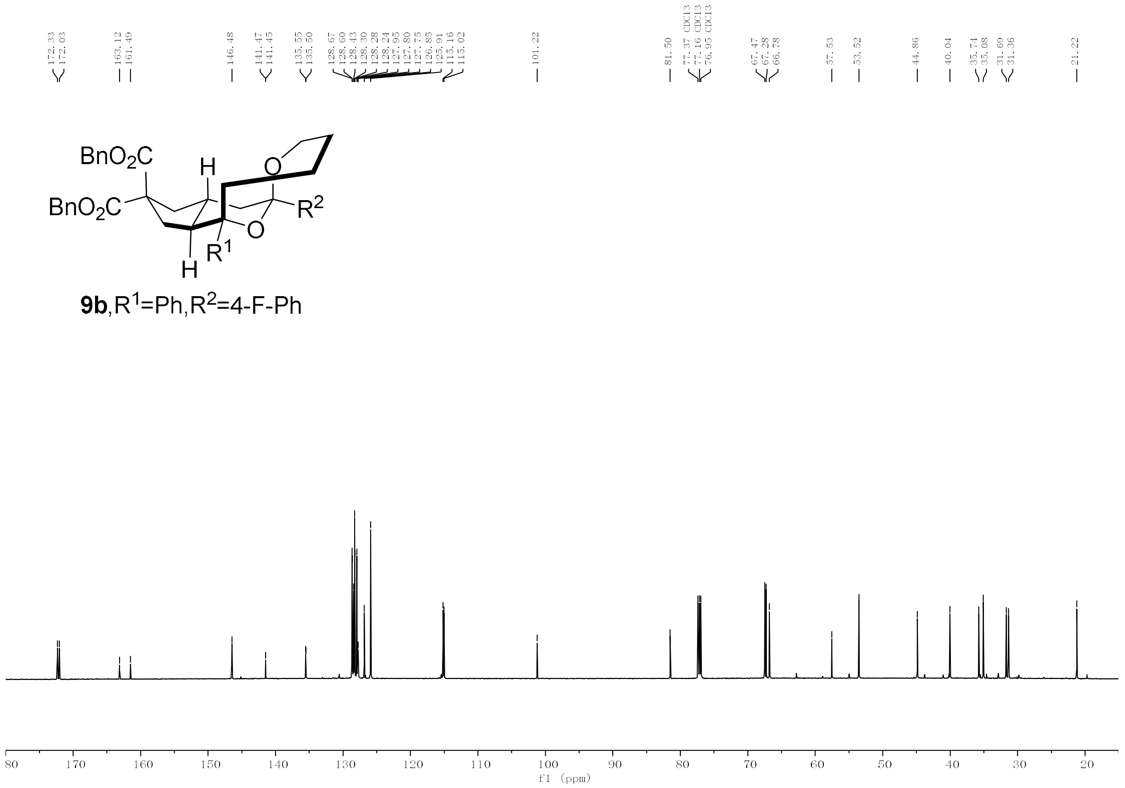

Supplementary Figure 179. 1 H NMR spectrum of 9c.

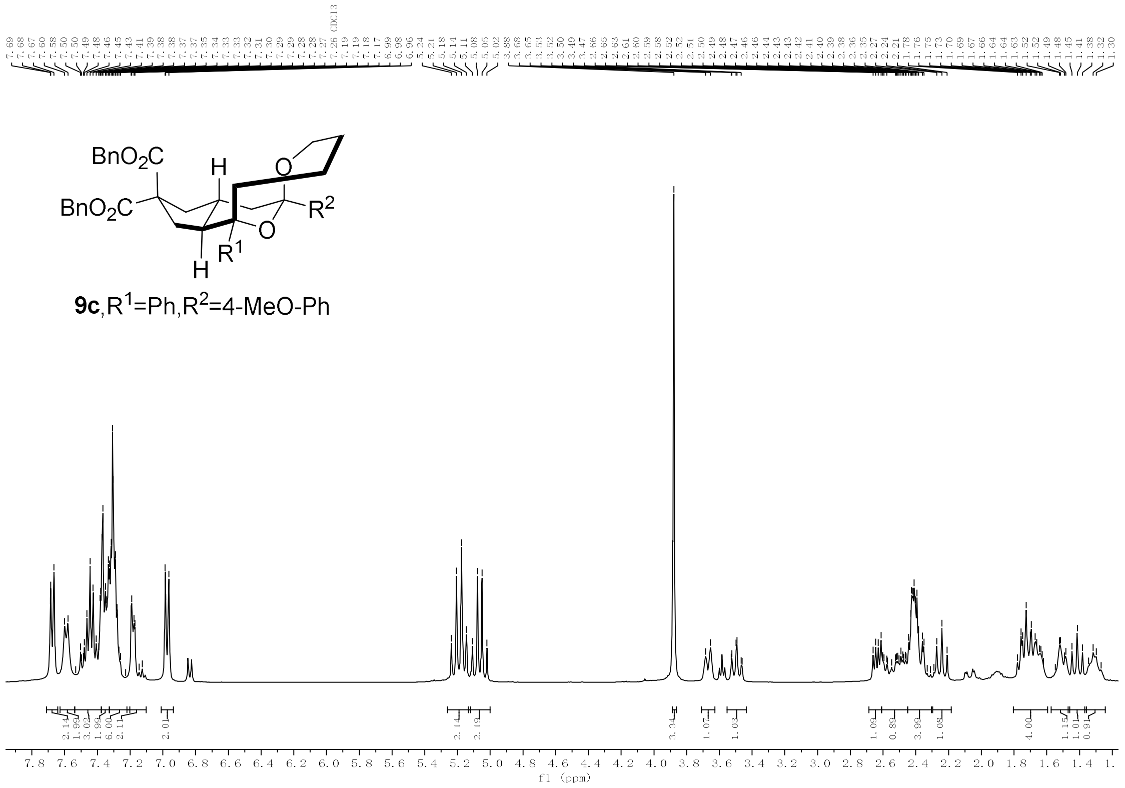

Supplementary Figure 180. 13 C NMR spectrum of 9c.

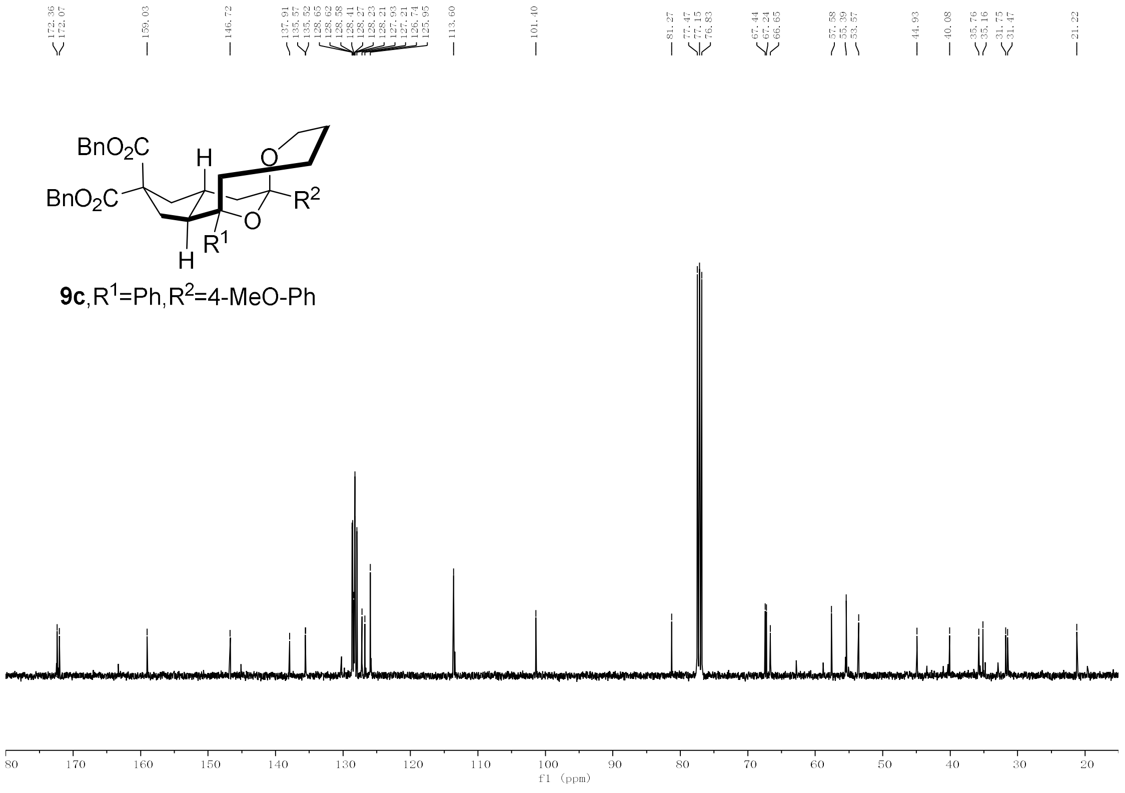

Supplementary Figure 181. ^{1}H NMR spectrum of 9d.

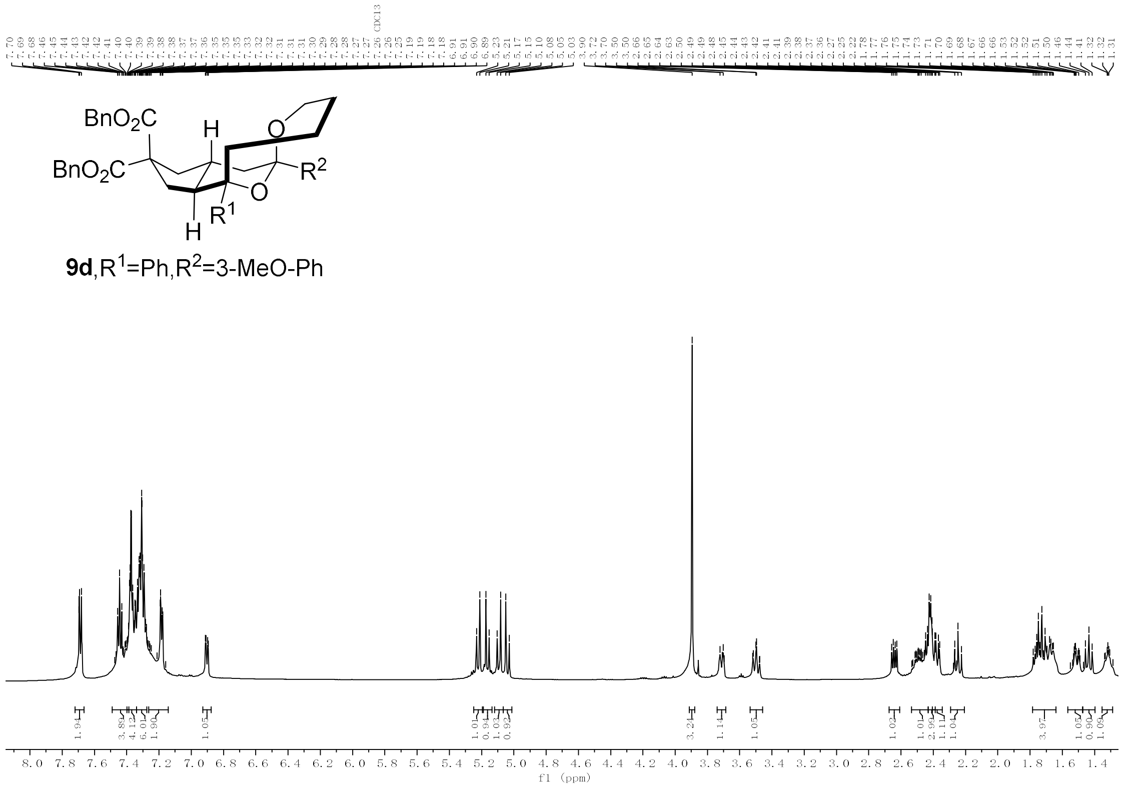

Supplementary Figure 182. ^{13}C NMR spectrum of 9d.

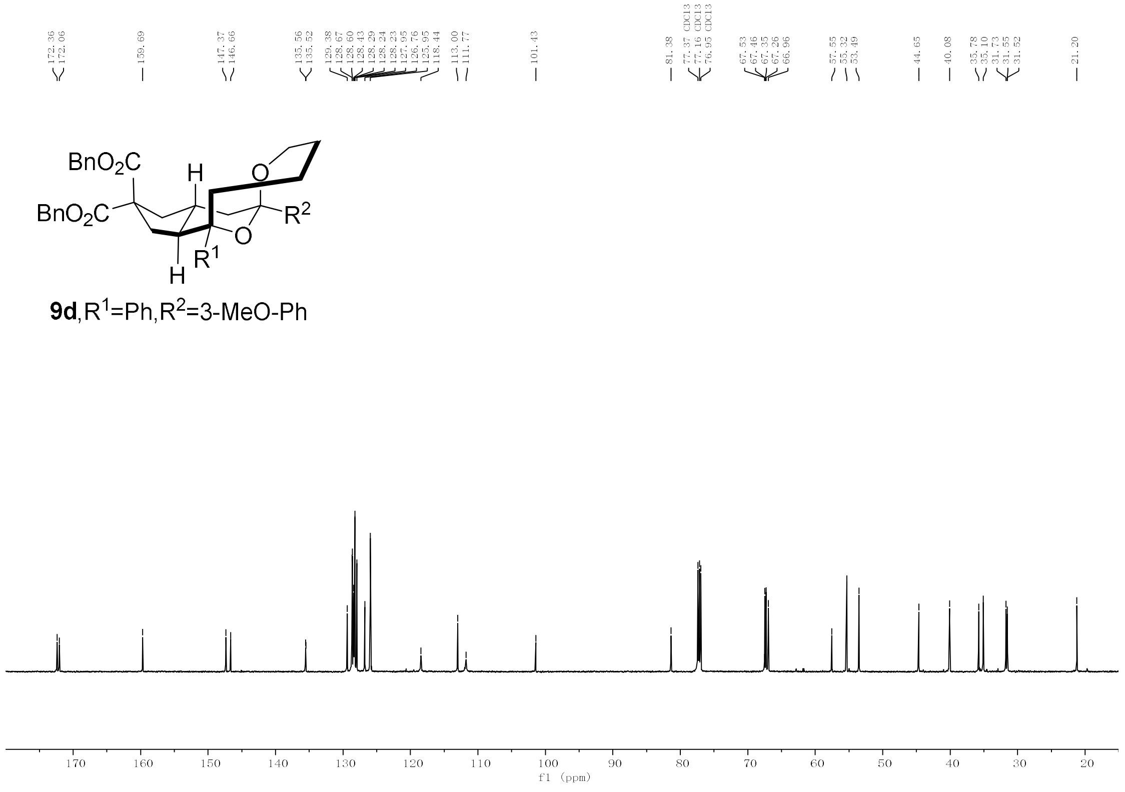

Supplementary Figure 183. ^{1}H NMR spectrum of 9e.

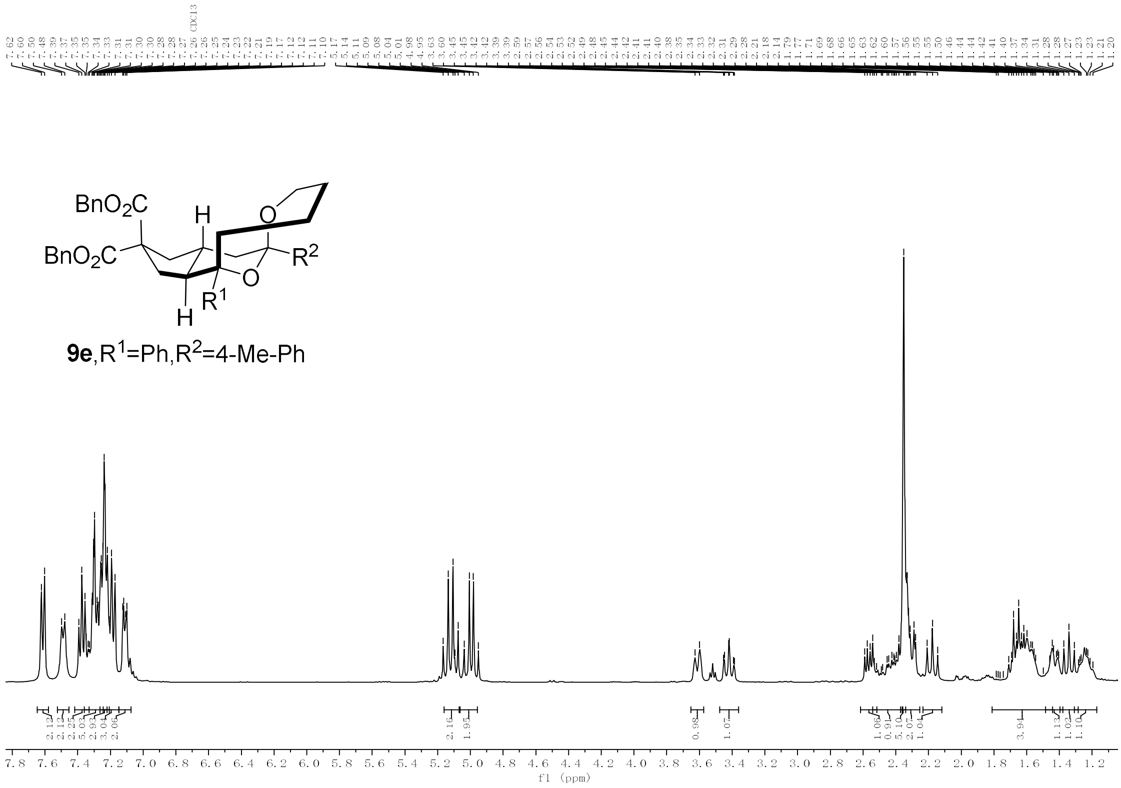

Supplementary Figure 184. ^{13}C NMR spectrum of 9e.

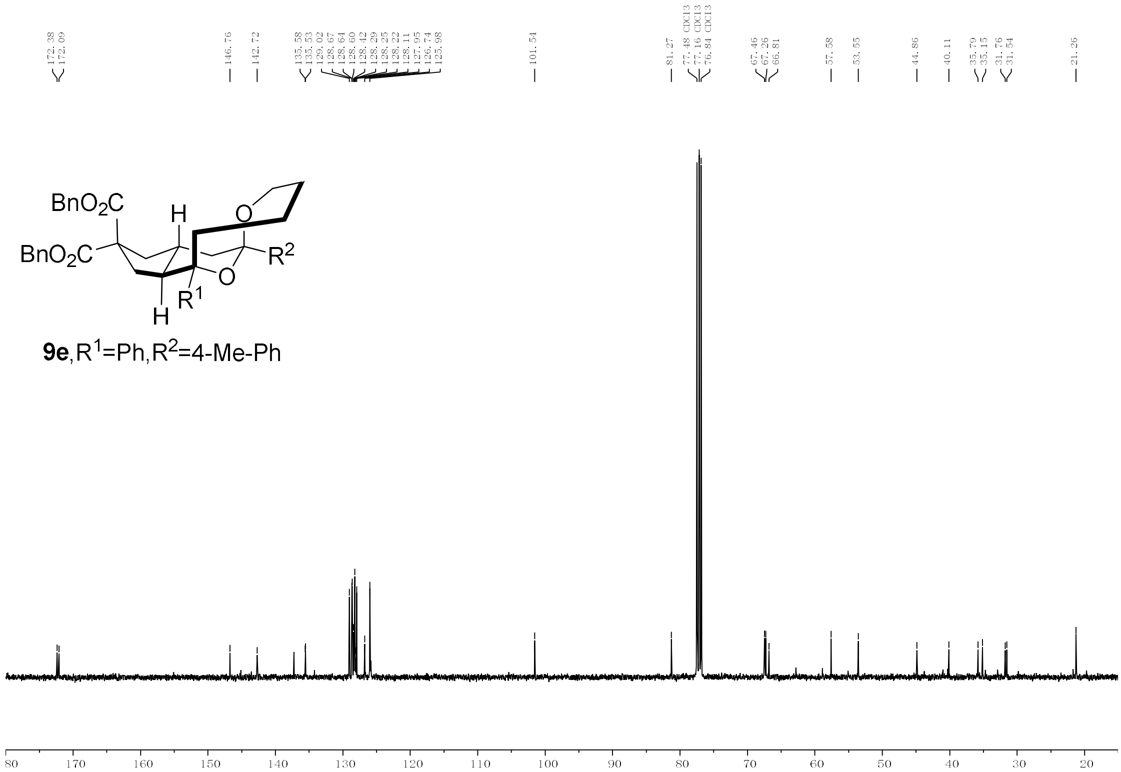

Supplementary Figure 185. [1] H NMR spectrum of 9f.

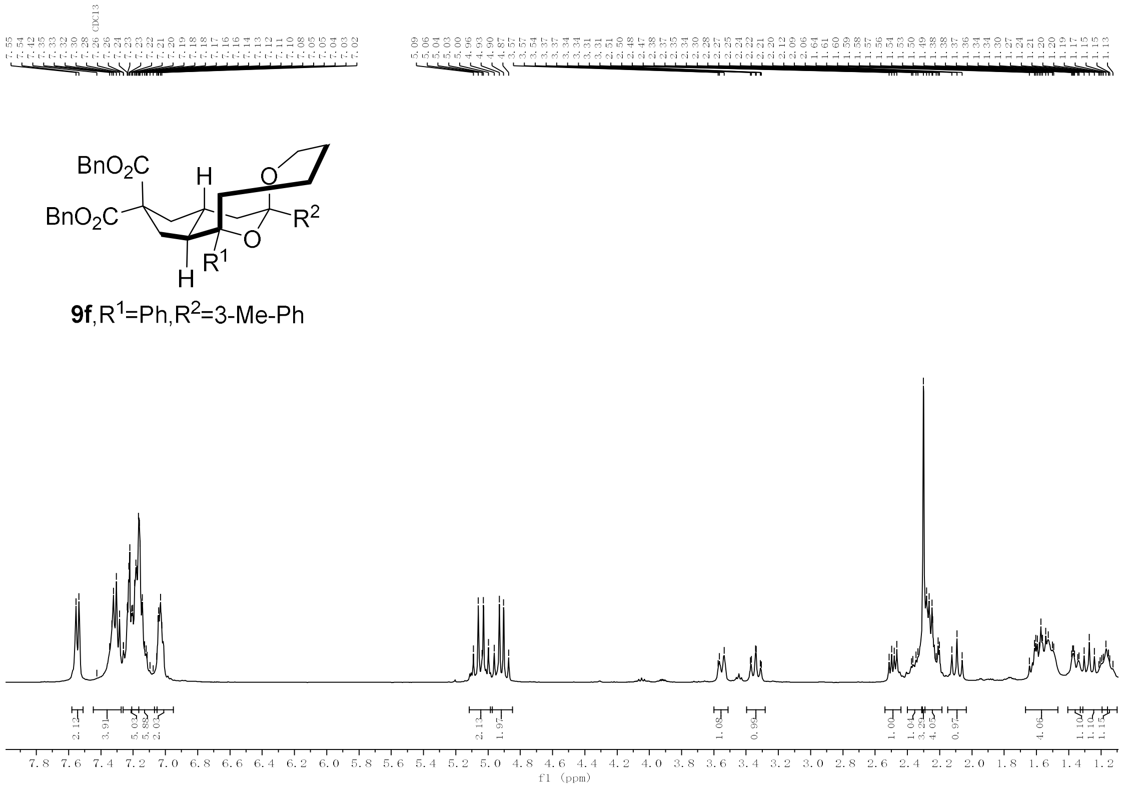

Supplementary Figure 186. [13] C NMR spectrum of 9f.

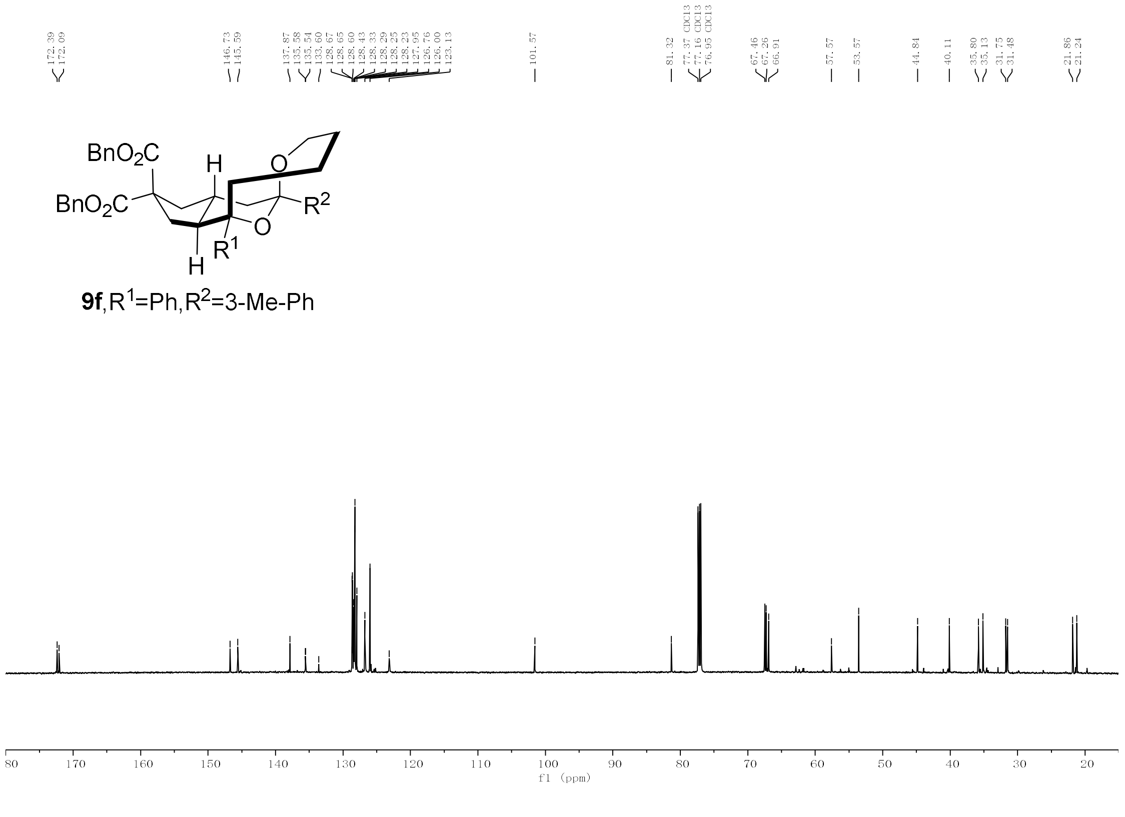

Supplementary Figure 187. ^{1}H NMR spectrum of 9g.

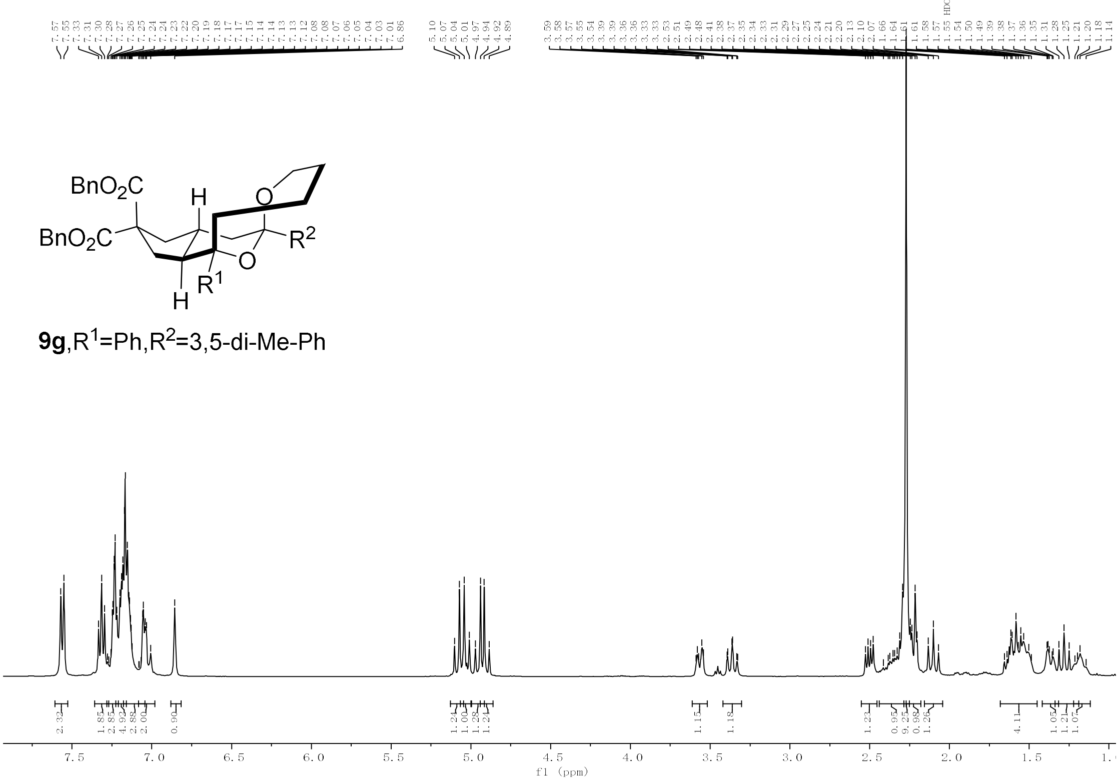

Supplementary Figure 188. ^{13}C NMR spectrum of 9g.

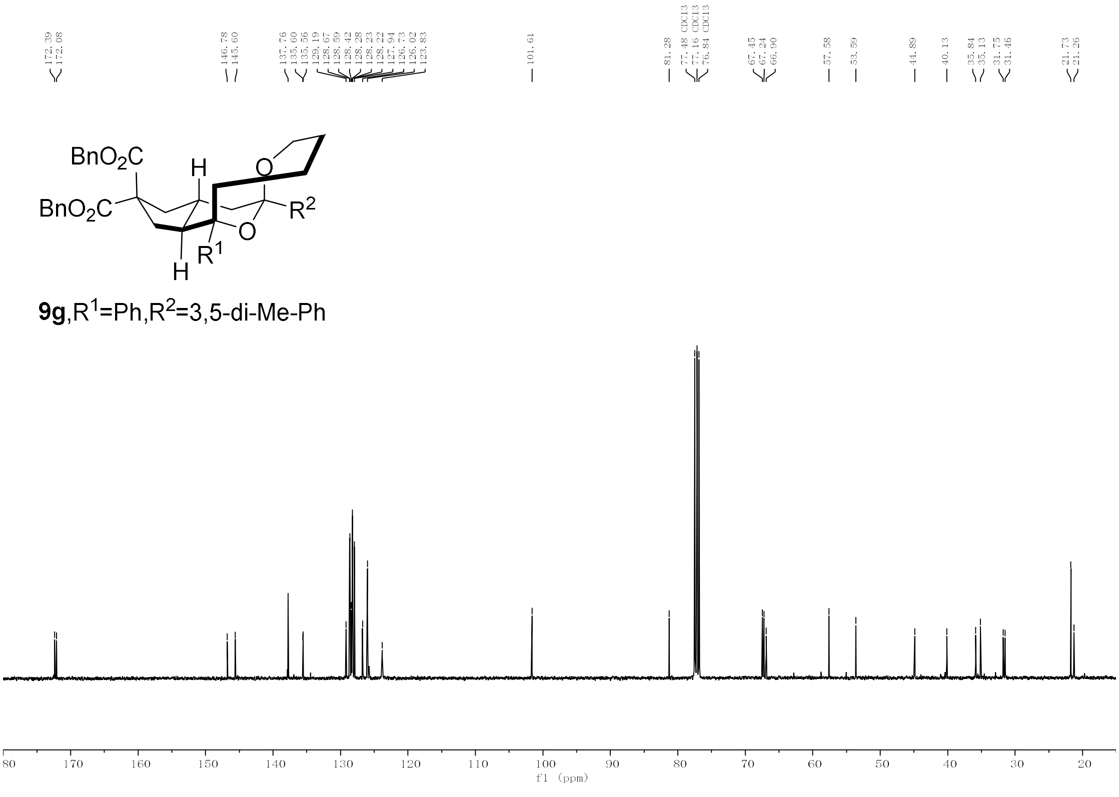

Supplementary Figure 189. [1] H NMR spectrum of 9h.

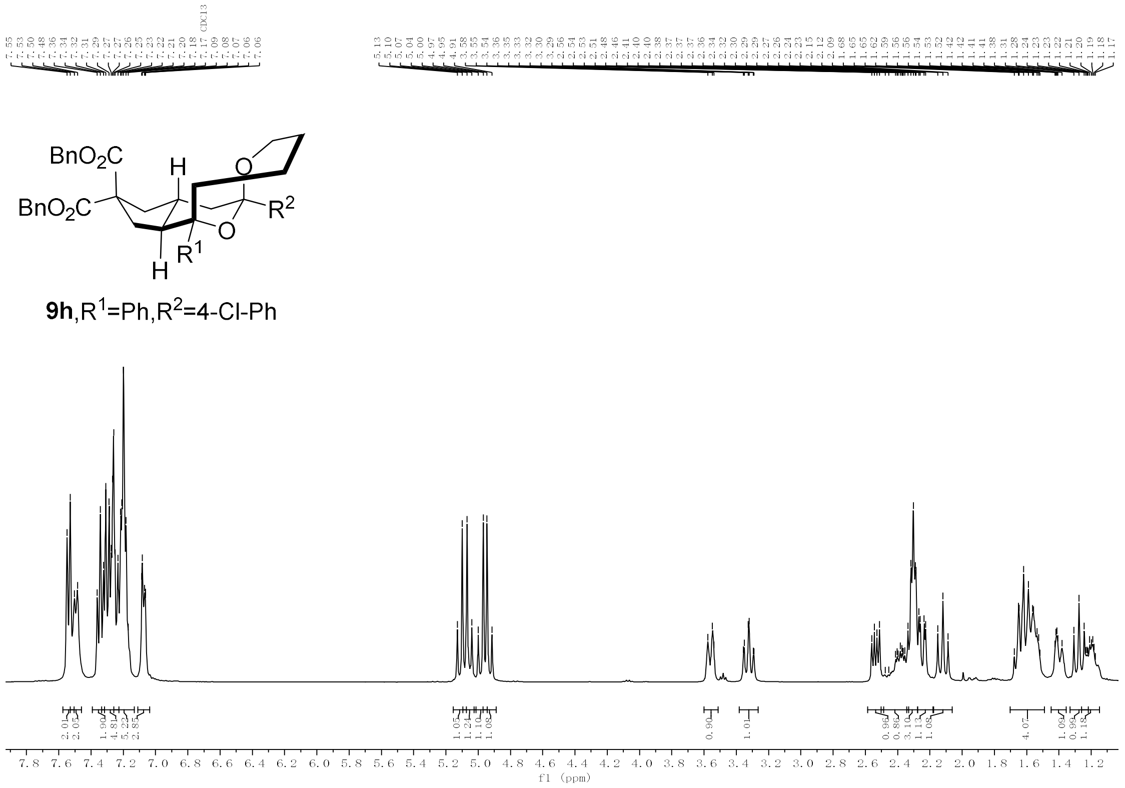

Supplementary Figure 190. [13] C NMR spectrum of 9h.

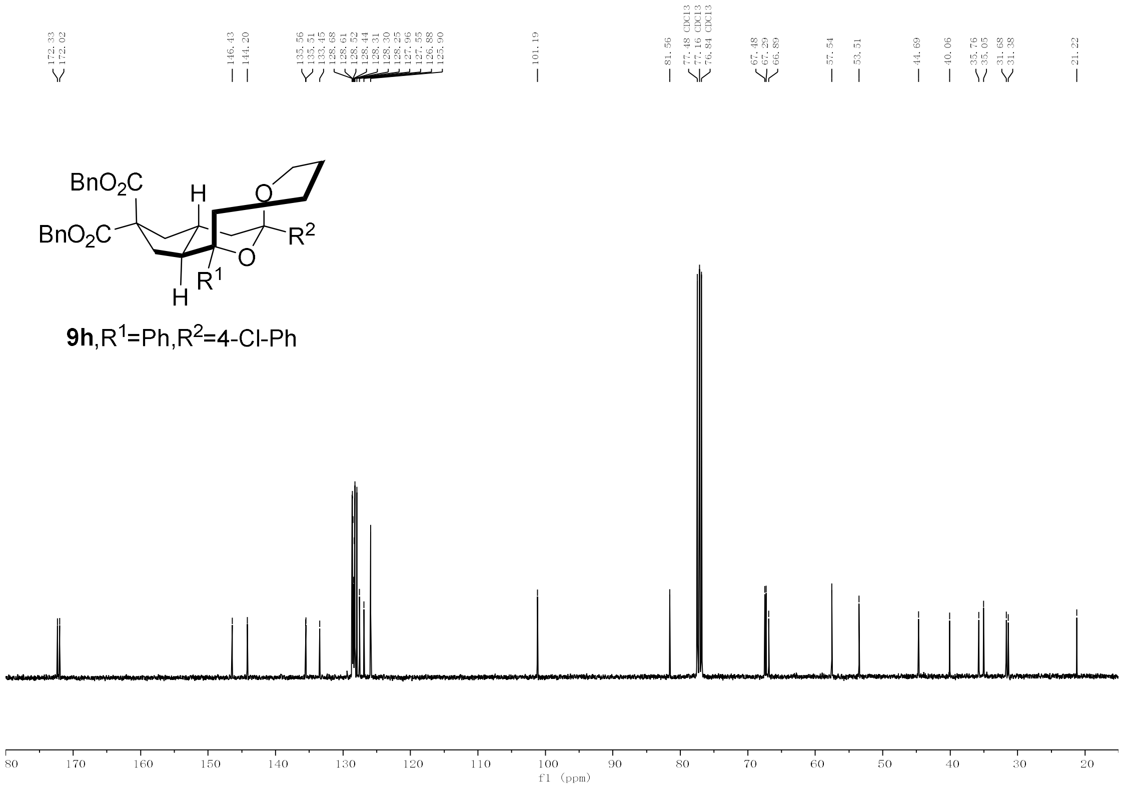

Supplementary Figure 191. ¹ H NMR spectrum of 9i.

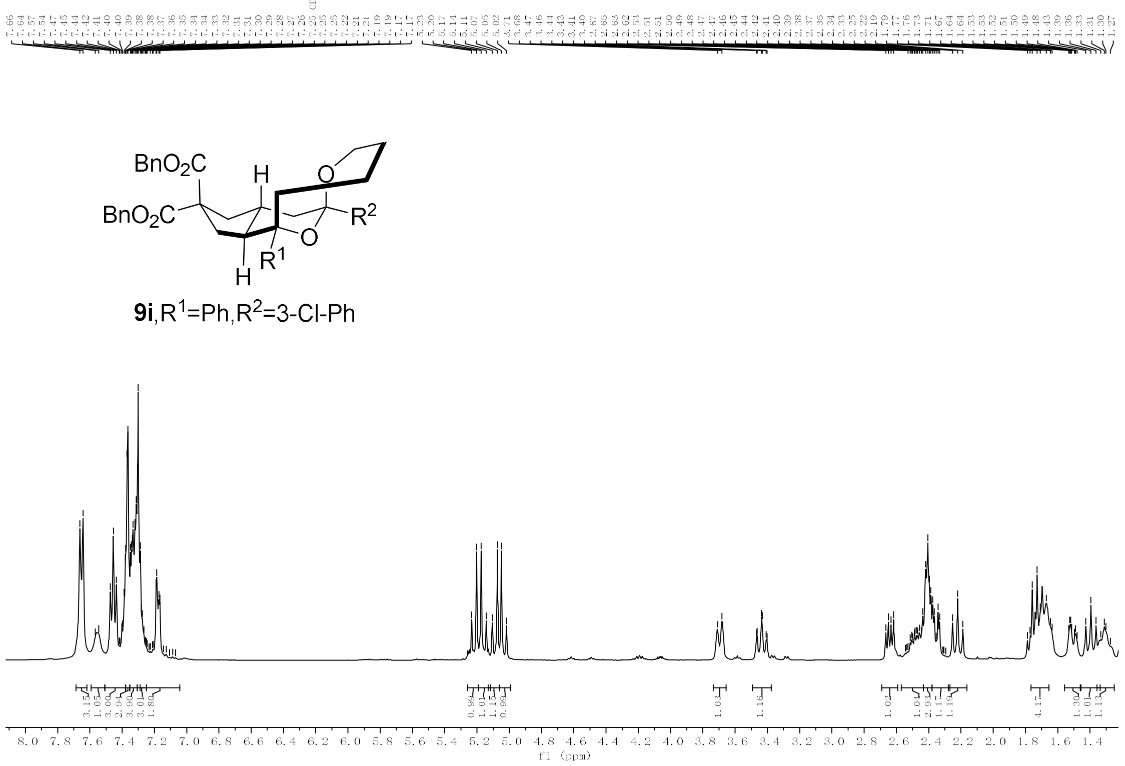

Supplementary Figure 192. ¹³ C NMR spectrum of 9i.

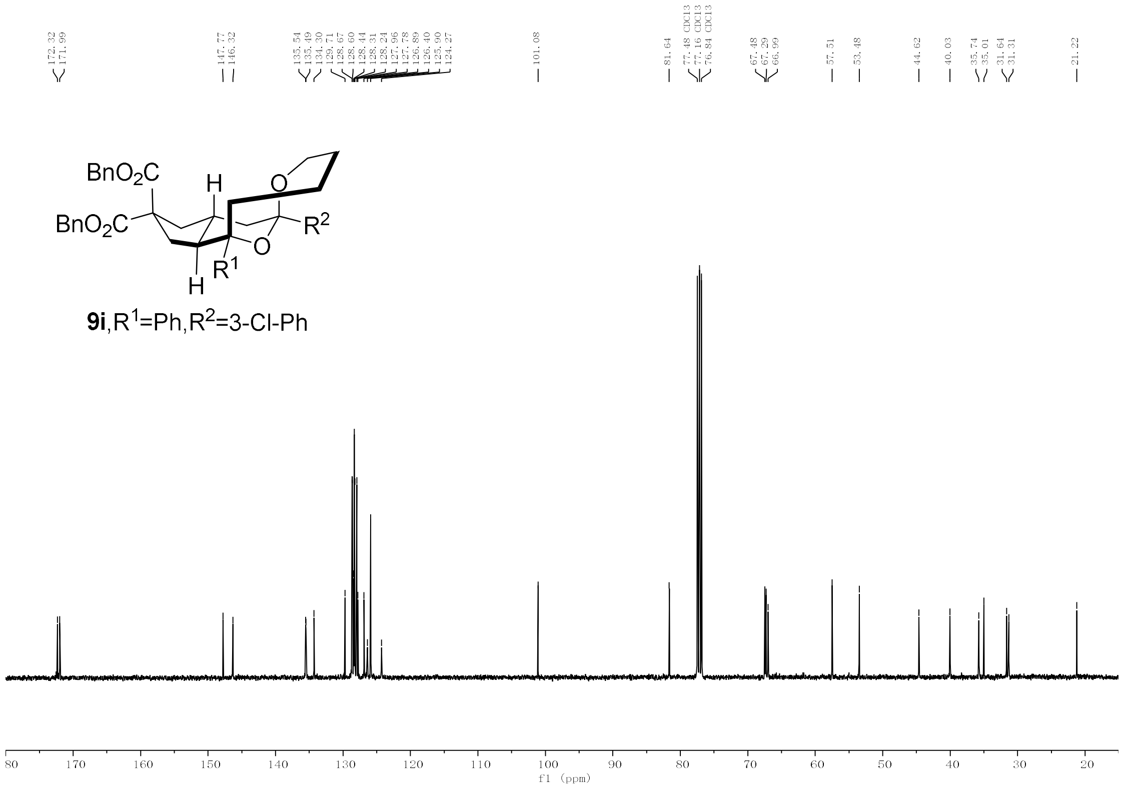

Supplementary Figure 193. ¹ H NMR spectrum of 9j.

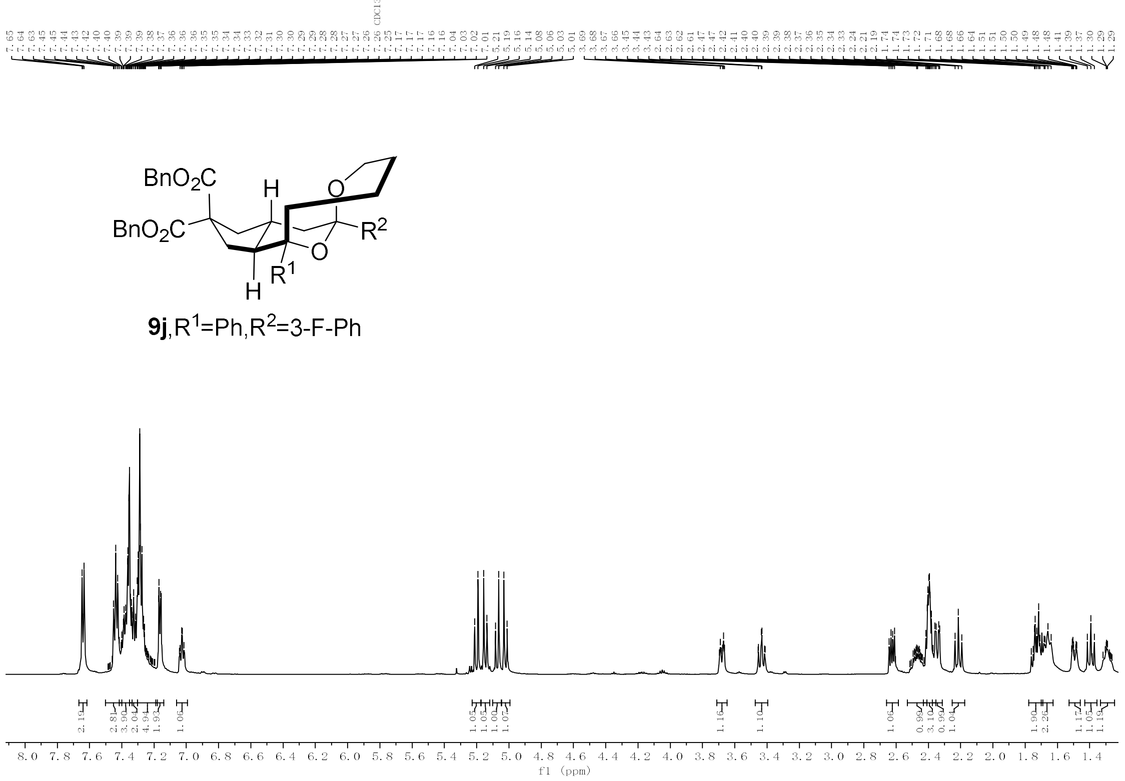

Supplementary Figure 194. ¹³ C NMR spectrum of 9j.

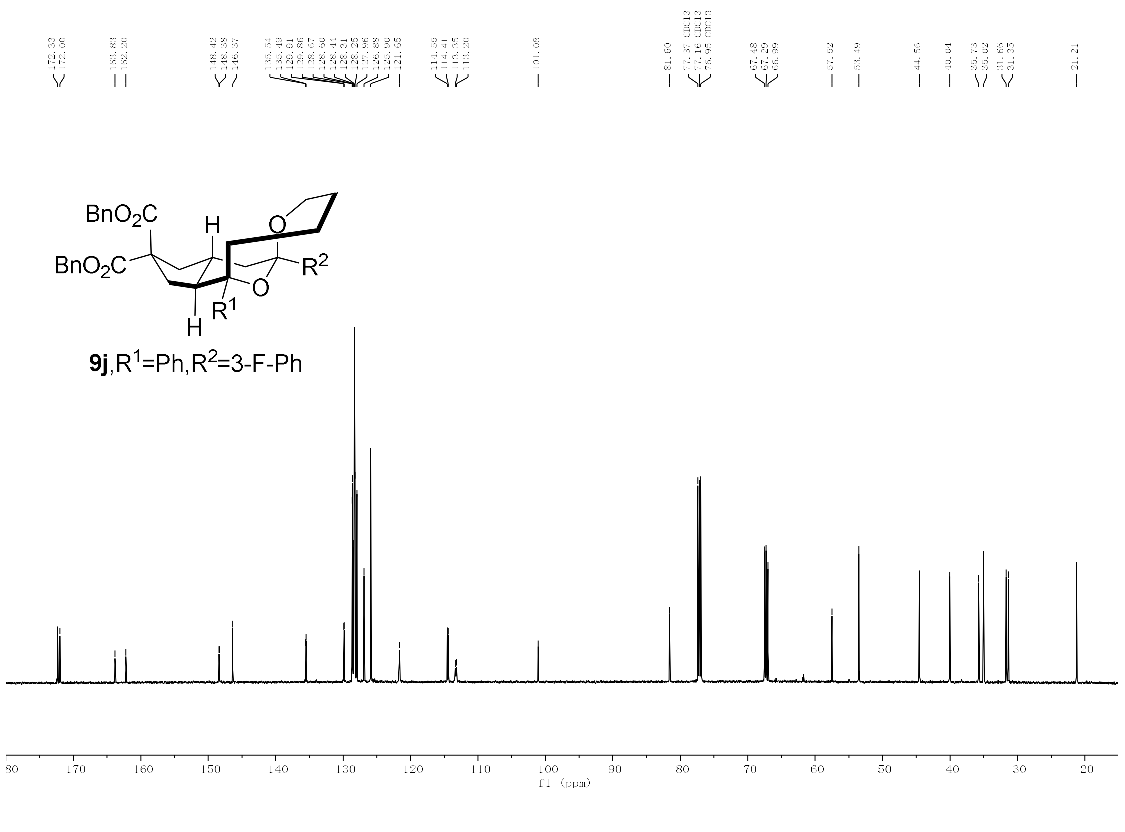

Supplementary Figure 195. ^{1}H NMR spectrum of 9k.

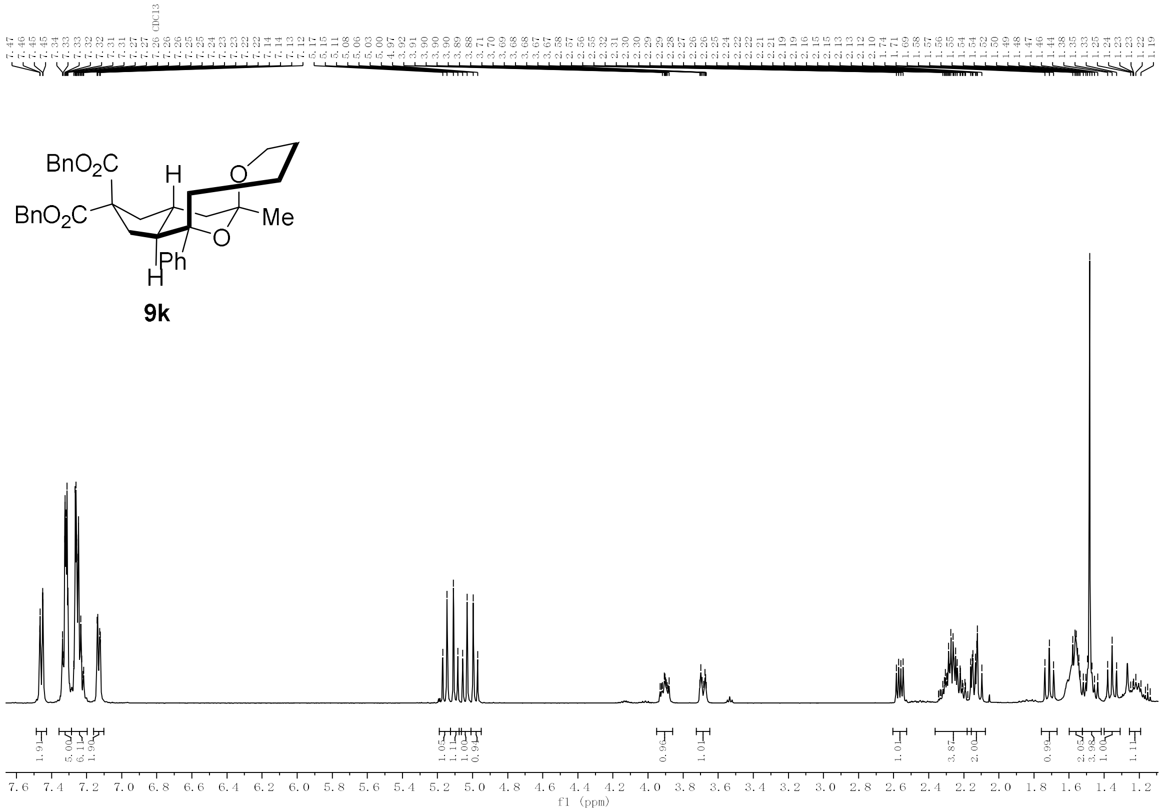

Supplementary Figure 196. ^{13}C NMR spectrum of 9k.

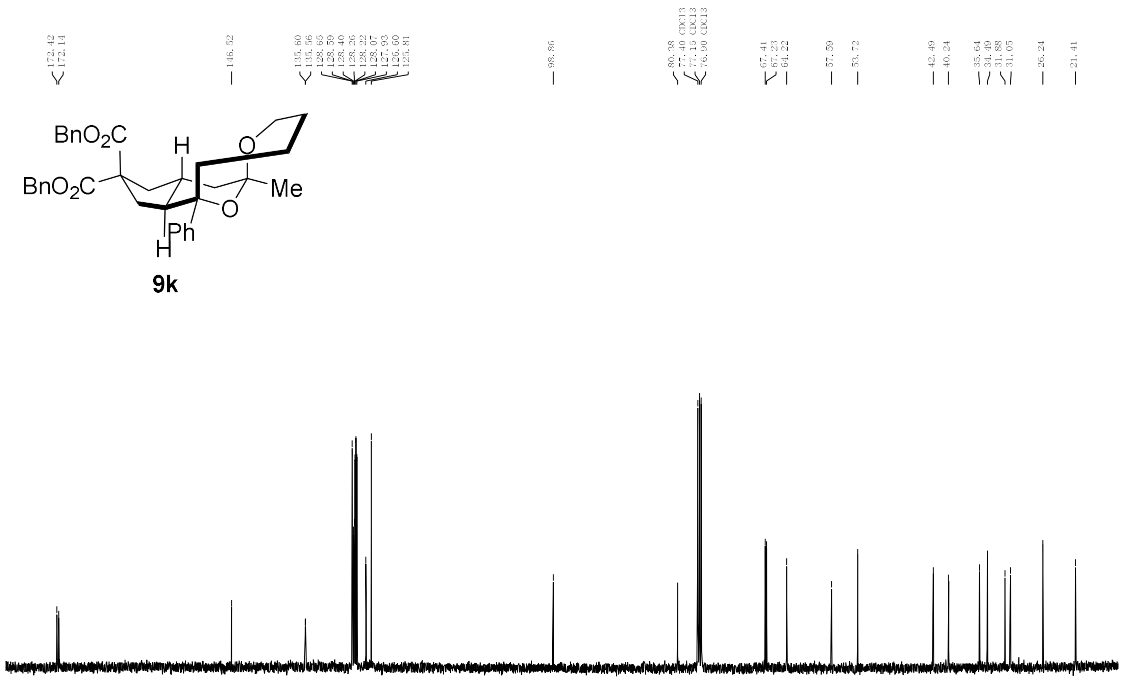

Supplementary Figure 197. [1] H NMR spectrum of 9l.

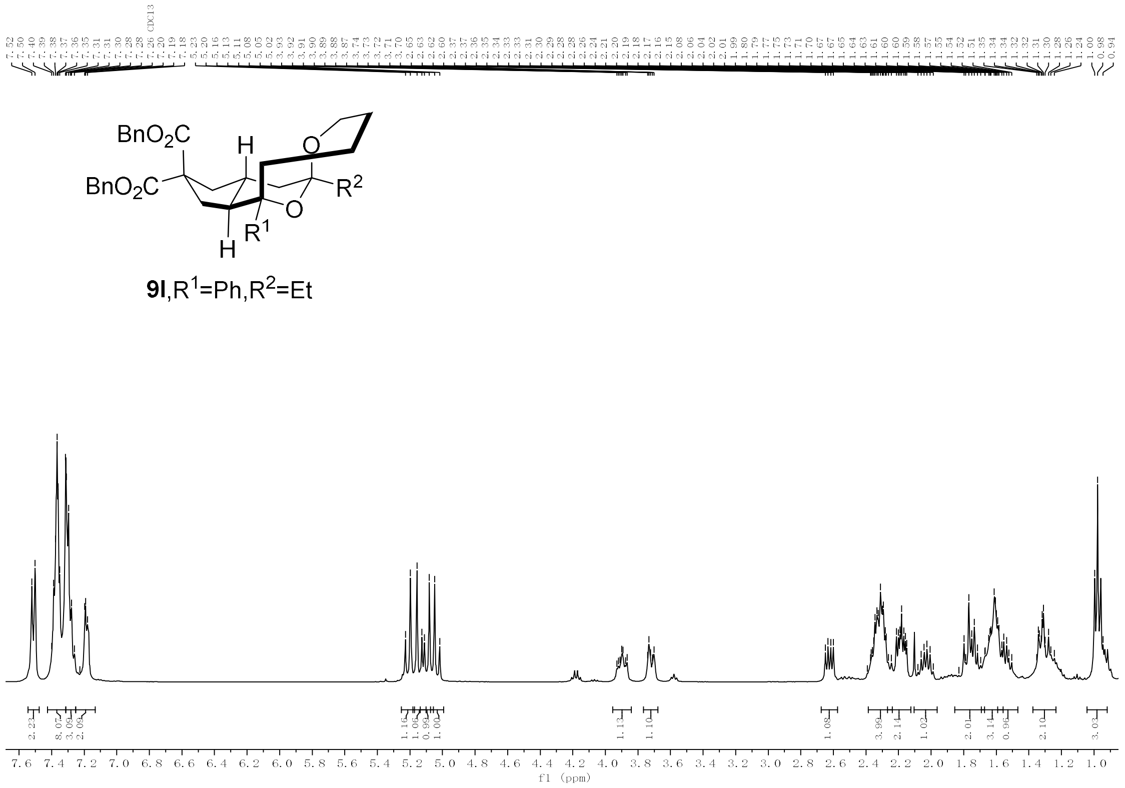

Supplementary Figure 198. [13] C NMR spectrum of 9l.

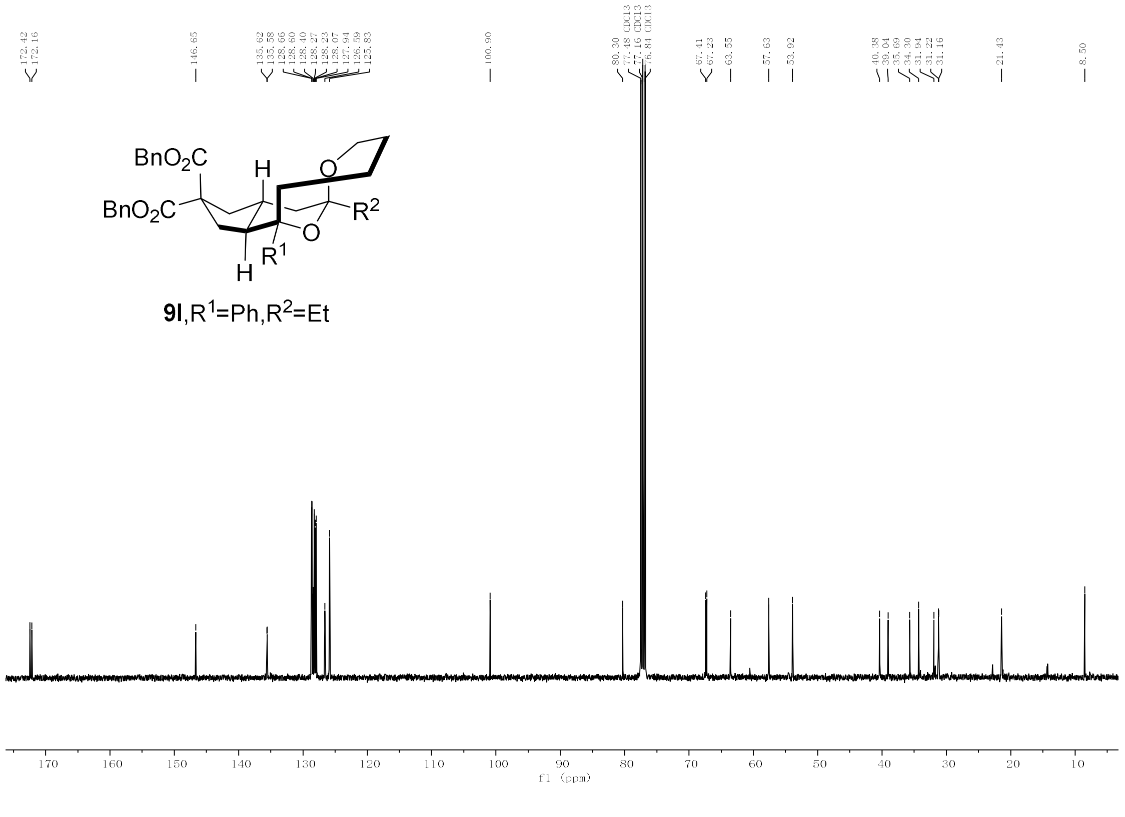

Supplementary Figure 199. ^{1}H NMR spectrum of 9m.

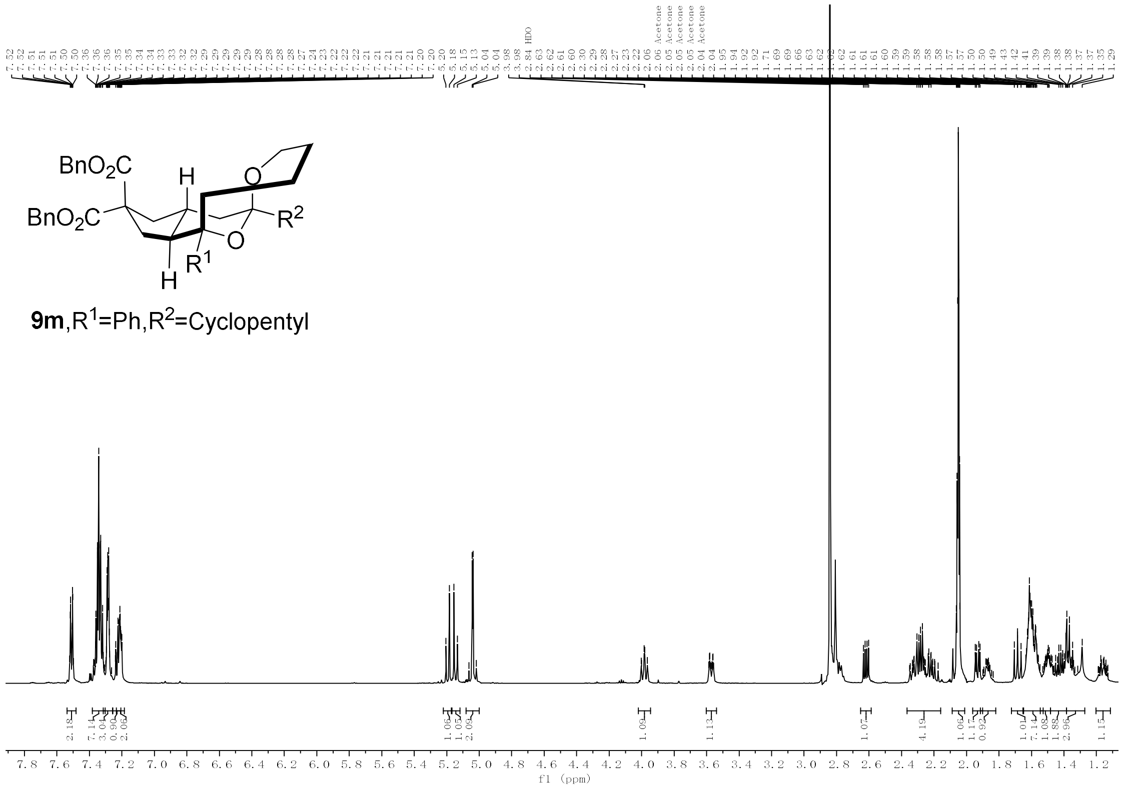

Supplementary Figure 200. ^{13}C NMR spectrum of 9m.

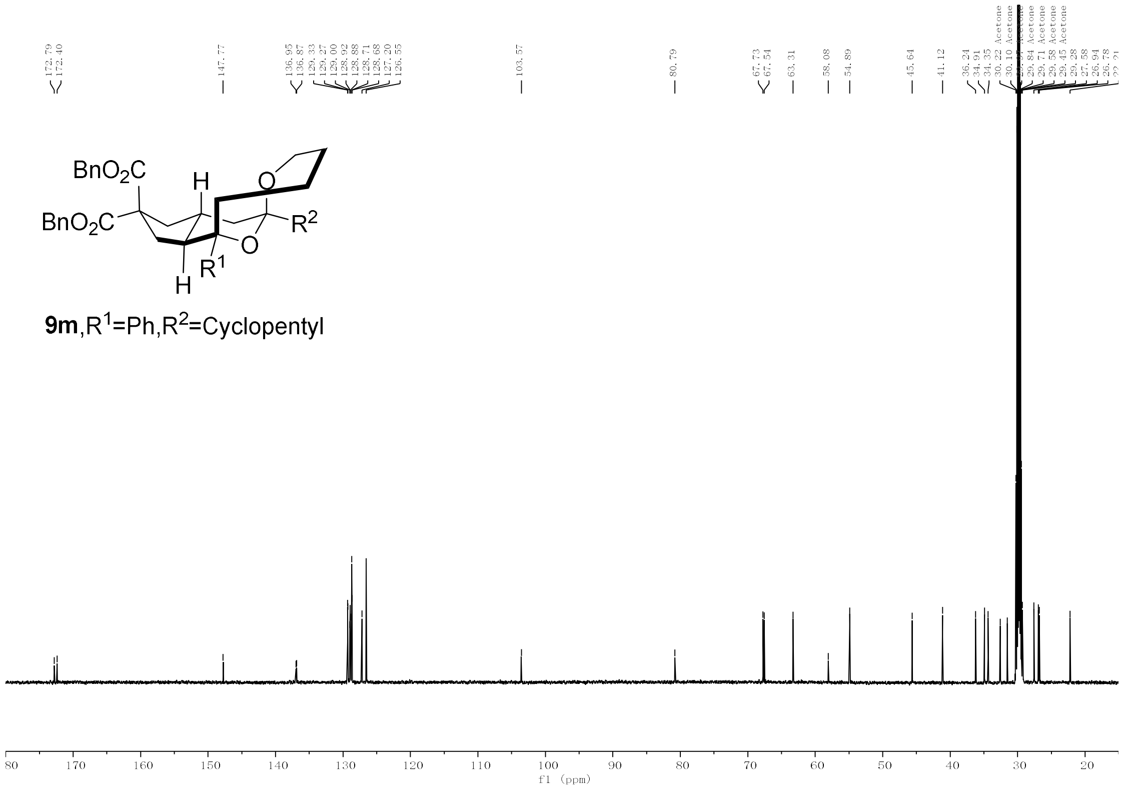

Supplementary Figure 201. [1] H NMR spectrum of 9n.

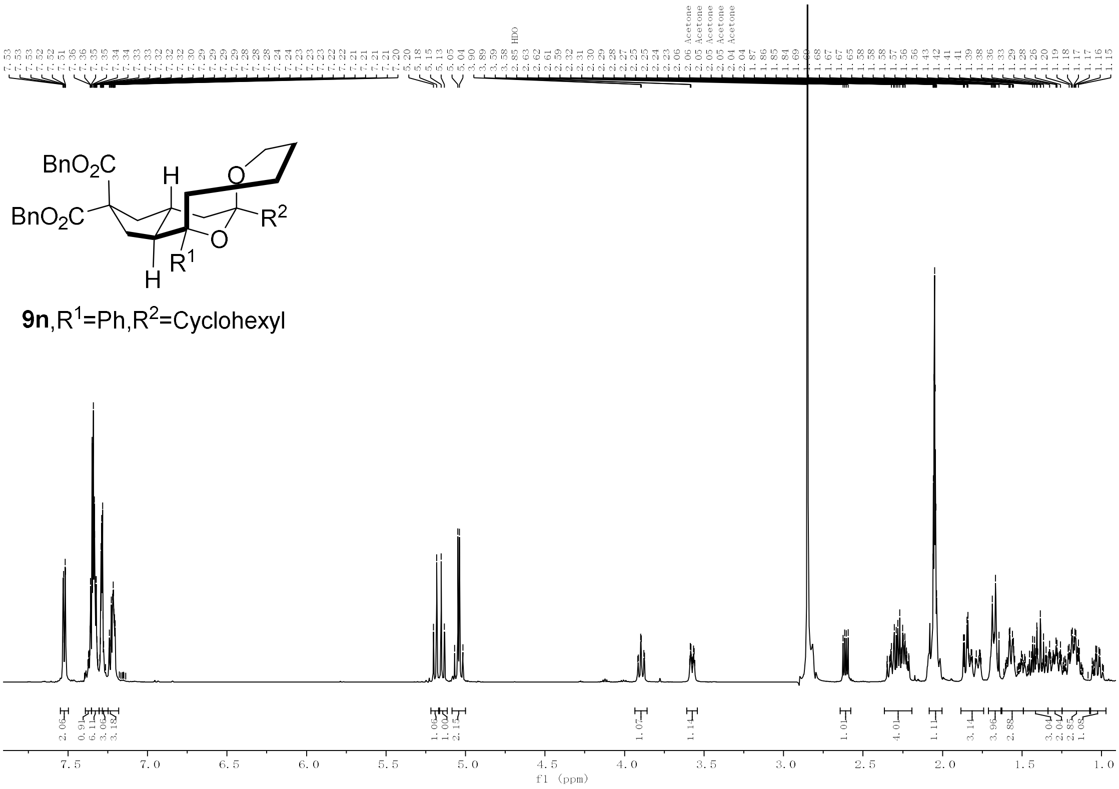

Supplementary Figure 202. [13] C NMR spectrum of 9n.

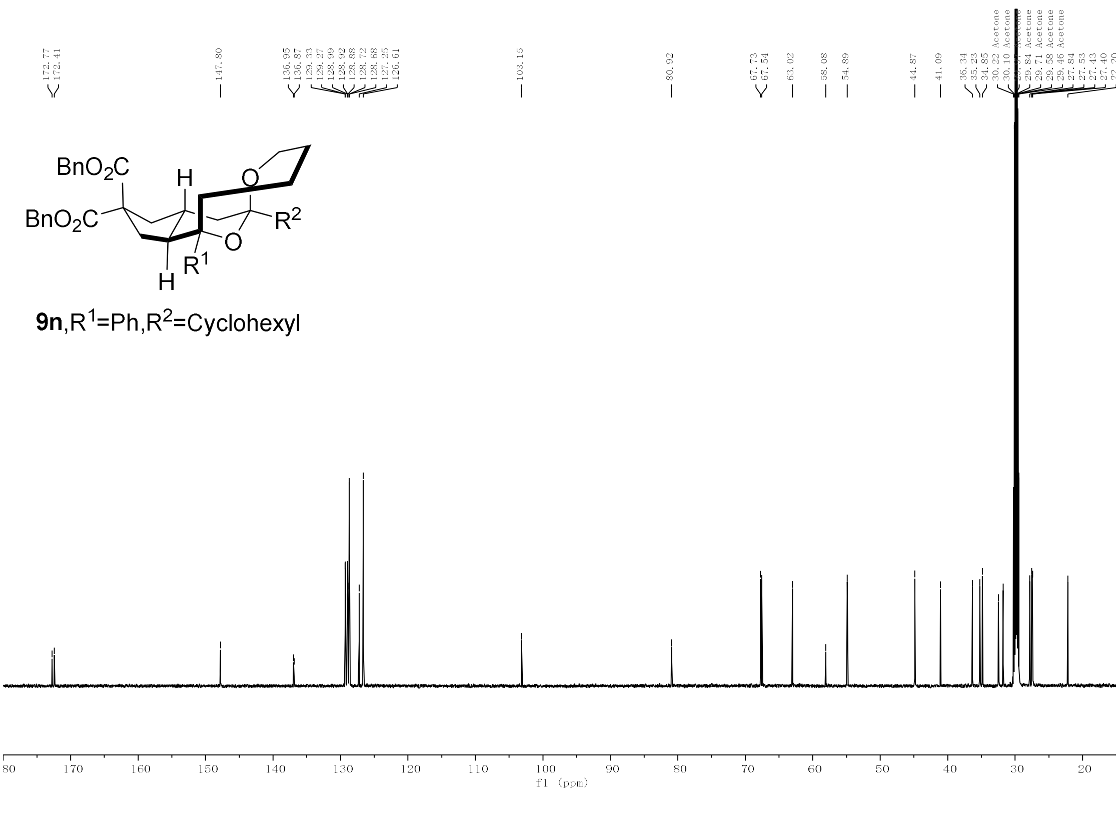

Supplementary Figure 203. [1] H NMR spectrum of 10a.

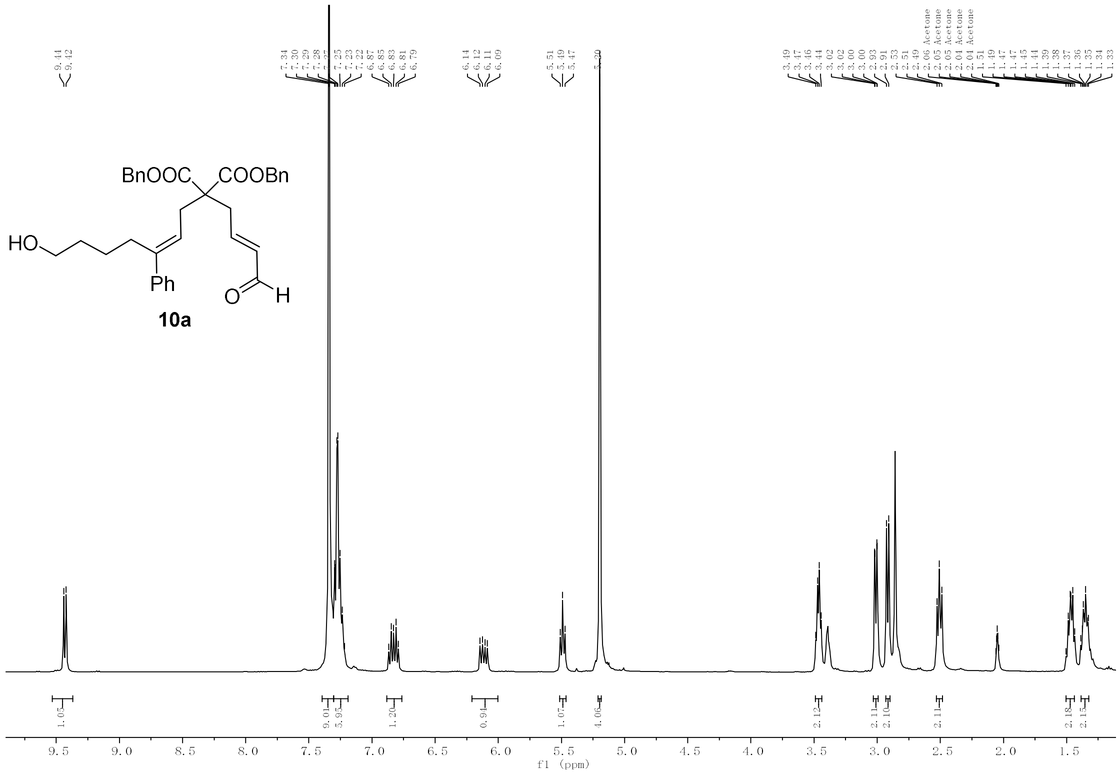

Supplementary Figure 204. [13] C NMR spectrum of 10a.

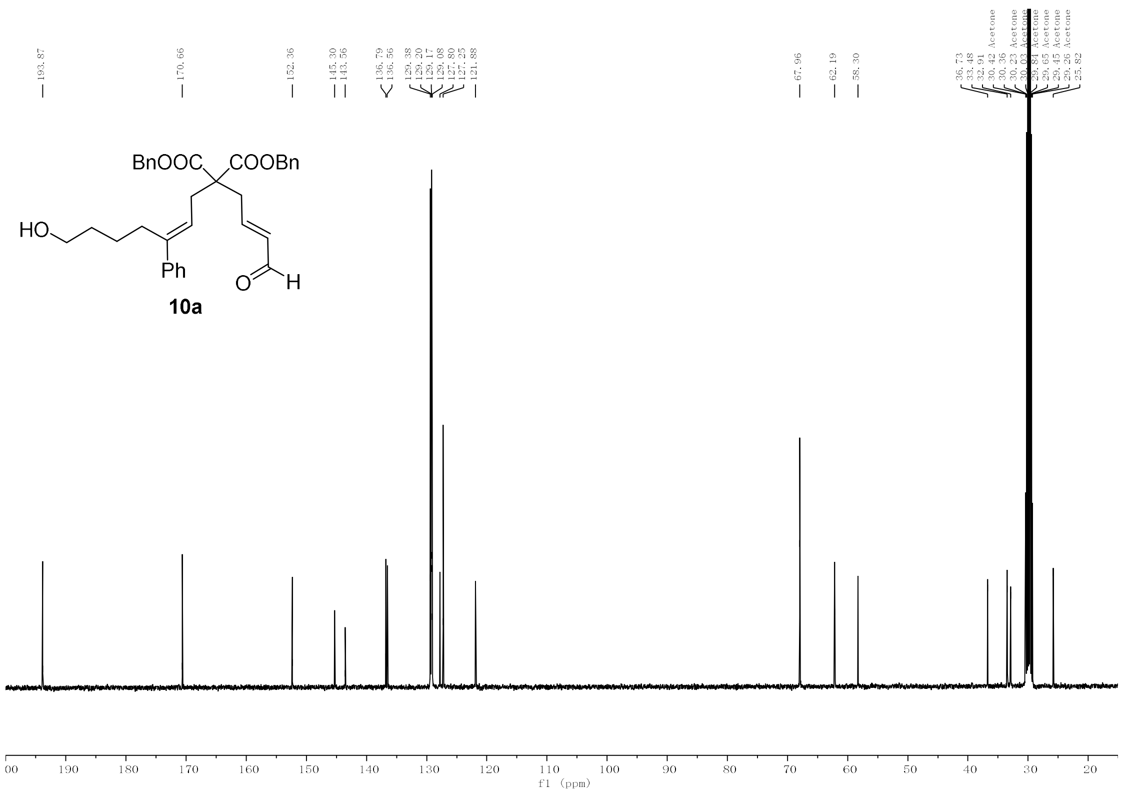

Supplementary Figure 205. ^{1}H NMR spectrum of 10b.

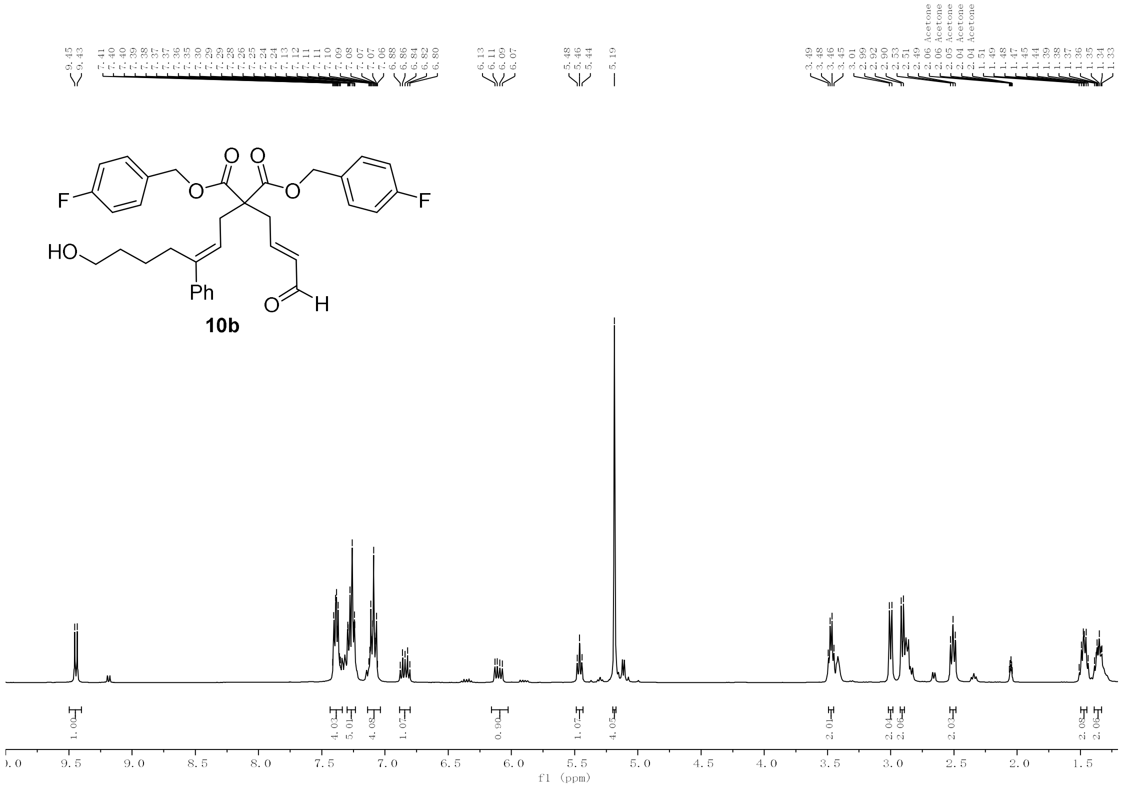

Supplementary Figure 206. ^{13}C NMR spectrum of 10b.

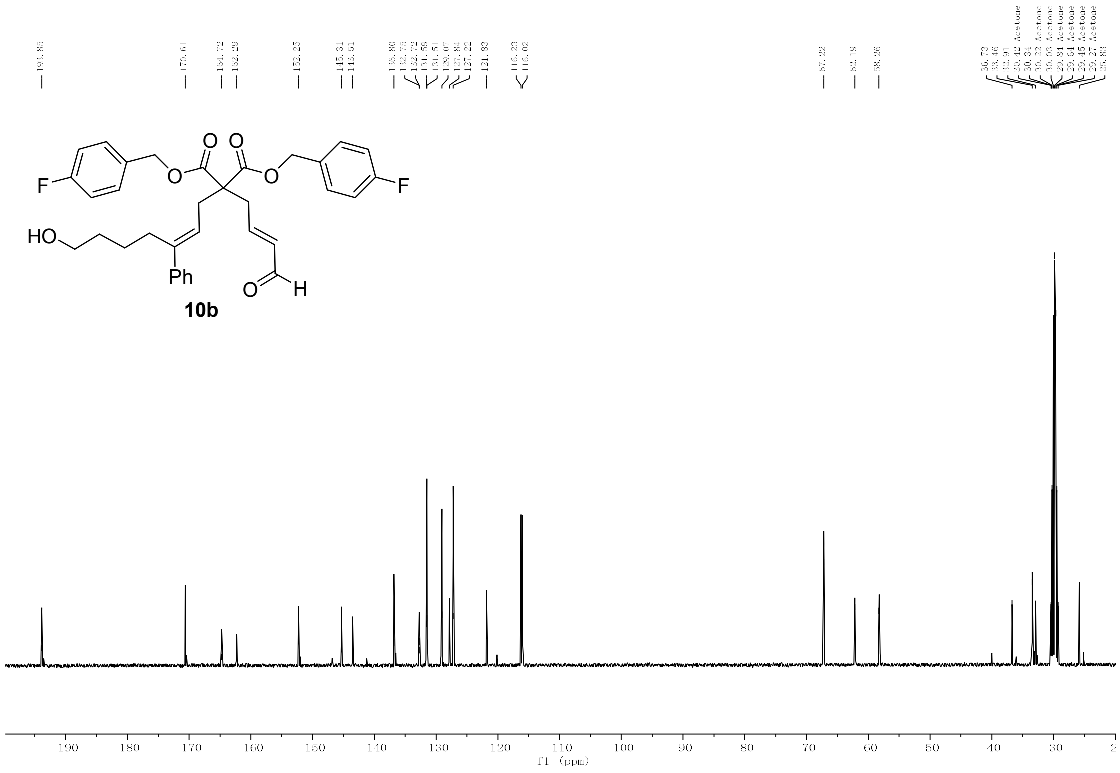

Supplementary Figure 207. [1] H NMR spectrum of 10c.

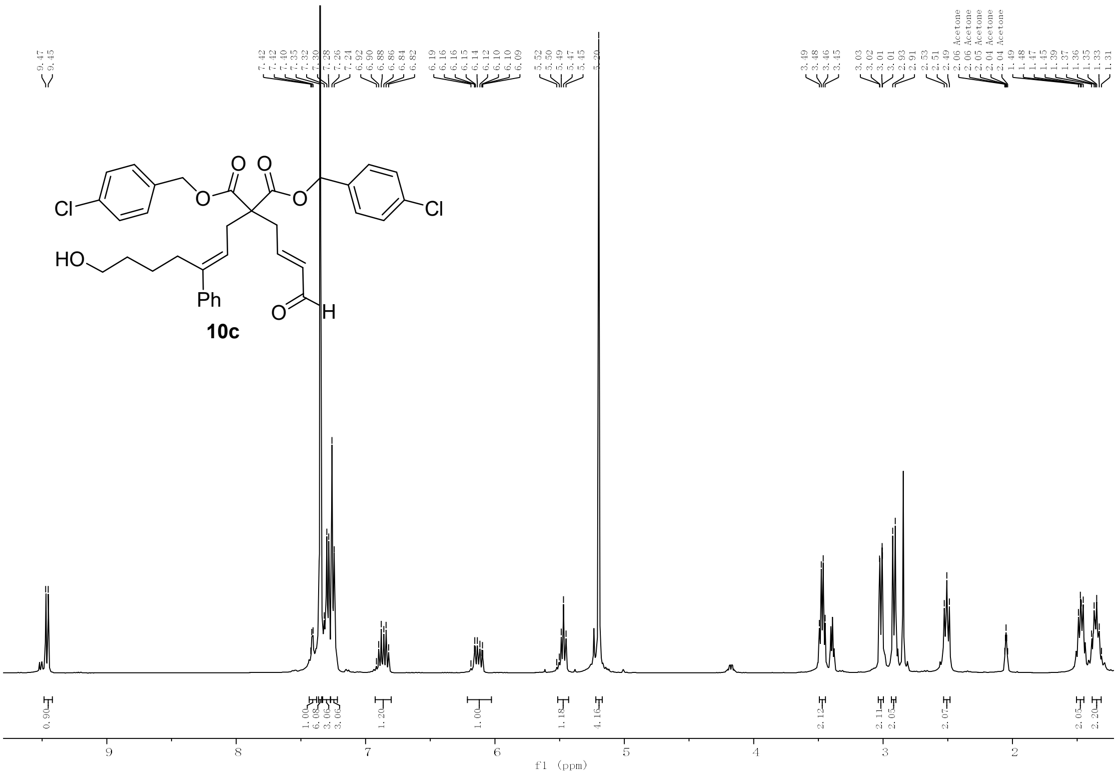

Supplementary Figure 208. [13] C NMR spectrum of 10c.

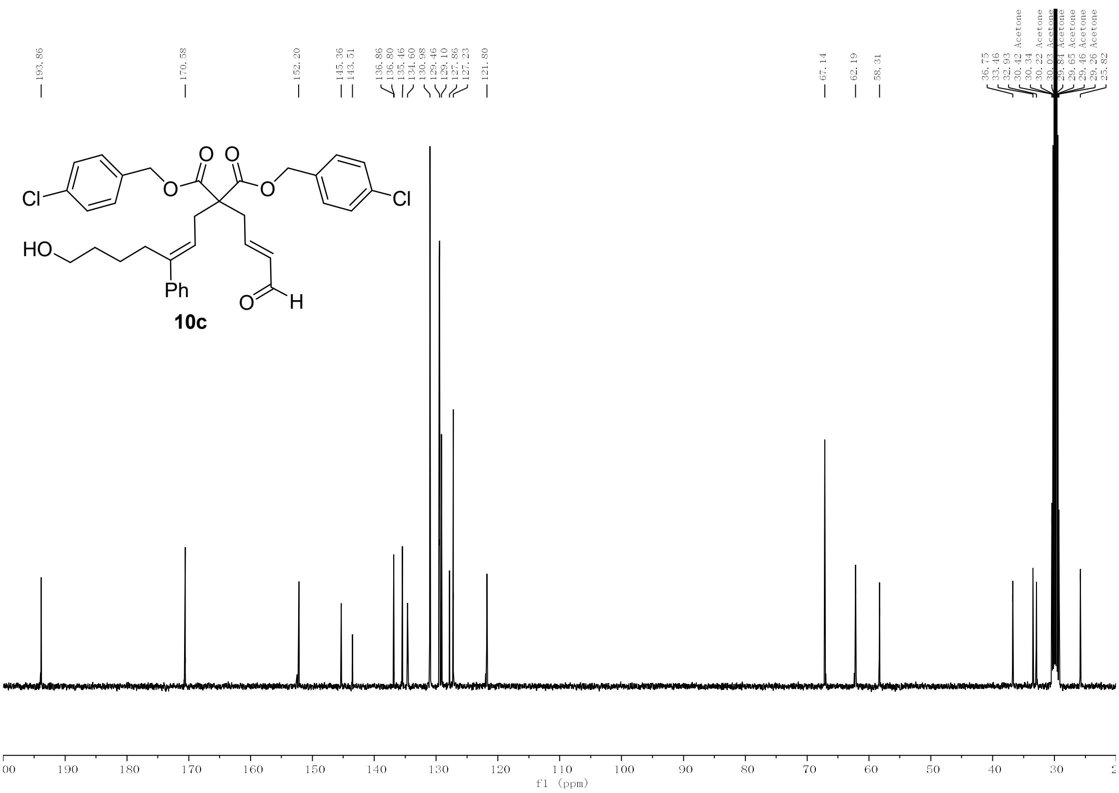

Supplementary Figure 209. [1] H NMR spectrum of 10d.

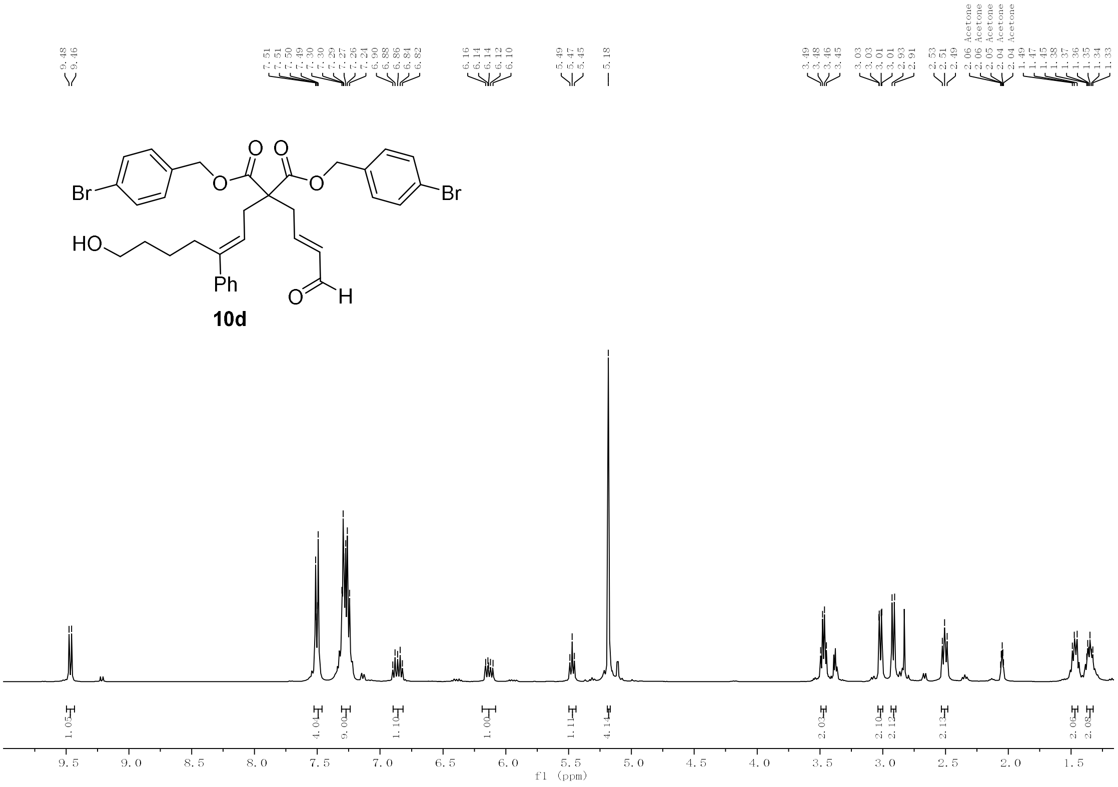

Supplementary Figure 210. [13] C NMR spectrum of 10d.

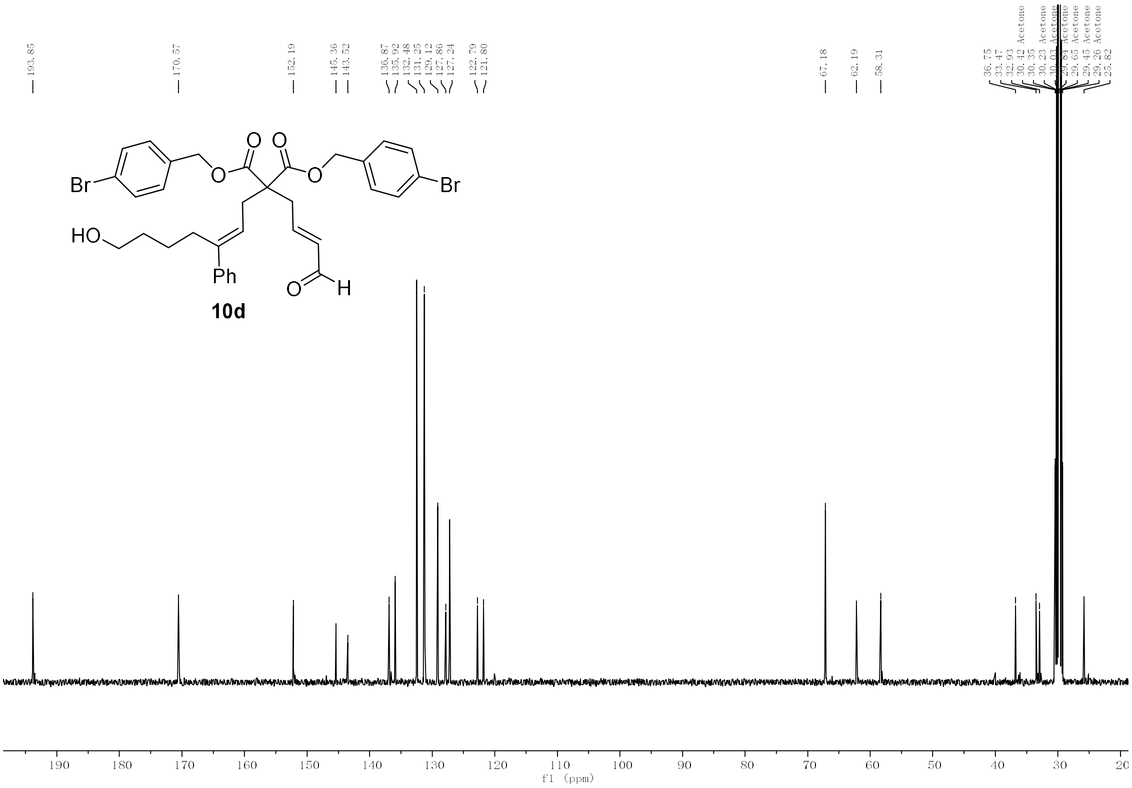

Supplementary Figure 211. [1] H NMR spectrum of 10e.

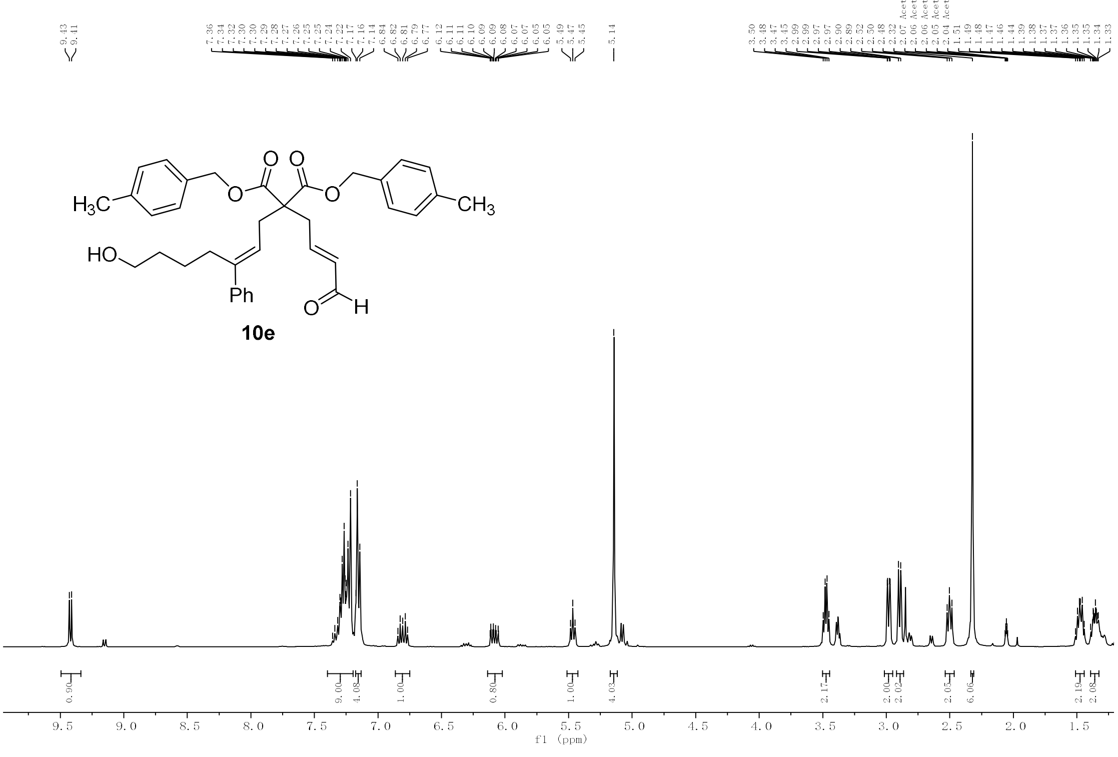

Supplementary Figure 212. [13] C NMR spectrum of 10e.

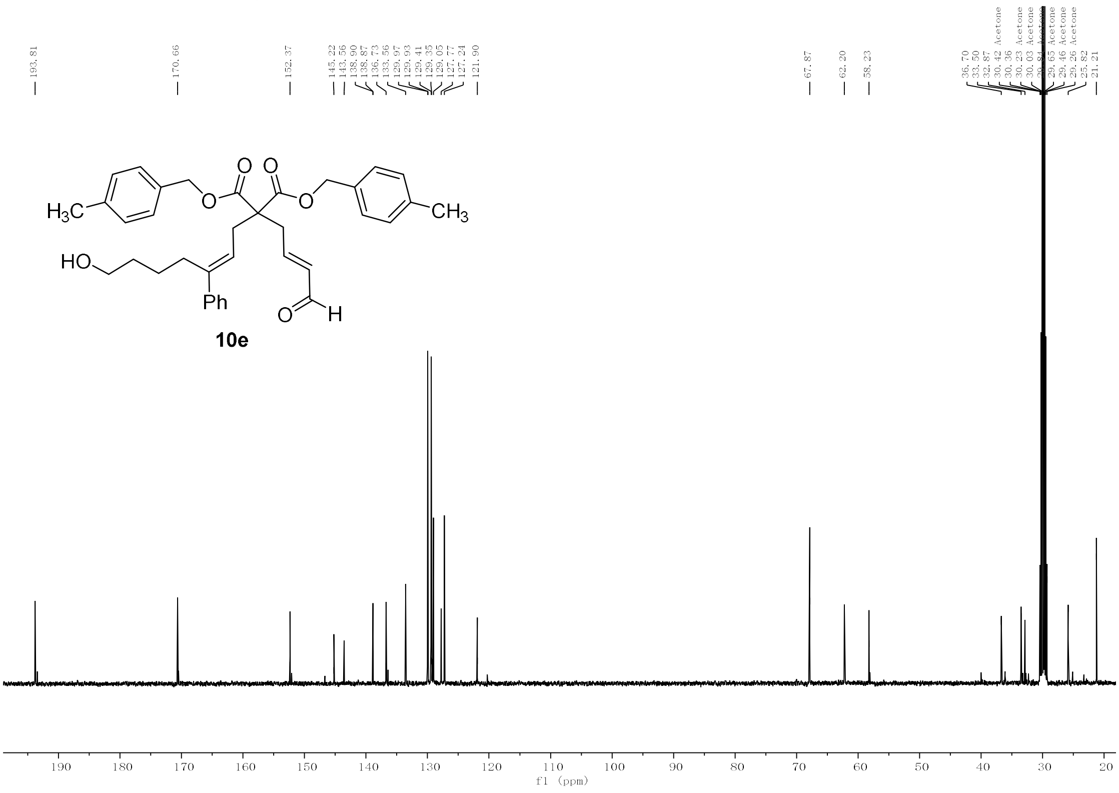

Supplementary Figure 213. ^{1}H NMR spectrum of 10f.

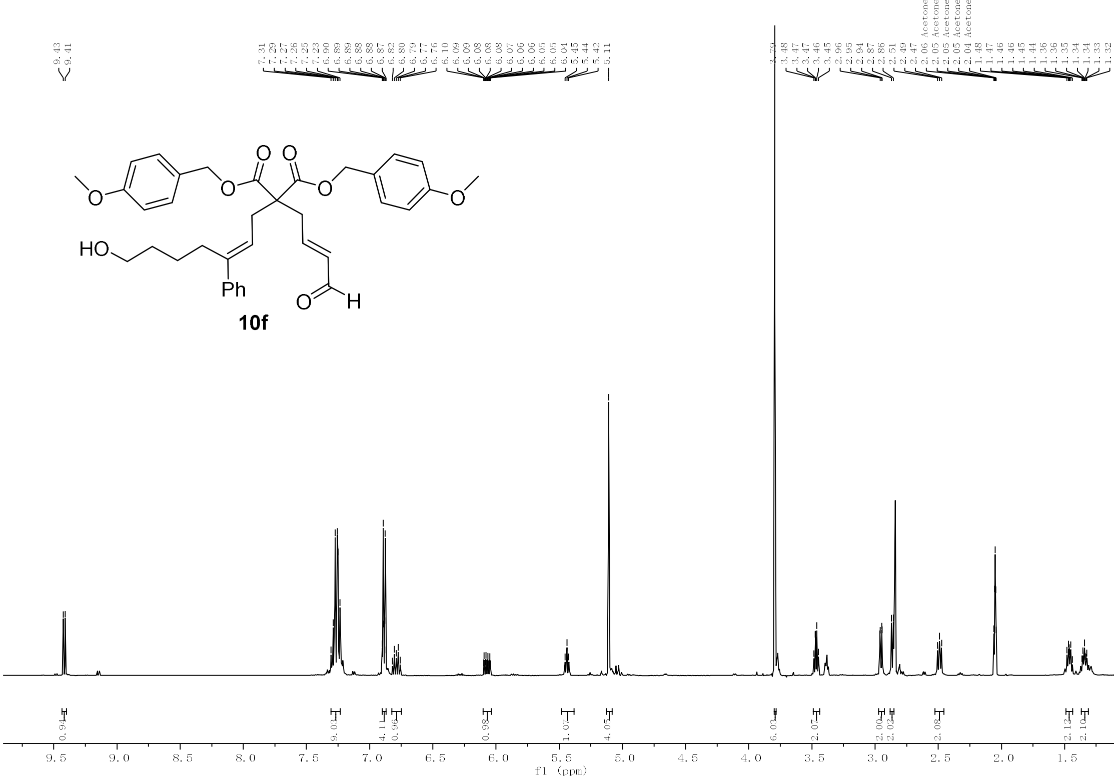

Supplementary Figure 214. ^{13}C NMR spectrum of 10f.

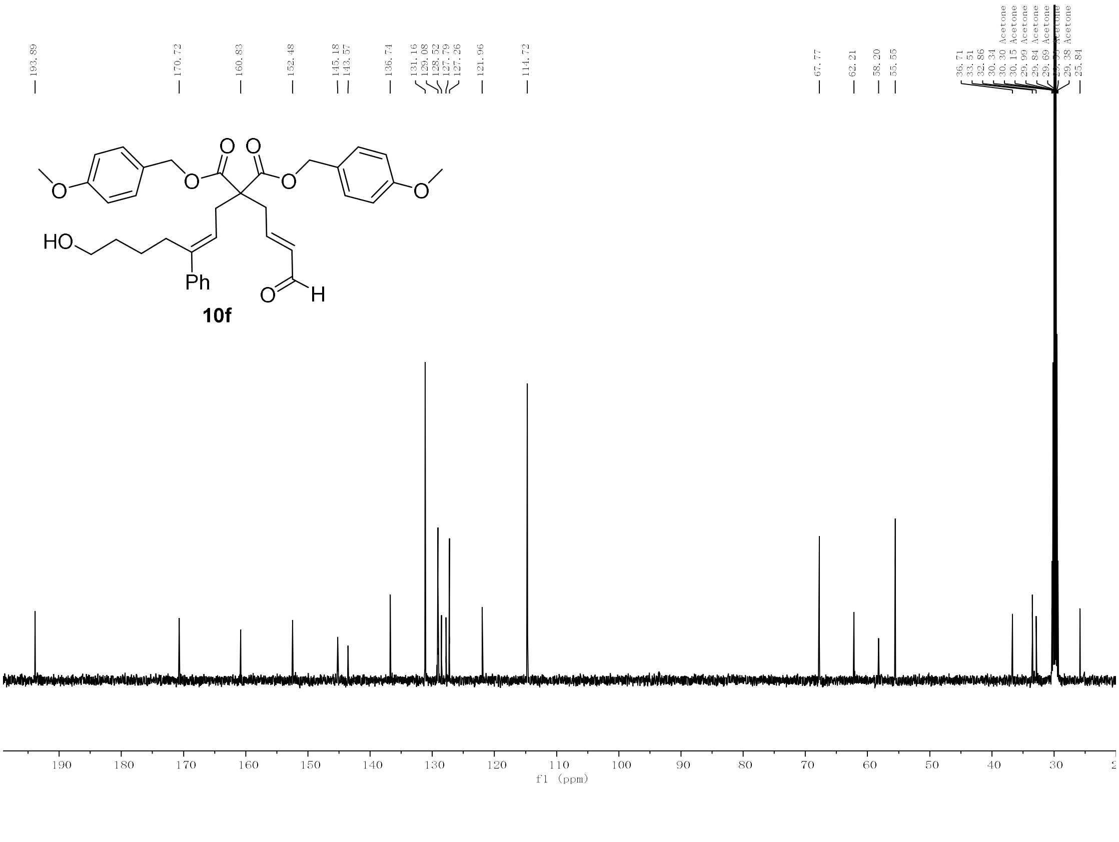

Supplementary Figure 215. ^{1}H NMR spectrum of 10g.

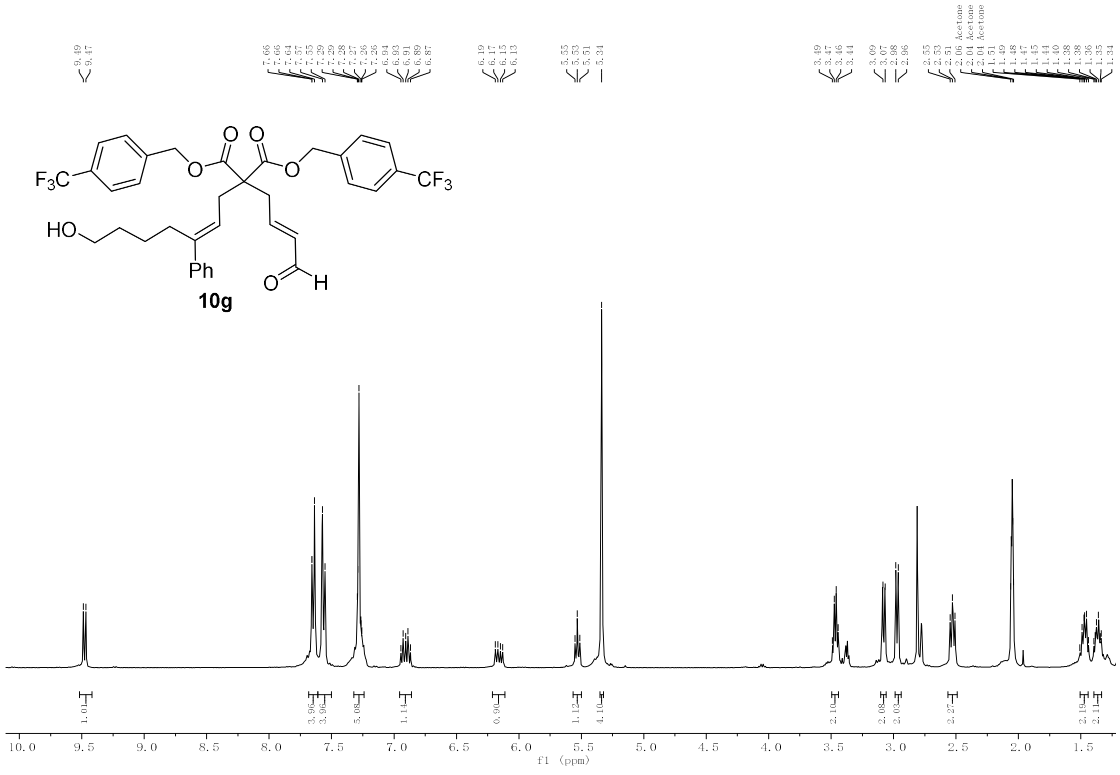

Supplementary Figure 216. ^{13}C NMR spectrum of 10g.

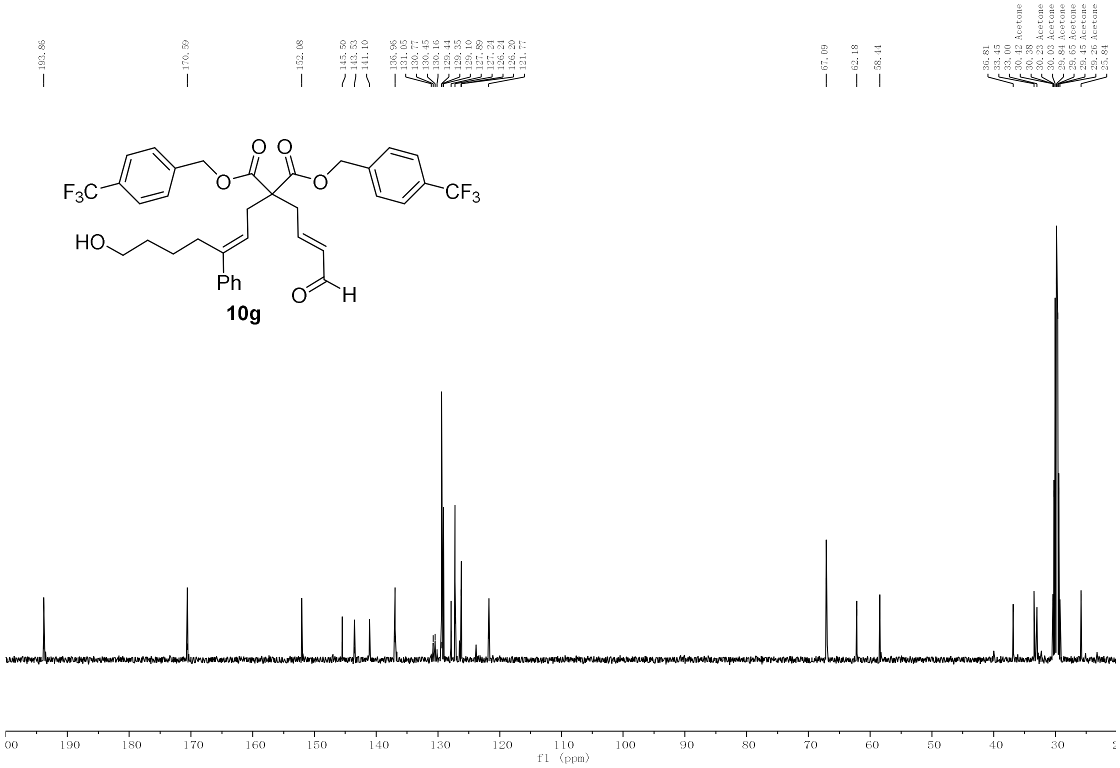

Supplementary Figure 217. [1] H NMR spectrum of 10h.

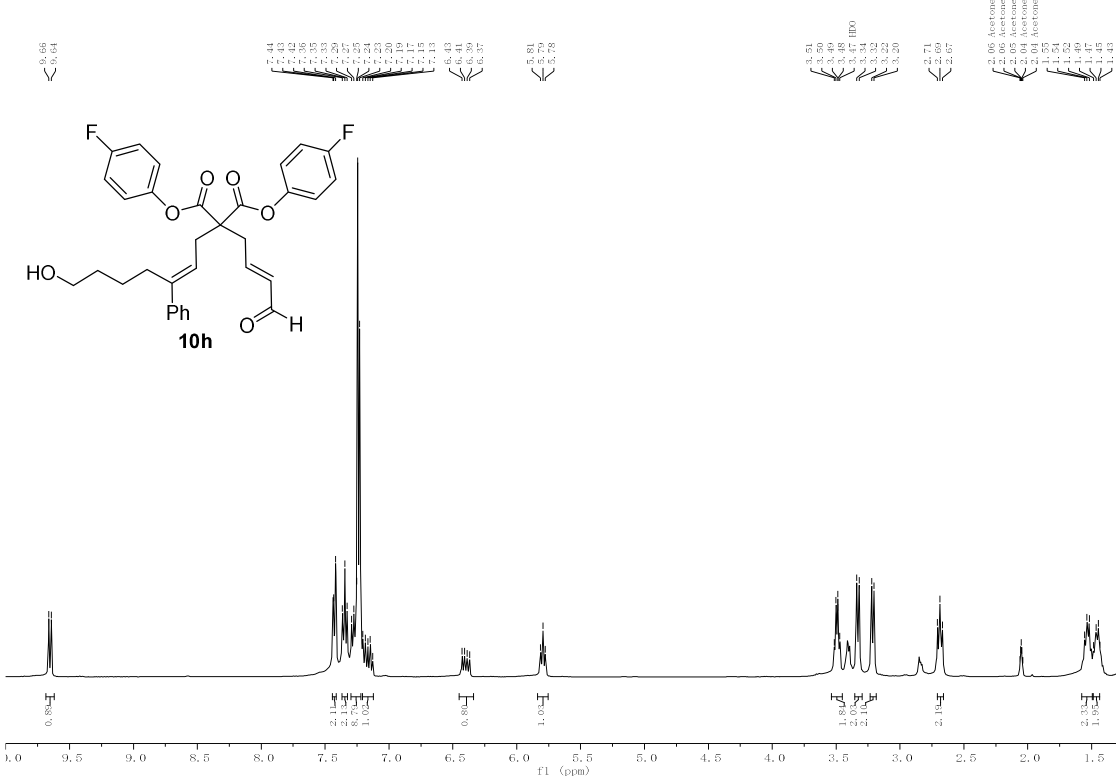

Supplementary Figure 218. [13] C NMR spectrum of 10h.

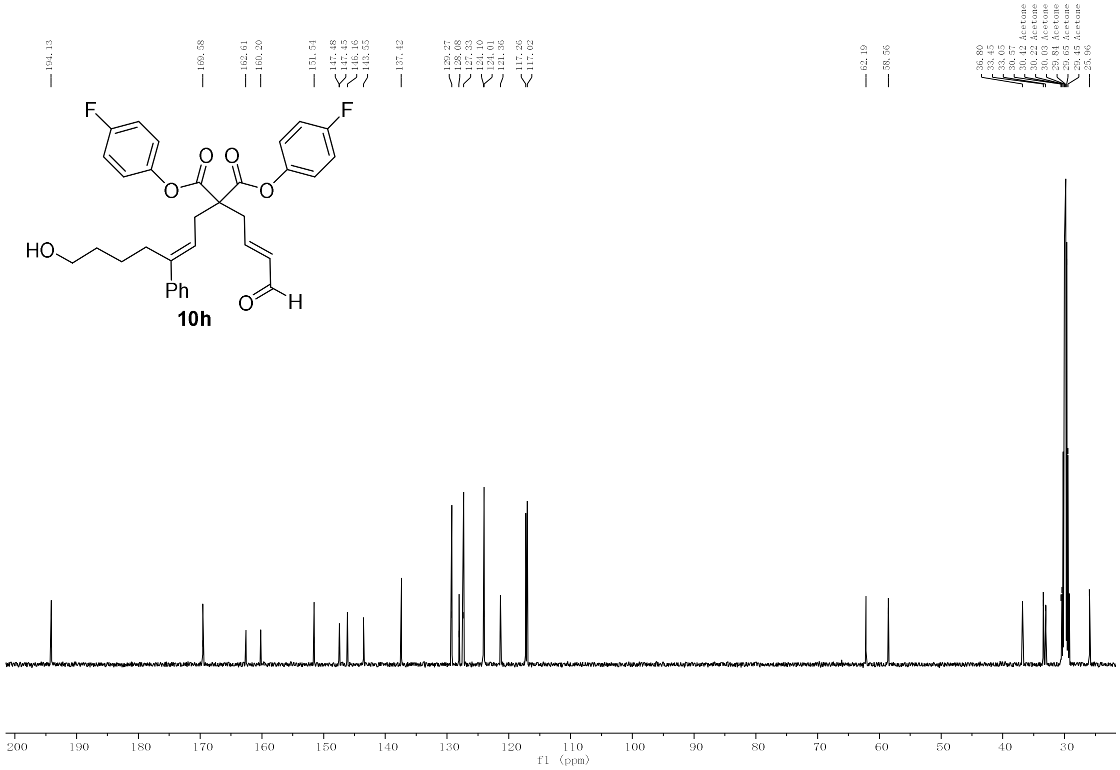

Supplementary Figure 219. ^{1}H NMR spectrum of 10i.

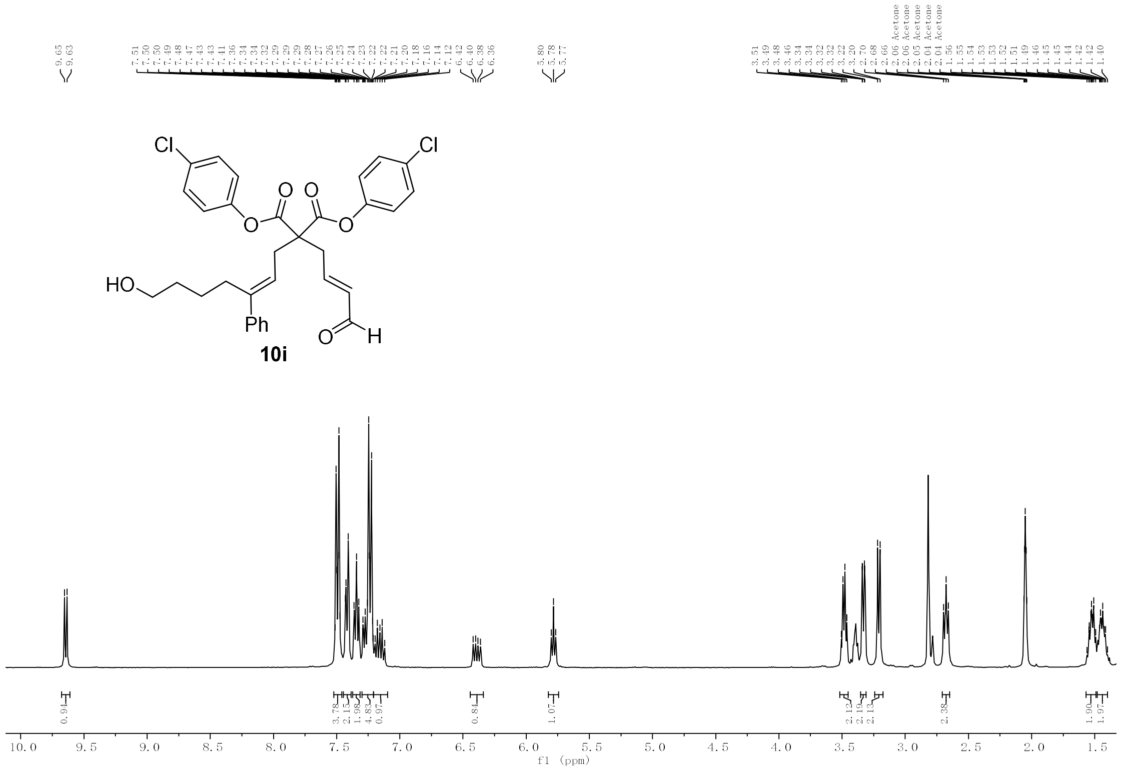

Supplementary Figure 220. ^{13}C NMR spectrum of 10i.

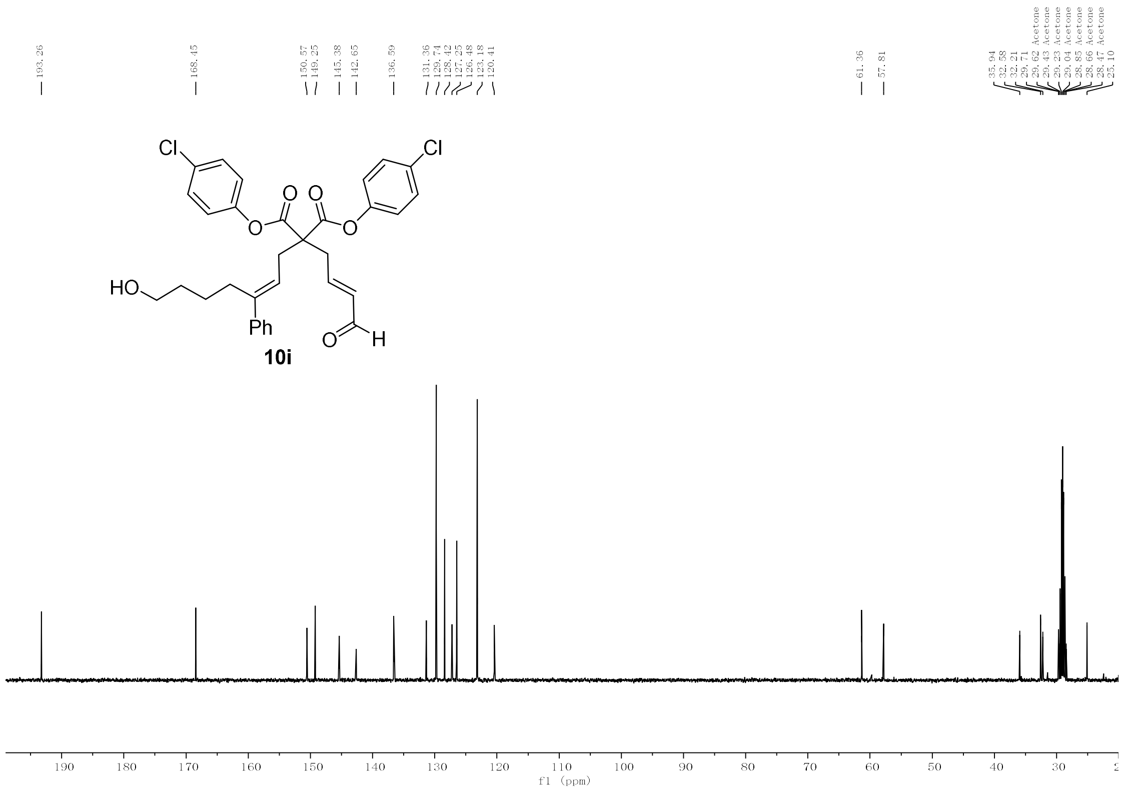

Supplementary Figure 221. ^{1}H NMR spectrum of 10j.

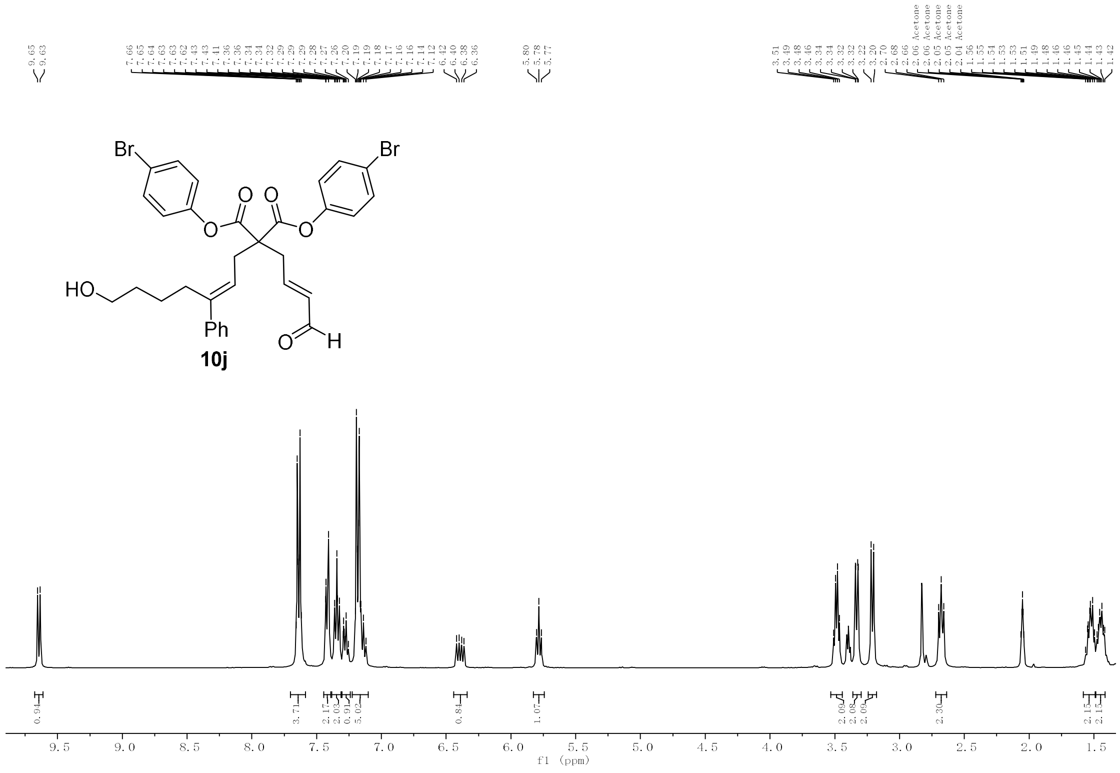

Supplementary Figure 222. ^{13}C NMR spectrum of 10j.

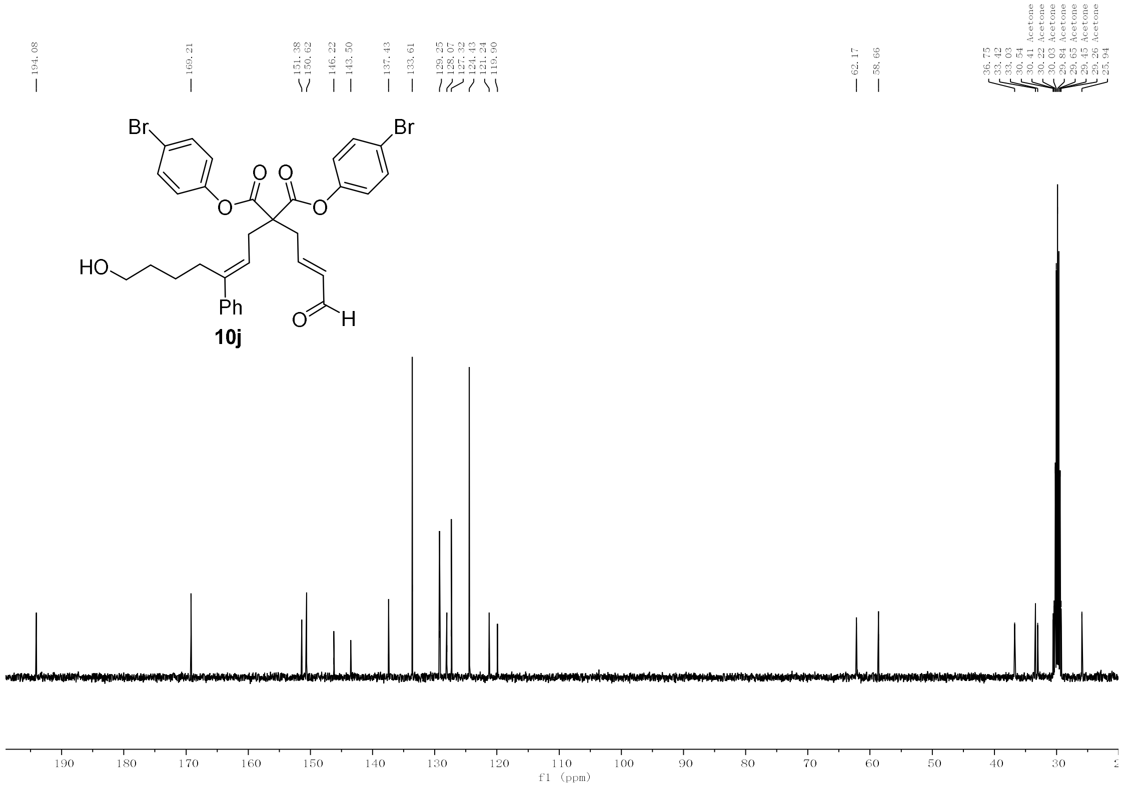

Supplementary Figure 223. ¹ H NMR spectrum of 10k.

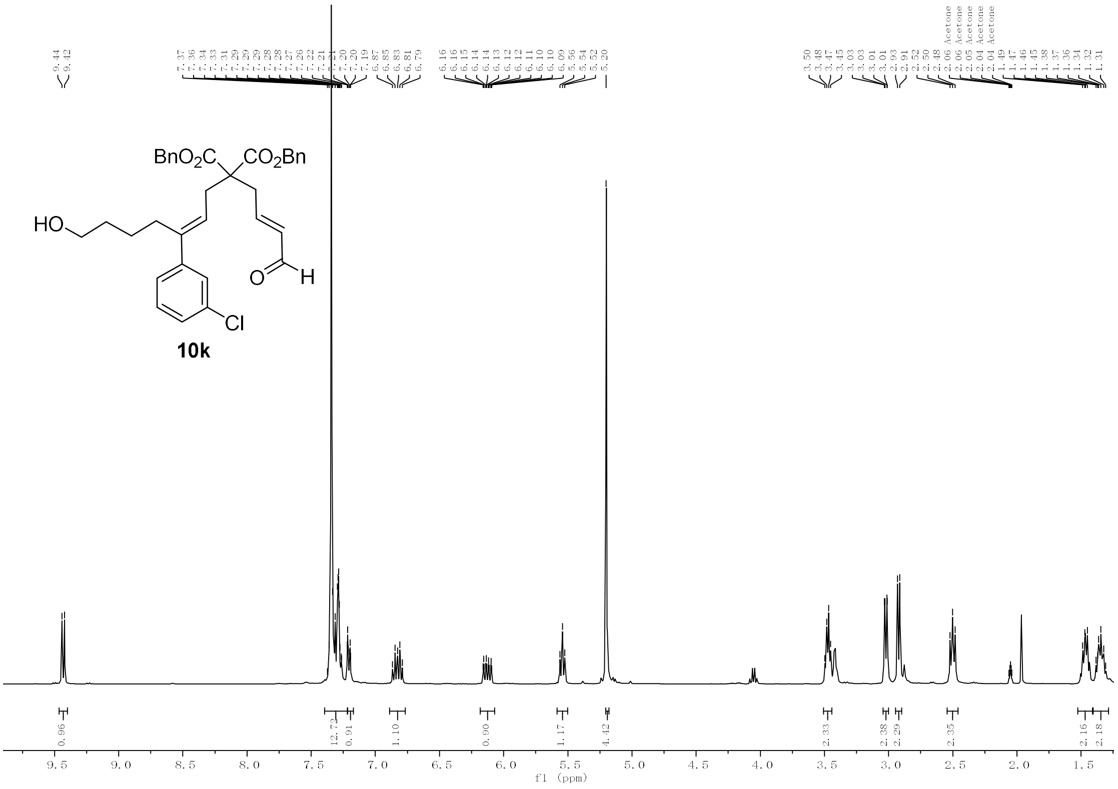

Supplementary Figure 224. ¹³ C NMR spectrum of 10k.

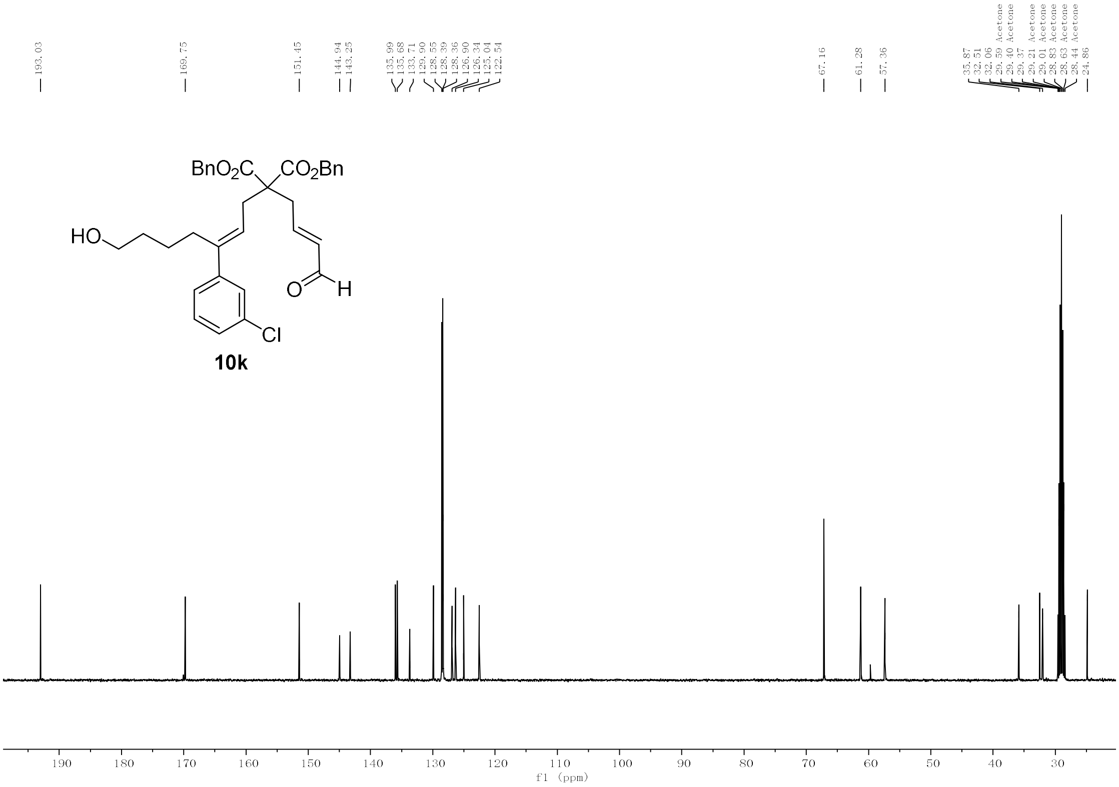

Supplementary Figure 225. [1] H NMR spectrum of 10l.

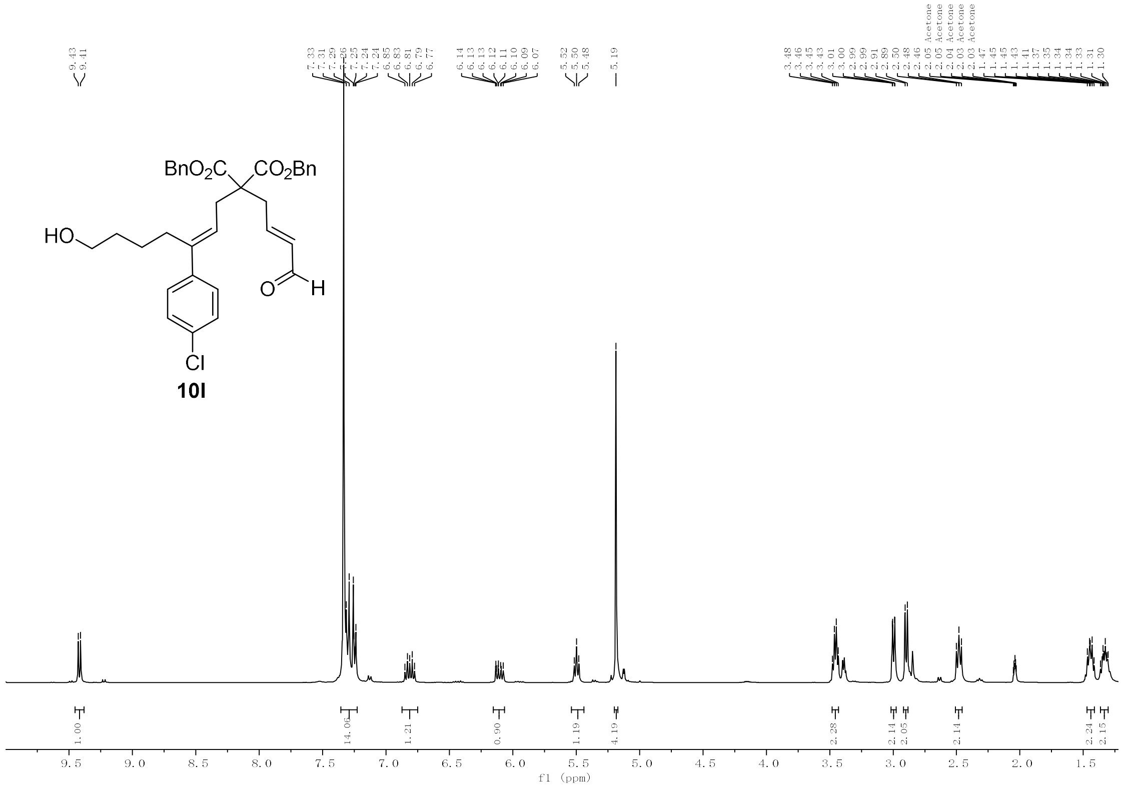

Supplementary Figure 226. [13] C NMR spectrum of 10l.

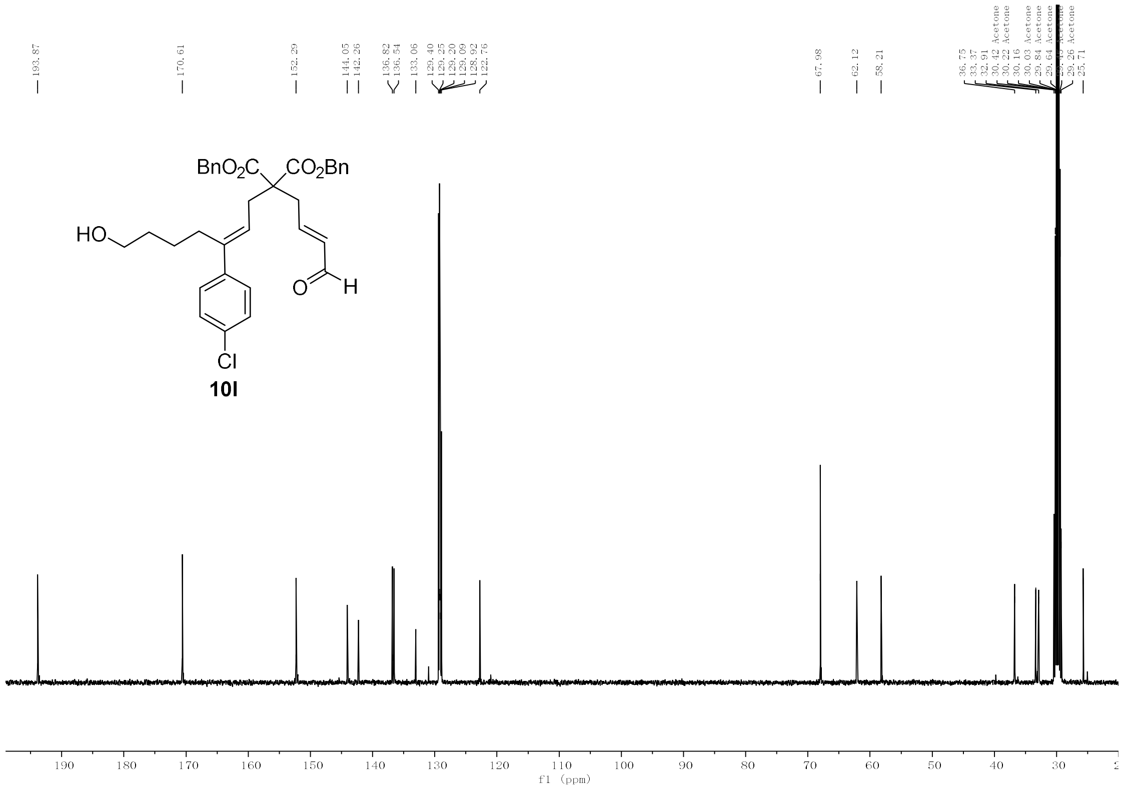

Supplementary Figure 227. [1] H NMR spectrum of 10m.

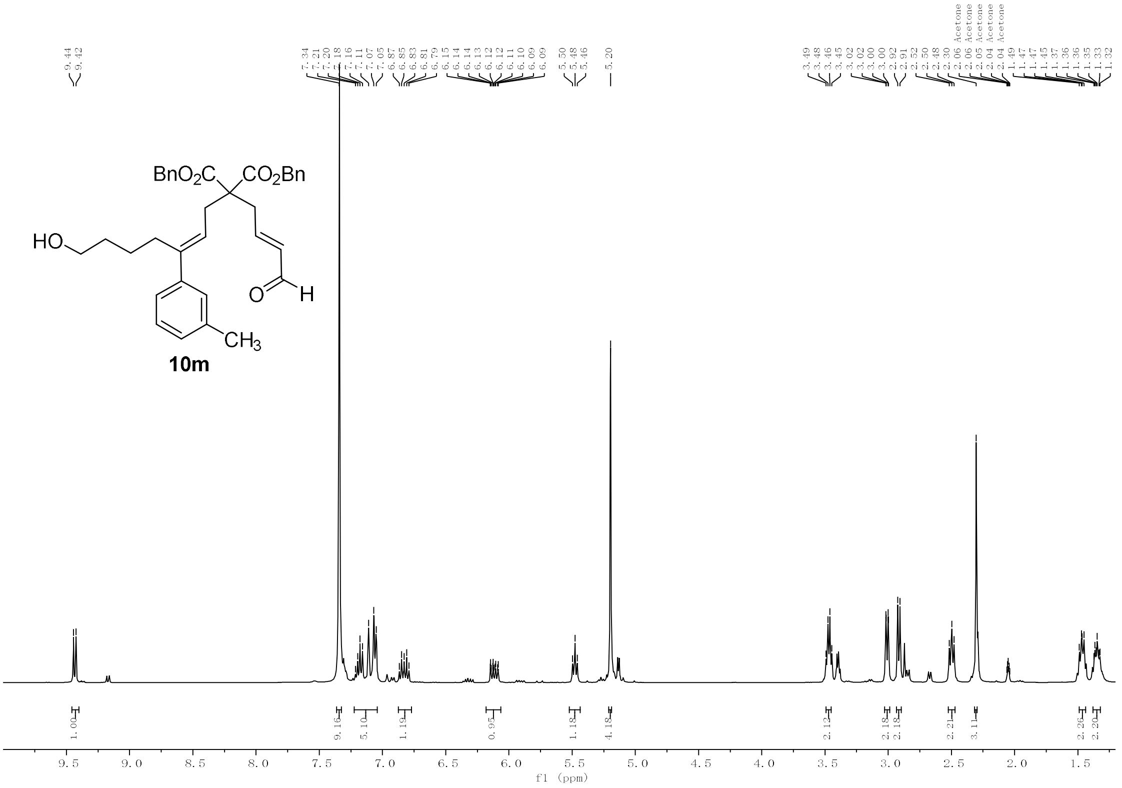

Supplementary Figure 228. [13] C NMR spectrum of 10m.

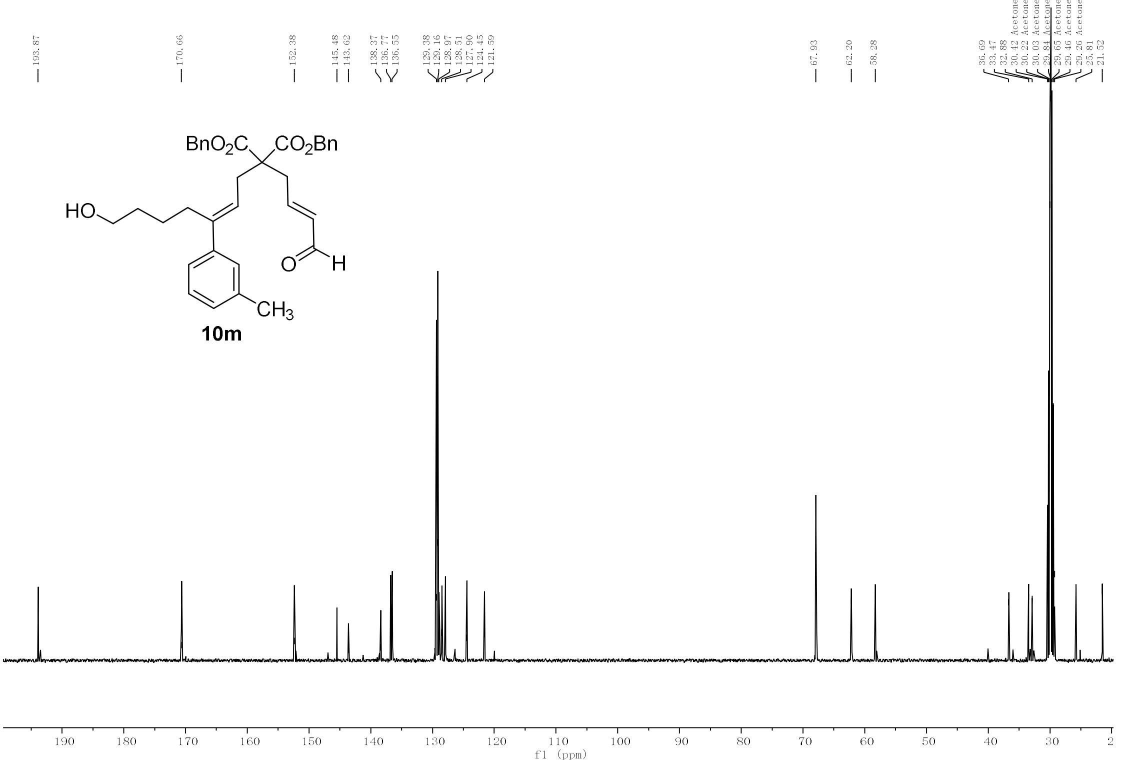

Supplementary Figure 229. ^{1}H NMR spectrum of 10n.

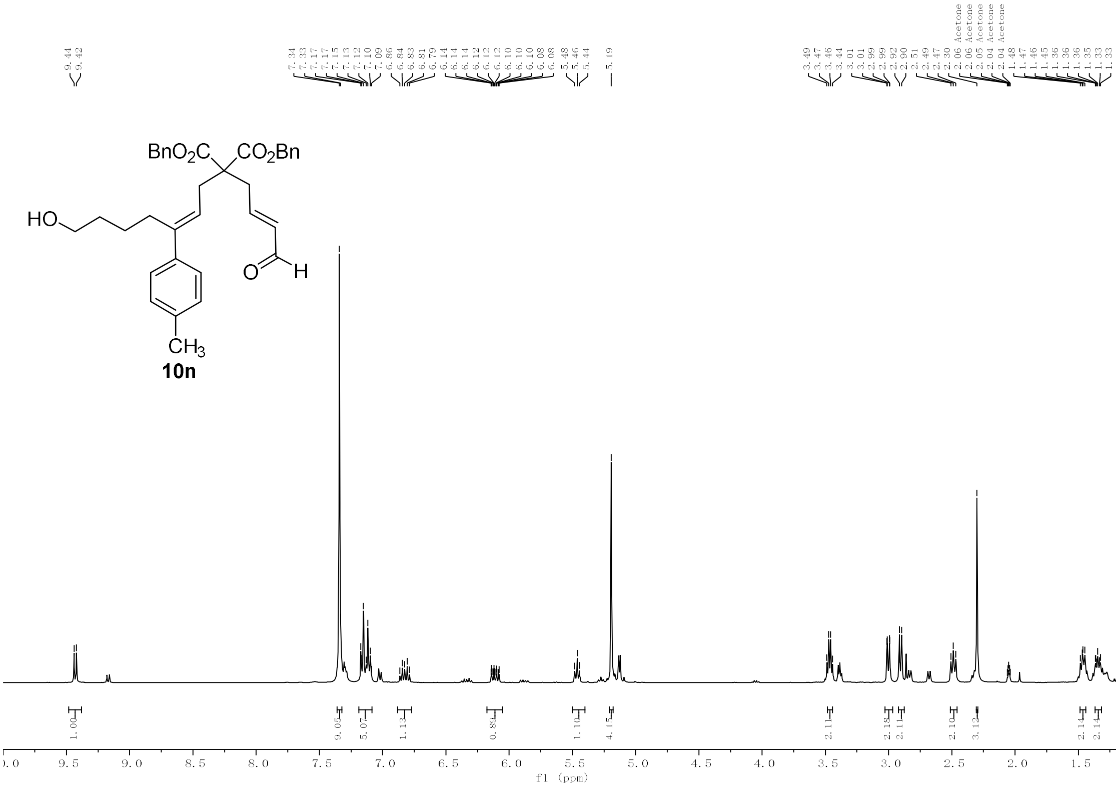

Supplementary Figure 230. ^{13}C NMR spectrum of 10n.

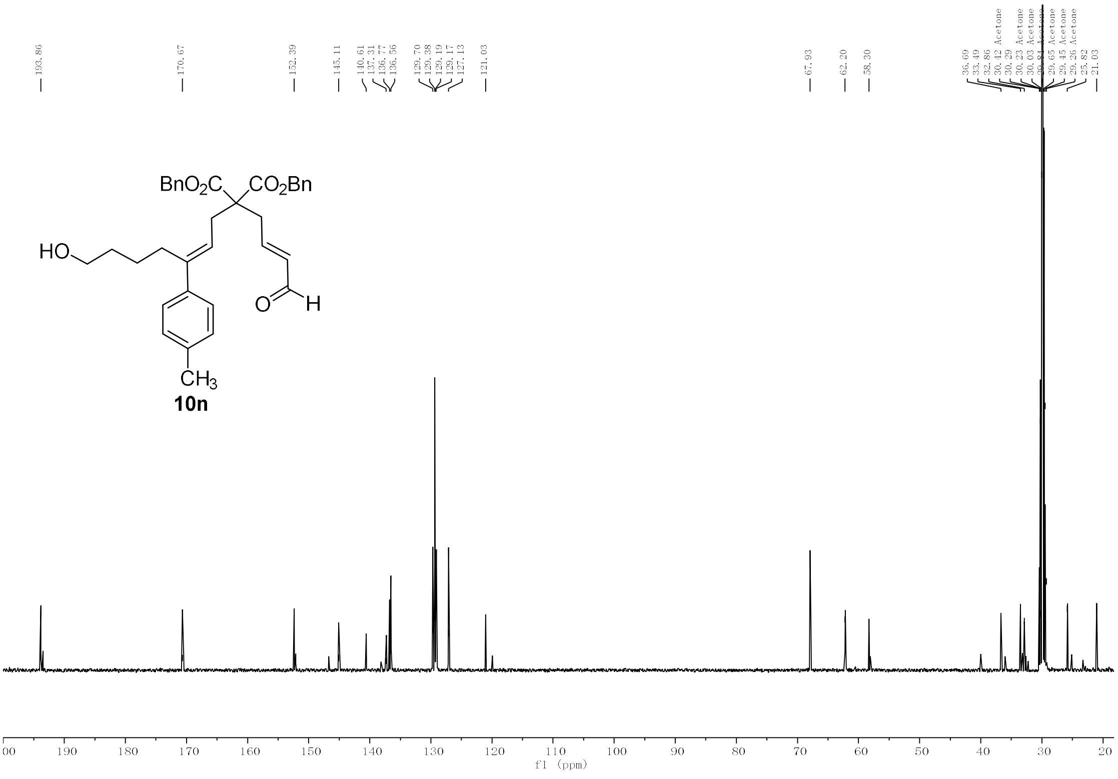

Supplementary Figure 231. [1] H NMR spectrum of 10o.

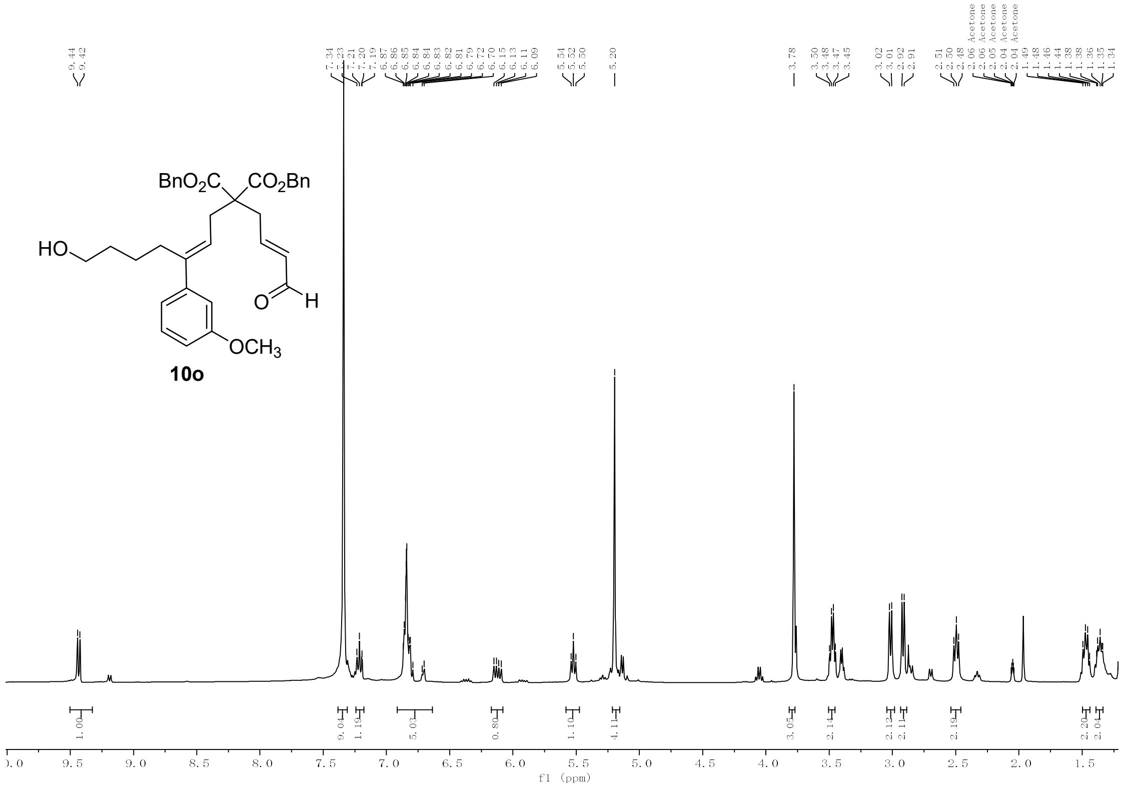

Supplementary Figure 232. [13] C NMR spectrum of 10o.

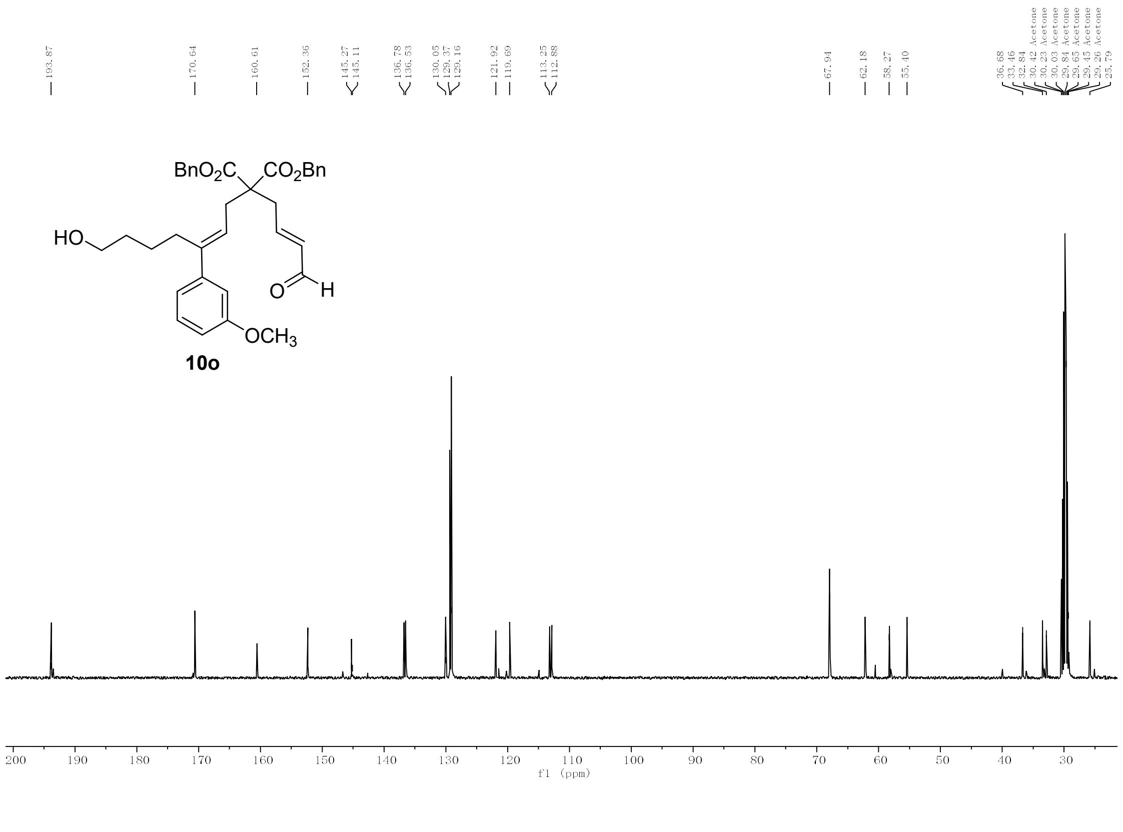

Supplementary Figure 233. [1] H NMR spectrum of 10p.

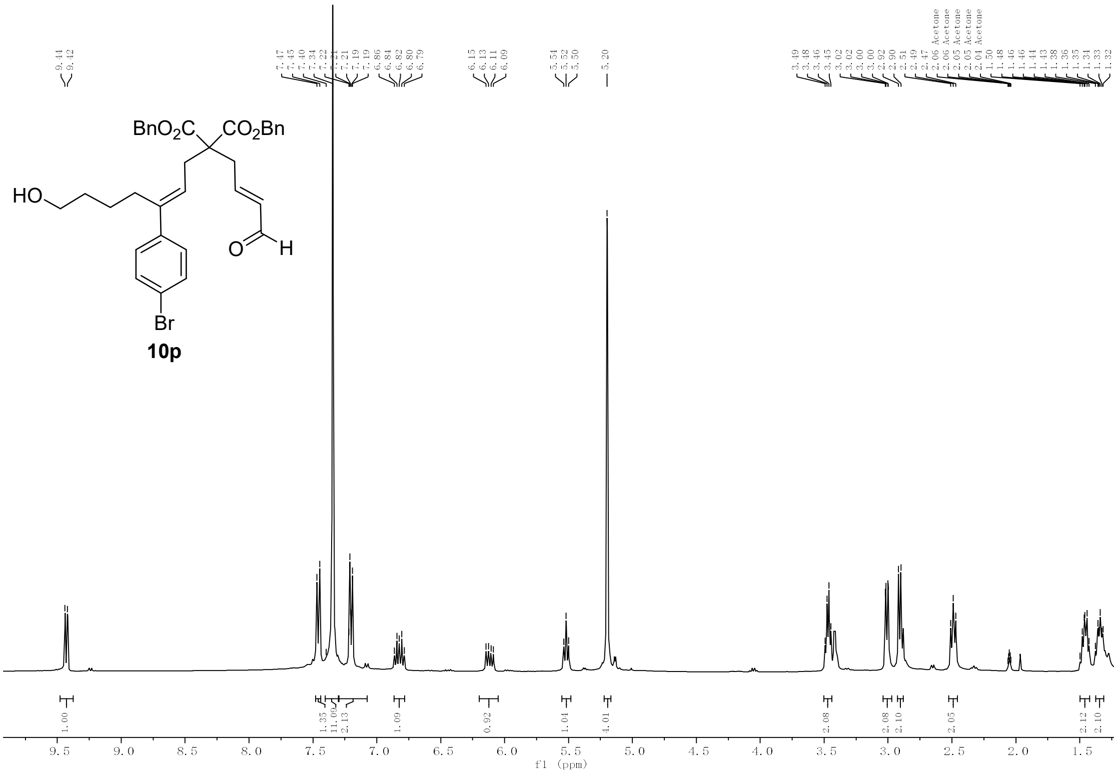

Supplementary Figure 234. [13] C NMR spectrum of 10p.

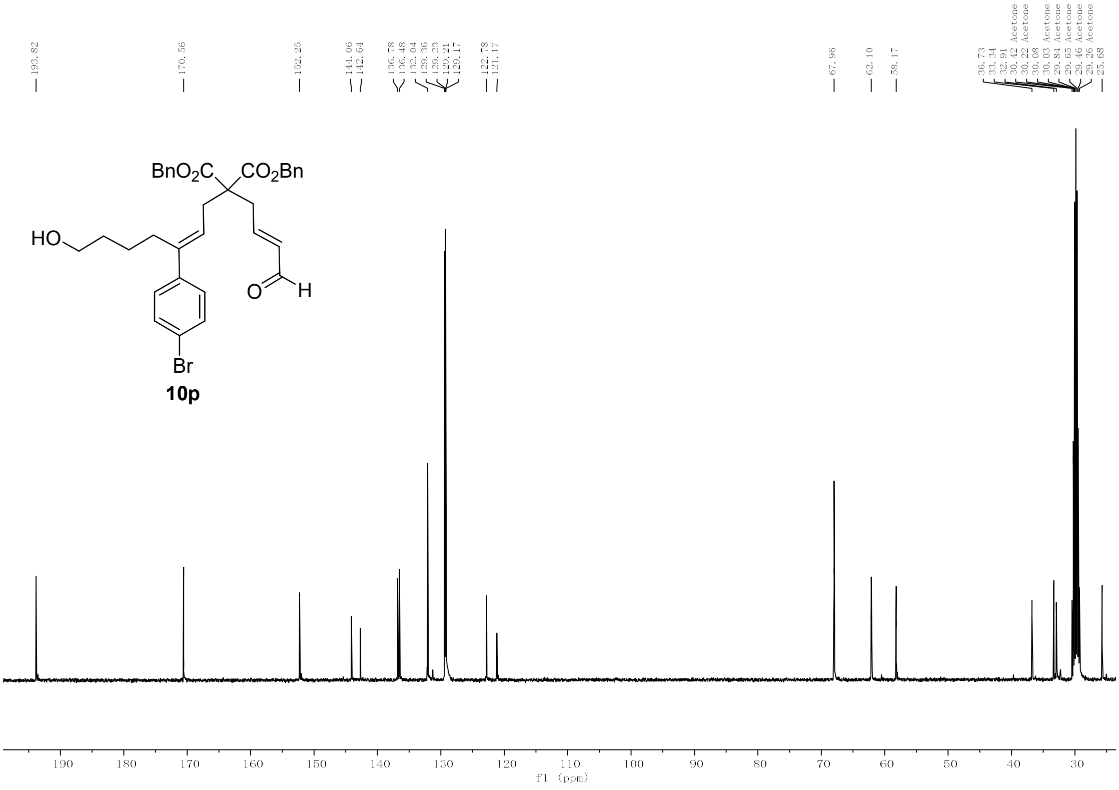

Supplementary Figure 235. [1] H NMR spectrum of 11a.

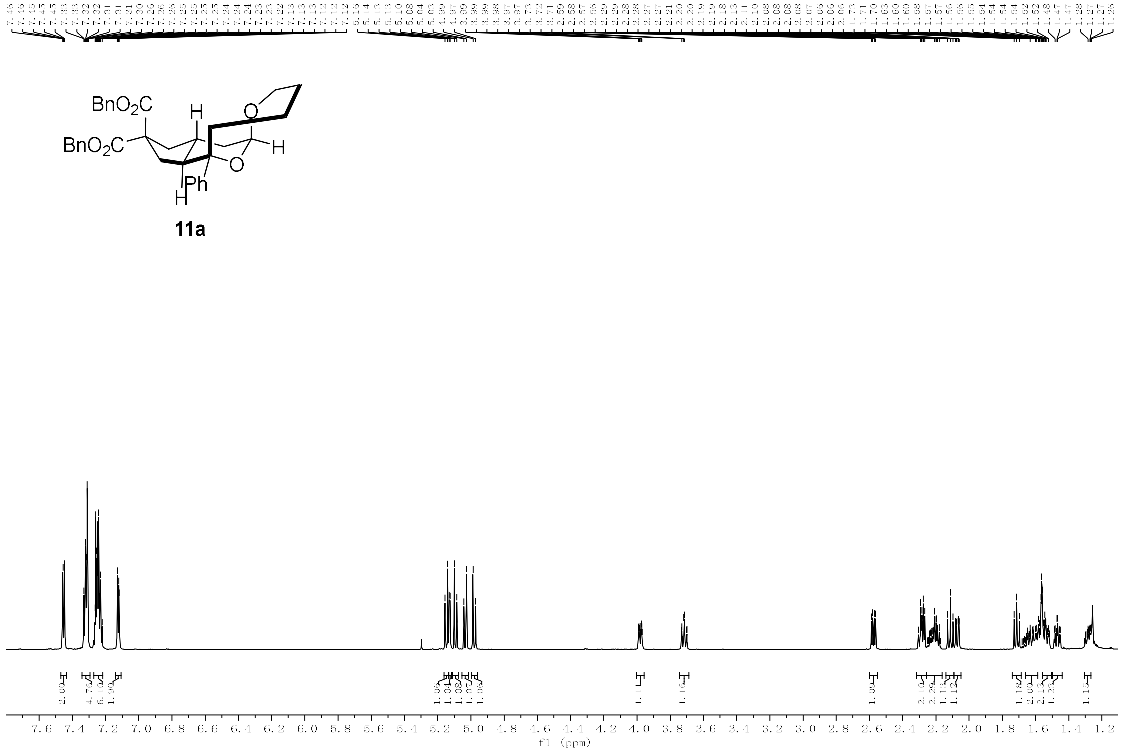

Supplementary Figure 236. [13] C NMR spectrum of 11a.

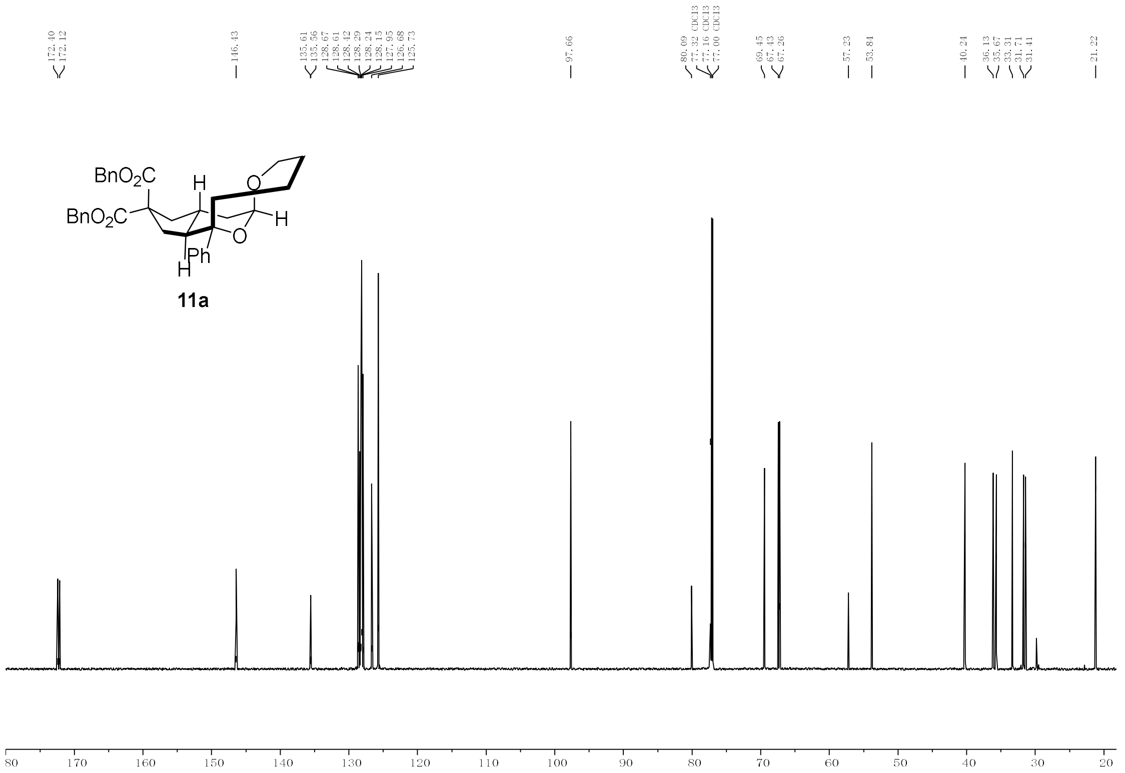

Supplementary Figure 237. ^{1}H NMR spectrum of 11b.

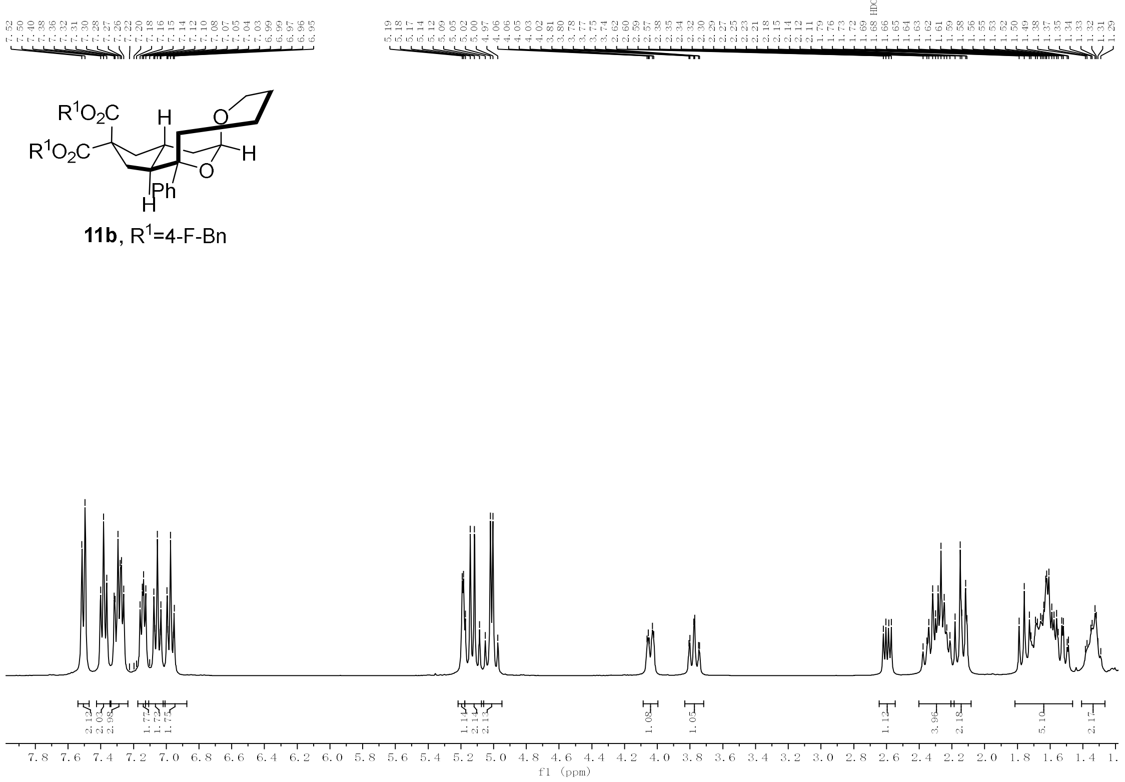

Supplementary Figure 238. ^{13}C NMR spectrum of 11b.

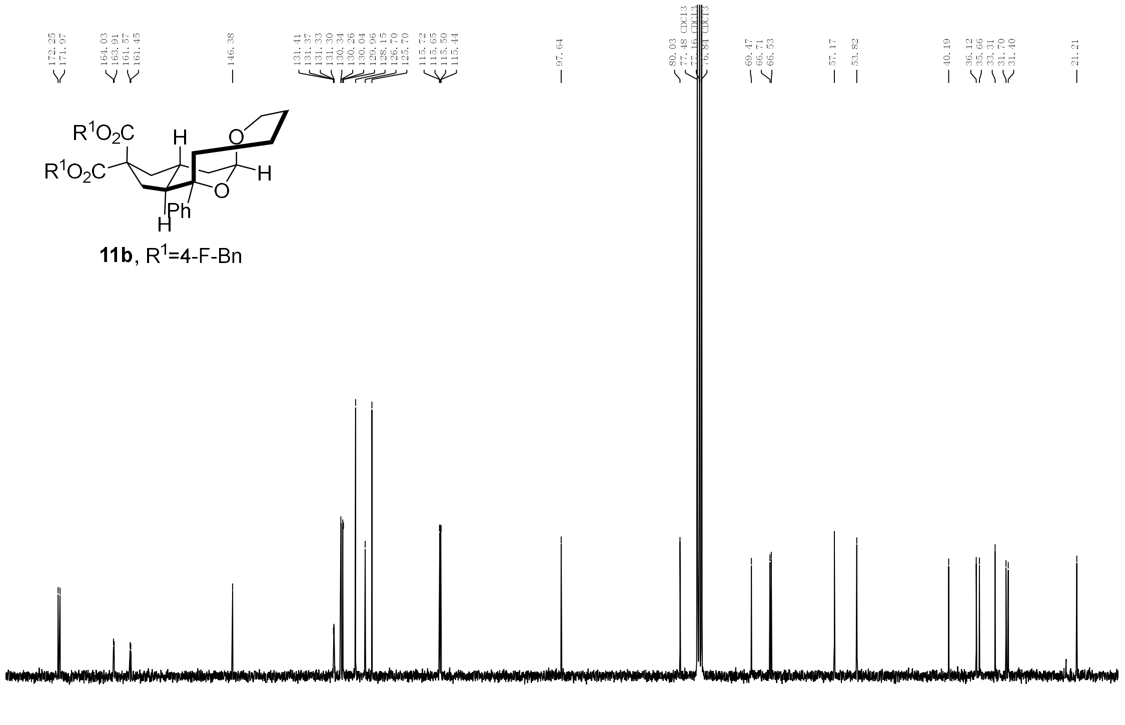

Supplementary Figure 239. ^{1}H NMR spectrum of 11c.

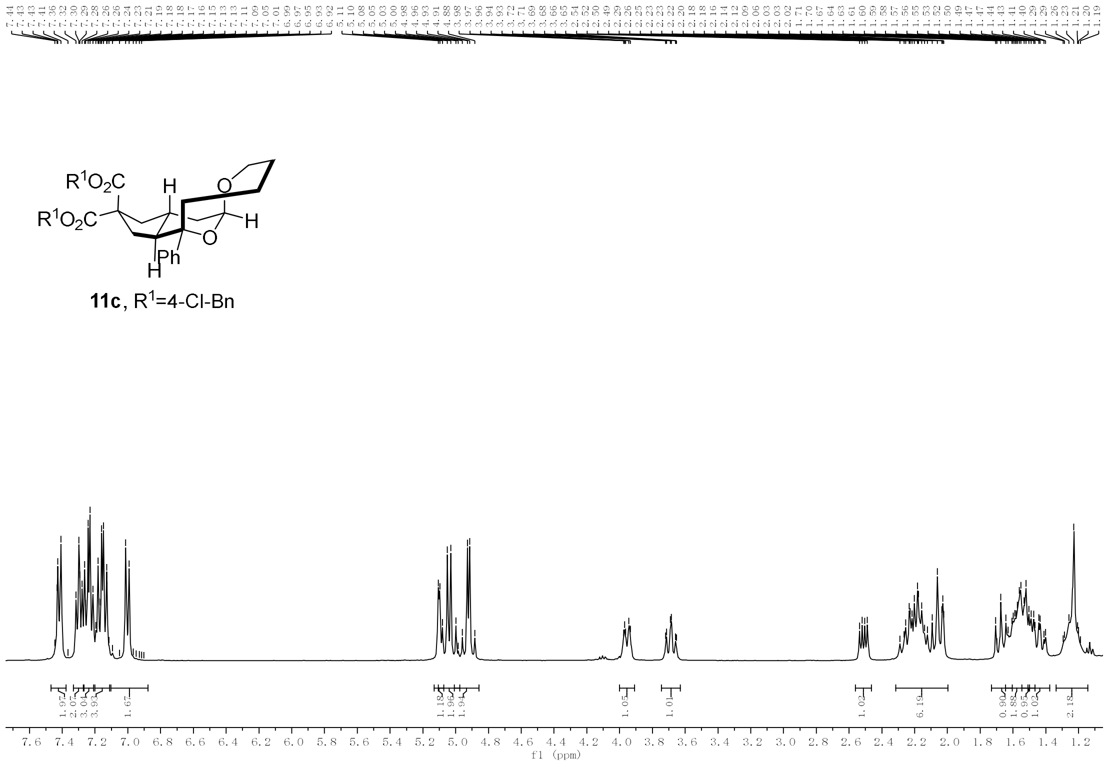

Supplementary Figure 240. ^{13}C NMR spectrum of 11c.

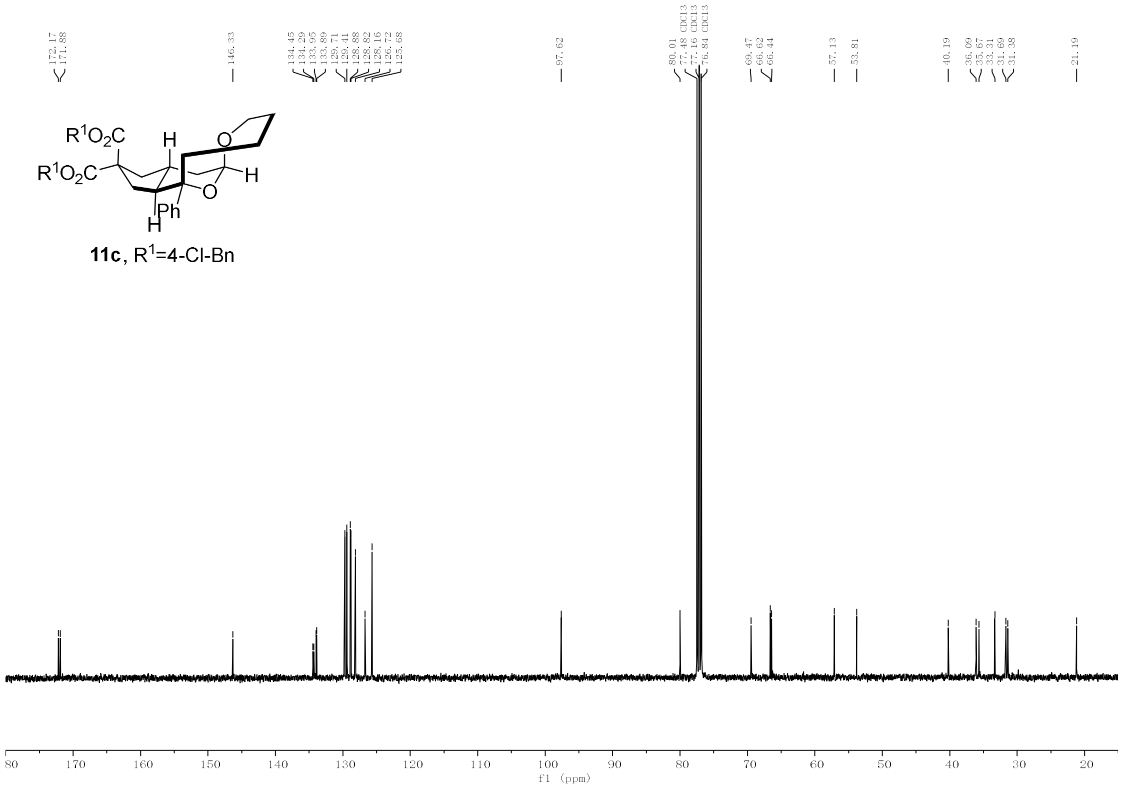

Supplementary Figure 241. ^{1} H NMR spectrum of 11d.

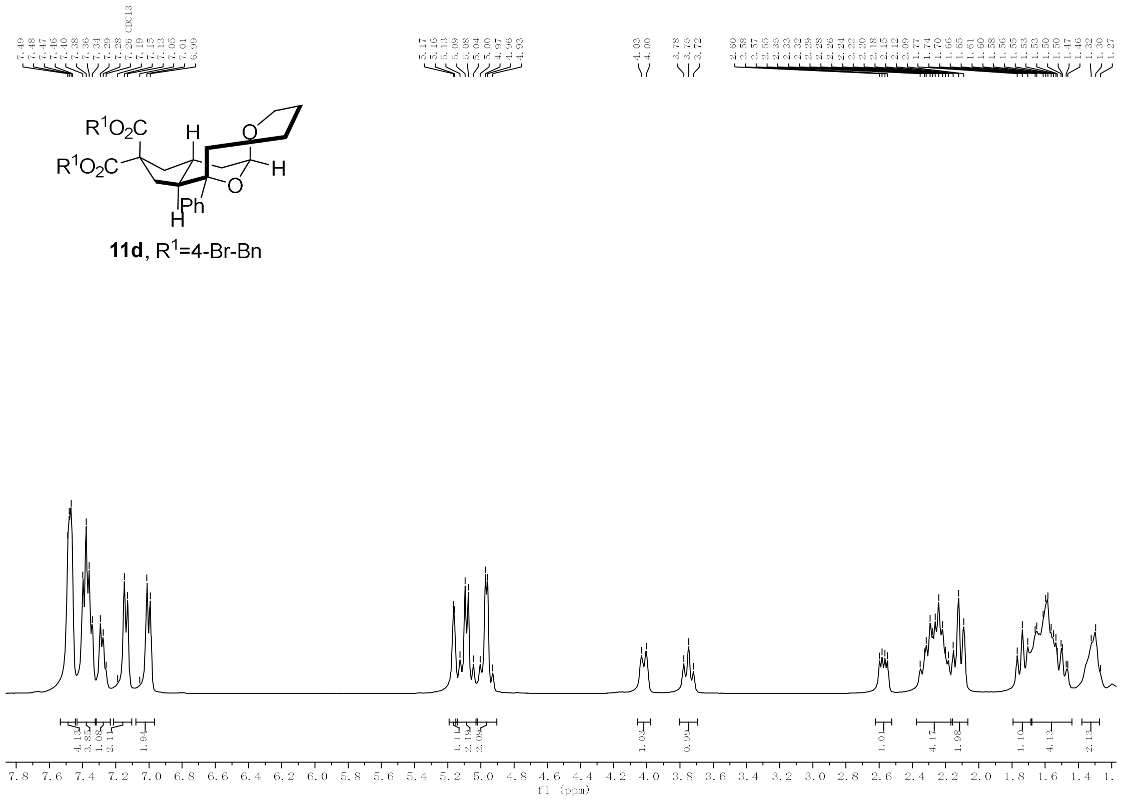

Supplementary Figure 242. ^{13} C NMR spectrum of 11d.

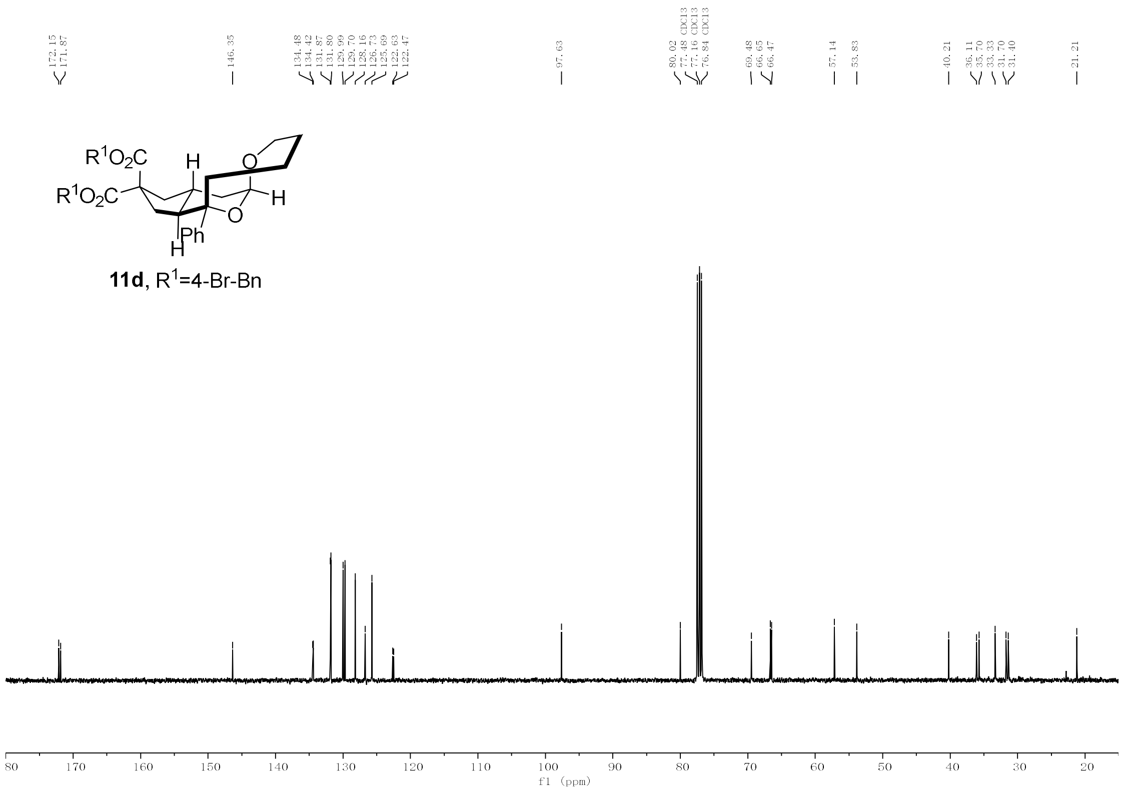

Supplementary Figure 243. [1] H NMR spectrum of 11e.

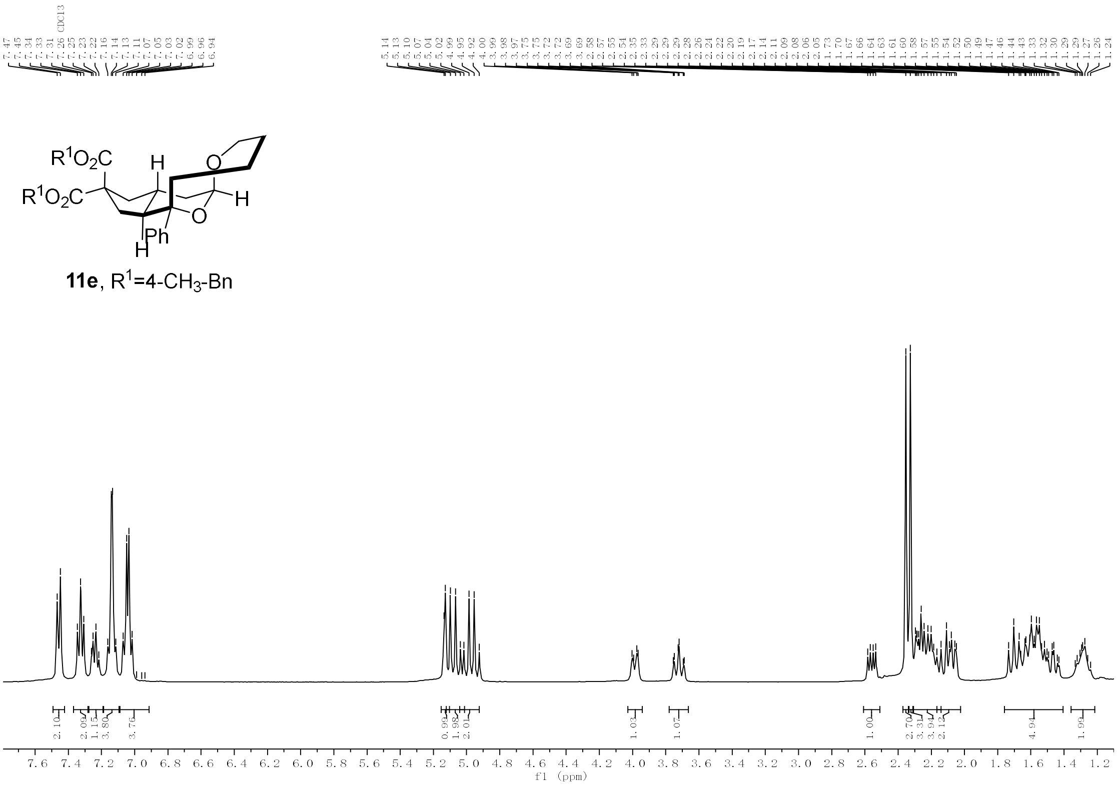

Supplementary Figure 244. [13] C NMR spectrum of 11e.

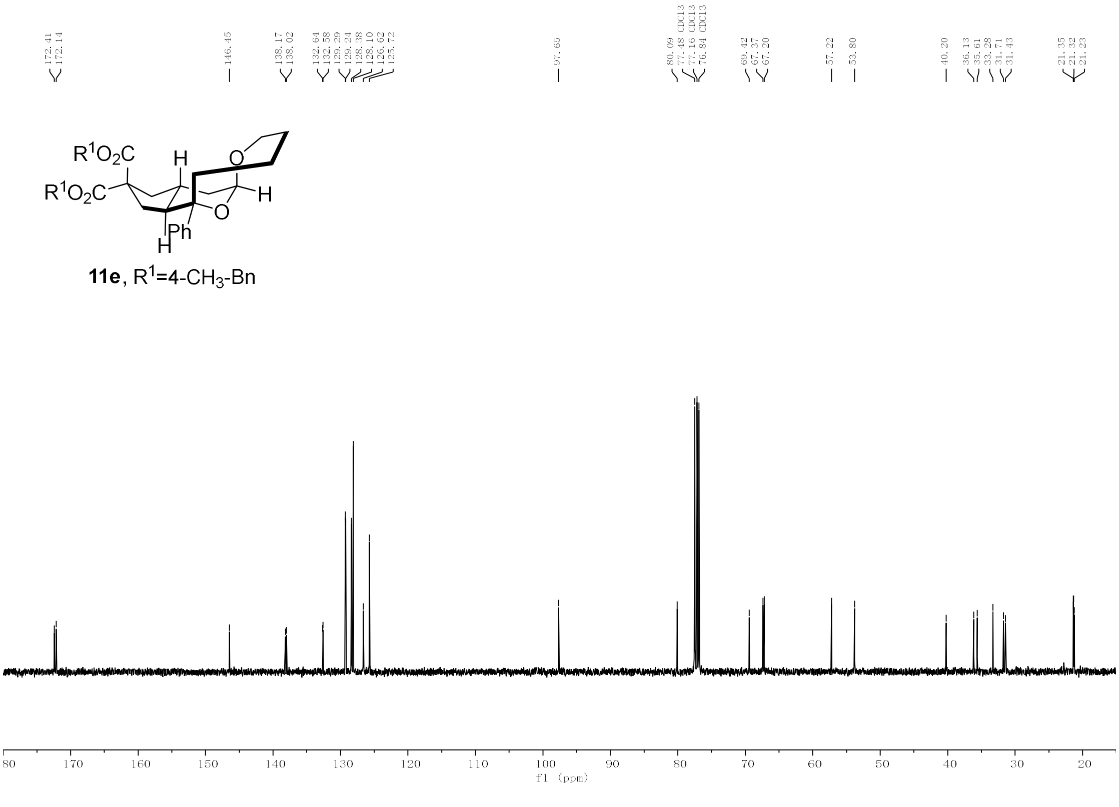

Supplementary Figure 245. ^{1}H NMR spectrum of 11f.

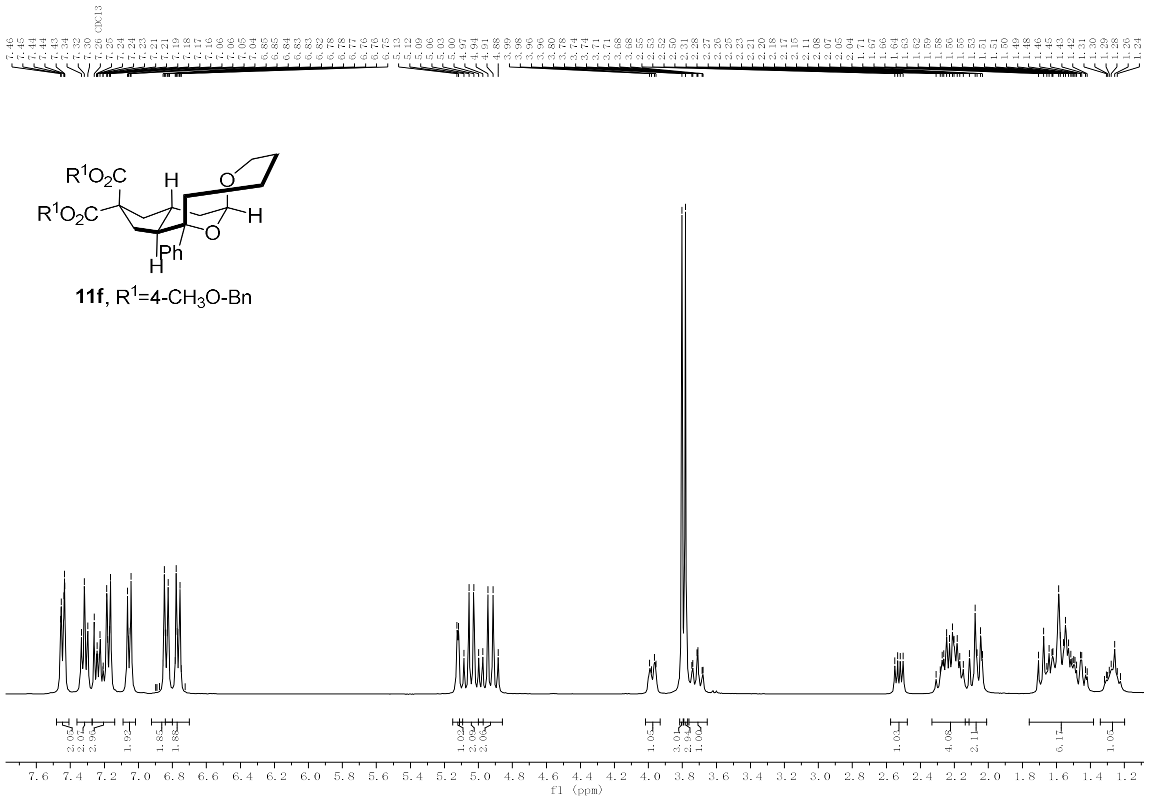

Supplementary Figure 246. ^{13}C NMR spectrum of 11f.

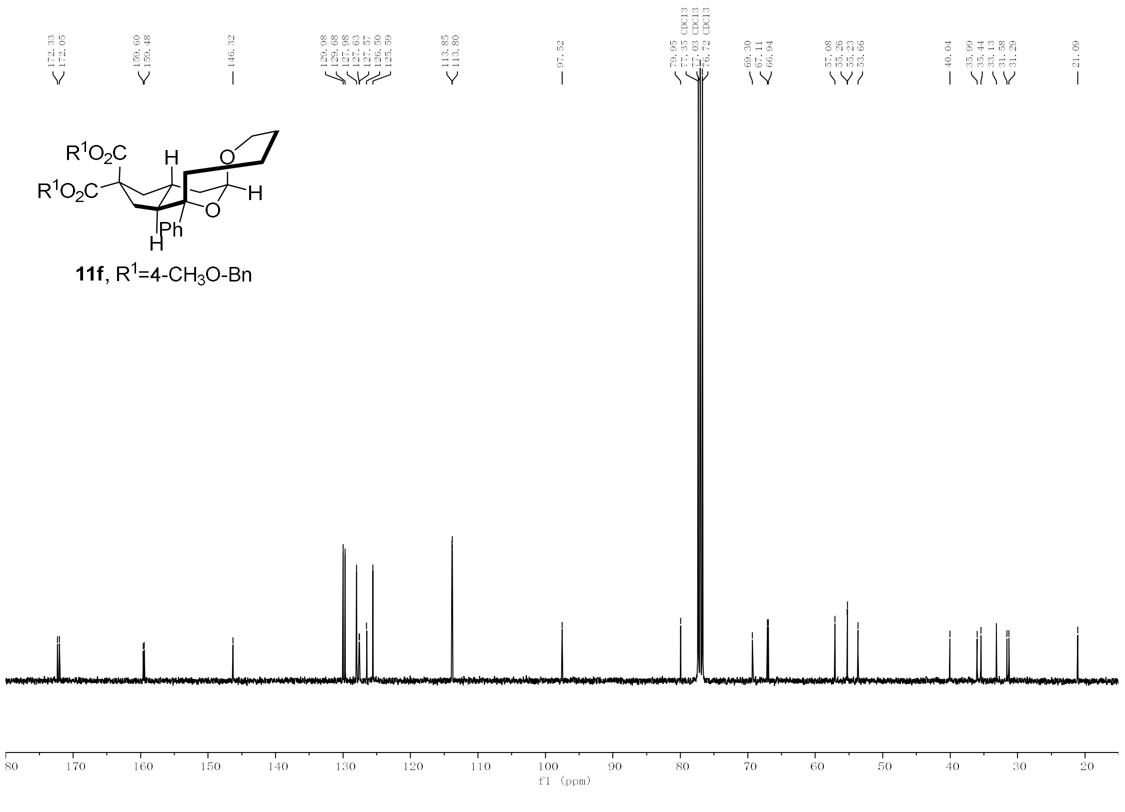

Supplementary Figure 247. ^{1} H NMR spectrum of 11g.

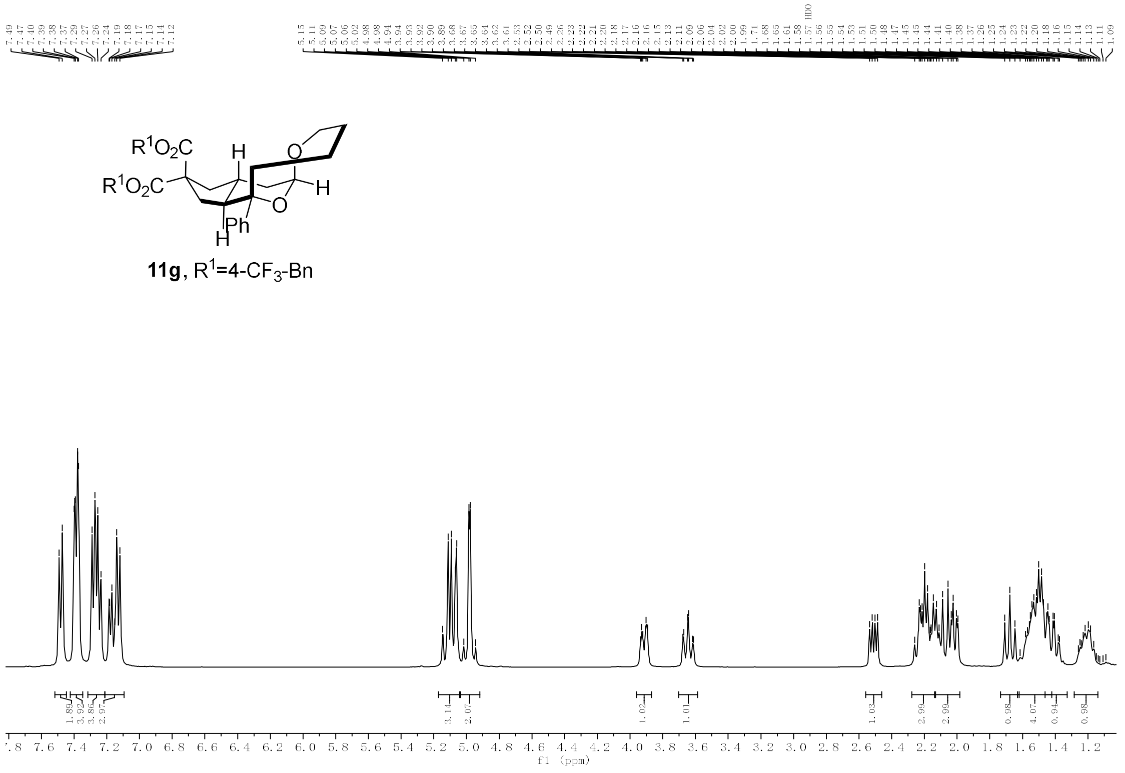

Supplementary Figure 248. ^{13} C NMR spectrum of 11g.

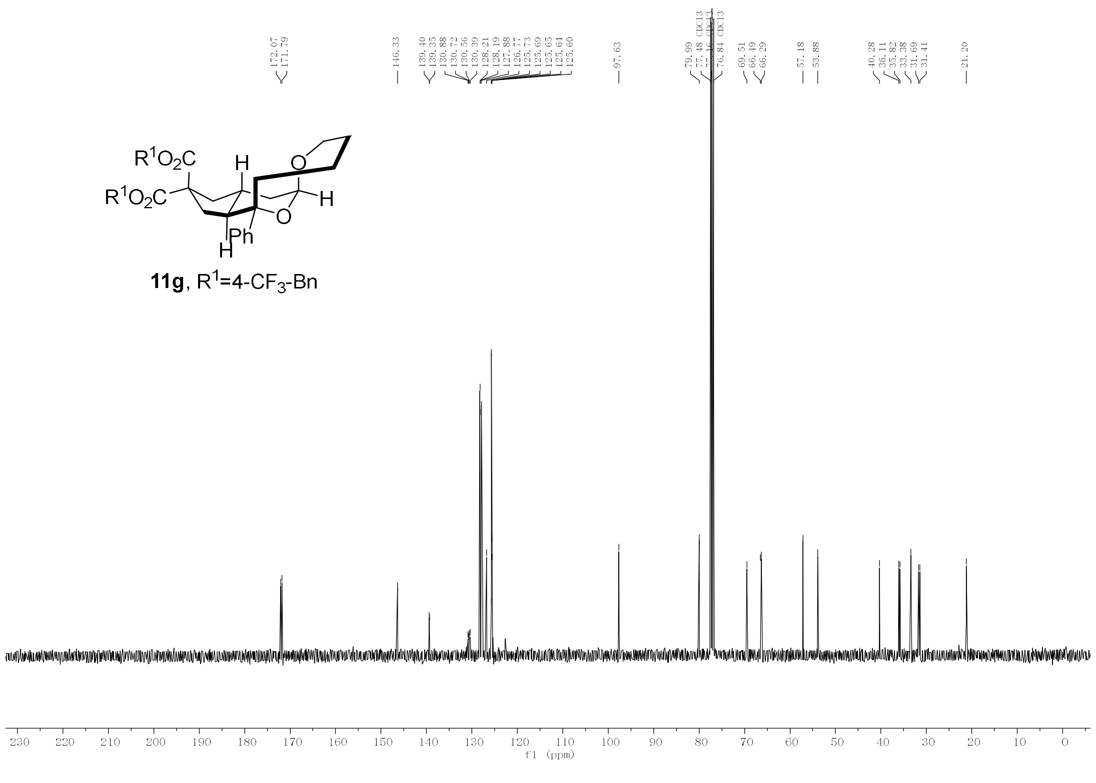

Supplementary Figure 249. ^{1}H NMR spectrum of 11h.

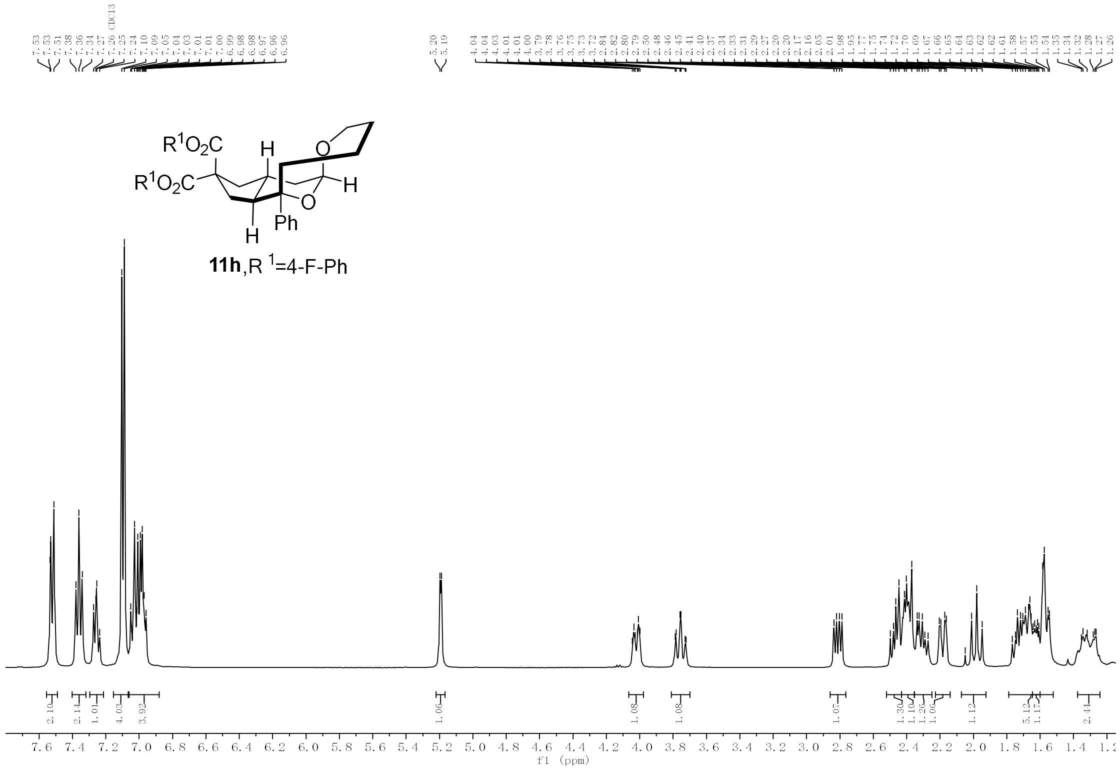

Supplementary Figure 250. ^{13}C NMR spectrum of 11h.

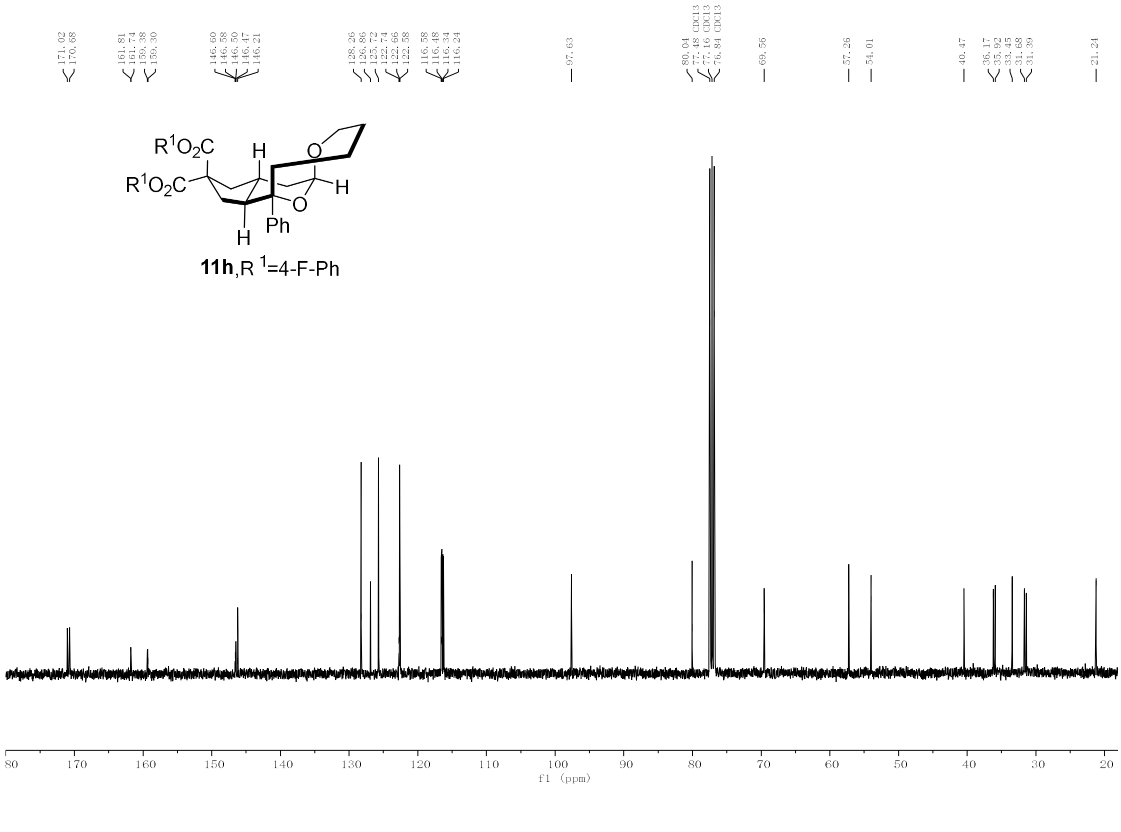

Supplementary Figure 251. ^{1}H NMR spectrum of 11i.

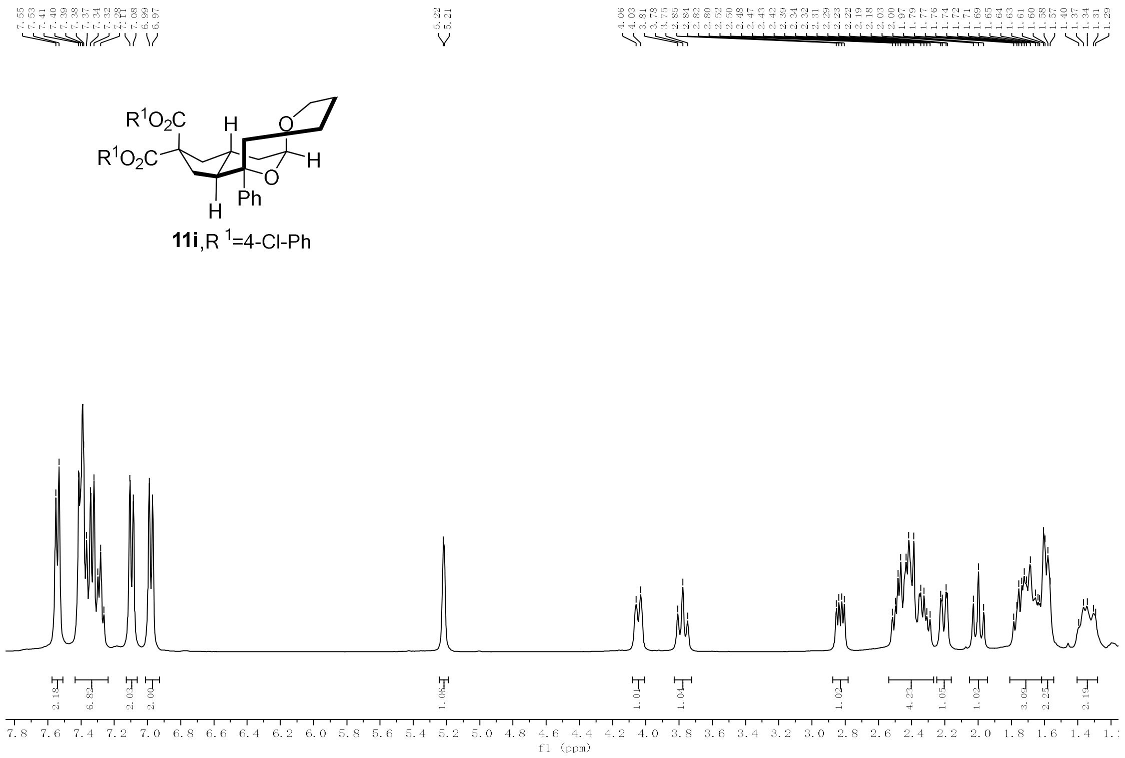

Supplementary Figure 252. ^{13}C NMR spectrum of 11i.

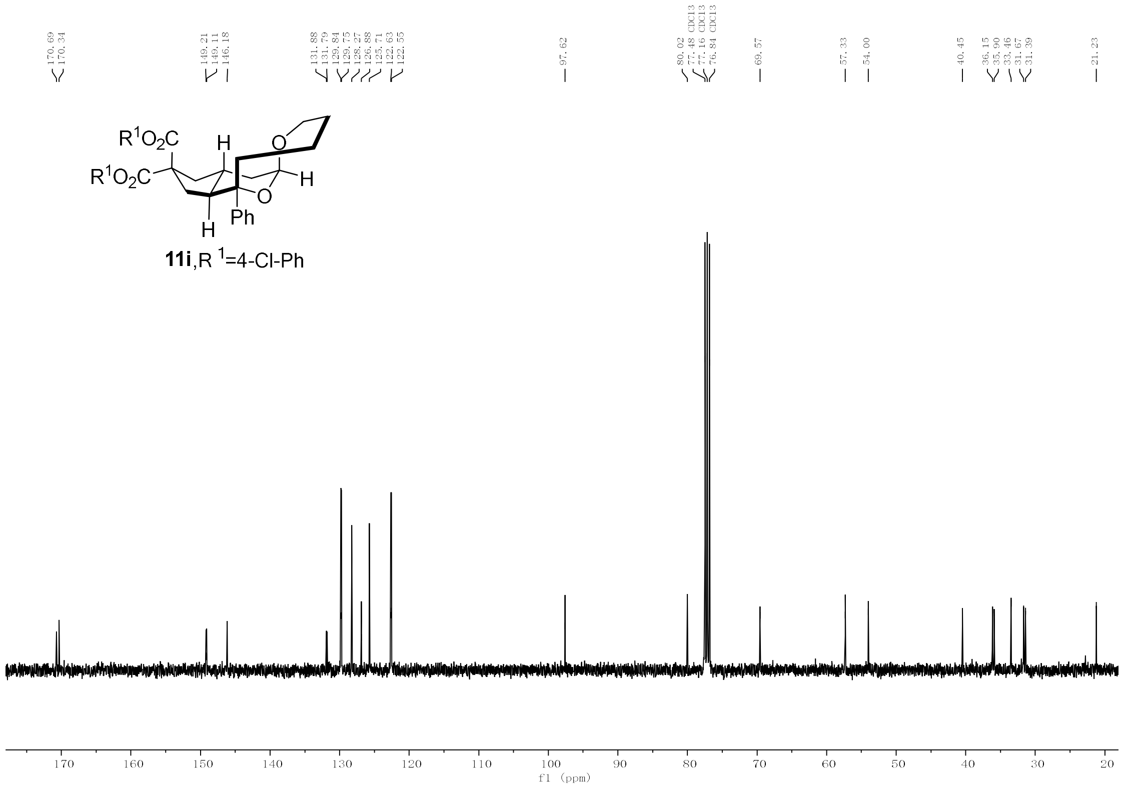

Supplementary Figure 253. ^{1} H NMR spectrum of 11j.

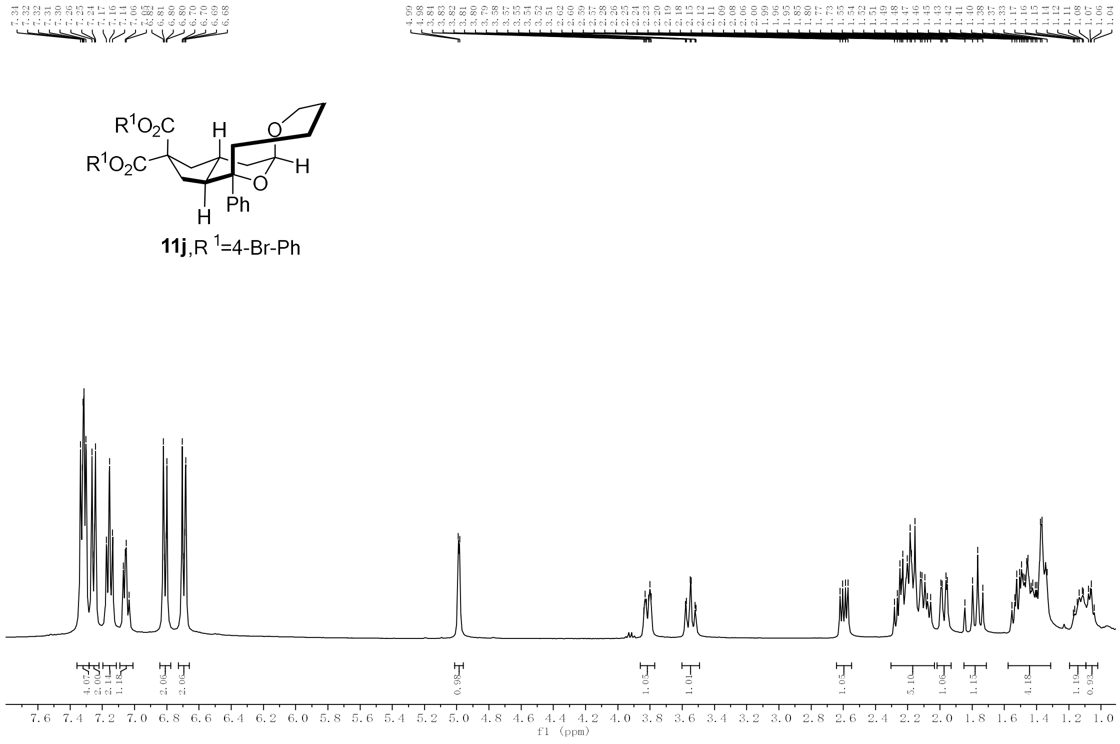

Supplementary Figure 254. ^{13} C NMR spectrum of 11j.

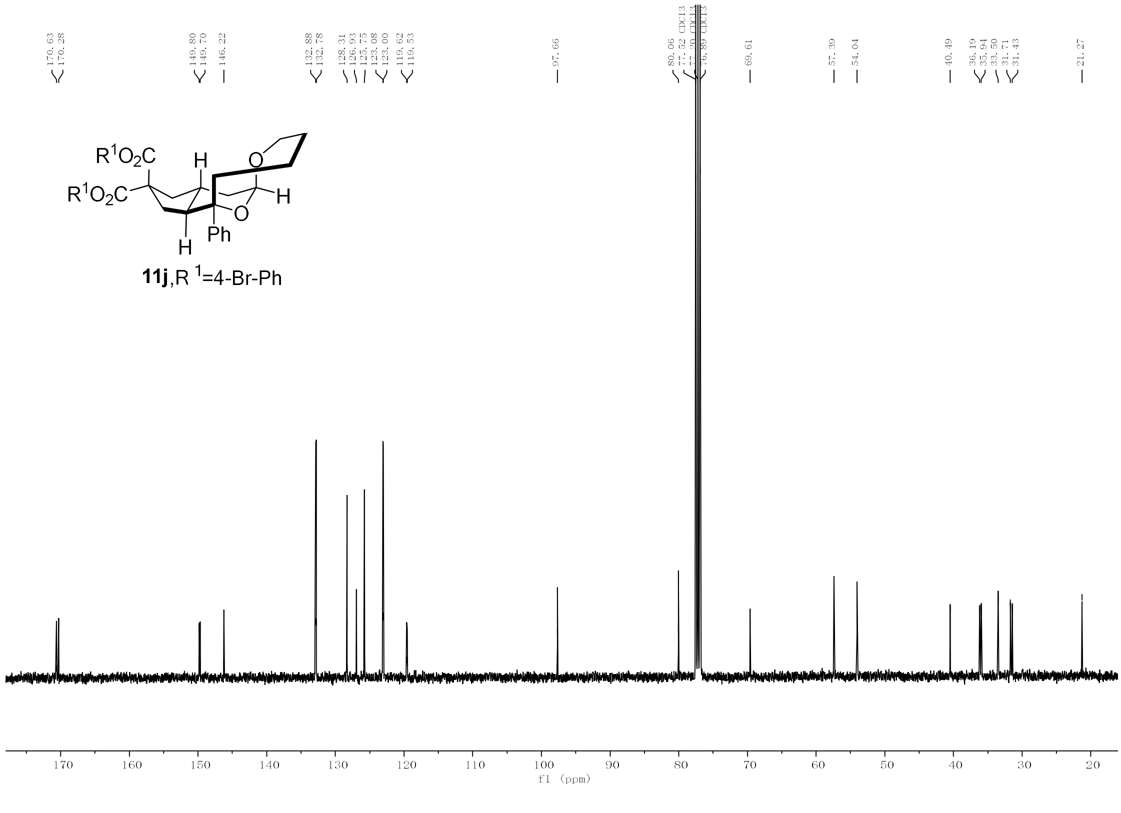

Supplementary Figure 255. ^{1} H NMR spectrum of 11k.

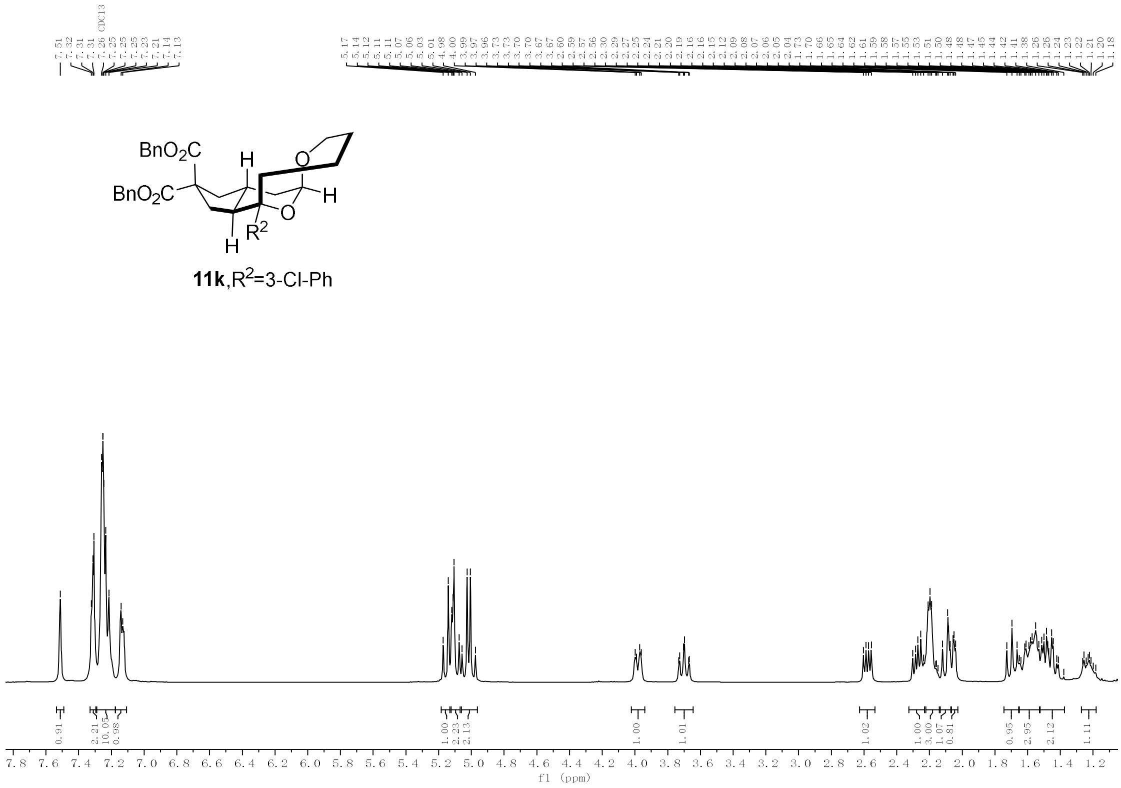

Supplementary Figure 256. ^{13} C NMR spectrum of 11k.

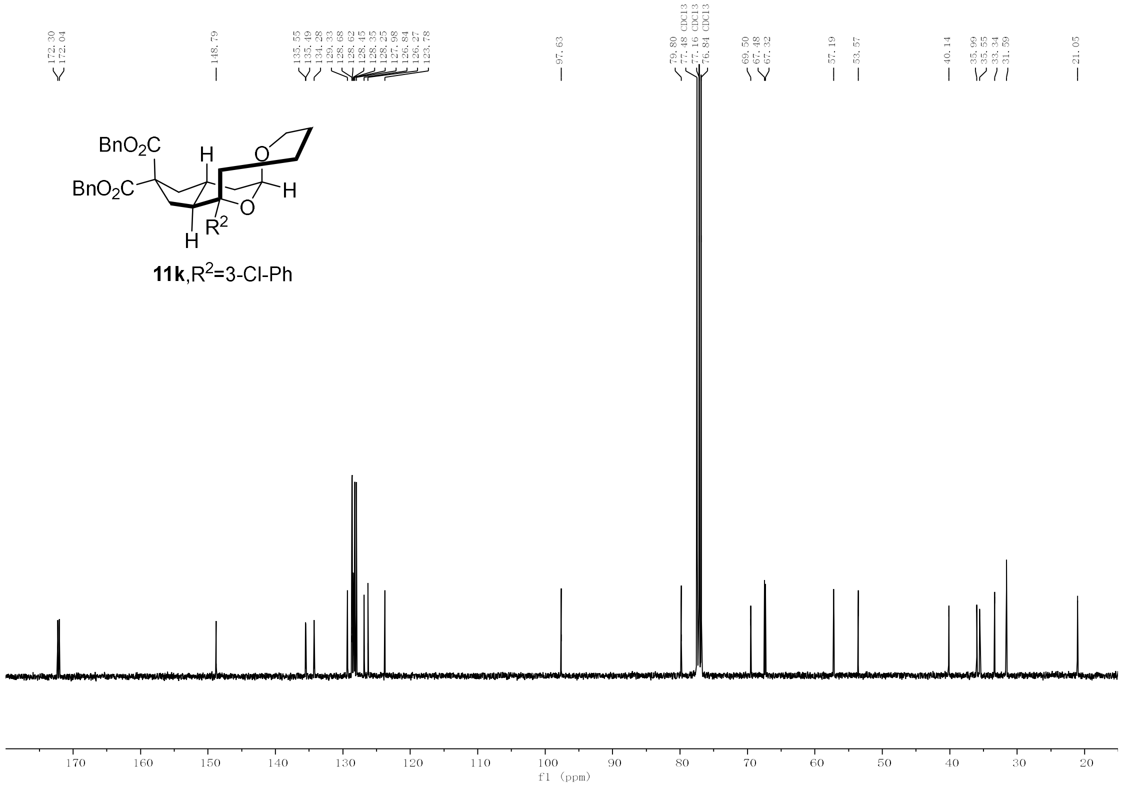

Supplementary Figure 257. ^{1} H NMR spectrum of 11l.

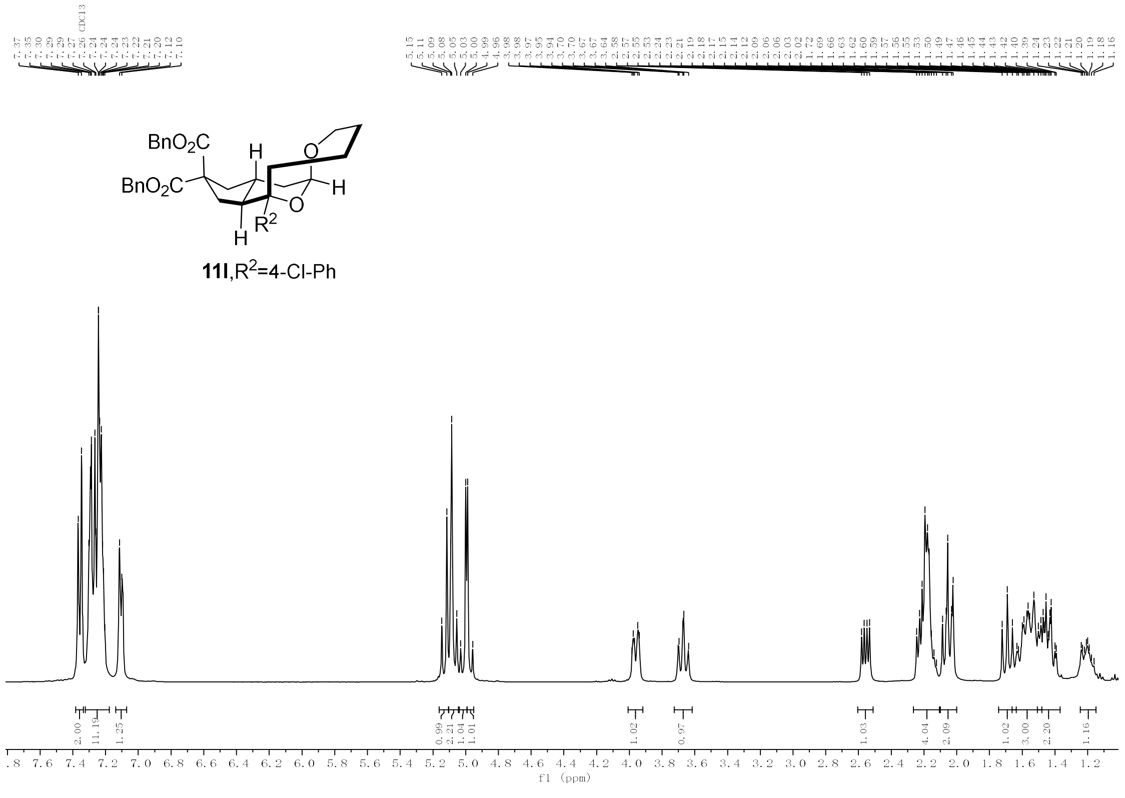

Supplementary Figure 258. ^{13} C NMR spectrum of 11l.

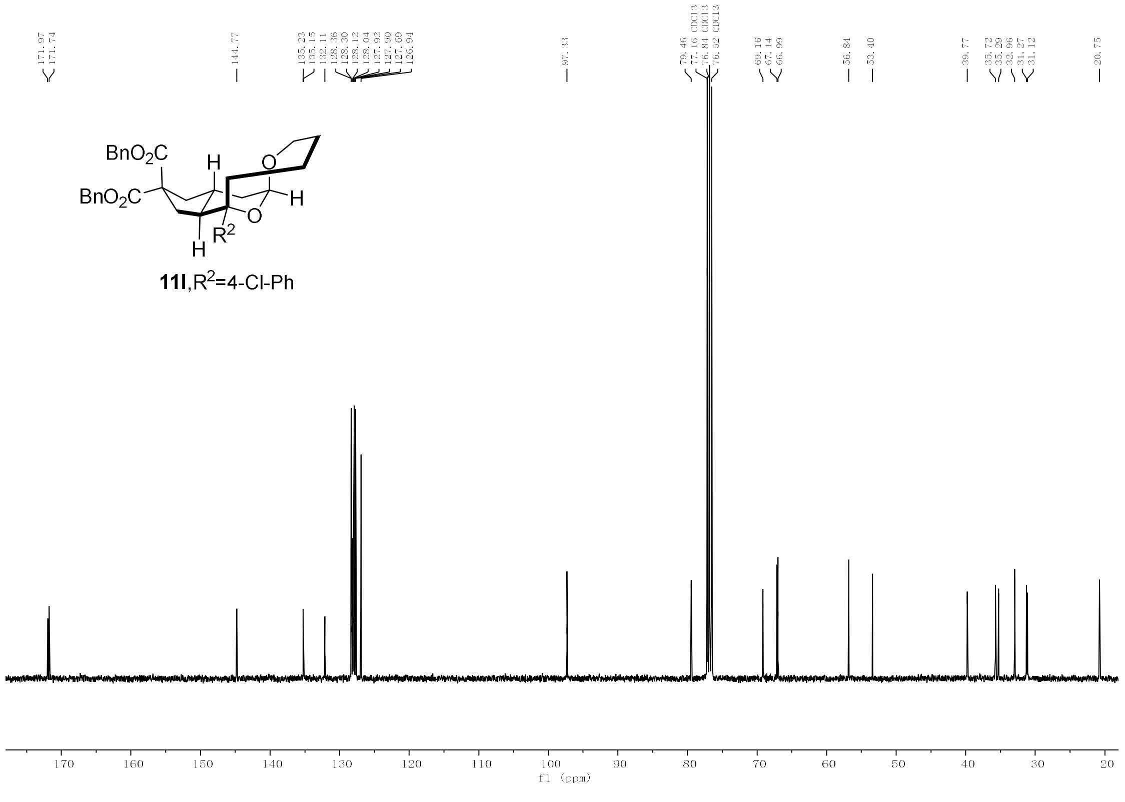

Supplementary Figure 259. ¹ H NMR spectrum of 11m.

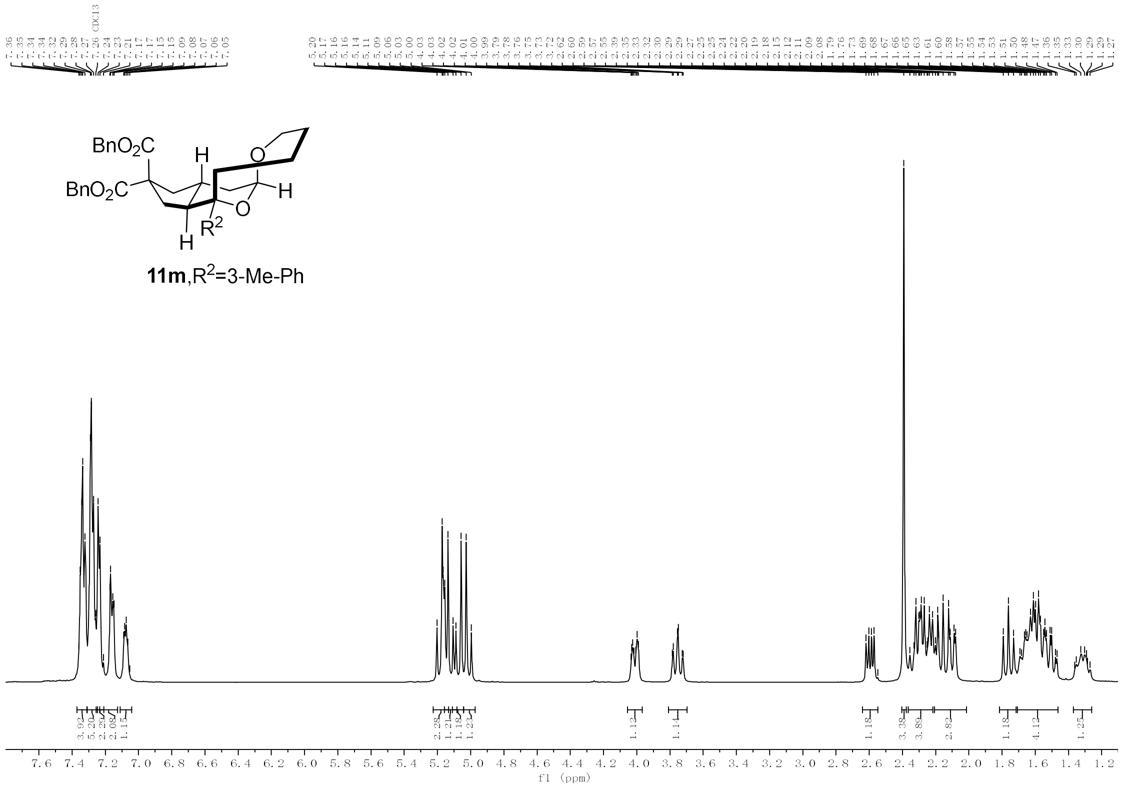

Supplementary Figure 260. ¹³ C NMR spectrum of 11m.

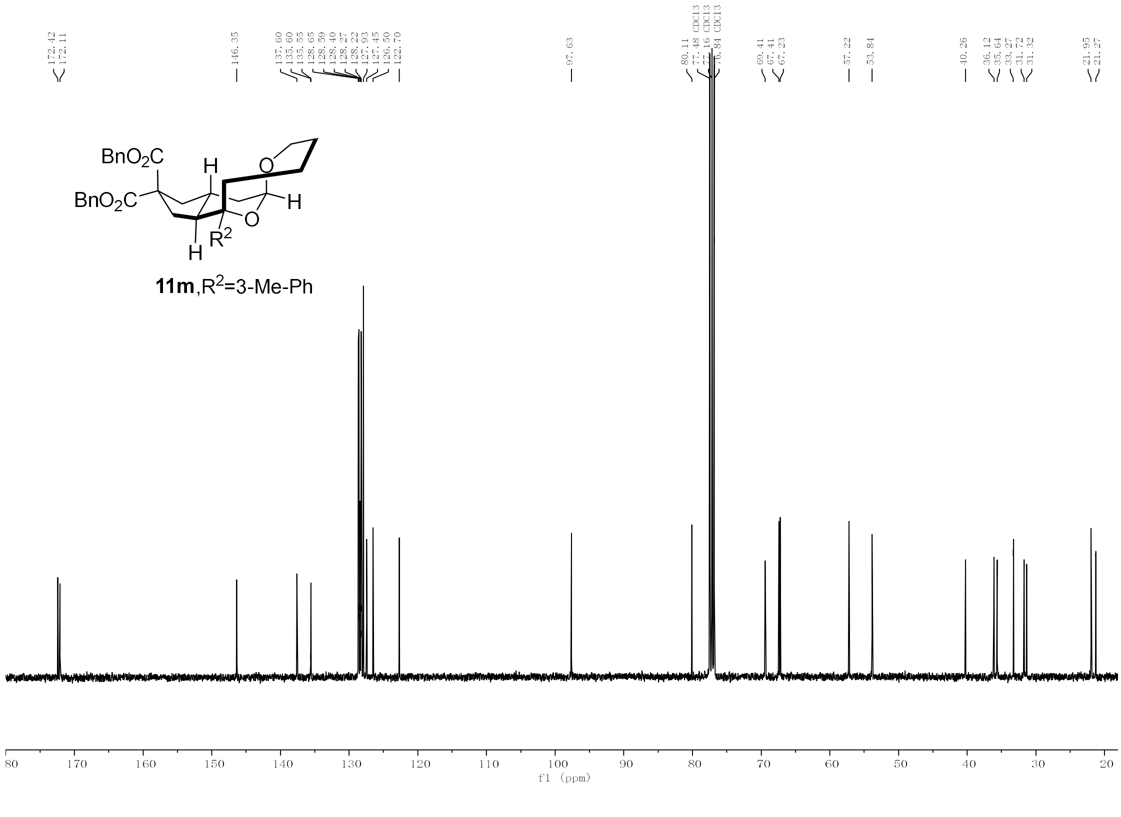

Supplementary Figure 261. ^{1}H NMR spectrum of 11n.

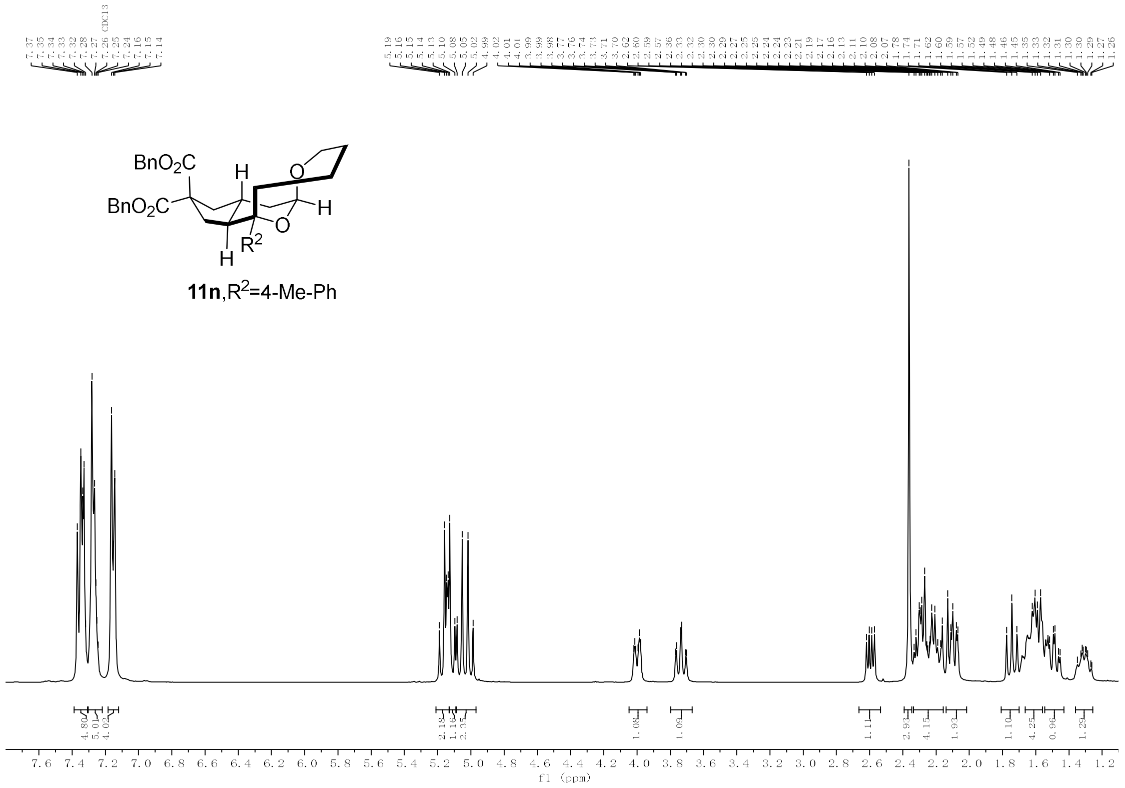

Supplementary Figure 262. ^{13}C NMR spectrum of 11n.

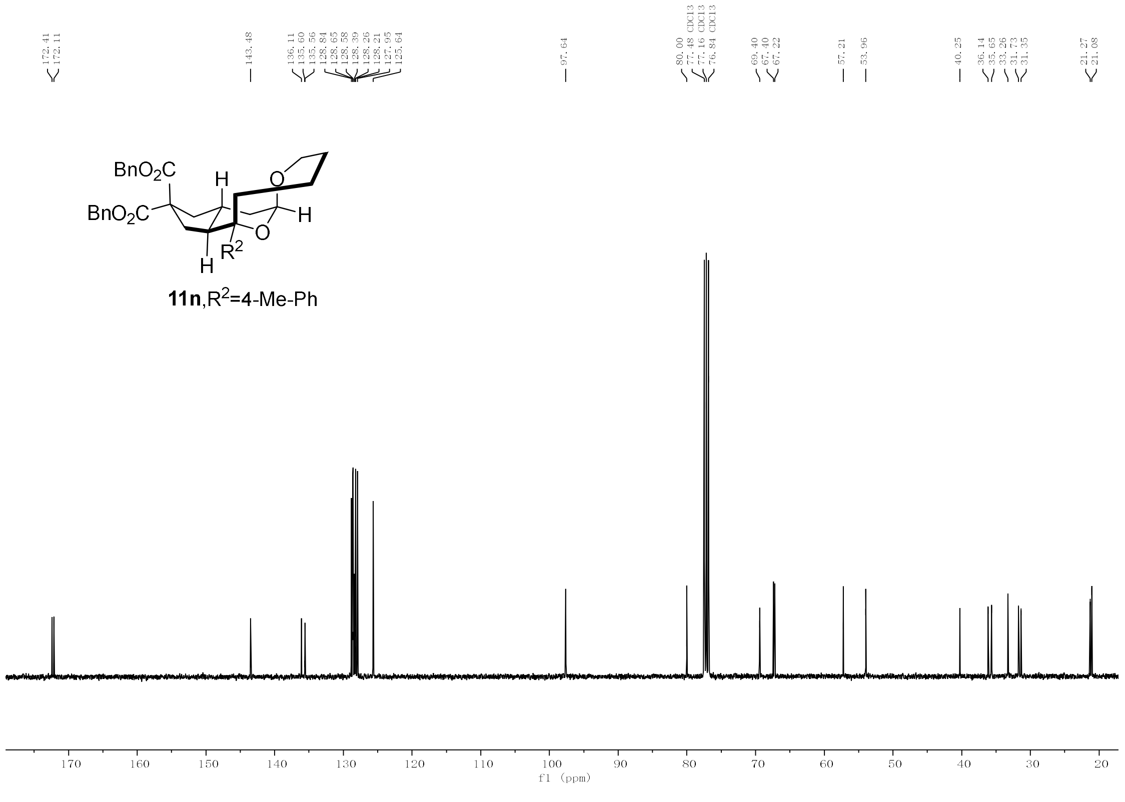

Supplementary Figure 263. [1] H NMR spectrum of 11o.

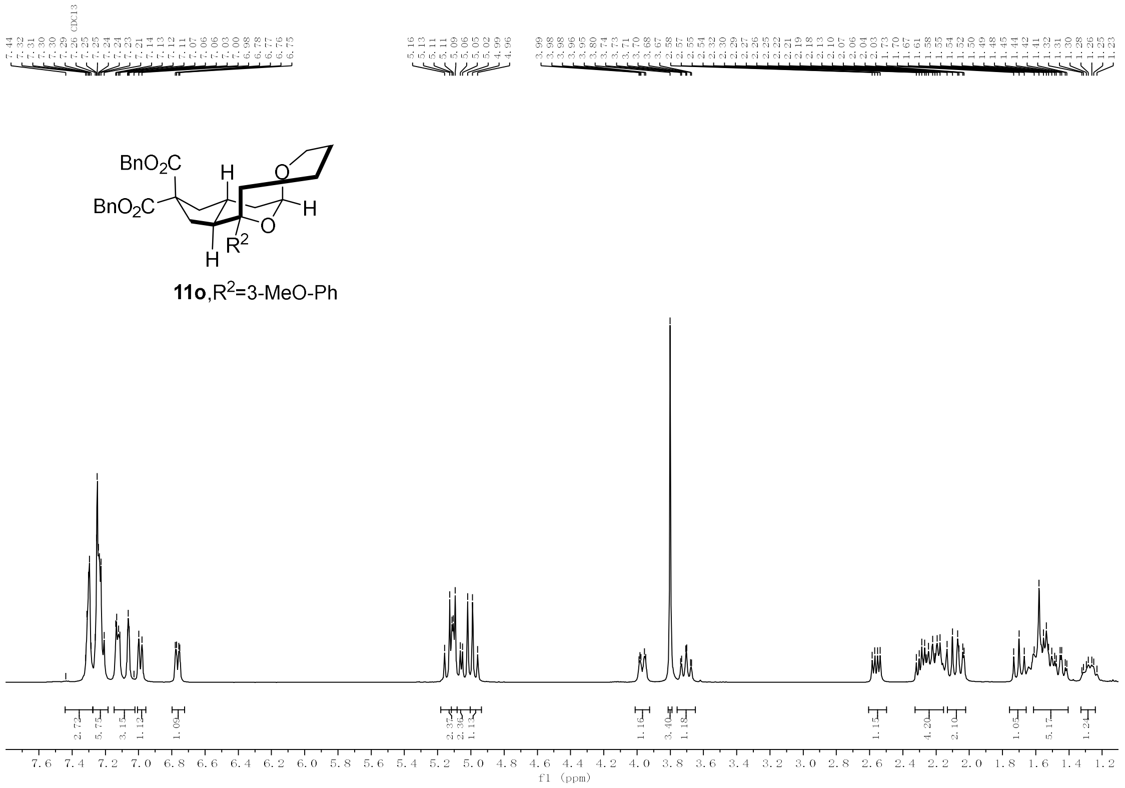

Supplementary Figure 264. [13] C NMR spectrum of 11o.

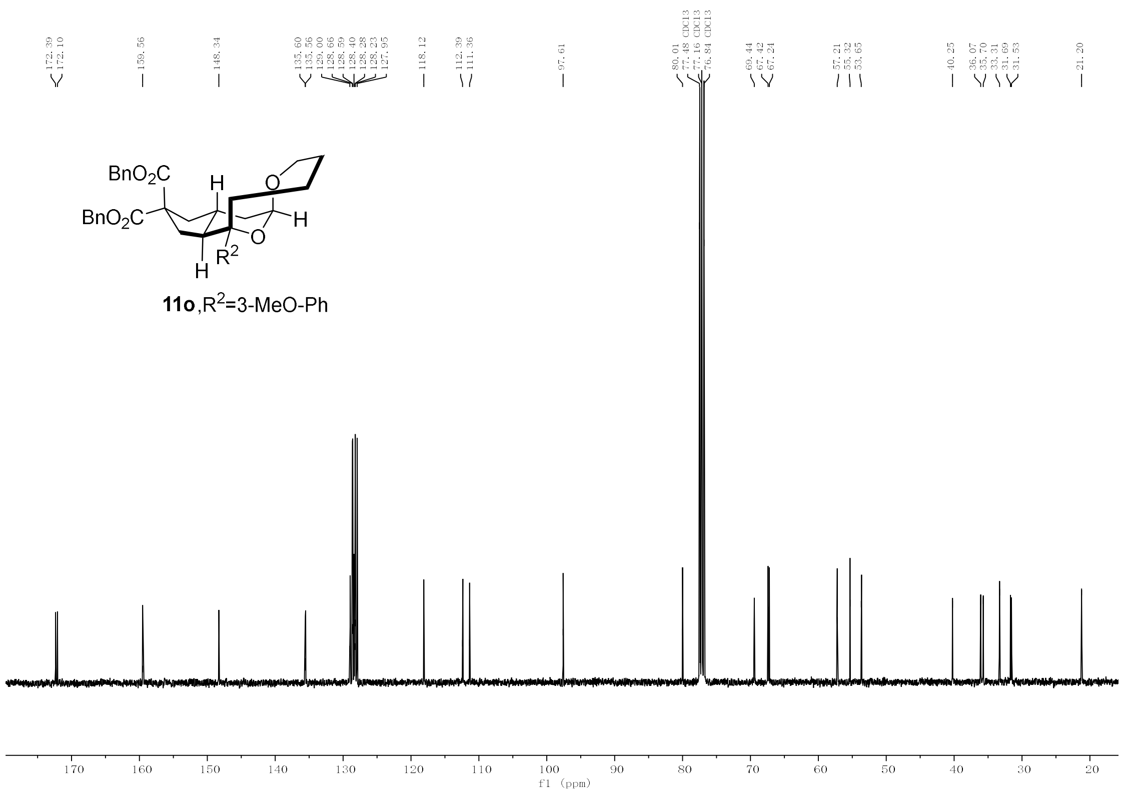

Supplementary Figure 265. ^{1} H NMR spectrum of 11p.

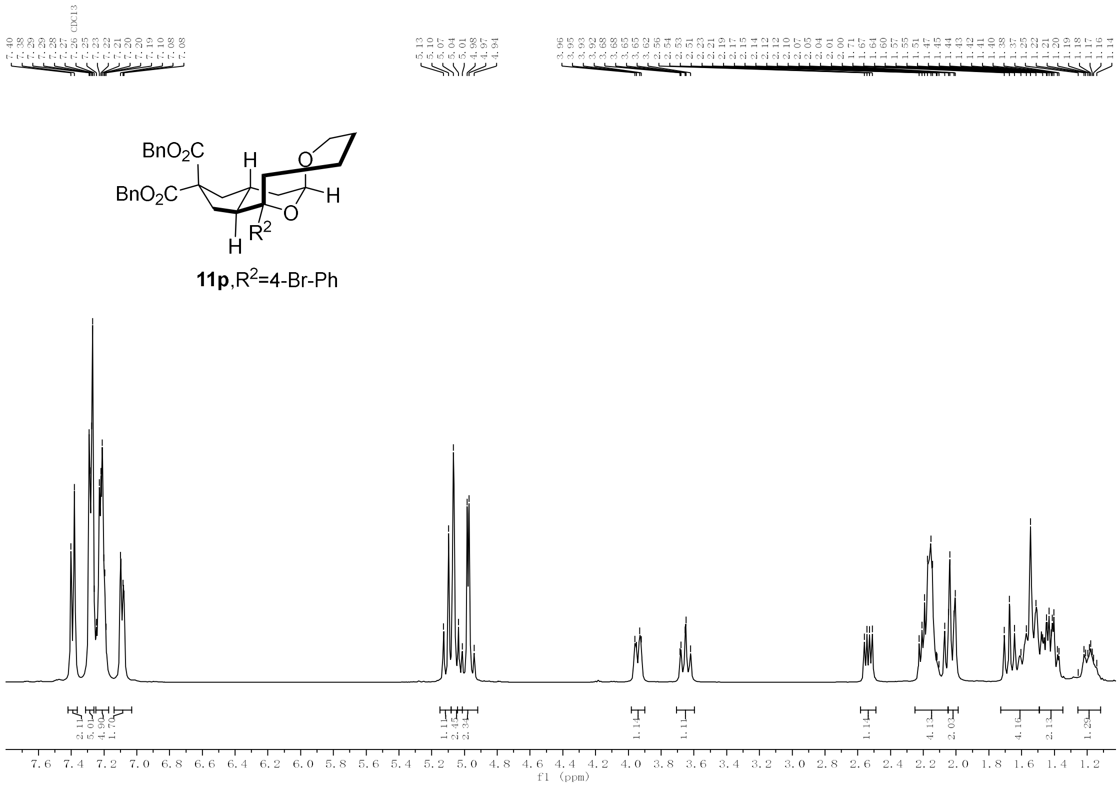

Supplementary Figure 266. ^{13} C NMR spectrum of 11p.

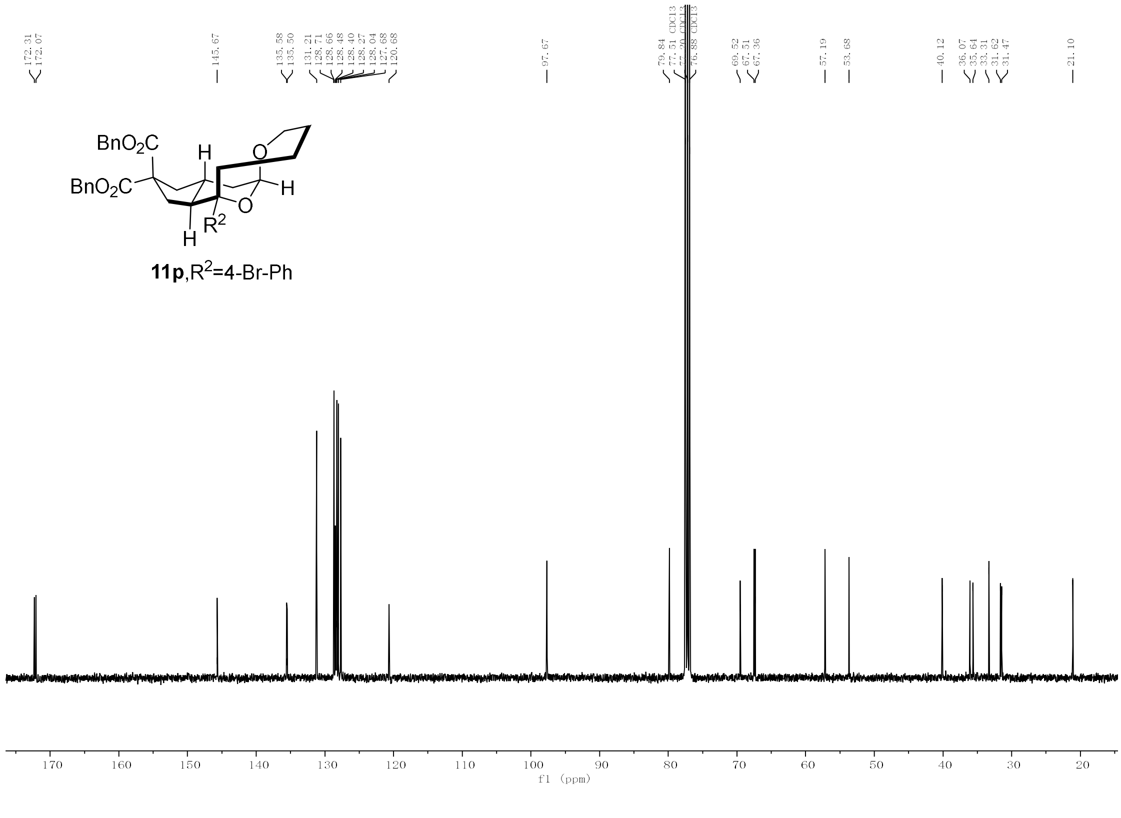

Supplementary Figure 267. ^{1}H NMR spectrum of 12.

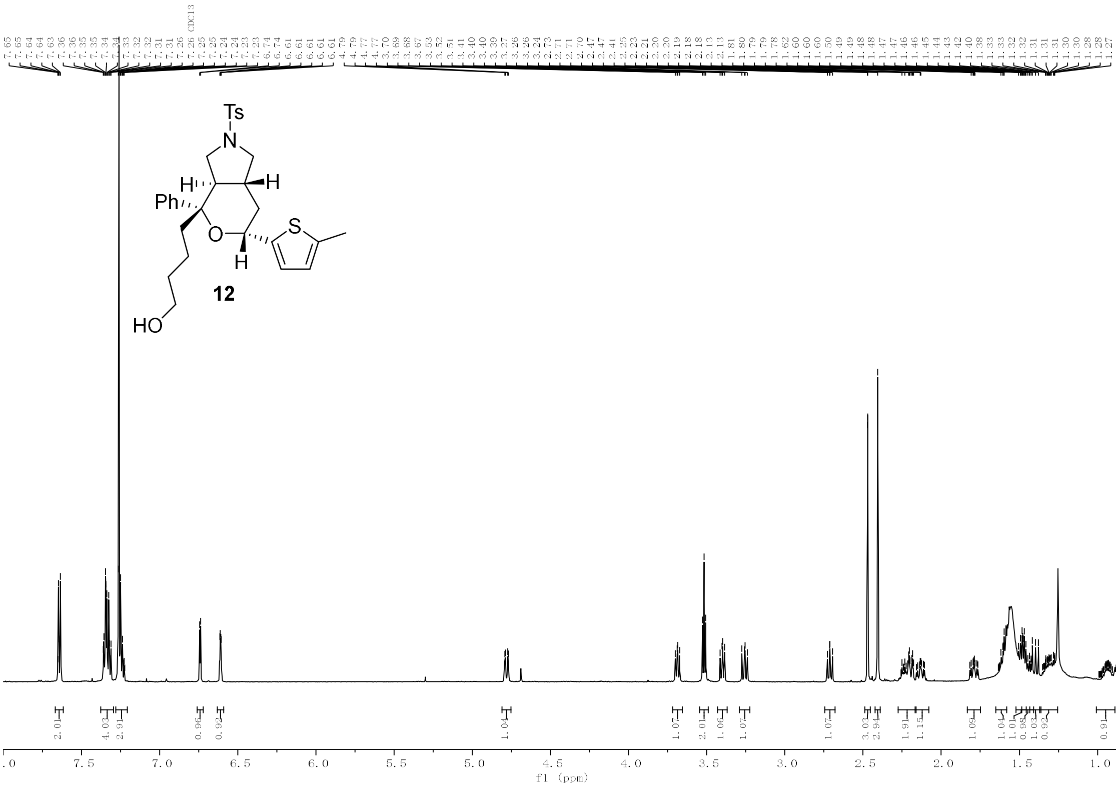

Supplementary Figure 268. ^{13}C NMR spectrum of 12.

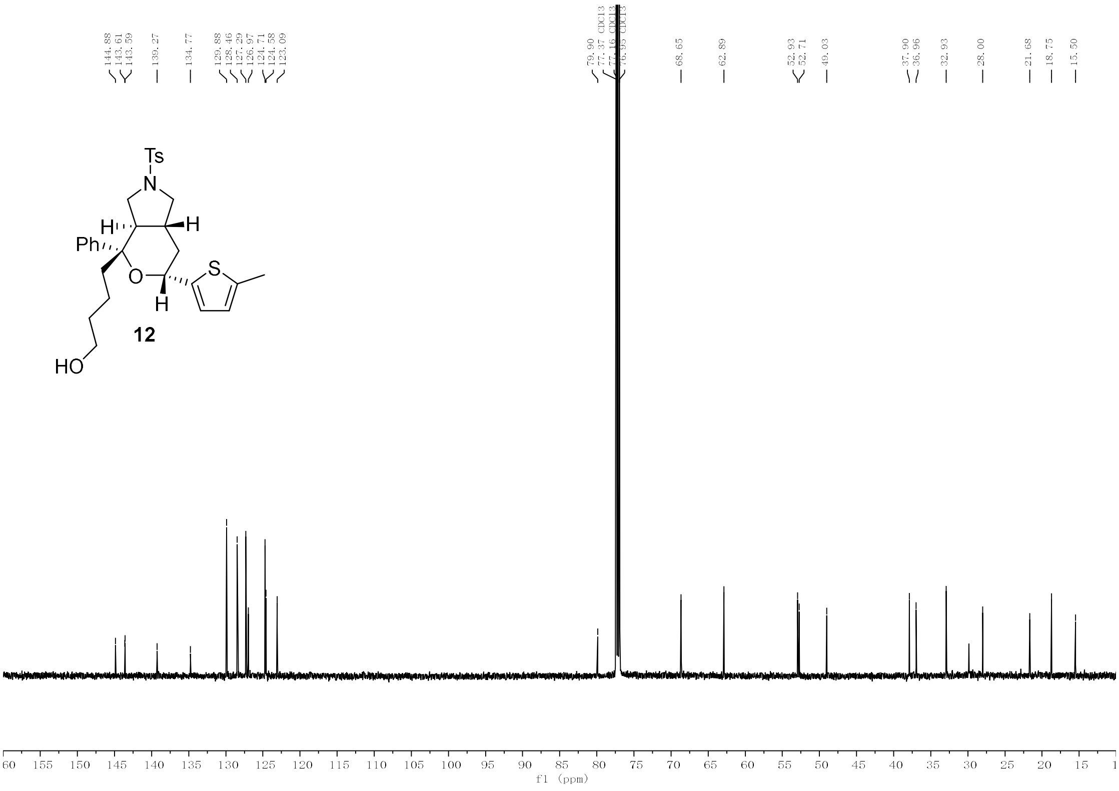

Supplementary Figure 269. [1] H NMR spectrum of 13.

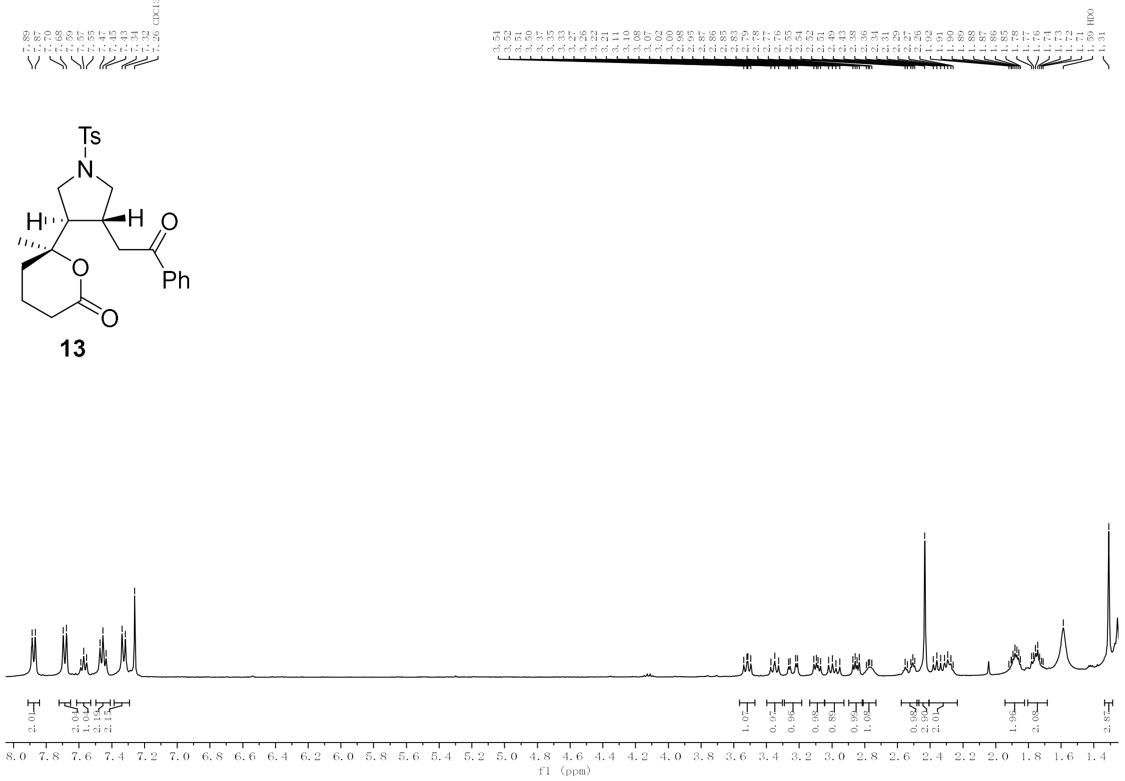

Supplementary Figure 270. [13] C NMR spectrum of 13.

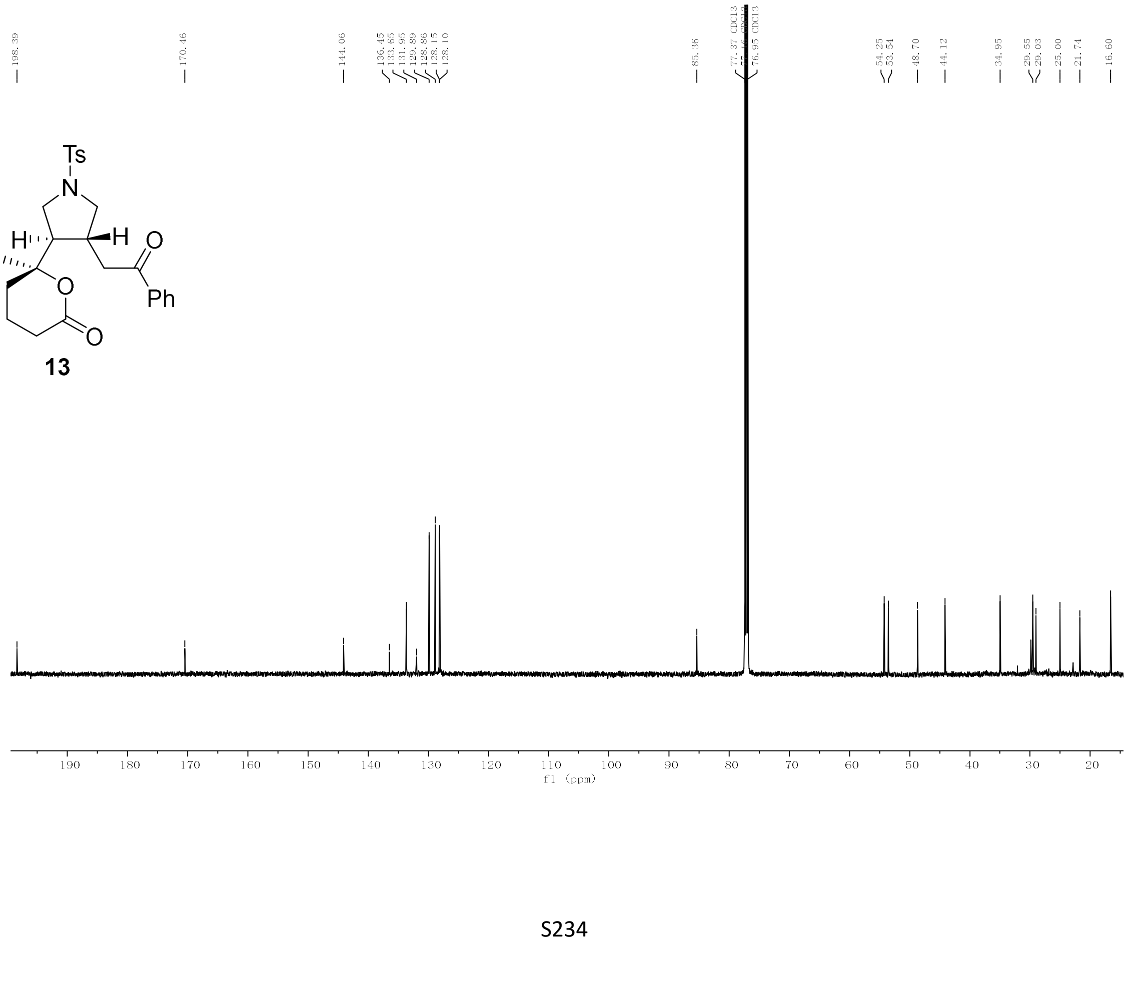

Supplementary Figure 271. ¹ **H NMR spectrum of 14**.

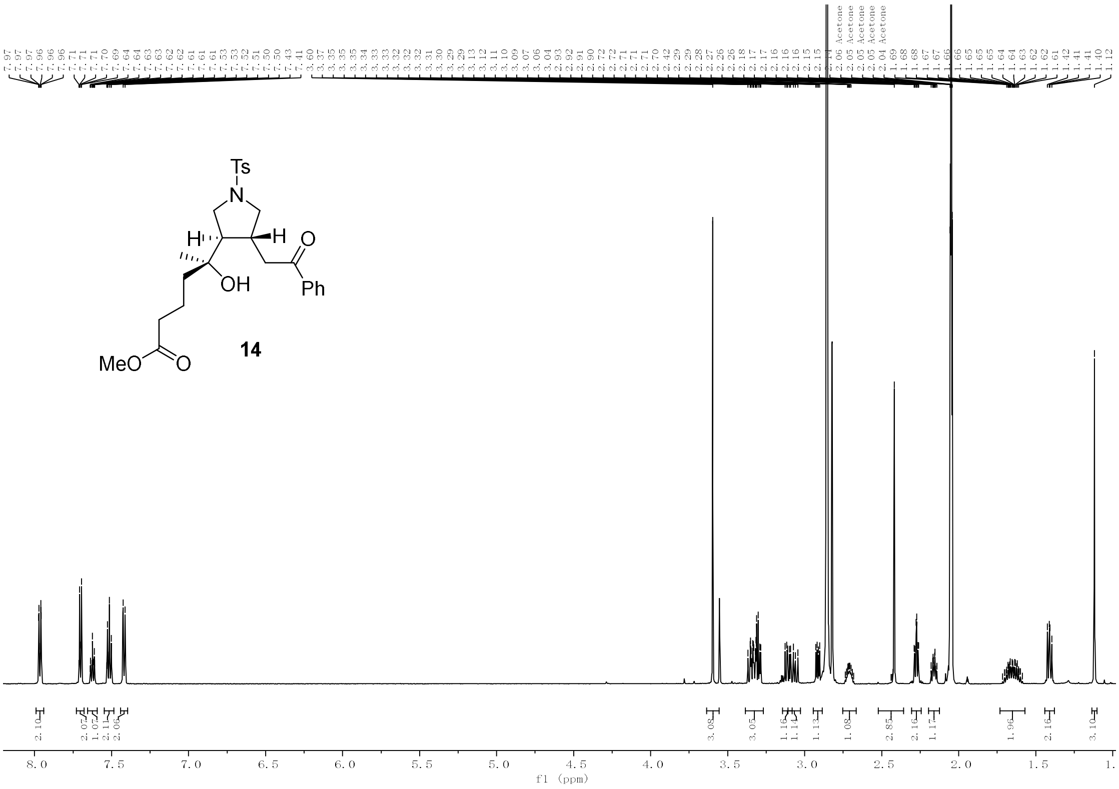

Supplementary Figure 272. ¹³ **C NMR spectrum of 14**.

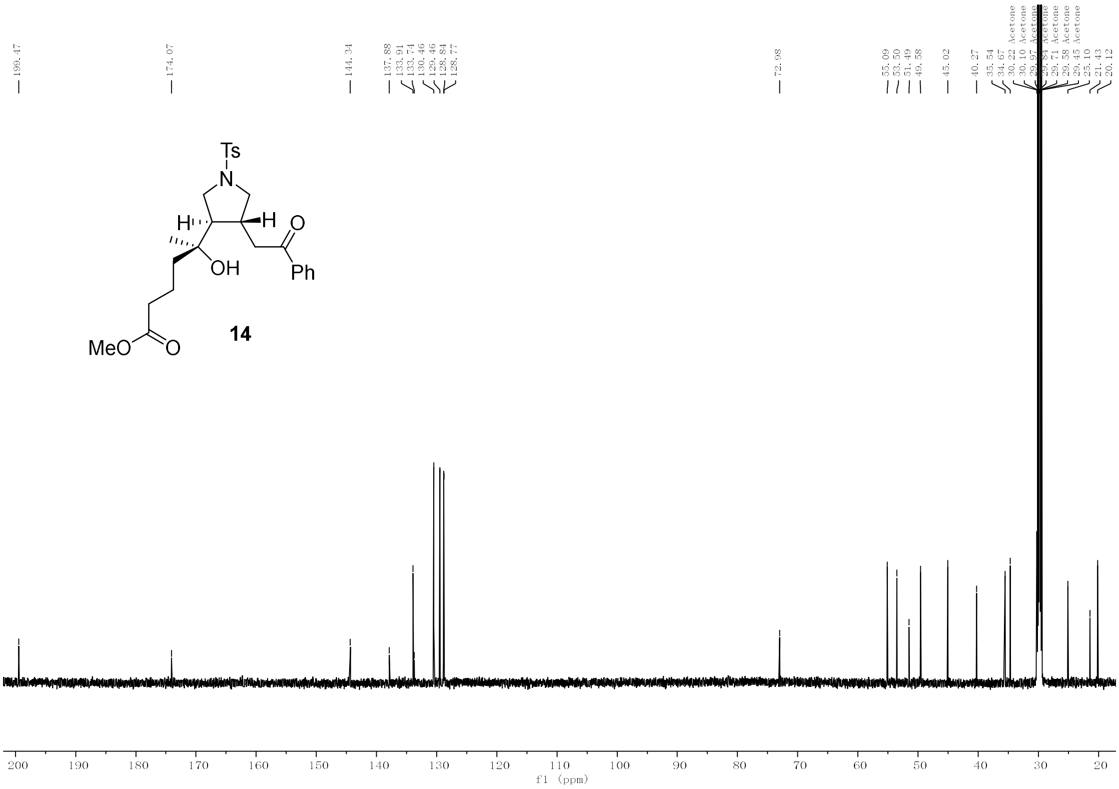

Supplementary Figure 273. ¹ H NMR spectrum of 16.

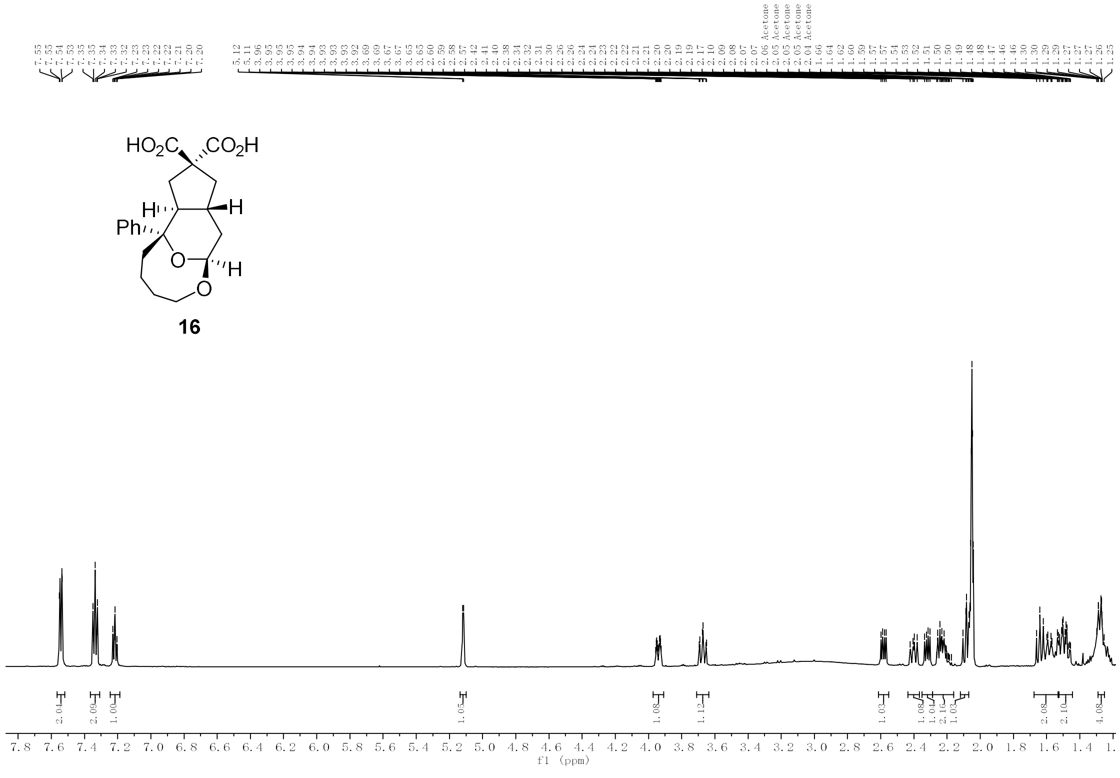

Supplementary Figure 274. ¹³ C NMR spectrum of 16.

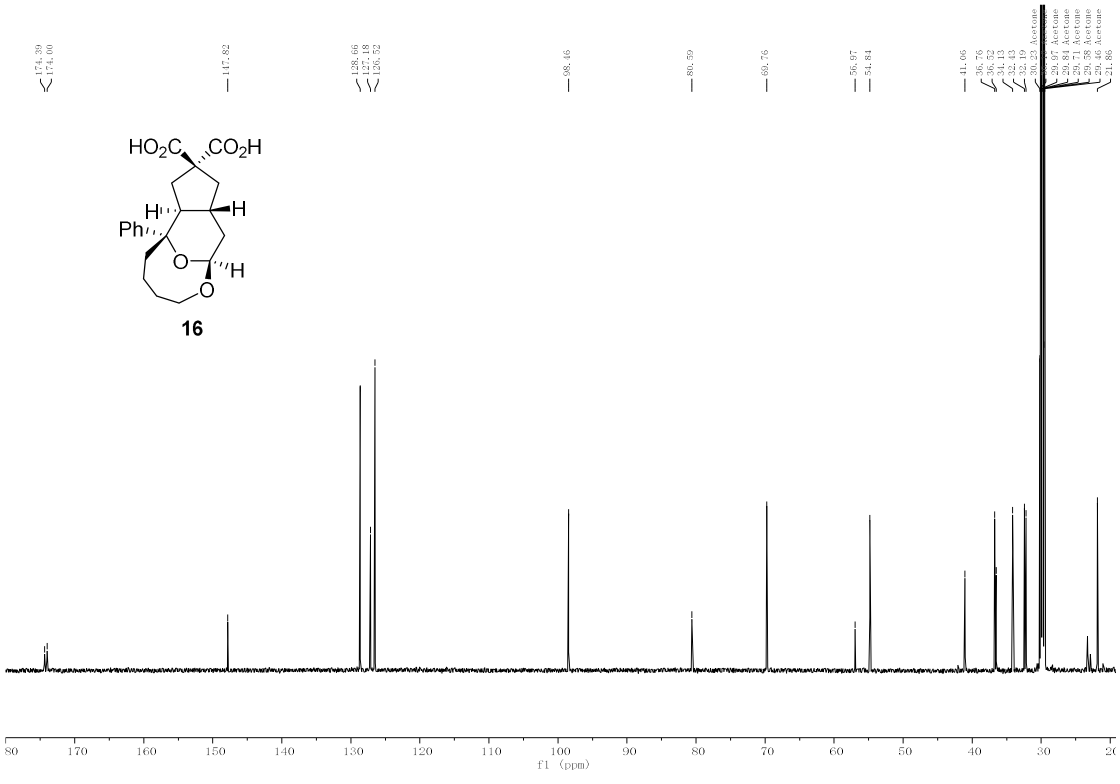

Supplementary Figure 275. ^{1}H NMR spectrum of 17.

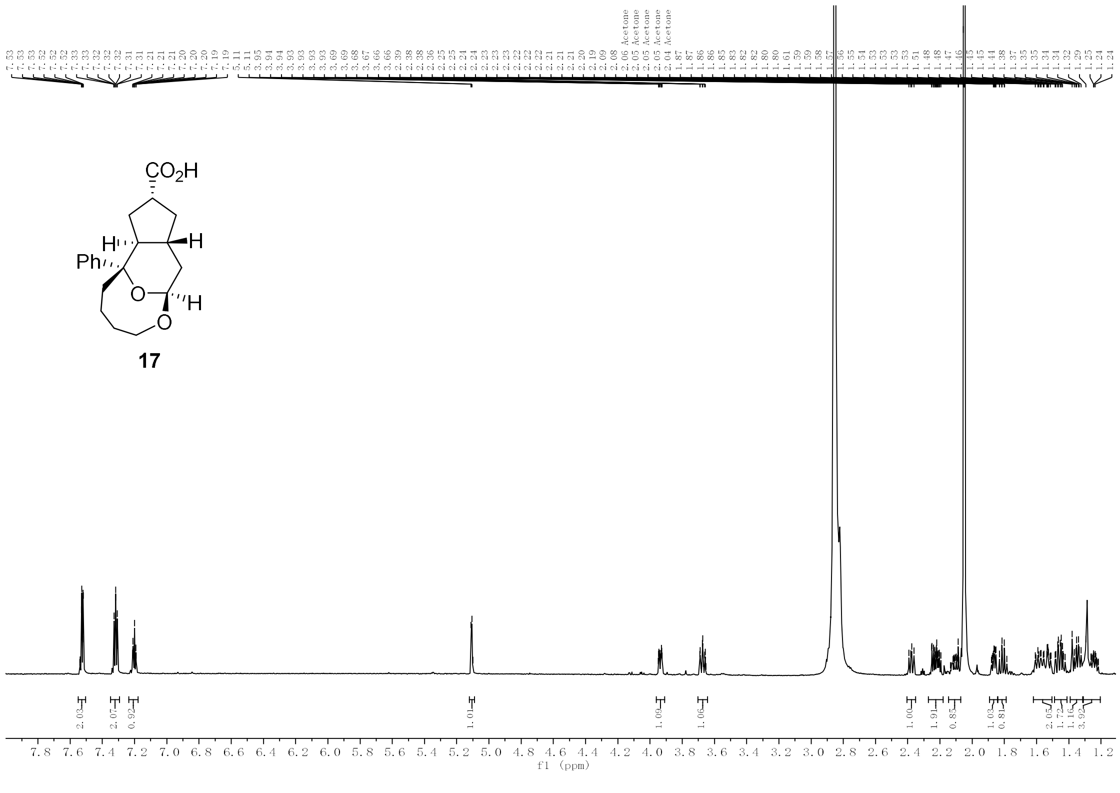

Supplementary Figure 276. ^{13}C NMR spectrum of 17.

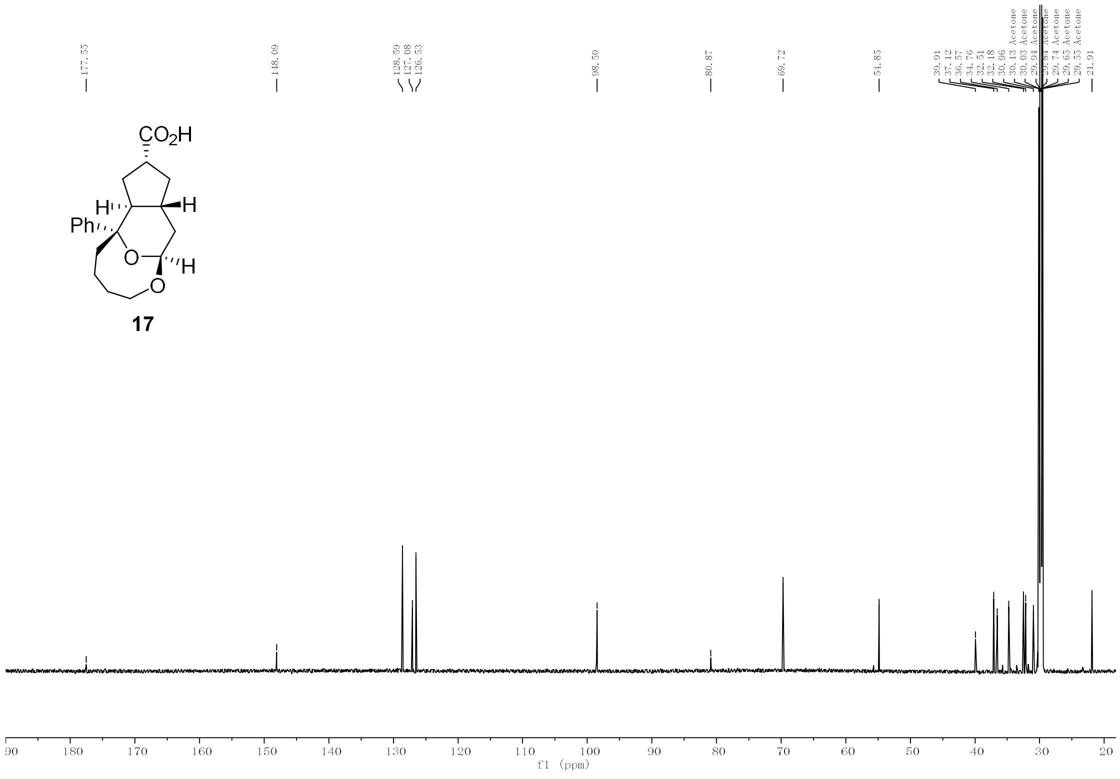

Supplementary Figure 277. ^{1}**H NMR spectrum of 15**.

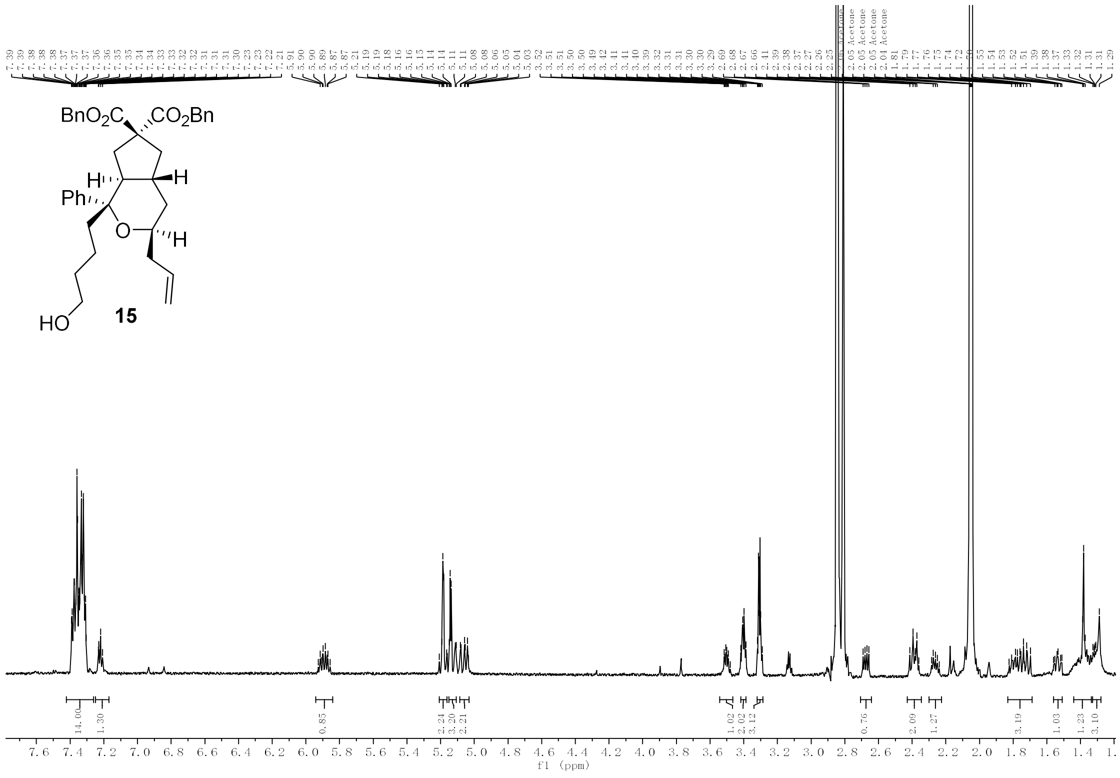

Supplementary Figure 278. ^{13}**C NMR spectrum of 15**.

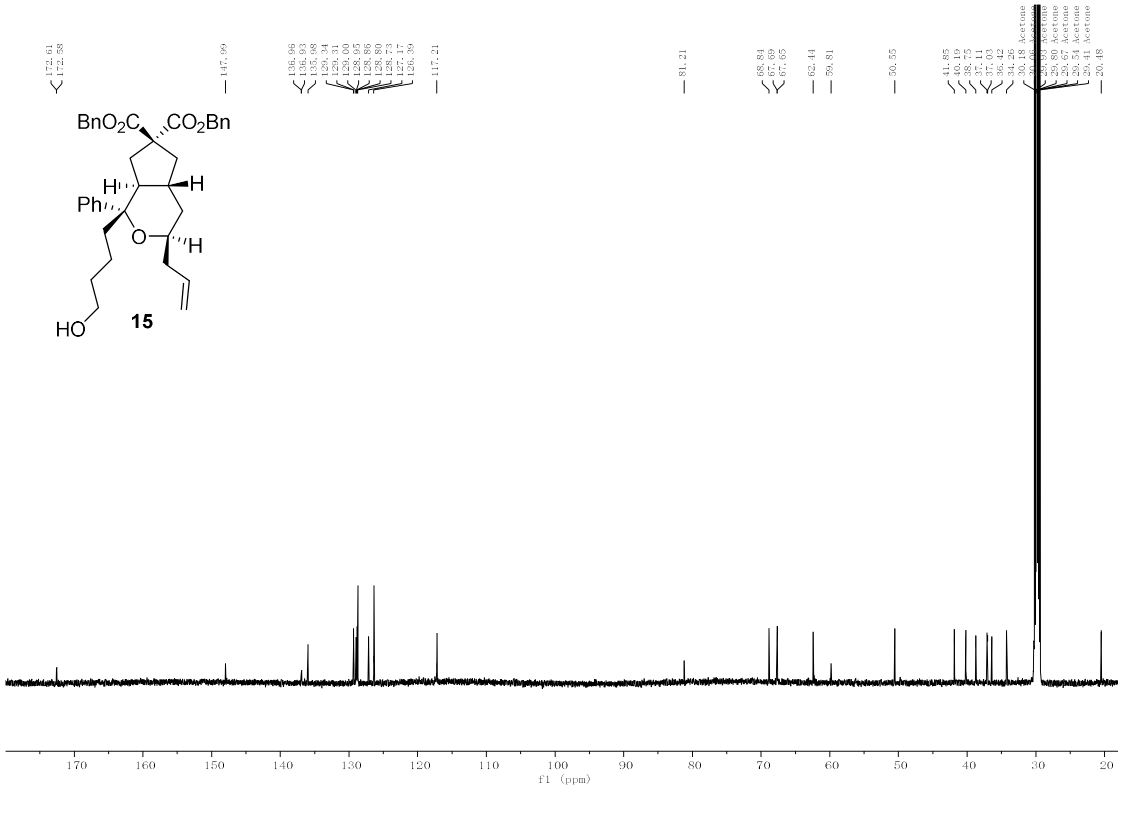

Supplementary Figure 279. [1] H NMR spectrum of 19.

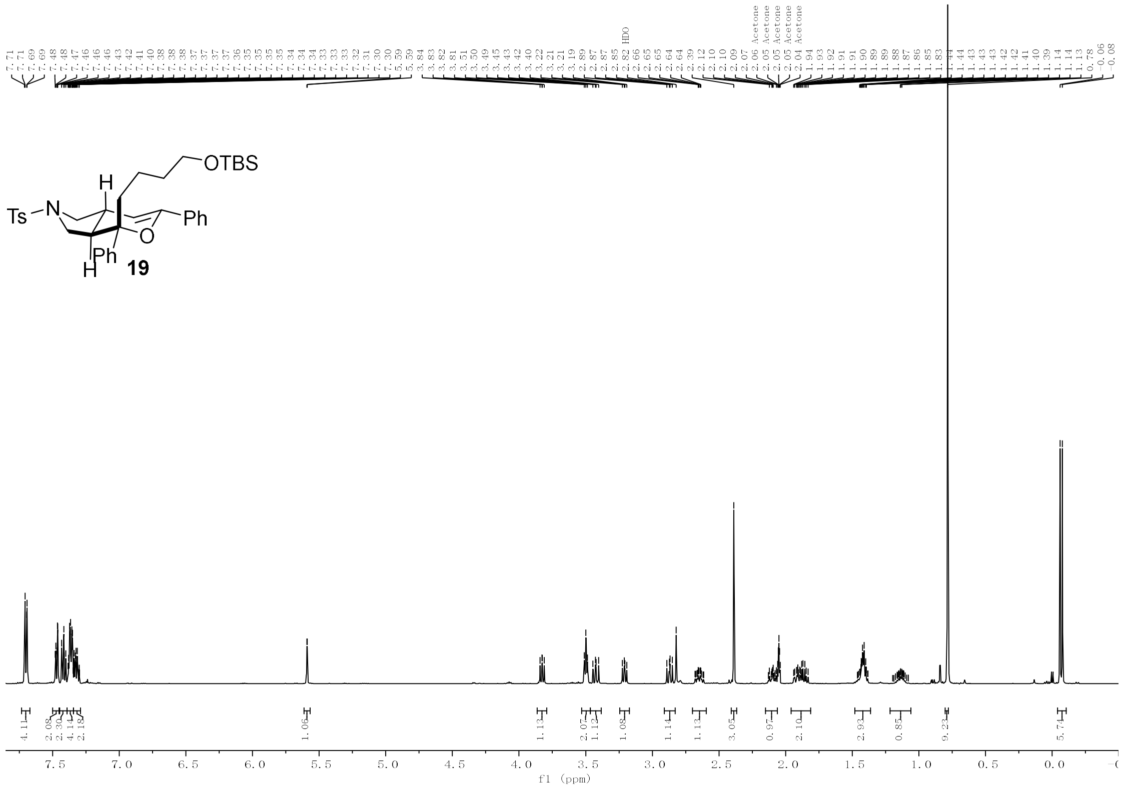

Supplementary Figure 280. [13] C NMR spectrum of 19.

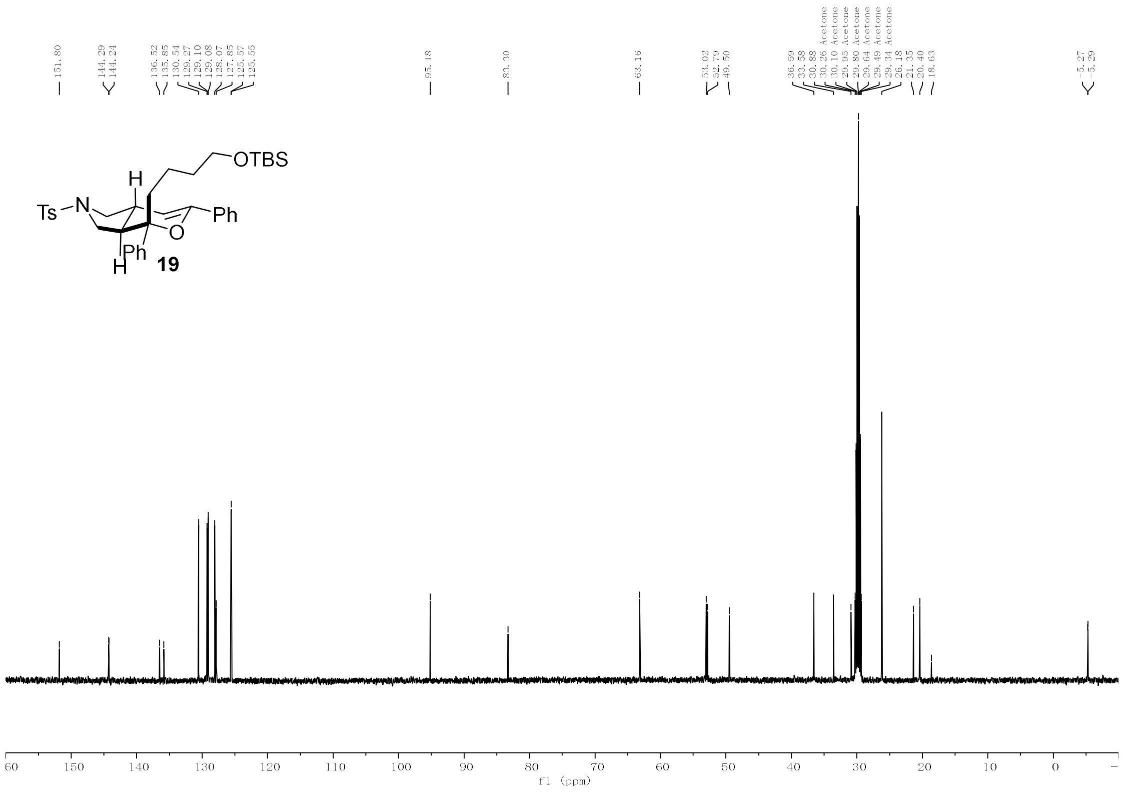

Supplementary Figure 281. ^{1} H NMR spectrum of 5u.

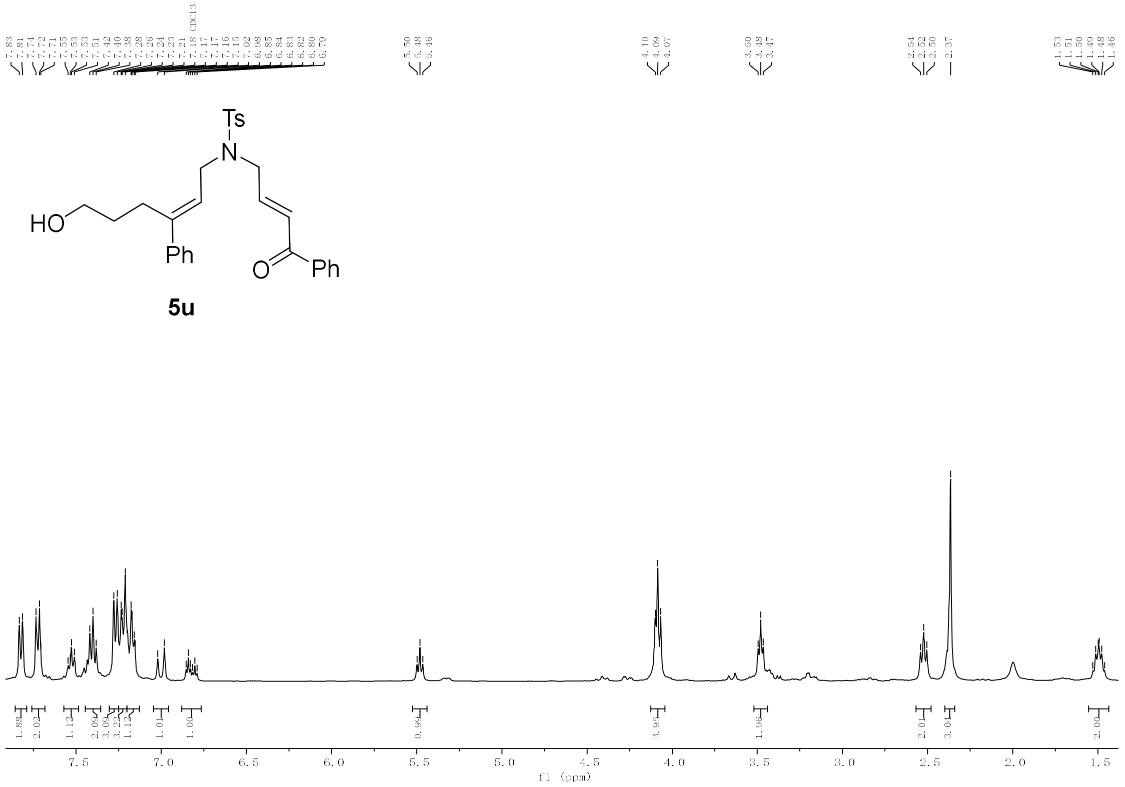

Supplementary Figure 282. ^{13} C NMR spectrum of 5u.

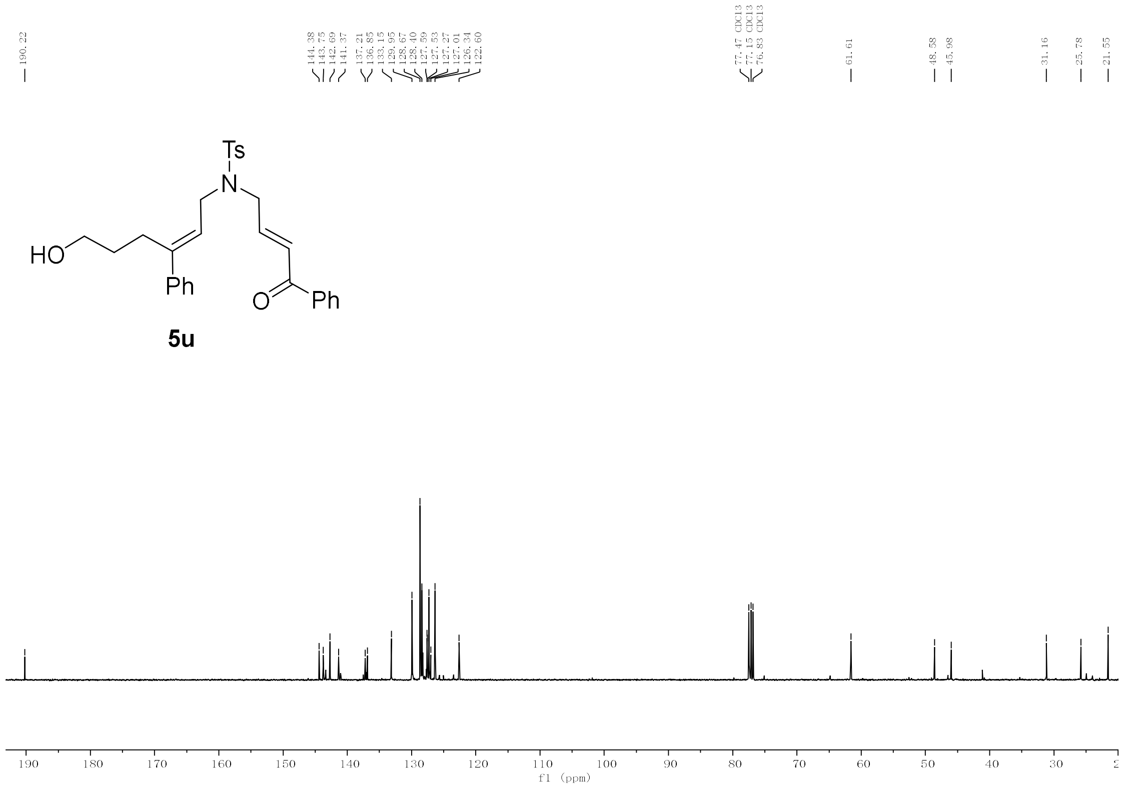

Supplementary Figure 283. ^{1}H NMR spectrum of 5v.

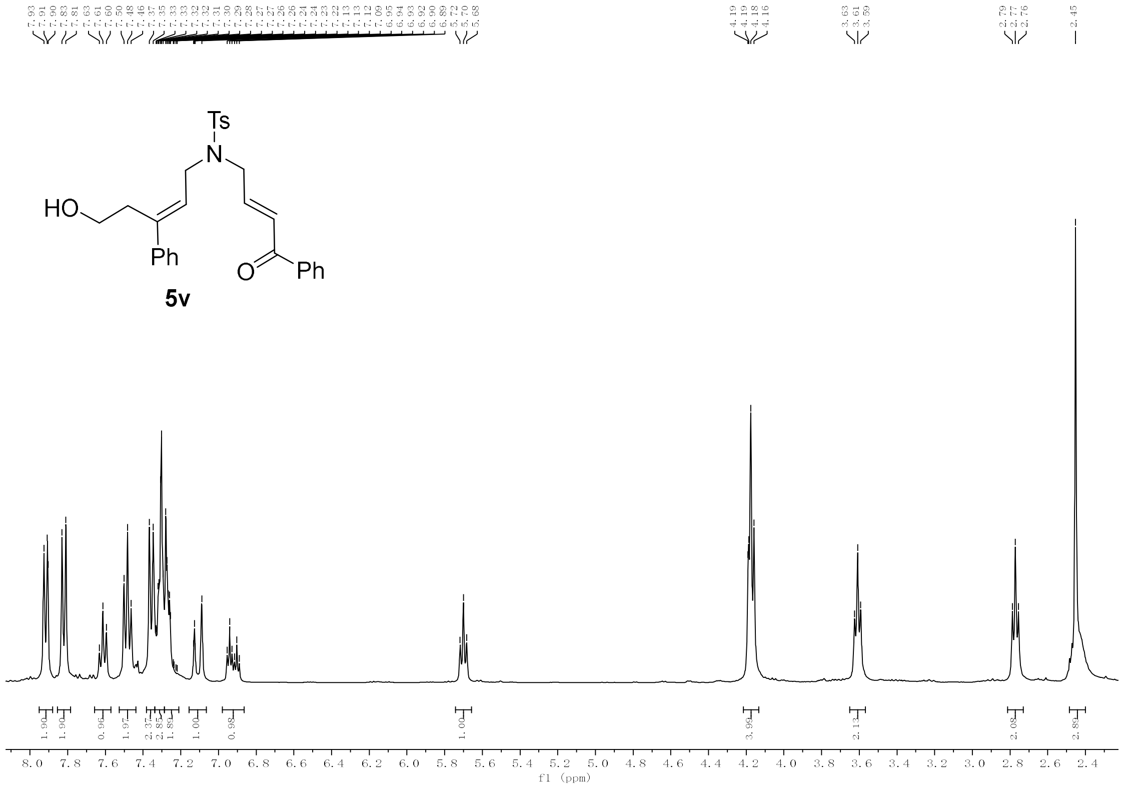

Supplementary Figure 284. ^{13}C NMR spectrum of 5v.

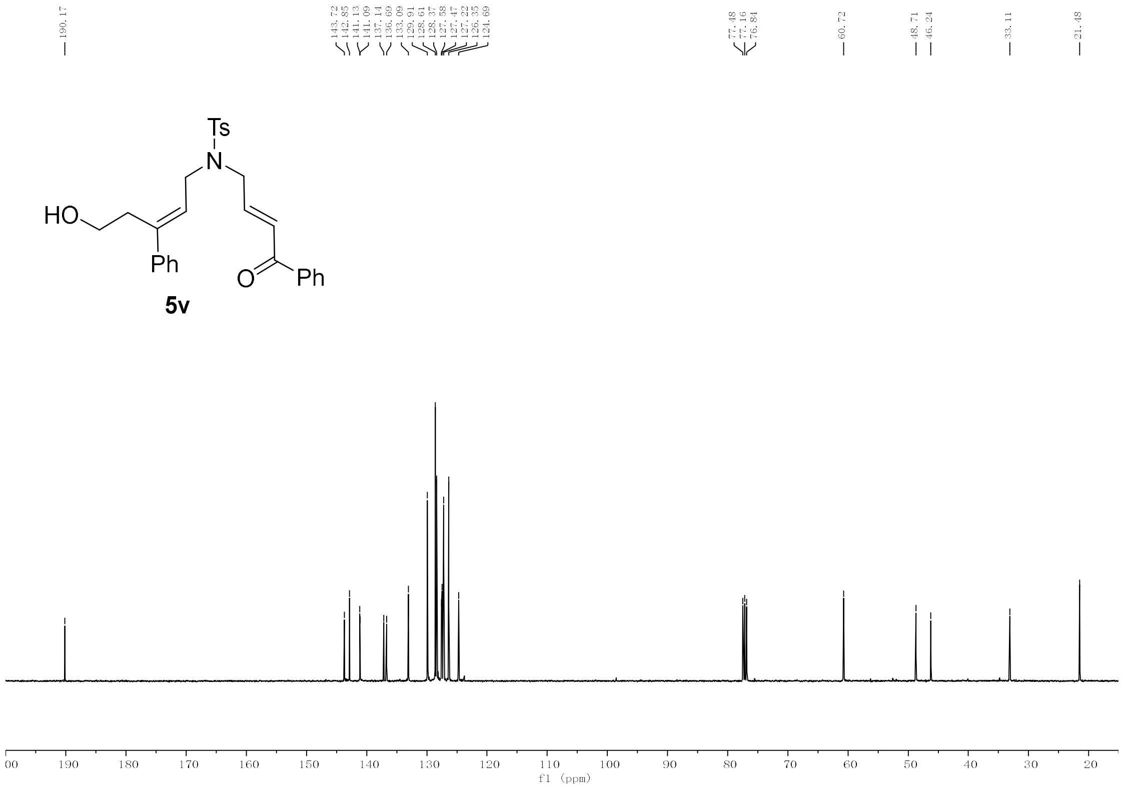

Supplementary Figure 285. [1] H NMR spectrum of 5w.

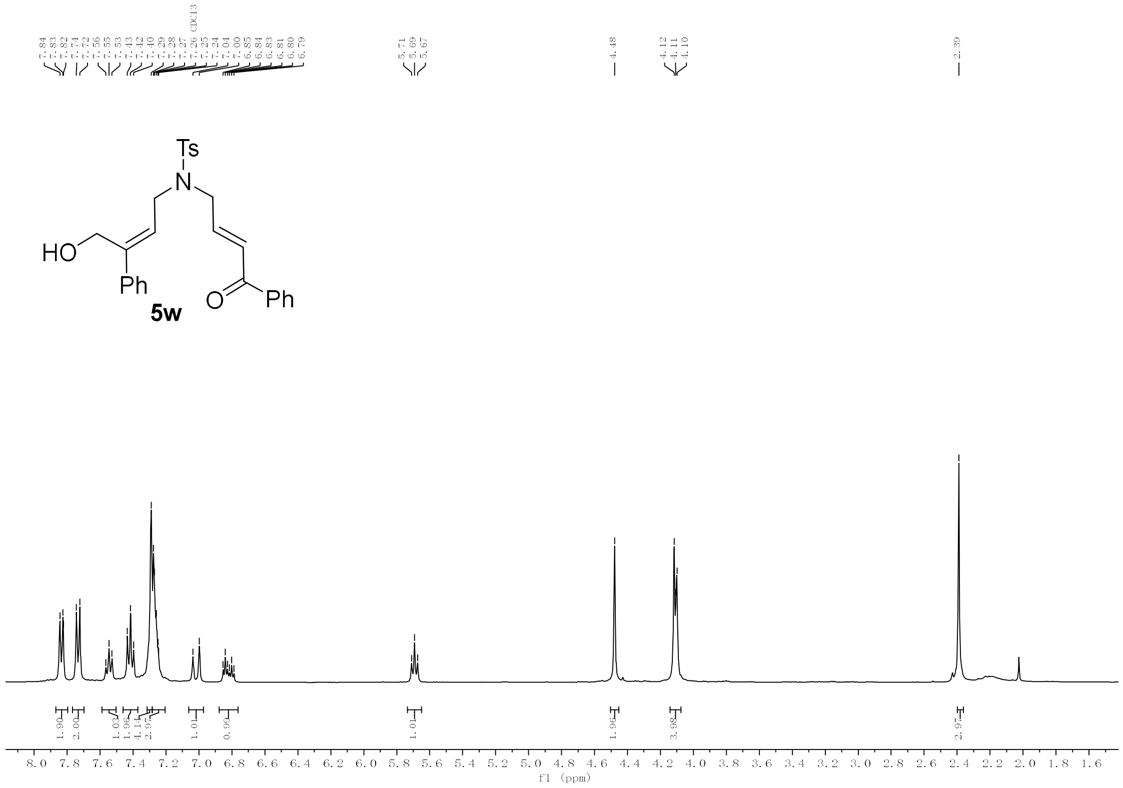

Supplementary Figure 286. [13] C NMR spectrum of 5w.

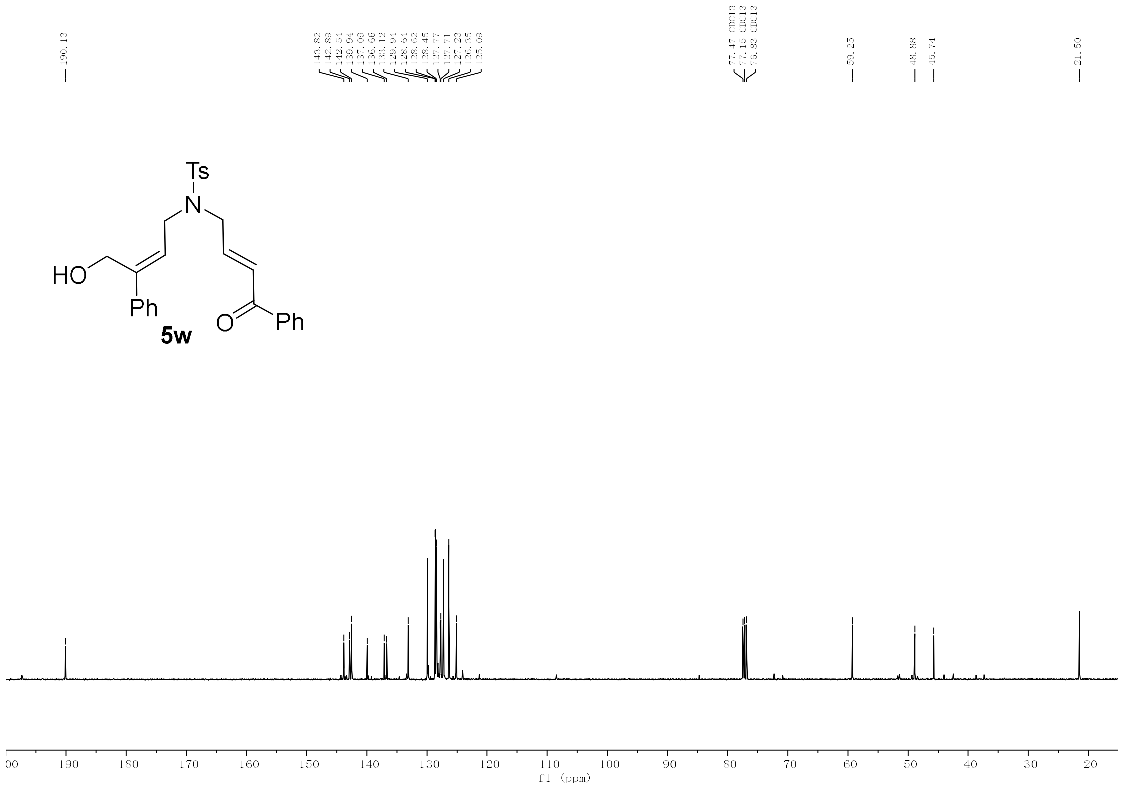

Supplementary Figure 287. [1] H NMR spectrum of 7u.

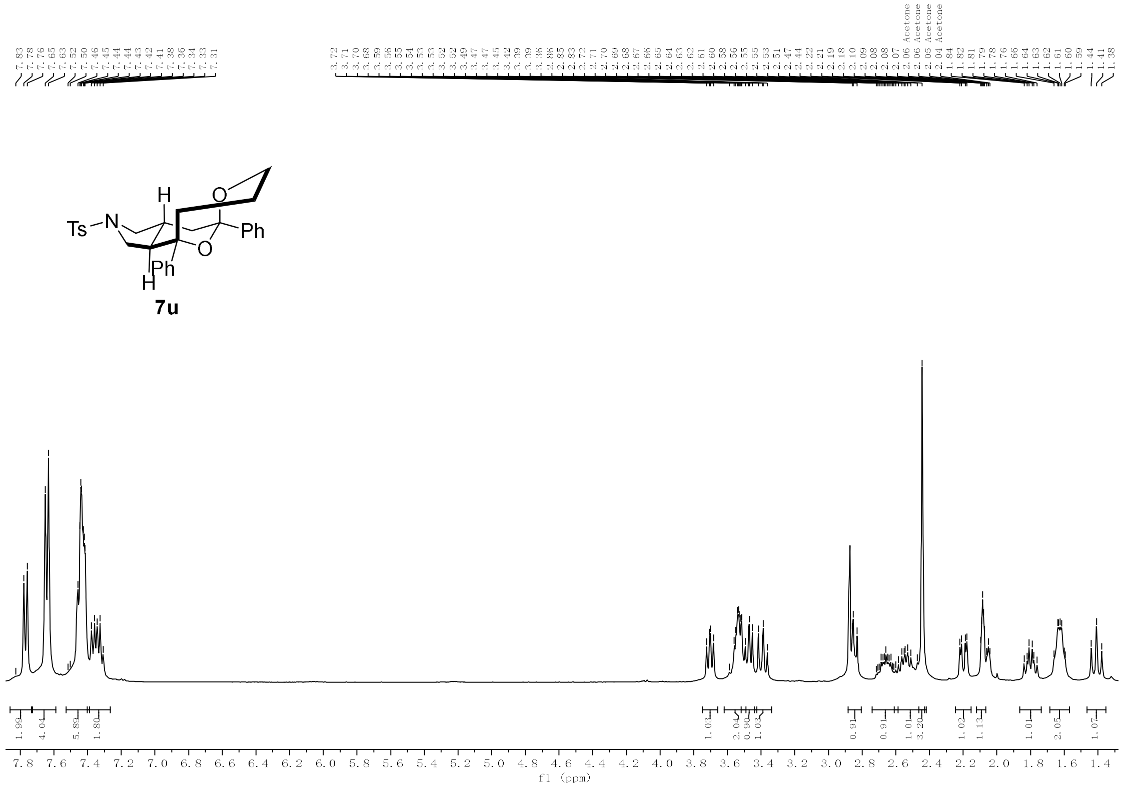

Supplementary Figure 288. [13] C NMR spectrum of 7u.

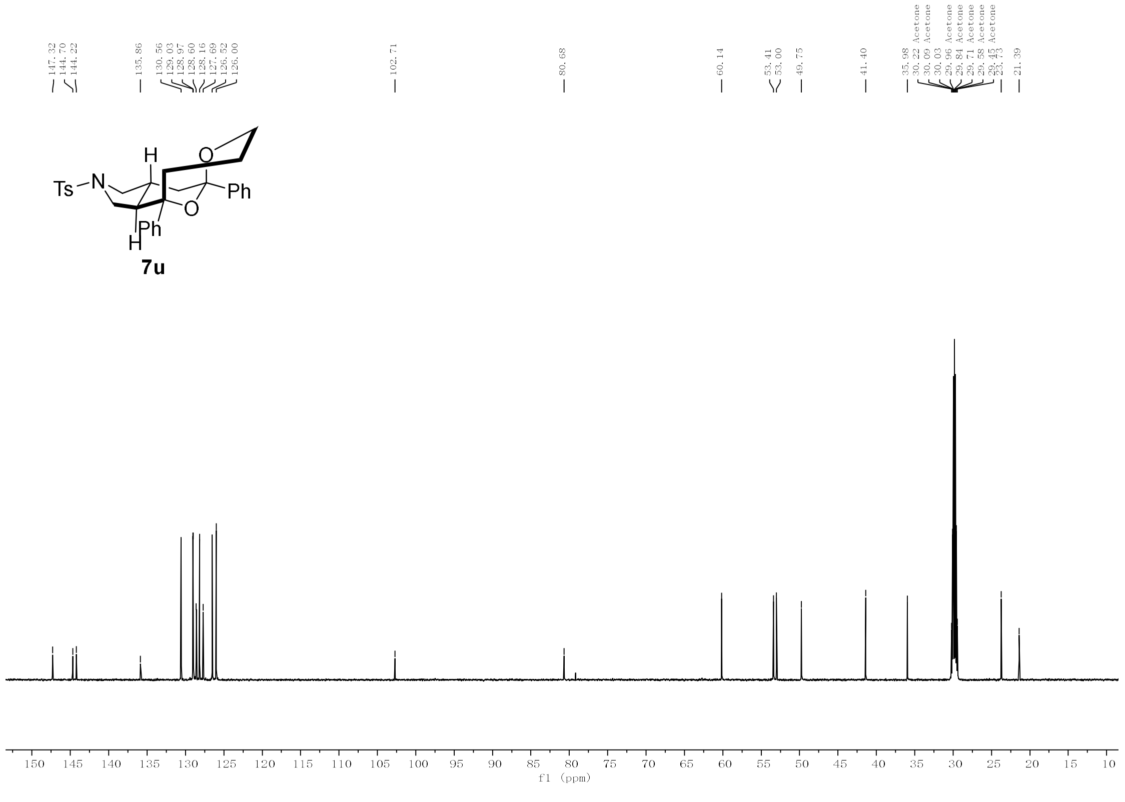

Supplementary Figure 289. [1] H NMR spectrum of 7v.

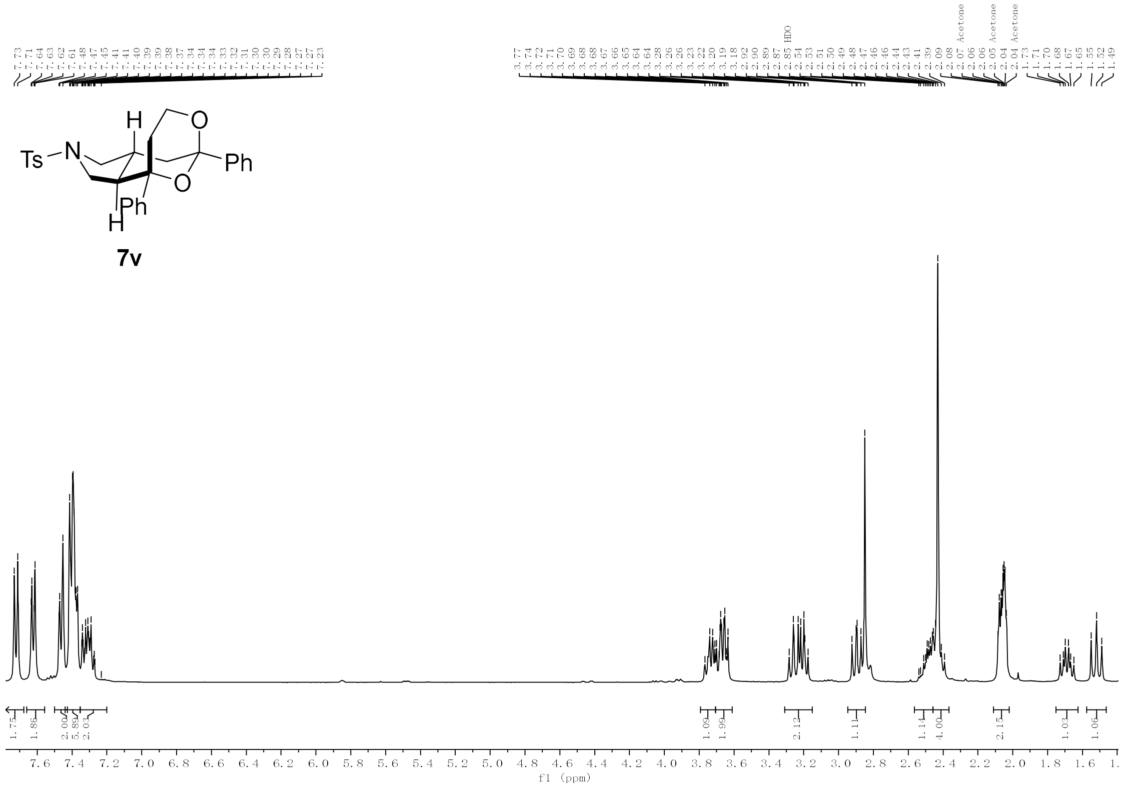

Supplementary Figure 290. [13] C NMR spectrum of 7v.

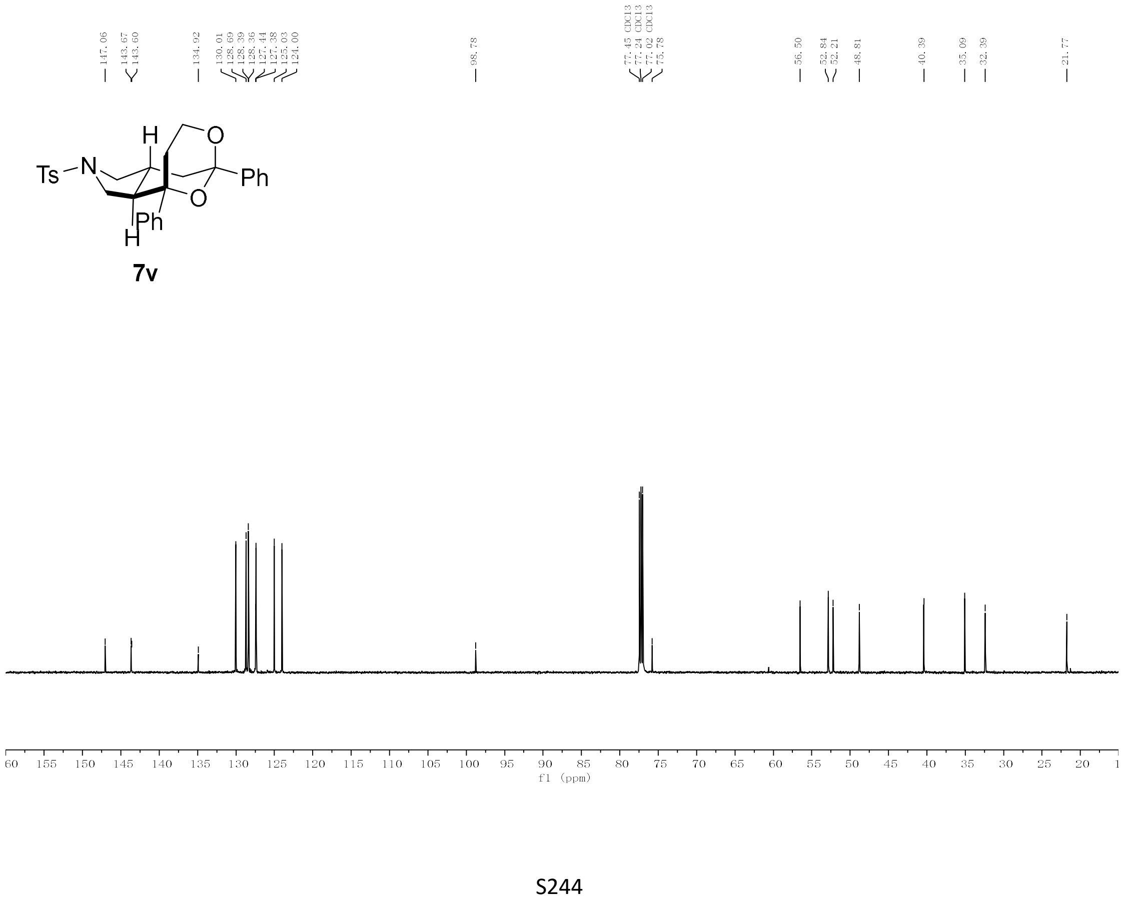

Supplementary Figure 291. [1] H NMR spectrum of 7w.

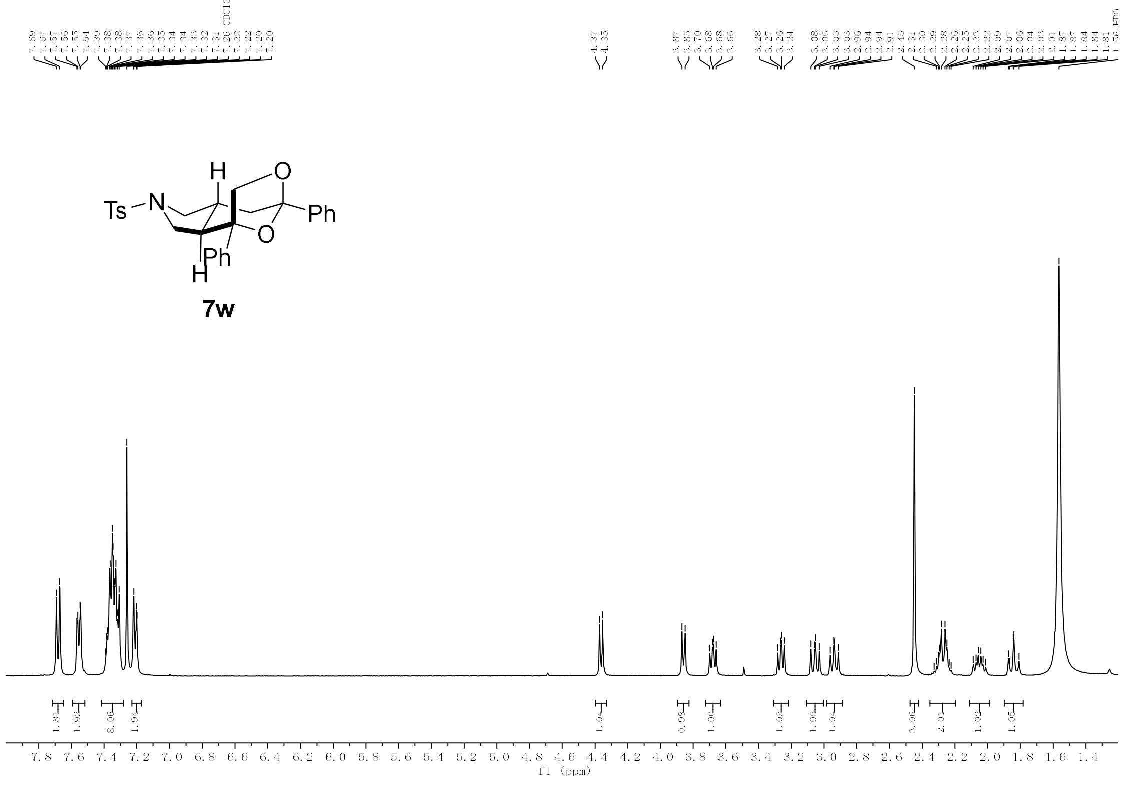

Supplementary Figure 292. [13] C NMR spectrum of 7w.

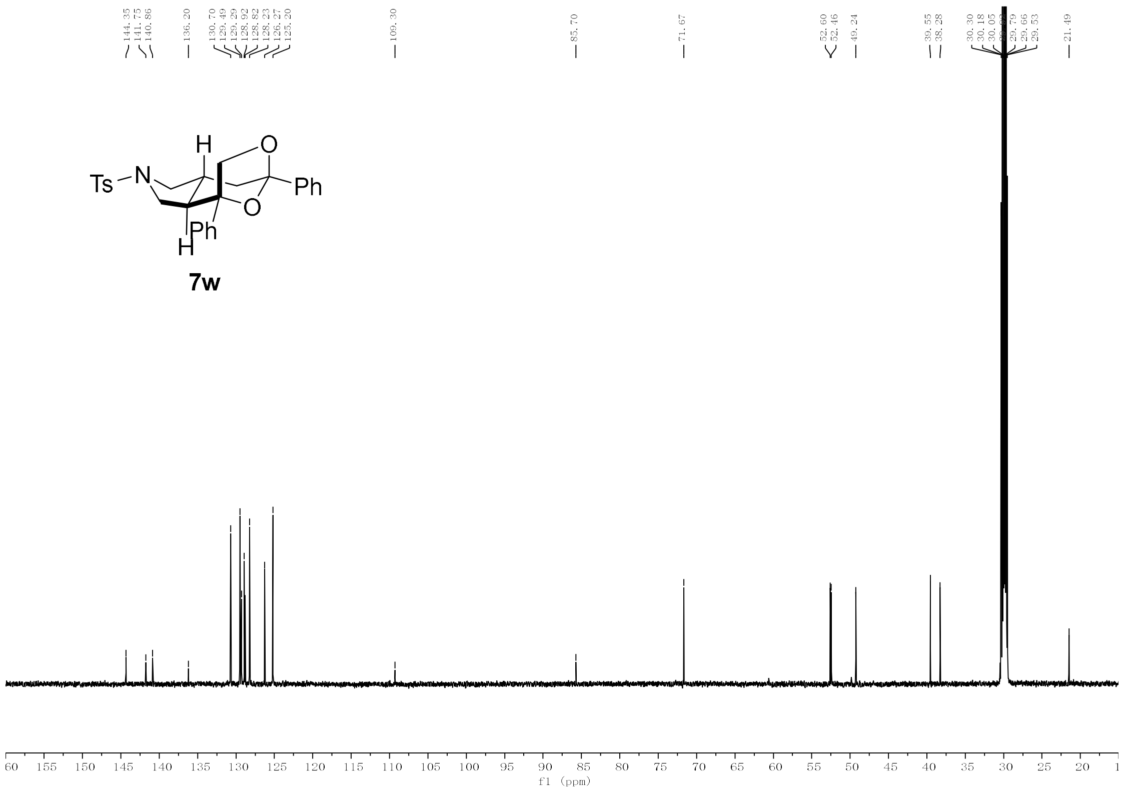

Supplementary Figure 293. [1] H NMR spectrum of 7u'.

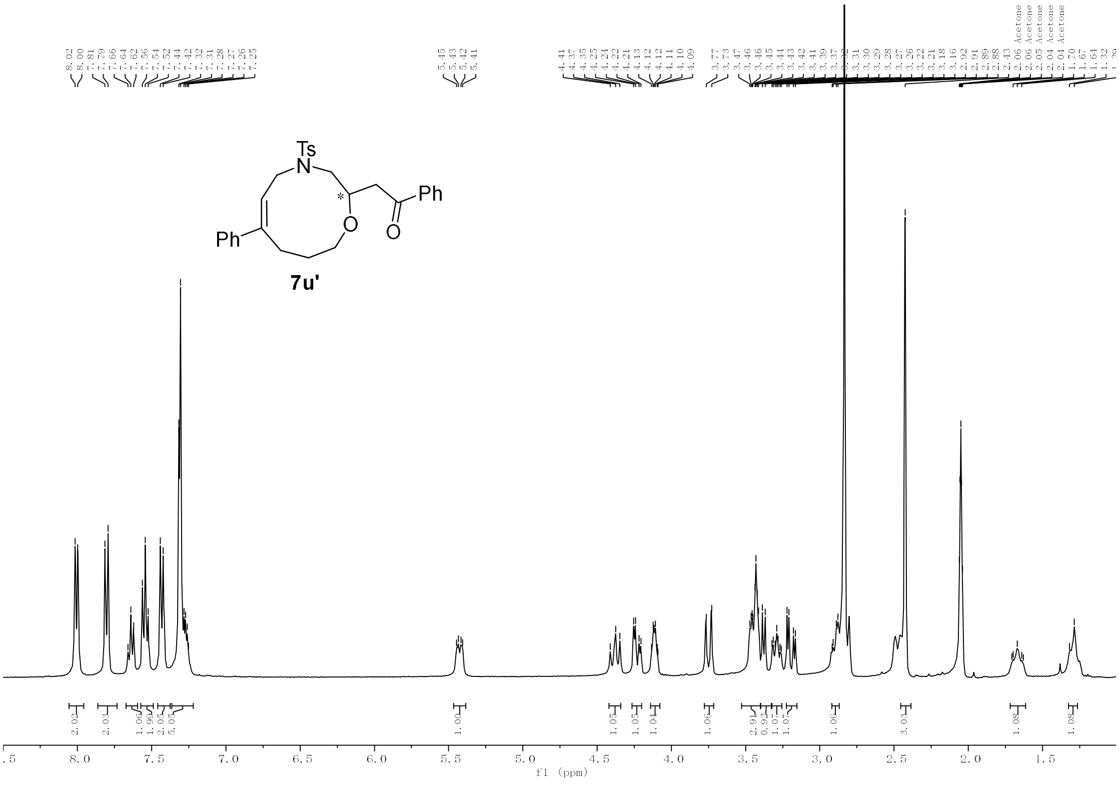

Supplementary Figure 294. [13] C NMR spectrum of 7u'.

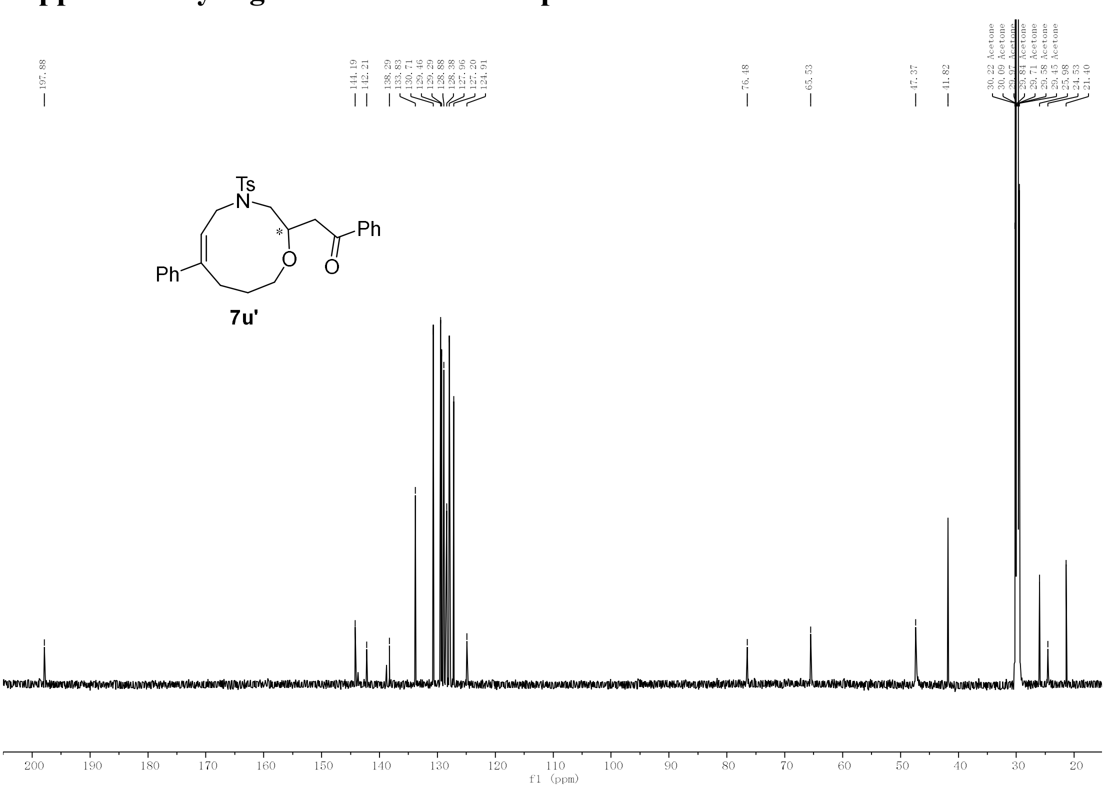

Supplementary Figure 295. [1] H NMR spectrum of 7v'.

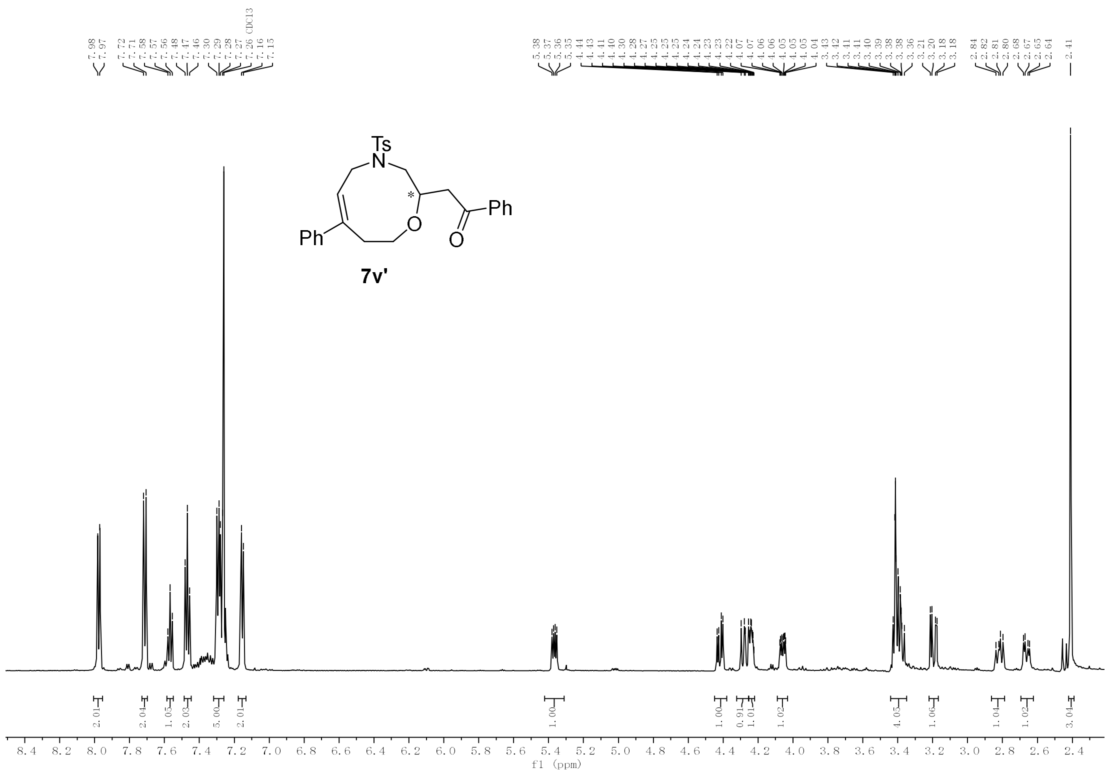

Supplementary Figure 296. [13] C NMR spectrum of 7v'.

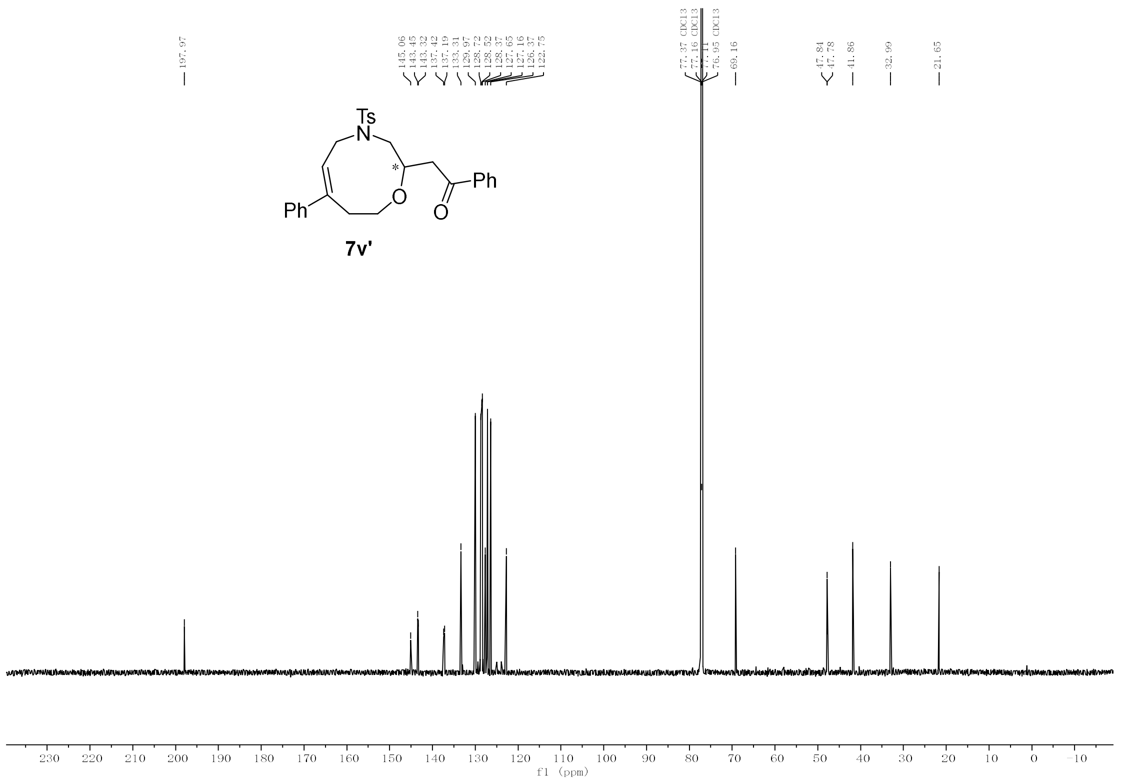

Supplementary Figure 297. ¹ H NMR spectrum of 7w'.

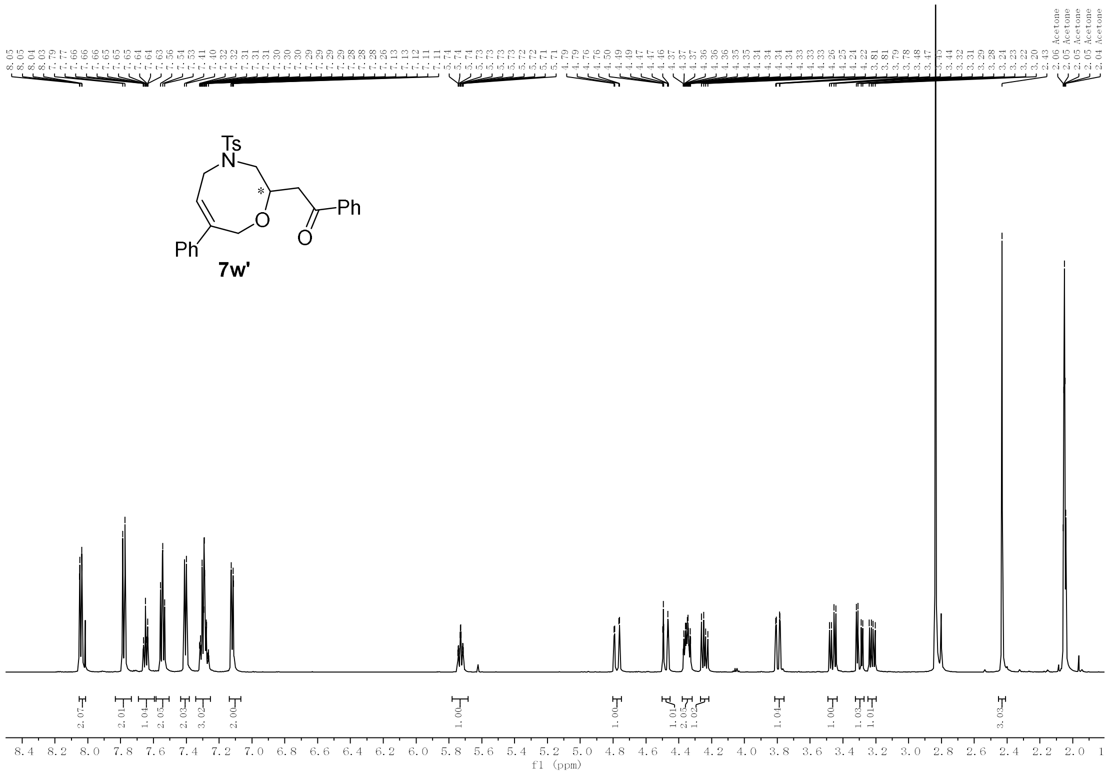

Supplementary Figure 298. ¹³ C NMR spectrum of 7w'.

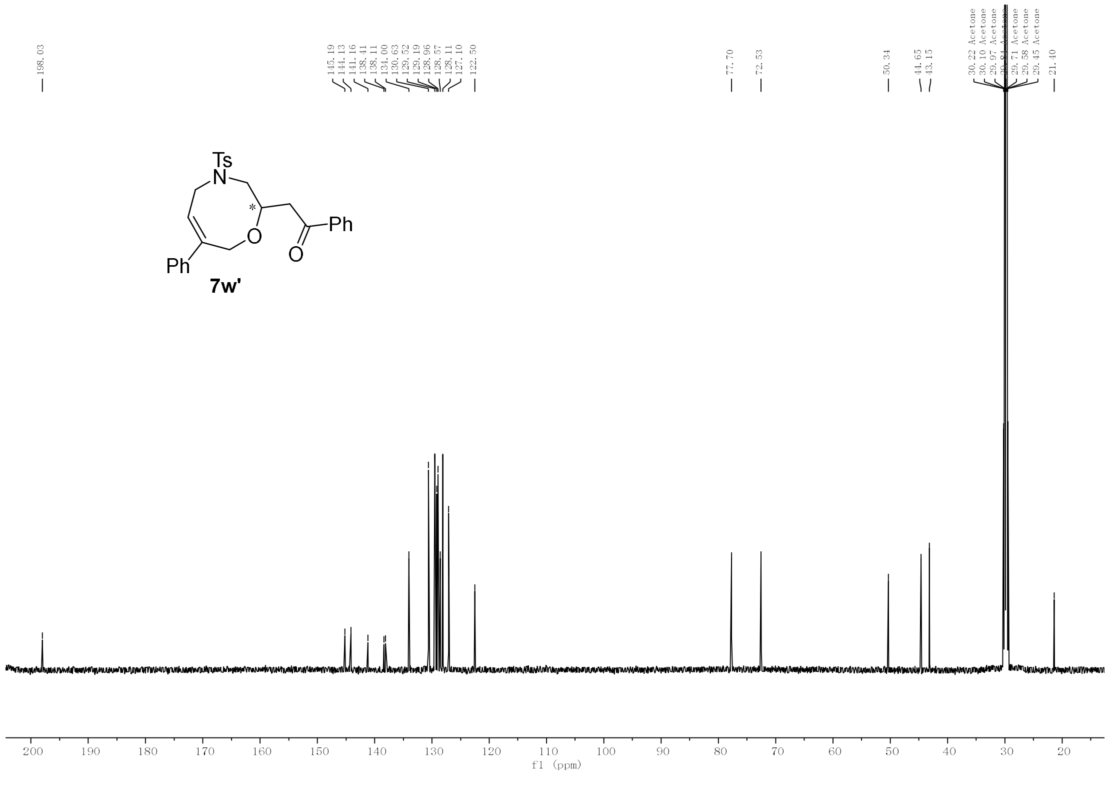

Supplementary Figure 299. [1] H NMR spectrum of 7w".

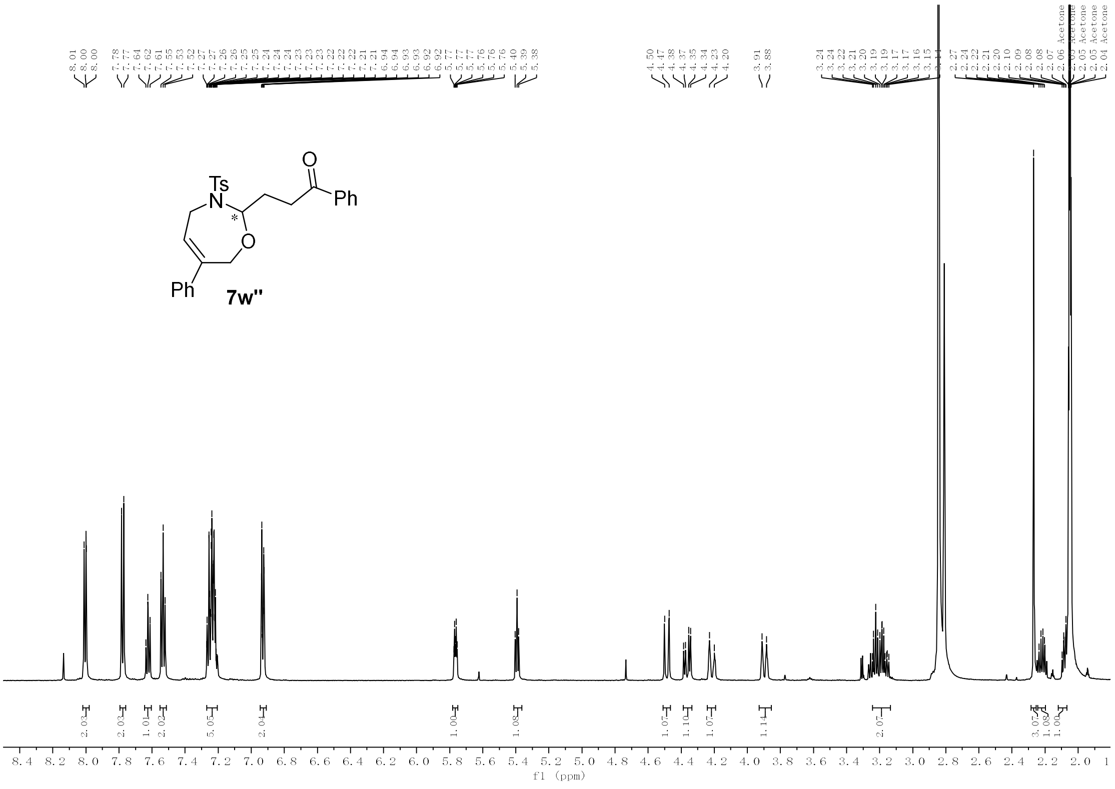

Supplementary Figure 300. [13] C NMR spectrum of 7w".

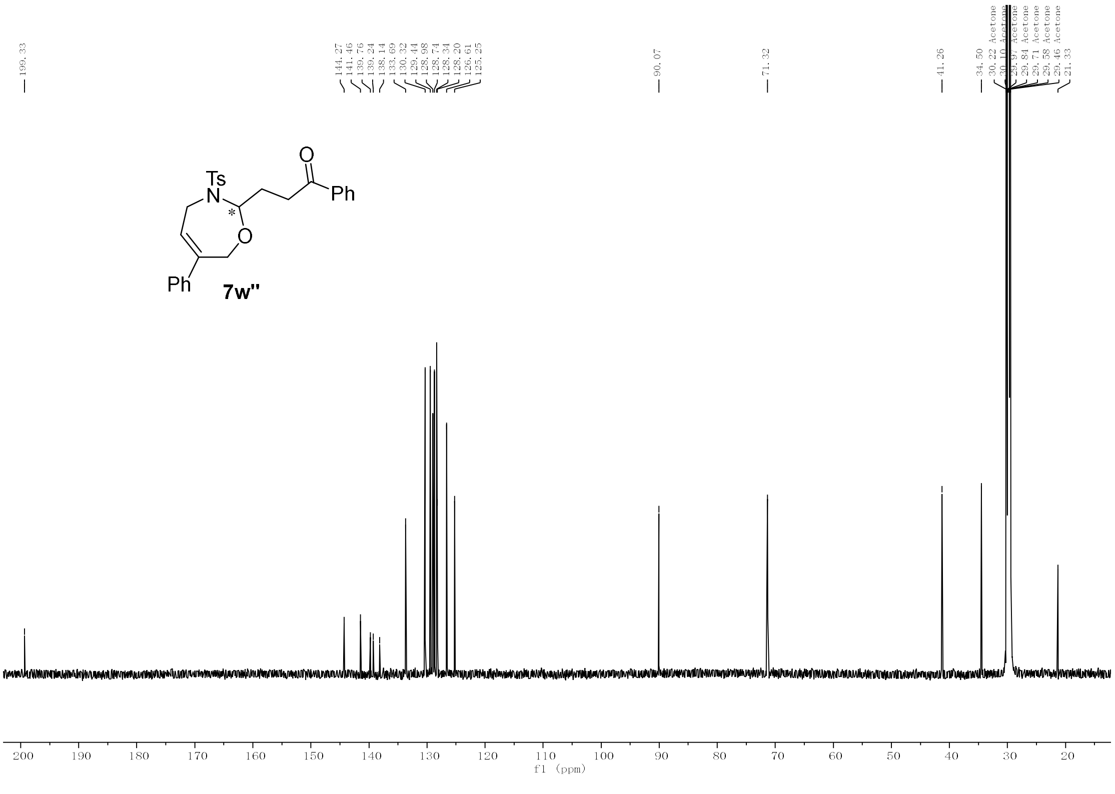

Supplementary Figure 301. ^{19}F NMR spectrum of 9b

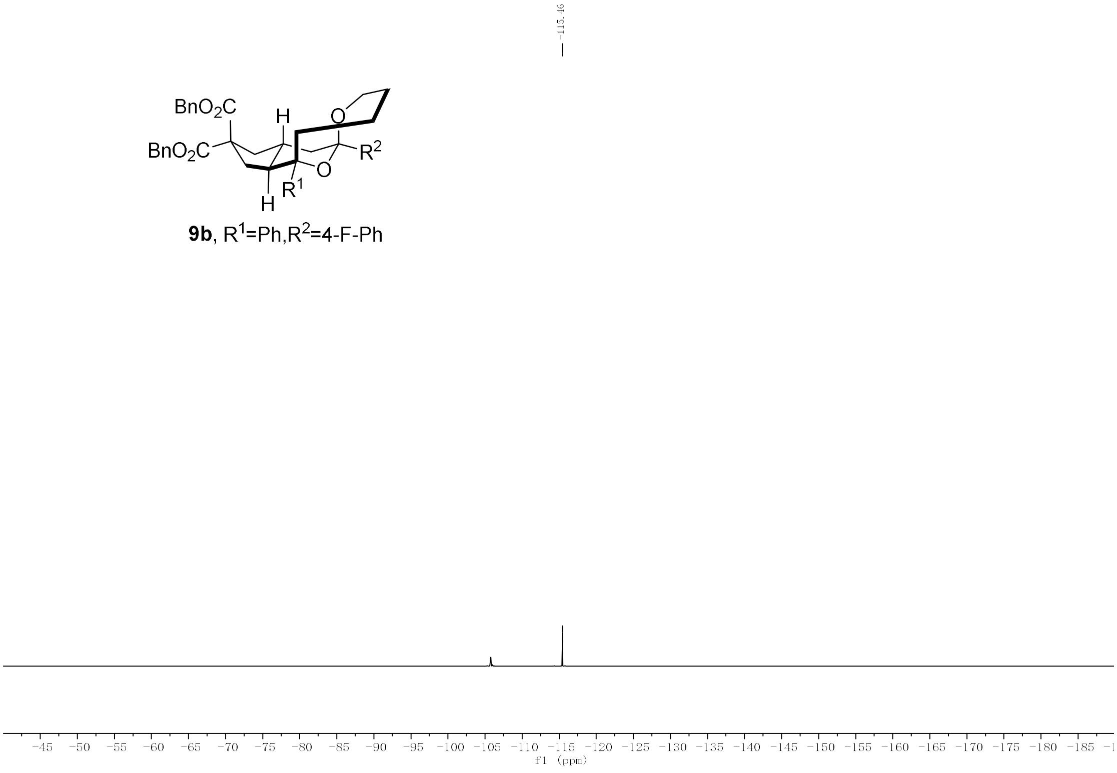

Supplementary Figure 302. ^{19}F NMR spectrum of 9j

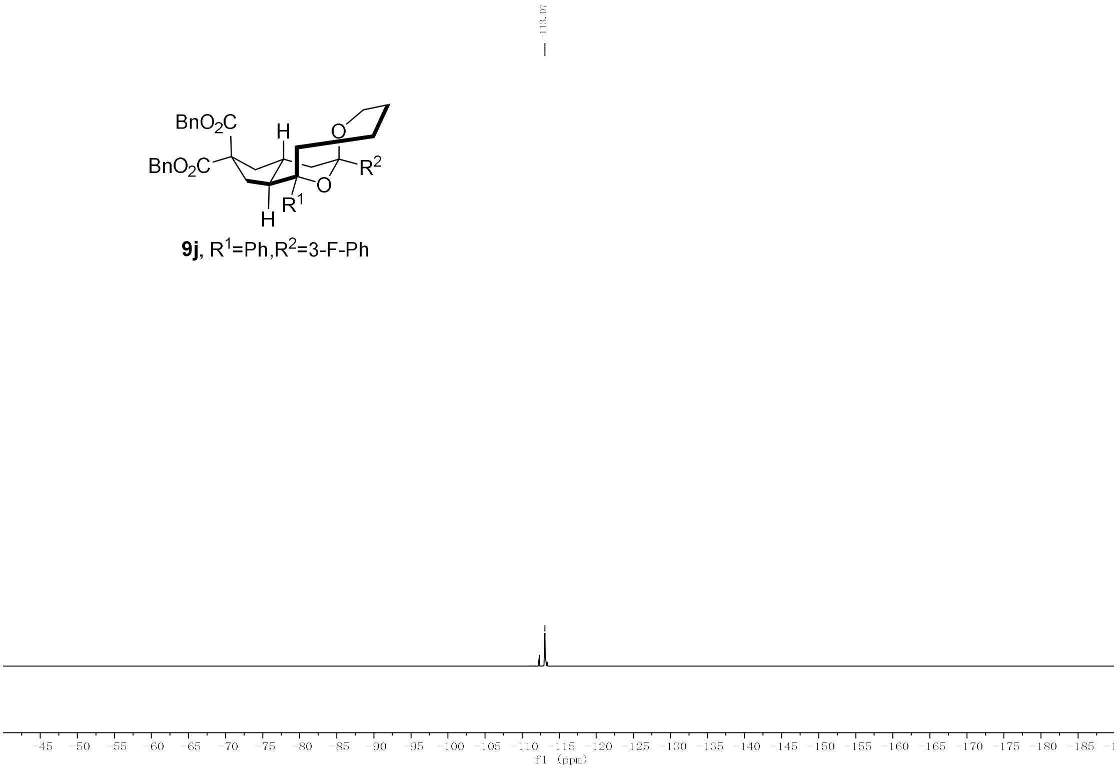

Supplementary Figure 303. ^{19}F NMR spectrum of 11b.

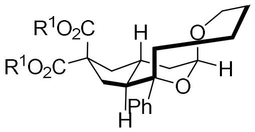

11b, R^1=4-F-Bn

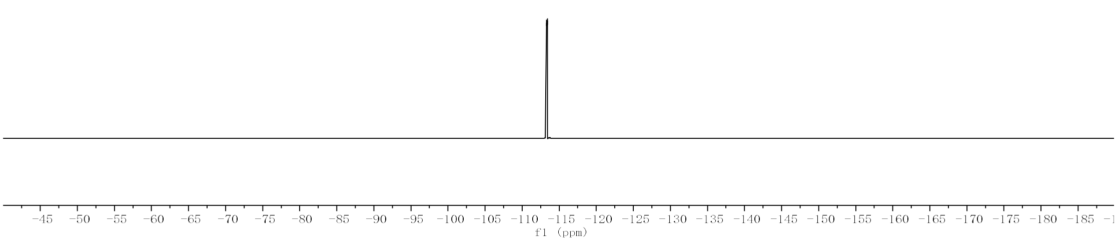

Supplementary Figure 304. ^{19}F NMR spectrum of 11g

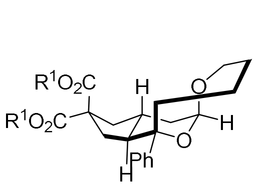

11g, R^1=4-CF_3-Bn

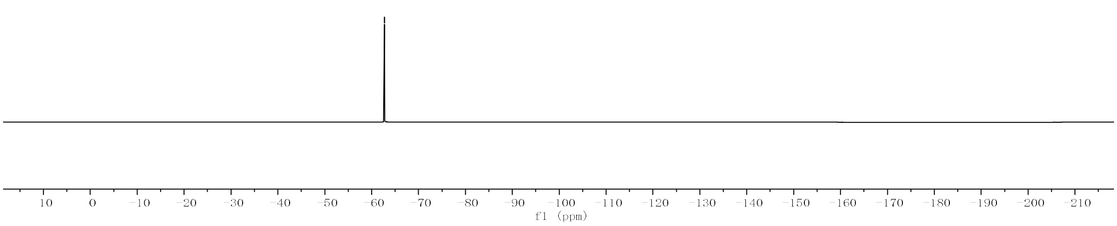

Supplementary Figure 305. ^{19}F NMR spectrum of 11h.

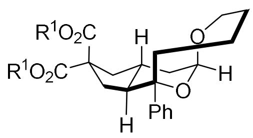

11h, R^1=4-F-Ph

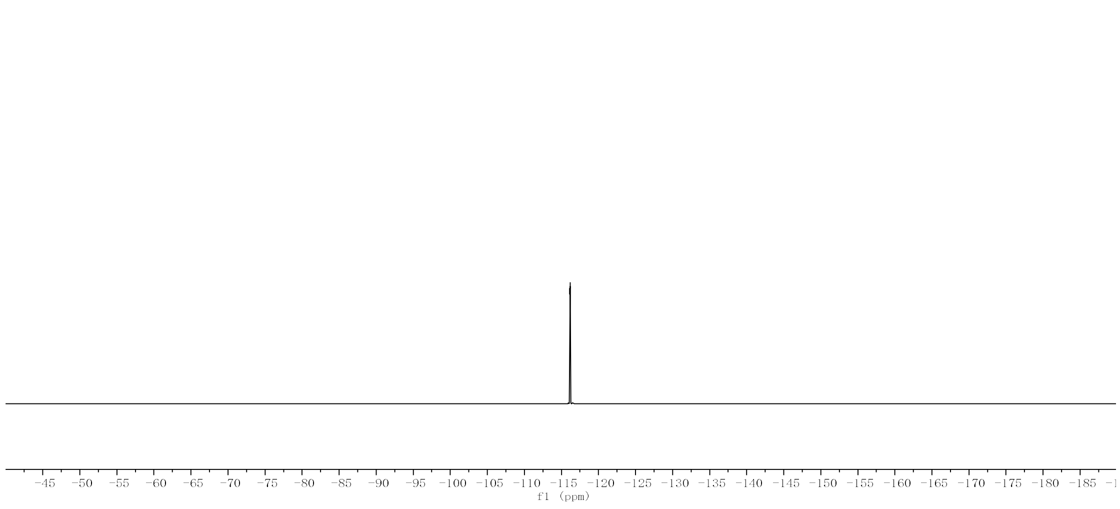

2D NMR spectra for selected compounds

Supplementary Figure 306. ^{1}H-^{1}H COSY for 7a in CDCl$_3$.

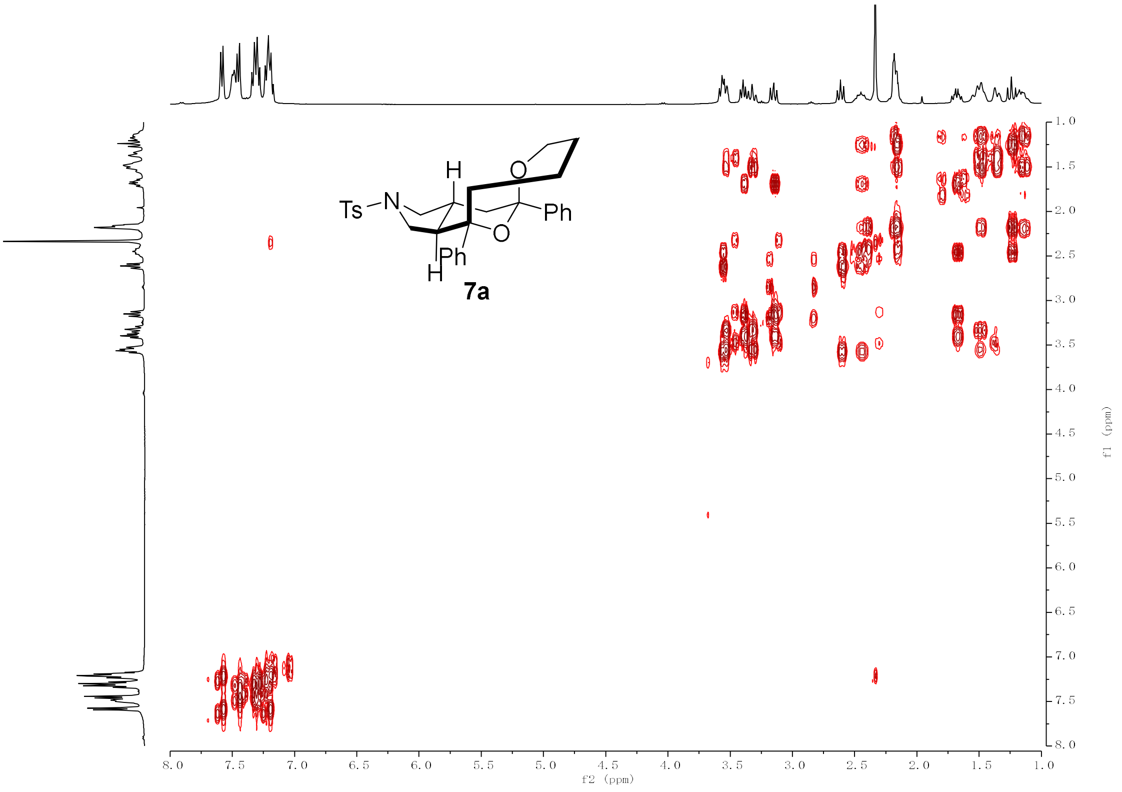

Supplementary Figure 307. ^{1}H-^{13}H ROESY for 7a in CDCl$_3$

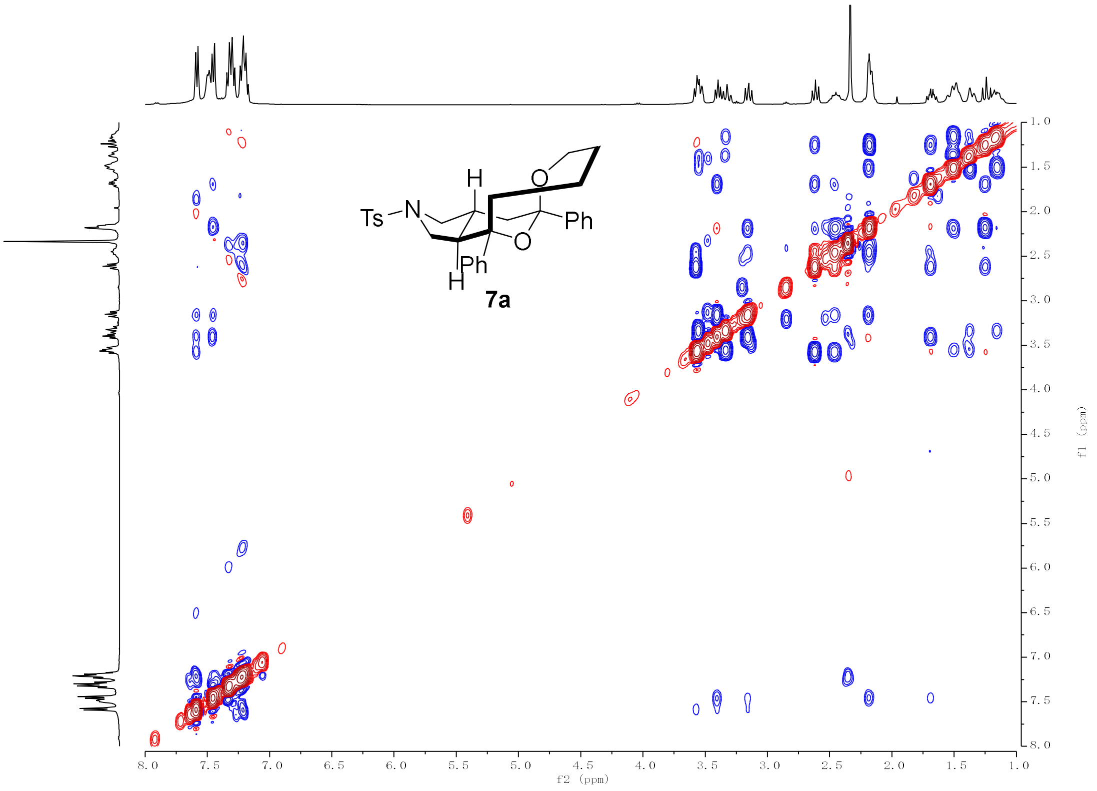

Supplementary Figure 308. ^1H-^{13}C HSQC for 7a in $CDCl_3$

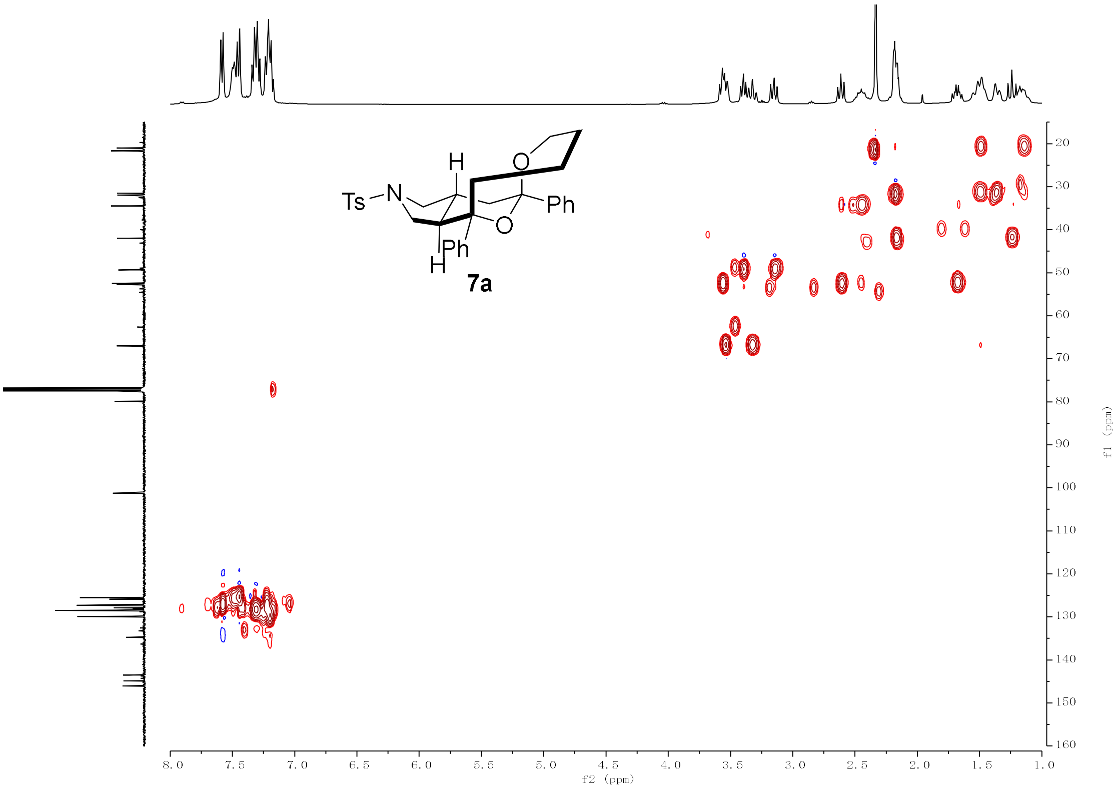

Supplementary Figure 309. ^1H-^{13}C HMBC for 7a in $CDCl_3$

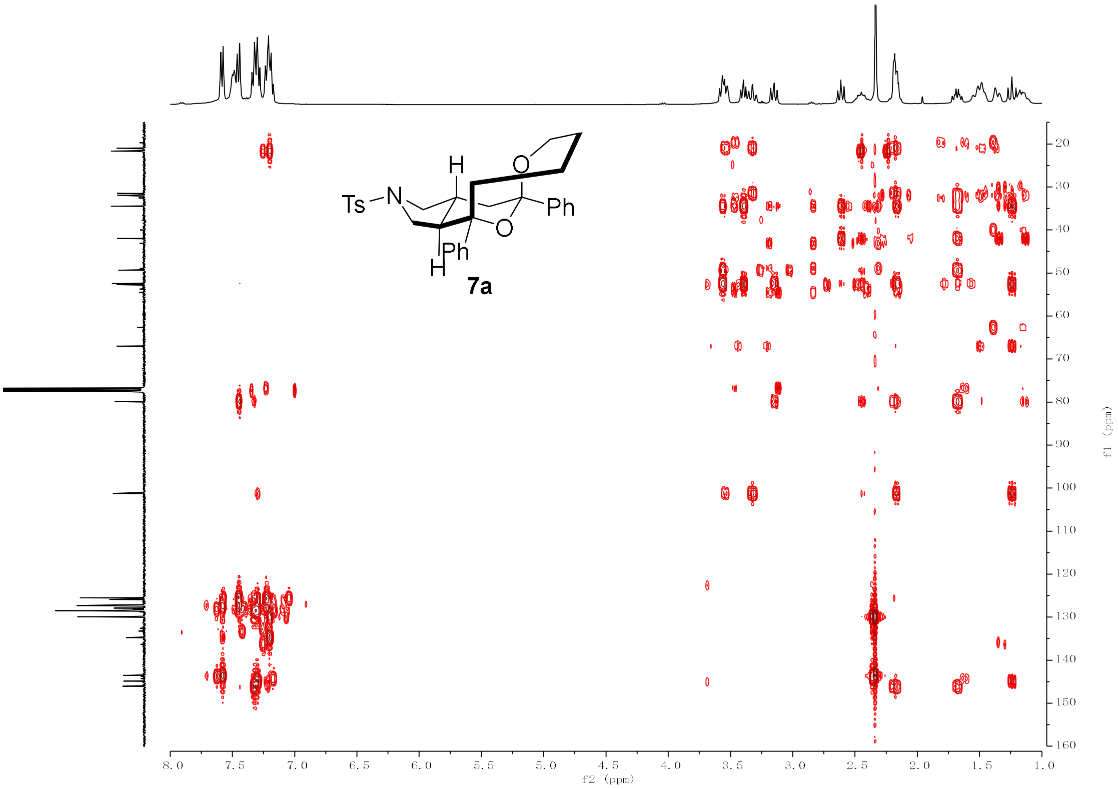

Supplementary Figure 310. ^{1}H-^{1}H COSY for 7h in CDCl₃

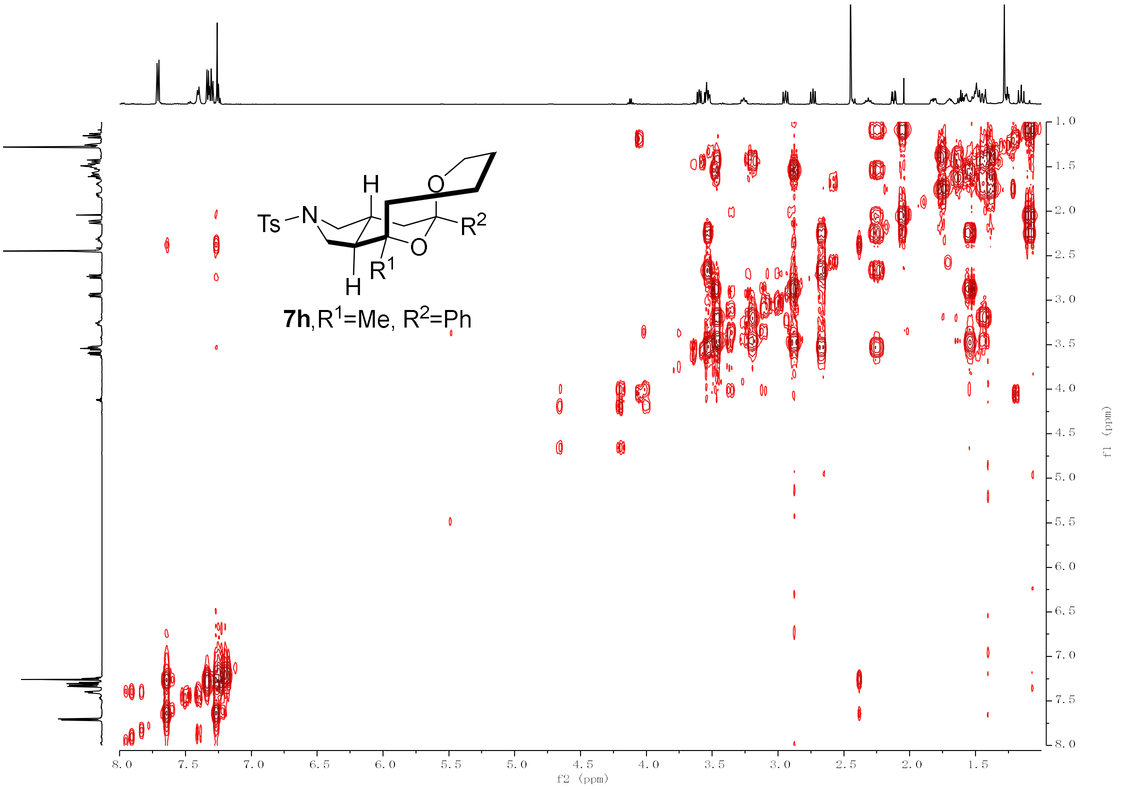

Supplementary Figure 311. ^{1}H-^{1}H ROESY for 7h in CDCl₃

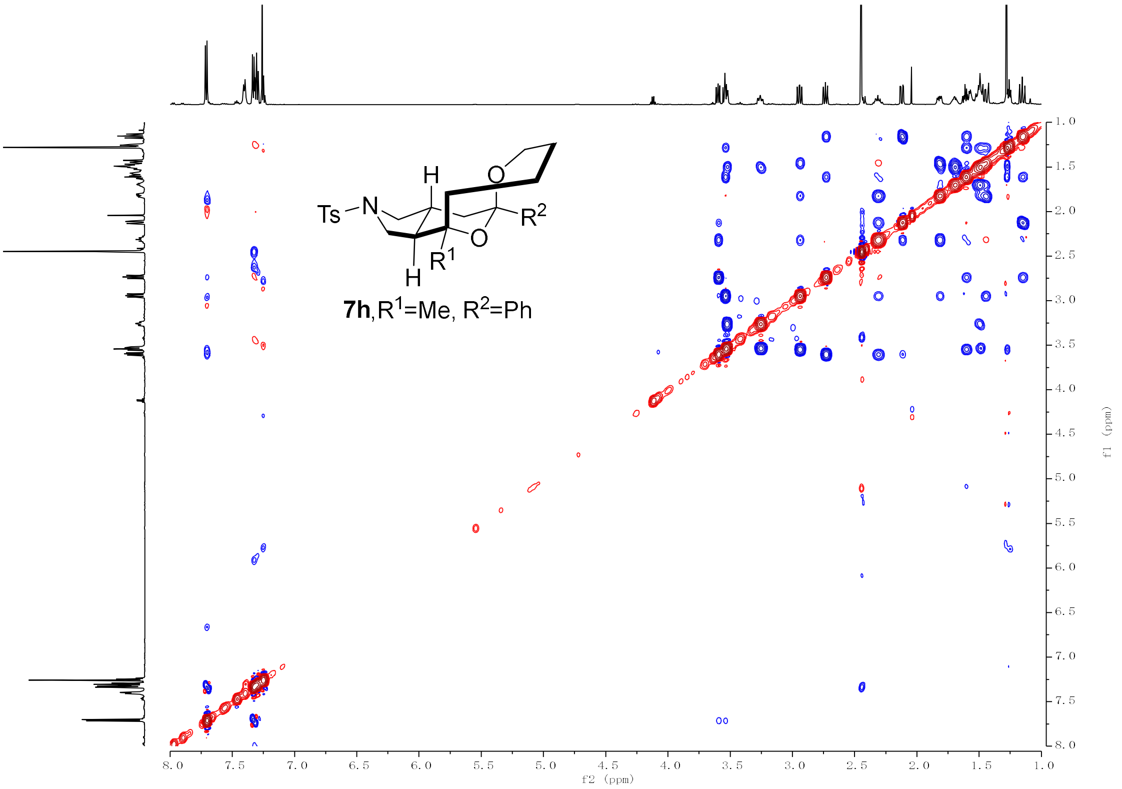

Supplementary Figure 312. ^{1}H-^{13}C HSQC for 7h in $CDCl_3$

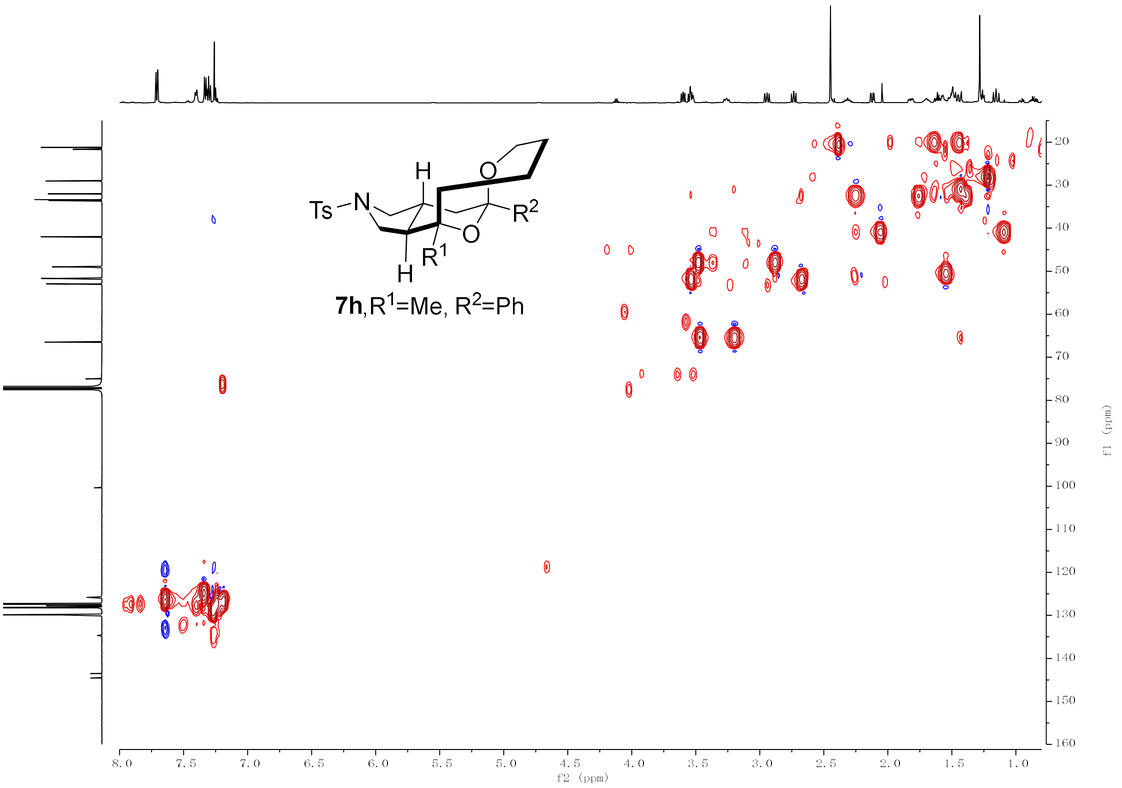

Supplementary Figure 313. ^{1}H-^{13}C HMBC for 7h in $CDCl_3$

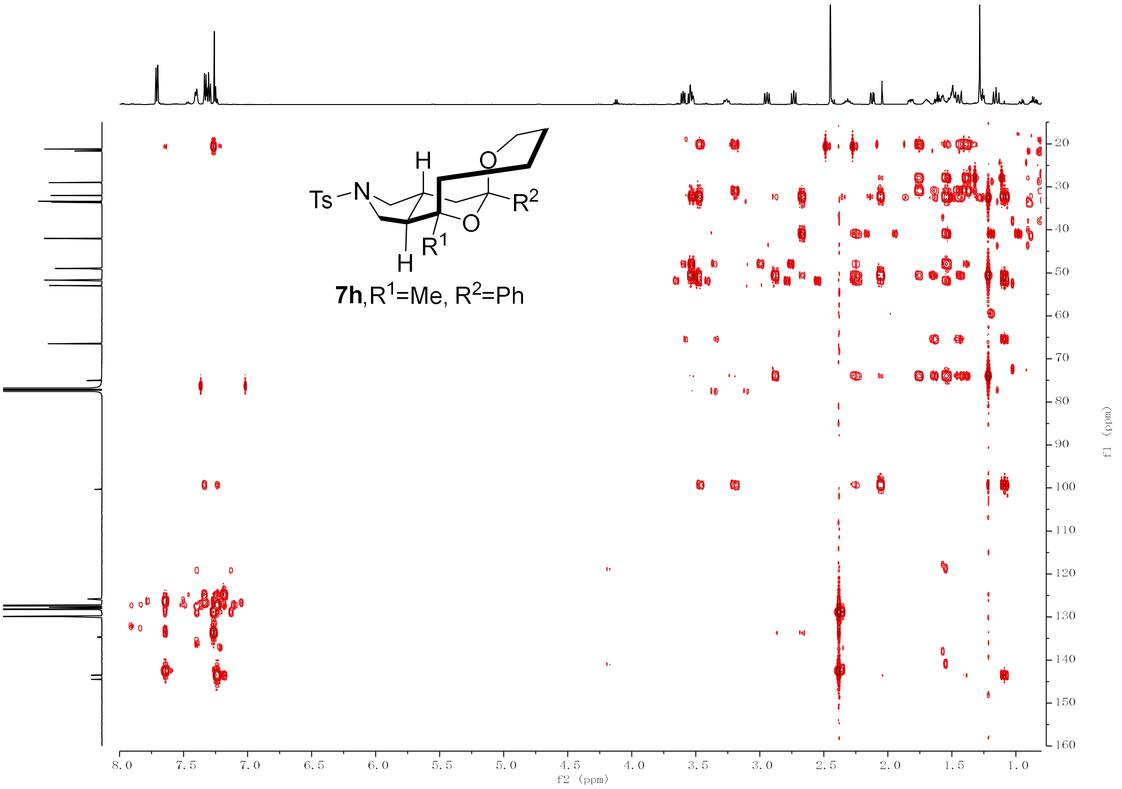

Supplementary Figure 314. ^{1}H-^{1}H COSY for 7t in CDCl$_3$

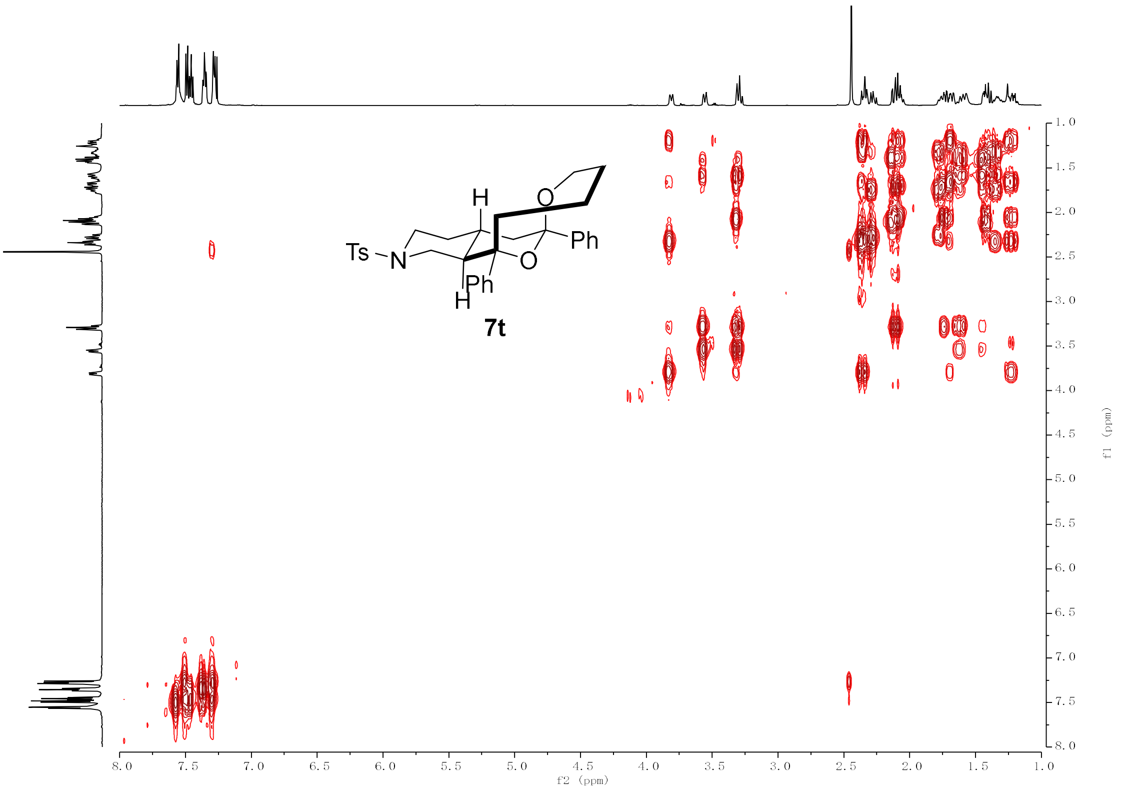

Supplementary Figure 315. ^{1}H-^{1}H ROESY for 7t in CDCl$_3$

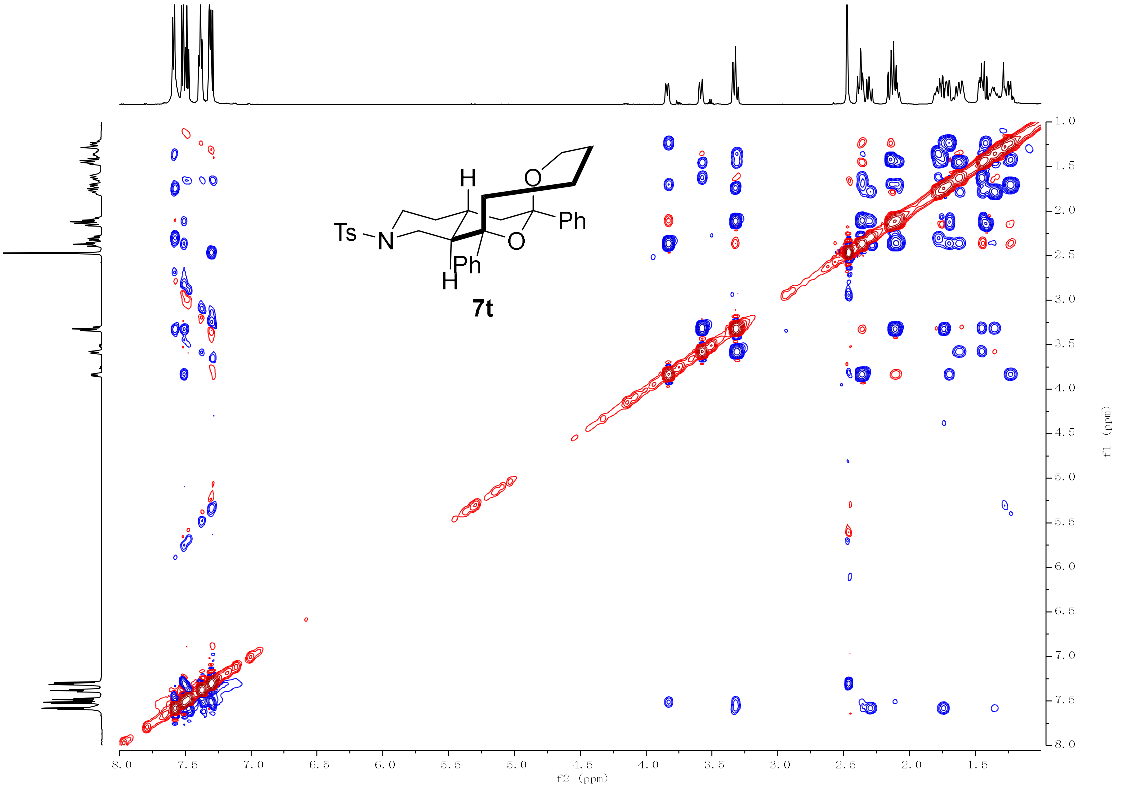

Supplementary Figure 316. ^{1}H-^{13}C HSQC for 7t in CDCl$_3$

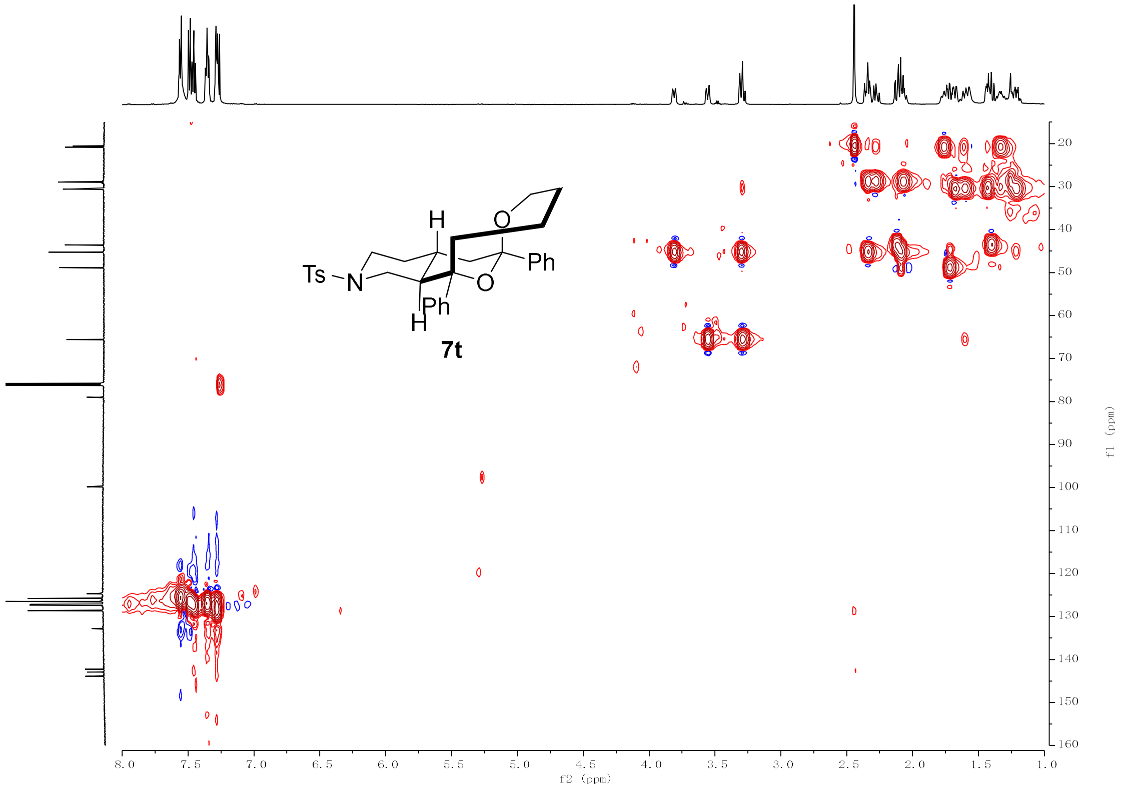

Supplementary Figure 317. ^{1}H-^{13}C HMBC for 7t in CDCl$_3$

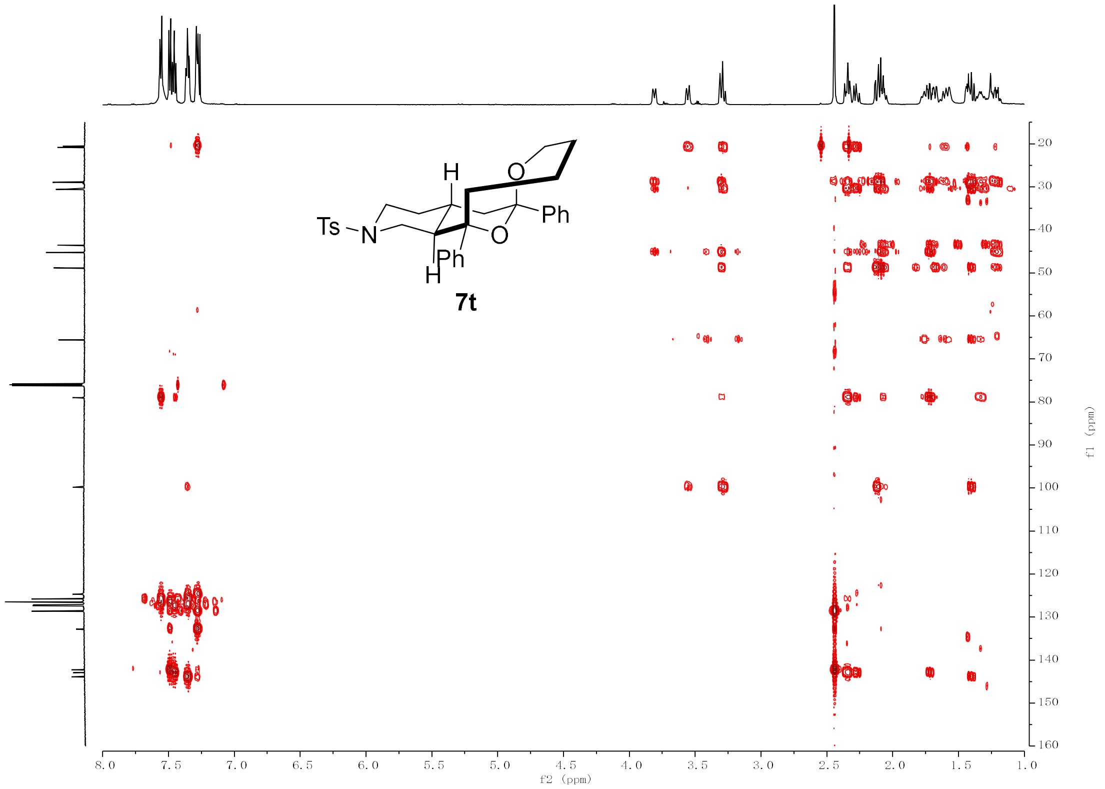

Supplementary Figure 318. ^{1}H-^{1}H COSY for 9a in acetone-d$_6$.

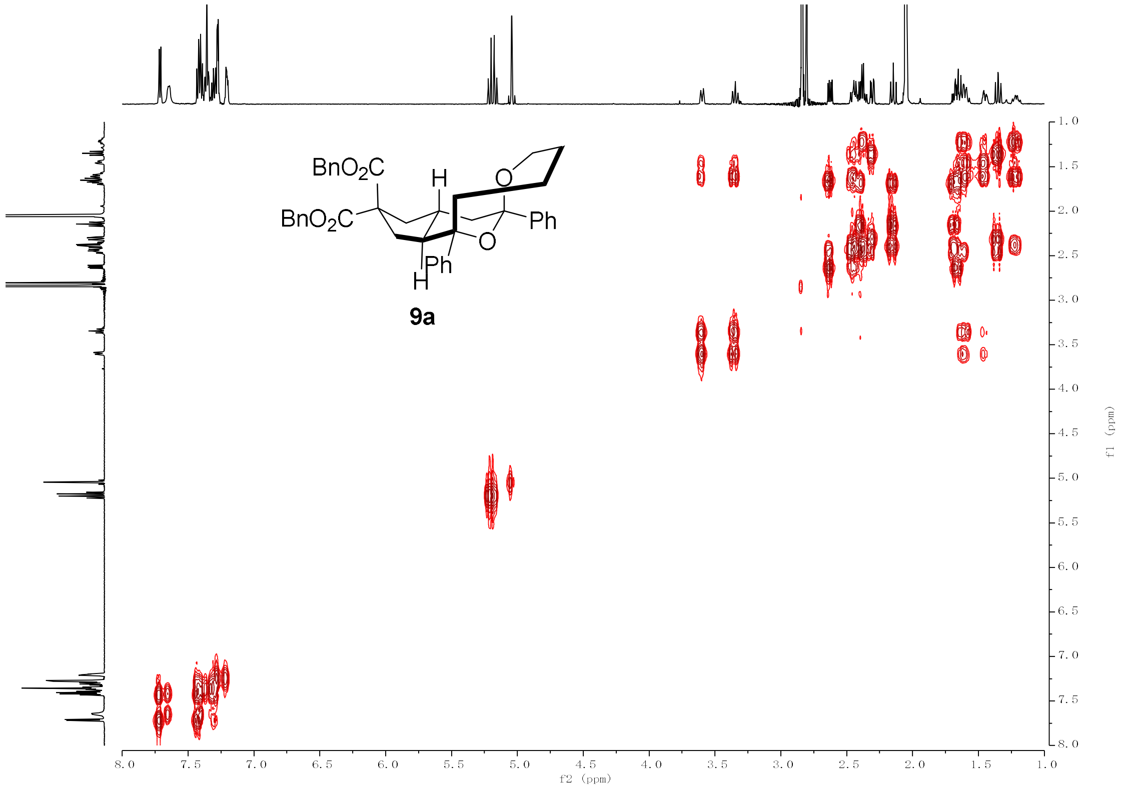

Supplementary Figure 319. ^{1}H-^{1}H ROESY for 9a in acetone-d$_6$.

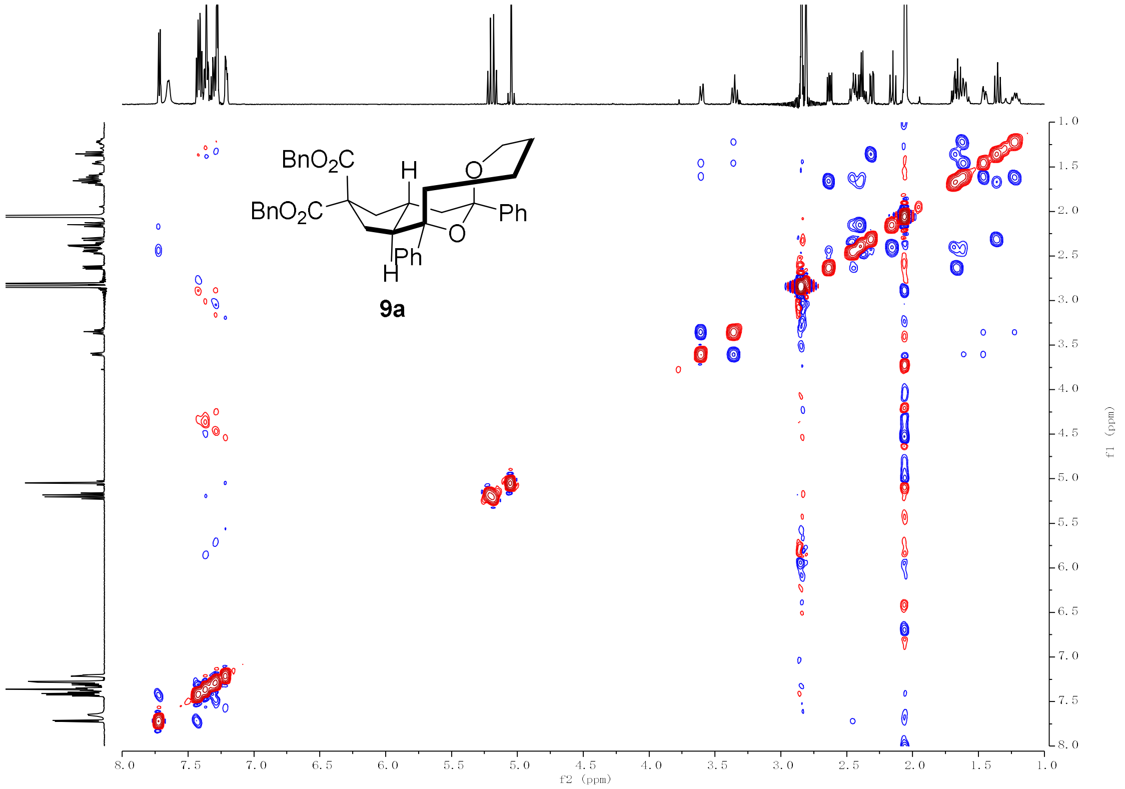

Supplementary Figure 320. ^{1}H-^{13}C HSQC for 9a in acetone-d_6.

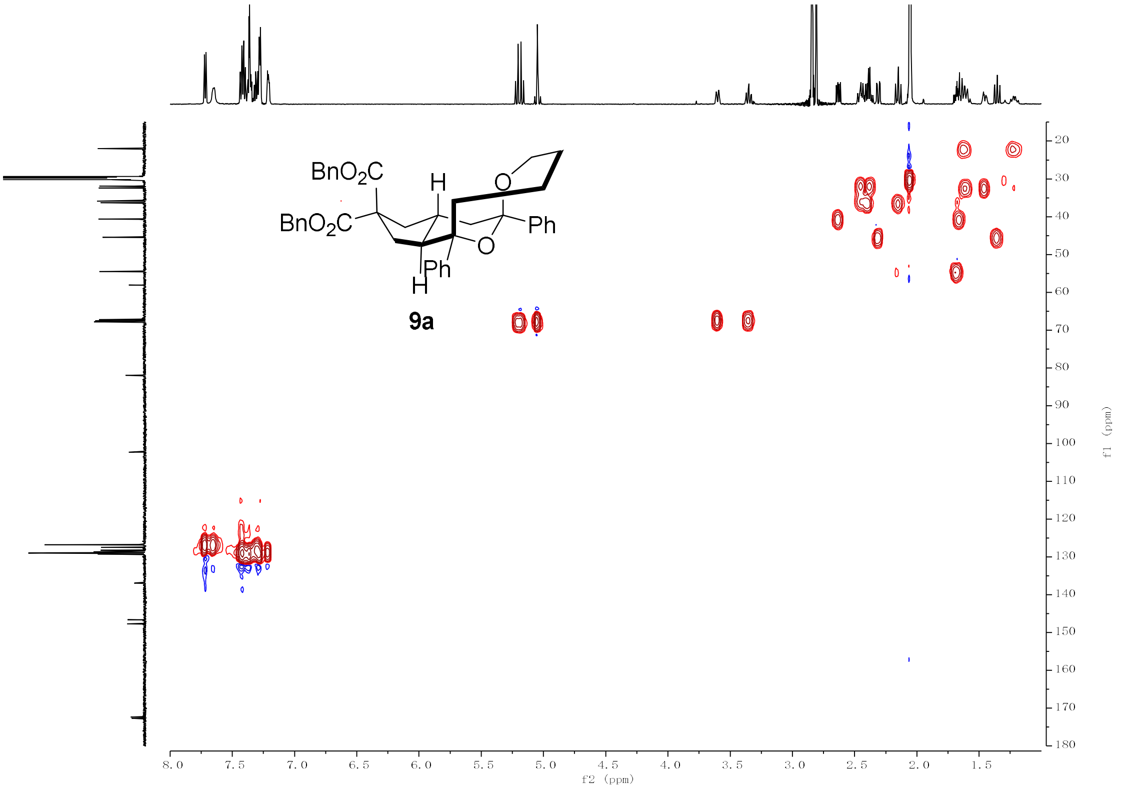

Supplementary Figure 321. ^{1}H-^{13}C HMBC for 9a in acetone-d_6.

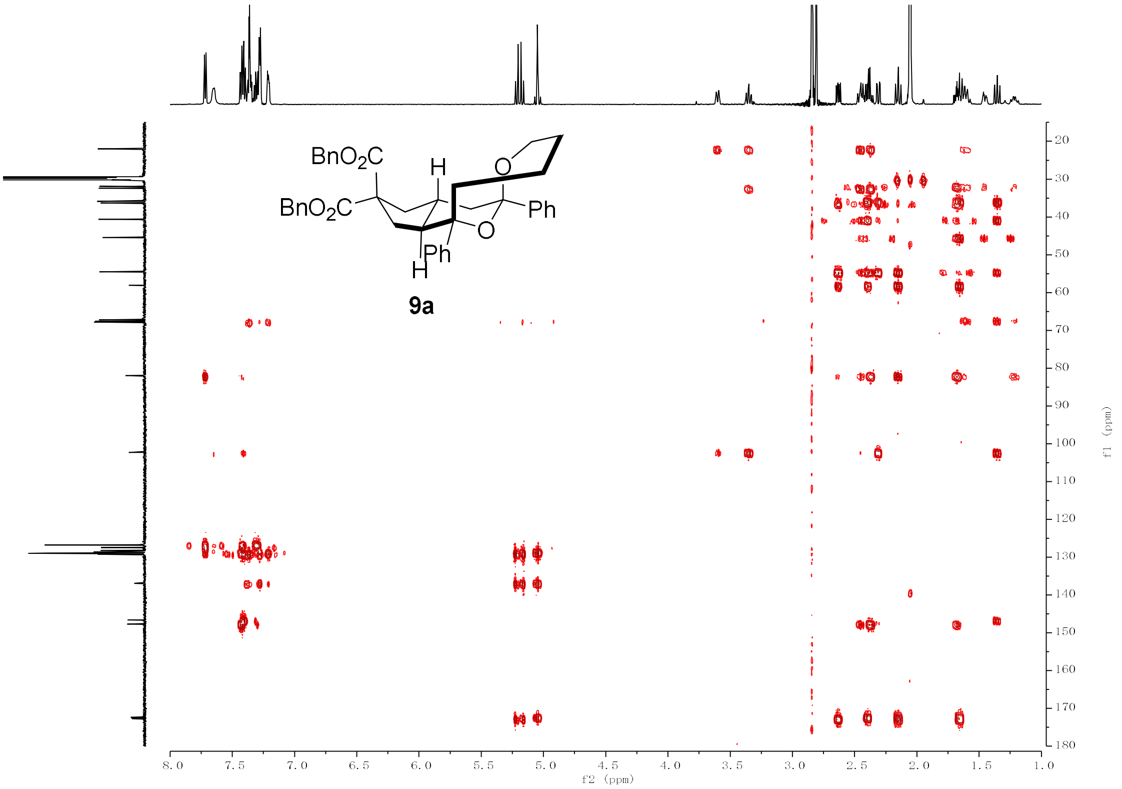

Supplementary Figure 322. ^1H-^1H COSY for 9k in $CDCl_3$.

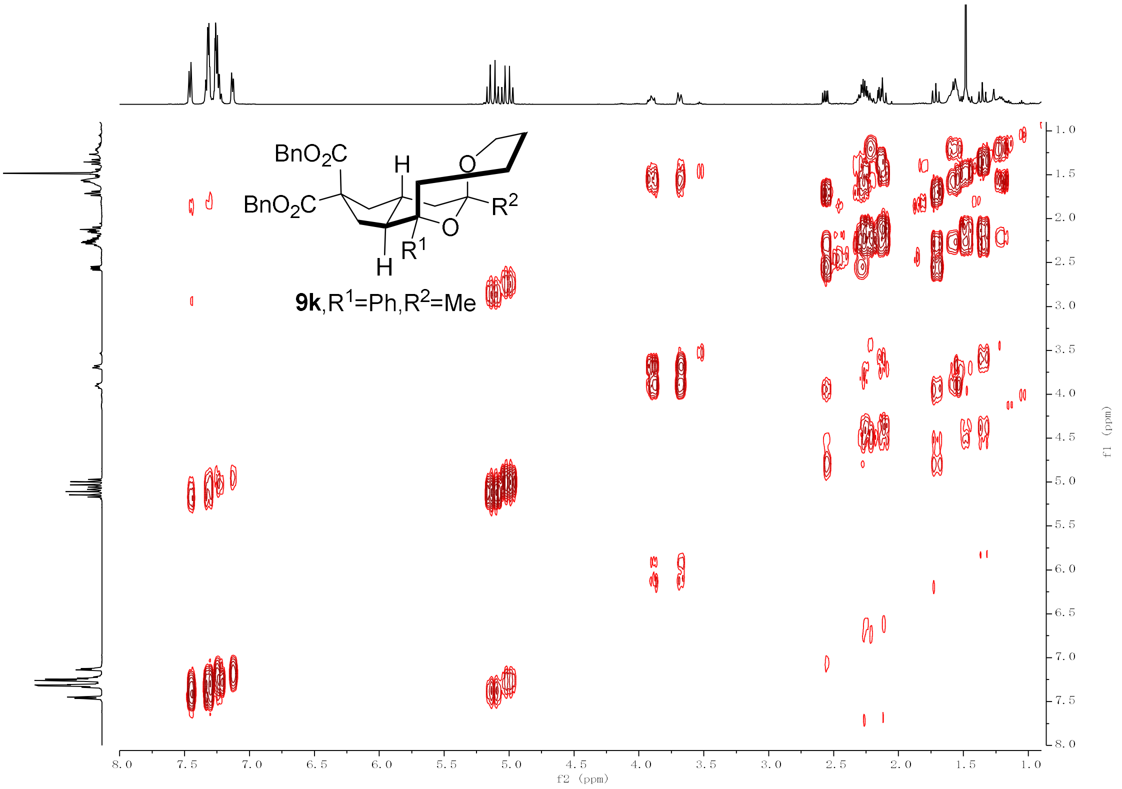

Supplementary Figure 323. ^1H-^1H ROESY for 9k in $CDCl_3$.

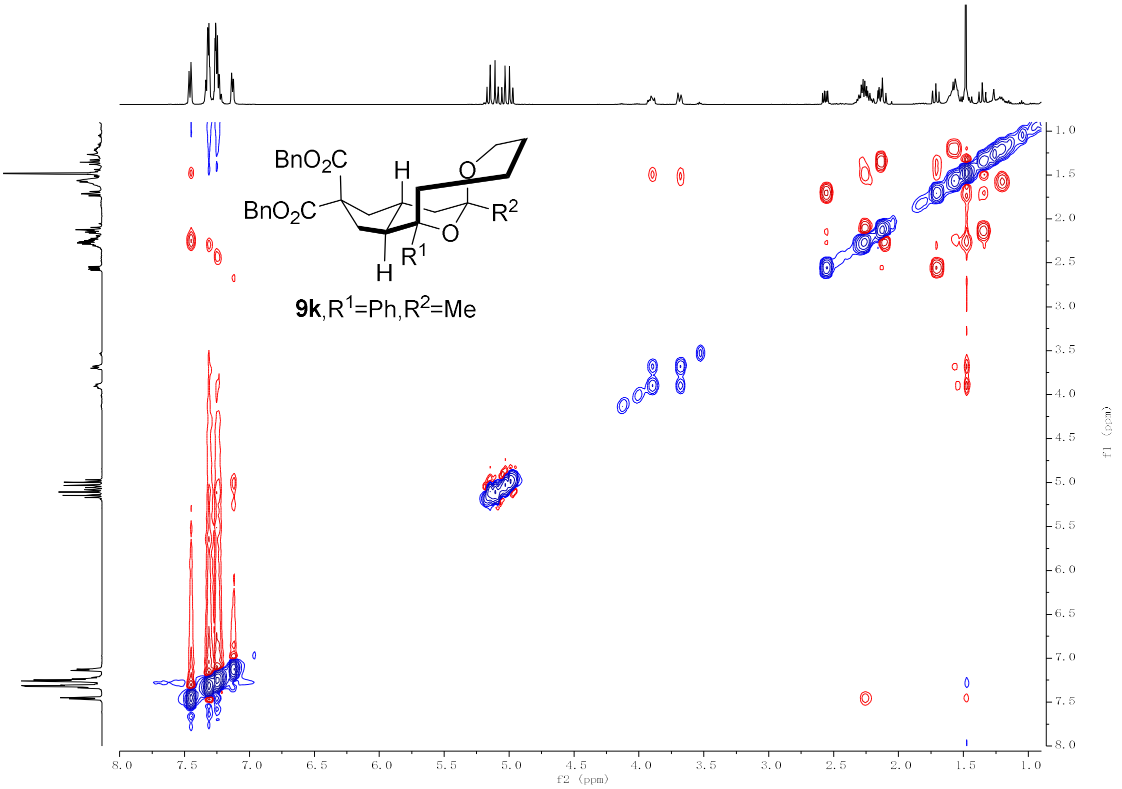

Supplementary Figure 324. ^{1}H-^{13}C **HSQC for 9k in** $CDCl_3$**.**

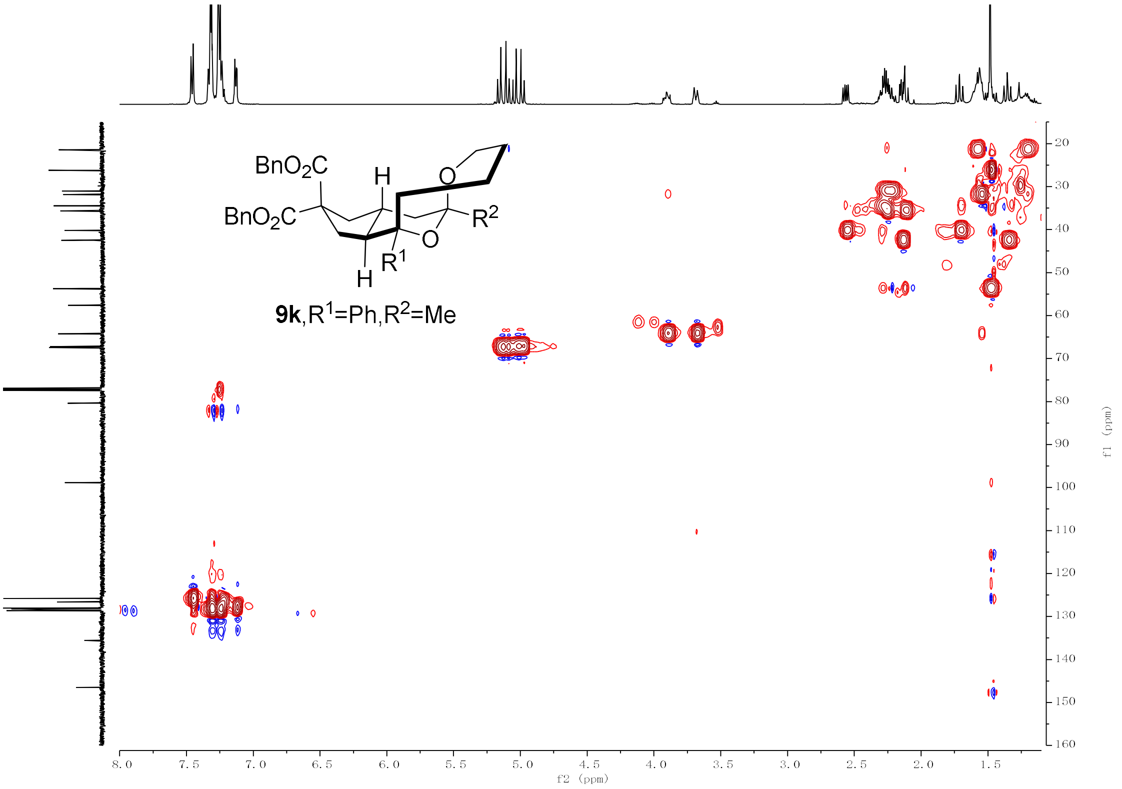

Supplementary Figure 325. ^{1}H-^{13}C **HMBC for 9k in** $CDCl_3$**.**

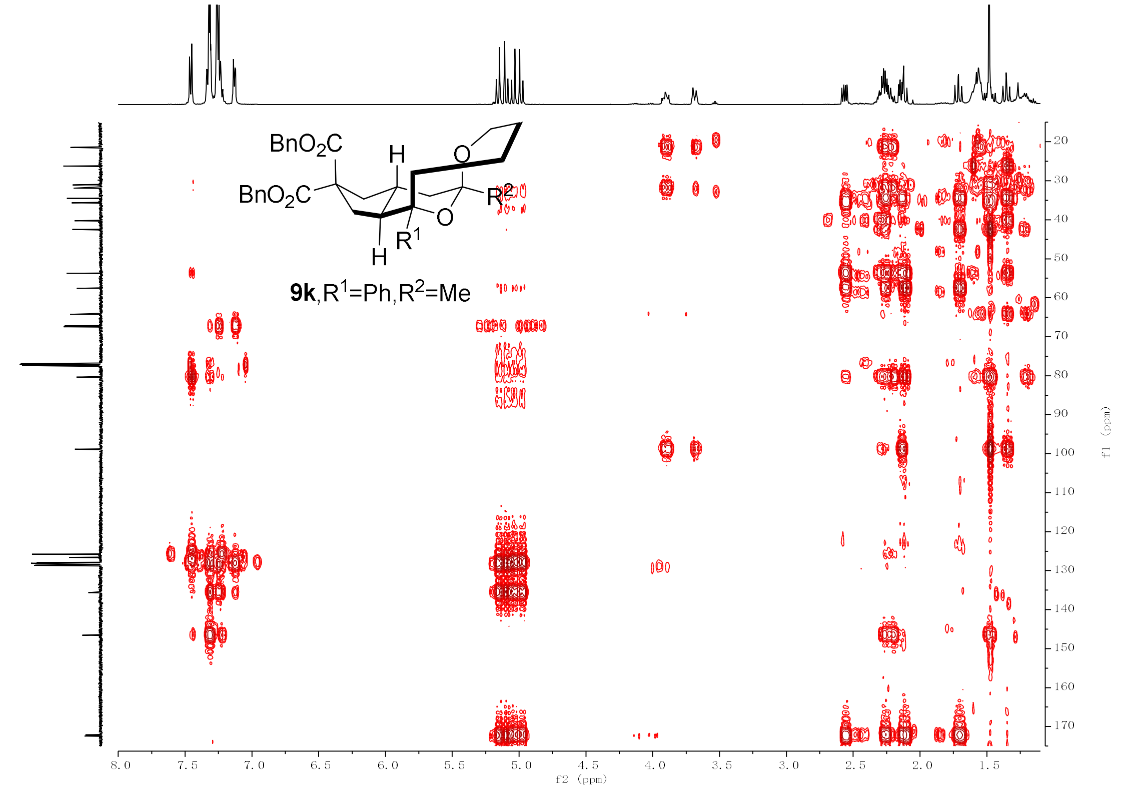

Supplementary Figure 326. ^1H-^1H COSY for 11a in $CDCl_3$.

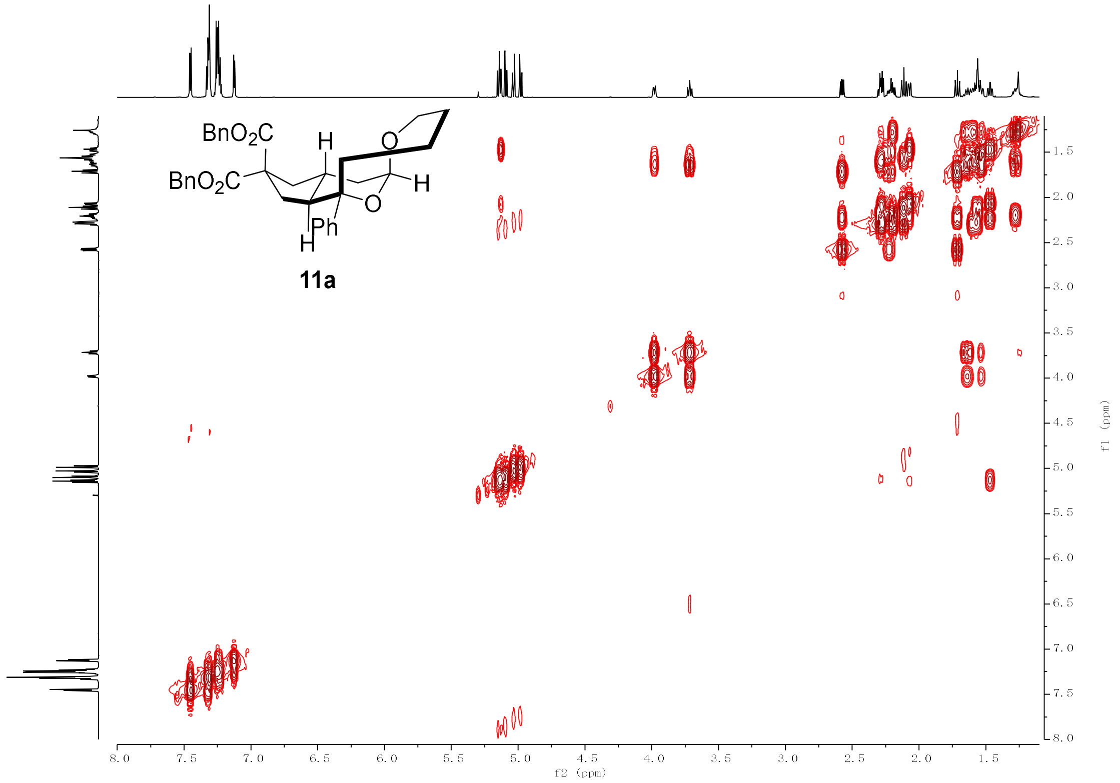

Supplementary Figure 327. ^1H-^1H ROESY for 11a in $CDCl_3$.

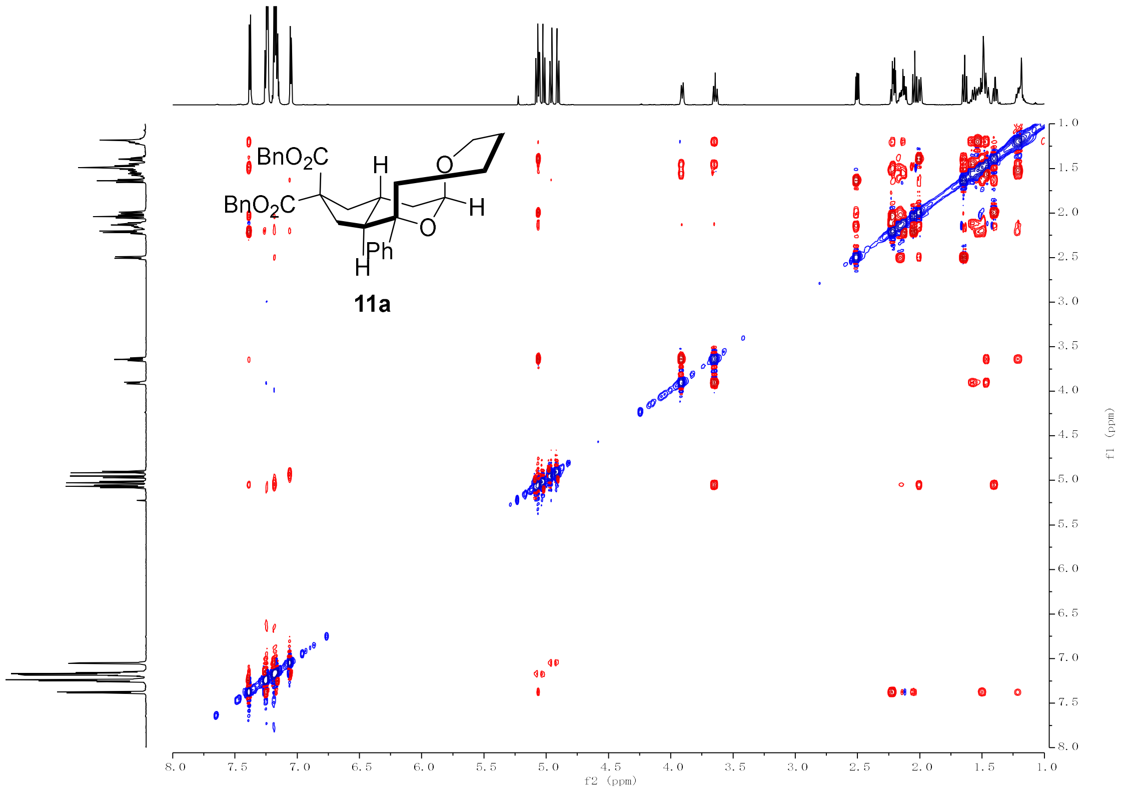

Supplementary Figure 328. ^{1}H-^{13}C HSQC for 11a in CDCl3.

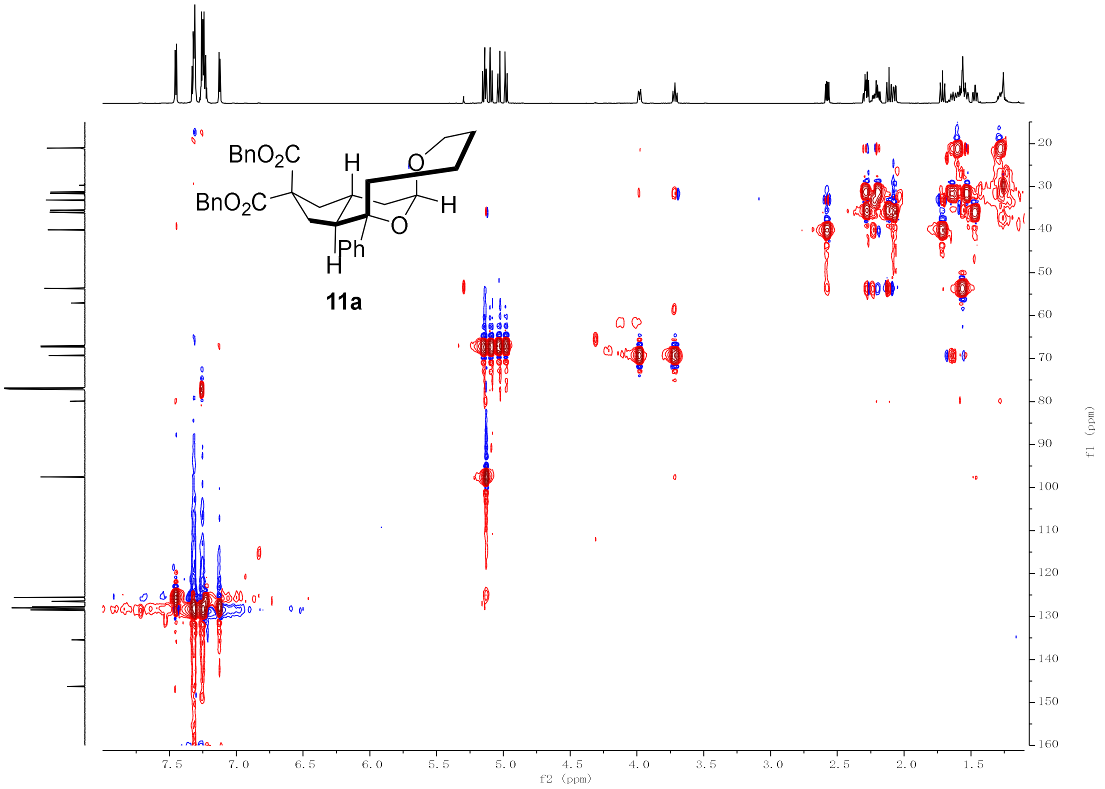

Supplementary Figure 329. ^{1}H-^{13}C HMBC for 11a in CDCl3.

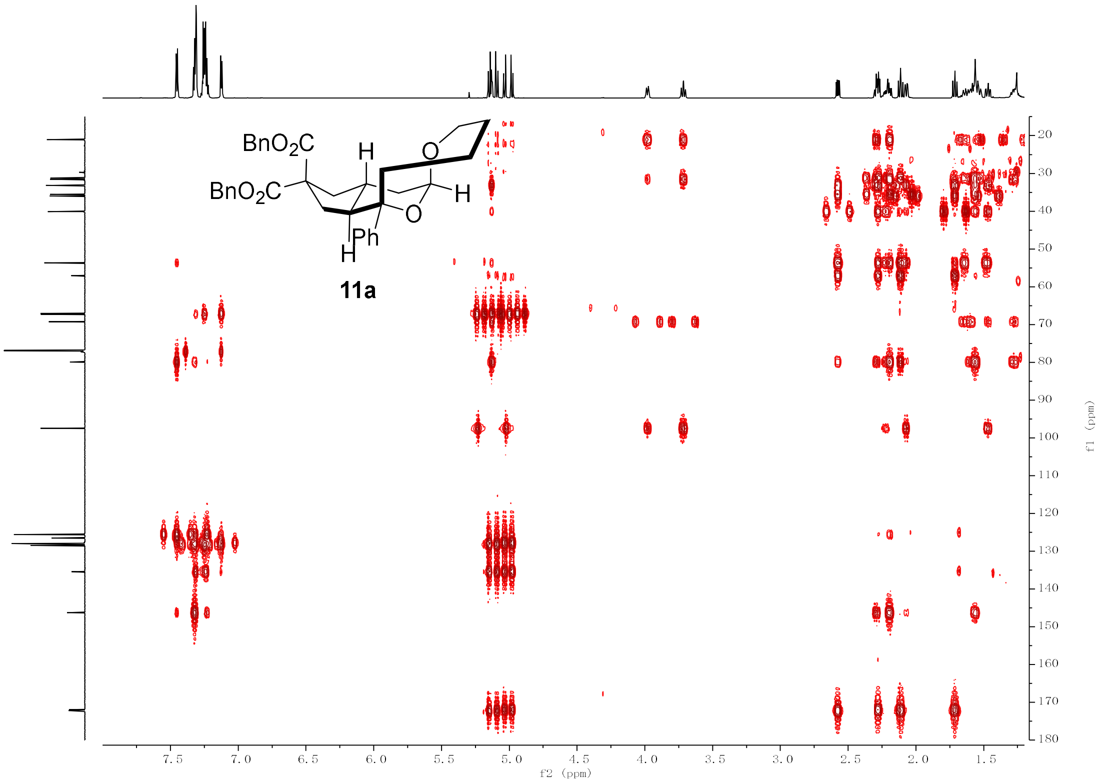

Supplementary Figure 330. ^{1}H-^{1}H COSY for 12 in $CDCl_3$.

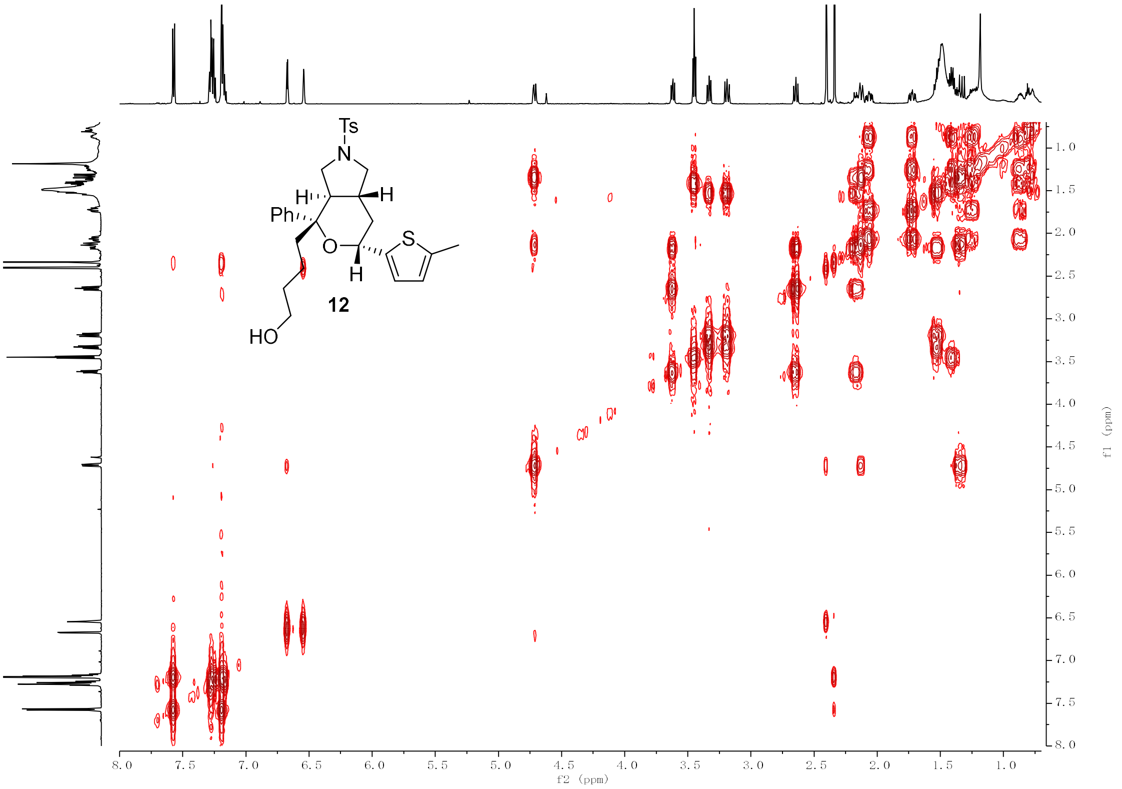

Supplementary Figure 331. ^{1}H-^{1}H ROESY for 12 in $CDCl_3$.

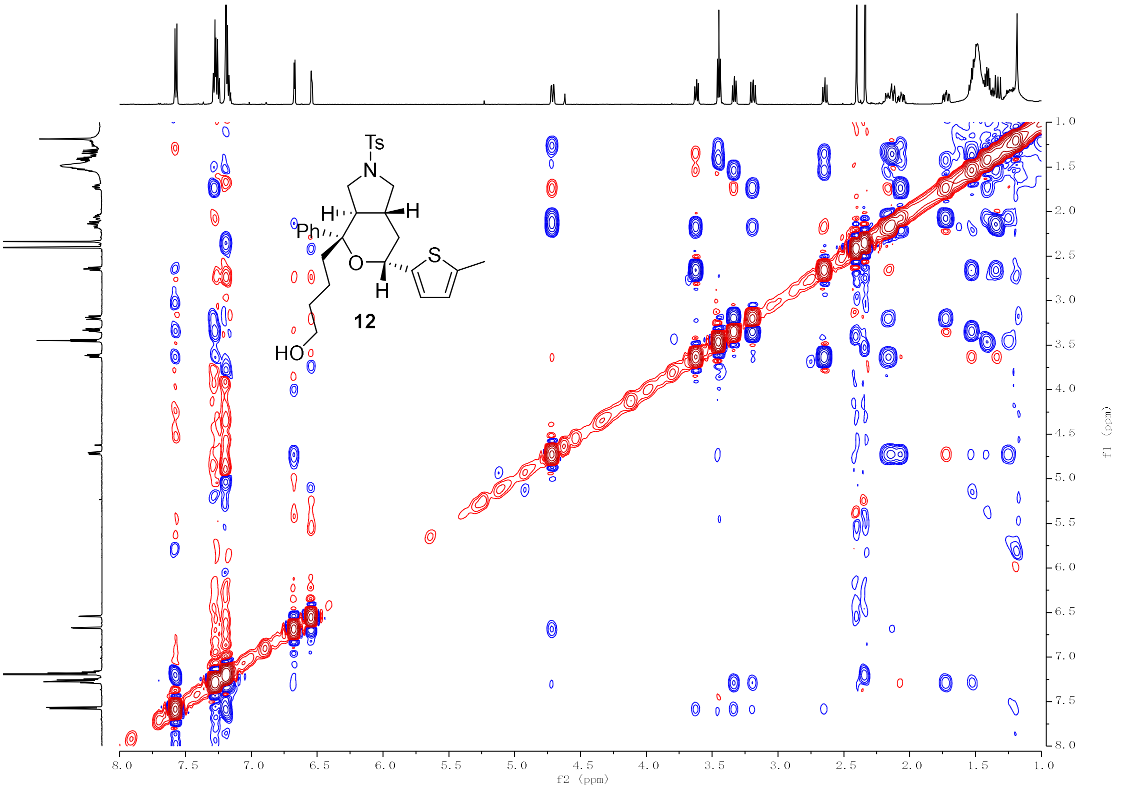

Supplementary Figure 332. ^{1}H-^{13}C HSQC for 12 in CDCl$_3$.

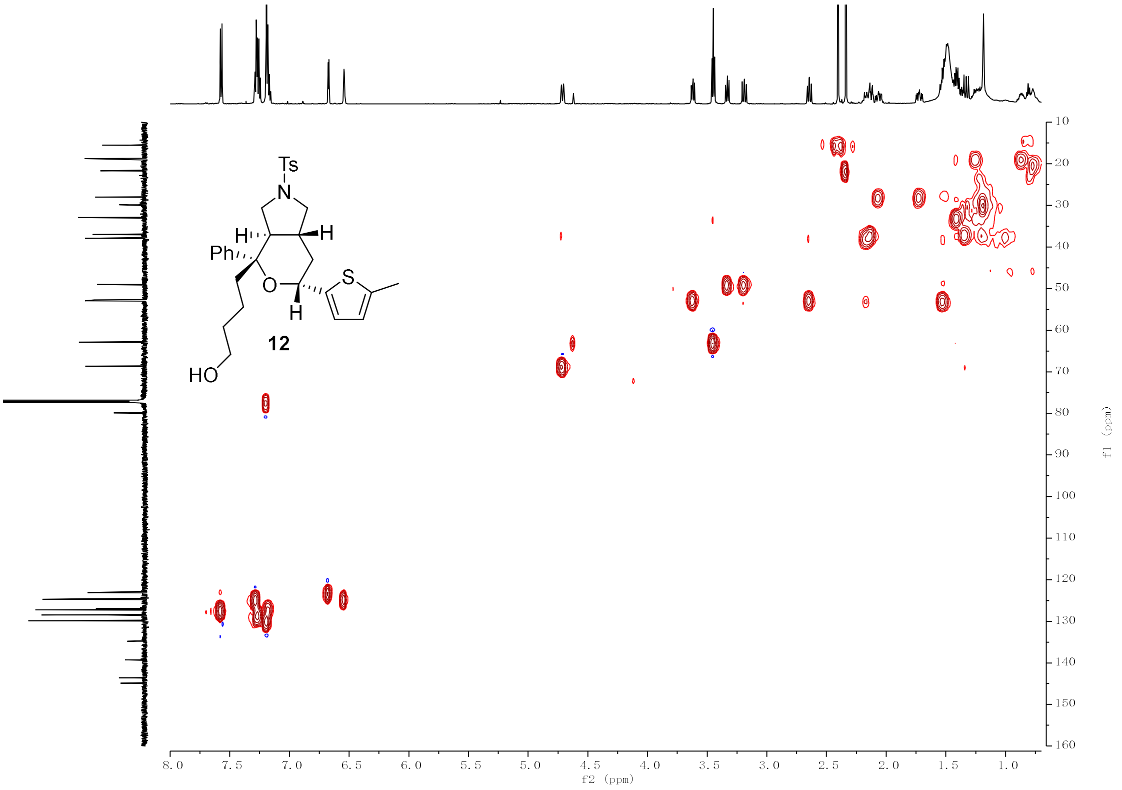

Supplementary Figure 333. ^{1}H-^{13}C HMBC for 12 in CDCl$_3$.

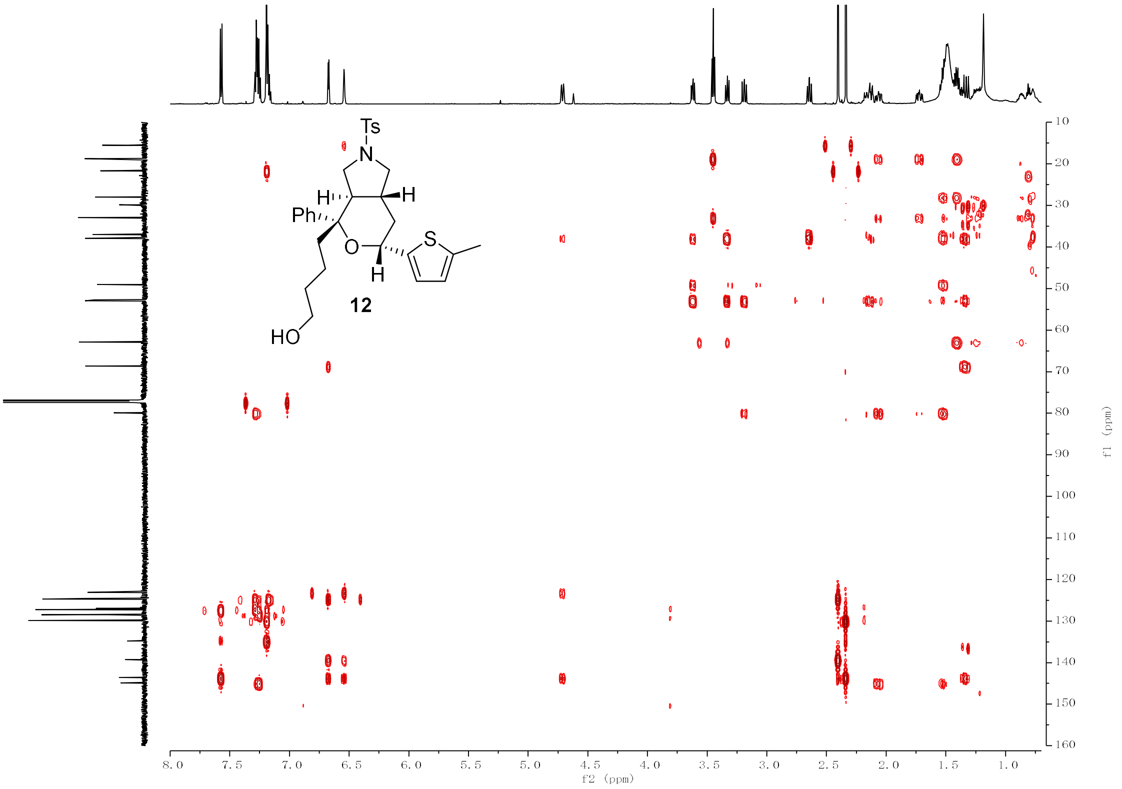

Supplementary Figure 334. ^{1}H-^{1}H COSY for 13 in CDCl$_3$.

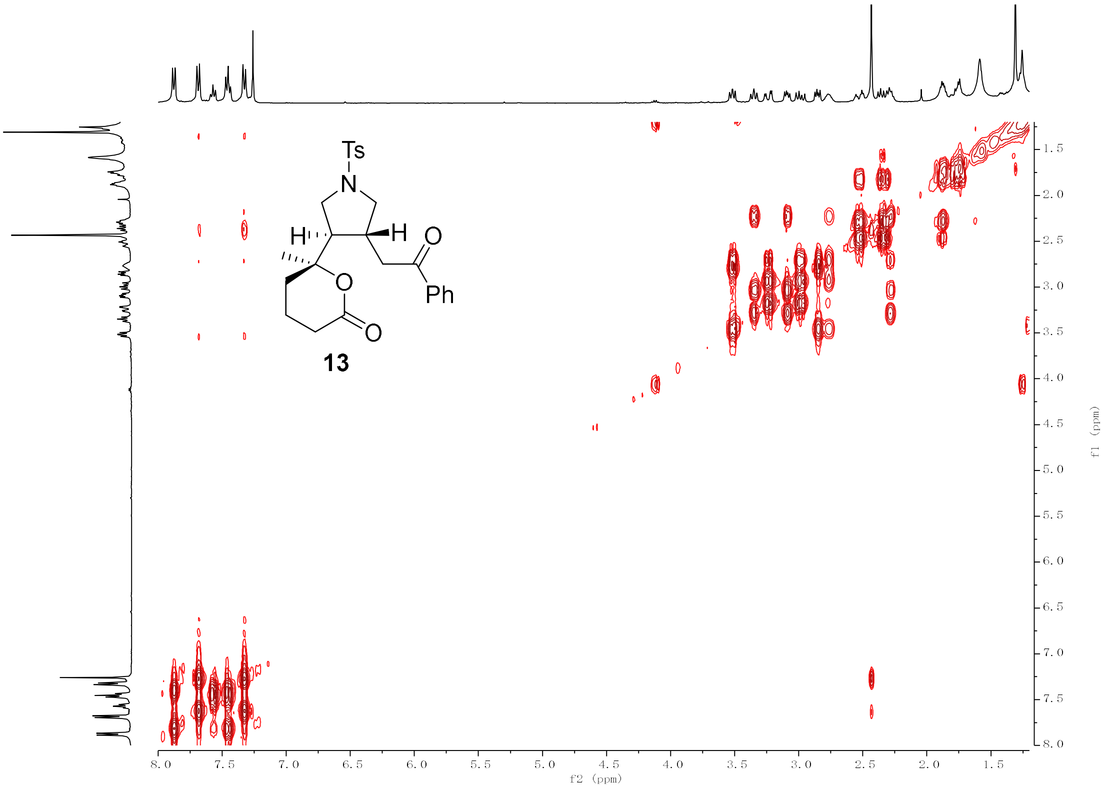

Supplementary Figure 335. ^{1}H-^{1}H ROESY for 13 in CDCl$_3$.

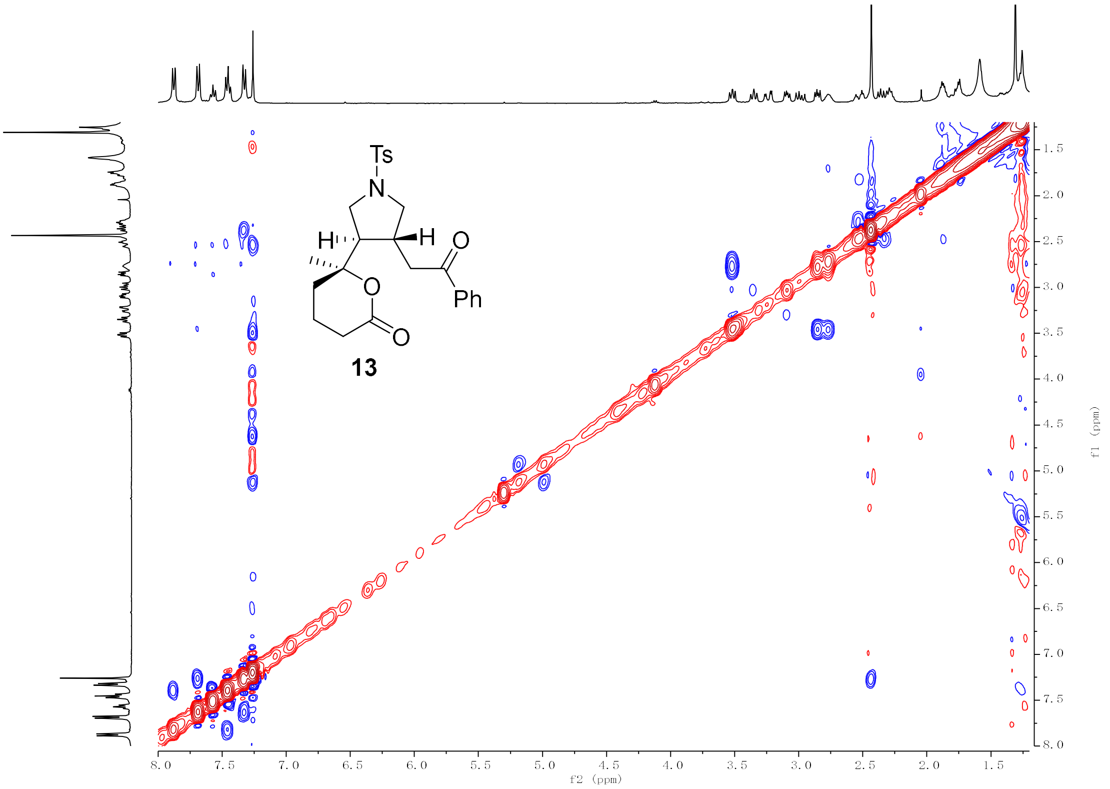

Supplementary Figure 336. ^{1}H-^{13}C HSQC for 13 in $CDCl_3$.

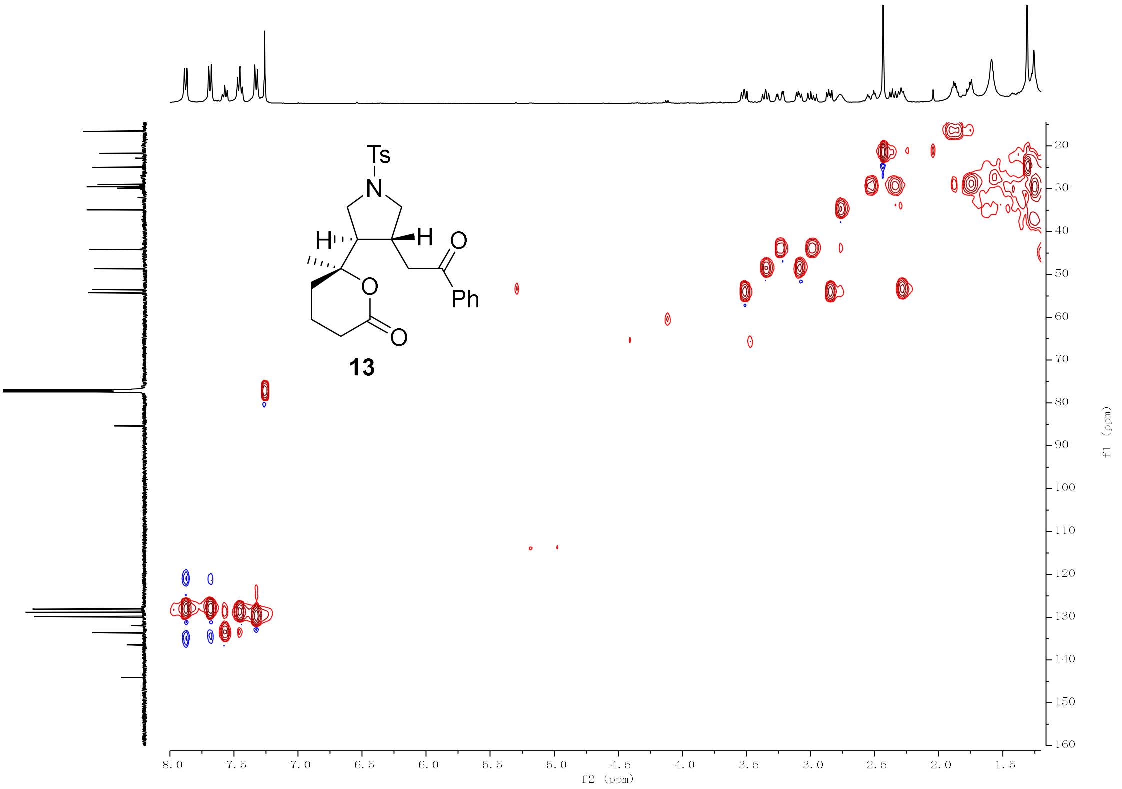

Supplementary Figure 337. ^{1}H-^{13}C HMBC for 13 in $CDCl_3$.

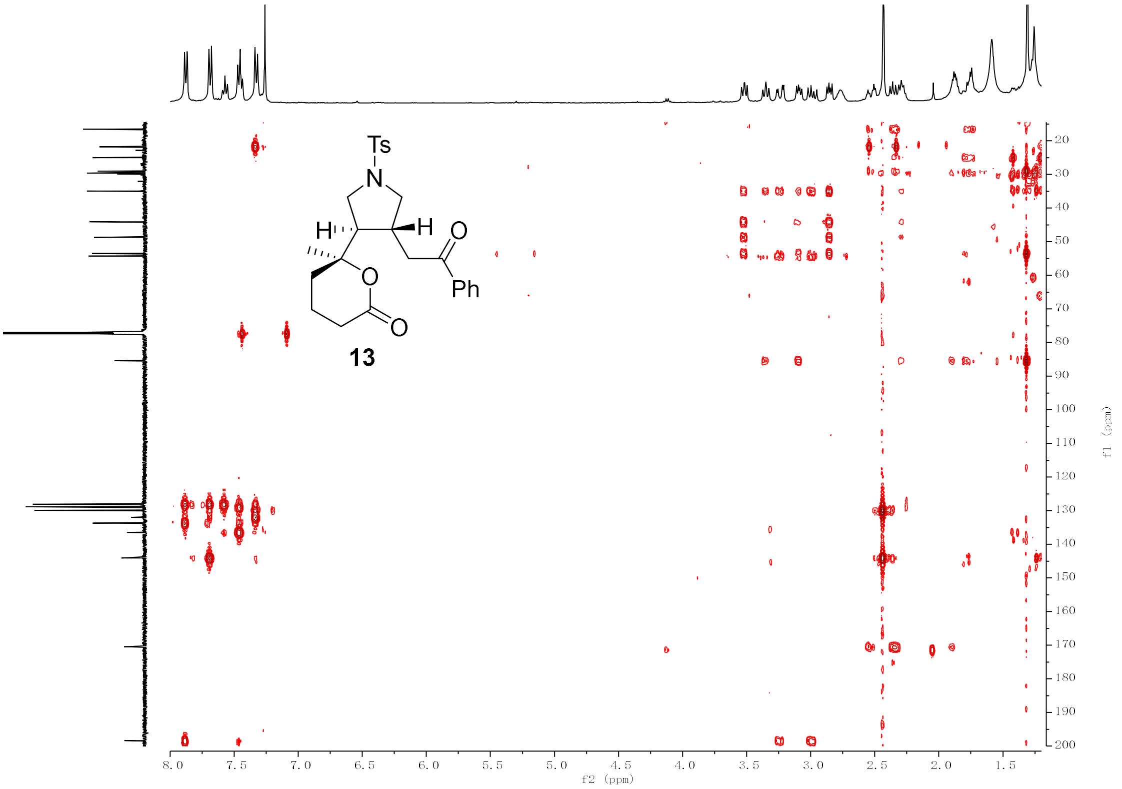

Supplementary Figure 338. ^{1}H-^{1}H COSY for 14 in acetone-d_6.

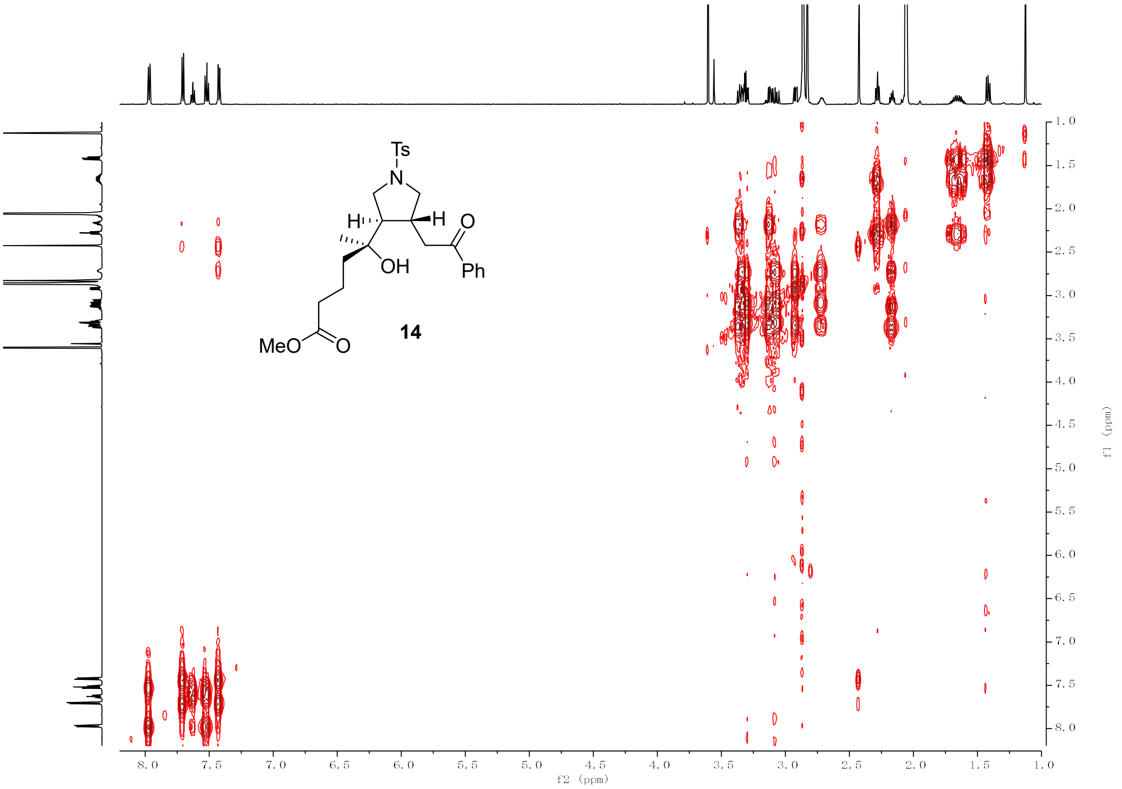

Supplementary Figure 339. ^{1}H-^{1}H ROESY for 14 in acetone-d_6.

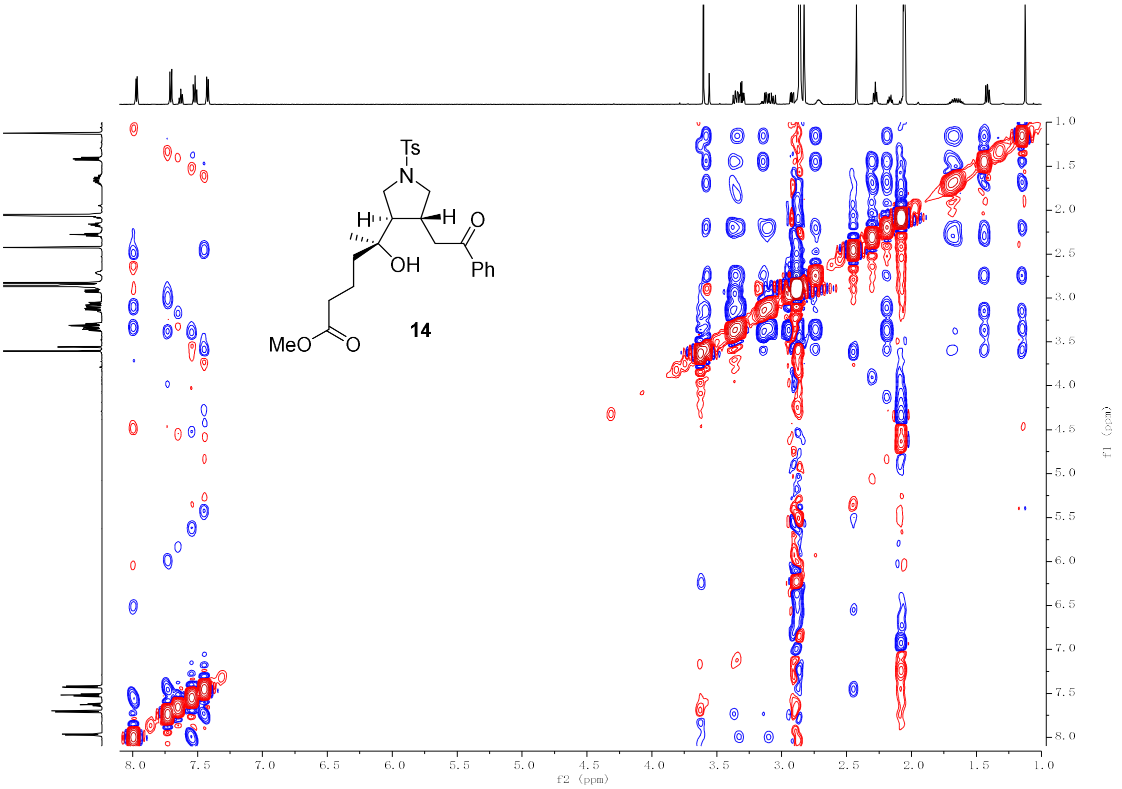

Supplementary Figure 340. ^{1}H-^{13}C HSQC for 14 in acetone-d$_6$.

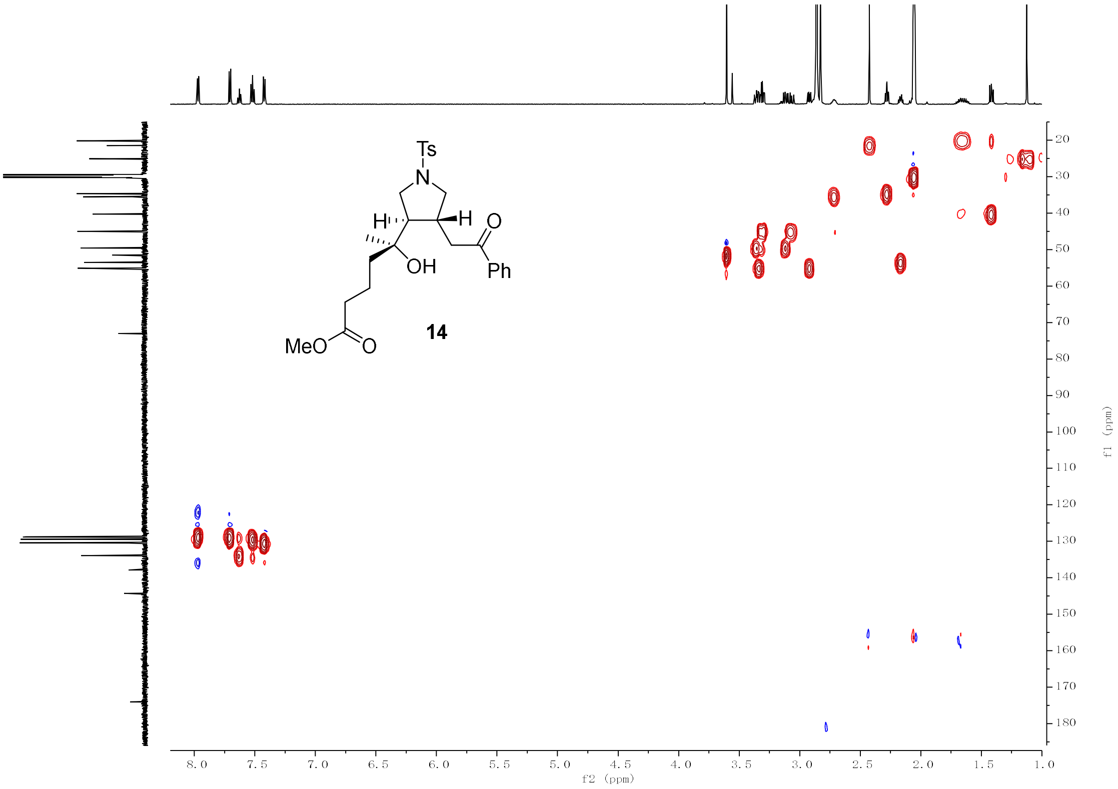

Supplementary Figure 341. ^{1}H-^{13}C HMBC for 14 in acetone-d$_6$.

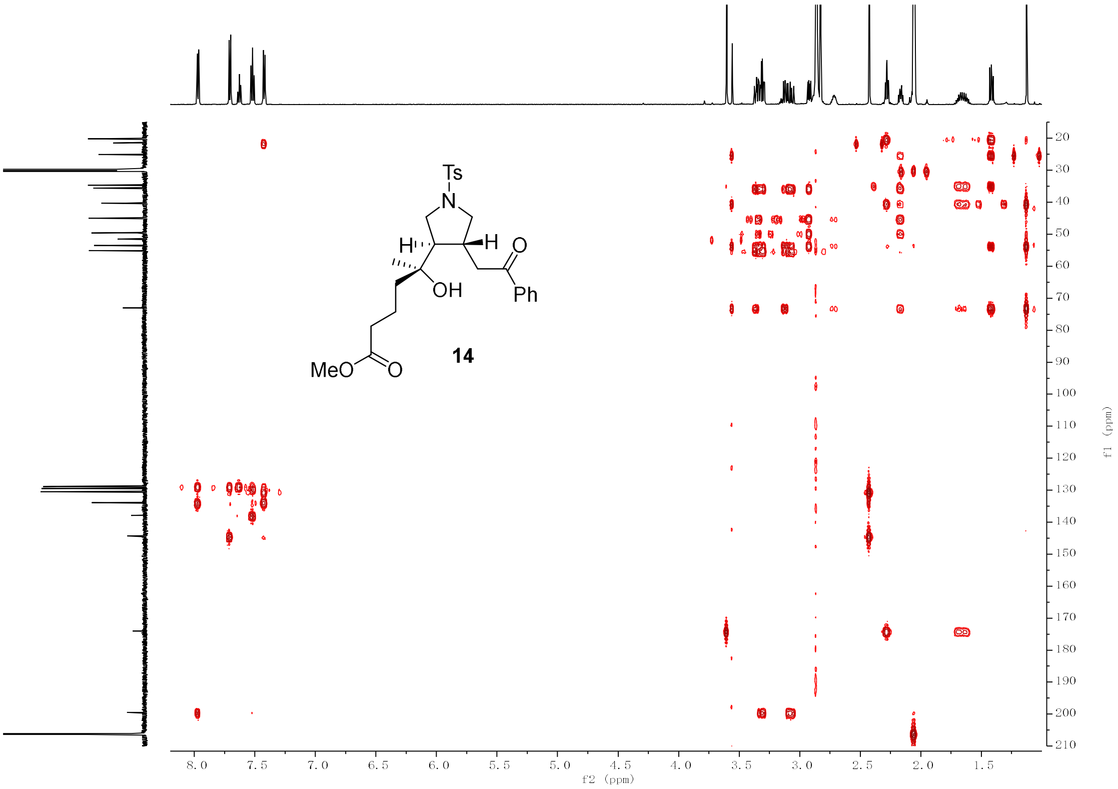

Supplementary Figure 342. ^{1}H-^{1}H COSY for 15 in acetone-d$_6$.

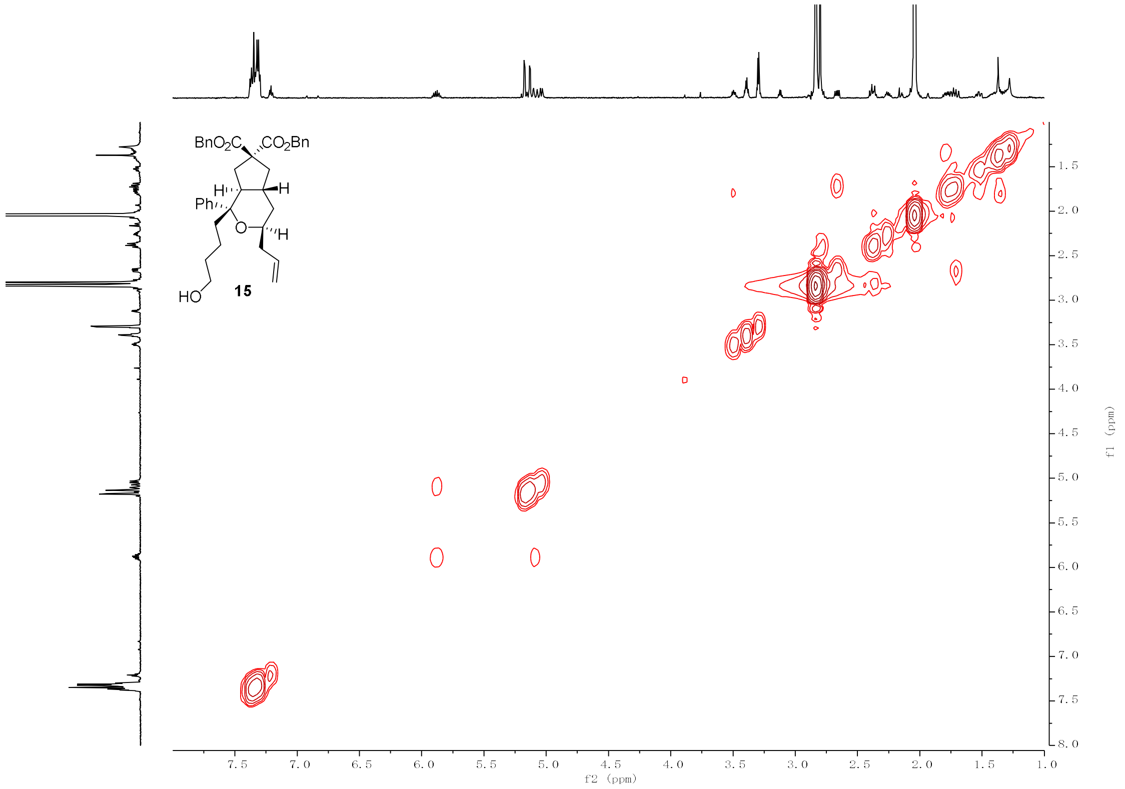

Supplementary Figure 343. ^{1}H-^{1}H ROESY for 15 in acetone-d$_6$.

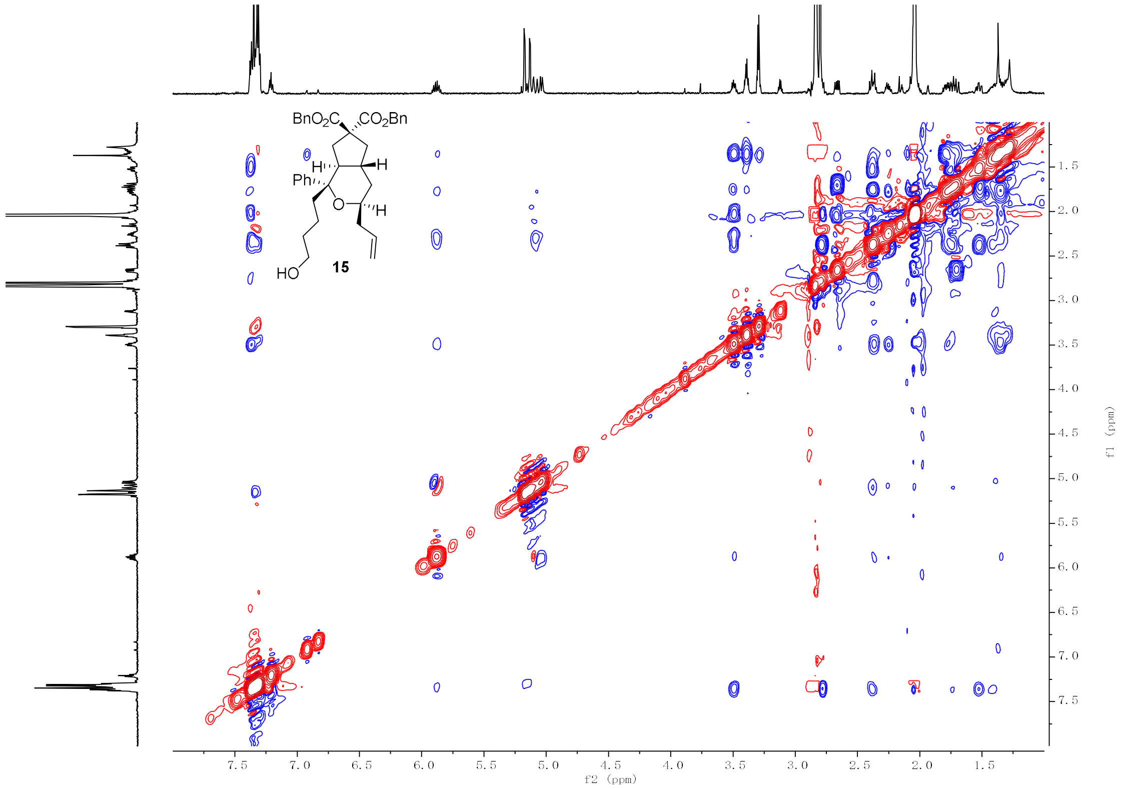

Supplementary Figure 344. ^{1}H-^{13}C HSQC for 15 in acetone-d₆.

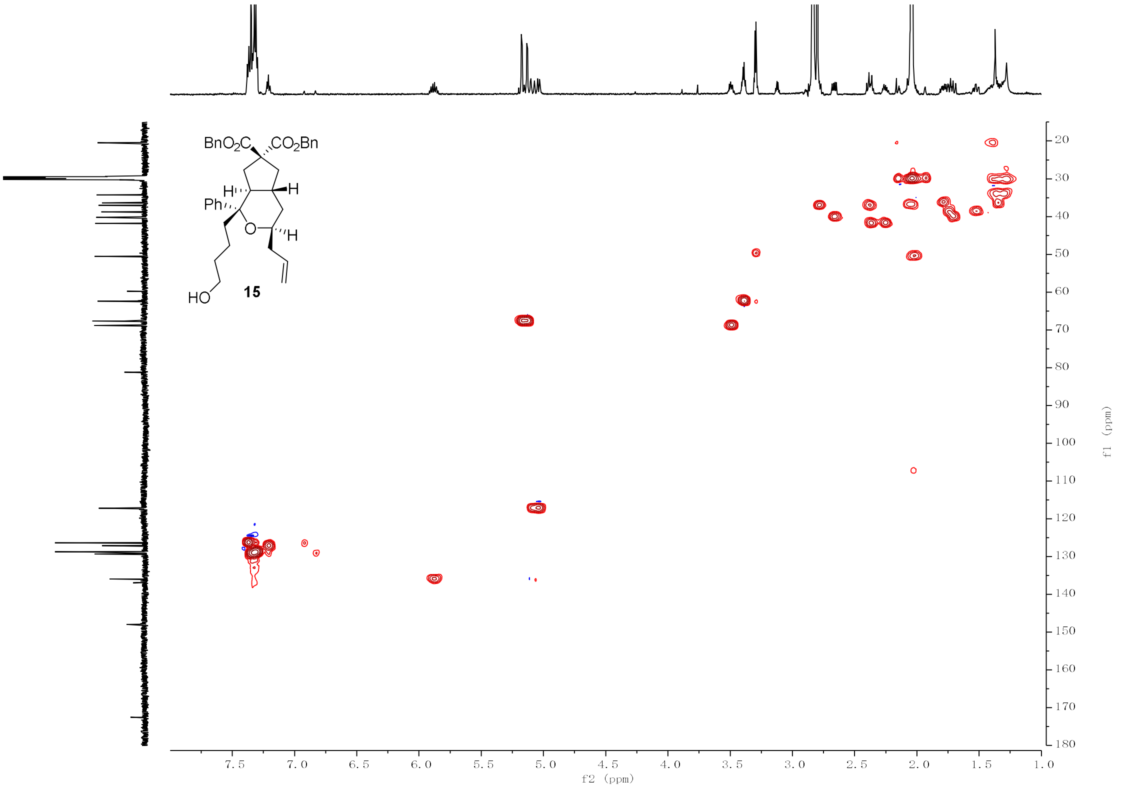

Supplementary Figure 345. ^{1}H-^{13}C HMBC for 15 in Acetone.

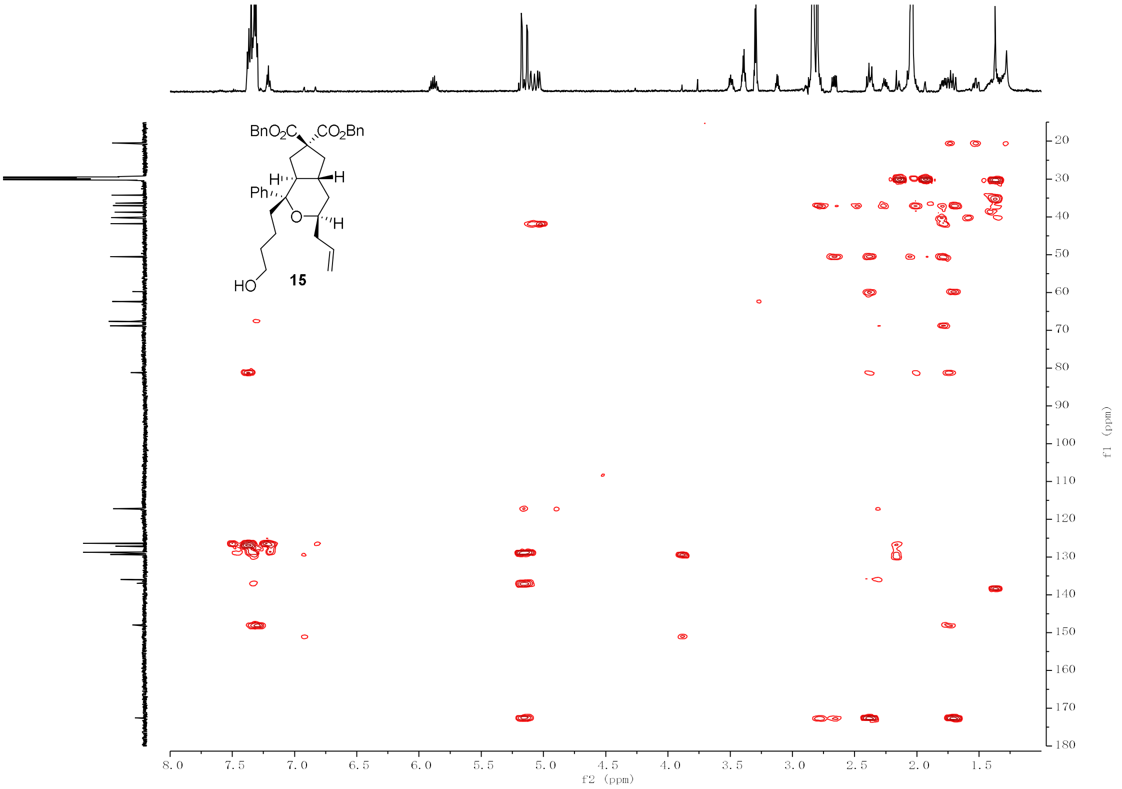

Supplementary Figure 346. ^{1}H-^{1}H COSY for 17 in acetone-d$_6$.

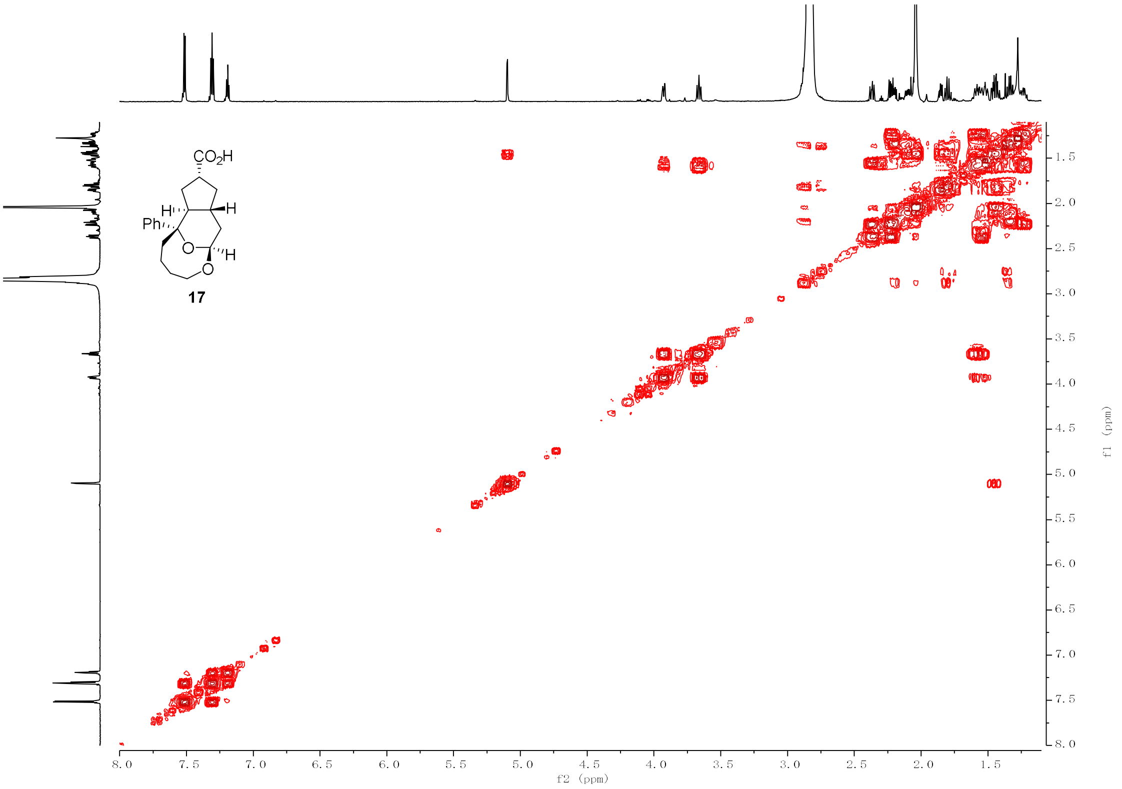

Supplementary Figure 347. ^{1}H-^{1}H ROESY for 17 in acetone-d$_6$.

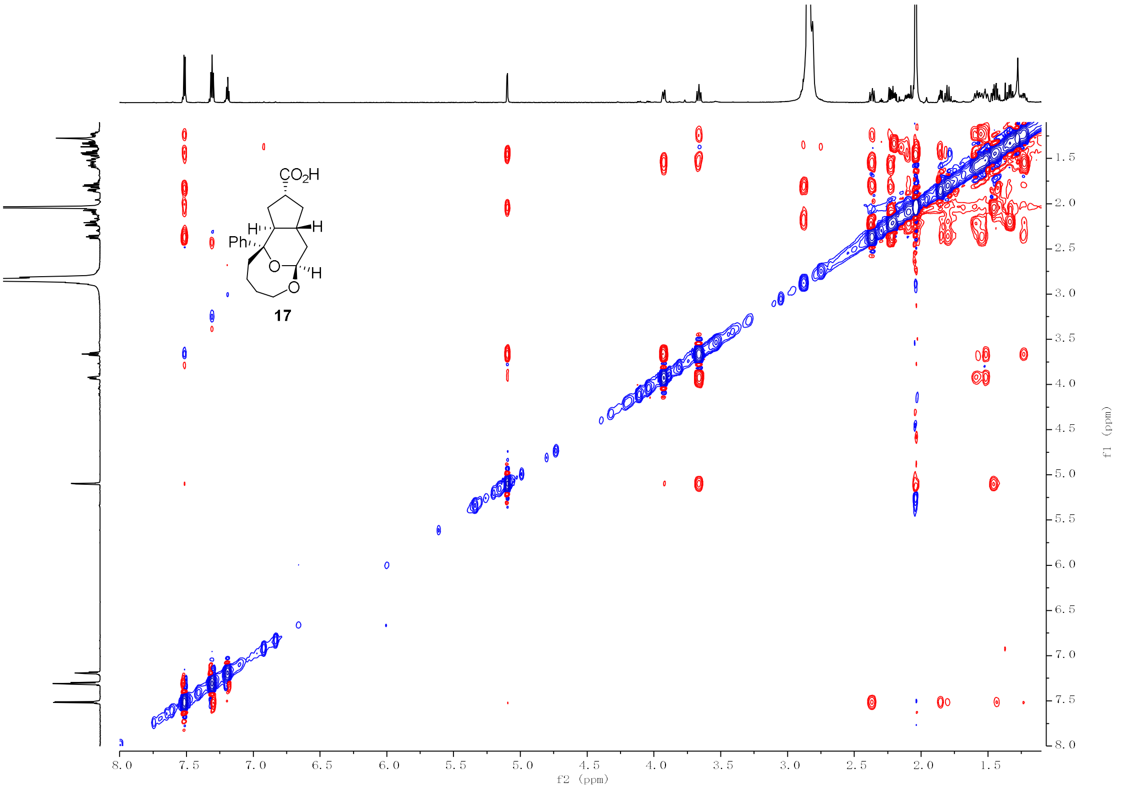

Supplementary Figure 348. ^{1}H-^{13}C HSQC for 17 in acetone-d$_6$.

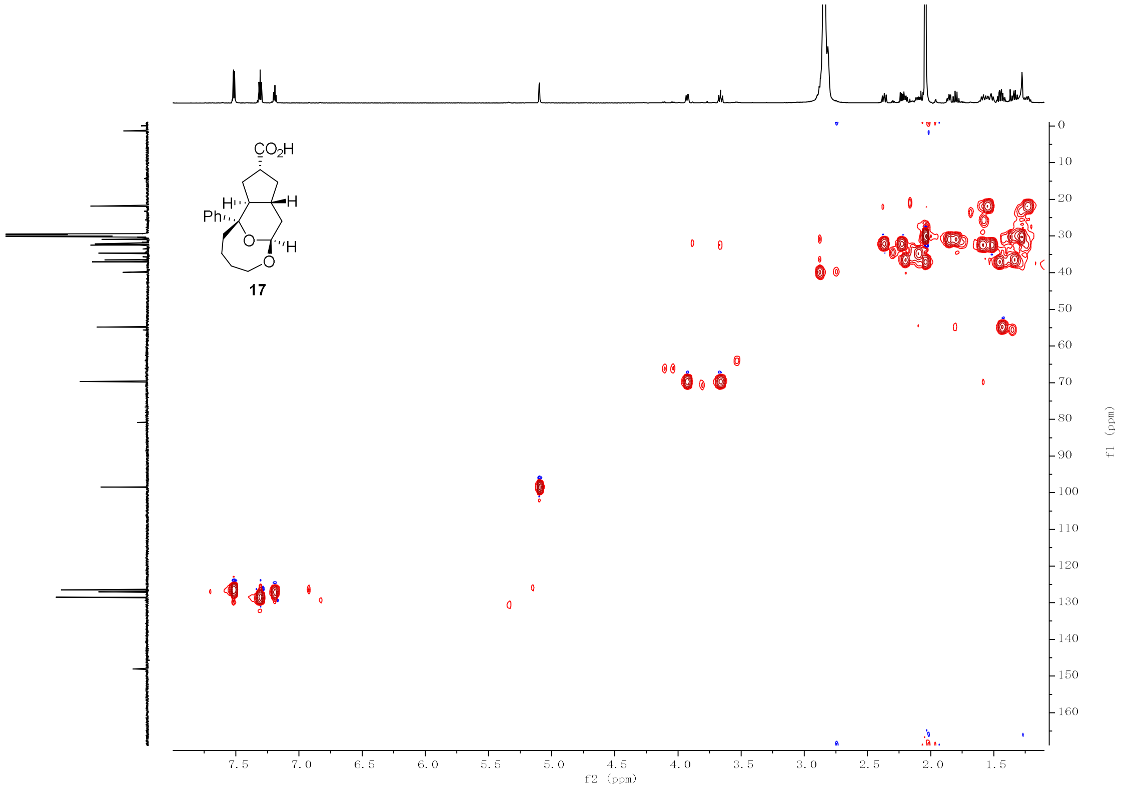

Supplementary Figure 349. ^{1}H-^{13}C HMBC for 17 in acetone-d$_6$.

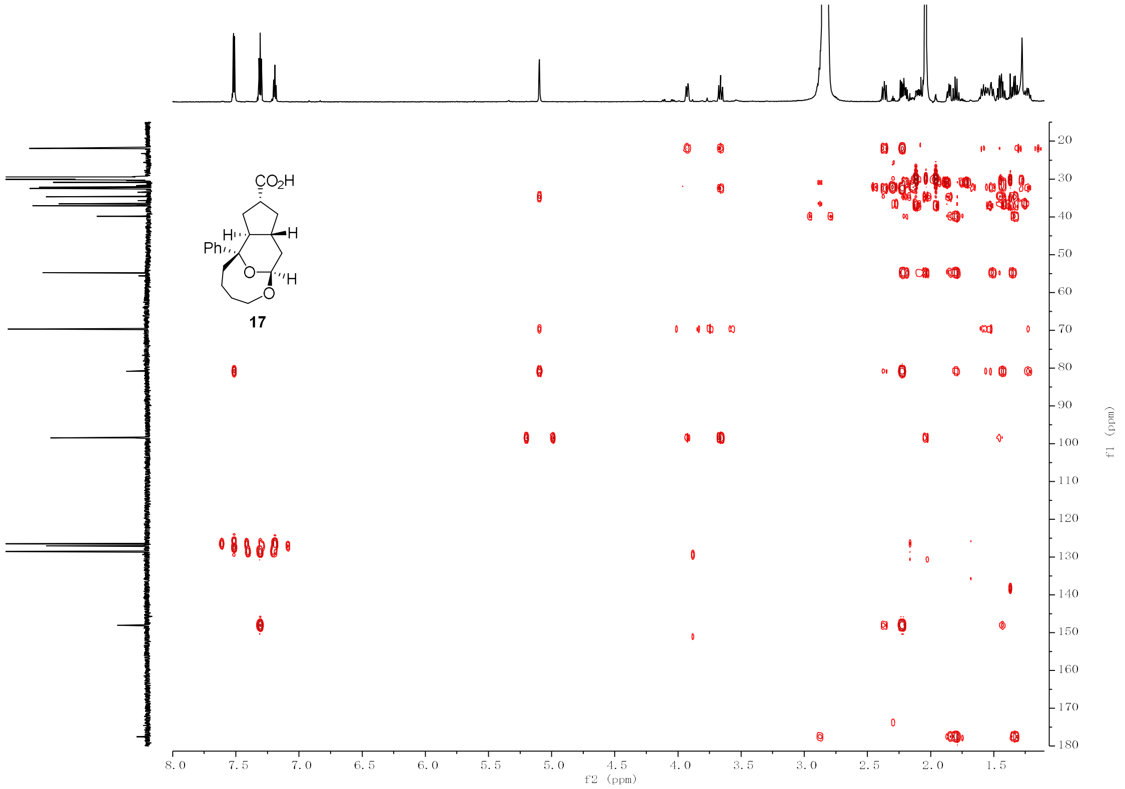

Supplementary Figure 350. ^{1}H-^{1}H COSY for 19 in acetone-d$_6$.

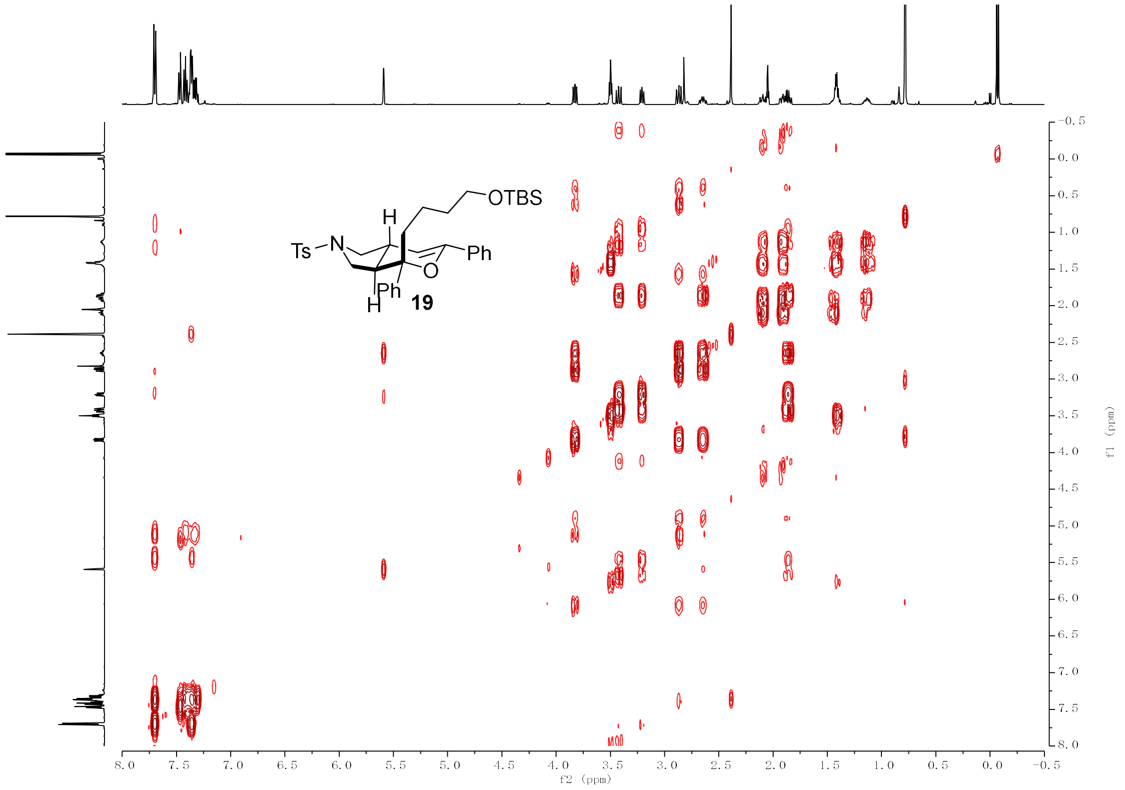

Supplementary Figure 351. ^{1}H-^{1}H ROESY for 19 in acetone-d$_6$.

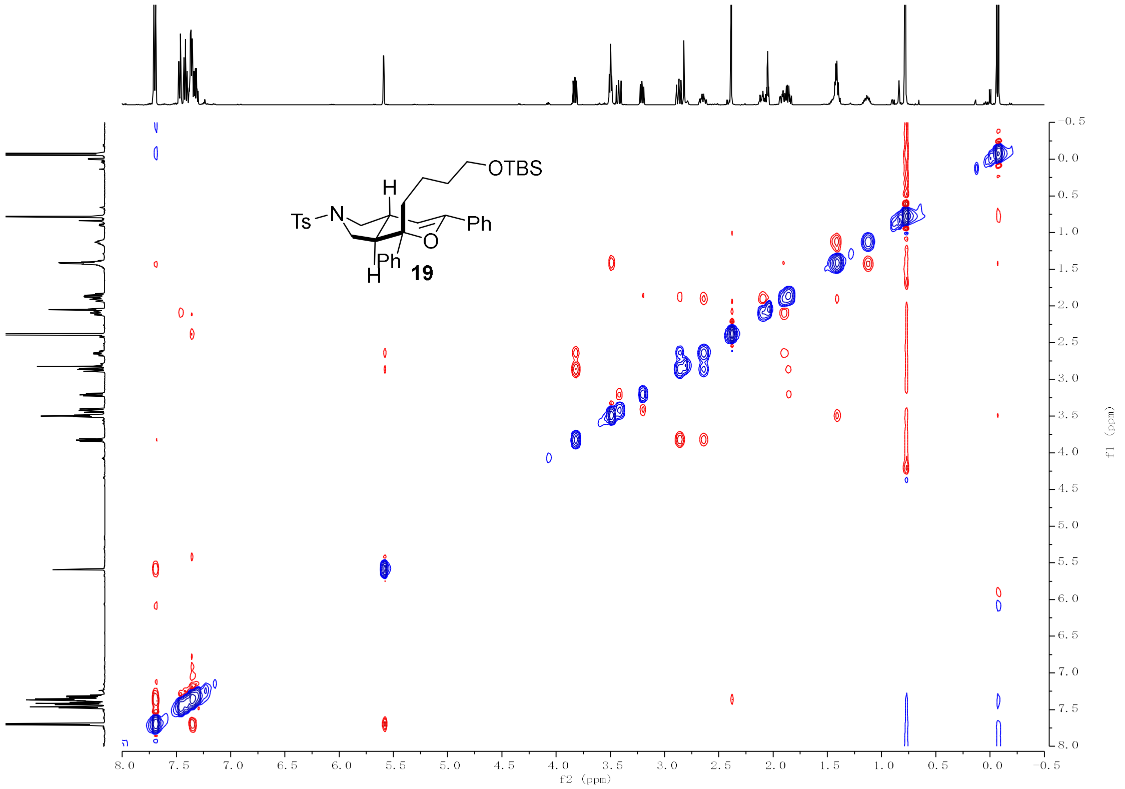

Supplementary Figure 352. ^{1}H-^{13}C HSQC for 19 in acetone-d_6.

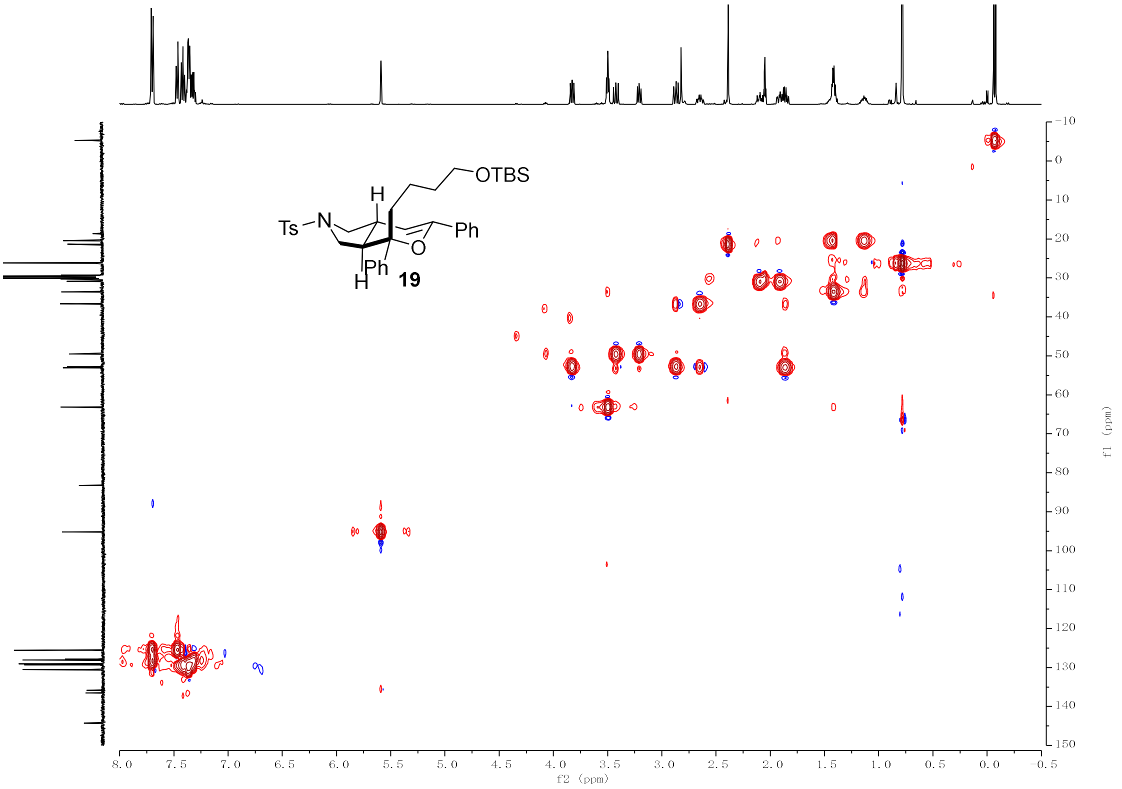

Supplementary Figure 353. ^{1}H-^{13}C HMBC for 19 in acetone-d_6.

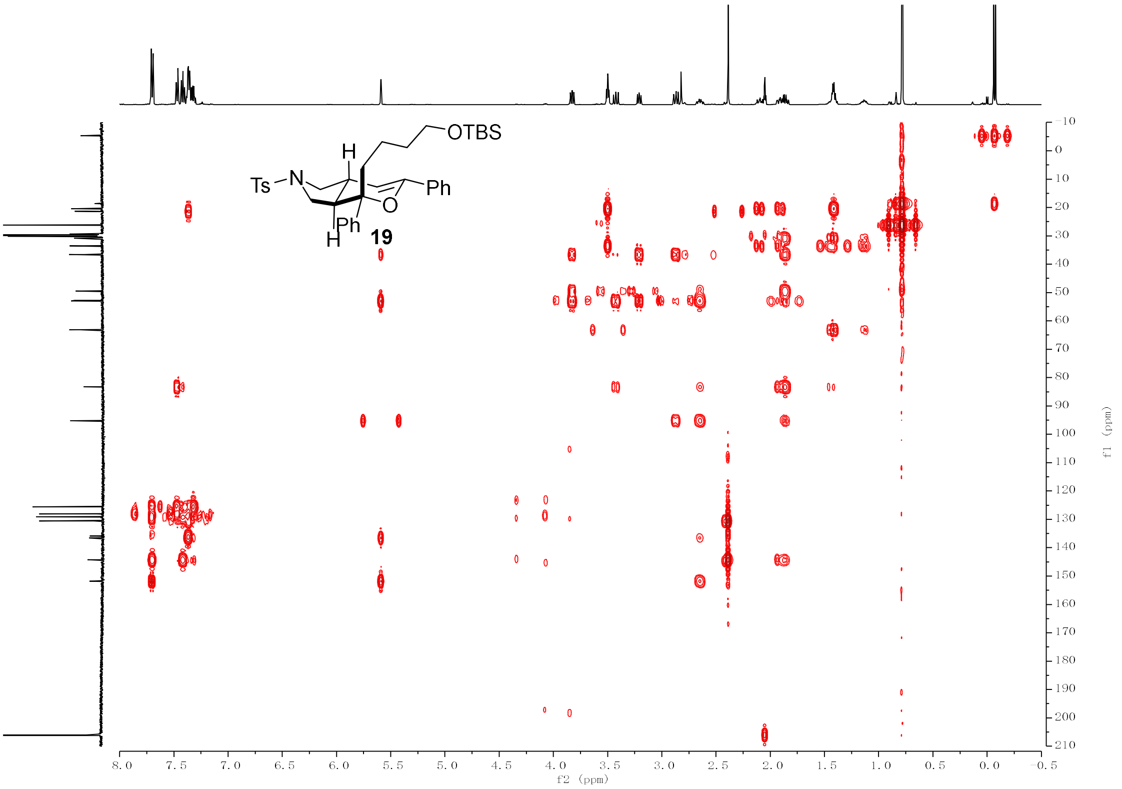

4. Supplementary References

1. Kohn, W., Sham, L. J. Self-Consistent Equations Including Exchange and Correlation Effects. *Phys. Rev.* **140**, A1133-A1138 (1965).
2. Frisch, M. J., Trucks, G. W., Schlegel, H. B., Scuseria, G. E., Robb, M. A., Cheeseman, J. R., Scalmani, G., Barone, V., Mennucci, B., Petersson, G. A., Nakatsuji, H., Caricato, M., Li, X., Hratchian, H. P., Izmaylov, A. F., Bloino, J., Zheng, G., Sonnenberg, J. L., Hada, M., Ehara, M., Toyota, K., Fukuda, R., Hasegawa, J., Ishida, M., Nakajima, T., Honda, Y., Kitao, O., Nakai, H., Vreven, T., Montgomery, J. A., Jr., Peralta, J. E., Ogliaro, F., Bearpark, M., Heyd, J. J., Brothers, E., Kudin, K. N., Staroverov, V. N., Keith, T., Kobayashi, R., Normand, J., Raghavachari, K., Rendell, A., Burant, J. C., Iyengar, S. S., Tomasi, J., Cossi, M., Rega, N., Millam, J. M., Klene, M., Knox, J. E., Cross, J. B., Bakken, V., Adamo, C., Jaramillo, J., Gomperts, R., Stratmann, R. E., Yazyev, O., Austin, A. J., Cammi, R., Pomelli, C., Ochterski, J. W., Martin, R. L., Morokuma, K., Zakrzewski, V. G., Voth, G. A., Salvador, P., Dannenberg, J. J., Dapprich, S., Daniels, A. D., Farkas, O., Foresman, J. B., Ortiz, J. V., Cioslowski, J., Fox, D. J. *Gaussian 09, Revision C. 01; Gaussian, Inc., Wallingford CT*, 2010.
3. Stephens, P. J., Devlin, F. J., Chabalowski, C. F. & Frisch, M. J. *Ab Initio* Calculation of Vibrational Absorption and Circular Dichroism Spectra Using Density Functional Force Fields. *J. Phys. Chem.* **98**, 11623-11627 (1994).
4. Sang‐Aroon, W., Ruangpornvisuti, V. Determination of aqueous acid‐dissociation constants of aspartic acid using PCM/DFT method. *Int. J. Quantum Chem* **108**, 1181-1188 (2008).
5. Mennucci, B., Tomasi, J. Continuum Solvation Models: A New Approach to the Problem of Solute's Charge Distribution and Cavity Boundaries. *J. Chem. Phys.* **106**, 5151-5158 (1997).
6. Marenich, A. V., Cramer, C. J. & Truhlar, D. G. Universal Solvation Model Based on Solute Electron Density and on a Continuum Model of the Solvent Defined by the Bulk Dielectric Constant and Atomic Surface Tensions. *J. Phys. Chem. B* **113**, 6378-6396 (2009).
7. Zhao, Y., Truhlar, D. G. The M06 Suite of Density Functionals for Main Group Thermochemistry, Thermochemical Kinetics, Noncovalent Interactions, Excited States, and Transition Elements: Two New Functionals and Systematic Testing of Four M06-Class Functionals and 12 Other Functionals. *Theor. Chem. Acc.* **120**, 215-241 (2008).
8. Weigend, F., Ahlrichs, R. Balanced basis sets of split valence, triple zeta valence and quadruple zeta valence quality for H to Rn: Design and assessment of accuracy. *Phys. Chem. Chem. Phys.* **7**, 3297-3305 (2005).
9. Grimme, S., Antony, J., Ehrlich, S. & Krieg, H. A consistent and accurate ab initio parametrization of density functional dispersion correction (DFT-D) for the 94 elements H-Pu. *J. Chem. Phys.* **132**, 154104 (2010).
10. Goerigk, L., Grimme, S. A thorough benchmark of density functional methods for general main group thermochemistry, kinetics, and noncovalent interactions. *Phys. Chem. Chem. Phys.* **13**, 6670-6688 (2011).
